# SNRPC promotes hepatocellular carcinoma cell motility by inducing epithelial‐mesenchymal transition

**DOI:** 10.1002/2211-5463.13175

**Published:** 2021-05-12

**Authors:** Yuanping Zhang, Jiliang Qiu, Dinglan Zuo, Yichuan Yuan, Yuxiong Qiu, Liang Qiao, Wei He, Binkui Li, Yunfei Yuan

**Affiliations:** ^1^ Department of Liver Surgery Sun Yat‐sen University Cancer Center Guangzhou China; ^2^ State Key Laboratory of Oncology in South China Sun Yat‐sen University Cancer Center Guangzhou China; ^3^ Collaborative Innovation Center for Cancer Medicine Guangzhou China

**Keywords:** functional network analysis, HCC, prognosis, SNRPC

## Abstract

The therapeutic outcome of hepatocellular carcinoma (HCC) remains unsatisfactory because of poor response and acquired drug resistance. To better elucidate the molecular mechanisms of HCC, here we used three Gene Expression Omnibus datasets to identify potential oncogenes, and thereby identified small nuclear ribonucleoprotein polypeptide C (SNRPC). We report that SNRPC is highly up‐regulated in HCC tissues as determined using immunohistochemistry assays of samples from a cohort of 224 patients with HCC, and overexpression of SNRPC was correlated with multiple tumors, advanced stage, and poor outcome. Kaplan–Meier analysis confirmed that patients with high SNRPC expression exhibited shorter survival in four independent HCC cohorts (all *P* < 0.05). Furthermore, *SNRPC* mutations are significantly more frequent in HCC tissues than in normal liver tissues and are an early event in the development of HCC. Functional network analysis suggested that *SNRPC* is linked to the regulation of ribosome, spliceosome, and proteasome signaling. Subsequently, gain‐ and loss‐of‐function assays showed that *SNRPC* promotes the motility and epithelial–mesenchymal transition of HCC cells *in vitro*. *SNRPC* expression was negatively correlated with the infiltration of CD4^+^ T cells, macrophage cells, and neutrophil cells (all *P* < 0.05), as determined by analyzing the TIMER (Tumor IMmune Estimation Resource) database. In conclusion, our findings suggest that *SNRPC* has a potential role in epithelial–mesenchymal transition and motility in HCC.

## Graphical abstract

Small nuclear ribonucleoprotein polypeptide C (SNRPC) was highly up‐regulated in hepatocellular carcinoma (HCC) tissues and correlated with poor outcome in patients with HCC. Functional network analysis suggested that *SNRPC* was linked to the regulation of ribosome, spliceosome and proteasome signaling. SNRPC promoted the motility and epithelial–mesenchymal transition of HCC cells *in vitro*.

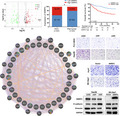

**Abbreviations**
CNV: copy number variation
DEG: differentially expressed gene
DFS: disease‐free survival
EMT: epithelial‐mesenchymal transition
FC: fold change
FDR: false discovery rate
GEO: Gene Expression Omnibus
GEPIA: Gene Expression Profiling Interactive Analysis
GO: Gene Ontology
GSEA: gene set enrichment analysis
HCC: hepatocellular carcinoma
HR: hazard ratio
IHC: immunohistochemistry
KEGG: Kyoto Encyclopedia of Genes and Genomes
miR: microRNA
OS: overall survival
PPI: protein–protein interaction
snRNP: small nuclear ribonucleoprotein
SNRPC: small nuclear ribonucleoprotein polypeptide C
TCGA: The Cancer Genome Atlas
TF: transcription factor
TIMER: Tumor IMmune Estimation Resource
TNM: tumor‐node‐metastasis

Hepatocellular carcinoma (HCC) is one of the most common cancers in Asia [1]. A large population‐based study also showed that the incidence of HCC increased rapidly by 2% to 3% annually from 2007 to 2016 in the United States [2]. Mortality rates also increased over the past decade for HCC, with a 5‐year survival rate of <20% [3]. New systemic therapy has changed drastically after the introduction of immune checkpoint inhibitors and molecularly targeted agents. However, the therapeutic outcome is still unsatisfactory due to poor response and acquired drug resistance [4]. Ongoing intense research efforts have been made to elucidate the pathogenesis of HCC. Our earlier studies have elucidated the biological features in HCC [5, 6]. However, the complex mechanism underlying the development and progression of HCC remains poorly understood. Therefore, the identification of new biomarkers may provide valuable insights into the understanding and treatment of patients with HCC.

Recently, high‐throughput assays and bioinformatic tools have been widely used to screen for novel differentially expressed genes (DEGs) involved in cancers [7, 8]. However, results based on only single microarray data have the limitation of a high false‐positive rate. To overcome the limitation, we used a strategy with multiple validation or microarray datasets. Thus, we tried to identify robust DEGs across three datasets from the Gene Expression Omnibus (GEO) and validated in four independent cohorts. Finally, small nuclear ribonucleoprotein (snRNP) polypeptide C (SNRPC) was identified as a potential oncogene in our study.

*SNRPC*, located on human chromosome 6p21.31, encodes a specific protein component of the U1 snRNP particle, which is essential for the splicing process [9]. An earlier study illustrated that SNRPC contributed to sex bias in systemic autoimmune diseases [10]. Through regulating alternative splicing of the coactivator‐associated arginine methyltransferase, SNRPC plays a role in spinal muscular atrophy pathogenesis [11]. However, the biological role and mechanism of SNRPC are unknown in cancer.

In this study, we demonstrated that SNRPC was overexpressed and correlated with poor survival in HCC. Using public databases, we explored genomic alterations and functional networks related to *SNRPC*. We further confirmed that SNRPC could promote HCC cellular motility *in vitro*. Our study demonstrates that *SNRPC* is a novel oncogene that is possibly involved in promoting HCC cell motility.

## Materials and methods

### Identification of DEGs

Three gene expression datasets (GSE65372, GSE39791 and GSE36376), which included more than 350 HCC samples with access to full clinicopathological data, were selected and downloaded from GEO (http://www.ncbi.nlm.nih.gov/geo) [12, 13, 14]. The DEGs between HCC and noncancerous samples were screened using GEO2R (http://www.ncbi.nlm.nih.gov/geo/geo2r). The adjusted *P* values and Benjamini and Hochberg false discovery rate (FDR) were applied to provide a balance between the discovery of statistically significant genes and the limitation of false positives. An adjusted *P* value < 0.01 was considered statistically significant to screen DEGs.

### Immunohistochemistry

Immunohistochemistry (IHC) western blot was performed as in our previous study [15]. The primary antibody was SNRPC (dilution 1 : 100; Santa Cruz Biotechnology, Santa Cruz, CA, USA). Histological and IHC evaluations were independently performed by two pathologists, who were blind to the clinicopathological outcomes of the patients. The intensity of the staining was graded as follows: 0 (negative), 1 (weak), 2 (moderate) or 3 (strong). The percentage of positive cells was graded as follows: 1 (0–25%), 2 (26–50%), 3 (51–75%) or 4 (76–100%). The expression of SNRPC was defined according to the final one obtained from the grade of intensity multiplied by the score of the percentage of positive cells: low SNRPC expression (0–5) and high SNRPC expression (6–12).

### Database analysis

Gene Expression Profiling Interactive Analysis (GEPIA) (http://gepia.cancer‐pku.cn) and HCCDB (http://lifeome.net/database/hccdb/home.html) databases were used to analyze overall survival (OS) and disease‐free survival (DFS). UALCAN (http://ualcan.path.uab.edu) facilitates analyses of various tumor subgroups based on individual cancer stages, tumor grade or other clinicopathological features. The cBioPortal (http://cbioportal.org) enables us to explore mutation, copy number variation (CNV) and mRNA expression of *SNRPC* and to link these to clinical outcomes. The mRNA expression and DNA copy number of *SNRPC* for HCC were analyzed within the Oncomine 4.5 database (https://www.oncomine.org). *SNRPC* expression was assessed in HCC relative to its expression in normal tissue, and differences associated with a *P* < 0.05 and a fold change (FC) > 1.5 were considered significant in Oncomine. The correlation of DEGs and *SNRPC* was analyzed with LinkedOmics (http://www.linkedomics.org) and presented using volcano plots and heatmaps. The function modules of LinkedOmics were used to perform analysis of Gene Ontology (GO) biological process, Kyoto Encyclopedia of Genes and Genomes (KEGG) pathways, kinase‐target enrichment, microRNA (miR)‐target enrichment and transcription factor (TF)‐target enrichment by the gene set enrichment analysis (GSEA). The rank criterion was FDR < 0.05, and 1000 simulations were performed. We used GeneMANIA (http://www.genemania.org) to construct a protein–protein interaction (PPI) network. Subsequently, the 30 genes most significantly correlated with SNRPC were selected with the use of GSEA. The prognostic value of screened target miRNAs was analyzed using the Kaplan–Meier plotter portal (http://kmplot.com/analysis). We employed the TIMER (Tumor IMmune Estimation Resource) database (https://cistrome.shinyapps.io/timer/) to deduce the abundance of tumor‐infiltrating immune cells from gene expression profiles [16].

### Human tissue specimens and HCC tissue microarray

From March 2010 to November 2013, a total of 224 patients who underwent radical resection and did not receive other anti‐HCC treatments at Sun Yat‐sen University Cancer Center were included in the study. The clinicopathological features of the enrolled patients are in Table 1. The study was conducted according to the standards of the 1975 Declaration of Helsinki. The protocol of this study was approved by the Ethics Committee of Sun Yat‐sen University Cancer Center. Written informed consents were obtained from all study subjects.

**Table 1:** Correlation of clinicopathological parameters and SNRPC expression in tumor nucleus in our TMA cohort. AFP, alpha‐fetoprotein; ALB, albumin; ALT, alanine aminotransferase; AST, aspartate transaminase; HBsAg, hepatitis B surface antigen; TBIL, total bilirubin.

Characteristics	Low SNRPC (*n* = 128)	High SNRPC (*n* = 96)	*P* value
Age, ≤60 : >60 years	98 : 30	72 : 24	0.875
Sex, male : female	111 : 17	88 : 8	0.244
HBsAg, yes : no	113 : 15	89 : 7	0.271
ALB, ≤35 : >35 g L^−1^	7 : 121	7 : 89	0.577
TBIL, >17.1 : ≤17.1 μmol L^−1^	25 : 103	29 : 67	0.064
ALT, >40 : ≤40, U L^−1^	49 : 79	38 : 58	0.843
AST, >45 : ≤45, U L^−1^	37 : 91	34 : 62	0.300
AFP, >400 : ≤400 ng mL^−1^	41 : 82	38 : 52	0.185
Tumor number (multiple : solitary)	17 : 111	27 : 69	0.006*
Tumor size, >5 : ≤5 cm	60 : 68	49 : 47	0.537
Tumor encapsulation, incomplete : complete	78 : 50	66 : 30	0.227
Vascular invasion, yes : no	31 : 97	34 : 62	0.068
Cirrhosis, yes : no	78 : 50	60 : 36	0.812
TNM stage, III : I–II	36 : 92	46 : 50	0.003*

* *P* < 0.05.

### Cell lines and cell culture

Human HCC cell lines (PLC‐8024, Huh7, Hep3B and SK‐Hep1) were purchased from the Shanghai Cell Bank of the Chinese Academy of Sciences (Shanghai, China) with short tandem repeat appraisal certificates. Cells were maintained in Dulbecco's modified Eagle’s medium (Thermo Fisher, Waltham, MA, USA) supplemented with 10% FBS (Gibco, Grand Island, NY, USA) at 37 °C in 5% CO_2_.

### Quantitative real‐time PCR and Western blot

Quantitative real‐time PCR and western blot were performed as previously described [15]. Primers were as follows: *SNRPC* forward: 5′‐GCAGTGGAAGGAAACACAAAGAG‐3′ and reverse: 5′‐GGAGCAGAGAATGGAGTAGGAG‐3′; *GAPDH* forward: 5′‐ GTCTCCTCTGACTTCAACAGCG‐3′ and reverse: 5′‐ACCACCCTGTTGCTGTAGCCAA‐3′. All reactions were run in triplicate. The antibodies used in our study were SNRPC (dilution 1 : 1000; Cat no. sc‐101549, RRID:AB_2295245; Santa Cruz Biotechnology), GAPDH, E‐cadherin and Vimentin (dilution 1 : 2000; Cell Signaling Technology, Danvers, MA, USA).

### *In vitro* cell proliferation assay and migration assay

For cell proliferation assay, we performed the Cell Counting Kit‐8 and colony formation assay. For cell migration assay, we performed Transwell and wound healing assay. All of the earlier‐mentioned experiments were performed as in our previous study [15].

### Statistical analysis

The experiments were repeated at least three times independently, and the measured data were represented as the mean ± standard deviation. Binary variables were compared using the chi‐square test, and ordinal categorical variables were compared by the Kruskal–Wallis test. Survival curves were constructed using the Kaplan–Meier method and analyzed by the log‐rank test. Significant prognostic factors found by univariate analysis were entered into a multivariate analysis using the Cox proportional hazards model. All analyses were two‐sided, and *P* < 0.05 was considered significant. Statistical analyses were performed using the statistical package for social science version 24.0 (SPSS Inc., Chicago, IL, USA) and graphpad prism 7.0 software (GraphPad, Inc., La Jolla, CA, USA).

## Results

### Identification of DEGs in HCC

**Fig. 1:**
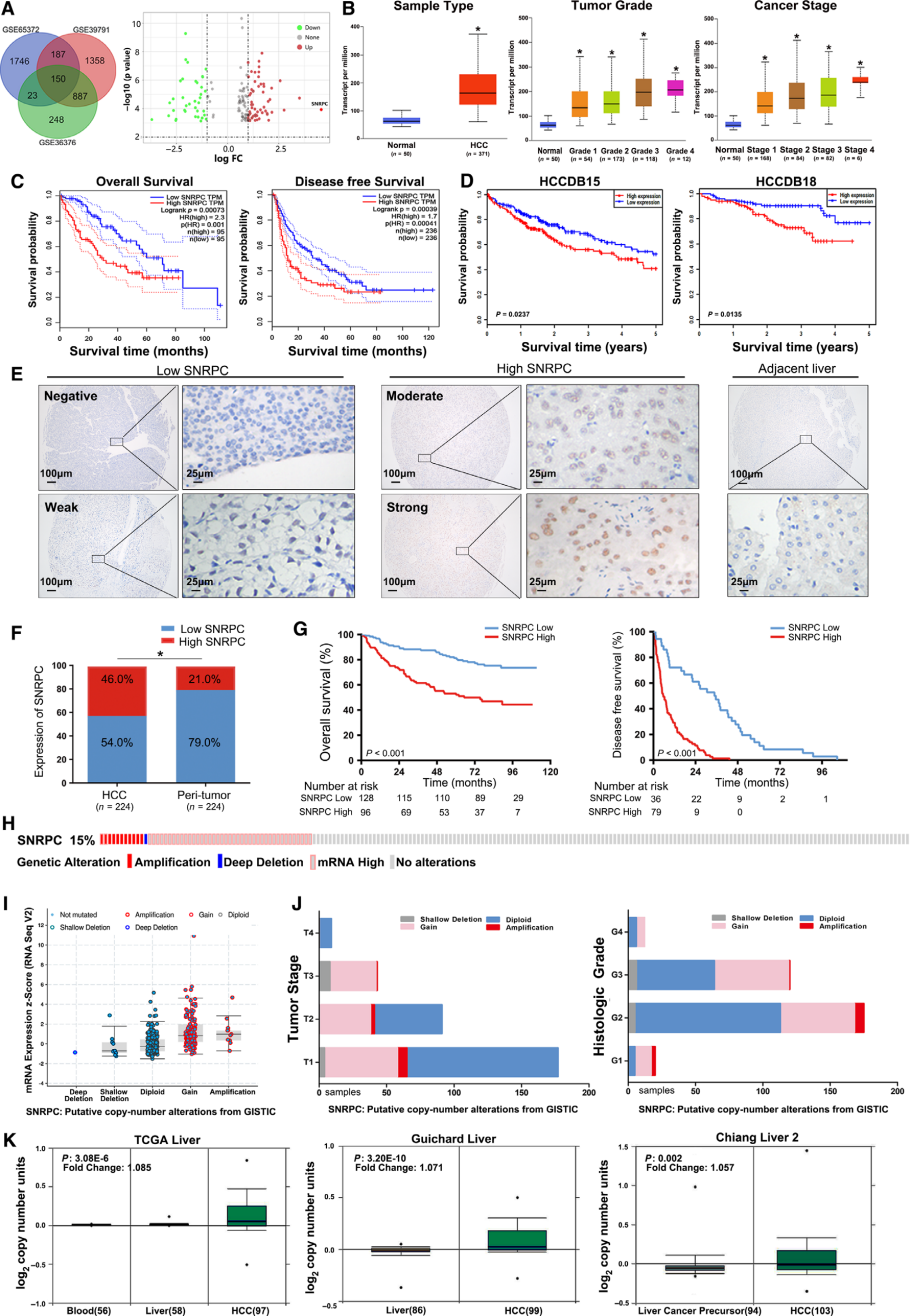
SNRPC correlates with poor prognosis in patients with HCC and presents with a higher CNV in HCC tissues compared with normal control tissues. (A) Venn diagram. DEGs were selected among the mRNA expression profiling sets GSE65372, GSE39791 and GSE36376. *SNRPC* transcription in the subgroups of patients with HCC stratified by (B) sample type, tumor grade and cancer stage, analyzed by UALCAN (from the TCGA database). Data are mean ± standard error. *P < 0.05. OS and DFS of SNRPC in (C) GEPIA and (D) HCCDB databases. (E) The images of different levels of SNRPC expression in HCC (left) and adjacent noncancerous liver tissues (right) detected by immunohistochemical staining (IHC). Scale bars: 100 or 25 μm. (F) SNRPC expression in HCC and peritumor tissues according to IHC scores: low SNRPC (0–5) and high SNRPC (6–12). *P < 0.05, based on the Wilcoxon matched pairs test. (G) Kaplan–Meier survival analysis of OS and DFS for patients with high and low SNRPC expression. (H) OncoPrint of *SNRPC* alterations in LIHC (cBioPortal). The different types of genetic alterations are highlighted in different colors. (I) *SNRPC* expression in different *SNRPC* CNV groups. *SNRPC* gain group has a significantly higher expression level. (J) Distribution of *SNRPC* CNV frequency in different tumor stage and histological grade subgroups. (K) *SNRPC* copy number in TCGA Liver, Guichard Liver and Chiang Liver 2 datasets, respectively.

The array data for GSE65372, GSE39791 and GSE36376 consisted of 39 HCC versus 15 controls, 72 HCC versus 72 control subjects and 240 HCC versus 193 control subjects, respectively. After standardization, 150 DEGs were selected from the overlap of three cohorts (2106 in GSE65372, 2582 in GSE39791 and 1309 in GSE36376, respectively; Fig. 1A). The overlap among the three datasets consisted of 44 down‐regulated genes and 106 up‐regulated genes between liver cancer tissues and noncancerous tissues (Table S1). Among the 150 DEGs, *SNRPC* was the most significantly different oncogene, with a logFC of 4.57 (Fig. 1A). We thus selected SNRPC for further analyses.

### SNRPC expression is elevated and associated with HCC survival

The expression of SNRPC was analyzed in HCC samples. *SNRPC* expression was up‐regulated in HCC versus healthy control subjects and was positively correlated with advanced tumor grade and tumor stage, which were obtained from The Cancer Genome Atlas (TCGA) datasets (Fig. 1B).

Survival analysis was performed using GEPIA to determine the prognostic value of *SNRPC* in patients with HCC. Compared with the low expression group, the high *SNRPC* expression group had significantly poorer OS (*P* = 7.3E−4) and DFS (*P* = 3.9E−4; Fig. 1C). Similarly, the high *SNRPC* expression group had significantly shorter OS versus the low expression group in the two independent cohorts (*P* = 0.0237 for HCCDB15 and *P* = 0.0135 for HCCDB18; Fig. 1D).

Next, we examined the expression of SNRPC in a tissue microarray (TMA) cohort consisting of 224 patients with HCC from the Sun Yat‐sen University Cancer Center. SNRPC was primarily located in the nucleus and significantly up‐regulated in HCC tissues compared with the paired adjacent liver tissues (Fig. 1E,F). According to the IHC score, patients with HCC were divided into two groups: high SNRPC expression (*n* = 96) and low SNRPC expression (*n* = 128). Statistical analysis showed that high SNRPC expression was associated with a higher number of tumors and a more advanced tumor‐node‐metastasis (TNM) stage (Table 1). Kaplan–Meier analysis confirmed that patients with high SNRPC expression had shorter OS and DFS (both *P* < 0.001; Fig. 1G). Multivariate analysis showed that SNRPC expression was an independent prognostic factor for OS [hazard ratio (HR) = 2.103, *P* = 0.002] and DFS (HR = 4.343, *P* < 0.001) in patients with HCC (Tables 2 and 3).

**Table 2:** Univariate and multivariate Cox regression analyses for OS in our TMA cohort. AFP, alpha‐fetoprotein; ALB, albumin; ALT, alanine aminotransferase; AST, aspartate transaminase; CI, confidence interval; HBsAg, hepatitis B surface antigen; SNRPC, expression in tumor nucleus; TBIL, total bilirubin.

Variables	Univariate analysis			Multivariate analysis		
	HR	95% CI	*P* value	HR	95% CI	*P* value
SNRPC (high : low)	2.792	1.791–4.350	<0.001*	2.103	1.324–3.340	0.002*
Age (≤51 : >51 years)	0.977	0.635–1.502	0.915			
Sex (male : female)	2.834	1.037–7.741	0.042*	3.212	1.159–8.902	0.025*
HBsAg (positive : negative)	0.768	0.397–1.488	0.434			
ALB (≤35 : >35 g L^−1^)	1.412	0.651–3.063	0.383			
TBIL (>17.1 : ≤17.1 μmol L^−1^)	1.157	0.710–1.883	0.559			
ALT (>40 : ≤40 U L^−1^)	1.429	0.927–2.201	0.106			
AST (>45 : ≤45 U L^−1^)	1.995	1.292–3.081	0.002*	1.857	1.192–2.894	0.006*
AFP (>400 : ≤400 ng mL^−1^)	1.063	0.676–1.671	0.792			
Tumor number (multiple : solitary)	2.742	1.729–4.349	<0.001*	2.091	1.290–3.389	0.003*
Tumor size (>5 : ≤5 cm)	1.448	0.939–2.232	0.094			
Tumor encapsulation (incomplete : complete)	1.108	0.705–1.740	0.657			
Vascular invasion (yes : no)	2.485	1.611–3.835	<0.001*	2.451	1.575–3.814	<0.001*
Cirrhosis (yes : no)	0.897	0.578–1.392	0.628			
TNM stage (III : I–II)	2.997	1.939–4.632	<0.001*	1.331	0.567–3.127	0.511

* *P* < 0.05.

**Table 3:** Univariate and multivariate Cox regression analyses for DFS in our TMA cohort. AFP, alpha‐fetoprotein; ALB, albumin; ALT, alanine aminotransferase; AST, aspartate transaminase; CI, confidence interval; HBsAg, hepatitis B surface antigen; SNRPC, expression in tumor nucleus; TBIL, total bilirubin.

Variables	Univariate analysis			Multivariate analysis		
	HR	95% CI	*P* value	HR	95% CI	*P* value
SNRPC (high : low)	4.875	2.922–8.131	<0.001*	4.343	2.544–7.411	<0.001*
Age (≤51 : >51 years)	0.884	0.609–1.281	0.514			
Sex (male : female)	1.429	0.743–2.749	0.285			
HBsAg (positive : negative)	0.933	0.433–2.011	0.859			
ALB (≤35 : >35 g L^−1^)	1.267	0.586–2.737	0.548			
TBIL (>17.1 : ≤17.1 μmol L^−1^)	1.171	0.776–1.767	0.453			
ALT (>40 : ≤40 U L^−1^)	0.945	0.647–1.380	0.769			
AST (> 45 vs ≤ 45 U L^−1^)	1.075	0.731–1.583	0.713			
AFP (>400 : ≤400 ng mL^−1^)	1.328	0.903–1.953	0.150			
Tumor number (multiple : solitary)	2.273	1.480–3.492	<0.001*	1.967	1.274–3.038	0.002*
Tumor size (>5 : ≤5 cm)	1.172	0.810–1.696	0.400			
Tumor encapsulation (incomplete : complete)	0.808	0.637–1.023	0.077			
Vascular invasion (yes : no)	1.637	1.084–2.474	0.019*	1.233	0.809–1.877	0.330
Cirrhosis (yes : no)	0.665	0.447–0.989	0.004*	0.885	0.588–1.333	0.560
TNM stage (III : I–II)	2.100	1.425–3.094	<0.001*	1.323	0.603–2.903	0.484

* *P* < 0.05.

### Genomic alterations of SNRPC in HCC

We used the cBioPortal to determine the types and frequency of *SNRPC* alterations in HCC based on DNA sequencing data in the TCGA database. *SNRPC* alteration was detected in 53 of the 349 (15.18%) patients (Fig. 1H), including mRNA up‐regulation in 41 cases (11.75%), amplification in 8 cases (2.29%), deep deletion in 1 case (0.29%) and multiple alterations in 2 cases (0.57%). Gain (14.04%) was the most common type of *SNRPC* CNV in HCC (Fig. 1I). The frequency distribution of *SNRPC* CNV patients at different stages and grade groupings is presented in Fig. 1J, suggesting that *SNRPC* CNV alteration is an early event in the trajectories of HCC. As shown in Fig. 1K, independent cohorts form the Oncomine database, and the CNV of *SNRPC* in HCC tissues was significantly higher than those in normal controls or liver cancer precursors (*P* ˂ 0.001). This confirmed the high rate of *SNRPC* CNV alteration in HCC.

### SNRPC coexpression networks in HCC

**Fig. 2:**
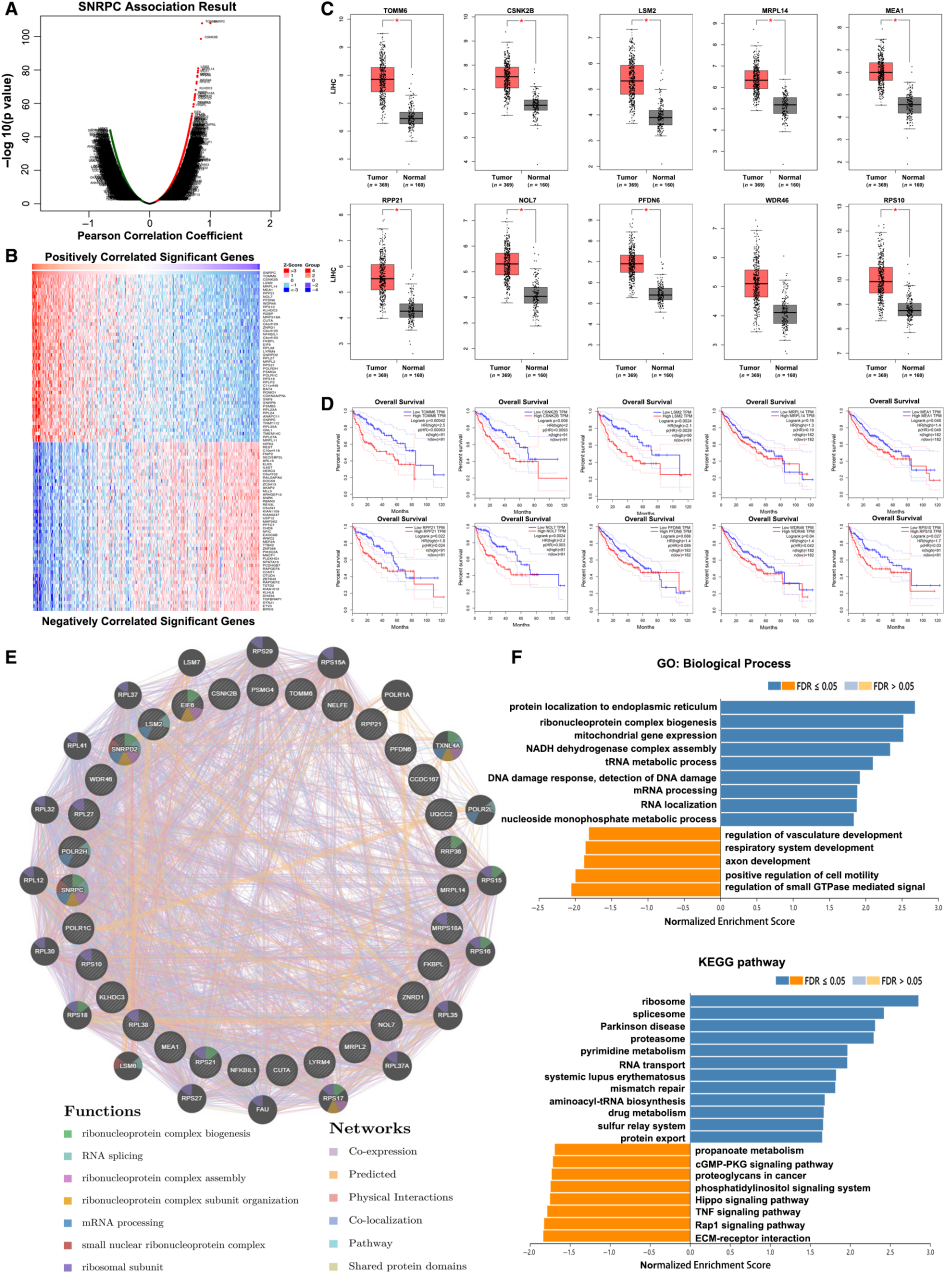
Coexpression genes and PPI analysis of SNRPC in HCC. (A) SNRPC coexpression genes in HCC (LinkedOmics). (B) Heatmaps showing the top 50 genes positively and negatively correlated with SNRPC in HCC. Red indicates positively correlated genes, and green indicates negatively correlated genes. (C) The top 10 most correlated with SNRPC genes were highly expressed in tumor tissues, analyzed by GEPIA. (D) OS of the top 10 most correlated with SNRPC genes from GEPIA. (E) PPI network and functional analysis of the top 30 genes correlated with SNRPC (GeneMANIA). Different colors of the network edge indicate the bioinformatics methods applied. The different colors for the network nodes indicate the biological functions of the set of enrichment genes. (F) The function module of LinkedOmics performs analysis of GO biological processes (BPs) and KEGG pathways among SNRPC coexpression genes in the LIHC cohort.

To gain a biological insight into SNRPC in HCC, we used the function module of LinkedOmics to examine *SNRPC* coexpression genes in the liver hepatocellular carcinoma (LIHC) cohort based on the TCGA datasets. As shown in Fig. 2A, 3419 genes (dark red dots) showed significant positive correlations with *SNRPC*, whereas 5047 (dark green dots) were negative with *SNRPC* (FDR < 0.001). The earlier selected coexpressed genes are detailed in Table S2. The top 50 significant genes positively and negatively correlated with *SNRPC* are shown in a heatmap (Fig. 2B). The 10 genes most correlated with *SNRPC* were analyzed based on the expression in HCC tissues. Compared with normal liver tissues, these 10 genes were highly expressed in tumors (Fig. 2C). Except for the mitochondrial ribosomal protein L14 (*MRPL14*) and prefoldin subunit 6 (*PFDN6*), the other eight genes showed negative correlations with survival (Fig. 2D).

With the use of GeneMANIA, the PPI network revealed correlation among the top 30 most significantly coexpressed genes (Fig. 2E). The enriched functions of SNRPC were mainly involved in the ribonucleoprotein complex's biogenesis, assembly and subunit organization, RNA splicing and mRNA processing. Analysis of significantly enriched GO terms indicated that these genes were mainly associated with the endoplasmic reticulum, ribosome and mitochondria (Fig. 2F). Similarly, the KEGG pathway analysis showed enrichment in ribosome signaling, spliceosome signaling and proteasome signaling pathways (Table S3).

### SNRPC networks of kinase, miRNA or TF targets in HCC

**Fig. 3:**
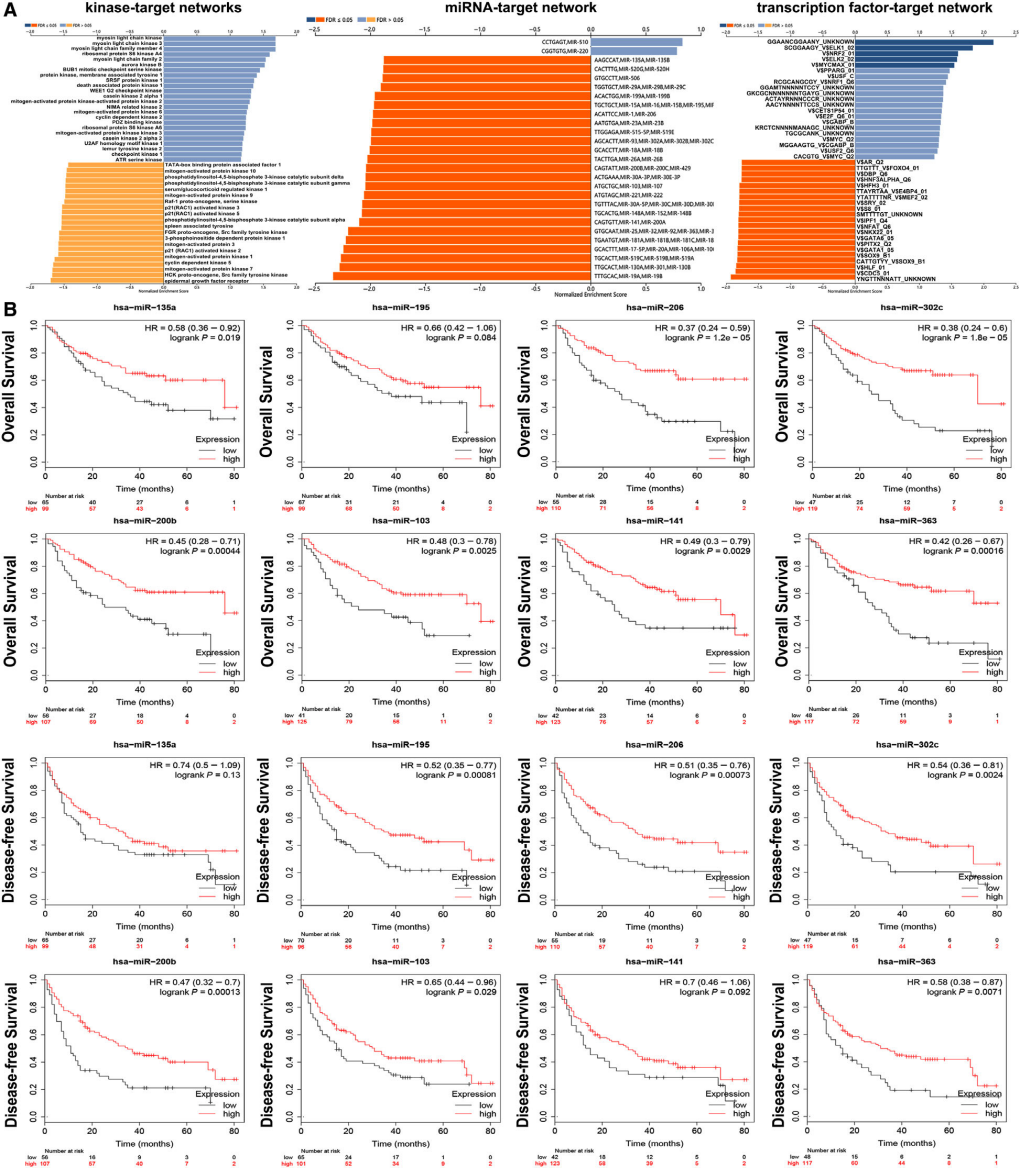
The kinase‐, miRNA‐ and TF‐target networks of SNRPC in HCC. (A) Function module of LinkedOmics performs analysis of kinase‐target enrichment, miR‐target enrichment and TF‐target enrichment among SNRPC coexpression genes in the LIHC cohort. (B) OS and DFS of the top eight most correlated with SNRPC miRNAs in the Kaplan–Meier plotter portal.

To further explore the mechanisms of *SNRPC* in HCC, we analyzed the kinase, miRNA and TF target networks of *SNRPC* coexpressed gene sets generated by GSEA (Tables [Link], [Link], [Link], [Link]). No significant kinase‐target network was enriched by GSEA for *SNRPC* coexpressed genes (Fig. 3A). The miRNA‐target networks showed that SNRPC was negatively regulated by miR‐135a, miR‐195, miR‐206, miR‐302c, miR‐200b, miR‐103, miR‐141 and miR‐363. Further Kaplan–Meier plots showed that all these miRNAs were positively related to OS or DFS in patients with HCC (Fig. 3B). These results also demonstrated that overexpressed miRNA can improve the prognosis of patients by inhibiting the expression of SNRPC. The most significant enrichments of already known TFs were HFH3_01 (gene symbol: *FOXI1*) and E4BP4_01 (*NFIL3*; Table 4).

**Table 4:** The kinase‐, miRNA‐ and TF‐target networks of SNRPC in HCC. FDR, FDR from Benjamini and Hochberg from GSEA; LeadingEdgeNum, the number of leading‐edge genes; V$, the annotation found in Molecular Signatures Database for TF.

Category	Geneset	LeadingEdgeNum	FDR
Kinase target	Kinase_CDK5	29	0.059058
	Kinase_MAPK1	77	0.064933
	Kinase_MAPK7	13	0.065269
	Kinase_MYLK	4	0.068161
	Kinase_MYLK3	4	0.068161
miRNA target	AAGCCAT, MIR‐135A, MIR‐135B	129	0
	CACTTTG, MIR‐520G, MIR‐520H	97	0
	GTGCCTT, MIR‐506	229	0
	TGGTGCT, MIR‐29A, MIR‐29B, MIR‐29C	201	0
	ACACTGG, MIR‐199A, MIR‐199B	72	0
TF target	GGAANCGGAANY_UNKNOWN	38	0
	YNGTTNNNATT_UNKNOWN	109	0
	V$HFH3_01	66	0.000435
	TTAYRTAA_V$E4BP4_01	74	0.000454
	SMTTTTGT_UNKNOWN	129	0.000471

### SNRPC promotes metastasis of HCC cells *in vitro*

**Fig. 4:**
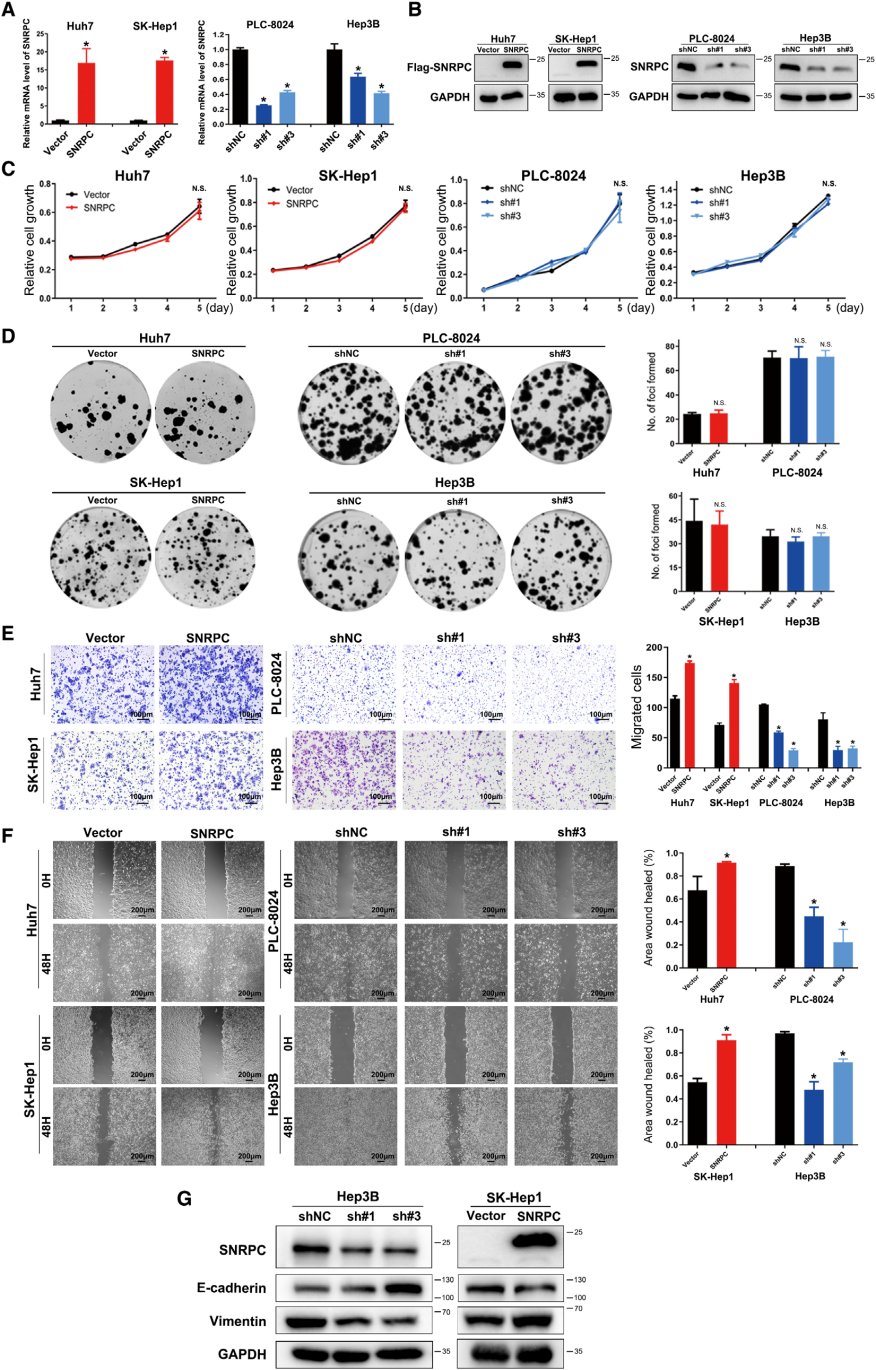
SNRPC promotes EMT and motility of HCC cells *in vitro*. The efficiency of SNRPC overexpression and knockdown was measured by (A) real‐time PCR and (B) western blotting assays. Cell growth of SNRPC overexpression and knockdown cells measured by (C) Cell Counting Kit‐8 assays and (D) colony formation assay is shown. Results were obtained from three independent assays. Cell motility of SNRPC overexpression and knockdown cells measured by (E) Transwell and (F) wound healing assay. Error bar presented as mean ± standard deviation (from triplicates), and significance is determined by the Student’s *t* test (*P < 0.05). Scale bars: 100 μm (E); 200 μm (F). (G) EMT markers in stably knocked down or overexpressed SNRPC cells examined by western blotting assays.

In evaluating the functions of SNRPC in HCC cells, we first constructed stable SNRPC overexpression and knockdown cells (Fig. 4A,B). To determine whether SNRPC regulated the growth of HCC cells, we performed cell proliferation and colony formation assays. No significant changes were observed in SNRPC overexpression and knockdown cells compared with the control groups (Fig. 4C,D).

The impact of SNRPC in cell motility was examined next. Transwell assays showed that the exogenous expression of SNRPC was enhanced, whereas the knockdown of SNRPC reduced the ability of cell migration in HCC cells (Fig. 4E). Wound healing assays further demonstrated that SNRPC‐overexpressed cells filled up the wound faster than cells in the control groups (Fig. 4F). These results indicated that SNRPC could promote the motility of HCC cells.

Epithelial–mesenchymal transition (EMT) plays crucial roles in tumor metastasis [17]. We used western blotting assays to examine the effect of SNRPC on the expression of EMT markers. Our results revealed that SNRPC knockdown in Hep3B cells up‐regulated the expression of epithelial marker E‐cadherin and down‐regulated the expression of mesenchymal marker Vimentin (Fig. 4G). In contrast, overexpressing SNRPC down‐regulated the expression of E‐cadherin and up‐regulated that of Vimentin. Together, our data suggested that SNRPC could promote EMT in HCC cells.

### SNRPC expression is correlated with tumor purity and immune infiltration level in HCC

**Fig. 5:**
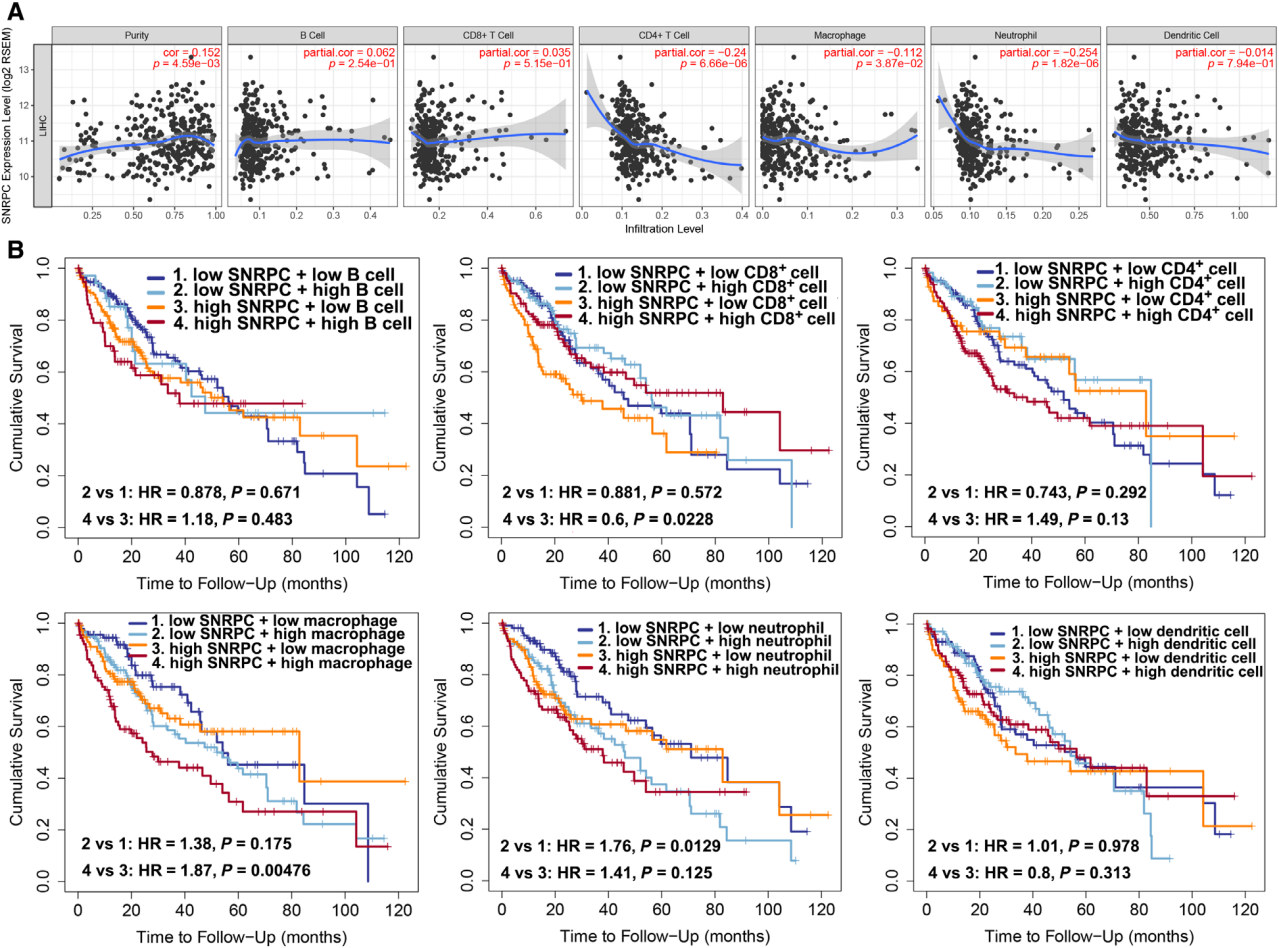
Correlations of *SNRPC* with immune infiltration levels in HCC. (A) Correlations of *SNRPC* expressions and immune infiltration levels in HCC. (B) The prognostic value of *SNRPC* expression and abundance of immune cell infiltration.

Much interest and active research efforts have been devoted recently to the communication between EMT and immune suppression [18, 19]. To broaden the understanding of *SNRPC* crosstalk with immune infiltrates, we explored the correlation between *SNRPC* expression and the abundance of six immune infiltrates (B cells, CD4^+^ T cells, CD8^+^ T cells, neutrophils, macrophages and dendritic cells) from the TIMER database. The results showed that *SNRPC* expression was negatively correlated with CD4^+^ T cells (*r* = −0.24, *P* = 6.66E−6), macrophage cells (*r* = −0.112, *P* = 3.87E−2) and neutrophil cells (*r* = −0.254, *P* = 1.82E−6; Fig. 5A). Furthermore, we analyzed the prognostic values of *SNRPC* expression and abundance of immune cell infiltration on patient survival in the TIMER portal (Fig. 5B). For patients with high *SNRPC* expression, more CD8^+^ T cell infiltration (*P* = 0.0228) or less macrophage infiltration (*P* = 0.0048) indicated better survival than less CD8^+^ T cell infiltration and more macrophage infiltration. Patients of low *SNRPC* expression with low neutrophil infiltration indicated a better prognosis (*P* = 0.0129) than patients with high neutrophil infiltration.

## Discussion

High‐throughput technology has proved a useful approach in screening for potential biomarkers in malignancies [20, 21]. In our study, bioinformatics analysis was performed to explore a newly discovered oncogene correlated with HCC. By overlapping three GEO databases, *SNRPC* was screened as a potential oncogene for HCC. SNRPC, a specific component of U1 snRNP, plays an important role in the sex bias of systemic autoimmune diseases and spinal muscular atrophy pathogenesis. However, the biological function of SNRPC in cancer is understudied.

Alternative splicing is assumed to be an important determinant for the diversity of transcriptional variants in HCC [22, 23]. U1 snRNP, which is required for the formation of spliceosome, is reported to suppress the migration and invasion of multiple types of tumor cells [24]. Early research found that snRNP polypeptide A, another important component of U1 snRNP, enhanced tumor cell growth in gastric cancer [25]. snRNP polypeptide G, another indispensable component in the biogenesis of spliceosome U snRNPs, also plays a critical role in the tumorigenesis and development in breast, lung and colon cancers [26]. As an essential element of spliceosome, the role of SNRPC in tumorigenesis and progression has rarely been studied. Therefore, we performed bioinformatics and functional analyses to gain a detailed insight into the potential function of SNRPC and its regulatory network in HCC.

In our study, of three independent public datasets (TCGA, HCCDB15 and HCCDB18), we found that the mRNA expression level of *SNRPC* was commonly up‐regulated in HCC, and increased *SNRPC* expression indicated poorer OS and DFS for patients with HCC. To validate it at the protein level, our TMA assay consistently showed that SNRPC expression was negatively affecting the prognosis of patients with HCC. Using cBioPortal, we identified that CNV of *SNRPC* was significantly higher in HCC than in normal liver tissues. It is noted that the top 10 genes, most of them negatively correlated with the outcome of HCC, were significantly correlated with SNRPC. We believe that SNRPC is a robust biomarker for HCC. In addition, both the PPI network and enrichment analysis showed that SNRPC is involved in the biogenesis, assembly and subunit organization of the ribonucleoprotein complex, RNA splicing and mRNA processing in HCC, which are consistent with the presumed physiological functions of SNRPC [27, 28].

Subsequently, our study identified several miRNAs that may potentially regulate *SNRPC*. miRs, by targeting key regulators involved in downstream signaling pathways, play an important role in oncogenesis and drug resistance [29, 30]. Earlier studies reported that overexpression of miR‐206, miR‐302c and miR‐200b inhibited proliferation, invasion and migration of HCC cells [31, 32, 33]. According to the enrichment, SNRPC was negatively regulated by these miRNAs. In agreement with the following result, SNRPC promotes the cell motility and EMT phenotype *in vitro*. Tumor cells with EMT signatures, as evidenced by reduction of the cell adhesion molecule E‐cadherin and overexpression of the mesenchymal molecule Vimentin, display an increased capability of metastasis. Our previous studies have also ascertained the key role of EMT in HCC metastasis [34, 35, 36].

Recent successes in clinical practice of cancer immunotherapy necessitate the study of the interaction between cancer cells and the host immune system [37, 38]. Depiction of the tumor‐infiltrating immune landscape is necessary for the understanding of tumor–immune interactions. Here we used TIMER to analyze and visualize the correlation between SNRPC expression and the immune cell infiltrates. The results showed that CD4^+^ T cells, macrophage cells and neutrophil cells were less infiltrated in tissues with high SNRPC expression. Patients with low SNRPC expression combined with more neutrophil cell infiltration indicated a poor prognosis. These results have potential implications for the selection of immunotherapy in patients with different SNRPC expression.

## Conclusions

We identified a novel functional oncogene, SNRPC, in HCC through multiple validations. SNRPC is up‐regulated in tumor tissues and can indicate worse prognosis in patients with HCC. Furthermore, SNRPC can enhance motility without affecting the proliferation of HCC cells. Data mining showed that SNRPC was potentially linked to the biogenesis, assembly and subunit organization of the ribonucleoprotein complex in HCC. Therefore, SNRPC may be a potential therapeutic target and marker for HCC.

## Author contributions

Yunfei Yuan and BL contributed to this article in the aspects of conceptualization and funding acquisition. Yichuan Yuan, YQ, LQ and WH collected and analyzed the data. YZ, JQ and DZ wrote the manuscript. All authors read and agreed to the published version of the manuscript.

## Conflict of interest

The authors declare no conflict of interest. The funders had no role in the design of the study; in the collection, analyses or interpretation of data; in the writing of the manuscript; or in the decision to publish the results.

## Supporting information


**Table S1**. 150 DEGs identified among 3 GEO databases.

**Table S2**. SNRPC coexpressed genes.

**Table S3**. KEGG pathway of SNRPC coexpressed genes.

**Table S4**. Kinase enrichment of SPRNC coexpressed genes.

**Table S5**. miRNA enrichment of SPRNC coexpressed genes.

**Table S6**. Transcription factor enrichment of SPRNC coexpressed genes.

## Data Availability

Gene expression DataSets are available in the GEO on the NCBI website (http://www.ncbi.nlm.nih.gov/geo; accession number GSE65372, GSE39791 and GSE36376).
