# Supplementary material for: SNRPC promotes hepatocellular carcinoma cell motility by inducing epithelial‐mesenchymal transition
Source: FEBS Open Bio. 2021 May 12;11(6):1757–70. doi: 10.1002/2211-5463.13175 (PMC8167856; doi:10.1002/2211-5463.13175)
Supplement: Supplementary file 1 — Table S1. 150 DEGs identified among 3 GEO databases. [file FEB4-11-1757-s003.pdf]

Supplementary Table 1. 150 DEGs identified among 3 GEO database.

gene	logFC	adj.P.Val	group value
SNRPC	4.574933	6.25E-97	up
YWHAZ	3.511826	1.92E-62	up
CPD	2.761155	2.67E-58	up
CAP2	2.569253	8E-114	up
LCAT	2.164215	8.7E-99	up
NCAPG	2.151238	5.11E-80	up
CCDC34	2.068671	9.21E-77	up
CAMK2B	2.007978	2.48E-42	up
AURKA	1.983505	1.37E-73	up
NOL7	1.938368	3.29E-56	up
CNIH4	1.933507	1.78E-67	up
MED20	1.855457	1.96E-92	up
SQLE	1.777251	9.44E-72	up
INMT	1.77531	4.82E-47	up
TP53I3	1.762078	7.05E-52	up
AURKB	1.73766	1.58E-61	up
HNRNPUL	1.693809	2.85E-74	up
SMYD3	1.679542	6.48E-51	up
PITX1	1.625843	1.24E-28	up
PLVAP	1.605489	3.19E-83	up
AKR1C3	1.546467	6.36E-83	up
UBE2C	1.535235	1.99E-92	up
COLEC11	1.53393	9.24E-22	up
PRSS8	1.526529	2.8E-08	up
RAP2A	1.514545	1.6E-56	up
CLEC1B	1.512202	5.2E-110	up
TOP2A	1.461528	3.25E-95	up
MCM4	1.436981	4.68E-59	up
H1F0	1.428966	2E-47	up
AKR1B10	1.366282	8.75E-39	up
MASP1	1.361788	6.75E-50	up
ACLY	1.345574	1.77E-86	up
PTTG1	1.339564	9E-106	up
PTH1R	1.336484	6.34E-78	up
HAMP	1.332288	1.42E-84	up
CCNB2	1.332113	2.43E-86	up
TUBB	1.252528	4.61E-63	up
PSMB4	1.250749	5.7E-90	up
ZFP36	1.248443	3.29E-19	up
CKAP2L	1.223952	3.12E-68	up
SNORA22	1.192105	2.42E-29	up
NAXE	1.189828	5.52E-85	up
PODXL	1.184661	2.64E-78	up
LOXL4	1.172358	8.79E-21	up

MYOM2	1.171715	1.13E-43	up
CDCA5	1.165991	1.46E-78	up
NPW	1.162049	2.83E-16	up
GBA	1.128271	1.04E-85	up
RACGAP1	1.108303	9.2E-70	up
KBTBD11	1.089561	4.06E-71	up
MID1IP1	1.087779	8.83E-65	up
SCRIB	1.081865	1.9E-48	up
AP3B1	1.07841	5.69E-87	up
TTC36	1.062688	8.6E-85	up
MRPL24	1.022922	5.6E-79	up
IGF2BP3	1.01544	4.7E-31	up
NOCT	1.005636	4.95E-43	up
ACACA	1.005588	3.12E-69	up
CTSA	1.003395	6.08E-67	up
CXCL12	1.00245	1.01E-55	up
ACSM3	0.99913	1.78E-60	up
XPR1	0.997282	7.34E-86	up
UCK2	0.974669	2.47E-68	up
NUSAP1	0.949334	2.41E-96	up
PSMD4	0.947235	1.3E-110	up
COPA	0.941485	4.38E-98	up
CDKN3	0.934959	7.79E-67	up
ACLY	0.91164	1.77E-86	up
S100A10	0.889657	6.97E-69	up
TBCE	0.888894	5.31E-73	up
PLOD3	0.881515	1.96E-88	up
SLC50A1	0.879415	7.97E-63	up
GLMP	0.866568	9.92E-80	up
ERBB3	0.85766	1.98E-58	up
COLEC11	0.84609	9.24E-22	up
HMGA1	0.842167	1.97E-52	up
HSPB1	0.806252	1.38E-94	up
NCSTN	0.797254	5.24E-76	up
MARCO	0.783121	7.38E-57	up
RRAGD	0.775129	3.86E-56	up
SLC39A1	0.772582	2.39E-91	up
SF3B4	0.771039	3.2E-110	up
CXCL12	0.765573	1.01E-55	up
NUP62	0.757396	2.1E-106	up
ID1	0.751046	2.79E-32	up
NEU1	0.729563	3.03E-88	up
UQCC2	0.721637	1.75E-90	up
SMG7	0.720902	4.68E-86	up
ANGPTL6	0.720576	3.12E-73	up
ETS2	0.715913	3.96E-17	up

LSM4	0.709653	1.12E-70	up
FAM13A	0.698537	3.54E-72	up
FCN3	0.692703	1.5E-110	up
DDX39A	0.691292	1.6E-120	up
SPINK1	0.682358	4.88E-49	up
CHST4	0.639911	7E-79	up
PSMD4	0.631005	1.3E-110	up
PNPLA7	0.610502	1.26E-47	up
CKS2	0.60899	1.53E-49	up
TACSTD2	0.594836	1.23E-15	up
TKT	0.587611	1.21E-63	up
AURKA	0.586151	1.37E-73	up
ANXA2	0.55389	9.52E-80	up
CSTB	0.551023	4.79E-49	up
RALY	0.550741	2.11E-69	up
FCN2	0.531051	3.5E-114	up
BSG	-0.69494	1.26E-51	down
FAM83H	-0.70917	7.65E-52	down
PRICKLE4	-0.7442	4.26E-73	down
IGFALS	-0.79952	1.69E-93	down
TUBG1	-0.86588	1.37E-84	down
SNX27	-0.95426	1.48E-85	down
FAM189B	-0.9838	3.69E-98	down
BGN	-1.04953	7.05E-08	down
DBH	-1.06068	1.4E-106	down
EGR1	-1.09117	1.2E-37	down
GOT2	-1.13106	2.65E-27	down
DNAJC9	-1.16818	2.56E-63	down
TRIM24	-1.21805	3.5E-63	down
FBXL18	-1.22517	2.33E-73	down
MGST1	-1.24268	1.01E-13	down
CCT3	-1.27097	1.37E-90	down
TPR	-1.30354	1.52E-82	down
ECM1	-1.30504	1.22E-98	down
FOSB	-1.34082	5.21E-42	down
RFWD2	-1.40704	3.79E-96	down
CTNNA1	-1.43659	6.4E-102	down
PRC1	-1.47347	1.08E-94	down
FAM20B	-1.48724	3.58E-85	down
LY6E	-1.55494	6.88E-55	down
SLC16A2	-1.57043	4.1E-23	down
GPC3	-1.57475	1.57E-82	down
TXNRD1	-1.59619	1.79E-44	down
EFTUD2	-1.86292	2.3E-101	down
CDC20	-1.95014	6.62E-94	down
COLEC10	-2.02262	5.37E-88	down

ASPM	-2.04551	5.81E-91	down
DPT	-2.05424	8.14E-35	down
CD1D	-2.06937	3.59E-23	down
PHPT1	-2.10255	7.74E-67	down
SQLE	-2.18494	9.44E-72	down
HMMR	-2.19695	8.59E-60	down
ATOH8	-2.30146	5.58E-57	down
SAE1	-2.51305	3.2E-123	down
FMO3	-2.53045	2.39E-24	down
CD34	-2.64031	2.24E-96	down
MFAP4	-2.65598	6.51E-25	down
PTK2	-2.82934	3.06E-90	down
ADGRG7	-2.87836	7.13E-78	down
RPL15	-3.70109	4.15E-43	down
