# Supplementary material for: SNRPC promotes hepatocellular carcinoma cell motility by inducing epithelial‐mesenchymal transition
Source: FEBS Open Bio. 2021 May 12;11(6):1757–70. doi: 10.1002/2211-5463.13175 (PMC8167856; doi:10.1002/2211-5463.13175)
Supplement: Supplementary file 2 — Table S2. SNRPC coexpressed genes. [file FEB4-11-1757-s006.pdf]

Supplementary Table2. SNRPC co-expressed genes.

Query	Correlation coefficient	P-value	FDR (BH)
SNRPC	1	1E-108	1E-104
TOMM6	0.863339324	1.1296E-108	1.1248E-104
CSNK2B	0.844399251	2.4217E-99	1.6076E-95
LSM2	0.80021318	1.00602E-81	5.0087E-78
MRPL14	0.796223443	2.36173E-80	9.40678E-77
MEA1	0.792983881	2.90956E-79	9.65731E-76
RPP21	0.787176885	2.34817E-77	6.68055E-74
NOL7	0.785940872	5.87365E-77	1.46217E-73
PFDN6	0.784776616	1.38535E-76	3.06547E-73
WDR46	0.775377838	1.16741E-73	2.32489E-70
RPS10	0.771796703	1.39589E-72	2.5272E-69
KLHDC3	0.758787165	7.93867E-69	1.31749E-65
RDBP	0.749740688	2.36088E-66	3.61669E-63
MRPS18A	0.747761473	7.94478E-66	1.13015E-62
CUTA	0.744706743	5.0569E-65	6.71388E-62
C6orf129	0.741206938	4.08142E-64	5.08009E-61
ZNRD1	0.740414576	6.51787E-64	7.63549E-61
C6orf125	0.735984767	8.65173E-63	9.57218E-60
NFKBIL1	0.726195282	2.18904E-60	2.29446E-57
C6orf153	0.725189223	3.81343E-60	3.79722E-57
FKBPL	0.719469891	8.54293E-59	8.10154E-56
EIF6	0.700904855	1.23606E-54	1.11891E-51
RPL38	0.695063608	2.1639E-53	1.87365E-50
LYRM4	0.691912646	9.85232E-53	8.17537E-50
SNRPD2	0.687115953	9.54036E-52	7.59985E-49
RPL27	0.685088024	2.45871E-51	1.88328E-48
MRPL2	0.681467355	1.30761E-50	9.64482E-48
RPS21	0.681152763	1.51023E-50	1.07415E-47
POLR2H	0.679866984	2.71606E-50	1.86519E-47
PSMG4	0.679595756	3.07286E-50	2.03987E-47
POLR1C	0.674834431	2.62534E-49	1.68657E-46
RPS18	0.673777716	4.20326E-49	2.61587E-46
RPLP2	0.672252615	8.26212E-49	4.98607E-46
C11orf48	0.668974561	3.48376E-48	2.04056E-45
BAT4	0.667774624	5.87249E-48	3.34145E-45
ROMO1	0.664210759	2.72995E-47	1.51019E-44
CDKN2AIPN	0.662945286	4.68714E-47	2.52282E-44
SNF8	0.662216553	6.39108E-47	3.34943E-44
SNRPB	0.661351536	9.22434E-47	4.71033E-44
PSMB3	0.661111214	1.02121E-46	5.01674E-44
RPL23A	0.661084495	1.03282E-46	5.01674E-44
RPL24	0.660792002	1.16879E-46	5.542E-44
ANAPC11	0.660065734	1.58796E-46	7.35448E-44
SNRPE	0.656706515	6.48182E-46	2.93376E-43

TRMT112	0.654312705	1.74681E-45	7.73062E-43
RPL35A	0.652308323	3.97882E-45	1.72257E-42
GNL1	0.651433657	5.68741E-45	2.40989E-42
TMEM14C	0.650002351	1.01792E-44	4.19054E-42
RPL27A	0.649970725	1.03107E-44	4.19054E-42
HIPK3	-0.649805996	1.10228E-44	4.39036E-42
MRPL11	0.649526903	1.23421E-44	4.81947E-42
TMEM101	0.649314805	1.34483E-44	5.15044E-42
RFXANK	0.648426147	1.92546E-44	7.23502E-42
REST	-0.648347773	1.98727E-44	7.32898E-42
RPL37	0.647665849	2.61503E-44	9.46879E-42
RPL23	0.647427168	2.87828E-44	1.02359E-41
RPS14	0.646837892	3.646E-44	1.27386E-41
C10orf118	-0.645242888	6.89636E-44	2.36795E-41
BYSL	0.644645927	8.7457E-44	2.95204E-41
FNIP2	-0.643735202	1.25532E-43	4.16662E-41
C6orf108	0.643428065	1.41765E-43	4.62828E-41
SECISBP2L	-0.6432814	1.50234E-43	4.82566E-41
TAF11	0.64270888	1.88365E-43	5.95443E-41
ARL15	-0.642666557	1.91538E-43	5.96011E-41
ELK3	-0.641251135	3.34331E-43	1.02434E-40
DYNLRB1	0.640158396	5.12954E-43	1.5478E-40
RPL19	0.639832761	5.82547E-43	1.73156E-40
IL6ST	-0.639584427	6.41834E-43	1.87972E-40
DTYMK	0.639243843	7.32972E-43	2.11553E-40
RPL32	0.638822775	8.63532E-43	2.45675E-40
FBL	0.637873676	1.24836E-42	3.50155E-40
HERC3	-0.637593297	1.3916E-42	3.79689E-40
NHP2	0.637592973	1.39178E-42	3.79689E-40
EMG1	0.637354998	1.52607E-42	4.10698E-40
RNF181	0.637214136	1.61152E-42	4.27913E-40
FLOT1	0.636853813	1.85229E-42	4.85372E-40
CCDC58	0.636697287	1.96767E-42	5.08911E-40
RPLP1	0.636318628	2.27708E-42	5.81385E-40
RPL37A	0.635475486	3.14984E-42	7.94038E-40
NSUN5	0.634336907	4.87393E-42	1.2133E-39
CCHCR1	0.633914523	5.72811E-42	1.40834E-39
RPL30	0.633397494	6.97765E-42	1.69463E-39
NME1	0.633241257	7.40583E-42	1.77695E-39
C9orf102	-0.63241979	1.01233E-41	2.40006E-39
RALGAPA2	-0.631651661	1.35483E-41	3.17429E-39
NDUFAF2	0.631606311	1.37831E-41	3.19175E-39
MYEOV2	0.631532493	1.41739E-41	3.24451E-39
PHB	0.631324201	1.53367E-41	3.4708E-39
DOCK9	-0.631035972	1.71031E-41	3.82706E-39
NDUFB11	0.630696045	1.94466E-41	4.30311E-39

ZC3H13	-0.630509205	2.08674E-41	4.56676E-39
EXOSC1	0.6304326	2.14793E-41	4.64956E-39
PFDN5	0.630350168	2.21575E-41	4.74479E-39
ATP5G2	0.630218995	2.32806E-41	4.93228E-39
RPS7	0.630164581	2.37629E-41	4.98146E-39
RBM42	0.629615242	2.92218E-41	6.06199E-39
AKAP2	-0.629057826	3.60287E-41	7.39703E-39
MLL3	-0.6270917	7.51536E-41	1.52723E-38
ARHGEF12	-0.626423795	9.63605E-41	1.93097E-38
SNRK	-0.626407086	9.69608E-41	1.93097E-38
COMMD4	0.626127865	1.07558E-40	2.12082E-38
LSM3	0.625903317	1.16907E-40	2.28254E-38
RPL14	0.625527048	1.34407E-40	2.59876E-38
RBM43	-0.624169802	2.21958E-40	4.25027E-38
PSMD13	0.623562429	2.77594E-40	5.24574E-38
RPS8	0.623546643	2.79211E-40	5.24574E-38
REV3L	-0.623289896	3.06852E-40	5.71117E-38
C5orf41	-0.623039657	3.36395E-40	6.18831E-38
TMEM14B	0.62302104	3.38702E-40	6.18831E-38
KIAA1109	-0.622383827	4.27861E-40	7.74623E-38
KIAA0247	-0.621417346	6.09231E-40	1.09305E-37
USP12	-0.620925953	7.28805E-40	1.29591E-37
MAP3K2	-0.619024855	1.45352E-39	2.56166E-37
PSMD4	0.618837093	1.55568E-39	2.71766E-37
PPTC7	-0.618675992	1.64898E-39	2.8556E-37
CHD9	-0.617800834	2.26136E-39	3.88232E-37
SCNM1	0.61748253	2.53598E-39	4.31659E-37
EHMT2	0.616698643	3.36118E-39	5.6727E-37
NFIC	-0.616437418	3.69135E-39	6.17759E-37
C8orf59	0.615860701	4.5383E-39	7.53168E-37
RPS20	0.614879902	6.44213E-39	1.06029E-36
C15orf63	0.614728814	6.79857E-39	1.10978E-36
EXOC6B	-0.613917181	9.07492E-39	1.46932E-36
ATP5E	0.61379963	9.4619E-39	1.51963E-36
WWC2	-0.613759102	9.59906E-39	1.52932E-36
RPS15A	0.613484632	1.05813E-38	1.67243E-36
TXNL4A	0.612962011	1.27345E-38	1.99692E-36
CCDC56	0.612859396	1.32057E-38	2.05462E-36
PSMG3	0.612514474	1.49194E-38	2.30326E-36
MRPL51	0.61168402	2.00019E-38	3.06413E-36
C11orf31	0.611653648	2.02171E-38	3.07347E-36
MEF2A	-0.611242921	2.33638E-38	3.52492E-36
SEC61G	0.610823715	2.7075E-38	4.05412E-36
RPS13	0.610702399	2.82539E-38	4.19908E-36
MRPL52	0.610510498	3.02234E-38	4.45851E-36
MAD2L1BP	0.610167638	3.40862E-38	4.99137E-36

TTBK2	-0.609413047	4.43933E-38	6.45324E-36
PRPF6	0.609314335	4.5952E-38	6.63141E-36
NUDT1	0.609104754	4.94432E-38	7.0839E-36
ZNF366	-0.609080473	4.98643E-38	7.0932E-36
PQBP1	0.609032713	5.07029E-38	7.16134E-36
CCDC72	0.608816018	5.46867E-38	7.66961E-36
EXOSC5	0.607883996	7.56618E-38	1.05371E-35
PIK3C2A	-0.607496033	8.65824E-38	1.19742E-35
NT5C	0.607061571	1.00671E-37	1.38267E-35
MACF1	-0.606863822	1.07814E-37	1.47062E-35
MXD3	0.606816759	1.09586E-37	1.48463E-35
UBA52	0.606753797	1.12002E-37	1.50711E-35
PLEKHG1	-0.606490519	1.22691E-37	1.63986E-35
CCDC12	0.606462684	1.23878E-37	1.64469E-35
RPS16	0.606386411	1.27191E-37	1.67748E-35
AARSD1	0.606169336	1.37105E-37	1.79635E-35
NOSIP	0.605243943	1.88686E-37	2.456E-35
RPL29	0.604850393	2.16064E-37	2.7941E-35
CHCHD1	0.604750692	2.23603E-37	2.87293E-35
SPATA13	-0.604651426	2.31367E-37	2.95363E-35
PCDHGB7	-0.604582858	2.36885E-37	3.00482E-35
RAPGEF6	-0.604524969	2.41645E-37	3.03763E-35
CCNT1	-0.604514419	2.42523E-37	3.03763E-35
GEMIN6	0.604301545	2.60921E-37	3.24765E-35
OTUD4	-0.603793257	3.10627E-37	3.82509E-35
ZBTB43	-0.603788301	3.11155E-37	3.82509E-35
RAPGEF2	-0.60358361	3.3376E-37	4.07781E-35
TSTD2	-0.603245005	3.74775E-37	4.55101E-35
TIMM17B	0.603117792	3.91441E-37	4.72458E-35
SNRPF	0.602667558	4.56536E-37	5.47705E-35
BRMS1	0.602501961	4.83081E-37	5.76081E-35
RUVBL2	0.602249031	5.26602E-37	6.24242E-35
KIAA1012	-0.602110761	5.52011E-37	6.50491E-35
C15orf40	0.601874936	5.98177E-37	7.00747E-35
CLTA	0.601831774	6.07031E-37	7.0696E-35
KLHL8	-0.601799362	6.13765E-37	7.10647E-35
SAC3D1	0.60173623	6.27094E-37	7.19536E-35
DHX33	-0.601728863	6.28668E-37	7.19536E-35
TGFBRAP1	-0.601614798	6.53543E-37	7.43732E-35
GEMIN7	0.601481277	6.83901E-37	7.73857E-35
SYNJ1	-0.601371937	7.09796E-37	7.98621E-35
DRG1	0.601203249	7.51663E-37	8.40976E-35
PA2G4	0.601085503	7.82326E-37	8.70392E-35
BOLA2	0.601003444	8.04424E-37	8.90005E-35
TOMM7	0.599907106	1.1662E-36	1.28314E-34
ETV3	-0.599512681	1.33244E-36	1.45655E-34

RPSA	0.59949939	1.33843E-36	1.45655E-34
BIRC6	-0.599268017	1.44713E-36	1.56628E-34
EIF3B	0.59922115	1.47019E-36	1.57817E-34
KIRREL	-0.599213539	1.47397E-36	1.57817E-34
GADD45GIP	0.599023205	1.57165E-36	1.67377E-34
MRPL23	0.598730088	1.73476E-36	1.83765E-34
BMPR2	-0.598559616	1.83722E-36	1.93588E-34
TBC1D7	0.598383629	1.94927E-36	2.04314E-34
ZBTB8OS	0.598356872	1.96688E-36	2.05081E-34
MFSD2B	0.598169166	2.09499E-36	2.173E-34
SAP30BP	0.598117132	2.13194E-36	2.19987E-34
PIH1D1	0.598086383	2.15407E-36	2.21126E-34
DDI2	-0.5980063	2.2128E-36	2.25205E-34
C7orf59	0.598001431	2.21643E-36	2.25205E-34
PSMB4	0.597904856	2.28948E-36	2.31446E-34
RPSAP58	0.597786264	2.38245E-36	2.39629E-34
MRPL27	0.597740255	2.41952E-36	2.42134E-34
LSM4	0.597393694	2.71768E-36	2.70613E-34
GNA11	-0.596978559	3.12302E-36	3.09427E-34
KIAA1370	-0.596813913	3.29986E-36	3.2533E-34
RPS24	0.596639456	3.49806E-36	3.43172E-34
SNRPD1	0.596567735	3.58292E-36	3.49773E-34
TDP2	0.596528304	3.63043E-36	3.52683E-34
KLHL28	-0.596507156	3.65618E-36	3.5346E-34
NDUFS6	0.596238622	3.99918E-36	3.84752E-34
RPS11	0.59620525	4.04397E-36	3.8719E-34
PRELID1	0.596109159	4.17573E-36	3.97893E-34
POP5	0.595921755	4.44505E-36	4.21538E-34
KIDINS220	-0.595827215	4.58736E-36	4.32973E-34
RPS23	0.5957748	4.66819E-36	4.38524E-34
MAGI1	-0.595753618	4.70126E-36	4.39557E-34
PFDN4	0.594973966	6.09372E-36	5.67086E-34
RNF160	-0.594954715	6.13282E-36	5.68071E-34
DNAJC7	0.594574662	6.95773E-36	6.41496E-34
RPS27A	0.593596857	9.61925E-36	8.82799E-34
C9orf41	-0.593347986	1.0444E-35	9.54097E-34
CLOCK	-0.593150107	1.11495E-35	1.01389E-33
SUPT4H1	0.592885768	1.21658E-35	1.10128E-33
SNRPA	0.592843832	1.23353E-35	1.11157E-33
ZNF791	-0.592711706	1.28846E-35	1.15584E-33
UHMK1	-0.592549126	1.35939E-35	1.214E-33
KRTCAP2	0.592447592	1.40563E-35	1.24969E-33
SNHG6	0.592277937	1.48638E-35	1.31561E-33
HEATR5A	-0.591918282	1.67308E-35	1.47431E-33
CLIC1	0.591572187	1.87457E-35	1.64459E-33
LOC388789	0.591510601	1.91286E-35	1.67082E-33

TUSC2	0.591460162	1.9448E-35	1.6913E-33
ABT1	0.591355553	2.01273E-35	1.74276E-33
MTX1	0.591201477	2.11707E-35	1.82517E-33
TIMM50	0.590799398	2.41525E-35	2.07326E-33
ERN1	-0.590625756	2.55653E-35	2.18512E-33
SBNO1	-0.590560608	2.61162E-35	2.22267E-33
RPL36	0.590123456	3.01291E-35	2.55328E-33
ABCF1	0.589966672	3.17122E-35	2.67605E-33
NEDD8	0.589952073	3.18637E-35	2.6775E-33
NBPF10	-0.589839118	3.30607E-35	2.7664E-33
SHFM1	0.589429764	3.77831E-35	3.14726E-33
PIKFYVE	-0.589417992	3.79283E-35	3.14726E-33
RPS5	0.589338931	3.89182E-35	3.216E-33
DOCK5	-0.589125084	4.17256E-35	3.43374E-33
C4orf29	-0.589020326	4.3173E-35	3.53823E-33
UBXN1	0.588972321	4.38528E-35	3.57922E-33
MYCBP2	-0.588704662	4.78418E-35	3.88885E-33
ITPA	0.58861063	4.93267E-35	3.99326E-33
CELF1	-0.588575726	4.98894E-35	4.02246E-33
USP53	-0.588355846	5.35832E-35	4.30286E-33
ZFPL1	0.588214781	5.60941E-35	4.4864E-33
LEPROT	-0.587635646	6.7681E-35	5.39147E-33
GTF2A1	-0.587518091	7.03073E-35	5.57836E-33
ASXL2	-0.587487713	7.10021E-35	5.61114E-33
C9orf129	-0.587121443	7.99349E-35	6.2921E-33
FRYL	-0.586947343	8.45622E-35	6.63014E-33
DPM2	0.586843273	8.74537E-35	6.82996E-33
ZNF281	-0.586464563	9.88259E-35	7.6726E-33
STK19	0.586458678	9.90137E-35	7.6726E-33
RPL8	0.586366424	1.02004E-34	7.8737E-33
RUNX1T1	-0.586043369	1.13198E-34	8.70399E-33
RGPD3	-0.585492173	1.35168E-34	1.03534E-32
MRPS23	0.585093946	1.53618E-34	1.17214E-32
KIAA1737	-0.585008154	1.57907E-34	1.20028E-32
RRP9	0.584958086	1.60465E-34	1.21508E-32
SOS2	-0.584924718	1.62193E-34	1.22351E-32
SHPRH	-0.584904017	1.63274E-34	1.22702E-32
SSH1	-0.584460863	1.882E-34	1.40903E-32
RPL18	0.584426619	1.90276E-34	1.41923E-32
ATP5G1	0.584251778	2.01234E-34	1.49536E-32
C6orf26	0.583982097	2.1937E-34	1.62407E-32
ZNF641	-0.583897971	2.25352E-34	1.66218E-32
PMF1	0.583742982	2.36798E-34	1.73478E-32
FAT4	-0.583741148	2.36936E-34	1.73478E-32
ENY2	0.58344324	2.60589E-34	1.90096E-32
PPIH	0.583361219	2.67502E-34	1.94427E-32

PPIA	0.58321361	2.80402E-34	2.03062E-32
BANF1	0.583164643	2.84815E-34	2.05511E-32
SASH1	-0.58305571	2.94883E-34	2.12007E-32
BUD31	0.583001398	3.00033E-34	2.14933E-32
WDR74	0.582963388	3.0369E-34	2.16773E-32
TBCEL	-0.582920558	3.07863E-34	2.18968E-32
HIP1	-0.582817597	3.1813E-34	2.25465E-32
REL	-0.581130933	5.43585E-34	3.83883E-32
ANKRD36BP	-0.58084395	5.95279E-34	4.18904E-32
SNRPD3	0.580610261	6.4094E-34	4.49448E-32
NOP10	0.580439731	6.76431E-34	4.72671E-32
ZMAT5	0.580388492	6.8747E-34	4.78705E-32
PTRH2	0.580205501	7.28367E-34	5.05416E-32
SYNE1	-0.579761403	8.37898E-34	5.79401E-32
NACA	0.579744408	8.42399E-34	5.80497E-32
C17orf95	0.579725667	8.4739E-34	5.81923E-32
C11orf83	0.579695659	8.55441E-34	5.8541E-32
C18orf21	0.579684898	8.58347E-34	5.8541E-32
RPL39	0.579637767	8.7119E-34	5.92142E-32
PPIL1	0.579053998	1.04692E-33	7.09163E-32
RPL35	0.578962776	1.07738E-33	7.27322E-32
NR2C2	-0.578556287	1.22412E-33	8.23591E-32
ZNF41	-0.577737966	1.58207E-33	1.06037E-31
CASC4	-0.577728645	1.58669E-33	1.06037E-31
TSSC4	0.577509694	1.69921E-33	1.13176E-31
POLR2I	0.577206237	1.8683E-33	1.24024E-31
FAM114A1	-0.577148821	1.90211E-33	1.25849E-31
PRKG1	-0.576782056	2.13287E-33	1.40649E-31
MRPS21	0.576464521	2.35486E-33	1.54776E-31
NDUFB9	0.576388361	2.41142E-33	1.57972E-31
CKS1B	0.576154077	2.59395E-33	1.69372E-31
RPL10A	0.576116929	2.62413E-33	1.70783E-31
MDN1	-0.575866367	2.8369E-33	1.84029E-31
RPL31	0.575795305	2.90029E-33	1.8753E-31
OBFC2B	0.575439612	3.23925E-33	2.08211E-31
AATF	0.575437824	3.24105E-33	2.08211E-31
RPS9	0.575376746	3.30311E-33	2.11516E-31
FAM128B	0.575049219	3.65649E-33	2.33394E-31
TAF10	0.574788544	3.96424E-33	2.5223E-31
ZFAND2B	0.574555175	4.26141E-33	2.70274E-31
CCNT2	-0.574493378	4.34372E-33	2.7462E-31
PSMC5	0.574332452	4.56553E-33	2.87729E-31
PCDHGA12	-0.574170375	4.80023E-33	3.01567E-31
RPS17	0.573793771	5.39267E-33	3.3772E-31
SNRPG	0.573743712	5.47668E-33	3.41059E-31
MYO9A	-0.573741609	5.48023E-33	3.41059E-31

RNF168	-0.573333409	6.21581E-33	3.85632E-31
PAFAH1B2	-0.573228536	6.42005E-33	3.96465E-31
ALG3	0.57322339	6.43024E-33	3.96465E-31
PSMA4	0.573064815	6.75223E-33	4.15033E-31
ELF1	-0.572902115	7.09918E-33	4.35016E-31
MAML2	-0.572822196	7.27599E-33	4.44483E-31
IMPDH2	0.572602056	7.78588E-33	4.74177E-31
HSPBP1	0.572564518	7.87629E-33	4.7822E-31
SCYL2	-0.572068357	9.17338E-33	5.55282E-31
ZNF581	0.571978762	9.42916E-33	5.69036E-31
ZKSCAN1	-0.571843013	9.8302E-33	5.91446E-31
MRPS24	0.571614409	1.0544E-32	6.32483E-31
CCNI	-0.571203919	1.19567E-32	7.15071E-31
FAM168A	-0.571164543	1.21017E-32	7.21574E-31
SYNE2	-0.571148114	1.21627E-32	7.23046E-31
EPAS1	-0.571066843	1.2469E-32	7.39046E-31
TIMM8B	0.571005044	1.27069E-32	7.50916E-31
DCUN1D1	-0.570876645	1.32158E-32	7.78678E-31
TSHZ1	-0.57059184	1.44176E-32	8.46983E-31
TLCD1	0.570532912	1.46795E-32	8.59828E-31
POLR2G	0.570512401	1.47717E-32	8.62693E-31
DOCK1	-0.570182767	1.63351E-32	9.5121E-31
CNPY2	0.570082728	1.68412E-32	9.77819E-31
CCT3	0.569938095	1.76003E-32	1.01552E-30
MIER1	-0.569934893	1.76175E-32	1.01552E-30
EIF2C4	-0.56993007	1.76434E-32	1.01552E-30
RPL28	0.569648283	1.92248E-32	1.10335E-30
EXOSC4	0.569238474	2.17777E-32	1.24627E-30
KIAA0754	-0.568947225	2.37931E-32	1.35771E-30
ROCK1	-0.568927038	2.39394E-32	1.36215E-30
COPS6	0.568883627	2.42571E-32	1.3763E-30
ATP5H	0.568108543	3.06851E-32	1.73606E-30
CHCHD2	0.568041738	3.13122E-32	1.76652E-30
VPS25	0.568011315	3.16019E-32	1.77783E-30
CPEB4	-0.567836107	3.33231E-32	1.86938E-30
LIN37	0.567818979	3.34963E-32	1.87382E-30
VPS13C	-0.567440397	3.75589E-32	2.0952E-30
RBL2	-0.567422655	3.77608E-32	2.10058E-30
MYST4	-0.567302724	3.9154E-32	2.17201E-30
MLL5	-0.566442836	5.07468E-32	2.80728E-30
EPC1	-0.566372367	5.18352E-32	2.85955E-30
PRPF31	0.566291955	5.31053E-32	2.92152E-30
EIF3F	0.566200317	5.45903E-32	2.99495E-30
MRPS16	0.566102316	5.62238E-32	3.07609E-30
ARL6IP4	0.566040086	5.72861E-32	3.12563E-30
43898	-0.565974888	5.84204E-32	3.1788E-30

KLHL20	-0.565840636	6.08263E-32	3.3007E-30
FAU	0.565574976	6.58796E-32	3.5652E-30
RNF5	0.565550098	6.63736E-32	3.5822E-30
COPZ1	0.565500683	6.73656E-32	3.62591E-30
UBL5	0.565439958	6.86048E-32	3.68265E-30
METTL1	0.565058036	7.69316E-32	4.11853E-30
UGCG	-0.565006827	7.81215E-32	4.17102E-30
MGAT5	-0.564903781	8.05713E-32	4.29031E-30
PPP1R12B	-0.564893229	8.08263E-32	4.29242E-30
LOC440957	0.564695849	8.57481E-32	4.54169E-30
KIAA2026	-0.564487388	9.1268E-32	4.82123E-30
RPL6	0.564201591	9.94107E-32	5.23747E-30
BAG4	-0.563915862	1.08269E-31	5.6891E-30
C17orf79	0.563902359	1.08706E-31	5.69705E-30
SH2B3	-0.563806877	1.11849E-31	5.83958E-30
SFRS2IP	-0.563801998	1.12012E-31	5.83958E-30
TFPT	0.563570795	1.20011E-31	6.24028E-30
ATP13A3	-0.563413391	1.25778E-31	6.52309E-30
PIGU	0.5633891	1.26692E-31	6.55343E-30
SURF2	0.563261379	1.31607E-31	6.79001E-30
RPLP0	0.563245825	1.32218E-31	6.79801E-30
REXO4	0.563240079	1.32444E-31	6.79801E-30
TCEB1	0.562979099	1.43147E-31	7.32848E-30
ADAM10	-0.56280956	1.50554E-31	7.6879E-30
WDFY3	-0.562471126	1.6649E-31	8.47992E-30
LOC650623	-0.562439394	1.68067E-31	8.53841E-30
UBR1	-0.562121958	1.84679E-31	9.35849E-30
C17orf89	0.561766212	2.0523E-31	1.03735E-29
FGD4	-0.561345561	2.32463E-31	1.17202E-29
HN1	0.561115321	2.48851E-31	1.25148E-29
STRN	-0.561080794	2.51405E-31	1.26114E-29
HERC1	-0.561053441	2.53447E-31	1.26819E-29
MRPL38	0.561000403	2.57452E-31	1.285E-29
FAM63B	-0.560965229	2.60143E-31	1.29519E-29
UXT	0.560897276	2.65421E-31	1.31817E-29
PRR3	0.560707909	2.80694E-31	1.39055E-29
RSPRY1	-0.560540017	2.9496E-31	1.4576E-29
RPL5	0.560423869	3.05246E-31	1.5047E-29
HCFC2	-0.560360071	3.11046E-31	1.5295E-29
NOP56	0.560018397	3.44013E-31	1.68744E-29
TPRKB	0.559998768	3.46008E-31	1.69306E-29
ERGIC3	0.559706088	3.77155E-31	1.84094E-29
ADAMTS15	-0.559551537	3.94704E-31	1.92189E-29
ARHGAP5	-0.559118564	4.48282E-31	2.17745E-29
TCEB2	0.559056356	4.56549E-31	2.21221E-29
EIF3K	0.559013959	4.62269E-31	2.23449E-29

DDX56	0.558977544	4.67239E-31	2.25304E-29
AKAP10	-0.558863854	4.83097E-31	2.32388E-29
EXOSC7	0.558380464	5.56675E-31	2.67137E-29
AP2S1	0.55821665	5.84042E-31	2.79596E-29
TNRC6B	-0.557848977	6.50399E-31	3.10616E-29
ZMYND11	-0.557520758	7.159E-31	3.4108E-29
MIIP	0.557457853	7.29178E-31	3.46577E-29
MAN1A2	-0.557374267	7.47199E-31	3.54297E-29
NME2	0.557317452	7.59699E-31	3.59368E-29
MRPL53	0.55718799	7.8896E-31	3.72325E-29
ACOT8	0.557119281	8.04939E-31	3.78968E-29
RP2	-0.556801931	8.82991E-31	4.14187E-29
SPG11	-0.556798386	8.83904E-31	4.14187E-29
ZNF800	-0.556769662	8.91335E-31	4.16689E-29
DNTTIP1	0.556746286	8.97428E-31	4.18554E-29
WDR70	0.556691349	9.1191E-31	4.24315E-29
DNAJB14	-0.555961534	1.12769E-30	5.23493E-29
JMJD1C	-0.555844568	1.16667E-30	5.40333E-29
RPS29	0.555671617	1.22678E-30	5.6685E-29
IMMP1L	0.555452289	1.30741E-30	6.02712E-29
LSM7	0.555405907	1.32513E-30	6.09467E-29
C14orf43	-0.555196378	1.40814E-30	6.46156E-29
RPL11	0.555077969	1.45731E-30	6.6718E-29
SERF2	0.554793078	1.58266E-30	7.21988E-29
ILKAP	0.554789556	1.58428E-30	7.21988E-29
HECTD1	-0.554605085	1.67116E-30	7.59842E-29
SNRPB2	0.554451214	1.74721E-30	7.92615E-29
UBXN4	-0.554351796	1.79817E-30	8.13874E-29
BOD1L	-0.55430518	1.82256E-30	8.23044E-29
ZDHHC20	-0.554222637	1.86656E-30	8.41006E-29
SEC24B	-0.554177394	1.89112E-30	8.50149E-29
TMEM223	0.554148806	1.9068E-30	8.54961E-29
YIPF3	0.554142264	1.91041E-30	8.54961E-29
AKAP11	-0.55407062	1.95035E-30	8.7088E-29
DMXL1	-0.554004175	1.98813E-30	8.85765E-29
NDUFA2	0.553956433	2.01573E-30	8.96054E-29
COX6B1	0.553933819	2.02893E-30	8.99914E-29
EIF3M	0.553707093	2.16611E-30	9.58623E-29
EIF2B4	0.55364524	2.2051E-30	9.73714E-29
BAT3	0.553495894	2.30212E-30	1.01431E-28
MRPL47	0.553435589	2.34248E-30	1.02981E-28
EEF1D	0.553393145	2.37131E-30	1.04019E-28
SS18L2	0.553367932	2.3886E-30	1.04547E-28
NAT9	0.553352909	2.39896E-30	1.0477E-28
KBTBD7	-0.553272369	2.45526E-30	1.06995E-28
ACER2	-0.552951451	2.69289E-30	1.17094E-28

PTTG1	0.552903041	2.73066E-30	1.18477E-28
RPS2	0.552756264	2.8484E-30	1.23317E-28
ETS1	-0.55272556	2.87365E-30	1.2414E-28
RPS19BP1	0.552498105	3.06777E-30	1.32239E-28
PDLIM5	-0.552171637	3.36923E-30	1.44921E-28
DRAP1	0.552009008	3.53015E-30	1.51515E-28
PPFIBP1	-0.551984434	3.55511E-30	1.52258E-28
BCL7C	0.551950101	3.59029E-30	1.53435E-28
RREB1	-0.551853413	3.6912E-30	1.5741E-28
CLASP1	-0.551746813	3.80572E-30	1.61946E-28
ALS2CR8	-0.551726478	3.82795E-30	1.62545E-28
C12orf47	0.551436027	4.15999E-30	1.76268E-28
DPP8	-0.551407795	4.19374E-30	1.77321E-28
WIBG	0.551307746	4.31555E-30	1.82085E-28
TAF1L	-0.551277761	4.35273E-30	1.83266E-28
RALY	0.551228638	4.41433E-30	1.85467E-28
ZNHIT3	0.551018667	4.68749E-30	1.96529E-28
ARL16	0.550752346	5.05809E-30	2.11622E-28
PCNX	-0.550578508	5.31547E-30	2.21924E-28
PTPN11	-0.55045749	5.50224E-30	2.29241E-28
ERAL1	0.550350335	5.67302E-30	2.35863E-28
SARNP	0.550264402	5.81376E-30	2.4121E-28
BTF3	0.550246661	5.84324E-30	2.4193E-28
PHF2	-0.550152735	6.0018E-30	2.47979E-28
MRPL9	0.54998359	6.29815E-30	2.59684E-28
MRTO4	0.54959373	7.03737E-30	2.89565E-28
HIPK1	-0.549525737	7.1748E-30	2.94611E-28
SSNA1	0.54945782	7.31473E-30	2.99738E-28
NME6	0.549357516	7.52632E-30	3.07776E-28
NBEAL1	-0.549223488	7.81852E-30	3.19069E-28
CHCHD8	0.549154885	7.9724E-30	3.24048E-28
C17orf90	0.549154591	7.97306E-30	3.24048E-28
PIK3R1	-0.54907932	8.14535E-30	3.2983E-28
UBE2H	-0.549077978	8.14846E-30	3.2983E-28
C12orf73	0.549029141	8.26225E-30	3.33758E-28
EP300	-0.549014949	8.29561E-30	3.33949E-28
PTOV1	0.549012865	8.30052E-30	3.33949E-28
RPS19	0.548658359	9.17893E-30	3.68545E-28
NBPF14	-0.548607397	9.31254E-30	3.72472E-28
USMG5	0.548606794	9.31413E-30	3.72472E-28
GPATCH3	0.548574601	9.39954E-30	3.75134E-28
TCF12	-0.548557162	9.44612E-30	3.76239E-28
AKAP13	-0.548391097	9.90133E-30	3.93583E-28
JAGN1	0.548279237	1.02201E-29	4.05445E-28
C11orf10	0.548166913	1.05504E-29	4.17716E-28
NBPF9	-0.548136628	1.06412E-29	4.20477E-28

FRMD4B	-0.547942778	1.12413E-29	4.43309E-28
NEU3	-0.547513765	1.26908E-29	4.99482E-28
VPS72	0.54743707	1.29688E-29	5.09414E-28
ATG2B	-0.547311087	1.34384E-29	5.26822E-28
PPAP2B	-0.546818845	1.544E-29	6.04103E-28
FNDC3A	-0.546744849	1.57654E-29	6.15623E-28
RPS3	0.546584375	1.64944E-29	6.4283E-28
C1orf35	0.546520893	1.67919E-29	6.53146E-28
C17orf37	0.546069833	1.9064E-29	7.40076E-28
OST4	0.545873079	2.01478E-29	7.8063E-28
PROX1	-0.545815165	2.04783E-29	7.91086E-28
ANKRD17	-0.545811891	2.04971E-29	7.91086E-28
LOC284441	-0.545579611	2.18785E-29	8.42767E-28
PDCD5	0.545540165	2.21221E-29	8.50504E-28
METTL5	0.545406518	2.29673E-29	8.81299E-28
KIAA0494	-0.544950206	2.6101E-29	9.99619E-28
FAM13C	-0.544711461	2.79053E-29	1.06667E-27
WASH2P	0.544568576	2.90434E-29	1.10805E-27
NHSL2	-0.54435425	3.08372E-29	1.17423E-27
PSENEN	0.544255776	3.16977E-29	1.20469E-27
NHLRC2	-0.544183677	3.23427E-29	1.22687E-27
ARFGEF2	-0.544122817	3.28972E-29	1.24553E-27
LONP2	-0.544115278	3.29665E-29	1.24579E-27
SF3B14	0.544003753	3.40094E-29	1.28276E-27
PCDHGB2	-0.54395965	3.44307E-29	1.2962E-27
LRCH1	-0.543838538	3.56144E-29	1.33823E-27
QARS	0.543747379	3.65318E-29	1.37011E-27
RPS15	0.543668181	3.73477E-29	1.39808E-27
NHP2L1	0.543626711	3.77822E-29	1.41169E-27
RPP30	0.543403988	4.0202E-29	1.4993E-27
SNRPA1	0.543331479	4.10224E-29	1.52703E-27
GAS5	0.543110337	4.3628E-29	1.62099E-27
PGP	0.542994121	4.50621E-29	1.67116E-27
DDR2	-0.542960963	4.54797E-29	1.68351E-27
RNF169	-0.542940585	4.57383E-29	1.68813E-27
GOLGA4	-0.542937773	4.57741E-29	1.68813E-27
NCOA2	-0.542779075	4.78397E-29	1.76105E-27
MRPL43	0.542218044	5.59078E-29	2.05425E-27
RBAK	-0.542171742	5.66308E-29	2.07699E-27
LMTK2	-0.541928955	6.05758E-29	2.21759E-27
TEAD1	-0.541855034	6.183E-29	2.25935E-27
TAOK1	-0.541847897	6.19524E-29	2.25968E-27
HRAS	0.541799991	6.27805E-29	2.28569E-27
GOLIM4	-0.54161516	6.60791E-29	2.4014E-27
C9orf119	0.54153869	6.74934E-29	2.44833E-27
EDF1	0.541511166	6.80097E-29	2.46257E-27

MRPS18B	0.541478202	6.86333E-29	2.48064E-27
POP7	0.541132071	7.55312E-29	2.72501E-27
MRPL17	0.540998106	7.8381E-29	2.82271E-27
MIA2	-0.540935707	7.97444E-29	2.86662E-27
SIRPB1	-0.540798571	8.28237E-29	2.97195E-27
C12orf10	0.540750354	8.3934E-29	3.00638E-27
DPY19L3	-0.540734185	8.43096E-29	3.01441E-27
GNB2L1	0.540713682	8.47883E-29	3.02609E-27
C12orf51	-0.540693853	8.52539E-29	3.03726E-27
ZNF462	-0.540499092	8.99625E-29	3.19929E-27
CKLF	0.540403406	9.2369E-29	3.27902E-27
MRPL48	0.54022617	9.69958E-29	3.43714E-27
PSMC1	0.540209249	9.74493E-29	3.44707E-27
BCL2L12	0.540112269	1.00089E-28	3.53418E-27
DPY30	0.539960601	1.0436E-28	3.67847E-27
NME2P1	0.539930454	1.0523E-28	3.70258E-27
PIK3CA	-0.539832071	1.0812E-28	3.79755E-27
LATS2	-0.539596328	1.15367E-28	4.04497E-27
TLR3	-0.53948096	1.19087E-28	4.16805E-27
PTPLB	-0.539368179	1.22838E-28	4.29178E-27
ATP5I	0.538775563	1.44548E-28	5.04145E-27
C20orf199	0.538620927	1.5081E-28	5.25068E-27
C11orf30	-0.538557388	1.53461E-28	5.33363E-27
MRPS17	0.537715346	1.93247E-28	6.70472E-27
XRCC6	0.537523236	2.03664E-28	7.05386E-27
RRP1	0.537262743	2.1868E-28	7.54818E-27
HEATR5B	-0.537262497	2.18695E-28	7.54818E-27
MED13L	-0.536973761	2.3662E-28	8.15274E-27
SLC30A4	-0.536899244	2.41477E-28	8.30572E-27
PUF60	0.536844976	2.45076E-28	8.41497E-27
CALD1	-0.536784256	2.49165E-28	8.54067E-27
C7orf47	0.536550047	2.65582E-28	9.08773E-27
POLK	-0.536538233	2.66437E-28	9.10138E-27
MANBAL	0.535986263	3.09609E-28	1.0558E-26
C19orf48	0.535871556	3.19412E-28	1.08737E-26
GBAP1	0.535757296	3.29482E-28	1.11973E-26
CCT7	0.535715023	3.33286E-28	1.13073E-26
FAM128A	0.53559776	3.44068E-28	1.16533E-26
ROCK2	-0.535540937	3.49416E-28	1.18143E-26
RIF1	-0.535420738	3.61E-28	1.21733E-26
EIF3G	0.535418132	3.61256E-28	1.21733E-26
LENG1	0.535137578	3.89812E-28	1.31133E-26
NXT1	0.534999608	4.04661E-28	1.35899E-26
MYSM1	-0.534959369	4.09097E-28	1.37158E-26
BIRC5	0.534953142	4.09787E-28	1.37158E-26
CTNND1	-0.534920895	4.13382E-28	1.38129E-26

MTMR6	-0.534887741	4.17111E-28	1.39142E-26
POLR2J	0.534841692	4.22344E-28	1.40652E-26
MKL2	-0.534585545	4.52664E-28	1.50498E-26
RPS12	0.534570385	4.54524E-28	1.50864E-26
WDR7	-0.534516934	4.61144E-28	1.52807E-26
RING1	0.534398316	4.76176E-28	1.57526E-26
NLE1	0.534380087	4.78529E-28	1.58041E-26
C4orf41	-0.5342986	4.89187E-28	1.61294E-26
TIMM10	0.534186186	5.04275E-28	1.65994E-26
MRPS7	0.534171281	5.0631E-28	1.66389E-26
BDP1	-0.534144615	5.09971E-28	1.67315E-26
UQCRB	0.534138529	5.1081E-28	1.67315E-26
UBE2M	0.534059574	5.2182E-28	1.70641E-26
POP4	0.533786532	5.61735E-28	1.83393E-26
TOMM40	0.533674447	5.7898E-28	1.88713E-26
MYLK	-0.533656356	5.81812E-28	1.89327E-26
RNF25	0.533548227	5.99026E-28	1.9461E-26
ARHGAP21	-0.533374527	6.27738E-28	2.03606E-26
TBC1D8B	-0.533248582	6.49402E-28	2.1029E-26
USP38	-0.532937265	7.06172E-28	2.28302E-26
LATS1	-0.532840738	7.2475E-28	2.33929E-26
MAPK8	-0.532780157	7.36655E-28	2.37387E-26
ARHGAP29	-0.532417997	8.11962E-28	2.61232E-26
NDUFA11	0.532329521	8.31486E-28	2.66749E-26
RP9P	0.532328161	8.3179E-28	2.66749E-26
OSBP	-0.532057075	8.94566E-28	2.86419E-26
ATP5J2	0.532048509	8.96624E-28	2.86617E-26
NCAPH2	0.532036536	8.99508E-28	2.87079E-26
KIAA1432	-0.532017809	9.04039E-28	2.88063E-26
FAM195B	0.531902614	9.32404E-28	2.96627E-26
RPL13AP20	0.531859519	9.4324E-28	2.99595E-26
PDRG1	0.531826617	9.51596E-28	3.01768E-26
TIMM9	0.531805506	9.56997E-28	3.02998E-26
ZFP91	-0.531647384	9.98419E-28	3.15611E-26
DAZAP1	0.531489259	1.04161E-27	3.28744E-26
PRDX5	0.531434791	1.05691E-27	3.33045E-26
CPSF4	0.531386523	1.07066E-27	3.36843E-26
TRPM7	-0.531266833	1.10551E-27	3.47259E-26
RPL18A	0.531237833	1.11412E-27	3.49413E-26
STRN3	-0.531181785	1.13095E-27	3.54134E-26
RC3H2	-0.53079487	1.25419E-27	3.92107E-26
METTL14	-0.530489975	1.36057E-27	4.24698E-26
C19orf56	0.530384611	1.39936E-27	4.36124E-26
HNRNPA1	0.53027311	1.44161E-27	4.48588E-26
RGPD4	-0.530201374	1.46945E-27	4.56538E-26
SLK	-0.530127036	1.49886E-27	4.6495E-26

SON	-0.529862452	1.60834E-27	4.98135E-26
PHPT1	0.529761136	1.65232E-27	5.10962E-26
NEU1	0.529541017	1.75201E-27	5.40949E-26
CD320	0.529184225	1.92635E-27	5.93858E-26
CDK5RAP1	0.529106609	1.96648E-27	6.05294E-26
CSGALNACT	-0.52905638	1.9929E-27	6.12478E-26
GTF2H4	0.529001825	2.02198E-27	6.20244E-26
NACA2	0.528997336	2.0244E-27	6.20244E-26
PEAR1	-0.528971064	2.03857E-27	6.23627E-26
C14orf2	0.528937547	2.05679E-27	6.27631E-26
CCDC137	0.528935408	2.05796E-27	6.27631E-26
XRN1	-0.528922755	2.06489E-27	6.2878E-26
RSF1	-0.528885288	2.08553E-27	6.34096E-26
LOC646471	-0.528294808	2.43907E-27	7.40458E-26
MFHAS1	-0.528144442	2.53817E-27	7.69371E-26
NENF	0.527839051	2.75185E-27	8.32874E-26
SBF2	-0.527679132	2.87073E-27	8.67536E-26
GTF2I	-0.527643216	2.89812E-27	8.74486E-26
IPMK	-0.527396959	3.09299E-27	9.31874E-26
DAD1	0.52734006	3.13982E-27	9.44555E-26
TTC37	-0.527224229	3.23734E-27	9.72422E-26
MRPL22	0.527085385	3.35818E-27	1.0072E-25
EIF2AK3	-0.52698424	3.449E-27	1.03288E-25
TRIM56	-0.526833561	3.58881E-27	1.07314E-25
TMF1	-0.526760013	3.65907E-27	1.09251E-25
UBE2S	0.526734189	3.68406E-27	1.09833E-25
SHARPIN	0.526625245	3.79136E-27	1.12862E-25
MAST4	-0.526571394	3.84554E-27	1.14304E-25
ERBB2IP	-0.526549852	3.86742E-27	1.14783E-25
MRPL55	0.52647369	3.94578E-27	1.16935E-25
RPL41	0.526197454	4.24337E-27	1.25484E-25
MRPL12	0.526194337	4.24685E-27	1.25484E-25
NUTF2	0.525996843	4.47328E-27	1.31978E-25
MORC3	-0.525829186	4.67482E-27	1.3772E-25
PATL1	-0.52581358	4.69403E-27	1.37898E-25
SART1	0.525813029	4.69471E-27	1.37898E-25
SDCCAG1	-0.525776473	4.74002E-27	1.39024E-25
RBX1	0.525657442	4.89057E-27	1.43027E-25
TMEM184C	-0.525657224	4.89085E-27	1.43027E-25
EIF2S2	0.525638899	4.91444E-27	1.43506E-25
POC1A	0.525614724	4.94574E-27	1.44133E-25
ZBTB1	-0.525611145	4.95039E-27	1.44133E-25
KLHDC10	-0.525598066	4.96742E-27	1.44323E-25
SSSCA1	0.525592849	4.97423E-27	1.44323E-25
C16orf42	0.525589472	4.97864E-27	1.44323E-25
RPL4	0.525433733	5.1864E-27	1.50127E-25

ENTPD4	-0.525208931	5.50146E-27	1.59015E-25
PDE10A	-0.524866183	6.01854E-27	1.73709E-25
MICAL2	-0.524690409	6.30204E-27	1.81628E-25
SSFA2	-0.524478604	6.66121E-27	1.91702E-25
ITGB3	-0.524433569	6.74015E-27	1.93694E-25
RP9	0.524345929	6.89642E-27	1.97677E-25
C19orf53	0.524344725	6.89859E-27	1.97677E-25
THOC4	0.524328563	6.92781E-27	1.98229E-25
GMPPA	0.524288476	7.0008E-27	2.0003E-25
ZNF236	-0.52414477	7.26876E-27	2.07389E-25
MTM1	-0.52400326	7.54252E-27	2.14892E-25
SMARCAD1	-0.523891143	7.76662E-27	2.2096E-25
KIAA2018	-0.52363765	8.29782E-27	2.35736E-25
SETX	-0.523577031	8.43007E-27	2.39152E-25
DYNLL1	0.523468954	8.67105E-27	2.45638E-25
ERC1	-0.523449449	8.71525E-27	2.4654E-25
TXLNG	-0.523394209	8.84166E-27	2.49761E-25
SLC41A2	-0.523273023	9.12536E-27	2.5741E-25
TSC22D2	-0.523133836	9.46231E-27	2.66537E-25
FBXO34	-0.523021713	9.74264E-27	2.74046E-25
RPL15	0.522905979	1.00406E-26	2.82029E-25
MAN2A1	-0.522701193	1.059E-26	2.97043E-25
MTCH1	0.52269074	1.06189E-26	2.97433E-25
SLC38A7	-0.522384183	1.14996E-26	3.2165E-25
ARL13B	-0.522200581	1.20613E-26	3.36886E-25
NPRL2	0.522079195	1.24474E-26	3.47184E-25
ARID1B	-0.522015614	1.26544E-26	3.52466E-25
PRKAR2A	-0.521942103	1.28981E-26	3.58751E-25
SNX13	-0.521918742	1.29765E-26	3.60428E-25
MICALCL	-0.521693224	1.37579E-26	3.81601E-25
MRPL28	0.521598632	1.40994E-26	3.90527E-25
MRPL24	0.521356858	1.50106E-26	4.15188E-25
PSMA6	0.521332129	1.5107E-26	4.17275E-25
ATRX	-0.52130838	1.52001E-26	4.19267E-25
BGLAP	0.521258013	1.53996E-26	4.2411E-25
ARPC4	0.521253325	1.54183E-26	4.2411E-25
NCRNA0015	0.521189236	1.56762E-26	4.30608E-25
AFF1	-0.521077974	1.6134E-26	4.42573E-25
TUBB	0.521047443	1.62619E-26	4.45468E-25
JTB	0.520998612	1.64686E-26	4.5051E-25
TRIM27	0.520967317	1.66024E-26	4.53548E-25
MRPS10	0.520547829	1.85032E-26	5.04781E-25
BLOC1S1	0.520159004	2.0456E-26	5.57294E-25
GPR176	-0.519999805	2.13131E-26	5.79229E-25
MON2	-0.519998672	2.13193E-26	5.79229E-25
TIMM13	0.519988211	2.13769E-26	5.80002E-25

THOC7	0.519962932	2.15166E-26	5.82999E-25
NACAP1	0.519797983	2.24508E-26	6.07483E-25
SCAMP3	0.51945088	2.45492E-26	6.63362E-25
PSMB7	0.519431513	2.46719E-26	6.65772E-25
DDAH2	0.519389261	2.49415E-26	6.72138E-25
NSMCE2	0.519225336	2.60154E-26	6.99786E-25
RPA3	0.519221993	2.60377E-26	6.99786E-25
LEMD2	0.519026632	2.73784E-26	7.34827E-25
SF3B5	0.5189989	2.75742E-26	7.39085E-25
KLHL11	-0.518749418	2.93986E-26	7.86925E-25
ZNF295	-0.518711874	2.96832E-26	7.93479E-25
ADAT1	-0.51853944	3.1026E-26	8.28261E-25
EIF4E2	0.518408988	3.20815E-26	8.55293E-25
SDF2	0.518325584	3.27749E-26	8.70638E-25
C14orf153	0.518324177	3.27867E-26	8.70638E-25
CDKL5	-0.518323986	3.27883E-26	8.70638E-25
HMGA1	0.518290388	3.30719E-26	8.76999E-25
GPS1	0.518154422	3.42444E-26	9.06885E-25
FCHO2	-0.518007755	3.55552E-26	9.40349E-25
RBM34	0.518001928	3.56083E-26	9.40504E-25
ATM	-0.517880046	3.67369E-26	9.69027E-25
URM1	0.517799026	3.75065E-26	9.8802E-25
ZNF192	-0.517675635	3.87094E-26	1.01836E-24
TRIO	-0.517611239	3.93522E-26	1.0339E-24
TAF1	-0.517552922	3.99434E-26	1.04805E-24
JAK1	-0.517509758	4.03866E-26	1.05829E-24
ATXN7	-0.517412322	4.1405E-26	1.08355E-24
RPL7A	0.517218012	4.35123E-26	1.1372E-24
ZNF609	-0.517181388	4.39212E-26	1.14638E-24
ATRIP	0.517114875	4.46734E-26	1.16449E-24
TMEM126A	0.517050112	4.54181E-26	1.18236E-24
LDLRAD3	-0.517005235	4.59413E-26	1.19441E-24
CYHR1	0.516854511	4.77425E-26	1.23962E-24
USO1	-0.516844798	4.78609E-26	1.24107E-24
NANS	0.516839739	4.79227E-26	1.24107E-24
LOC653653	-0.516800457	4.84053E-26	1.25194E-24
LOC150786	-0.516466227	5.27099E-26	1.3615E-24
COX6C	0.516381063	5.38658E-26	1.38956E-24
PRKCE	-0.516328114	5.45971E-26	1.4066E-24
NUDC	0.516191463	5.65298E-26	1.45451E-24
LOC100288	0.516115139	5.76386E-26	1.48113E-24
ATP5EP2	0.515966775	5.98557E-26	1.53612E-24
SGK269	-0.515779472	6.27751E-26	1.60897E-24
SMUG1	0.515767214	6.2971E-26	1.61191E-24
TEK	-0.51569181	6.41894E-26	1.64099E-24
VPS28	0.515676333	6.44423E-26	1.64534E-24

PDE3A	-0.515359988	6.98331E-26	1.7807E-24
CDH5	-0.515310487	7.0716E-26	1.80091E-24
AHCYL2	-0.515284468	7.11845E-26	1.81052E-24
ATIC	0.515279268	7.12785E-26	1.8106E-24
ZNF622	0.515255704	7.1706E-26	1.81914E-24
SPATA24	0.515211102	7.2522E-26	1.8375E-24
TMEM160	0.515149862	7.36575E-26	1.8639E-24
RPL13	0.515052217	7.55043E-26	1.90821E-24
KIAA0564	-0.514812856	8.02269E-26	2.02499E-24
ANKRD19	0.514505952	8.67109E-26	2.18588E-24
FTSJD1	-0.514358307	9.00118E-26	2.26623E-24
ICT1	0.513986645	9.88802E-26	2.48636E-24
MPZL3	-0.513949305	9.98174E-26	2.50676E-24
KIAA1632	-0.513839775	1.02618E-25	2.57384E-24
PDZD8	-0.513788205	1.03963E-25	2.6043E-24
EEF1G	0.51373055	1.05487E-25	2.63917E-24
C20orf27	0.513647792	1.07714E-25	2.6915E-24
MUDENG	-0.513601487	1.0898E-25	2.71973E-24
N4BP2	-0.513580822	1.0955E-25	2.73053E-24
LOC100271	0.513492495	1.12019E-25	2.78857E-24
ATF2	-0.513422869	1.14003E-25	2.83443E-24
TECTA	-0.513305507	1.17428E-25	2.91593E-24
LSM5	0.513181575	1.21154E-25	3.0047E-24
RSC1A1	-0.513148107	1.2218E-25	3.02638E-24
DDX49	0.513018086	1.26248E-25	3.12327E-24
PTAR1	-0.512993345	1.27037E-25	3.13889E-24
MRPS15	0.512958536	1.28156E-25	3.16261E-24
EIF2B5	0.512782233	1.33972E-25	3.30204E-24
SLIT2	-0.512761615	1.34669E-25	3.31512E-24
C11orf51	0.512723294	1.35974E-25	3.34311E-24
MYL6	0.512664402	1.38004E-25	3.38883E-24
KIAA1033	-0.512525017	1.42928E-25	3.50542E-24
SNRNP35	0.512405596	1.47284E-25	3.60782E-24
LOC100132	-0.512371394	1.48555E-25	3.6345E-24
LOC442454	0.512342888	1.49623E-25	3.65614E-24
SDHAF2	0.512090021	1.59436E-25	3.89114E-24
WDR11	-0.512066796	1.60369E-25	3.90911E-24
HEG1	-0.511848296	1.69409E-25	4.12443E-24
PRDM2	-0.511823575	1.70463E-25	4.14502E-24
SMG1	-0.511801783	1.71397E-25	4.16266E-24
PALM2-AKA	-0.511750849	1.73601E-25	4.21105E-24
DMAP1	0.511224247	1.98091E-25	4.79925E-24
RNF111	-0.510998307	2.09616E-25	5.0723E-24
TMEM183A	0.510911721	2.14206E-25	5.17708E-24
C7orf58	-0.510894511	2.1513E-25	5.19311E-24
PSMC3	0.510709561	2.25312E-25	5.43231E-24

CLCN3	-0.510581766	2.32624E-25	5.60182E-24
LIMS1	-0.510567872	2.33433E-25	5.61451E-24
BAZ2B	-0.510532263	2.35519E-25	5.65785E-24
NSMCE1	0.510467833	2.3934E-25	5.74271E-24
MRPL33	0.510399916	2.43434E-25	5.83392E-24
TRUB1	-0.510323997	2.48092E-25	5.93841E-24
SETBP1	-0.510053552	2.65412E-25	6.34536E-24
THOC6	0.50997376	2.70746E-25	6.46512E-24
C14orf156	0.509820326	2.81303E-25	6.70916E-24
TRIP11	-0.509753315	2.8604E-25	6.81397E-24
HM13	0.50955135	3.00798E-25	7.15697E-24
SETD7	-0.509494222	3.05107E-25	7.25083E-24
PPP2R5E	-0.509467534	3.0714E-25	7.29047E-24
INO80C	0.509324209	3.18294E-25	7.54622E-24
ZNF423	-0.509238082	3.25188E-25	7.70049E-24
TAF13	-0.50921972	3.26676E-25	7.72655E-24
PSD3	-0.509205451	3.27838E-25	7.74483E-24
TMEM199	0.509188013	3.29263E-25	7.76927E-24
EMP1	-0.509150431	3.32354E-25	7.83248E-24
PTDSS2	0.509145916	3.32728E-25	7.83248E-24
CDKN2AIP	-0.509138907	3.33308E-25	7.83688E-24
LIG4	-0.509126912	3.34304E-25	7.85102E-24
ZNF689	-0.509049455	3.40804E-25	7.99425E-24
RASA2	-0.509030263	3.42434E-25	8.02303E-24
ADSL	0.508769168	3.65386E-25	8.55071E-24
MYO18A	-0.508706629	3.71105E-25	8.67435E-24
COMMD9	0.508701673	3.71562E-25	8.67485E-24
EDIL3	-0.508485947	3.92E-25	9.14131E-24
COX7C	0.508162149	4.24776E-25	9.89406E-24
RPL22	0.508133762	4.27776E-25	9.95228E-24
PES1	0.508044793	4.37312E-25	1.01623E-23
TET2	-0.507853586	4.58521E-25	1.06427E-23
GPR17	-0.507626071	4.85084E-25	1.12461E-23
FAM179B	-0.50751911	4.9809E-25	1.15343E-23
PSMA7	0.507456979	5.05803E-25	1.16993E-23
LMBRD2	-0.50743842	5.0813E-25	1.17237E-23
RPL13A	0.507435232	5.08531E-25	1.17237E-23
C3orf10	0.507434461	5.08627E-25	1.17237E-23
NDUFB7	0.507395456	5.13557E-25	1.18237E-23
ORMDL2	0.506975872	5.69652E-25	1.31E-23
EEF1B2	0.506472811	6.44921E-25	1.48139E-23
ITSN2	-0.506399654	6.56655E-25	1.5066E-23
NFKB1	-0.506371016	6.61305E-25	1.51552E-23
KLF10	-0.50635741	6.63526E-25	1.51886E-23
OSBPL11	-0.506326912	6.68531E-25	1.52856E-23
TOR1AIP2	-0.506168502	6.95131E-25	1.58756E-23

APC	-0.505793163	7.62397E-25	1.73919E-23
USE1	0.505748607	7.70796E-25	1.75634E-23
ZNF75D	-0.505639567	7.9174E-25	1.802E-23
SSBP1	0.505603701	7.9875E-25	1.81588E-23
POLR2L	0.505551401	8.09083E-25	1.83727E-23
HSPG2	-0.50544596	8.30318E-25	1.88335E-23
MCRS1	0.505416593	8.36329E-25	1.89482E-23
SGMS2	-0.505402672	8.39194E-25	1.89915E-23
PRPF19	0.505294922	8.61696E-25	1.94786E-23
SMAD1	-0.505260771	8.68952E-25	1.96204E-23
FAM122A	-0.505162989	8.90061E-25	2.00742E-23
ZNF441	-0.505102544	9.03362E-25	2.03512E-23
ANO6	-0.504759399	9.82673E-25	2.21129E-23
ECE2	0.504678055	1.00246E-24	2.25327E-23
KBTBD4	-0.504584489	1.0257E-24	2.30292E-23
PRMT10	-0.504534092	1.03844E-24	2.3289E-23
CYLD	-0.504504125	1.04609E-24	2.34341E-23
HIVEP2	-0.50430687	1.09785E-24	2.45659E-23
MRPS34	0.504050902	1.16879E-24	2.6124E-23
RHBDD1	-0.504045626	1.1703E-24	2.61284E-23
WASH3P	0.503899343	1.21291E-24	2.70494E-23
C14orf4	-0.50385138	1.22721E-24	2.73377E-23
NFATC2	-0.503816642	1.23767E-24	2.754E-23
PDCD2L	0.503773081	1.25092E-24	2.78036E-23
RPUSD3	0.503601986	1.3043E-24	2.89577E-23
UBR3	-0.503484871	1.34213E-24	2.97644E-23
NR2C2AP	0.503458261	1.35087E-24	2.9925E-23
LRBA	-0.503284698	1.4093E-24	3.11848E-23
PARL	0.503238932	1.42512E-24	3.14998E-23
SLC12A6	-0.503215666	1.43323E-24	3.16439E-23
PPP4R2	-0.503085034	1.47961E-24	3.26318E-23
PCDHGA5	-0.503015946	1.50474E-24	3.31492E-23
SUCNR1	-0.502966673	1.52291E-24	3.35125E-23
TIMM16	0.50295206	1.52835E-24	3.35949E-23
HSPE1	0.502633889	1.65147E-24	3.62614E-23
PPP1R7	0.502606026	1.66271E-24	3.64679E-23
PAQR3	-0.502458028	1.72367E-24	3.77634E-23
NAA10	0.502391782	1.75167E-24	3.83347E-23
GFER	0.502278171	1.80074E-24	3.93652E-23
RAB2B	-0.502270379	1.80415E-24	3.93966E-23
RASGEF1B	-0.502183332	1.84273E-24	4.01949E-23
MED14	-0.502137001	1.86359E-24	4.06056E-23
RBBP5	-0.501998378	1.92742E-24	4.19504E-23
C6orf136	0.501935344	1.95716E-24	4.2551E-23
POLR2F	0.501905253	1.97151E-24	4.28164E-23
CDK8	-0.501762826	2.04086E-24	4.42743E-23

PTPRB	-0.501746385	2.04902E-24	4.44029E-23
C17orf49	0.501646739	2.09917E-24	4.54402E-23
PDPR	-0.501456767	2.19815E-24	4.75311E-23
CDK17	-0.501350716	2.25539E-24	4.87159E-23
DNAJC27	-0.501343706	2.25922E-24	4.87459E-23
UBL7	0.501126417	2.38134E-24	5.13252E-23
KIAA0355	-0.500869196	2.53434E-24	5.45637E-23
IPP	-0.500673797	2.65701E-24	5.71429E-23
HBXIP	0.500663419	2.66369E-24	5.72247E-23
FTH1	0.500636932	2.6808E-24	5.75304E-23
ADAM17	-0.500484446	2.78146E-24	5.96263E-23
PSMB6	0.500312029	2.89978E-24	6.20843E-23
JMJD4	0.500308347	2.90236E-24	6.20843E-23
C22orf32	0.500193155	2.98423E-24	6.37671E-23
ATXN1L	-0.500176449	2.99629E-24	6.39562E-23
TNRC6A	-0.500101266	3.05117E-24	6.50578E-23
RAD54L2	-0.499990922	3.13351E-24	6.67421E-23
PRRG1	-0.499960301	3.15675E-24	6.71652E-23
CTU1	0.499814528	3.26971E-24	6.94945E-23
STRA13	0.49979032	3.28886E-24	6.98268E-23
MRPS5	0.499696145	3.36438E-24	7.13542E-23
CENPC1	-0.499637217	3.41251E-24	7.22979E-23
FAM73A	-0.499479544	3.54464E-24	7.50176E-23
MYL6B	0.499435284	3.58263E-24	7.57411E-23
CHMP2A	0.49934403	3.66223E-24	7.73418E-23
SAMD8	-0.499291354	3.70897E-24	7.82459E-23
SLC25A40	-0.49924624	3.74946E-24	7.90165E-23
C20orf20	0.499136156	3.85012E-24	8.1052E-23
MLL2	-0.499105247	3.87886E-24	8.15707E-23
OSBPL8	-0.499080278	3.90223E-24	8.19756E-23
CCPG1	-0.498853854	4.12059E-24	8.64716E-23
TMEM141	0.498660925	4.31613E-24	9.04797E-23
C1orf182	0.49860727	4.37212E-24	9.1557E-23
SRRD	0.498550663	4.43196E-24	9.27127E-23
STAG1	-0.498392591	4.60339E-24	9.61977E-23
HSPB1	0.498246349	4.7678E-24	9.9529E-23
EXOC5	-0.498219426	4.79869E-24	1.00069E-22
RPS25	0.498095279	4.94372E-24	1.02985E-22
RRP8	0.498007801	5.0485E-24	1.05015E-22
CISD3	0.49800515	5.05171E-24	1.05015E-22
TNKS2	-0.497974013	5.08955E-24	1.05692E-22
SPSB2	0.49785319	5.23907E-24	1.08683E-22
CDC14C	-0.497776395	5.33634E-24	1.10586E-22
CPEB2	-0.497766848	5.34856E-24	1.10724E-22
RPL34	0.497713091	5.41787E-24	1.12035E-22
CNOT6L	-0.497709017	5.42316E-24	1.12035E-22

TMEM115	0.497520235	5.67388E-24	1.17094E-22
ZNF776	-0.497486915	5.71931E-24	1.17909E-22
RASSF3	-0.497373232	5.877E-24	1.21035E-22
ANKIB1	-0.497362379	5.89228E-24	1.21224E-22
GPR116	-0.496961866	6.48431E-24	1.33266E-22
C16orf13	0.496829209	6.69304E-24	1.37414E-22
MAGOH	0.496789435	6.75691E-24	1.38583E-22
MRPL13	0.496604332	7.06213E-24	1.44694E-22
CHD6	-0.496596706	7.07499E-24	1.44808E-22
COL27A1	-0.496585996	7.09309E-24	1.4503E-22
NAA38	0.496417582	7.38384E-24	1.5082E-22
ANKRD50	-0.496318766	7.55987E-24	1.54257E-22
CASS4	-0.496228786	7.72375E-24	1.5744E-22
RPS6	0.496138584	7.89156E-24	1.60696E-22
MAF1	0.49608777	7.98767E-24	1.62487E-22
C19orf62	0.495766424	8.62276E-24	1.75227E-22
JHDM1D	-0.495715945	8.72695E-24	1.77163E-22
KLHL24	-0.495617602	8.93351E-24	1.81172E-22
CNPY3	0.495414496	9.37549E-24	1.89942E-22
DAXX	0.495373967	9.46624E-24	1.91585E-22
TOP1	-0.495264312	9.71612E-24	1.96443E-22
SOCS4	-0.495191305	9.88608E-24	1.99587E-22
LCMT1	0.495188939	9.89164E-24	1.99587E-22
SMARCA2	-0.495062588	1.01929E-23	2.05457E-22
PLSCR4	-0.494934262	1.05081E-23	2.11597E-22
ATE1	-0.494838242	1.07503E-23	2.16255E-22
PCDHGA6	-0.494793919	1.08639E-23	2.1832E-22
FBXL3	-0.494658477	1.12185E-23	2.25219E-22
ZNF444	0.494588616	1.14059E-23	2.28592E-22
RANBP1	0.494587268	1.14095E-23	2.28592E-22
C14orf118	-0.494216617	1.24566E-23	2.4932E-22
MTMR9	-0.494198054	1.25114E-23	2.50166E-22
INO80E	0.493992058	1.31365E-23	2.6226E-22
SPTBN1	-0.493990073	1.31427E-23	2.6226E-22
NOC4L	0.493938946	1.33026E-23	2.65186E-22
PPAN	0.493883311	1.34788E-23	2.68429E-22
LYST	-0.493857144	1.35624E-23	2.69826E-22
HINT1	0.493768389	1.385E-23	2.75273E-22
LOC152217	0.493678993	1.41458E-23	2.80704E-22
FUBP3	-0.493677293	1.41515E-23	2.80704E-22
NMB	0.493608845	1.43822E-23	2.84997E-22
DOM3Z	0.493589636	1.44476E-23	2.86009E-22
C1orf31	0.493461024	1.48933E-23	2.94538E-22
DYRK1A	-0.493287917	1.55146E-23	3.0652E-22
PIK3R4	-0.493019969	1.6527E-23	3.26199E-22
SMEK2	-0.492823753	1.73094E-23	3.41303E-22

CDC42BPB	-0.492643457	1.80605E-23	3.55761E-22
WASH7P	0.492613356	1.8189E-23	3.57721E-22
NSFL1C	0.492611738	1.81959E-23	3.57721E-22
CHD2	-0.492549149	1.8466E-23	3.62674E-22
PRR13	0.492477267	1.87812E-23	3.685E-22
PPP4C	0.492470722	1.88102E-23	3.68705E-22
CENPM	0.492319839	1.94901E-23	3.81656E-22
NDUFB4	0.492202485	2.00356E-23	3.91953E-22
ZNF25	-0.492186644	2.01104E-23	3.9303E-22
NUMB	-0.492073724	2.06514E-23	4.03209E-22
LCOR	-0.49201884	2.09196E-23	4.08045E-22
RANBP6	-0.491888836	2.15686E-23	4.20291E-22
ESYT2	-0.491809077	2.19765E-23	4.27821E-22
HERC2	-0.491722528	2.24277E-23	4.3618E-22
SFMBT2	-0.491516687	2.3538E-23	4.57326E-22
ADRM1	0.491416017	2.41005E-23	4.67644E-22
PLEKHM3	-0.491413281	2.4116E-23	4.67644E-22
SNED1	-0.491252708	2.50414E-23	4.85117E-22
FYCO1	-0.491129962	2.57724E-23	4.98792E-22
RASSF8	-0.490989432	2.66351E-23	5.14989E-22
FAM86A	0.49097157	2.67468E-23	5.16646E-22
YTHDC2	-0.490922552	2.70556E-23	5.22106E-22
SAV1	-0.490882981	2.73075E-23	5.26456E-22
NEK1	-0.490799804	2.78445E-23	5.3629E-22
C7orf30	0.490674961	2.86701E-23	5.51658E-22
EGFR	-0.490352783	3.0914E-23	5.94258E-22
PDPK1	-0.490308529	3.12354E-23	5.99858E-22
HIVEP1	-0.490222582	3.18691E-23	6.11438E-22
RPL12	0.49017185	3.22491E-23	6.18133E-22
RRN3	-0.490164411	3.23052E-23	6.18613E-22
RPL17	0.490133653	3.25381E-23	6.22475E-22
PSMB1	0.490086832	3.28959E-23	6.28716E-22
PHF1	0.489899491	3.43667E-23	6.56197E-22
TNPO3	-0.489843654	3.48175E-23	6.64168E-22
DIABLO	0.489755388	3.5542E-23	6.77339E-22
USP34	-0.489639517	3.65157E-23	6.95231E-22
PFDN2	0.489393005	3.86756E-23	7.35649E-22
ATAD2B	-0.489307306	3.94556E-23	7.4977E-22
CCDC61	0.489211814	4.0343E-23	7.65902E-22
FDX1L	0.489098162	4.14249E-23	7.85691E-22
DDX3X	-0.488946745	4.29107E-23	8.13099E-22
ARPC3	0.488939717	4.29809E-23	8.13655E-22
ACAP2	-0.488868562	4.36984E-23	8.26451E-22
RAB24	0.48863306	4.61581E-23	8.72143E-22
LOC100144	0.488576297	4.67711E-23	8.82887E-22
MOB2	0.488427961	4.84111E-23	9.1298E-22

HDDC3	0.488319913	4.96413E-23	9.34733E-22
B3GNT2	-0.488318427	4.96584E-23	9.34733E-22
ERI3	0.4882295	5.06943E-23	9.53331E-22
SPTLC2	-0.488155002	5.15785E-23	9.69043E-22
XPO5	0.488147041	5.16739E-23	9.6992E-22
YY2	-0.488092962	5.23264E-23	9.81244E-22
FAM161B	-0.48793656	5.42597E-23	1.01654E-21
ARHGAP42	-0.487922572	5.4436E-23	1.01888E-21
AKAP9	-0.487837785	5.55168E-23	1.03814E-21
NDUFS5	0.487770489	5.63896E-23	1.05347E-21
JMJD6	0.487740556	5.67822E-23	1.05981E-21
FBXO3	-0.487714849	5.71215E-23	1.06514E-21
PEX26	-0.487517695	5.97909E-23	1.11388E-21
KDR	-0.487502025	6.00083E-23	1.11688E-21
ZNF699	-0.487482106	6.02857E-23	1.121E-21
ANKFY1	-0.48738801	6.16135E-23	1.14462E-21
CHCHD5	0.487370094	6.18696E-23	1.14785E-21
SCAND1	0.487367784	6.19027E-23	1.14785E-21
HSPB11	0.487233239	6.38605E-23	1.18305E-21
BBS1	-0.487135069	6.53273E-23	1.2091E-21
ZNF593	0.487130922	6.539E-23	1.20914E-21
KRIT1	-0.487105923	6.57692E-23	1.2146E-21
ZMYND19	0.487103432	6.58071E-23	1.2146E-21
RASA1	-0.487067399	6.63578E-23	1.22363E-21
COMMD5	0.486955943	6.809E-23	1.25402E-21
TTC1	0.486953271	6.81321E-23	1.25402E-21
NOL12	0.486917877	6.86918E-23	1.26315E-21
AURKB	0.486710654	7.20607E-23	1.32388E-21
CLDN16	-0.486601825	7.38948E-23	1.35633E-21
UVRAG	-0.486596524	7.39853E-23	1.35674E-21
ARID4A	-0.486192373	8.12165E-23	1.48797E-21
FAM96B	0.486018287	8.4542E-23	1.54748E-21
SAMD4B	-0.485914248	8.65932E-23	1.58357E-21
RPL26L1	0.485829844	8.82932E-23	1.61317E-21
ANKRD12	-0.485605593	9.29713E-23	1.69709E-21
KIF22	0.48554798	9.42121E-23	1.71816E-21
CHMP6	0.48535689	9.84456E-23	1.79373E-21
SLC26A6	0.485198159	1.02104E-22	1.85869E-21
ITGA1	-0.485143857	1.03387E-22	1.88031E-21
STT3B	-0.484976847	1.0743E-22	1.95207E-21
P2RY1	-0.484911821	1.09047E-22	1.97964E-21
AKT3	-0.484888882	1.09622E-22	1.98828E-21
MAP2K1	-0.484823481	1.11281E-22	2.01652E-21
APOOL	-0.48481906	1.11394E-22	2.01673E-21
CRYBG3	-0.484787932	1.12192E-22	2.02935E-21
PJA2	-0.484780875	1.12374E-22	2.03079E-21

TMED4	-0.484639692	1.16074E-22	2.09576E-21
SLC10A6	-0.484627135	1.16409E-22	2.0999E-21
VPS13D	-0.484283893	1.2594E-22	2.26978E-21
C12orf45	0.48415929	1.29587E-22	2.33339E-21
CCDC82	-0.484072697	1.32183E-22	2.37798E-21
COX17	0.484067276	1.32347E-22	2.37878E-21
C6orf204	-0.48405289	1.32784E-22	2.38448E-21
MRPS26	0.48403318	1.33384E-22	2.39311E-21
USP25	-0.484006924	1.34189E-22	2.40374E-21
TOMM22	0.484005963	1.34218E-22	2.40374E-21
PPFIA1	-0.483931792	1.36516E-22	2.4427E-21
SSR2	0.483874949	1.38304E-22	2.47246E-21
SEC13	0.483845324	1.39245E-22	2.48705E-21
PPM1L	-0.48357991	1.47959E-22	2.64033E-21
NCOA1	-0.483455885	1.52214E-22	2.71382E-21
SLC25A39	0.483405561	1.53974E-22	2.74275E-21
RORA	-0.483347015	1.56047E-22	2.77552E-21
C1orf58	-0.483345743	1.56093E-22	2.77552E-21
PRDX1	0.483325718	1.56808E-22	2.78576E-21
TALDO1	0.483315551	1.57173E-22	2.78975E-21
ITPR1	-0.48323485	1.60097E-22	2.83911E-21
PHLPP2	-0.483228738	1.6032E-22	2.84055E-21
RAB3GAP1	-0.483064449	1.66447E-22	2.94649E-21
ZNF397	-0.4829765	1.69822E-22	3.00355E-21
C13orf31	-0.482918864	1.72069E-22	3.0406E-21
LAGE3	0.48271591	1.8022E-22	3.18181E-21
MRPL20	0.482701786	1.80802E-22	3.18925E-21
GPR125	-0.482592242	1.85372E-22	3.26698E-21
TIAF1	0.48255811	1.8682E-22	3.28958E-21
ZNF787	0.48254443	1.87403E-22	3.29693E-21
GPR157	-0.482520401	1.88432E-22	3.31211E-21
MPDZ	-0.482500214	1.893E-22	3.32444E-21
GMCL1	-0.482468725	1.90663E-22	3.34542E-21
DENND4C	-0.482340854	1.96297E-22	3.44124E-21
RSBN1L	-0.482144756	2.05257E-22	3.59423E-21
PSMA5	0.482142023	2.05385E-22	3.59423E-21
TXN	0.482019365	2.11196E-22	3.69269E-21
SCARB2	-0.481891238	2.1744E-22	3.79853E-21
NCRNA0012	-0.4818733	2.18329E-22	3.8096E-21
FKBP1A	0.48187073	2.18456E-22	3.8096E-21
THAP3	0.481796657	2.22166E-22	3.87089E-21
UQCR10	0.481707878	2.26693E-22	3.94633E-21
BRI3	0.481538865	2.35566E-22	4.0972E-21
MFSD5	0.481388115	2.43768E-22	4.23615E-21
COMMD7	0.48127816	2.49927E-22	4.3394E-21
BHLHE40	-0.481259332	2.50997E-22	4.35418E-21

EIF3D	0.481144174	2.5764E-22	4.46553E-21
ARHGAP32	-0.481135777	2.58131E-22	4.46854E-21
PKN2	-0.481133531	2.58262E-22	4.46854E-21
NDUFA13	0.481051405	2.63117E-22	4.54859E-21
RNASEH2A	0.480933228	2.70261E-22	4.66803E-21
KIF27	-0.480838994	2.76094E-22	4.76465E-21
PHC3	-0.48079619	2.78784E-22	4.80691E-21
HIGD2A	0.480723045	2.83441E-22	4.88299E-21
SERINC1	-0.480696144	2.85174E-22	4.90858E-21
PNPLA8	-0.480574238	2.93154E-22	5.0416E-21
SHROOM4	-0.480529034	2.96169E-22	5.08905E-21
COMMD1	0.480360439	3.07686E-22	5.28239E-21
EME1	0.480355126	3.08056E-22	5.28418E-21
SEC24D	-0.48012348	3.24622E-22	5.56355E-21
PKD2	-0.480099804	3.26364E-22	5.5886E-21
PGLS	0.47990861	3.40772E-22	5.8303E-21
SERPINB6	0.479724583	3.55231E-22	6.07247E-21
PCDHGB3	-0.479719817	3.55613E-22	6.07379E-21
STK38L	-0.479687506	3.58216E-22	6.11301E-21
UBE2Q2P1	-0.47938289	3.83699E-22	6.54226E-21
R3HDM2	-0.479276875	3.9298E-22	6.69478E-21
C22orf40	0.479196245	4.00187E-22	6.81173E-21
LYPLA2	0.479098183	4.09127E-22	6.95796E-21
C7orf50	0.479040811	4.14449E-22	7.04245E-21
SNX12	-0.478958095	4.22241E-22	7.16874E-21
SKI	-0.47891572	4.26289E-22	7.2313E-21
LRCH3	-0.478695145	4.47985E-22	7.59288E-21
DAAM1	-0.478562066	4.61598E-22	7.81694E-21
RBM10	0.478437168	4.74744E-22	8.03273E-21
NPAT	-0.478297283	4.89906E-22	8.28223E-21
ARHGEF6	-0.47829044	4.90659E-22	8.28794E-21
SACM1L	-0.478222705	4.98183E-22	8.40383E-21
SLC2A10	-0.478221082	4.98364E-22	8.40383E-21
CCDC124	0.478082554	5.14112E-22	8.66205E-21
NDUFA12	0.478067926	5.15804E-22	8.6832E-21
ERG	-0.478040428	5.18998E-22	8.72959E-21
TOPORS	-0.477962365	5.28172E-22	8.87641E-21
LOC388796	0.477857141	5.40792E-22	9.08083E-21
SPEN	-0.477830455	5.44039E-22	9.12767E-21
RPP40	0.47775842	5.52901E-22	9.26855E-21
FICD	-0.477751284	5.53787E-22	9.27558E-21
KCTD18	-0.477676915	5.631E-22	9.42365E-21
TRIM44	-0.477462078	5.9088E-22	9.87298E-21
RAPGEF5	-0.477461615	5.90941E-22	9.87298E-21
VARS	0.477410391	5.97763E-22	9.97858E-21
MOBKL1B	-0.477262175	6.17939E-22	1.03068E-20

DNLZ	0.477218102	6.24068E-22	1.04003E-20
LOC100272	-0.477201432	6.26401E-22	1.04304E-20
UNC5C	-0.47714636	6.34171E-22	1.0551E-20
C10orf4	0.4770767	6.44136E-22	1.07078E-20
COMMD3	0.477018523	6.52576E-22	1.08391E-20
WDR47	-0.477012741	6.53421E-22	1.08441E-20
KAT2B	-0.476949008	6.62804E-22	1.09906E-20
TRAPPC10	-0.476772507	6.89489E-22	1.14236E-20
SRPRB	0.476639202	7.10341E-22	1.17593E-20
MYH9	-0.476615996	7.14034E-22	1.18106E-20
S1PR1	-0.476605449	7.15719E-22	1.18287E-20
LARP1B	-0.476506437	7.31727E-22	1.20832E-20
GNAQ	-0.476436264	7.43285E-22	1.22639E-20
NDUFA3	0.476359609	7.56117E-22	1.24653E-20
MRPS12	0.476268119	7.71718E-22	1.2712E-20
NOP16	0.476237346	7.77036E-22	1.2789E-20
PPIB	0.476001981	8.18926E-22	1.34571E-20
EPB41L5	-0.476001685	8.1898E-22	1.34571E-20
NME3	0.475887	8.40191E-22	1.37942E-20
TRAF6	-0.475859814	8.45298E-22	1.38666E-20
COX6A1	0.47582569	8.51751E-22	1.3961E-20
AQR	-0.475640744	8.87582E-22	1.45363E-20
RPS3A	0.475601439	8.95386E-22	1.46521E-20
USP9X	-0.475504217	9.14981E-22	1.49605E-20
UFC1	0.475425216	9.31214E-22	1.52134E-20
PPP1R12A	-0.475176448	9.84207E-22	1.6066E-20
LOC100190	-0.475120035	9.96631E-22	1.62555E-20
PCF11	-0.475074626	1.00674E-21	1.6407E-20
ADAMTS12	-0.474953688	1.03417E-21	1.68402E-20
NCK1	-0.474937385	1.03793E-21	1.68875E-20
SURF6	0.474908116	1.0447E-21	1.69838E-20
ENSA	0.474660073	1.10387E-21	1.79311E-20
AUP1	0.474622976	1.113E-21	1.80647E-20
TECPR1	-0.474599259	1.11887E-21	1.81453E-20
PDZD2	-0.474561409	1.12831E-21	1.82835E-20
C11orf59	0.474521008	1.13848E-21	1.84331E-20
CPEB3	-0.47451573	1.13981E-21	1.84397E-20
TMEM147	0.474302521	1.19502E-21	1.93172E-20
C2orf28	0.4741611	1.23308E-21	1.99162E-20
PSMC4	0.474057804	1.26163E-21	2.03609E-20
TRAIP	0.474002277	1.27725E-21	2.05963E-20
LCA5	-0.473882159	1.31169E-21	2.11345E-20
SKIV2L	0.473833886	1.32578E-21	2.13443E-20
CDCA3	0.473448261	1.44388E-21	2.32268E-20
FAM190B	-0.473443777	1.44531E-21	2.32311E-20
AP1G1	-0.473436881	1.44752E-21	2.32478E-20

CACYBP	0.473430791	1.44947E-21	2.32604E-20
GHR	-0.473387902	1.46328E-21	2.34631E-20
EEF1E1	0.473262216	1.5045E-21	2.41047E-20
POLR2K	0.473150768	1.54201E-21	2.46858E-20
NDUFA7	0.47311085	1.55567E-21	2.48844E-20
HSP90AB1	0.472746329	1.68603E-21	2.6948E-20
TNS3	-0.472662283	1.71758E-21	2.74303E-20
HMGN2	0.472617148	1.73476E-21	2.76825E-20
ERCC4	-0.472525153	1.77031E-21	2.82272E-20
CBL	-0.472476866	1.78926E-21	2.85065E-20
PCDHGA11	-0.472425202	1.80975E-21	2.88099E-20
CHM	-0.472246628	1.88237E-21	2.99421E-20
PTPRM	-0.47214154	1.92645E-21	3.06186E-20
SPIN1	-0.472088283	1.94917E-21	3.09551E-20
RECQL4	0.472039392	1.97026E-21	3.12651E-20
SLC35F5	-0.471986208	1.99346E-21	3.1608E-20
TBCC	0.471974581	1.99856E-21	3.16511E-20
MICAL3	-0.471972786	1.99935E-21	3.16511E-20
UTP14C	-0.471968791	2.00111E-21	3.16538E-20
MOSPD2	-0.471846711	2.05559E-21	3.24898E-20
PTPMT1	0.47182561	2.06516E-21	3.2615E-20
XIAP	-0.471814187	2.07035E-21	3.26476E-20
CYSLTR2	-0.471813871	2.07049E-21	3.26476E-20
ZFPM2	-0.471677412	2.13357E-21	3.36155E-20
LSMD1	0.471435027	2.2503E-21	3.54267E-20
SUV39H1	0.471429465	2.25305E-21	3.5442E-20
GJD3	-0.471227445	2.35526E-21	3.70205E-20
NUDT16L1	0.471155174	2.39292E-21	3.75828E-20
FARSB	0.470988408	2.4821E-21	3.89527E-20
COMMD6	0.470974275	2.48981E-21	3.90429E-20
NF1	-0.470960066	2.49758E-21	3.9134E-20
MRPS11	0.470710229	2.6382E-21	4.13049E-20
L2HGDH	-0.470585492	2.7113E-21	4.2416E-20
IL1R1	-0.470504898	2.75959E-21	4.31375E-20
ANKRD39	0.470430878	2.80469E-21	4.38081E-20
C6orf1	0.470371699	2.84126E-21	4.43446E-20
PCYOX1	-0.470168903	2.9702E-21	4.63206E-20
TGOLN2	-0.470156971	2.97796E-21	4.64054E-20
STOML2	0.470133484	2.9933E-21	4.66079E-20
SFXN4	0.470117165	3.004E-21	4.6738E-20
KIF26A	-0.470093863	3.01935E-21	4.69402E-20
CWC15	0.470053855	3.04589E-21	4.73158E-20
SERINC5	-0.470041204	3.05432E-21	4.74099E-20
ARL5B	-0.469952843	3.11391E-21	4.82971E-20
HTRA2	0.469880778	3.16335E-21	4.90257E-20
PAPOLG	-0.469862776	3.17582E-21	4.91807E-20

LOC100130	0.469838725	3.19255E-21	4.94015E-20
CPSF3	0.469817162	3.20763E-21	4.95963E-20
NDUFS8	0.469718997	3.27717E-21	5.06322E-20
COG6	-0.469714766	3.2802E-21	5.06397E-20
PSMC3IP	0.469673449	3.30994E-21	5.10592E-20
GLO1	0.469634832	3.33797E-21	5.14518E-20
PTGES2	0.469550509	3.4E-21	5.23674E-20
EHBP1	-0.469536941	3.41009E-21	5.24821E-20
FAM115A	-0.469293477	3.59617E-21	5.53032E-20
THBD	-0.469287621	3.60076E-21	5.53312E-20
HS3ST3B1	-0.469203496	3.66744E-21	5.63123E-20
CDT1	0.469070474	3.77536E-21	5.79248E-20
ATF7IP	-0.468906841	3.91242E-21	5.99814E-20
PDP2	-0.468815911	3.99069E-21	6.11342E-20
QKI	-0.468801252	4.00345E-21	6.12826E-20
CCDC59	0.468688102	4.10331E-21	6.2763E-20
ZNF580	0.46860518	4.17805E-21	6.38571E-20
ABHD2	-0.468521035	4.25526E-21	6.49873E-20
UQCRH	0.468404113	4.36488E-21	6.66105E-20
PREPL	-0.468343762	4.42255E-21	6.74388E-20
PPIP5K2	-0.46827496	4.48921E-21	6.84029E-20
CTAGE1	-0.467820757	4.95478E-21	7.54392E-20
WRAP53	0.467788718	4.98936E-21	7.59076E-20
SEC61B	0.467777342	5.0017E-21	7.60372E-20
TRIM28	0.467707005	5.07863E-21	7.7148E-20
OPA1	-0.46766914	5.12053E-21	7.77252E-20
HDGFRP2	0.467257053	5.59914E-21	8.48887E-20
DICER1	-0.467255526	5.60099E-21	8.48887E-20
EIF3H	0.46724181	5.61766E-21	8.50766E-20
SIPA1L1	-0.467158216	5.72033E-21	8.65656E-20
C17orf61	0.467076406	5.82259E-21	8.80462E-20
TRAPPC2L	0.466999942	5.9198E-21	8.94482E-20
PCDHGA9	-0.466939791	5.99739E-21	9.05519E-20
IDE	-0.466912139	6.03339E-21	9.10265E-20
KPTN	0.466817243	6.15857E-21	9.28448E-20
WDR83	0.466747417	6.25232E-21	9.41868E-20
SUMO2	0.4666758	6.34992E-21	9.55241E-20
WDTC1	-0.466675244	6.35069E-21	9.55241E-20
MYST3	-0.466669215	6.35897E-21	9.55766E-20
NARF	0.466650344	6.38498E-21	9.5895E-20
PPP2R1A	0.466588154	6.47142E-21	9.71201E-20
FTL	0.466544508	6.53277E-21	9.7967E-20
ATP2A2	-0.466474054	6.63302E-21	9.93955E-20
LOC541471	0.466421175	6.70926E-21	1.00462E-19
FBXO11	-0.466349915	6.81336E-21	1.01944E-19
VCL	-0.466346469	6.81843E-21	1.01944E-19

YLPM1	-0.4662484	6.96441E-21	1.04048E-19
ATP11B	-0.46616321	7.09371E-21	1.05901E-19
COL4A3BP	-0.466158983	7.10019E-21	1.05918E-19
HECW2	-0.466041863	7.28199E-21	1.08548E-19
RGS7BP	-0.466012001	7.32907E-21	1.09169E-19
EIF2AK4	-0.465970617	7.39481E-21	1.10066E-19
INO80	-0.465902456	7.50436E-21	1.11587E-19
IK	0.465900058	7.50824E-21	1.11587E-19
TRMU	0.465814165	7.64864E-21	1.13589E-19
DDX21	-0.465790464	7.68784E-21	1.14086E-19
GAR1	0.465717003	7.81058E-21	1.15821E-19
SENP7	-0.465707957	7.82583E-21	1.15961E-19
MRPL36	0.465686358	7.86235E-21	1.16415E-19
UEVLD	-0.465546725	8.10256E-21	1.19883E-19
PXK	-0.465509489	8.16782E-21	1.20759E-19
ZEB1	-0.465471323	8.23526E-21	1.21666E-19
CALCRL	-0.46539505	8.37167E-21	1.23589E-19
HIAT1	-0.465238157	8.6593E-21	1.27741E-19
KLHL23	-0.465066539	8.98508E-21	1.32448E-19
INTS4L2	-0.464682197	9.75908E-21	1.43751E-19
WDR31	-0.464671916	9.78066E-21	1.43963E-19
PEX1	-0.464623942	9.88199E-21	1.45347E-19
WDR44	-0.464581717	9.97202E-21	1.46505E-19
DDX39	0.464580116	9.97545E-21	1.46505E-19
44084	-0.46431341	1.05633E-20	1.55025E-19
ZNF408	0.464300967	1.05916E-20	1.5524E-19
LNPEP	-0.464300071	1.05936E-20	1.5524E-19
CLN3	0.46428878	1.06193E-20	1.55503E-19
TRNAU1AP	0.464243135	1.07238E-20	1.56918E-19
ZMIZ1	-0.464191893	1.08424E-20	1.58536E-19
LDB2	-0.464169961	1.08935E-20	1.59166E-19
KLF7	-0.46405155	1.11737E-20	1.6314E-19
GPS2	0.464015926	1.12593E-20	1.64271E-19
PSMB5	0.463876249	1.16015E-20	1.69139E-19
CHCHD7	0.463827035	1.17245E-20	1.70807E-19
SGK196	-0.463812476	1.17611E-20	1.71216E-19
ETV2	0.463730583	1.19693E-20	1.74119E-19
DCAF5	-0.463687274	1.20808E-20	1.75613E-19
PPP1R3B	-0.463401224	1.28437E-20	1.86566E-19
TRIM78P	-0.463364132	1.2946E-20	1.87915E-19
DHRS13	0.46330254	1.31177E-20	1.90269E-19
LOC100271	-0.463235115	1.33082E-20	1.92892E-19
RPSAP9	0.463203801	1.33976E-20	1.94047E-19
IRF2	-0.4631722	1.34885E-20	1.9522E-19
DAAM2	-0.462952334	1.41374E-20	2.04464E-19
ACBD5	-0.462878682	1.43616E-20	2.07556E-19

CLINT1	-0.462788024	1.46424E-20	2.1146E-19
TSEN54	0.462736142	1.48055E-20	2.1366E-19
QPCTL	0.462706218	1.49004E-20	2.14874E-19
LOC220429	-0.462527512	1.54796E-20	2.23065E-19
LOC200030	-0.46251013	1.55371E-20	2.23732E-19
EPC2	-0.462456728	1.57151E-20	2.26132E-19
DCAF17	-0.462400553	1.59045E-20	2.28692E-19
TCOF1	0.462390569	1.59384E-20	2.29014E-19
POLR2A	-0.462363982	1.6029E-20	2.3015E-19
P2RX7	-0.462353404	1.60652E-20	2.30504E-19
CACNA2D1	-0.462300386	1.62479E-20	2.32956E-19
ZYG11B	-0.462280021	1.63186E-20	2.33801E-19
CUEDC2	0.462249736	1.64242E-20	2.35147E-19
C11orf74	0.462243092	1.64475E-20	2.35311E-19
SNAPC3	-0.462223554	1.65162E-20	2.36123E-19
ZNF81	-0.462124928	1.6867E-20	2.40965E-19
SOX7	-0.462120783	1.68819E-20	2.41005E-19
RAPGEF4	-0.46207085	1.70624E-20	2.43408E-19
USP47	-0.462029497	1.72134E-20	2.45386E-19
GPATCH8	-0.46191261	1.76472E-20	2.51391E-19
FILIP1L	-0.461885418	1.77497E-20	2.5267E-19
TOMM5	0.46176975	1.81921E-20	2.58783E-19
MGRN1	-0.461766189	1.82059E-20	2.58794E-19
LOC90834	-0.461726502	1.83603E-20	2.60803E-19
NDUFS3	0.461664581	1.86038E-20	2.64073E-19
KLHL15	-0.461431849	1.95477E-20	2.77274E-19
ZNF136	-0.461351401	1.98849E-20	2.81855E-19
MFAP3	-0.461325086	1.99964E-20	2.83234E-19
DNASE1L1	-0.461195778	2.05533E-20	2.90917E-19
ANKRD28	-0.461118107	2.08952E-20	2.95545E-19
AGPAT1	0.460937125	2.17137E-20	3.06904E-19
PYCRL	0.460926387	2.17632E-20	3.07386E-19
GCC2	-0.460879369	2.19814E-20	3.10248E-19
ZNF524	0.46084519	2.21414E-20	3.12285E-19
C21orf70	0.46078011	2.24492E-20	3.16403E-19
IREB2	-0.460347649	2.46045E-20	3.46533E-19
ATP5L	0.460337316	2.46584E-20	3.47047E-19
GPR75	-0.460212645	2.53181E-20	3.56022E-19
RCBTB1	-0.460210076	2.53319E-20	3.56022E-19
FRRS1	-0.460184889	2.54673E-20	3.57674E-19
DPCD	0.460062446	2.6136E-20	3.66806E-19
TCP11L2	-0.460023652	2.63514E-20	3.69569E-19
ZNF32	0.459979758	2.65973E-20	3.72747E-19
EIF1	0.459976535	2.66154E-20	3.72747E-19
NCRNA0021	0.459821115	2.75049E-20	3.84933E-19
PUSL1	0.459736127	2.80036E-20	3.91637E-19

TNS1	-0.459688893	2.82846E-20	3.95289E-19
TBRG4	0.459578896	2.89498E-20	4.04302E-19
EPHA4	-0.459510778	2.93694E-20	4.09875E-19
GABPA	-0.459482318	2.95465E-20	4.12058E-19
C5orf24	-0.459317721	3.05915E-20	4.26333E-19
ROBLD3	0.45930958	3.06441E-20	4.26768E-19
ZCCHC11	-0.45921916	3.12346E-20	4.34687E-19
MAN1A1	-0.459198442	3.13714E-20	4.36287E-19
MRPL40	0.459076938	3.21861E-20	4.47303E-19
BOLA1	0.45903108	3.24989E-20	4.51336E-19
KIAA1715	-0.458984591	3.28191E-20	4.55465E-19
NDUFS1	-0.458975873	3.28795E-20	4.55985E-19
ZDHHC21	-0.458887447	3.34982E-20	4.64243E-19
FAM126B	-0.458809299	3.40546E-20	4.71625E-19
ZZEF1	-0.458804001	3.40926E-20	4.71824E-19
PEX13	-0.45843328	3.68614E-20	5.09787E-19
PPCDC	0.458424329	3.69309E-20	5.10394E-19
PPM1B	-0.45834272	3.75707E-20	5.18876E-19
SF4	0.458317915	3.77673E-20	5.2095E-19
ATF7	-0.45831718	3.77731E-20	5.2095E-19
VPS4B	-0.458285118	3.80288E-20	5.24113E-19
C11orf67	0.458238505	3.84035E-20	5.28912E-19
NDUFS4	0.458216782	3.85794E-20	5.30967E-19
TARBP2	0.458187756	3.88157E-20	5.3385E-19
NR2F2	-0.458114184	3.94209E-20	5.418E-19
GAN	-0.458106478	3.94848E-20	5.42304E-19
CDC20	0.458086824	3.96483E-20	5.44174E-19
ZFP106	-0.45804781	3.99749E-20	5.48278E-19
GTF3A	0.45771645	4.28573E-20	5.87407E-19
TSEN34	0.457678964	4.3196E-20	5.91642E-19
TRIM35	-0.457632825	4.36164E-20	5.96991E-19
C12orf62	0.457621973	4.37159E-20	5.97941E-19
INTS6	-0.457472585	4.51083E-20	6.16563E-19
HIPK2	-0.457451741	4.5306E-20	6.1884E-19
CASD1	-0.457314234	4.66319E-20	6.36514E-19
IRAK3	-0.457279403	4.69738E-20	6.40741E-19
EFNB2	-0.457274316	4.70239E-20	6.40986E-19
C6orf48	0.457069877	4.90829E-20	6.68595E-19
SUPT3H	0.457047604	4.93125E-20	6.71264E-19
SKIL	-0.457038838	4.94032E-20	6.72039E-19
SLC5A4	-0.456905281	5.08049E-20	6.90635E-19
C3orf26	0.456848557	5.1412E-20	6.98411E-19
C5orf36	-0.456833385	5.15756E-20	7.00156E-19
EXOSC8	0.456792162	5.20227E-20	7.05744E-19
NEK9	-0.456658741	5.34961E-20	7.25237E-19
ATP10D	-0.456595906	5.42041E-20	7.34336E-19

ZNF619	-0.45643409	5.60701E-20	7.591E-19
ZXDA	-0.456399469	5.64775E-20	7.64096E-19
JAK2	-0.45627961	5.79105E-20	7.82952E-19
TAF9	0.456120071	5.98736E-20	8.08944E-19
PSMD8	0.456082922	6.034E-20	8.14693E-19
RNASEK	0.456077381	6.04099E-20	8.15084E-19
BCCIP	0.456059523	6.06357E-20	8.17576E-19
PUS1	0.456008295	6.12879E-20	8.2581E-19
PCDH17	-0.455977908	6.1678E-20	8.30505E-19
ARHGEF10	-0.455935412	6.22277E-20	8.3734E-19
KLF8	-0.455893887	6.27694E-20	8.4406E-19
ZNF436	-0.455731605	6.49316E-20	8.72546E-19
MTP18	0.455719246	6.50993E-20	8.74209E-19
SEL1L	-0.455616237	6.65132E-20	8.92594E-19
COX5B	0.455444893	6.89323E-20	9.24435E-19
PDAP1	0.455259371	7.16492E-20	9.60225E-19
ANO8	-0.455221212	7.2221E-20	9.67236E-19
SEC24A	-0.45519344	7.26399E-20	9.72194E-19
SRBD1	-0.455176988	7.28892E-20	9.74335E-19
KIAA1430	-0.455176423	7.28978E-20	9.74335E-19
ZC3H6	-0.455135042	7.35287E-20	9.82108E-19
LMBR1	-0.455066024	7.45929E-20	9.95655E-19
SLTM	-0.455002103	7.5592E-20	1.00832E-18
NEK7	-0.454827214	7.83935E-20	1.04498E-18
PIN4	0.454778849	7.91861E-20	1.05484E-18
CNO	0.454585962	8.24263E-20	1.09727E-18
DPM3	0.454540559	8.32078E-20	1.10694E-18
NDUFB10	0.454465461	8.45164E-20	1.12359E-18
PDE8A	-0.45439155	8.58241E-20	1.14022E-18
ITSN1	-0.454331544	8.69004E-20	1.15361E-18
UBE3C	-0.454328908	8.6948E-20	1.15361E-18
TECPR2	-0.454299688	8.74772E-20	1.15986E-18
ETFDH	-0.454283763	8.77669E-20	1.16293E-18
IGFBP5	-0.454094132	9.12906E-20	1.20881E-18
CCDC23	0.454026423	9.25822E-20	1.2251E-18
ZNF692	0.453968082	9.37095E-20	1.23919E-18
TGFBR2	-0.453955438	9.39556E-20	1.24162E-18
SIRT7	0.453857543	9.58825E-20	1.26625E-18
HAUS7	0.453820529	9.66212E-20	1.27516E-18
MTG1	0.453785916	9.7317E-20	1.28349E-18
C14orf135	-0.453762886	9.77827E-20	1.28878E-18
USF1	0.453739273	9.82625E-20	1.29424E-18
ARFGAP1	0.453466831	1.03968E-19	1.36849E-18
NR3C1	-0.453463406	1.04042E-19	1.36856E-18
PPP1R16A	0.453288406	1.07881E-19	1.41811E-18
MAP4K5	-0.45323686	1.09038E-19	1.43238E-18

SP3	-0.453223301	1.09344E-19	1.43545E-18
FLJ45340	-0.453186997	1.10168E-19	1.44532E-18
PTPRG	-0.45306883	1.12894E-19	1.48011E-18
GSDMD	0.453041329	1.13538E-19	1.48758E-18
DENND4A	-0.452993282	1.14672E-19	1.50144E-18
FAM198B	-0.452892248	1.17092E-19	1.53212E-18
MLST8	0.452873507	1.17547E-19	1.53706E-18
DTNBP1	0.452848381	1.18159E-19	1.54405E-18
FAM50A	0.452730698	1.21067E-19	1.58102E-18
DYNLL2	-0.452694008	1.21988E-19	1.592E-18
SIRT6	0.452637169	1.23429E-19	1.60974E-18
LMNA	0.452579262	1.24913E-19	1.62804E-18
EBF2	-0.452494565	1.27117E-19	1.65568E-18
RCHY1	-0.452419155	1.29111E-19	1.68055E-18
HSF1	0.452282378	1.32806E-19	1.72752E-18
TBC1D2B	-0.452253705	1.33594E-19	1.73664E-18
RSBN1	-0.452112296	1.37547E-19	1.78614E-18
PRR24	0.452111063	1.37582E-19	1.78614E-18
BTAF1	-0.452087775	1.38244E-19	1.79357E-18
OGFOD2	0.451979008	1.41378E-19	1.83304E-18
TLN1	-0.451970491	1.41627E-19	1.83506E-18
PPOX	0.451869864	1.44594E-19	1.87229E-18
AKT1S1	0.451809902	1.4639E-19	1.89433E-18
POLR3C	0.45177275	1.47515E-19	1.90764E-18
EIF4G3	-0.451716184	1.49243E-19	1.92873E-18
LOC100128	-0.45170942	1.49451E-19	1.93017E-18
SLC30A6	-0.451663398	1.50874E-19	1.94728E-18
LRRC37A4	-0.451491734	1.56299E-19	2.016E-18
AAAS	0.451424427	1.58479E-19	2.04278E-18
CASP8AP2	-0.451406467	1.59065E-19	2.04902E-18
CCDC90A	0.451364748	1.60436E-19	2.06534E-18
MRPL10	0.450849411	1.78357E-19	2.29456E-18
WBSCR22	0.45078388	1.80772E-19	2.32413E-18
MGC16384	-0.45076195	1.81588E-19	2.33311E-18
LYSMD4	0.450749722	1.82044E-19	2.33747E-18
EFHA2	-0.450685705	1.84452E-19	2.36685E-18
NBPF16	-0.450655871	1.85584E-19	2.37985E-18
PAF1	0.450652491	1.85713E-19	2.37997E-18
MAP4K3	-0.450646405	1.85945E-19	2.38141E-18
HMBOX1	-0.450392692	1.95876E-19	2.50699E-18
C2orf79	0.450331555	1.98347E-19	2.53698E-18
NDUFB3	0.449997588	2.12391E-19	2.71487E-18
CCDC101	0.449874493	2.1781E-19	2.78236E-18
ARHGAP20	-0.449741345	2.23826E-19	2.85736E-18
IRF3	0.449644568	2.283E-19	2.91262E-18
ZNF654	-0.449588729	2.30922E-19	2.94418E-18

RNF6	-0.449580011	2.31334E-19	2.94754E-18
POLL	0.449391375	2.40427E-19	3.06145E-18
CMC1	0.44930515	2.447E-19	3.11386E-18
RPL23P8	0.44917467	2.51308E-19	3.19591E-18
APOA1BP	0.449034572	2.58599E-19	3.28653E-18
FEM1C	-0.448803908	2.71058E-19	3.44268E-18
IKZF3	-0.448745267	2.74319E-19	3.48188E-18
PPP1CA	0.448700759	2.7682E-19	3.51138E-18
AFF4	-0.448693118	2.77251E-19	3.51461E-18
DIS3L	-0.448528824	2.86692E-19	3.63197E-18
ZFX	-0.448519055	2.87263E-19	3.6369E-18
C17orf106	0.448451519	2.91242E-19	3.68494E-18
HCFC1R1	0.448397954	2.94437E-19	3.72299E-18
KRBA2	-0.44829288	3.00805E-19	3.80109E-18
RPS4X	0.448155981	3.09304E-19	3.90602E-18
SPRED2	-0.44802662	3.17553E-19	4.00764E-18
ZEB2	-0.447818773	3.31261E-19	4.178E-18
ADRA1A	-0.447609342	3.45663E-19	4.35596E-18
ARHGAP23	-0.447607271	3.45808E-19	4.35596E-18
TBCA	0.447578069	3.47865E-19	4.3791E-18
SLC35D1	-0.44753481	3.50935E-19	4.41495E-18
WDR85	0.447525525	3.51597E-19	4.42049E-18
NT5C3L	0.447357076	3.63829E-19	4.57139E-18
PRRG4	-0.447287447	3.69007E-19	4.63352E-18
MED8	0.447253008	3.71594E-19	4.66047E-18
CUL5	-0.447252659	3.71621E-19	4.66047E-18
SNX19	-0.446894662	3.996E-19	5.00821E-18
C19orf70	0.44681279	4.06285E-19	5.08793E-18
C12orf57	0.446807826	4.06694E-19	5.08793E-18
BRWD3	-0.446807412	4.06728E-19	5.08793E-18
TAB2	-0.446796088	4.07662E-19	5.09641E-18
CDC42EP3	-0.446777959	4.09162E-19	5.11195E-18
C10orf128	-0.446691856	4.1636E-19	5.19863E-18
SLMAP	-0.446600744	4.24113E-19	5.29211E-18
PRKAG2	-0.446525801	4.30596E-19	5.36964E-18
TRRAP	-0.446499983	4.32852E-19	5.39439E-18
GLI4	0.446493165	4.33449E-19	5.39846E-18
MAZ	0.446484083	4.34247E-19	5.40502E-18
C9orf23	0.446244637	4.55799E-19	5.66973E-18
PITPNM2	-0.44607514	4.71688E-19	5.86371E-18
IFT20	0.446039725	4.75076E-19	5.90215E-18
CYC1	0.446001313	4.78778E-19	5.94443E-18
SLC39A7	0.44595352	4.83424E-19	5.99837E-18
C15orf61	0.445943045	4.84448E-19	6.00733E-18
LLPH	0.445925528	4.86165E-19	6.02488E-18
PMVK	0.445785606	5.00099E-19	6.1937E-18

THAP7	0.445758225	5.02872E-19	6.22417E-18
BAT2L1	-0.445708024	5.07994E-19	6.28366E-18
SMYD5	0.445681331	5.10738E-19	6.31369E-18
RRAGC	-0.445676368	5.1125E-19	6.3161E-18
PREX2	-0.445578944	5.21401E-19	6.43751E-18
ARFIP1	-0.445545294	5.24953E-19	6.47735E-18
MED27	0.445528732	5.2671E-19	6.495E-18
PPP1R14B	0.445502688	5.29485E-19	6.52518E-18
IDH3B	0.445416988	5.38717E-19	6.63484E-18
TK1	0.445393153	5.41312E-19	6.66269E-18
LOC389333	-0.445240936	5.58181E-19	6.86607E-18
SEC23A	-0.445202834	5.62484E-19	6.91473E-18
H2AFX	0.445140849	5.69553E-19	6.99732E-18
METTL11A	0.445104796	5.73705E-19	7.04398E-18
RPP38	0.445087365	5.75723E-19	7.06441E-18
STARD3	0.445069461	5.77804E-19	7.08557E-18
RBM22	0.444946293	5.92316E-19	7.25905E-18
ARF5	0.444919032	5.95576E-19	7.29452E-18
HIF1A	-0.444760115	6.14934E-19	7.52699E-18
SYNJ2	-0.444717716	6.20203E-19	7.58682E-18
CD2BP2	0.444661865	6.27211E-19	7.66784E-18
B4GALT7	0.444468283	6.5211E-19	7.96734E-18
SRM	0.444337723	6.69449E-19	8.17417E-18
C6orf226	0.444298365	6.74764E-19	8.23402E-18
MTMR14	0.444151982	6.949E-19	8.47454E-18
BTBD19	-0.444132181	6.97669E-19	8.5031E-18
POMP	0.444075004	7.05725E-19	8.59603E-18
ATG7	0.44403015	7.12108E-19	8.66848E-18
C14orf119	0.444024928	7.12855E-19	8.67227E-18
SHE	-0.44396741	7.21133E-19	8.76435E-18
PSMD9	0.443966226	7.21304E-19	8.76435E-18
NDUFC2	0.443924331	7.27395E-19	8.83297E-18
RPS6KA2	-0.443905704	7.30119E-19	8.86064E-18
SNHG3	0.443783097	7.48301E-19	9.07576E-18
GPR20	-0.443699519	7.60949E-19	9.22356E-18
NDUFAF3	0.443643992	7.69469E-19	9.32115E-18
NDUFB2	0.443574614	7.80245E-19	9.44595E-18
AR	-0.44351936	7.88934E-19	9.54534E-18
ARHGAP31	-0.443474367	7.9608E-19	9.62594E-18
MYOF	-0.443410101	8.06396E-19	9.74477E-18
CREBBP	-0.443221596	8.37422E-19	1.01136E-17
LYSMD3	-0.443187482	8.43161E-19	1.01767E-17
WDR4	0.443157964	8.48158E-19	1.02257E-17
TRMT1	0.44315745	8.48245E-19	1.02257E-17
LRRC40	-0.443107715	8.56731E-19	1.03217E-17
GTF3C5	0.443069767	8.63262E-19	1.03941E-17

COPE	0.443062805	8.64466E-19	1.04023E-17
NCKAP1	-0.44303416	8.69435E-19	1.04558E-17
NHLRC3	-0.442950323	8.8414E-19	1.06262E-17
TCF4	-0.442938858	8.8617E-19	1.06442E-17
ANP32A	0.442796106	9.11833E-19	1.09458E-17
44085	-0.442324359	1.00195E-18	1.20203E-17
SRRM2	-0.442215857	1.02388E-18	1.22761E-17
RNASEH2C	0.442098689	1.0481E-18	1.25589E-17
CYB561D2	0.442067697	1.0546E-18	1.26292E-17
RPL26	0.442059483	1.05633E-18	1.26423E-17
RAB6A	-0.442007655	1.0673E-18	1.27659E-17
H3F3A	0.441962277	1.077E-18	1.28742E-17
AKAP8L	0.441958446	1.07782E-18	1.28763E-17
ETFB	0.441781501	1.11652E-18	1.33306E-17
TJP1	-0.441776176	1.1177E-18	1.33367E-17
SERINC3	-0.441710428	1.13244E-18	1.35045E-17
NIPSNAP3B	-0.441702021	1.13434E-18	1.3519E-17
RRP7A	0.441627242	1.15136E-18	1.37137E-17
C19orf10	0.441596134	1.15851E-18	1.37907E-17
KIAA1468	-0.441490749	1.18308E-18	1.40747E-17
FKBP2	0.44148029	1.18555E-18	1.40956E-17
AKR1C3	0.44140708	1.20295E-18	1.4294E-17
TRIM22	-0.441389017	1.20728E-18	1.43369E-17
NPHP3	-0.441370043	1.21185E-18	1.43826E-17
HIVEP3	-0.441323916	1.22302E-18	1.45065E-17
TNFRSF10B	-0.441293285	1.2305E-18	1.45865E-17
BAZ1A	-0.441278861	1.23403E-18	1.46197E-17
C1orf91	0.441234079	1.24507E-18	1.47418E-17
G3BP2	-0.441222907	1.24784E-18	1.47658E-17
KIAA0776	-0.441055445	1.29009E-18	1.52567E-17
SCARF1	-0.441017829	1.29978E-18	1.5362E-17
IMP3	0.440923781	1.3243E-18	1.56426E-17
ZNF827	-0.440885593	1.33438E-18	1.57524E-17
BMP2K	-0.440880528	1.33573E-18	1.57589E-17
TMEM30A	-0.440841727	1.34606E-18	1.58714E-17
MEIS3P1	-0.440765318	1.36665E-18	1.60964E-17
WASH5P	0.440764893	1.36676E-18	1.60964E-17
PCGF1	0.440680472	1.38987E-18	1.63589E-17
COL8A1	-0.440674002	1.39165E-18	1.63702E-17
RAB33B	-0.440635049	1.40246E-18	1.64876E-17
DCTPP1	0.440630851	1.40363E-18	1.64916E-17
CENPV	0.440511633	1.43723E-18	1.68765E-17
CREBZF	-0.440420716	1.46339E-18	1.71735E-17
RPF2	0.440385621	1.47362E-18	1.72833E-17
DENND2C	-0.44032951	1.49011E-18	1.74664E-17
TSHZ3	-0.440318442	1.49338E-18	1.74832E-17

EEFSEC	0.440317066	1.49379E-18	1.74832E-17
LOC729176	-0.440315782	1.49417E-18	1.74832E-17
SAMHD1	-0.440283221	1.50385E-18	1.75845E-17
C12orf35	-0.440280717	1.50459E-18	1.75845E-17
LOC643387	0.440243101	1.51586E-18	1.77057E-17
SEC31A	-0.440221719	1.5223E-18	1.77705E-17
TSPYL4	-0.440083975	1.56443E-18	1.82517E-17
SDHAF1	0.439982445	1.59621E-18	1.86116E-17
RFNG	0.439960251	1.60325E-18	1.86827E-17
AUTS2	-0.439832244	1.64441E-18	1.91511E-17
SLC25A46	-0.439758724	1.66851E-18	1.9412E-17
IDS	-0.439757971	1.66876E-18	1.9412E-17
MPST	0.439674241	1.69665E-18	1.97249E-17
UBE2F	0.439597578	1.72258E-18	2.00147E-17
NDUFB1	0.439455505	1.77167E-18	2.0573E-17
RNF113A	0.439433129	1.77952E-18	2.06522E-17
FAM45B	-0.439331908	1.81549E-18	2.10574E-17
F8	-0.439292882	1.82955E-18	2.12081E-17
RDX	-0.439284407	1.83262E-18	2.12313E-17
THSD4	-0.439205879	1.86128E-18	2.15508E-17
STMN1	0.439070447	1.91174E-18	2.21222E-17
RBM27	-0.438933991	1.96395E-18	2.27131E-17
LOC729799	-0.438919325	1.96964E-18	2.27585E-17
CAPNS1	0.438917992	1.97016E-18	2.27585E-17
CYBRD1	-0.438863997	1.99127E-18	2.2989E-17
DBH	-0.438748838	2.03703E-18	2.35038E-17
RLF	-0.438731599	2.04397E-18	2.35702E-17
TIGD5	0.438719638	2.0488E-18	2.36122E-17
STAM2	-0.438706918	2.05395E-18	2.36578E-17
ATOX1	0.43864339	2.07984E-18	2.39422E-17
NCOR1	-0.438599922	2.09775E-18	2.41344E-17
FAM107B	-0.438453407	2.15922E-18	2.48273E-17
PPM1G	0.438431538	2.16855E-18	2.49201E-17
RNF125	-0.438346395	2.20523E-18	2.53271E-17
MAP3K13	-0.438330305	2.21224E-18	2.53929E-17
FBN1	-0.438083806	2.32227E-18	2.66406E-17
SLC35A5	-0.438077635	2.32509E-18	2.66576E-17
SNX9	-0.437952618	2.38301E-18	2.73059E-17
KIAA1712	-0.43794071	2.3886E-18	2.73542E-17
CENPW	0.437897051	2.4092E-18	2.75743E-17
ZNF275	-0.437671795	2.51832E-18	2.88066E-17
FRMD3	-0.437556897	2.57584E-18	2.94476E-17
PTPN21	-0.43754676	2.58097E-18	2.94894E-17
C19orf24	0.437496658	2.6065E-18	2.97641E-17
SLC35B2	0.437491966	2.60891E-18	2.97744E-17
ZFYVE9	-0.437334125	2.69105E-18	3.06944E-17

AOC3	-0.437207277	2.75891E-18	3.14503E-17
TOR1AIP1	-0.43714229	2.79432E-18	3.18358E-17
PSME2	0.437105631	2.81449E-18	3.20473E-17
TEP1	-0.43701756	2.86354E-18	3.25871E-17
PLEKHA7	-0.436866136	2.94985E-18	3.35501E-17
GNE	-0.436751808	3.0167E-18	3.42909E-17
ATP11A	-0.436410129	3.2255E-18	3.66434E-17
MRPS9	0.436326163	3.27895E-18	3.72175E-17
CD93	-0.436324883	3.27977E-18	3.72175E-17
MLLT4	-0.436240219	3.33456E-18	3.78177E-17
ADAMTS1	-0.436179507	3.3744E-18	3.82477E-17
APRT	0.436012593	3.48636E-18	3.94943E-17
C18orf25	-0.435984031	3.50588E-18	3.96928E-17
LRRFIP1	-0.435880733	3.57738E-18	4.04793E-17
GALNT11	-0.435867715	3.5865E-18	4.05443E-17
SMAD9	-0.435866711	3.5872E-18	4.05443E-17
RPL13AP3	0.435820873	3.61947E-18	4.08859E-17
SNTB2	-0.435778114	3.64984E-18	4.12055E-17
BCL2L2	-0.435708826	3.69957E-18	4.17433E-17
TMOD2	-0.435614765	3.76815E-18	4.2493E-17
ASH1L	-0.435610126	3.77156E-18	4.25075E-17
SCP2	-0.43558571	3.78958E-18	4.26864E-17
VDAC2	0.4355715	3.80011E-18	4.27601E-17
CEP170	-0.435571085	3.80042E-18	4.27601E-17
SPRY3	-0.43555089	3.81543E-18	4.29047E-17
ANP32B	0.435543784	3.82073E-18	4.294E-17
C19orf50	0.435100322	4.16593E-18	4.67933E-17
FTHL3	0.435003674	4.24514E-18	4.76561E-17
SLC10A7	-0.43498199	4.26311E-18	4.78309E-17
KLHDC1	-0.434861394	4.36445E-18	4.89403E-17
DUS1L	0.434846341	4.37726E-18	4.90564E-17
SRRM1	-0.434774407	4.43901E-18	4.97204E-17
ITGA9	-0.434671585	4.52875E-18	5.06971E-17
RUNDC2A	-0.434635958	4.56026E-18	5.10212E-17
DGUOK	0.434598219	4.59388E-18	5.13684E-17
AURKAIP1	0.434536949	4.64897E-18	5.19552E-17
LIMD1	-0.434487326	4.69407E-18	5.24298E-17
TRMT11	-0.434369858	4.80254E-18	5.36113E-17
AP1AR	-0.434320278	4.84906E-18	5.41002E-17
TRMT61A	0.434283589	4.88376E-18	5.4457E-17
MORN2	0.434172838	4.99002E-18	5.56107E-17
TUBA1C	0.434043563	5.11693E-18	5.69931E-17
SNX18	-0.434003709	5.15669E-18	5.74039E-17
SLC35C2	0.433989085	5.17136E-18	5.7535E-17
TMEM93	0.433957004	5.20368E-18	5.78622E-17
KIFC1	0.433925595	5.23551E-18	5.81837E-17

ZCCHC17	0.433719214	5.44948E-18	6.05278E-17
GAS2L3	-0.433364584	5.83741E-18	6.48004E-17
TUBA1B	0.433294357	5.91738E-18	6.56516E-17
F2R	-0.433285218	5.92787E-18	6.57314E-17
RPL10	0.433141854	6.09479E-18	6.75447E-17
FBXO22OS	0.433128044	6.11111E-18	6.76879E-17
RAB13	0.433100293	6.14404E-18	6.80147E-17
WRN	-0.432876034	6.4166E-18	7.09926E-17
MANEA	-0.432580825	6.79358E-18	7.51217E-17
NIPAL1	-0.43251652	6.87854E-18	7.6019E-17
SLAIN2	-0.431874376	7.78634E-18	8.60038E-17
TMEM150C	-0.431722261	8.01807E-18	8.85143E-17
FKBP3	0.431714127	8.03065E-18	8.86041E-17
THAP8	0.431709668	8.03755E-18	8.86311E-17
STX17	-0.431653924	8.12436E-18	8.95389E-17
TBK1	-0.431360825	8.5962E-18	9.46866E-17
UBA6	-0.431315794	8.67104E-18	9.54581E-17
HMBS	0.431232906	8.81048E-18	9.69396E-17
TKT	0.431077006	9.07873E-18	9.98359E-17
ZNF511	0.430995213	9.22266E-18	1.01363E-16
ELF2	-0.430948176	9.30644E-18	1.02227E-16
DIP2A	-0.430794223	9.58594E-18	1.05239E-16
UBL3	-0.430733591	9.69826E-18	1.06414E-16
ATP8B5P	0.430714784	9.73336E-18	1.0674E-16
SYNJ2BP	-0.430592812	9.96407E-18	1.0921E-16
PA2G4P4	0.430536621	1.00722E-17	1.10334E-16
FAM83G	-0.430479451	1.01833E-17	1.1149E-16
COX8A	0.430453125	1.02349E-17	1.11993E-16
PDE2A	-0.430350625	1.04382E-17	1.14155E-16
SOCS6	-0.430294379	1.05515E-17	1.15331E-16
IL7R	-0.429856238	1.14759E-17	1.25351E-16
NNAT	-0.429854012	1.14808E-17	1.25351E-16
ALKBH4	0.429815963	1.15648E-17	1.26199E-16
GOPC	-0.429783807	1.16363E-17	1.26909E-16
MRPL21	0.429755253	1.17001E-17	1.27524E-16
CIB1	0.429752874	1.17054E-17	1.27524E-16
ANKZF1	0.429645102	1.19494E-17	1.30111E-16
DOPEY1	-0.429636965	1.19681E-17	1.30243E-16
TANC1	-0.429452527	1.2398E-17	1.34848E-16
ST8SIA6	-0.42938142	1.25678E-17	1.3662E-16
C8orf37	-0.429337237	1.26744E-17	1.37704E-16
ZFP36L1	-0.429327381	1.26983E-17	1.37888E-16
ZNF354B	-0.429256158	1.28724E-17	1.39703E-16
BNIP1	0.429226343	1.2946E-17	1.40425E-16
KLF13	-0.429186775	1.30443E-17	1.41414E-16
DUSP23	0.429058431	1.33682E-17	1.44846E-16

CEP97	-0.428947705	1.36539E-17	1.47862E-16
VPS16	0.428931056	1.36974E-17	1.48252E-16
GLYR1	-0.428898497	1.37828E-17	1.49096E-16
TRAK1	-0.428661581	1.44204E-17	1.55908E-16
UCKL1	0.428628314	1.45122E-17	1.56816E-16
C21orf91	-0.42862418	1.45237E-17	1.56854E-16
RAPH1	-0.42832627	1.53726E-17	1.65932E-16
CYTH3	-0.428243688	1.56164E-17	1.68473E-16
C19orf43	0.428143816	1.59164E-17	1.71616E-16
GOLGA6L5	-0.428117379	1.59967E-17	1.72389E-16
RAD23A	0.428068916	1.61451E-17	1.73893E-16
CDC25C	0.427941399	1.65418E-17	1.7807E-16
SCRN3	-0.427859274	1.68024E-17	1.80778E-16
KIAA0562	-0.427789651	1.70264E-17	1.83089E-16
MGAT2	-0.427773044	1.70803E-17	1.8357E-16
SPRY1	-0.427729954	1.72209E-17	1.84981E-16
SNX25	-0.427667161	1.74278E-17	1.87102E-16
CCT5	0.427602607	1.7643E-17	1.89311E-16
NDUFA8	0.427510765	1.79538E-17	1.92541E-16
LSM10	0.427473764	1.80805E-17	1.93796E-16
MEGF9	-0.427442819	1.81871E-17	1.94834E-16
C14orf166	0.42731953	1.86182E-17	1.99344E-16
RAB18	-0.427203553	1.90328E-17	2.03674E-16
CACNA1C	-0.427065509	1.95381E-17	2.0897E-16
DDX41	0.426998703	1.97874E-17	2.11523E-16
RPL36AL	0.426988231	1.98268E-17	2.11799E-16
ZBTB9	0.426986171	1.98345E-17	2.11799E-16
CCDC132	-0.426951227	1.99665E-17	2.13093E-16
TM9SF2	-0.426944285	1.99928E-17	2.1326E-16
LOC440354	-0.426871851	2.02694E-17	2.16095E-16
ATP6V1F	0.426680601	2.10179E-17	2.23955E-16
C14orf167	-0.426580961	2.14186E-17	2.28102E-16
ACTR2	-0.426499583	2.17514E-17	2.31522E-16
SMARCA5	-0.426438479	2.20046E-17	2.34092E-16
MIB1	-0.426334088	2.24439E-17	2.38638E-16
TBC1D23	-0.426095177	2.3482E-17	2.49543E-16
ATP11C	-0.425936228	2.41986E-17	2.57022E-16
SNHG9	0.425917247	2.42856E-17	2.57808E-16
ZNF627	-0.425837315	2.46554E-17	2.61594E-16
CHSY1	-0.4257272	2.51738E-17	2.66952E-16
SPNS1	0.425564097	2.59615E-17	2.75159E-16
NAT1	-0.425461153	2.64711E-17	2.8041E-16
RANBP2	-0.425447465	2.65395E-17	2.80986E-16
CENPB	0.425392337	2.68172E-17	2.83775E-16
RBMS3	-0.425325642	2.71568E-17	2.87216E-16
NPM3	0.425308294	2.72459E-17	2.88005E-16

CDK5RAP3	0.425210661	2.77524E-17	2.93203E-16
C1orf192	-0.425116252	2.82509E-17	2.98312E-16
CUTC	0.425023801	2.87477E-17	3.03397E-16
HAX1	0.424989696	2.89331E-17	3.05192E-16
MED7	0.424794364	3.00178E-17	3.16466E-16
KIAA0664P3	-0.424778153	3.01096E-17	3.17266E-16
TPI1	0.424584771	3.12262E-17	3.28858E-16
BOLA3	0.424401271	3.23233E-17	3.40232E-16
LNX2	-0.424327859	3.27727E-17	3.4478E-16
ZC3H7B	-0.424141797	3.39395E-17	3.56866E-16
ZFP36	-0.42407258	3.43839E-17	3.61348E-16
ZSWIM6	-0.424052615	3.45131E-17	3.62515E-16
RCOR1	-0.423931221	3.53092E-17	3.70682E-16
LMAN2	0.423921844	3.53715E-17	3.7114E-16
NIPBL	-0.423639598	3.72962E-17	3.91129E-16
TMOD3	-0.4236333	3.73403E-17	3.91386E-16
GPKOW	0.423621142	3.74256E-17	3.92073E-16
CENPP	0.423603488	3.75498E-17	3.93167E-16
OIT3	-0.423576386	3.77412E-17	3.94964E-16
NOTCH2	-0.423543375	3.79756E-17	3.97208E-16
TNFSF8	-0.423500211	3.82843E-17	4.00227E-16
CCT4	0.423459646	3.85767E-17	4.03072E-16
DDX6	-0.423441615	3.87074E-17	4.04225E-16
UBE2T	0.423384705	3.91226E-17	4.08347E-16
MYBL2	0.423375784	3.91881E-17	4.08817E-16
FEM1B	-0.423370502	3.92269E-17	4.09008E-16
ANTXR2	-0.42334341	3.94267E-17	4.10875E-16
CHMP4B	0.423325631	3.95583E-17	4.12031E-16
NKTR	-0.42325831	4.00606E-17	4.17045E-16
NRIP1	-0.423233399	4.0248E-17	4.18777E-16
TRIM11	0.423225368	4.03086E-17	4.19189E-16
LUZP1	-0.423163245	4.07806E-17	4.23875E-16
C3orf75	0.423124792	4.10754E-17	4.26717E-16
NUP37	0.423102486	4.12474E-17	4.2828E-16
RHOJ	-0.423086381	4.1372E-17	4.2935E-16
CINP	0.423058992	4.15848E-17	4.31334E-16
ZFAND2A	0.423030079	4.18105E-17	4.3345E-16
C10orf18	-0.422981156	4.21953E-17	4.37211E-16
PELP1	0.422889674	4.29242E-17	4.44532E-16
TMEM133	-0.422736051	4.4176E-17	4.57259E-16
IGF2R	-0.422641729	4.49623E-17	4.65156E-16
TOB2	-0.422385958	4.71644E-17	4.87684E-16
ZBTB11	-0.42234441	4.7532E-17	4.91225E-16
ANKRD44	-0.422341689	4.75562E-17	4.91225E-16
HIST2H2AC	0.422331125	4.76501E-17	4.9194E-16
C9orf142	0.422248008	4.83957E-17	4.99379E-16

FAM76B	-0.422195381	4.88737E-17	5.0405E-16
TRAPPC5	0.422184568	4.89725E-17	5.04807E-16
MKLN1	-0.422148251	4.93058E-17	5.07979E-16
TOP1MT	0.42207088	5.00231E-17	5.15104E-16
VGLL3	-0.422048776	5.02299E-17	5.16966E-16
U2AF1	0.422018937	5.05105E-17	5.19585E-16
CNTN4	-0.422007342	5.06199E-17	5.20429E-16
C1orf50	0.422004706	5.06448E-17	5.20429E-16
PTPN9	-0.421978394	5.08941E-17	5.22721E-16
TSPO2	0.421953347	5.11325E-17	5.24899E-16
MMRN2	-0.421919667	5.14549E-17	5.27936E-16
NID2	-0.421905555	5.15905E-17	5.29055E-16
CPNE8	-0.421828449	5.23379E-17	5.36443E-16
SRPK2	-0.421729658	5.33111E-17	5.46137E-16
ZNF487	-0.421679733	5.38096E-17	5.50961E-16
FAT1	-0.421595054	5.46657E-17	5.59438E-16
DNAJC17	0.421470335	5.5951E-17	5.72123E-16
SETD2	-0.421469217	5.59626E-17	5.72123E-16
AVPR1A	-0.421446727	5.61976E-17	5.7423E-16
BRD1	-0.421441219	5.62553E-17	5.74525E-16
ITGB1	-0.421327697	5.74573E-17	5.865E-16
IL6R	-0.421202802	5.88089E-17	5.99989E-16
LPHN2	-0.421199464	5.88455E-17	6.00055E-16
LOC728323	-0.421044394	6.05682E-17	6.17306E-16
SLC5A3	-0.421030232	6.0728E-17	6.18618E-16
RNF7	0.420993337	6.11462E-17	6.22559E-16
NAV1	-0.420920244	6.1983E-17	6.30757E-16
SLC35B1	0.420908607	6.21173E-17	6.31801E-16
ZNF521	-0.420900776	6.22078E-17	6.32399E-16
C16orf59	0.420843804	6.28703E-17	6.38807E-16
THOC5	0.420720331	6.43298E-17	6.53303E-16
MLXIP	-0.420615929	6.55898E-17	6.6576E-16
TMEM203	0.420477029	6.73039E-17	6.8281E-16
TLR4	-0.420423019	6.79821E-17	6.8934E-16
TACC1	-0.420366685	6.86968E-17	6.96232E-16
GRWD1	0.420337404	6.90712E-17	6.9967E-16
PPIAL4G	0.420225533	7.05199E-17	7.13983E-16
STX5	0.420136654	7.16922E-17	7.25483E-16
UBXN7	-0.4201208	7.19033E-17	7.2725E-16
CLIP1	-0.42009978	7.21842E-17	7.2972E-16
CCDC107	0.419729778	7.73078E-17	7.81118E-16
SIRT1	-0.41967091	7.81552E-17	7.89085E-16
RNF8	0.419669513	7.81754E-17	7.89085E-16
LOC92659	0.419626807	7.87961E-17	7.94946E-16
TPD52L2	0.419442751	8.15268E-17	8.22079E-16
NOTCH4	-0.41943902	8.15831E-17	8.2223E-16

SPRY2	-0.419434125	8.1657E-17	8.22559E-16
TRAPPC6A	0.419390118	8.23246E-17	8.28865E-16
CCDC85A	-0.419364339	8.27182E-17	8.32406E-16
ZNF217	-0.41925381	8.44266E-17	8.49169E-16
EHMT1	-0.419238829	8.46608E-17	8.51095E-16
BOD1	0.419192085	8.53957E-17	8.5805E-16
USP6NL	-0.419186978	8.54764E-17	8.58427E-16
PCDHGB5	-0.419113104	8.66517E-17	8.69792E-16
SNHG7	0.419079137	8.71974E-17	8.74829E-16
KIF16B	-0.418932137	8.95982E-17	8.98464E-16
INHBA	-0.418888256	9.03274E-17	9.0532E-16
ARFRP1	0.418795684	9.18849E-17	9.20466E-16
NFAT5	-0.418527092	9.65545E-17	9.66759E-16
PICALM	-0.418439359	9.81297E-17	9.82037E-16
SMAD7	-0.418335977	1.00018E-16	1.00043E-15
KLF3	-0.418302059	1.00646E-16	1.0062E-15
NRM	0.418220575	1.02169E-16	1.02092E-15
CLIC4	-0.418108048	1.04309E-16	1.04178E-15
C14orf49	-0.418094824	1.04563E-16	1.0438E-15
ACD	0.418034085	1.0574E-16	1.05501E-15
ZFYVE28	-0.418028751	1.05844E-16	1.05552E-15
CDCA5	0.417974872	1.06899E-16	1.0652E-15
ZNF510	-0.41797377	1.06921E-16	1.0652E-15
LTBP1	-0.417917571	1.08033E-16	1.07574E-15
NSA2	0.417809686	1.102E-16	1.09677E-15
ABCA1	-0.417720264	1.12028E-16	1.11441E-15
DBT	-0.417701849	1.12408E-16	1.11763E-15
PHLDB2	-0.417674203	1.12981E-16	1.12277E-15
MFSD8	-0.417668521	1.131E-16	1.12338E-15
ZDHHC16	0.417650131	1.13483E-16	1.12662E-15
APOL6	-0.417570822	1.1515E-16	1.14261E-15
KIF13B	-0.417382073	1.19216E-16	1.18236E-15
EIF3I	0.417372353	1.19429E-16	1.18389E-15
CKS2	0.417256229	1.22004E-16	1.20882E-15
VHL	-0.417226323	1.22676E-16	1.21487E-15
DDX27	0.417139398	1.24651E-16	1.23381E-15
IMP4	0.417129571	1.24876E-16	1.23542E-15
HCG11	-0.417060365	1.26473E-16	1.2506E-15
TMEM111	0.417049884	1.26716E-16	1.25238E-15
CDK4	0.416968329	1.28627E-16	1.27064E-15
CLDN22	0.41694736	1.29123E-16	1.27491E-15
GABARAP	0.41681464	1.32306E-16	1.30568E-15
STAG3L2	-0.416793988	1.32808E-16	1.30999E-15
TANK	-0.416787693	1.32961E-16	1.31085E-15
KIAA0586	-0.416681563	1.35574E-16	1.33596E-15
NFE2L2	-0.416635349	1.36728E-16	1.34666E-15

GARNL3	-0.416627395	1.36927E-16	1.34795E-15
MPHOSPH9	-0.416546666	1.38969E-16	1.36737E-15
MED19	0.416437849	1.41767E-16	1.39422E-15
GPX4	0.41635202	1.44014E-16	1.41561E-15
PRCC	0.416339098	1.44355E-16	1.41827E-15
ATP6V0E1	0.416335562	1.44448E-16	1.41849E-15
TNFRSF10A	-0.416280118	1.45922E-16	1.43225E-15
CMIP	-0.416250756	1.46709E-16	1.43927E-15
TBCB	0.416195009	1.48214E-16	1.45331E-15
LRP6	-0.416144444	1.49592E-16	1.4661E-15
FYTTD1	-0.416025327	1.52888E-16	1.49767E-15
OAZ1	0.415934968	1.55436E-16	1.52188E-15
DCUN1D3	-0.415905664	1.56271E-16	1.52931E-15
GET4	0.415898351	1.56481E-16	1.5306E-15
LOC100129	-0.415805445	1.59161E-16	1.55606E-15
NUPL1	-0.415770116	1.60193E-16	1.56538E-15
TRAF4	0.41571326	1.61866E-16	1.58095E-15
UFD1L	0.415655735	1.63576E-16	1.59687E-15
UQCRHL	0.415620058	1.64646E-16	1.60653E-15
SH3D19	-0.415589013	1.65583E-16	1.61488E-15
FAM165B	0.415582591	1.65777E-16	1.61598E-15
KIAA1958	-0.415542104	1.67008E-16	1.62718E-15
KLHL2	-0.415517675	1.67754E-16	1.63366E-15
BARD1	-0.415362635	1.72571E-16	1.67974E-15
ANAPC7	0.415340866	1.73258E-16	1.68496E-15
SPC25	0.415340292	1.73276E-16	1.68496E-15
MXRA5	-0.415330856	1.73575E-16	1.68704E-15
ZCCHC14	-0.415289754	1.74882E-16	1.69891E-15
SENP6	-0.415257805	1.75905E-16	1.70801E-15
LOC440356	0.415182731	1.78331E-16	1.73073E-15
MRPS33	0.415160136	1.79067E-16	1.73703E-15
C1orf57	0.415150705	1.79375E-16	1.73917E-15
TXNDC17	0.415142551	1.79642E-16	1.74091E-15
P2RY12	-0.415115962	1.80516E-16	1.74853E-15
PRICKLE4	-0.415061124	1.8233E-16	1.76524E-15
RAPGEF1	-0.414978993	1.85081E-16	1.79101E-15
HPS5	-0.414971962	1.85319E-16	1.79243E-15
IMPACT	-0.414968126	1.85448E-16	1.79282E-15
ITGA8	-0.414925328	1.86901E-16	1.80549E-15
GMNN	0.41492417	1.8694E-16	1.80549E-15
BBS10	-0.414858495	1.89191E-16	1.82634E-15
FAM38B	-0.414792654	1.91474E-16	1.84749E-15
PLEKHJ1	0.414785333	1.9173E-16	1.84906E-15
LOC202181	-0.414565382	1.99566E-16	1.9237E-15
MFSD9	-0.414552587	2.00032E-16	1.92721E-15
RASEF	-0.414550038	2.00124E-16	1.92721E-15

PACSIN2	-0.414492943	2.02215E-16	1.94641E-15
KANK2	-0.414379954	2.06416E-16	1.98588E-15
C1orf123	0.414362026	2.0709E-16	1.99141E-15
ZNF91	-0.414082331	2.17896E-16	2.0943E-15
44081	-0.414001275	2.21129E-16	2.12436E-15
ZNF562	-0.413988692	2.21636E-16	2.12753E-15
FKBP8	0.413987758	2.21673E-16	2.12753E-15
ZFR	-0.41397886	2.22032E-16	2.12994E-15
FRMD6	-0.413971241	2.22339E-16	2.13187E-15
TTC7B	-0.41392044	2.24401E-16	2.1506E-15
DNAJC22	-0.413906663	2.24963E-16	2.15495E-15
PWP1	0.413883308	2.2592E-16	2.16307E-15
SYNC	0.413856005	2.27043E-16	2.17278E-15
SLC30A1	-0.413837855	2.27793E-16	2.17891E-15
GGCT	0.413832057	2.28032E-16	2.18016E-15
NUDT3	0.413804611	2.29172E-16	2.19E-15
FBXW11	-0.413765343	2.30812E-16	2.20461E-15
VPS13A	-0.413760323	2.31022E-16	2.20557E-15
C12orf66	-0.413756066	2.31201E-16	2.20621E-15
H2AFZ	0.41368061	2.3439E-16	2.23557E-15
DDX54	0.413661882	2.35188E-16	2.24211E-15
MPHOSPH8	-0.413639164	2.3616E-16	2.25029E-15
GVIN1	-0.413636546	2.36272E-16	2.25029E-15
PTTG3P	0.413614814	2.37206E-16	2.2581E-15
CDK13	-0.413529587	2.40902E-16	2.2922E-15
DKK2	-0.413514738	2.41552E-16	2.29728E-15
MTMR12	-0.413484853	2.42865E-16	2.30867E-15
RFC4	0.413415697	2.45931E-16	2.3367E-15
SRP14	0.413382255	2.47427E-16	2.34979E-15
FAM136A	0.413347328	2.48999E-16	2.36359E-15
HEATR3	-0.413288233	2.51681E-16	2.38791E-15
EYA3	-0.412918362	2.69124E-16	2.55219E-15
KIF9	0.412819244	2.73996E-16	2.59716E-15
COL12A1	-0.412796953	2.75104E-16	2.60642E-15
PPIE	0.412770427	2.76427E-16	2.61771E-15
LRRC8A	-0.412730847	2.78414E-16	2.63498E-15
IL1RL1	-0.412728839	2.78516E-16	2.63498E-15
RBM9	-0.41249615	2.90487E-16	2.74694E-15
IPPK	-0.412457586	2.9252E-16	2.76485E-15
TAF2	-0.412430364	2.93963E-16	2.77717E-15
PPP2R5D	0.41239564	2.95814E-16	2.79333E-15
CSNK2A1P	-0.412314969	3.00158E-16	2.83301E-15
NFKBIE	0.412309762	3.0044E-16	2.83433E-15
EDEM1	-0.412268457	3.02691E-16	2.85421E-15
SEC23IP	-0.412243422	3.04063E-16	2.86579E-15
FLJ10213	-0.412221862	3.05249E-16	2.87561E-15

YDJC	0.412184818	3.07299E-16	2.89355E-15
LOC283856	-0.41201961	3.16604E-16	2.97975E-15
SYNPO2	-0.411964255	3.19783E-16	3.00825E-15
CTBS	-0.41195276	3.20447E-16	3.01308E-15
PTGES3	0.41182845	3.27716E-16	3.07997E-15
FARS2	0.41170796	3.34915E-16	3.14615E-15
COX18	-0.411682023	3.36485E-16	3.15941E-15
TAL1	-0.411657647	3.37967E-16	3.17183E-15
B3GNTL1	0.41164142	3.38958E-16	3.17962E-15
FAM13B	-0.411616811	3.40465E-16	3.19226E-15
PXDN	-0.411491928	3.48215E-16	3.26226E-15
DOCK4	-0.411491226	3.48259E-16	3.26226E-15
MCCC2	-0.411483639	3.48735E-16	3.26519E-15
GTF3C6	0.411474993	3.49279E-16	3.26875E-15
FAM65C	-0.411385766	3.54939E-16	3.32015E-15
NDEL1	-0.411365104	3.56262E-16	3.33097E-15
GAPDH	0.411274146	3.62146E-16	3.38439E-15
NAT14	0.411257588	3.63227E-16	3.39291E-15
CCDC39	-0.411204173	3.66737E-16	3.42409E-15
COX5A	0.411192162	3.67531E-16	3.42989E-15
SNHG8	0.411170165	3.68989E-16	3.44188E-15
MAPK1	-0.411161534	3.69563E-16	3.44562E-15
COL25A1	-0.41114945	3.70368E-16	3.45151E-15
RSRC1	-0.41103842	3.77842E-16	3.51952E-15
GDF7	-0.411019311	3.79143E-16	3.52999E-15
CYP2U1	-0.410805159	3.94031E-16	3.66688E-15
STX4	0.410723695	3.99843E-16	3.71923E-15
SLC25A26	0.410711554	4.00717E-16	3.72562E-15
ZNFX1	-0.410585677	4.09883E-16	3.80906E-15
CFDP1	0.410492599	4.16794E-16	3.87108E-15
ATP6V0B	0.410490567	4.16946E-16	3.87108E-15
HLCS	-0.410448632	4.20098E-16	3.89853E-15
RBCK1	0.410444955	4.20375E-16	3.89929E-15
KIF2C	0.410259004	4.34644E-16	4.02976E-15
CIDECP	0.410245131	4.35727E-16	4.03793E-15
PAFAH1B3	0.410190239	4.4004E-16	4.076E-15
CCNB1	0.410117614	4.4581E-16	4.12651E-15
ZNF90	0.410116398	4.45907E-16	4.12651E-15
UTP18	0.4100974	4.47429E-16	4.13867E-15
UCK2	0.410058125	4.50592E-16	4.16599E-15
FAM100B	0.409853376	4.67438E-16	4.31974E-15
TPRA1	0.409777543	4.73833E-16	4.37681E-15
ZNF224	-0.409662681	4.83684E-16	4.46572E-15
XAB2	0.409629225	4.8659E-16	4.49047E-15
MLL	-0.409486884	4.99149E-16	4.60424E-15
SSU72	0.409468668	5.0078E-16	4.61714E-15

USP16	-0.409352124	5.11333E-16	4.71226E-15
TPRN	0.409331386	5.13233E-16	4.72759E-15
SFRS3	0.409323057	5.13999E-16	4.73245E-15
HMGXB4	-0.409266077	5.19264E-16	4.77872E-15
ZP3	0.409133374	5.31733E-16	4.89121E-15
LOC729991	0.409118736	5.33127E-16	4.90176E-15
ARFGAP2	0.409094473	5.35444E-16	4.9208E-15
ABHD14A	0.409075779	5.37236E-16	4.93499E-15
ABL1	-0.409063365	5.3843E-16	4.94367E-15
USP8	-0.409004654	5.44109E-16	4.99352E-15
LEMD3	-0.408962341	5.48238E-16	5.0291E-15
NPFF	0.408811534	5.63208E-16	5.16403E-15
YIF1A	0.408772667	5.6713E-16	5.19761E-15
SYTL2	-0.408768411	5.67561E-16	5.19917E-15
NTHL1	0.408758693	5.68547E-16	5.2058E-15
UACA	-0.408578758	5.87104E-16	5.37324E-15
ERCC6	-0.40856399	5.88653E-16	5.38495E-15
DLG1	-0.408556674	5.89422E-16	5.38951E-15
TMEM192	-0.408549327	5.90196E-16	5.3941E-15
MBLAC2	-0.408394595	6.06712E-16	5.54251E-15
PSMG1	0.408352273	6.11309E-16	5.58194E-15
GIMAP6	-0.408321195	6.14705E-16	5.61038E-15
MBNL2	-0.40824774	6.22808E-16	5.68079E-15
ZNF37A	-0.408246097	6.2299E-16	5.68079E-15
ZMAT1	-0.408166429	6.31899E-16	5.75838E-15
TMEM42	0.40816484	6.32078E-16	5.75838E-15
TTC39B	-0.408106864	6.38641E-16	5.81479E-15
MAMDC2	-0.408105002	6.38853E-16	5.81479E-15
FBXO8	-0.408059432	6.4406E-16	5.85951E-15
KIAA1671	-0.408052346	6.44874E-16	5.86423E-15
MAP2K2	0.407991194	6.51936E-16	5.92574E-15
ZCCHC2	-0.407982859	6.52905E-16	5.93184E-15
ZNF76	0.407865674	6.66671E-16	6.05415E-15
UBE2I	0.407861693	6.67143E-16	6.05568E-15
MRP63	0.407764469	6.78788E-16	6.15669E-15
HGS	0.40776363	6.7889E-16	6.15669E-15
UTP6	0.407752303	6.80259E-16	6.1663E-15
MAGOHB	0.407581634	7.0123E-16	6.3535E-15
CYSLTR1	-0.407544782	7.05841E-16	6.39237E-15
TMEM179B	0.40750146	7.11299E-16	6.43887E-15
BAX	0.407399112	7.24359E-16	6.55412E-15
TMX3	-0.407361149	7.29263E-16	6.59549E-15
DHFRL1	-0.407358388	7.29621E-16	6.59573E-15
RARS	0.407251539	7.43605E-16	6.7191E-15
ABHD12	0.407198998	7.50578E-16	6.77903E-15
FBXO21	-0.40710517	7.6319E-16	6.88981E-15

NECAP1	-0.407079104	7.6673E-16	6.91864E-15
BRMS1L	-0.407073351	7.67514E-16	6.92257E-15
TMEM219	0.406969376	7.81811E-16	7.04834E-15
LAS1L	0.406903241	7.91041E-16	7.12832E-15
ZC3H7A	-0.406900312	7.91453E-16	7.1288E-15
ERCC1	0.406888801	7.93071E-16	7.14015E-15
FAM24B	0.406784667	8.07858E-16	7.26999E-15
STXBP3	-0.406777142	8.08937E-16	7.27641E-15
ZSCAN29	-0.406736801	8.14745E-16	7.32535E-15
NOP58	0.406688351	8.21776E-16	7.38523E-15
UROD	0.406606999	8.33714E-16	7.48914E-15
FCHSD2	-0.406602732	8.34345E-16	7.49142E-15
ABCC9	-0.406549896	8.42195E-16	7.5585E-15
RDM1	0.40654385	8.43098E-16	7.5632E-15
KLHL9	-0.406424628	8.61099E-16	7.7212E-15
C20orf24	0.406390699	8.6629E-16	7.76266E-15
ALDH1A3	-0.406389314	8.66502E-16	7.76266E-15
PLXNC1	-0.406272225	8.84659E-16	7.92175E-15
C12orf44	0.406145251	9.04769E-16	8.09819E-15
LOC728989	-0.406084638	9.14527E-16	8.18185E-15
C8orf30A	0.406071572	9.16644E-16	8.19711E-15
BNIP2	-0.406040447	9.21706E-16	8.23868E-15
CCR4	-0.405986768	9.30501E-16	8.31356E-15
LMAN1	-0.405918092	9.41873E-16	8.41139E-15
CFL1	0.405848067	9.53609E-16	8.51238E-15
PVRL3	-0.405832658	9.5621E-16	8.53178E-15
RUVBL1	0.405789219	9.63582E-16	8.5937E-15
IKZF4	-0.405742525	9.71568E-16	8.66105E-15
RBM25	-0.405717076	9.75948E-16	8.6962E-15
SRA1	0.405547294	1.00567E-15	8.95702E-15
C1orf55	-0.405506419	1.01296E-15	9.01789E-15
LOC100132	-0.405459761	1.02134E-15	9.08844E-15
KDM6A	-0.405337772	1.04357E-15	9.28215E-15
ANKRD56	-0.40529755	1.05101E-15	9.34259E-15
CYP4V2	-0.405295941	1.05131E-15	9.34259E-15
ACADM	-0.405079588	1.09221E-15	9.70176E-15
LARP7	-0.404919866	1.1234E-15	9.97441E-15
UBE2D2	0.404758736	1.15576E-15	1.02571E-14
ZNF460	-0.404708374	1.16606E-15	1.03439E-14
ZNF828	-0.404667494	1.17448E-15	1.0414E-14
BCO2	-0.404643614	1.17943E-15	1.04532E-14
C16orf53	0.404531727	1.2029E-15	1.06565E-14
NDUFB8	0.404431492	1.22431E-15	1.08398E-14
NPM1	0.404429772	1.22468E-15	1.08398E-14
HDGF	0.404414013	1.22808E-15	1.08651E-14
AIP	0.404387925	1.23373E-15	1.09102E-14

ALDH1L2	-0.40437579	1.23637E-15	1.09287E-14
C7orf27	0.404351125	1.24175E-15	1.09713E-14
PDCL3	0.404260765	1.26164E-15	1.11422E-14
KDM4A	-0.40419681	1.27591E-15	1.12632E-14
AP4E1	-0.404142011	1.28826E-15	1.13672E-14
NCRNA0018	0.404066101	1.30557E-15	1.15148E-14
UHRF1BP1L	-0.404045535	1.3103E-15	1.15514E-14
TNFSF14	-0.404032999	1.31319E-15	1.15717E-14
PEX16	0.403934792	1.33604E-15	1.17679E-14
ADNP	-0.403915926	1.34048E-15	1.18018E-14
WNK1	-0.403871899	1.35089E-15	1.18881E-14
MYO1B	-0.403812547	1.36504E-15	1.20074E-14
STAG2	-0.403774983	1.37407E-15	1.20815E-14
RNF38	-0.403744927	1.38134E-15	1.21401E-14
C5orf42	-0.403664535	1.40098E-15	1.23072E-14
C2orf68	0.403640923	1.4068E-15	1.23529E-14
SLC1A1	-0.403517693	1.43755E-15	1.26173E-14
FBXO38	-0.403496076	1.44301E-15	1.26597E-14
RUNDC2C	-0.403430701	1.45965E-15	1.28001E-14
ENPEP	-0.403408425	1.46536E-15	1.28445E-14
CEMP1	0.403355067	1.47914E-15	1.29596E-14
FILIP1	-0.403063095	1.5568E-15	1.36328E-14
UBE2C	0.403061059	1.55735E-15	1.36328E-14
TBC1D5	-0.402948381	1.58839E-15	1.38984E-14
NAIP	-0.402751301	1.64414E-15	1.43799E-14
PAPD4	-0.402737852	1.64802E-15	1.44075E-14
DMXL2	-0.402725022	1.65172E-15	1.44335E-14
RSL1D1	0.402668612	1.6681E-15	1.45703E-14
STX8	0.402660612	1.67044E-15	1.45843E-14
ZNF12	-0.402611946	1.68472E-15	1.47025E-14
ZNF644	-0.402557911	1.70071E-15	1.48356E-14
BRWD1	-0.402440747	1.73591E-15	1.5136E-14
FBXL14	-0.40238635	1.7525E-15	1.52739E-14
CHRAC1	0.402346474	1.76475E-15	1.5374E-14
INO80D	-0.402232216	1.80033E-15	1.56771E-14
FAM188A	-0.402109571	1.83931E-15	1.60096E-14
INPP4A	-0.402044992	1.86017E-15	1.6184E-14
CXorf38	-0.401963296	1.88688E-15	1.64093E-14
RAD51C	0.401955184	1.88956E-15	1.64254E-14
NUDT2	0.401898398	1.90837E-15	1.65817E-14
NUMA1	-0.401868621	1.91831E-15	1.66608E-14
HS3ST3A1	-0.401863875	1.9199E-15	1.66673E-14
MCM7	0.401834824	1.92966E-15	1.67407E-14
CAB39	-0.40183369	1.93004E-15	1.67407E-14
BRIX1	0.401550345	2.02779E-15	1.75809E-14
NGDN	0.401482537	2.05189E-15	1.77821E-14

BBX	-0.401413363	2.07677E-15	1.79899E-14
TRAPPC3	0.40132386	2.1094E-15	1.82646E-14
ZC3H12C	-0.401155009	2.17232E-15	1.88013E-14
FTO	-0.40110941	2.18963E-15	1.89429E-14
ZNF621	-0.401103604	2.19185E-15	1.89538E-14
RPL23AP53	-0.401090456	2.19687E-15	1.8989E-14
KIAA0430	-0.401072734	2.20365E-15	1.90394E-14
RPL22L1	0.401031979	2.21933E-15	1.91665E-14
ZBTB39	-0.401013602	2.22644E-15	1.92196E-14
ESR1	-0.400981643	2.23885E-15	1.93183E-14
DKFZp686O	-0.400959861	2.24735E-15	1.93833E-14
CXorf56	-0.40091561	2.26471E-15	1.95246E-14
TXNIP	-0.400813065	2.30546E-15	1.98672E-14
GPR173	-0.400782045	2.31792E-15	1.9966E-14
SH3RF1	-0.400766773	2.32408E-15	2.00104E-14
FAM21A	-0.400718573	2.34363E-15	2.017E-14
TMSB10	0.400695528	2.35304E-15	2.02342E-14
MMAB	0.400695337	2.35312E-15	2.02342E-14
FARP2	-0.400598857	2.3929E-15	2.05673E-14
SNHG5	0.400584497	2.39887E-15	2.06098E-14
TRAPPC1	0.400566735	2.40628E-15	2.06646E-14
ADCK5	0.400551216	2.41278E-15	2.07114E-14
FTSJ3	0.400536051	2.41914E-15	2.07571E-14
MCC	-0.400468681	2.44761E-15	2.09923E-14
TMEM204	-0.400449255	2.45588E-15	2.10542E-14
NAGPA	0.400438713	2.46038E-15	2.10837E-14
ZMYND17	0.400370868	2.48953E-15	2.13243E-14
MGC70857	0.400348658	2.49915E-15	2.13975E-14
UBE2V1	0.400341117	2.50242E-15	2.14163E-14
MBLAC1	0.400302083	2.51943E-15	2.15526E-14
B3GAT3	0.400291993	2.52385E-15	2.15811E-14
MAN1B1	0.400161821	2.5815E-15	2.20646E-14
ZDHHC17	-0.400139168	2.59166E-15	2.2142E-14
STUB1	0.400129262	2.59612E-15	2.21706E-14
WDR37	-0.400102718	2.6081E-15	2.22633E-14
ZBTB26	-0.40002664	2.64274E-15	2.25493E-14
FRG1	0.400011742	2.64957E-15	2.2598E-14
FLT4	-0.399906422	2.69839E-15	2.30045E-14
DNAJB11	0.399885463	2.70821E-15	2.30719E-14
MYOCD	-0.399884605	2.70861E-15	2.30719E-14
TNKS	-0.399726246	2.78396E-15	2.37035E-14
LOC388955	0.399509035	2.89065E-15	2.46014E-14
ZNHIT1	0.399498854	2.89575E-15	2.46343E-14
ARFIP2	0.399485833	2.90228E-15	2.46793E-14
COL4A1	-0.399450128	2.92027E-15	2.48217E-14
BRD3	-0.399319624	2.98696E-15	2.53777E-14

HERC4	-0.399236995	3.02995E-15	2.5732E-14
LOC728024	-0.39922107	3.03831E-15	2.57919E-14
TWSG1	-0.399210918	3.04365E-15	2.58262E-14
EIF4EBP3	0.399182104	3.05885E-15	2.59442E-14
CCDC146	-0.399172559	3.0639E-15	2.5976E-14
EEA1	-0.399124652	3.08938E-15	2.61808E-14
PQLC2	0.399111201	3.09657E-15	2.62306E-14
RAC1	0.399087803	3.10912E-15	2.63257E-14
TMED7	-0.39908157	3.11247E-15	2.63429E-14
C1orf152	-0.399072557	3.11732E-15	2.63728E-14
DLC1	-0.398909574	3.20636E-15	2.71071E-14
PRKCH	-0.398908691	3.20685E-15	2.71071E-14
MIER3	-0.398888535	3.21803E-15	2.71901E-14
LOC729603	-0.398799594	3.26784E-15	2.75992E-14
C21orf57	0.398619972	3.37075E-15	2.84563E-14
OVCA2	0.398498142	3.44235E-15	2.90485E-14
RAG1AP1	0.398482736	3.45151E-15	2.91134E-14
SNRNP25	0.398475097	3.45606E-15	2.91395E-14
LDLR	-0.398432179	3.48174E-15	2.93436E-14
VPS36	-0.398405961	3.49752E-15	2.94641E-14
DDX60	-0.398351349	3.53061E-15	2.97302E-14
ZMYM2	-0.398308278	3.55692E-15	2.99392E-14
DDX51	0.398249142	3.59336E-15	3.02331E-14
KRT10	0.398226204	3.60759E-15	3.034E-14
SNORA76	0.398223116	3.60951E-15	3.03434E-14
TTC17	-0.398204887	3.62087E-15	3.0426E-14
CHCHD6	0.39812456	3.67133E-15	3.0837E-14
PCDHB18	-0.398097087	3.68875E-15	3.09703E-14
EPM2AIP1	-0.398084623	3.69668E-15	3.10238E-14
PCDHGB6	-0.398021777	3.73691E-15	3.13482E-14
EGFL8	0.398015495	3.74096E-15	3.13689E-14
AK3	-0.3979897	3.75761E-15	3.14953E-14
PRKAA1	-0.397970742	3.7699E-15	3.1585E-14
WDR82	-0.397956161	3.77937E-15	3.16511E-14
TADA2B	-0.397950685	3.78294E-15	3.16676E-14
TBC1D12	-0.397748346	3.91701E-15	3.27761E-14
KDM5A	-0.397711538	3.94189E-15	3.29705E-14
IL18R1	-0.397655215	3.98027E-15	3.32775E-14
PCDHB15	-0.397624529	4.00133E-15	3.34396E-14
EDNRB	-0.397418442	4.14565E-15	3.46311E-14
AASDH	-0.397415013	4.14809E-15	3.4637E-14
NNT	-0.397391603	4.16481E-15	3.47621E-14
KDELC2	-0.397308761	4.22452E-15	3.52456E-14
STK25	0.397201962	4.30274E-15	3.58832E-14
FLT1	-0.397187815	4.3132E-15	3.59554E-14
CLPX	-0.397135389	4.35221E-15	3.62654E-14

KIAA0182	-0.396821187	4.59333E-15	3.82585E-14
PARD3B	-0.396651734	4.72875E-15	3.937E-14
AIFM2	0.39661858	4.7557E-15	3.95778E-14
NAA30	-0.396593828	4.77592E-15	3.97295E-14
LOC653501	-0.396580959	4.78646E-15	3.97988E-14
SDF2L1	0.396577385	4.7894E-15	3.97988E-14
CD82	-0.396576357	4.79024E-15	3.97988E-14
COL15A1	-0.396566536	4.79831E-15	3.98492E-14
LOC100170	-0.396553533	4.80901E-15	3.99214E-14
ZC3HC1	0.396475546	4.87371E-15	4.04416E-14
REV1	-0.396419359	4.92084E-15	4.08157E-14
PIK3CG	-0.396405661	4.9324E-15	4.08946E-14
NDUFA1	0.396390463	4.94526E-15	4.09841E-14
GNA14	-0.39633157	4.99539E-15	4.13823E-14
BTBD7	-0.396278675	5.04083E-15	4.17415E-14
ZNF710	-0.396143929	5.15845E-15	4.26976E-14
HES6	0.39613916	5.16266E-15	4.27147E-14
SERGEF	0.396126361	5.17398E-15	4.27906E-14
CMTM6	-0.396075012	5.21964E-15	4.31433E-14
AZI1	0.396073534	5.22096E-15	4.31433E-14
RPL7	0.396039398	5.25154E-15	4.3378E-14
PTK2B	-0.396028709	5.26115E-15	4.34394E-14
TACC3	0.395846781	5.4274E-15	4.47935E-14
PCDHGA4	-0.395652826	5.61032E-15	4.6284E-14
NR3C2	-0.395643043	5.61971E-15	4.63422E-14
CENPH	0.395607662	5.65377E-15	4.66039E-14
LRRK2	-0.395476482	5.78187E-15	4.764E-14
C16orf91	0.395459854	5.7983E-15	4.77557E-14
UQCRQ	0.395425282	5.83263E-15	4.80185E-14
TROAP	0.395346454	5.91164E-15	4.86489E-14
GON4L	-0.395338176	5.92E-15	4.86975E-14
MGC72080	0.39514612	6.11718E-15	5.02988E-14
LOC144438	-0.395113084	6.15175E-15	5.05621E-14
WDR34	0.395025258	6.24456E-15	5.13038E-14
PDE7B	-0.394988596	6.28371E-15	5.16042E-14
EPS8	-0.394805167	6.48323E-15	5.32207E-14
MESP1	0.394796558	6.49274E-15	5.32769E-14
USP15	-0.394793822	6.49577E-15	5.32798E-14
XYLT1	-0.394775656	6.5159E-15	5.34229E-14
SUCLA2	-0.394561044	6.75842E-15	5.53885E-14
ONECUT1	-0.394356017	6.99836E-15	5.73313E-14
FAM18B	-0.394133799	7.26785E-15	5.95145E-14
RBM16	-0.394112314	7.29444E-15	5.97076E-14
LOC641298	-0.394037935	7.38722E-15	6.04423E-14
MDK	0.394002292	7.43209E-15	6.07844E-14
FIS1	0.393964213	7.48033E-15	6.11538E-14

TMEM170A	-0.393798752	7.6935E-15	6.28707E-14
PFN1	0.39370369	7.81865E-15	6.38673E-14
GLTSCR2	0.393694155	7.83132E-15	6.39445E-14
POLR1D	0.393671558	7.86141E-15	6.41639E-14
GTF3C4	-0.393665585	7.86938E-15	6.42026E-14
RPL23AP64	-0.393646906	7.89436E-15	6.43801E-14
ZNF326	-0.393618231	7.93287E-15	6.46676E-14
EGLN2	0.393602404	7.9542E-15	6.4815E-14
COL4A4	-0.393563058	8.00747E-15	6.52224E-14
FAM160B1	-0.393540965	8.03754E-15	6.54405E-14
LOC401052	0.393517232	8.06996E-15	6.56777E-14
GTF2H3	-0.393480016	8.12106E-15	6.60665E-14
C6orf72	-0.39341273	8.21425E-15	6.67974E-14
POLE4	0.393400747	8.23095E-15	6.69059E-14
HELB	-0.393387344	8.24968E-15	6.70307E-14
LOC650368	0.393368899	8.27552E-15	6.72133E-14
CCNB2	0.393330533	8.32951E-15	6.76242E-14
EFTUD2	0.393277443	8.4048E-15	6.82077E-14
ZNF264	-0.393235749	8.4644E-15	6.86634E-14
CRYGS	0.393196029	8.52156E-15	6.90989E-14
OTUB1	0.393164203	8.56763E-15	6.94442E-14
KARS	0.393100339	8.66082E-15	7.0171E-14
C14orf93	0.393072341	8.70199E-15	7.04758E-14
LONRF1	-0.393046837	8.73965E-15	7.07521E-14
C2CD3	-0.393022727	8.77541E-15	7.10127E-14
ZNF720	-0.392825525	9.07329E-15	7.33934E-14
SF3A2	0.392818851	9.08354E-15	7.34465E-14
SPATA1	-0.392677146	9.30393E-15	7.51979E-14
BCAP31	0.392631068	9.37671E-15	7.57555E-14
NANOS1	0.392532502	9.53429E-15	7.69973E-14
ZBTB37	-0.392499376	9.58783E-15	7.73983E-14
HSCB	0.39243882	9.68646E-15	7.81628E-14
CLVS1	0.392424832	9.70938E-15	7.83161E-14
NLRP6	-0.392407862	9.73727E-15	7.85092E-14
MEX3C	-0.392311479	9.89712E-15	7.97658E-14
ATP5O	0.392264286	9.97633E-15	8.03716E-14
TAOK3	-0.39224673	1.0006E-14	8.05777E-14
USP49	-0.392197941	1.00887E-14	8.12115E-14
ARID4B	-0.3921628	1.01488E-14	8.16618E-14
PPM1A	-0.392137616	1.0192E-14	8.19766E-14
KIAA0101	0.392065254	1.03173E-14	8.29506E-14
FBXW9	0.392050834	1.03424E-14	8.31191E-14
ZNF292	-0.391969865	1.04847E-14	8.42287E-14
TRIAP1	0.391919281	1.05746E-14	8.49163E-14
EHD3	-0.391905083	1.05999E-14	8.50828E-14
DPF3	-0.391902893	1.06038E-14	8.50828E-14

ERP29	0.391819802	1.07535E-14	8.62488E-14
CAPS2	-0.391724698	1.09273E-14	8.76077E-14
EPS15	-0.39167584	1.10177E-14	8.82967E-14
ZNF484	-0.391631215	1.11009E-14	8.89276E-14
ZMAT2	0.391586474	1.11849E-14	8.95324E-14
THAP6	-0.391586222	1.11854E-14	8.95324E-14
WIPF2	-0.391567472	1.12208E-14	8.97796E-14
HCRT	0.391507137	1.13354E-14	9.06605E-14
GOLGB1	-0.391478754	1.13897E-14	9.10584E-14
TUBGCP4	-0.39145824	1.14292E-14	9.13369E-14
NDUFAB1	0.391447645	1.14496E-14	9.14634E-14
PPP1R11	0.391363364	1.16132E-14	9.27336E-14
AHNAK	-0.391246293	1.18444E-14	9.45414E-14
WNK3	-0.391192717	1.19517E-14	9.53595E-14
RIN2	-0.391186964	1.19632E-14	9.54051E-14
COASY	0.391185116	1.1967E-14	9.54051E-14
RAB30	-0.391151813	1.20342E-14	9.59029E-14
TYSND1	0.391006413	1.23322E-14	9.82382E-14
PUM2	-0.390967035	1.24141E-14	9.88513E-14
SOS1	-0.390958142	1.24327E-14	9.89597E-14
GPAA1	0.390932583	1.24862E-14	9.93462E-14
RHOG	0.390924511	1.25032E-14	9.94414E-14
BAG2	0.390792232	1.27843E-14	1.01637E-13
TLE4	-0.390768054	1.28364E-14	1.0201E-13
PRSS54	0.390655946	1.30804E-14	1.03908E-13
MVD	0.390570623	1.32692E-14	1.05366E-13
RPUSD1	0.390508705	1.34079E-14	1.06424E-13
CMTM1	-0.390380405	1.36998E-14	1.08698E-13
ZNF420	-0.390376181	1.37095E-14	1.08731E-13
NOS1AP	-0.390369142	1.37257E-14	1.08811E-13
TMEM106B	-0.39036707	1.37305E-14	1.08811E-13
THSD7A	-0.390354649	1.37591E-14	1.08995E-13
PTCH1	-0.390321483	1.38359E-14	1.0956E-13
C1orf86	0.390214238	1.40871E-14	1.11504E-13
SMCHD1	-0.390159831	1.42162E-14	1.12481E-13
CTTNBP2NL	-0.390154479	1.4229E-14	1.12538E-13
RGP1	-0.390113799	1.43264E-14	1.13263E-13
ALKBH2	0.390054802	1.44688E-14	1.14344E-13
SMC1A	-0.390051346	1.44772E-14	1.14364E-13
DMD	-0.389994749	1.46151E-14	1.15409E-13
SNUPN	0.389975468	1.46625E-14	1.15736E-13
CDKN3	0.389950509	1.47239E-14	1.16175E-13
CLPP	0.389893266	1.48658E-14	1.17231E-13
FKBP14	-0.389891822	1.48694E-14	1.17231E-13
MSRB2	0.389844636	1.49874E-14	1.18114E-13
C9orf114	0.389814683	1.50628E-14	1.18661E-13

SYF2	0.38978969	1.5126E-14	1.19112E-13
NUBP2	0.389709839	1.53296E-14	1.20668E-13
PLXNA4	-0.389697149	1.53622E-14	1.20877E-13
C19orf71	-0.389612089	1.55825E-14	1.2254E-13
SFRS9	0.389610764	1.5586E-14	1.2254E-13
RPL21	0.389567274	1.56999E-14	1.23387E-13
TIRAP	-0.389504982	1.58644E-14	1.24631E-13
MRPL54	0.389390752	1.61704E-14	1.26985E-13
PKMYT1	0.389223269	1.66297E-14	1.3054E-13
WNT3	-0.389179309	1.67523E-14	1.31451E-13
LOC100133	-0.389109178	1.69498E-14	1.32948E-13
TRPT1	0.388997349	1.72695E-14	1.35402E-13
SAMD9	-0.388934607	1.74514E-14	1.36775E-13
HACE1	-0.38886882	1.76441E-14	1.38231E-13
DSCR3	-0.388784757	1.78935E-14	1.40129E-13
FKBP9L	-0.388742924	1.80189E-14	1.41056E-13
RGL1	-0.388595836	1.84665E-14	1.44503E-13
C16orf52	-0.388562848	1.85684E-14	1.45243E-13
DENND1B	-0.388553225	1.85982E-14	1.4542E-13
ILF2	0.388510032	1.87327E-14	1.46413E-13
RASSF7	0.388506797	1.87428E-14	1.46435E-13
NFU1	0.388383227	1.91329E-14	1.49424E-13
SORBS2	-0.388333187	1.92932E-14	1.50617E-13
BAHCC1	-0.38832448	1.93212E-14	1.50776E-13
RAP2C	-0.388234506	1.9613E-14	1.52994E-13
FOXO1	-0.388165893	1.98385E-14	1.54692E-13
DCHS1	-0.388154478	1.98762E-14	1.54883E-13
ZNF428	0.388153793	1.98785E-14	1.54883E-13
C2orf86	-0.388093279	2.00799E-14	1.5639E-13
TSSC1	0.387863413	2.0863E-14	1.62427E-13
ZNF674	-0.3878563	2.08877E-14	1.62555E-13
MLLT10	-0.387762786	2.12152E-14	1.6504E-13
CLASP2	-0.387578806	2.18743E-14	1.70077E-13
DIP2C	-0.387577285	2.18798E-14	1.70077E-13
MAP3K5	-0.387564775	2.19254E-14	1.70333E-13
BCKDHB	-0.387563523	2.19299E-14	1.70333E-13
CCDC34	0.387524482	2.20727E-14	1.71375E-13
HAUS8	0.387510072	2.21256E-14	1.71719E-13
PPP1R15B	-0.387499414	2.21648E-14	1.71957E-13
SLC39A14	-0.387480538	2.22345E-14	1.7243E-13
EBNA1BP2	0.387459656	2.23118E-14	1.72962E-13
PCDHB11	-0.387385823	2.25871E-14	1.75028E-13
GPRIN3	-0.387335247	2.27777E-14	1.76436E-13
NTN3	-0.387321094	2.28313E-14	1.76783E-13
TTC8	-0.387297936	2.29193E-14	1.77395E-13
MRPL45	0.387294258	2.29333E-14	1.77434E-13

ZBTB44	-0.387282359	2.29786E-14	1.77716E-13
GSTO1	0.387277477	2.29973E-14	1.77791E-13
C1orf151	0.387250906	2.3099E-14	1.78508E-13
LOC150381	0.387222689	2.32074E-14	1.79277E-13
TTC27	0.387081658	2.37571E-14	1.83452E-13
TBC1D9B	-0.387057319	2.38533E-14	1.84123E-13
AGL	-0.387044868	2.39026E-14	1.84432E-13
SLC25A19	0.38700744	2.40515E-14	1.85509E-13
RBBP6	-0.386956334	2.42563E-14	1.87017E-13
TTC14	-0.386811411	2.48463E-14	1.91491E-13
NIPA1	-0.386719504	2.52277E-14	1.94356E-13
MANF	0.386711208	2.52624E-14	1.94548E-13
RUSC2	-0.386707441	2.52782E-14	1.94594E-13
GFM1	-0.386683895	2.53771E-14	1.9528E-13
C8orf76	0.386662735	2.54662E-14	1.9589E-13
SLC24A1	-0.386657386	2.54888E-14	1.95988E-13
TADA3	0.386654974	2.5499E-14	1.95991E-13
UQCR11	0.386631281	2.55993E-14	1.96686E-13
HGSNAT	-0.386602291	2.57225E-14	1.97557E-13
USPL1	-0.386571027	2.58561E-14	1.98506E-13
EAF1	-0.386272627	2.71657E-14	2.0848E-13
CCDC75	-0.386224056	2.73849E-14	2.10081E-13
TBC1D8	-0.386196495	2.75101E-14	2.1096E-13
LOC729020	-0.386004143	2.83993E-14	2.17695E-13
C4orf12	-0.385950813	2.86508E-14	2.19539E-13
LIN7B	0.385897715	2.89034E-14	2.21389E-13
SEC14L1	-0.385857991	2.90938E-14	2.22735E-13
GLRX3	0.385856395	2.91014E-14	2.22735E-13
CBR4	-0.385650112	3.01102E-14	2.30367E-13
TMEM47	-0.385608174	3.03195E-14	2.31879E-13
NTN4	-0.385592673	3.03972E-14	2.32384E-13
FOXJ2	-0.385562136	3.05508E-14	2.33469E-13
EPHA3	-0.385531041	3.0708E-14	2.3458E-13
VPRBP	-0.385525368	3.07368E-14	2.3471E-13
KIAA0317	-0.385508549	3.08223E-14	2.35272E-13
RABIF	0.385477058	3.09829E-14	2.36408E-13
PARP9	-0.385402436	3.13668E-14	2.39245E-13
GIGYF2	-0.385396526	3.13974E-14	2.39387E-13
PIP5K1A	-0.385354043	3.16182E-14	2.40978E-13
HDGFRP3	-0.385308629	3.1856E-14	2.42698E-13
CSE1L	0.385296572	3.19194E-14	2.43088E-13
KCTD9	-0.385264129	3.20906E-14	2.44299E-13
TPP2	-0.385221486	3.23171E-14	2.45929E-13
POU2F1	0.385195529	3.24557E-14	2.46889E-13
IL33	-0.3851745	3.25685E-14	2.47652E-13
C1orf85	0.385161115	3.26404E-14	2.48104E-13

HDX	-0.385157713	3.26587E-14	2.48149E-13
MBP	-0.385096887	3.29878E-14	2.50554E-13
SEC14L4	-0.384991285	3.3567E-14	2.54855E-13
PCSK5	-0.384969652	3.36868E-14	2.55668E-13
ZNF430	-0.384965724	3.37086E-14	2.55736E-13
MBD5	-0.384949476	3.3799E-14	2.56324E-13
MYO10	-0.384927821	3.39198E-14	2.57142E-13
DRG2	0.384910522	3.40166E-14	2.57778E-13
ANO1	-0.384882519	3.41738E-14	2.58871E-13
SRP19	0.384798525	3.46498E-14	2.62377E-13
SKA1	0.384719754	3.51021E-14	2.65701E-13
RC3H1	-0.384607399	3.57573E-14	2.70557E-13
SLC35A3	-0.38456599	3.60017E-14	2.72303E-13
PLAT	-0.384531026	3.62094E-14	2.7377E-13
YIF1B	0.384430621	3.68124E-14	2.78223E-13
PIK3R3	-0.384395473	3.70258E-14	2.7973E-13
DNAJB2	0.384371212	3.71738E-14	2.80741E-13
FBXL15	0.384250655	3.79178E-14	2.86252E-13
DAPK1	-0.3842202	3.81081E-14	2.87579E-13
OIP5	0.384168372	3.8434E-14	2.89929E-13
TIPARP	-0.384116405	3.87636E-14	2.92304E-13
C14orf37	-0.384078109	3.90082E-14	2.94038E-13
FKBP4	0.384043715	3.92292E-14	2.95592E-13
HELZ	-0.384034741	3.9287E-14	2.95916E-13
DPYSL2	-0.383886818	4.02529E-14	3.03076E-13
ARAP3	-0.383883908	4.02721E-14	3.03106E-13
EPB41L4A	-0.383861783	4.04186E-14	3.04094E-13
MTF1	-0.383780412	4.0962E-14	3.08066E-13
ZNF576	0.383770238	4.10304E-14	3.08464E-13
LYAR	0.383745817	4.11952E-14	3.09586E-13
SPOPL	-0.383698148	4.15186E-14	3.11784E-13
C16orf45	-0.383698088	4.1519E-14	3.11784E-13
TUBE1	-0.383672999	4.16902E-14	3.12952E-13
EBP	0.383640191	4.19152E-14	3.14522E-13
CTNNBL1	0.383584871	4.22972E-14	3.17269E-13
LOC100132	-0.383470671	4.30966E-14	3.23143E-13
DOCK10	-0.383388082	4.36839E-14	3.27424E-13
BCOR	-0.38337054	4.38096E-14	3.28243E-13
LMO7	-0.383343208	4.40063E-14	3.29592E-13
SETD5	-0.383330103	4.41009E-14	3.30176E-13
KALRN	-0.383318411	4.41854E-14	3.30685E-13
SLC4A4	-0.383262634	4.4591E-14	3.33595E-13
UFSP1	0.383249037	4.46904E-14	3.34213E-13
FIBP	0.383195934	4.50808E-14	3.37006E-13
C8orf33	0.383165436	4.53065E-14	3.38566E-13
MAML3	-0.383141091	4.54875E-14	3.39791E-13

TUBD1	0.383089087	4.58764E-14	3.42568E-13
CHD1	-0.383083915	4.59153E-14	3.42653E-13
GAS1	-0.383082995	4.59222E-14	3.42653E-13
CNOT10	0.383053033	4.61479E-14	3.44208E-13
TSPYL3	-0.382935134	4.7047E-14	3.50783E-13
TSPYL1	-0.382878992	4.74811E-14	3.53887E-13
SPTY2D1	-0.38266313	4.9187E-14	3.66465E-13
LOC283922	-0.382647621	4.93119E-14	3.67257E-13
SUMO1P3	0.382602407	4.96777E-14	3.69843E-13
TIE1	-0.382577869	4.98773E-14	3.71191E-13
RAN	0.382564413	4.99871E-14	3.71869E-13
RRP12	0.382534282	5.02338E-14	3.73565E-13
TATDN1	0.382488649	5.06097E-14	3.7622E-13
SP4	-0.382458262	5.08615E-14	3.77951E-13
HOOK3	-0.382319686	5.20257E-14	3.86457E-13
MAD2L2	0.382210082	5.29649E-14	3.93287E-13
PSMB2	0.382169326	5.33184E-14	3.95765E-13
HSBP1L1	0.382118915	5.37588E-14	3.98885E-13
ADAMTS2	-0.382087126	5.40383E-14	4.0081E-13
ZNF691	0.3820696	5.41931E-14	4.01808E-13
FAM149A	-0.381971085	5.50709E-14	4.08164E-13
ERLIN2	-0.3819148	5.55787E-14	4.11774E-13
RAD51	0.381881962	5.5877E-14	4.13831E-13
MND1	0.381818572	5.64574E-14	4.17974E-13
SYNGR2	0.381782177	5.67933E-14	4.20304E-13
RASAL2	-0.38170989	5.74662E-14	4.25126E-13
BCLAF1	-0.381702728	5.75333E-14	4.25465E-13
GOLGA5	-0.381677548	5.77698E-14	4.27055E-13
HEXIM2	0.381656914	5.79644E-14	4.28334E-13
TSSK1B	-0.381608125	5.84269E-14	4.31592E-13
RND3	-0.381505984	5.94068E-14	4.38668E-13
SLCO2A1	-0.381377966	6.06578E-14	4.47739E-13
GLCCI1	-0.381286663	6.15657E-14	4.54272E-13
KCNB1	-0.381280805	6.16244E-14	4.54537E-13
AIDA	0.381238091	6.20541E-14	4.57537E-13
NDUFA4	0.38113508	6.31025E-14	4.65095E-13
LOC645166	0.381116258	6.3296E-14	4.66348E-13
ZFYVE1	-0.3811104	6.33563E-14	4.6662E-13
RSL24D1	0.381068855	6.37857E-14	4.69609E-13
RPS26	0.381035227	6.41353E-14	4.72009E-13
FGD6	-0.381008401	6.44156E-14	4.73896E-13
DCBLD1	-0.380873521	6.58431E-14	4.84055E-13
CEP192	-0.380873338	6.58451E-14	4.84055E-13
STON1	-0.380797218	6.66643E-14	4.89897E-13
REPS2	-0.380743677	6.72465E-14	4.93993E-13
APP	-0.380696426	6.77645E-14	4.97614E-13

BPTF	-0.38065068	6.82696E-14	5.01139E-13
ABCA9	-0.380567883	6.91933E-14	5.07732E-13
NDUFA6	0.380544172	6.94601E-14	5.09502E-13
PLS3	-0.380474027	7.02552E-14	5.15145E-13
NFKBIL2	0.380470663	7.02936E-14	5.15236E-13
EIF5A	0.380393357	7.11807E-14	5.21546E-13
SUB1	0.380378715	7.13499E-14	5.22594E-13
RBMX2	0.380359533	7.15722E-14	5.2403E-13
FLAD1	0.380069794	7.5014E-14	5.49027E-13
DDHD2	-0.380011709	7.57232E-14	5.54014E-13
TIMM8A	0.379980993	7.61009E-14	5.56573E-13
POLR2B	-0.37983605	7.79081E-14	5.69523E-13
VPS13B	-0.379834421	7.79287E-14	5.69523E-13
KLHDC4	0.379809146	7.82482E-14	5.71648E-13
CRAMP1L	-0.379744572	7.90703E-14	5.77442E-13
TNNI3K	-0.37974055	7.91218E-14	5.77607E-13
FAM158A	0.379678341	7.99223E-14	5.83237E-13
ZZZ3	-0.379674087	7.99773E-14	5.83425E-13
HCG18	0.379647002	8.03286E-14	5.85772E-13
ZFP161	-0.379629331	8.05586E-14	5.87234E-13
ZBTB4	-0.379507815	8.21575E-14	5.98671E-13
RASGRF2	-0.379496827	8.23037E-14	5.99516E-13
LRRC58	-0.379443373	8.30181E-14	6.04499E-13
PSRC1	0.379287702	8.51334E-14	6.19675E-13
TMEM106C	0.379260901	8.55029E-14	6.22137E-13
ATG4B	0.379216104	8.61239E-14	6.26427E-13
POLG	-0.379118901	8.74868E-14	6.36108E-13
EIF4H	-0.379112015	8.75841E-14	6.36583E-13
LOC90586	-0.379086939	8.79395E-14	6.38933E-13
SYNPO	-0.378977947	8.95006E-14	6.50038E-13
TRANK1	-0.378803898	9.20499E-14	6.6831E-13
TNFAIP1	-0.378733213	9.31054E-14	6.75727E-13
C17orf53	0.3787276	9.31897E-14	6.76092E-13
HDAC3	0.378716076	9.33631E-14	6.77103E-13
DPH3B	-0.378617839	9.48537E-14	6.87664E-13
SLC8A1	-0.378456354	9.73549E-14	7.05539E-13
LIN52	-0.378434171	9.77035E-14	7.07808E-13
TLR6	-0.378358284	9.89052E-14	7.16254E-13
USP32	-0.378336416	9.92542E-14	7.18346E-13
ITGA4	-0.378335655	9.92663E-14	7.18346E-13
GRK6	0.378314467	9.96057E-14	7.2054E-13
GNPTAB	-0.378248223	1.00674E-13	7.28003E-13
GOLGA1	-0.378240929	1.00792E-13	7.28594E-13
TMEM11	0.378227191	1.01015E-13	7.29942E-13
ADAMTSL3	-0.378221585	1.01107E-13	7.30336E-13
STS	-0.378117153	1.02821E-13	7.42231E-13

KCND3	-0.378116717	1.02828E-13	7.42231E-13
LOC339674	0.378103629	1.03045E-13	7.43527E-13
MTOR	-0.377831337	1.07658E-13	7.76535E-13
CHCHD4	0.377781938	1.08517E-13	7.82364E-13
KIAA0913	-0.377780322	1.08545E-13	7.82364E-13
TAZ	0.377747123	1.09126E-13	7.86265E-13
CAMK1	0.377728148	1.09459E-13	7.88382E-13
B3GALT6	0.377686852	1.10188E-13	7.93344E-13
STARD13	-0.377597188	1.11787E-13	8.04566E-13
PRMT1	0.377553804	1.12569E-13	8.099E-13
KL	-0.377514542	1.13281E-13	8.14729E-13
SOCS5	-0.377507167	1.13415E-13	8.154E-13
ARL2	0.377409156	1.15214E-13	8.28036E-13
BCL2	-0.377385569	1.15651E-13	8.30877E-13
YES1	-0.377366339	1.16009E-13	8.33146E-13
NAF1	-0.377328848	1.16709E-13	8.37872E-13
CTDSPL2	-0.377285808	1.17518E-13	8.43376E-13
ATR	-0.377259787	1.1801E-13	8.466E-13
INHBC	-0.377081506	1.21433E-13	8.70848E-13
RALGPS1	-0.377024483	1.22549E-13	8.78434E-13
COX7A2	0.377022926	1.22579E-13	8.78434E-13
MAN1C1	-0.377012574	1.22783E-13	8.79577E-13
TRAF2	0.3770097	1.2284E-13	8.79666E-13
ACAD8	-0.376997112	1.23088E-13	8.81126E-13
SGCB	-0.376945374	1.24113E-13	8.88145E-13
IFI27L1	0.376873556	1.2555E-13	8.98105E-13
SGK1	-0.376836161	1.26304E-13	9.03178E-13
DPY19L1	-0.376787394	1.27295E-13	9.09936E-13
ARL3	0.37675161	1.28027E-13	9.14839E-13
ADPRHL2	0.376730223	1.28466E-13	9.17649E-13
PTMA	0.376715867	1.28762E-13	9.19432E-13
PIN1	0.376688926	1.29319E-13	9.23076E-13
C8orf4	-0.376659508	1.29929E-13	9.27103E-13
COL6A3	-0.376613919	1.30881E-13	9.33561E-13
ZNF432	-0.376560203	1.32012E-13	9.41287E-13
CLEC1A	-0.376510809	1.3306E-13	9.48419E-13
ZDHHC12	0.376475415	1.33816E-13	9.53465E-13
BLOC1S3	0.376445107	1.34466E-13	9.57758E-13
FMO2	-0.376270437	1.38276E-13	9.84545E-13
LRIG2	-0.376242499	1.38895E-13	9.886E-13
RNF11	-0.376185353	1.4017E-13	9.97318E-13
PLCL1	-0.376178887	1.40315E-13	9.97993E-13
UBE2J2	0.376100109	1.42093E-13	1.01028E-12
JRKL	-0.376062361	1.42953E-13	1.0159E-12
UBN2	-0.376060919	1.42986E-13	1.0159E-12
WDR18	0.375969433	1.45092E-13	1.03049E-12

RFC2	0.375920368	1.46233E-13	1.03823E-12
SLC31A1	-0.375783855	1.49456E-13	1.06073E-12
HIST2H2BA	-0.375776754	1.49626E-13	1.06156E-12
SNHG11	0.375638035	1.52976E-13	1.08494E-12
DOC2B	-0.37562755	1.53232E-13	1.08637E-12
SF3B4	0.375591031	1.54127E-13	1.09233E-12
SESN3	-0.375506835	1.56211E-13	1.10671E-12
NR1D2	-0.375475697	1.56989E-13	1.11182E-12
ARRDC4	-0.375384704	1.59284E-13	1.12748E-12
ADAMTSL1	-0.375383517	1.59314E-13	1.12748E-12
KRT18	0.375273789	1.62125E-13	1.14697E-12
COPS5	0.375236877	1.63081E-13	1.15333E-12
DSCC1	0.375203041	1.63963E-13	1.15915E-12
U2AF1L4	0.375165081	1.64958E-13	1.16571E-12
ALAD	-0.375163152	1.65008E-13	1.16571E-12
GCNT4	-0.375116435	1.66241E-13	1.174E-12
DNAJC13	-0.375078391	1.67251E-13	1.18072E-12
CAND1	-0.37502604	1.68651E-13	1.19018E-12
MARS2	-0.375010712	1.69064E-13	1.19267E-12
SH3BP5	-0.374991441	1.69583E-13	1.19591E-12
FAS	-0.374918118	1.71574E-13	1.20952E-12
PPIAL4C	0.374845778	1.73561E-13	1.2231E-12
EIF4EBP1	0.374765698	1.75787E-13	1.23834E-12
ZNF354C	-0.374748976	1.76255E-13	1.2412E-12
LRIG1	-0.374720376	1.77059E-13	1.24642E-12
C9orf16	0.374575247	1.81193E-13	1.27467E-12
EFNA4	0.374575	1.812E-13	1.27467E-12
FUT11	-0.374562154	1.8157E-13	1.27683E-12
YTHDC1	-0.374521656	1.82743E-13	1.28462E-12
SORBS1	-0.37451717	1.82874E-13	1.28508E-12
TCEA3	0.374485941	1.83784E-13	1.29058E-12
MTMR1	-0.374485876	1.83786E-13	1.29058E-12
VPS52	0.374458894	1.84575E-13	1.29567E-12
ADARB1	-0.374420904	1.85693E-13	1.30306E-12
LOC100128	-0.374396349	1.86419E-13	1.30769E-12
MYD88	-0.374374439	1.87069E-13	1.31179E-12
ISY1	0.374319431	1.88711E-13	1.32284E-12
KDM6B	-0.374294017	1.89474E-13	1.32772E-12
LOC441208	-0.374290229	1.89588E-13	1.32805E-12
SORL1	-0.37425025	1.90796E-13	1.33604E-12
NBPF1	-0.374231729	1.91358E-13	1.33951E-12
NOTCH1	-0.374208444	1.92067E-13	1.344E-12
CYYR1	-0.37416638	1.93354E-13	1.35253E-12
RECQL	-0.374035696	1.97407E-13	1.38039E-12
ZNF205	0.373971225	1.99437E-13	1.3941E-12
NOS3	-0.373961437	1.99747E-13	1.39577E-12

SDAD1	-0.373940407	2.00414E-13	1.39995E-12
GXYLT1	-0.373870048	2.02663E-13	1.41516E-12
SVIL	-0.37385867	2.0303E-13	1.41722E-12
ZNF175	-0.37381546	2.04426E-13	1.42647E-12
TSHZ2	-0.373798885	2.04964E-13	1.42972E-12
MRPS14	0.373785464	2.054E-13	1.43226E-12
PARP3	-0.37377262	2.05819E-13	1.43468E-12
EME2	-0.373757567	2.06311E-13	1.43761E-12
SLC9A7	-0.37371239	2.07794E-13	1.44743E-12
DLL1	-0.373703512	2.08086E-13	1.44896E-12
USP28	-0.373678946	2.08898E-13	1.45411E-12
EIF4A3	0.373633527	2.10407E-13	1.4641E-12
ASPSCR1	0.373548644	2.13256E-13	1.48341E-12
LOC100216	0.373539246	2.13574E-13	1.4851E-12
CD226	-0.373531591	2.13833E-13	1.48638E-12
TRAPPC2P1	0.373254764	2.23415E-13	1.55245E-12
ATP6V1A	-0.373143679	2.27377E-13	1.57942E-12
DCAF13	0.373046382	2.30903E-13	1.60336E-12
PGM2	-0.373033011	2.31392E-13	1.6062E-12
MFSD3	0.373024222	2.31714E-13	1.60787E-12
SATB2	-0.373007676	2.32321E-13	1.61152E-12
LILRA1	-0.372886763	2.36805E-13	1.64205E-12
ALPK1	-0.372861029	2.3777E-13	1.64817E-12
PDCD6	0.37284473	2.38384E-13	1.65185E-12
SYMPK	0.372815653	2.39482E-13	1.65888E-12
NUDT5	0.372792447	2.40361E-13	1.66439E-12
GEM	-0.372711912	2.43439E-13	1.68512E-12
POLD1	0.372695767	2.44061E-13	1.68884E-12
KIAA1267	-0.372643007	2.46103E-13	1.70238E-12
ZNF182	-0.372548276	2.49812E-13	1.72744E-12
PLEKHA1	-0.37241286	2.5521E-13	1.76414E-12
WISP1	-0.372352319	2.57659E-13	1.78046E-12
BTBD8	-0.372228567	2.62739E-13	1.81493E-12
FLJ35776	0.372187474	2.64447E-13	1.82609E-12
SKA3	0.37218138	2.64701E-13	1.82722E-12
UBXN11	0.372077921	2.69054E-13	1.85662E-12
CCDC111	-0.372052075	2.70152E-13	1.86355E-12
SRL	-0.372042324	2.70568E-13	1.86577E-12
ENDOD1	-0.372019837	2.71528E-13	1.87175E-12
COX16	0.37200745	2.72059E-13	1.87476E-12
RICTOR	-0.371887208	2.77262E-13	1.90995E-12
LIN54	-0.371857256	2.78573E-13	1.91832E-12
TRIM23	-0.371836031	2.79506E-13	1.92408E-12
FAM98B	-0.371808809	2.80707E-13	1.93168E-12
TRIM52	-0.371802531	2.80985E-13	1.93292E-12
GUCY1A3	-0.371764875	2.82656E-13	1.94375E-12

COX4I1	0.371739349	2.83794E-13	1.9509E-12
EEF2K	-0.371671522	2.86841E-13	1.97116E-12
INTS2	-0.37154419	2.92647E-13	2.01037E-12
NEXN	-0.371455373	2.96764E-13	2.03795E-12
MYPOP	0.371443915	2.97299E-13	2.04092E-12
WDR77	0.371314525	3.0341E-13	2.08216E-12
C2orf69	-0.371158972	3.1092E-13	2.13295E-12
FAM108B1	-0.371096383	3.13992E-13	2.15329E-12
RGPD5	-0.371073732	3.15111E-13	2.16022E-12
PDCD6IP	-0.371046491	3.16463E-13	2.16874E-12
LOC100132	-0.371039407	3.16815E-13	2.17041E-12
TMED9	0.370924004	3.22609E-13	2.20934E-12
GTF2H5	0.370919986	3.22812E-13	2.20997E-12
C10orf12	-0.370904101	3.23618E-13	2.21473E-12
FGFR4	0.370857264	3.26006E-13	2.2303E-12
PYGO1	-0.37080758	3.28558E-13	2.24699E-12
SSR4	0.370788669	3.29535E-13	2.2529E-12
PI4K2B	-0.370742765	3.31917E-13	2.2684E-12
PUS10	-0.370692363	3.34552E-13	2.28563E-12
ITCH	-0.370660293	3.3624E-13	2.29637E-12
KDM1A	0.370651042	3.36728E-13	2.29891E-12
NAB1	-0.370638454	3.37393E-13	2.30267E-12
DHX36	-0.370613199	3.38732E-13	2.31102E-12
MAP3K3	-0.370512717	3.44112E-13	2.34691E-12
ASCC3	-0.370376262	3.51551E-13	2.39683E-12
GIMAP7	-0.370329741	3.54123E-13	2.41354E-12
HSBP1	0.370300455	3.55751E-13	2.42381E-12
BAZ1B	-0.370296752	3.55958E-13	2.42438E-12
CGREF1	0.370209232	3.60871E-13	2.45701E-12
C1orf109	-0.370182555	3.62382E-13	2.46645E-12
SFRP1	-0.370168489	3.63181E-13	2.47105E-12
ZNF225	-0.37013039	3.65354E-13	2.48498E-12
TBL3	0.370059424	3.69436E-13	2.51189E-12
LMBRD1	-0.369922518	3.77437E-13	2.56541E-12
ERH	0.369898164	3.78877E-13	2.57432E-12
SNX4	-0.369841328	3.82261E-13	2.59643E-12
EIF2B3	0.369797262	3.84905E-13	2.61349E-12
UBQLN4	0.369785631	3.85605E-13	2.61736E-12
YAP1	-0.369737938	3.88492E-13	2.63605E-12
RIMKLB	-0.369690461	3.91386E-13	2.65479E-12
RBM7	-0.369660416	3.93229E-13	2.66638E-12
C7orf36	0.36958028	3.98185E-13	2.69907E-12
ARAP2	-0.369554234	3.99809E-13	2.70915E-12
GPR172A	0.369529948	4.01329E-13	2.71853E-12
MCF2L	-0.369432296	4.07498E-13	2.75874E-12
SPRN	0.369431593	4.07543E-13	2.75874E-12

S1PR3	-0.369426249	4.07883E-13	2.76011E-12
APBA1	-0.369361746	4.12013E-13	2.78711E-12
DENND5A	-0.369347883	4.12906E-13	2.7922E-12
IFI35	0.36931523	4.15016E-13	2.80552E-12
CFLAR	-0.369303789	4.15758E-13	2.80958E-12
IDI2	0.369285224	4.16965E-13	2.81678E-12
PACSIN3	0.369259364	4.18652E-13	2.82721E-12
CYFIP1	-0.36925683	4.18817E-13	2.82737E-12
EIF3A	-0.369204781	4.22234E-13	2.84947E-12
PLA2G12A	-0.369194846	4.22889E-13	2.85293E-12
STX2	-0.369167532	4.24695E-13	2.86414E-12
PLAC8L1	0.369086464	4.30101E-13	2.89962E-12
ZNF148	-0.369070904	4.31147E-13	2.90568E-12
TXNDC16	-0.369016402	4.34828E-13	2.9295E-12
DTWD2	-0.368974514	4.37678E-13	2.9477E-12
FRS2	-0.368920213	4.41399E-13	2.97176E-12
LOC728758	0.368875665	4.44476E-13	2.99146E-12
EIF4A1	0.368825396	4.47972E-13	3.01397E-12
GPR182	-0.36880522	4.49383E-13	3.02245E-12
G2E3	-0.368735036	4.54325E-13	3.05465E-12
FGFR1OP2	-0.368707752	4.56261E-13	3.06663E-12
RAB11FIP2	-0.36860301	4.63766E-13	3.11603E-12
EVI5	-0.368593858	4.64428E-13	3.11942E-12
C3orf45	0.36846726	4.73673E-13	3.18045E-12
KRR1	-0.368436717	4.75931E-13	3.19453E-12
RAI1	-0.368405961	4.78214E-13	3.20877E-12
C1RL	-0.368375517	4.80486E-13	3.22293E-12
SHROOM2	-0.368262038	4.89045E-13	3.27923E-12
MAML1	-0.368242294	4.90549E-13	3.28821E-12
AIM1L	0.368132244	4.99017E-13	3.34385E-12
ARPC5L	0.368126637	4.99452E-13	3.34564E-12
CCDC121	-0.368103887	5.01222E-13	3.35637E-12
FAM113A	0.367727417	5.31415E-13	3.55735E-12
CD63	0.36766507	5.36584E-13	3.59075E-12
CNIH4	0.367624407	5.39982E-13	3.61227E-12
SH3BP4	-0.367606529	5.41482E-13	3.62109E-12
KDELR1	0.367490273	5.51339E-13	3.68577E-12
CBX3	0.367449214	5.54862E-13	3.70808E-12
DHX34	0.367342363	5.64134E-13	3.76878E-12
SWAP70	-0.367319332	5.66152E-13	3.78099E-12
ARMC7	0.367171641	5.79263E-13	3.86726E-12
GALNT10	-0.367136635	5.82414E-13	3.88699E-12
RANGRF	0.367100604	5.85675E-13	3.90744E-12
RPS6KA5	-0.367047055	5.90554E-13	3.93867E-12
UBE3A	-0.366987461	5.9603E-13	3.97387E-12
ANTXR1	-0.366815912	6.12074E-13	4.07947E-12

ADCY9	-0.366809345	6.12696E-13	4.08225E-12
EIF4G2	-0.366665074	6.26528E-13	4.17301E-12
LOC654342	0.36663339	6.29606E-13	4.19211E-12
SPCS1	0.366607849	6.32099E-13	4.2073E-12
ARHGAP6	-0.366588429	6.34E-13	4.21855E-12
NOTCH3	-0.366281082	6.64848E-13	4.42233E-12
PKN1	0.366274343	6.65541E-13	4.42546E-12
HNRNPA3	0.366258271	6.67195E-13	4.43498E-12
ALDH6A1	-0.366122278	6.81357E-13	4.5276E-12
GLT8D2	-0.366095506	6.84179E-13	4.54484E-12
USP46	-0.366083932	6.85403E-13	4.55145E-12
DNAJC8	0.366050314	6.88969E-13	4.57361E-12
AASDHPPT	-0.366011876	6.9307E-13	4.59929E-12
CDH11	-0.365949443	6.9978E-13	4.64228E-12
ZMAT3	-0.36594659	7.00088E-13	4.64278E-12
CNTF	-0.365891457	7.06069E-13	4.68088E-12
TUBB2C	0.36587943	7.07381E-13	4.68802E-12
CRY1	-0.365861354	7.09356E-13	4.69954E-12
PCDHB4	-0.365777776	7.1856E-13	4.75894E-12
LRP5	-0.365740037	7.22754E-13	4.78512E-12
ITPRIP	-0.365720761	7.24906E-13	4.79777E-12
43891	-0.365715025	7.25547E-13	4.80042E-12
SUMO1	0.36570858	7.26269E-13	4.8036E-12
ZNRF1	0.36565766	7.31993E-13	4.83985E-12
NAV2	-0.36563945	7.34051E-13	4.85185E-12
FAM176B	0.365583245	7.40437E-13	4.89244E-12
GINS2	0.365458893	7.54762E-13	4.98543E-12
SVEP1	-0.365411283	7.60318E-13	5.02047E-12
GPRASP1	-0.365400184	7.61619E-13	5.02739E-12
KRAS	-0.365340856	7.68609E-13	5.07185E-12
POLR2D	0.365317937	7.71327E-13	5.0881E-12
RECQL5	0.365276645	7.76246E-13	5.11886E-12
RALBP1	-0.365258191	7.78455E-13	5.13172E-12
ARHGEF7	-0.365130082	7.93957E-13	5.23218E-12
CAMSAP1	-0.365114316	7.95885E-13	5.24315E-12
RPRD1B	-0.365105426	7.96974E-13	5.24859E-12
YPEL3	0.365098596	7.97812E-13	5.25238E-12
EIF1AD	0.365084403	7.99557E-13	5.26212E-12
DCTN3	0.365037232	8.0538E-13	5.29869E-12
SIRPB2	-0.36501976	8.07547E-13	5.3112E-12
C19orf55	-0.364955595	8.15556E-13	5.3621E-12
TRIM2	-0.364924678	8.19443E-13	5.38588E-12
UBE3B	-0.364890634	8.23744E-13	5.41236E-12
KIF13A	-0.364882851	8.2473E-13	5.41705E-12
TTC30B	-0.364844624	8.29591E-13	5.44718E-12
FRAT2	0.364829746	8.31491E-13	5.45786E-12

CYP1B1	-0.364750087	8.41734E-13	5.52327E-12
C9orf100	0.364742692	8.42691E-13	5.52773E-12
SCO2	0.364716427	8.46099E-13	5.54826E-12
SF3B1	-0.36460832	8.60268E-13	5.63932E-12
IPO8	-0.364530363	8.7063E-13	5.70536E-12
RNF165	-0.364458944	8.8023E-13	5.76637E-12
GIMAP8	-0.364434438	8.83547E-13	5.7862E-12
ZNF678	-0.364379651	8.91009E-13	5.83315E-12
EIF3CL	0.364303592	9.01469E-13	5.89969E-12
IRF6	-0.364296407	9.02464E-13	5.90426E-12
ACSL1	-0.364259395	9.07603E-13	5.93593E-12
PCDH18	-0.364152169	9.22654E-13	6.03239E-12
DLK2	0.364143743	9.23847E-13	6.0382E-12
MTMR3	-0.364053694	9.36691E-13	6.12014E-12
CCDC94	0.364041452	9.38451E-13	6.12963E-12
EIF3E	0.364038192	9.3892E-13	6.13068E-12
DOHH	0.363950685	9.51598E-13	6.21143E-12
MCAT	0.363872043	9.63134E-13	6.28467E-12
MAP1B	-0.363847829	9.66713E-13	6.30596E-12
SUV420H1	-0.363798667	9.74021E-13	6.35155E-12
ZADH2	-0.363775409	9.77497E-13	6.37213E-12
AKAP6	-0.363745524	9.81981E-13	6.39926E-12
DUSP16	-0.363718497	9.86054E-13	6.4237E-12
SNX29	-0.363582806	1.00675E-12	6.55639E-12
COL4A3	-0.363541865	1.01308E-12	6.59357E-12
ATP2C1	-0.363541587	1.01312E-12	6.59357E-12
IFRD2	0.36348402	1.02209E-12	6.64973E-12
PRIC285	-0.363459459	1.02593E-12	6.67063E-12
SUPV3L1	0.363458649	1.02606E-12	6.67063E-12
LASS5	0.363457113	1.0263E-12	6.67063E-12
UBAC2	0.363441409	1.02877E-12	6.68449E-12
EXD2	-0.363401994	1.03499E-12	6.72272E-12
USP42	-0.363396733	1.03582E-12	6.72594E-12
ZNF518A	-0.36337719	1.03893E-12	6.74387E-12
LOC100009	0.363205433	1.06657E-12	6.92107E-12
DDRGK1	0.363147422	1.07607E-12	6.98043E-12
MRPS25	0.36281191	1.13265E-12	7.34506E-12
FBXO30	-0.362793099	1.13591E-12	7.36379E-12
C11orf73	0.362756751	1.14222E-12	7.40234E-12
NDFIP2	-0.362728575	1.14715E-12	7.43182E-12
OSBPL5	-0.362709201	1.15054E-12	7.4514E-12
KIAA0947	-0.362516656	1.18484E-12	7.671E-12
ZAK	-0.362483002	1.19093E-12	7.70796E-12
ZNF330	-0.362449422	1.19704E-12	7.745E-12
SCN8A	-0.36234083	1.21702E-12	7.87171E-12
43896	-0.362302075	1.22423E-12	7.91576E-12

EIF2B1	0.362257881	1.2325E-12	7.96665E-12
RCBTB2	-0.362080402	1.26627E-12	8.18227E-12
KDM3B	-0.362072149	1.26786E-12	8.18991E-12
AMMECR1L	-0.362039955	1.27409E-12	8.22748E-12
HNRNPC	0.361939307	1.29376E-12	8.35178E-12
ADIPOR2	-0.361880638	1.30536E-12	8.42394E-12
RABGAP1	-0.361868778	1.30772E-12	8.43642E-12
PPP1R16B	-0.361855867	1.31029E-12	8.45027E-12
LOC399959	-0.361782537	1.32499E-12	8.54229E-12
NUP85	0.361742788	1.33302E-12	8.59131E-12
KIAA1644	-0.361723797	1.33688E-12	8.61337E-12
KIAA0415	0.361720562	1.33754E-12	8.61482E-12
TWF1	-0.361665844	1.34871E-12	8.68398E-12
CCNG2	-0.361616483	1.35887E-12	8.74656E-12
LRRC32	-0.361579238	1.36658E-12	8.79338E-12
SLC35D2	0.361512655	1.38048E-12	8.87994E-12
CDC14B	-0.36143776	1.39628E-12	8.97866E-12
NAMPT	-0.361402991	1.40367E-12	9.02329E-12
SEMA6D	-0.361249369	1.4368E-12	9.23328E-12
SFRS12	-0.361206187	1.44625E-12	9.29101E-12
ANKS3	0.361191193	1.44955E-12	9.30917E-12
IMPG1	-0.361125607	1.46405E-12	9.39927E-12
USP45	-0.361108325	1.46789E-12	9.42091E-12
SLC12A4	-0.361032096	1.48497E-12	9.52743E-12
CTU2	0.36097456	1.49798E-12	9.60785E-12
USP33	-0.360837278	1.52949E-12	9.80678E-12
TMEM208	0.360730206	1.55452E-12	9.96402E-12
PPIL4	-0.360714424	1.55824E-12	9.98467E-12
ZFHX3	-0.36060113	1.58522E-12	1.01543E-11
NPTN	-0.360508447	1.60762E-12	1.02945E-11
SLFN5	-0.360460162	1.61942E-12	1.03667E-11
NDUFB6	0.360445532	1.62301E-12	1.03863E-11
SF3A3	0.36043327	1.62603E-12	1.04023E-11
IFI27L2	0.360411367	1.63143E-12	1.04335E-11
KBTBD11	-0.360407643	1.63235E-12	1.0436E-11
B4GALT2	0.360378846	1.63948E-12	1.04782E-11
PABPN1	0.360270404	1.6666E-12	1.06482E-11
DHX16	0.360216138	1.68034E-12	1.07325E-11
ABI3BP	-0.360202532	1.6838E-12	1.07512E-11
ATP5C1	0.36014522	1.69846E-12	1.08413E-11
DBI	0.360059015	1.72074E-12	1.098E-11
THRAP3	-0.360025244	1.72955E-12	1.10327E-11
FBXW2	-0.360017618	1.73155E-12	1.10419E-11
WDYHV1	0.35987261	1.76991E-12	1.12829E-11
OSMR	-0.359799086	1.78968E-12	1.14053E-11
RAB5A	-0.35968673	1.8203E-12	1.15967E-11

HMCN1	-0.359586536	1.84805E-12	1.17697E-11
VEZT	-0.359563854	1.85438E-12	1.18063E-11
SFRS11	-0.359402165	1.90018E-12	1.2094E-11
GLI3	-0.359385511	1.90496E-12	1.21205E-11
PAN3	-0.359322219	1.92323E-12	1.22329E-11
ROR1	-0.359271759	1.93792E-12	1.23224E-11
GANC	-0.359183714	1.96381E-12	1.2483E-11
ARID2	-0.359158449	1.9713E-12	1.25259E-11
FKBP11	0.359156719	1.97182E-12	1.25259E-11
ITGB3BP	0.359125579	1.98109E-12	1.25808E-11
PDXDC1	-0.359116522	1.9838E-12	1.2594E-11
GOLGA2	-0.359085921	1.99296E-12	1.26481E-11
PCDHGC5	-0.358989478	2.02213E-12	1.28292E-11
H2AFJ	0.358974019	2.02684E-12	1.2855E-11
SIK2	-0.358941565	2.03677E-12	1.29138E-11
CALCOCO1	-0.358933411	2.03928E-12	1.29256E-11
ZNF784	0.358852205	2.06436E-12	1.30804E-11
RGPD6	-0.35875124	2.09598E-12	1.32765E-11
DHTKD1	-0.358747677	2.0971E-12	1.32794E-11
C14orf179	0.358658889	2.12531E-12	1.34537E-11
RAB21	-0.358618656	2.13821E-12	1.35311E-11
AFAP1	-0.35859526	2.14575E-12	1.35745E-11
RMND5A	-0.358542636	2.1628E-12	1.3678E-11
ADCY3	-0.358527213	2.16782E-12	1.37054E-11
TMEM18	0.358489534	2.18013E-12	1.37789E-11
CISD1	0.358439046	2.19675E-12	1.38795E-11
RB1	-0.358345261	2.22793E-12	1.40721E-11
CWF19L1	0.358313927	2.23845E-12	1.4134E-11
POLI	-0.358265404	2.25483E-12	1.42329E-11
PCDHGA7	-0.358261345	2.2562E-12	1.42371E-11
KLF9	-0.358249289	2.26029E-12	1.42584E-11
MDP1	0.358136483	2.29891E-12	1.44933E-11
MBNL1	-0.358136268	2.29899E-12	1.44933E-11
MAPK6	-0.358084842	2.31681E-12	1.4601E-11
NAALADL2	-0.358064483	2.3239E-12	1.46411E-11
SATB1	-0.358016246	2.34079E-12	1.47428E-11
ZNF526	0.357916303	2.37617E-12	1.49609E-11
OXSR1	-0.357878669	2.38962E-12	1.50409E-11
ERCC2	0.357811233	2.41392E-12	1.5189E-11
SBNO2	-0.357776953	2.42636E-12	1.52625E-11
PSME1	0.357673518	2.46429E-12	1.54962E-11
ING3	-0.357575075	2.50092E-12	1.57216E-11
TMEM149	0.357553843	2.5089E-12	1.57667E-11
MBIP	-0.357518235	2.52232E-12	1.5846E-11
CCDC22	0.357485491	2.53472E-12	1.5919E-11
ZNF713	-0.357477553	2.53774E-12	1.59329E-11

FGF7	-0.357443848	2.55059E-12	1.60085E-11
AP2M1	0.357403928	2.56589E-12	1.60994E-11
FAM58A	0.357400816	2.56708E-12	1.61019E-11
TEX264	0.357392079	2.57044E-12	1.61179E-11
GTF2A2	0.357375316	2.5769E-12	1.61533E-11
TRMT2A	0.35733263	2.59343E-12	1.62518E-11
SIL1	0.357245095	2.62764E-12	1.6461E-11
ZNF358	0.357236508	2.63102E-12	1.6477E-11
MTIF3	0.357193104	2.64817E-12	1.65792E-11
RPL19P12	0.35712811	2.67406E-12	1.6736E-11
ETS2	-0.357000967	2.72541E-12	1.7052E-11
PTPN4	-0.356890448	2.77083E-12	1.73307E-11
ATP8B4	-0.356886821	2.77233E-12	1.73347E-11
MFN1	-0.356881448	2.77456E-12	1.73432E-11
FRK	-0.3568656	2.78114E-12	1.73788E-11
PCDHAC2	-0.356784499	2.81506E-12	1.75853E-11
ATF4	0.356726816	2.83943E-12	1.7732E-11
KAT2A	0.356686784	2.85647E-12	1.78328E-11
RSPO3	-0.356576877	2.90375E-12	1.81223E-11
C5orf51	-0.356511679	2.93216E-12	1.82939E-11
ANUBL1	-0.356477499	2.94716E-12	1.83817E-11
MYCT1	-0.356471077	2.94999E-12	1.83936E-11
CREB3L2	-0.356451183	2.95876E-12	1.84425E-11
LIMA1	-0.356248881	3.04945E-12	1.90018E-11
ATAD3B	0.35619562	3.07377E-12	1.91474E-11
LRRC69	0.356163193	3.08868E-12	1.92342E-11
NPR3	-0.356114269	3.11129E-12	1.9369E-11
CREBL2	-0.356105733	3.11526E-12	1.93858E-11
ENTPD7	-0.356104259	3.11594E-12	1.93858E-11
AFTPH	-0.356055416	3.13871E-12	1.95214E-11
ZNF704	-0.356042222	3.14489E-12	1.95537E-11
MEPCE	0.355965639	3.181E-12	1.9772E-11
ZFYVE16	-0.355871096	3.22613E-12	2.00463E-11
HDAC11	0.355845098	3.23864E-12	2.01178E-11
ACBD6	0.35577924	3.27057E-12	2.03098E-11
ARID1A	-0.355758933	3.28048E-12	2.03649E-11
NCAPG	0.355540024	3.38914E-12	2.1033E-11
EIF5B	0.355508013	3.40532E-12	2.11268E-11
ANKHD1-EI	-0.355465347	3.42701E-12	2.12547E-11
ELK1	-0.355460805	3.42933E-12	2.12625E-11
NFIX	-0.355442231	3.43881E-12	2.13147E-11
GNAO1	-0.355387086	3.46714E-12	2.14835E-11
MECOM	-0.355380301	3.47064E-12	2.14985E-11
CENPA	0.355276891	3.52442E-12	2.18249E-11
FAM196B	-0.355239462	3.54408E-12	2.19398E-11
CLTB	0.355057282	3.64134E-12	2.25349E-11

WDR60	-0.355022	3.66047E-12	2.26463E-11
PPP3R1	-0.35489211	3.73177E-12	2.30802E-11
EML1	-0.354856429	3.75159E-12	2.31955E-11
KLHL26	-0.354841207	3.76007E-12	2.32408E-11
CRIPT	0.354813043	3.77583E-12	2.33309E-11
STAG3L3	-0.354726416	3.82468E-12	2.36255E-11
PARP11	-0.354703978	3.83744E-12	2.36969E-11
RGPD1	-0.354694568	3.8428E-12	2.37227E-11
MED23	-0.354634485	3.8772E-12	2.39276E-11
MED30	0.354630787	3.87933E-12	2.39333E-11
CSGALNACT	-0.354546029	3.9284E-12	2.42286E-11
NPBWR1	-0.354520318	3.94341E-12	2.43136E-11
TMEM81	0.354466076	3.97525E-12	2.45023E-11
C5orf23	-0.354425906	3.99899E-12	2.46411E-11
MPDU1	0.354381609	4.02533E-12	2.47957E-11
FAM104B	0.354343988	4.04784E-12	2.49266E-11
P2RX4	0.354325086	4.05919E-12	2.49888E-11
NCOA4	-0.35428054	4.08607E-12	2.51465E-11
AFP	0.35427483	4.08953E-12	2.516E-11
SLC2A13	-0.354226242	4.11907E-12	2.53339E-11
C5orf45	0.354203347	4.13306E-12	2.54118E-11
B9D1	0.354201363	4.13427E-12	2.54118E-11
TLL1	-0.354180796	4.14689E-12	2.54814E-11
THUMPD1	-0.354151044	4.1652E-12	2.5586E-11
GAB1	-0.354104772	4.19383E-12	2.5754E-11
SNX6	-0.354073713	4.21316E-12	2.58647E-11
MTUS1	-0.354043555	4.23202E-12	2.59724E-11
PPIP5K1	-0.354015059	4.2499E-12	2.60742E-11
NFASC	-0.354009636	4.25332E-12	2.60871E-11
NPC2	0.353988048	4.26693E-12	2.61625E-11
SERF1A	0.353879448	4.33605E-12	2.65781E-11
FLI1	-0.353866008	4.34468E-12	2.66229E-11
STIP1	0.353770982	4.40618E-12	2.69914E-11
LUC7L2	-0.35374634	4.42226E-12	2.70816E-11
CCDC126	-0.353740049	4.42638E-12	2.70985E-11
PDS5B	-0.353628469	4.5E-12	2.75407E-11
LOC728554	0.353533703	4.56347E-12	2.79206E-11
TMEM222	0.353523155	4.57058E-12	2.79555E-11
CDYL2	-0.35348466	4.59665E-12	2.81063E-11
C8orf44	0.35347864	4.60074E-12	2.81227E-11
NSD1	-0.353322336	4.70819E-12	2.87706E-11
KCTD12	-0.353264342	4.74867E-12	2.90091E-11
MSRB3	-0.353183114	4.80595E-12	2.935E-11
LMLN	-0.353146015	4.83233E-12	2.95021E-11
DYNC1LI2	-0.35310352	4.86272E-12	2.96786E-11
SLC30A9	-0.353081251	4.87873E-12	2.97671E-11

UBE2L3	0.353071506	4.88574E-12	2.98008E-11
FBXL5	-0.353031259	4.91484E-12	2.99691E-11
EXOSC2	0.353021201	4.92213E-12	3.00044E-11
CXXC5	-0.352966079	4.96231E-12	3.024E-11
INS-IGF2	-0.352931767	4.98748E-12	3.03841E-11
IKZF2	-0.352925967	4.99175E-12	3.04008E-11
SMEK1	-0.352861495	5.03942E-12	3.06818E-11
SRP54	-0.352754156	5.11978E-12	3.11615E-11
LOC93622	0.352698714	5.16177E-12	3.14029E-11
LEPRE1	0.352697643	5.16259E-12	3.14029E-11
TRIM4	-0.352695304	5.16437E-12	3.14041E-11
LAMA2	-0.35255076	5.27549E-12	3.207E-11
PNN	-0.352504057	5.31189E-12	3.22815E-11
ABO	-0.352499831	5.3152E-12	3.22917E-11
METTL2A	0.35248857	5.32402E-12	3.23354E-11
TMEM161B	-0.352474969	5.33469E-12	3.23904E-11
RALGAPA1	-0.352143139	5.60159E-12	3.40005E-11
DIP2B	-0.352126776	5.61509E-12	3.40721E-11
FAM125A	0.352124282	5.61715E-12	3.40742E-11
ZNF770	-0.35210098	5.63643E-12	3.41807E-11
MCTS1	0.352086026	5.64883E-12	3.42455E-11
THAP4	0.352058411	5.67181E-12	3.43743E-11
WDR36	-0.352055647	5.67411E-12	3.43778E-11
TBC1D22A	0.351952877	5.76046E-12	3.48904E-11
FAF1	0.351950677	5.76233E-12	3.48911E-11
DUSP28	0.351909463	5.79733E-12	3.50923E-11
SNX11	0.351884649	5.8185E-12	3.52098E-11
LOC647288	-0.351866838	5.83374E-12	3.52913E-11
CDK7	0.351764002	5.92252E-12	3.58175E-11
MAMSTR	0.351735395	5.94745E-12	3.59573E-11
PUS7L	-0.351728222	5.95372E-12	3.59843E-11
KAL1	-0.351707982	5.97144E-12	3.60804E-11
B4GALT3	0.351506012	6.15109E-12	3.71546E-11
C1orf56	0.351495369	6.1607E-12	3.72014E-11
GUK1	0.351358336	6.28577E-12	3.79452E-11
DLG4	-0.351340789	6.30196E-12	3.80314E-11
PGR	-0.351336881	6.30557E-12	3.80417E-11
SETD1B	-0.351289737	6.34931E-12	3.82939E-11
PARK7	0.351256088	6.3807E-12	3.84716E-11
ATP8B1	-0.351253984	6.38267E-12	3.84718E-11
DUSP13	0.351244589	6.39147E-12	3.85132E-11
VPS37A	-0.351240639	6.39517E-12	3.85197E-11
FBLN2	-0.351239315	6.39641E-12	3.85197E-11
PION	-0.351194906	6.43818E-12	3.87595E-11
LRRC3	-0.351153436	6.47742E-12	3.89839E-11
MDM1	-0.351112187	6.51669E-12	3.92084E-11

ZMYM6	-0.351092112	6.53588E-12	3.9312E-11
PABPC4L	-0.351066128	6.5608E-12	3.945E-11
TMEM131	-0.351048716	6.57756E-12	3.95388E-11
FAM135A	-0.350915829	6.70681E-12	4.03036E-11
SNIP1	-0.350785156	6.83632E-12	4.10695E-11
THAP11	0.350779837	6.84165E-12	4.10891E-11
NOC2L	0.350763154	6.85837E-12	4.11771E-11
FAM199X	-0.350706766	6.91518E-12	4.15057E-11
RBM4	0.35053297	7.0932E-12	4.25614E-11
DPH5	0.350381852	7.25163E-12	4.34988E-11
PTPRJ	-0.350326862	7.31012E-12	4.38365E-11
KPNA1	-0.350282778	7.35735E-12	4.41065E-11
GPR89C	0.350247709	7.39514E-12	4.43196E-11
NEDD4	-0.350207182	7.43904E-12	4.45693E-11
EFCAB6	-0.350150412	7.50096E-12	4.49268E-11
RAB12	-0.350136338	7.51639E-12	4.50057E-11
SELK	0.350128271	7.52525E-12	4.50452E-11
PDF	0.350124954	7.5289E-12	4.50535E-11
RFT1	0.350113029	7.54201E-12	4.51184E-11
OPHN1	-0.35002367	7.64103E-12	4.5697E-11
RAE1	0.350009878	7.65643E-12	4.57754E-11
CTCF	-0.35000666	7.66002E-12	4.57831E-11
PCDHB8	-0.349996878	7.67097E-12	4.58348E-11
KDM2A	-0.349983058	7.68645E-12	4.59135E-11
PREX1	-0.349980622	7.68918E-12	4.59161E-11
FOXJ3	-0.349961916	7.7102E-12	4.60278E-11
ANKRD26	-0.349920934	7.75644E-12	4.62899E-11
FARSA	0.349881098	7.80165E-12	4.65458E-11
CXorf26	0.349697302	8.01357E-12	4.77958E-11
KCNIP4	-0.349523954	8.21858E-12	4.90039E-11
LOC100125	0.34943881	8.32115E-12	4.96006E-11
COX19	0.34939691	8.37208E-12	4.98893E-11
UPRT	-0.34937452	8.39942E-12	5.00372E-11
ADAMTS4	-0.349359043	8.41837E-12	5.01351E-11
APTX	0.349343456	8.43749E-12	5.0234E-11
MID2	-0.349330219	8.45377E-12	5.03158E-11
EFNA3	0.349277961	8.51832E-12	5.06849E-11
PSTK	0.349261232	8.53909E-12	5.07885E-11
STYXL1	0.349259831	8.54083E-12	5.07885E-11
ZBTB7A	-0.34910258	8.73851E-12	5.19485E-11
PLEKHA5	-0.349093366	8.75023E-12	5.20026E-11
NDUFB5	0.349059798	8.79306E-12	5.22416E-11
LGR4	-0.349050944	8.80439E-12	5.22933E-11
LTBP4	-0.349018004	8.84667E-12	5.25287E-11
C16orf61	0.348974996	8.90217E-12	5.28425E-11
FOXC2	-0.348855153	9.05862E-12	5.37552E-11

PTPRE	-0.348842535	9.07524E-12	5.38378E-11
HRCT1	0.348750641	9.19724E-12	5.45453E-11
SLC25A3	0.348719043	9.23956E-12	5.47799E-11
ANGPTL1	-0.3486006	9.39988E-12	5.57138E-11
CDC123	0.348576818	9.43239E-12	5.58899E-11
ALS2	-0.348499408	9.53899E-12	5.65048E-11
CCDC52	-0.34842038	9.64903E-12	5.71396E-11
SUN1	-0.348414631	9.65709E-12	5.71703E-11
LOC100129	-0.348378061	9.70847E-12	5.74574E-11
KAZ	-0.348355663	9.74007E-12	5.76273E-11
GPN2	0.348280527	9.84681E-12	5.82415E-11
TOP1P1	-0.348268007	9.86471E-12	5.83301E-11
NAPEPLD	-0.348193554	9.97179E-12	5.89458E-11
MAGI2	-0.34816925	1.0007E-11	5.91363E-11
TXN2	0.348077111	1.01416E-11	5.99137E-11
ARHGAP24	-0.348074639	1.01452E-11	5.99174E-11
APAF1	-0.348000772	1.02544E-11	6.05443E-11
C6orf155	-0.34792347	1.03699E-11	6.1208E-11
CRNKL1	0.347899341	1.04062E-11	6.14041E-11
GPX1	0.347894368	1.04137E-11	6.14302E-11
RWDD1	0.347882997	1.04308E-11	6.15132E-11
BNC2	-0.347867218	1.04547E-11	6.16357E-11
PCDHGB1	-0.347794758	1.0565E-11	6.22674E-11
TAF5L	-0.347776967	1.05922E-11	6.24096E-11
EMILIN3	0.347763342	1.06131E-11	6.25143E-11
LOC388588	0.347709955	1.06955E-11	6.29807E-11
TMUB1	0.347615606	1.08425E-11	6.38277E-11
NIN	-0.347561239	1.09282E-11	6.43128E-11
FSTL1	-0.347518844	1.09954E-11	6.46893E-11
SUN2	-0.347447224	1.11099E-11	6.53436E-11
KIAA1161	-0.347412819	1.11653E-11	6.56501E-11
PTRH1	0.347408996	1.11715E-11	6.5667E-11
XCR1	-0.347369713	1.12351E-11	6.60047E-11
STAG3L1	-0.347369433	1.12356E-11	6.60047E-11
TMEM233	-0.347361844	1.12479E-11	6.60577E-11
ZNF333	-0.347330265	1.12994E-11	6.63404E-11
RANBP10	-0.347325895	1.13065E-11	6.63628E-11
MBTPS1	-0.34729974	1.13493E-11	6.65945E-11
FNIP1	-0.347294265	1.13583E-11	6.66276E-11
TRAPPC4	0.347265295	1.1406E-11	6.68874E-11
ZNF579	0.347202924	1.15092E-11	6.74732E-11
AGK	-0.347171018	1.15624E-11	6.7765E-11
TMEM26	-0.34716531	1.15719E-11	6.78009E-11
RPUSD2	0.347127689	1.1635E-11	6.81504E-11
AIMP1	0.347125281	1.16391E-11	6.8154E-11
RASSF1	0.347098695	1.16838E-11	6.83962E-11

TENC1	-0.347057129	1.17542E-11	6.87878E-11
CDC34	0.347040412	1.17826E-11	6.89338E-11
USP24	-0.346928057	1.19753E-11	7.00405E-11
SDHD	-0.34680486	1.21901E-11	7.1276E-11
MRPS18C	0.346799244	1.22E-11	7.13129E-11
PCDHGC4	-0.346738311	1.23077E-11	7.19214E-11
SLC31A2	-0.346733847	1.23156E-11	7.19466E-11
NCOA6	-0.346705033	1.23669E-11	7.22251E-11
WWTR1	-0.346666239	1.24363E-11	7.2609E-11
CDC42BPA	-0.346570262	1.26096E-11	7.35993E-11
TMEM154	-0.346513681	1.27129E-11	7.41803E-11
SFRS14	-0.346505677	1.27276E-11	7.42442E-11
WDR53	0.346480307	1.27742E-11	7.44943E-11
CTSO	-0.346451692	1.2827E-11	7.47803E-11
FAR2	-0.346447613	1.28345E-11	7.48023E-11
MRPL41	0.346364352	1.29894E-11	7.5683E-11
CCDC86	0.346351697	1.30131E-11	7.57989E-11
MBTPS2	-0.34629688	1.31163E-11	7.63774E-11
TM7SF3	-0.346291845	1.31258E-11	7.64105E-11
KLRA1	-0.346207371	1.32864E-11	7.7323E-11
C11orf68	0.346129572	1.34361E-11	7.81711E-11
ALDH9A1	-0.346071445	1.3549E-11	7.87931E-11
QTRT1	0.34607045	1.35509E-11	7.87931E-11
TEX2	-0.34601857	1.36524E-11	7.93603E-11
GOLGA6L10	-0.345901821	1.38837E-11	8.06809E-11
PER3	-0.345892314	1.39027E-11	8.07677E-11
IQGAP1	-0.345828958	1.40299E-11	8.14831E-11
AARS2	0.345825744	1.40364E-11	8.1497E-11
RQCD1	-0.345763114	1.41634E-11	8.22102E-11
KLF11	-0.345731964	1.42269E-11	8.25551E-11
SMYD3	0.345727518	1.4236E-11	8.25838E-11
KCTD20	-0.345719457	1.42525E-11	8.26555E-11
TSPAN18	-0.345702694	1.42869E-11	8.28307E-11
PODXL	-0.345699989	1.42925E-11	8.28388E-11
CCDC77	0.345651721	1.43919E-11	8.33912E-11
ZNF280D	-0.345556726	1.45897E-11	8.45125E-11
ZNF440	-0.345537346	1.46304E-11	8.47235E-11
RAI2	-0.34549763	1.47141E-11	8.51834E-11
IGBP1	0.345445678	1.48243E-11	8.57963E-11
FBXW4	0.345369045	1.49883E-11	8.67203E-11
AMOTL1	-0.3453367	1.5058E-11	8.70986E-11
THYN1	0.345182759	1.53943E-11	8.90181E-11
C22orf30	-0.345099909	1.55784E-11	9.00561E-11
ATMIN	-0.345057536	1.56733E-11	9.05788E-11
MYO6	-0.345025309	1.57459E-11	9.09719E-11
IER3IP1	0.345020549	1.57567E-11	9.10076E-11

ALKBH7	0.34475336	1.63717E-11	9.45324E-11
CXorf23	-0.344693064	1.65137E-11	9.53247E-11
PHF6	-0.344677217	1.65512E-11	9.55135E-11
CDC45	0.344670596	1.65669E-11	9.55764E-11
PTPN1	-0.344666179	1.65774E-11	9.56092E-11
LPAR4	-0.344633776	1.66545E-11	9.6026E-11
KATNAL1	-0.344622039	1.66825E-11	9.61597E-11
PDS5A	-0.344617035	1.66944E-11	9.62008E-11
CCDC134	0.344529447	1.69051E-11	9.73863E-11
RBMS2	-0.344525447	1.69148E-11	9.74139E-11
APOBEC2	0.344509268	1.6954E-11	9.76115E-11
ARIH1	-0.344501297	1.69733E-11	9.76947E-11
EDC3	0.344496403	1.69852E-11	9.77349E-11
RCAN1	-0.344471427	1.7046E-11	9.80565E-11
BMS1P4	-0.344404668	1.72096E-11	9.8969E-11
RNF220	0.344376431	1.72793E-11	9.93409E-11
PTPRN2	-0.344296077	1.7479E-11	1.0046E-10
CRLF3	-0.3442638	1.75599E-11	1.00896E-10
PARP4	-0.344177528	1.77778E-11	1.02119E-10
MKI67IP	0.344164787	1.78102E-11	1.02275E-10
TTC33	-0.34414552	1.78593E-11	1.02528E-10
FAM21B	0.344029702	1.81574E-11	1.04209E-10
TMEM120A	0.344025242	1.81689E-11	1.04245E-10
PBX3	-0.344021182	1.81795E-11	1.04275E-10
SUGT1L1	-0.343989249	1.82626E-11	1.04722E-10
SFRS15	-0.343972197	1.83071E-11	1.04947E-10
PDK4	-0.343858848	1.86059E-11	1.06629E-10
ZBTB38	-0.343832454	1.86761E-11	1.07001E-10
MSTO1	0.343767833	1.88492E-11	1.07962E-10
NMD3	-0.343745278	1.891E-11	1.08279E-10
POLDIP2	0.343679369	1.90887E-11	1.0927E-10
TAB3	-0.343670715	1.91123E-11	1.09346E-10
CRTC3	-0.343670486	1.91129E-11	1.09346E-10
SSPO	-0.343660438	1.91403E-11	1.09472E-10
ING5	0.343642538	1.91893E-11	1.0972E-10
PTPN13	-0.343633202	1.92149E-11	1.09835E-10
CNTN3	-0.343626541	1.92331E-11	1.09908E-10
ZNF587	-0.343484596	1.96265E-11	1.12123E-10
GPM6A	-0.343453588	1.97134E-11	1.12588E-10
PALM2	-0.343395187	1.98783E-11	1.13496E-10
GRAMD1C	-0.343347987	2.00124E-11	1.1423E-10
LAMA5	-0.343234936	2.03374E-11	1.16052E-10
ZNF319	-0.343167684	2.05332E-11	1.17135E-10
C19orf61	0.343122291	2.06664E-11	1.17861E-10
RFX7	-0.343112643	2.06948E-11	1.17989E-10
TTPA	-0.34309737	2.07398E-11	1.18212E-10

BRD4	-0.343013229	2.09898E-11	1.19603E-10
FHL1	-0.343007563	2.10067E-11	1.19665E-10
METTL12	0.342977663	2.10963E-11	1.20141E-10
ELFN1	-0.342935047	2.12247E-11	1.20837E-10
NRP2	-0.342889855	2.13616E-11	1.21582E-10
DMTF1	-0.342792933	2.16582E-11	1.23235E-10
CLN6	0.342769303	2.17311E-11	1.23615E-10
CLMN	-0.342764815	2.1745E-11	1.23658E-10
RNASEH2B	0.342753552	2.17798E-11	1.23821E-10
CSNK1G3	-0.342680887	2.20061E-11	1.25072E-10
SLC38A6	0.342618597	2.22018E-11	1.26148E-10
TMEM127	-0.342519706	2.25161E-11	1.27897E-10
TXNRD2	0.342460485	2.27063E-11	1.28941E-10
WHSC1L1	-0.342356175	2.30453E-11	1.30829E-10
SEMA5A	-0.34228703	2.32727E-11	1.32082E-10
CAMTA2	-0.342227326	2.34708E-11	1.33168E-10
FBXL6	0.342196447	2.35739E-11	1.33715E-10
LDHAL6B	-0.342183631	2.36168E-11	1.3392E-10
ZRANB3	-0.342171902	2.36561E-11	1.34105E-10
GNG12	-0.34214201	2.37567E-11	1.34637E-10
RNF19A	-0.341974795	2.4327E-11	1.3783E-10
COG3	-0.341954404	2.43975E-11	1.3819E-10
CD46	-0.341915562	2.45322E-11	1.38914E-10
NEURL1B	-0.341898331	2.45922E-11	1.39214E-10
NHLRC1	0.341879413	2.46583E-11	1.39548E-10
AMOTL2	-0.341817486	2.48757E-11	1.40738E-10
DSCR9	0.341776457	2.50208E-11	1.41519E-10
PLA2R1	-0.341748302	2.51208E-11	1.42045E-10
PCDHGA2	-0.341595065	2.56721E-11	1.45121E-10
PAPPA	-0.341590316	2.56894E-11	1.45177E-10
CDKAL1	0.341563889	2.57857E-11	1.4568E-10
SLC30A7	-0.341545606	2.58526E-11	1.46016E-10
TMEM186	0.341483578	2.60806E-11	1.47263E-10
ITGA10	-0.341470149	2.61303E-11	1.47501E-10
CDK12	-0.341451233	2.62003E-11	1.47855E-10
FASTK	0.341428755	2.62838E-11	1.48284E-10
NCRNA0011	0.34140555	2.63703E-11	1.48713E-10
FBXO28	-0.341404356	2.63748E-11	1.48713E-10
JDP2	-0.341397824	2.63992E-11	1.48808E-10
MYO1E	-0.341383613	2.64523E-11	1.49066E-10
ZNF561	-0.341377983	2.64734E-11	1.49142E-10
SURF1	0.341334032	2.66386E-11	1.5003E-10
KDM4C	-0.341324571	2.66742E-11	1.50189E-10
PCBP4	0.341291521	2.67993E-11	1.5085E-10
DIRAS3	-0.341266768	2.68933E-11	1.51336E-10
PHKG1	-0.341241564	2.69893E-11	1.51834E-10

AHSA1	0.341163426	2.72893E-11	1.53478E-10
NUP98	-0.340945669	2.81423E-11	1.58231E-10
SIN3B	-0.340891344	2.83592E-11	1.59405E-10
ZNF296	0.340836839	2.85784E-11	1.60592E-10
ARHGEF9	-0.340792365	2.87585E-11	1.61558E-10
ISOC2	0.34078515	2.87878E-11	1.61678E-10
PNMA2	-0.340732696	2.90018E-11	1.62834E-10
HFE	-0.340727708	2.90222E-11	1.62902E-10
COBLL1	-0.340679533	2.92203E-11	1.63968E-10
SPAG9	-0.340619231	2.94701E-11	1.65323E-10
APOM	0.340578684	2.96392E-11	1.66225E-10
PRICKLE1	-0.340557374	2.97285E-11	1.66679E-10
TH1L	0.340499776	2.99711E-11	1.67991E-10
C9orf7	0.340467969	3.01058E-11	1.68699E-10
GNG5	0.340464259	3.01216E-11	1.6874E-10
GMDS	0.34040121	3.03907E-11	1.702E-10
ATP7A	-0.340363536	3.05526E-11	1.71058E-10
STARD8	-0.340354299	3.05924E-11	1.71233E-10
HTR7P1	-0.340278349	3.09217E-11	1.73028E-10
NRP1	-0.340242722	3.10774E-11	1.7385E-10
BICD1	-0.340231844	3.11251E-11	1.74068E-10
WDFY1	-0.340206043	3.12385E-11	1.74653E-10
FKTN	-0.340171881	3.13892E-11	1.75447E-10
CNNM4	-0.340143304	3.15159E-11	1.76105E-10
OXR1	-0.340120246	3.16184E-11	1.76629E-10
SNX8	0.340115503	3.16396E-11	1.76697E-10
DUS2L	0.340090916	3.17493E-11	1.7726E-10
ZRANB1	-0.340082294	3.17879E-11	1.77426E-10
LOC285593	-0.340014243	3.20941E-11	1.79085E-10
RNPS1	0.339999385	3.21613E-11	1.7941E-10
PIPSL	0.33998864	3.221E-11	1.79631E-10
ABHD13	-0.339957967	3.23494E-11	1.80358E-10
FKBP15	-0.339935931	3.245E-11	1.80868E-10
CDC25A	0.339891932	3.26516E-11	1.81941E-10
DDIT3	0.339840942	3.28868E-11	1.83178E-10
LOC728640	-0.339839811	3.2892E-11	1.83178E-10
TMEM100	-0.339835557	3.29117E-11	1.83236E-10
RNF103	-0.339831697	3.29296E-11	1.83285E-10
VAMP5	0.339785886	3.31426E-11	1.84419E-10
CBLN3	-0.339782744	3.31572E-11	1.84449E-10
ZNF616	-0.339670804	3.36836E-11	1.87324E-10
NR2F6	0.339620133	3.39245E-11	1.88611E-10
YBX1	0.339583521	3.40996E-11	1.89532E-10
NUPR1	0.339503873	3.44836E-11	1.91613E-10
PL-5283	0.339431528	3.4836E-11	1.93517E-10
APBB2	-0.339412567	3.4929E-11	1.9398E-10

PPP2R3B	0.33940295	3.49762E-11	1.94188E-10
ALG14	0.339361648	3.51798E-11	1.95264E-10
C2orf7	0.339350271	3.52361E-11	1.95522E-10
STEAP4	-0.339338472	3.52946E-11	1.95791E-10
CCDC144B	-0.33925637	3.5704E-11	1.98007E-10
TNFSF13	-0.33924423	3.57649E-11	1.9829E-10
BVES	-0.339239516	3.57886E-11	1.98366E-10
BMI1	-0.339185974	3.60587E-11	1.99808E-10
PCYT2	0.339169376	3.61428E-11	2.00218E-10
RBM8A	0.339107942	3.64559E-11	2.01896E-10
SLC33A1	-0.338994558	3.70407E-11	2.05078E-10
ZNF180	-0.338960406	3.72186E-11	2.06006E-10
PEF1	0.338914233	3.74605E-11	2.07287E-10
MED12	-0.338911707	3.74738E-11	2.07303E-10
SRF	0.338902994	3.75196E-11	2.07499E-10
RNF187	0.338891673	3.75792E-11	2.07771E-10
RAB8B	-0.338862617	3.77327E-11	2.08561E-10
MUTYH	0.338837918	3.78636E-11	2.09227E-10
CDK5	0.338831397	3.78982E-11	2.0936E-10
SHROOM3	-0.338813959	3.7991E-11	2.09815E-10
ZWINT	0.338809385	3.80154E-11	2.09891E-10
GPR124	-0.338779873	3.81731E-11	2.10668E-10
ATP6V0C	0.338779097	3.81772E-11	2.10668E-10
INO80B	0.338775047	3.81989E-11	2.10729E-10
SAMD9L	-0.33871601	3.85164E-11	2.12421E-10
NDC80	0.338685462	3.86816E-11	2.13274E-10
SUGT1	0.338659416	3.88231E-11	2.13995E-10
SEC22B	-0.338617634	3.90511E-11	2.15192E-10
CYB5D1	-0.338470679	3.98634E-11	2.19607E-10
SCN7A	-0.338455657	3.99474E-11	2.20009E-10
TRIM14	-0.338453667	3.99585E-11	2.20009E-10
ATPIF1	0.338412021	4.01922E-11	2.21235E-10
TMEM161A	0.338404296	4.02357E-11	2.21413E-10
CSF1	-0.3383868	4.03344E-11	2.21895E-10
C9orf64	-0.338376716	4.03914E-11	2.22147E-10
SGPP1	-0.338336105	4.06217E-11	2.23352E-10
MPP5	-0.338218444	4.12962E-11	2.26955E-10
ISPD	-0.338217824	4.12998E-11	2.26955E-10
TBC1D24	-0.338089375	4.20486E-11	2.31006E-10
VPS41	-0.338053975	4.22573E-11	2.32089E-10
PHF8	-0.338007408	4.25334E-11	2.33541E-10
ABTB2	-0.337847552	4.34945E-11	2.38752E-10
LOC100126	-0.337829379	4.36051E-11	2.39293E-10
PPP1R3E	-0.337807937	4.37359E-11	2.39945E-10
SCAPER	-0.33778677	4.38655E-11	2.4059E-10
NBPF15	-0.337784705	4.38781E-11	2.40593E-10

BAT1	0.337725519	4.42424E-11	2.42524E-10
PHACTR2	-0.337723269	4.42563E-11	2.42533E-10
SPTB	-0.337720463	4.42737E-11	2.42561E-10
FLJ11235	-0.337703727	4.43773E-11	2.43062E-10
PLEKHM1P	-0.337692884	4.44446E-11	2.43364E-10
ECM2	-0.337674713	4.45575E-11	2.43915E-10
ST6GAL2	-0.337655779	4.46755E-11	2.44494E-10
C6orf130	0.337647624	4.47264E-11	2.44705E-10
KRCC1	-0.337639082	4.47798E-11	2.4493E-10
LARGE	-0.337508668	4.56025E-11	2.49361E-10
RNF217	-0.337376795	4.64495E-11	2.53923E-10
PPP1CC	0.337291577	4.70049E-11	2.56889E-10
LRRC14	0.337233372	4.7388E-11	2.58911E-10
TMEM200C	-0.337150323	4.79398E-11	2.61855E-10
KPNA3	-0.337145223	4.79739E-11	2.61969E-10
ISLR2	-0.337055553	4.85772E-11	2.65191E-10
TMEM91	0.337033875	4.87242E-11	2.6592E-10
PCDHB12	-0.336892727	4.96917E-11	2.71126E-10
SEPSECS	-0.336876067	4.98071E-11	2.71681E-10
KCTD3	-0.336691593	5.11028E-11	2.78672E-10
ILK	0.336679294	5.11903E-11	2.79073E-10
G6PD	0.336655906	5.13572E-11	2.79906E-10
LOC100133	-0.336512094	5.2395E-11	2.85484E-10
AQP4	-0.336475593	5.26616E-11	2.86859E-10
FRY	-0.336389853	5.32931E-11	2.90219E-10
BMX	-0.33637467	5.34057E-11	2.90753E-10
DLST	-0.336372127	5.34246E-11	2.90776E-10
RBPJ	0.33633286	5.3717E-11	2.92288E-10
SCN4B	-0.336293168	5.40141E-11	2.93824E-10
ABCB10	-0.336237529	5.44333E-11	2.96024E-10
ELOF1	0.33617829	5.48832E-11	2.98389E-10
XPA	0.336115628	5.53629E-11	3.00915E-10
AGTRAP	0.336091865	5.55459E-11	3.01827E-10
FZD4	-0.336088203	5.55742E-11	3.01898E-10
AFAP1L2	-0.336065934	5.57463E-11	3.02751E-10
ATP5D	0.336055314	5.58285E-11	3.03115E-10
CBLB	-0.335969745	5.64957E-11	3.06653E-10
ATAD3A	0.335902958	5.70218E-11	3.09425E-10
ZNF706	0.335859995	5.73627E-11	3.1119E-10
SLC2A14	-0.335780285	5.80006E-11	3.14565E-10
CRIM1	-0.335772795	5.80609E-11	3.14806E-10
ZBED1	-0.335769059	5.8091E-11	3.14883E-10
FAM110C	-0.335653311	5.90309E-11	3.19891E-10
STAM	-0.335632873	5.91984E-11	3.20712E-10
ADCY5	-0.335622463	5.92839E-11	3.21088E-10
SESN1	-0.335575201	5.96736E-11	3.2311E-10

ZNF407	-0.335567804	5.97348E-11	3.23318E-10
C9orf37	0.335566651	5.97443E-11	3.23318E-10
ZNF551	-0.335529797	6.00503E-11	3.24885E-10
CCDC85B	0.33548437	6.04295E-11	3.26848E-10
DST	-0.335441225	6.07918E-11	3.28718E-10
RNASET2	0.335377496	6.13308E-11	3.31543E-10
SOAT2	0.335315761	6.18575E-11	3.34299E-10
GPR174	-0.335250746	6.24168E-11	3.3723E-10
RAB43	-0.335238498	6.25227E-11	3.37711E-10
C7orf55	0.335234951	6.25534E-11	3.37785E-10
IGSF9B	-0.335141755	6.33656E-11	3.42078E-10
FANCI	0.335120283	6.35542E-11	3.43003E-10
FGD5	-0.335091634	6.38066E-11	3.44272E-10
PHKA1	-0.335018126	6.44588E-11	3.47627E-10
GPATCH4	0.335017619	6.44633E-11	3.47627E-10
GRINL1A	-0.335010443	6.45273E-11	3.47878E-10
UFM1	-0.334973535	6.48576E-11	3.49564E-10
PHACTR4	-0.334970769	6.48824E-11	3.49603E-10
BIRC2	-0.334967559	6.49112E-11	3.49664E-10
DENND5B	-0.33493384	6.52146E-11	3.51203E-10
NME1-NME	0.334842558	6.60429E-11	3.55543E-10
ADNP2	-0.334841095	6.60563E-11	3.55543E-10
RAD50	-0.334792296	6.65033E-11	3.57853E-10
TAF1D	0.334776281	6.66507E-11	3.58549E-10
CD84	-0.334736645	6.70168E-11	3.60421E-10
CHMP4A	0.334716304	6.72054E-11	3.61338E-10
TRO	-0.334642195	6.78971E-11	3.64958E-10
TRIM16L	0.334556079	6.87095E-11	3.69225E-10
ENO1	0.334469641	6.95345E-11	3.73558E-10
INE1	-0.334458069	6.96456E-11	3.74054E-10
DGAT1	0.334425864	6.99559E-11	3.75619E-10
BID	0.334421044	7.00025E-11	3.75768E-10
ZDHHC5	-0.334401459	7.0192E-11	3.76684E-10
KPNA2	0.334378686	7.04129E-11	3.77768E-10
SNX22	0.334376483	7.04344E-11	3.77781E-10
WHAMML2	-0.334286373	7.13156E-11	3.82404E-10
CASQ2	-0.334257233	7.16029E-11	3.83842E-10
GADD45A	-0.334226103	7.1911E-11	3.8539E-10
FBXW7	-0.334202864	7.21419E-11	3.86523E-10
FANCE	0.334176947	7.24002E-11	3.87802E-10
FLOT2	0.334135056	7.28196E-11	3.89944E-10
DOCK7	-0.334102453	7.31477E-11	3.91596E-10
ZNF680	-0.334060282	7.35742E-11	3.93773E-10
FAM120A	-0.334013711	7.4048E-11	3.96203E-10
ENTPD6	0.333935505	7.48504E-11	4.00388E-10
ARHGEF17	-0.333841457	7.58265E-11	4.05501E-10

AHCY	0.333822217	7.60277E-11	4.06468E-10
CHEK1	0.33370619	7.72522E-11	4.12903E-10
NKAP	0.333696323	7.73572E-11	4.13283E-10
UQCRC1	0.333695623	7.73647E-11	4.13283E-10
ZSCAN16	0.33368918	7.74333E-11	4.13538E-10
THAP9	-0.333657245	7.77745E-11	4.15249E-10
TAPT1	-0.333619517	7.81794E-11	4.17299E-10
ABCB6	0.333601058	7.83783E-11	4.18249E-10
AKR1C1	0.333599022	7.84002E-11	4.18254E-10
HNRNPA3P	0.333589557	7.85024E-11	4.18687E-10
DBNL	0.333568917	7.87257E-11	4.19765E-10
PDE12	-0.33356145	7.88067E-11	4.20084E-10
MIF	0.333481656	7.96766E-11	4.24608E-10
FAM124B	-0.333424125	8.03096E-11	4.27867E-10
FLJ39653	0.333361294	8.10065E-11	4.31464E-10
PIAS2	-0.333342171	8.12198E-11	4.32407E-10
CASC5	-0.333341537	8.12269E-11	4.32407E-10
ST6GALNAC	-0.333310388	8.15755E-11	4.34146E-10
RNFT2	0.333303116	8.16571E-11	4.34464E-10
GK3P	-0.333283521	8.18773E-11	4.3552E-10
GLUD2	-0.333270717	8.20216E-11	4.36171E-10
MRPS2	0.333234403	8.2432E-11	4.38236E-10
SHPK	-0.333228164	8.25027E-11	4.38495E-10
C3orf63	-0.333142344	8.34814E-11	4.43579E-10
SMAD5	-0.333084248	8.41504E-11	4.47014E-10
AKT1	-0.333007847	8.5038E-11	4.51609E-10
MFSD1	-0.332993862	8.52015E-11	4.52356E-10
CRKL	-0.332853468	8.68596E-11	4.61037E-10
FAM198A	-0.332734118	8.82939E-11	4.68525E-10
PYCR2	0.33271705	8.85009E-11	4.69498E-10
GLP2R	-0.332708097	8.86096E-11	4.6995E-10
PTPRC	-0.332642226	8.94138E-11	4.74089E-10
FAM98C	0.332607578	8.98397E-11	4.7622E-10
FRS3	0.33258857	9.00742E-11	4.77336E-10
RXRB	0.332574931	9.02428E-11	4.78102E-10
RBBP4	-0.332494404	9.12446E-11	4.83281E-10
FAM120B	-0.33247377	9.1503E-11	4.84521E-10
MINK1	-0.332466821	9.15902E-11	4.84853E-10
THEM4	0.332440058	9.19267E-11	4.86506E-10
RP1L1	-0.332354124	9.30156E-11	4.92137E-10
CMPK2	-0.332267287	9.41286E-11	4.97894E-10
NDUFAF4	0.332242807	9.44447E-11	4.99434E-10
BTC	-0.332208711	9.48867E-11	5.01638E-10
CEP290	-0.33218089	9.52489E-11	5.03419E-10
ATP6V1E1	0.332054203	9.69151E-11	5.12089E-10
FBLN5	-0.331984118	9.7849E-11	5.16887E-10

FATE1	0.331923947	9.86578E-11	5.21021E-10
MCM3	0.331915959	9.87656E-11	5.21452E-10
RSRC2	-0.331899478	9.89885E-11	5.2249E-10
GABRR2	-0.331890148	9.91149E-11	5.22914E-10
CACNB2	-0.331889684	9.91212E-11	5.22914E-10
DUSP1	-0.331887139	9.91557E-11	5.22957E-10
ZNF417	-0.331836898	9.98394E-11	5.26423E-10
NACC2	-0.331825713	9.99922E-11	5.27023E-10
IBTK	-0.331824707	1.00006E-10	5.27023E-10
FAM195A	0.331670203	1.02141E-10	5.3813E-10
ATG16L1	-0.331639015	1.02577E-10	5.40285E-10
TRMT2B	-0.331632767	1.02665E-10	5.40604E-10
SHANK2	-0.331622726	1.02805E-10	5.41203E-10
KLHL5	-0.331573135	1.03504E-10	5.44738E-10
GOLGA9P	-0.331509358	1.0441E-10	5.4936E-10
HIATL1	-0.331426043	1.05605E-10	5.55499E-10
TIFA	-0.331367081	1.06458E-10	5.59841E-10
PELI2	-0.331334345	1.06935E-10	5.622E-10
KIF1B	-0.331043999	1.11256E-10	5.84762E-10
PYGO2	0.331035199	1.1139E-10	5.85309E-10
ACE	-0.330996754	1.11975E-10	5.88099E-10
FOSL2	-0.330996459	1.11979E-10	5.88099E-10
C17orf85	-0.330961294	1.12518E-10	5.90769E-10
COL14A1	-0.330957353	1.12578E-10	5.90931E-10
TGFBR1	-0.330906973	1.13354E-10	5.94846E-10
KIAA0284	-0.330899977	1.13462E-10	5.95256E-10
ZNHIT2	0.33079984	1.15021E-10	6.03275E-10
C17orf59	0.330779688	1.15337E-10	6.04774E-10
FANCG	0.330675228	1.16989E-10	6.13278E-10
WFDC3	0.33064669	1.17445E-10	6.15504E-10
C1QTNF7	-0.330569438	1.18687E-10	6.21848E-10
C6orf150	-0.330495778	1.19882E-10	6.27948E-10
SMURF2	-0.330491051	1.1996E-10	6.28187E-10
ASF1B	0.33037551	1.2186E-10	6.37972E-10
CXXC1	0.330262981	1.23739E-10	6.4764E-10
PITPNA	-0.33013512	1.25909E-10	6.58822E-10
NEO1	-0.33009066	1.26672E-10	6.62641E-10
SP1	-0.330084715	1.26774E-10	6.63002E-10
LY6G5C	0.330023319	1.27836E-10	6.6838E-10
ZBTB20	-0.329886679	1.30231E-10	6.80721E-10
DHX29	-0.329868583	1.30551E-10	6.82217E-10
ZNF397OS	-0.329783936	1.3206E-10	6.89921E-10
TRAPPC6B	-0.329742108	1.32812E-10	6.93666E-10
PDGFRB	-0.329731661	1.33E-10	6.94468E-10
PFKFB3	-0.329725198	1.33117E-10	6.94896E-10
SPDYE3	-0.329647746	1.34523E-10	7.02053E-10

MGA	-0.329588474	1.35609E-10	7.07535E-10
FLNA	-0.329582354	1.35722E-10	7.07937E-10
NCKAP5	-0.329574233	1.35872E-10	7.08531E-10
EGR2	-0.329554316	1.36239E-10	7.10262E-10
PCDHGB4	-0.329490341	1.37426E-10	7.16263E-10
CHKA	0.329316628	1.407E-10	7.33136E-10
WDR20	-0.329237907	1.42209E-10	7.40803E-10
SEC11C	0.329052751	1.4582E-10	7.59414E-10
ATP2B4	-0.329009274	1.4668E-10	7.63697E-10
IQGAP2	-0.328982089	1.47221E-10	7.66312E-10
C14orf80	0.32895946	1.47673E-10	7.68461E-10
SRCAP	-0.32889053	1.49056E-10	7.75459E-10
ZBTB6	-0.328886693	1.49134E-10	7.75659E-10
NBLA00301	-0.328863942	1.49594E-10	7.77847E-10
WNT9B	-0.328859115	1.49691E-10	7.78152E-10
SLC46A3	-0.32859095	1.55218E-10	8.06674E-10
ZBTB10	-0.328580915	1.55429E-10	8.07558E-10
TMTC3	-0.328571216	1.55633E-10	8.08406E-10
ECSIT	0.328538733	1.56318E-10	8.11751E-10
EGR3	-0.328501064	1.57115E-10	8.1568E-10
NAPRT1	0.328492307	1.57301E-10	8.16433E-10
NDUFV3	0.328484684	1.57463E-10	8.17061E-10
TJAP1	0.328368744	1.59948E-10	8.2974E-10
DCTN2	0.328361734	1.601E-10	8.3031E-10
PDZD11	0.328273762	1.62013E-10	8.40012E-10
DCK	-0.328269796	1.621E-10	8.40243E-10
ISCA2	0.328212616	1.63355E-10	8.46532E-10
AP4M1	0.328188364	1.63891E-10	8.48937E-10
MCM5	0.328187744	1.63905E-10	8.48937E-10
APLP2	-0.328156436	1.64599E-10	8.52309E-10
UTP11L	0.32811608	1.65497E-10	8.5674E-10
SCN11A	-0.328040925	1.67184E-10	8.65217E-10
PTER	-0.328039236	1.67222E-10	8.65217E-10
PDE8B	-0.328005923	1.67975E-10	8.68887E-10
OMD	-0.328003174	1.68037E-10	8.68983E-10
PHOSPHO1	-0.327997685	1.68161E-10	8.69401E-10
HIST1H1C	0.327995567	1.68209E-10	8.69424E-10
NEURL2	0.327990881	1.68316E-10	8.69748E-10
KHDRBS1	0.327974055	1.68698E-10	8.71497E-10
C20orf94	-0.327968686	1.6882E-10	8.71902E-10
CYP2W1	0.327890407	1.70611E-10	8.80626E-10
STAT3	-0.327890207	1.70616E-10	8.80626E-10
ITGB1BP1	0.327889069	1.70642E-10	8.80626E-10
TRIM33	-0.32785108	1.71518E-10	8.84918E-10
CDC73	-0.327817802	1.72289E-10	8.88665E-10
FLRT2	-0.327781466	1.73135E-10	8.92796E-10

NT5DC1	-0.327701737	1.75005E-10	9.02204E-10
CHSY3	-0.327685486	1.75388E-10	9.03948E-10
ZFYVE19	0.327655453	1.76099E-10	9.07377E-10
TWF2	0.327652772	1.76163E-10	9.0747E-10
C7	-0.327596474	1.77503E-10	9.1414E-10
MRPL37	0.327487454	1.80128E-10	9.27416E-10
RUFY2	-0.327480815	1.80289E-10	9.28005E-10
C16orf75	0.327374148	1.82895E-10	9.41179E-10
DOCK6	-0.327306119	1.84577E-10	9.49587E-10
PANK2	0.327251978	1.85926E-10	9.5628E-10
VPS39	-0.32716061	1.88224E-10	9.67851E-10
PHF3	-0.327151146	1.88464E-10	9.68833E-10
ZNF232	0.327123972	1.89154E-10	9.72128E-10
RNF180	-0.327103484	1.89675E-10	9.74557E-10
RIOK1	0.327063648	1.90694E-10	9.79536E-10
CLN8	-0.327004156	1.92224E-10	9.87144E-10
COL3A1	-0.32693489	1.94021E-10	9.96116E-10
C15orf52	-0.326804406	1.97451E-10	1.01347E-09
PHF12	-0.326790202	1.97828E-10	1.01514E-09
KCTD2	0.326702455	2.00172E-10	1.0269E-09
PSMG2	0.326698404	2.00281E-10	1.0272E-09
KIAA1530	-0.326648106	2.01638E-10	1.03389E-09
KIF3A	-0.326638442	2.01899E-10	1.03496E-09
NDUFC1	0.326622883	2.02321E-10	1.03686E-09
RASGRP3	-0.326576824	2.03575E-10	1.04302E-09
MAP3K7	-0.326551308	2.04273E-10	1.04632E-09
HMGN1	0.326424314	2.07782E-10	1.06402E-09
TMEM9	0.326421947	2.07848E-10	1.06409E-09
MRPS30	0.326380327	2.09011E-10	1.06977E-09
ADRA1B	-0.326377919	2.09079E-10	1.06984E-09
C9orf80	-0.326343753	2.10039E-10	1.07447E-09
ABAT	-0.32632784	2.10487E-10	1.07649E-09
LARP4	-0.32632461	2.10578E-10	1.07668E-09
TUFM	0.32631228	2.10927E-10	1.07818E-09
MGMT	0.326270689	2.12105E-10	1.08393E-09
DNAJC16	-0.326258222	2.1246E-10	1.08547E-09
PRDM10	-0.326251517	2.12651E-10	1.08616E-09
CLDND1	-0.326204573	2.13993E-10	1.09274E-09
ZNF721	-0.326047128	2.18553E-10	1.11574E-09
JOSD2	0.325999382	2.19955E-10	1.1226E-09
CRHBP	-0.325982025	2.20467E-10	1.12493E-09
CAPN7	-0.325980028	2.20526E-10	1.12494E-09
TMEM38A	-0.325947341	2.21493E-10	1.12958E-09
ZNF446	0.325914951	2.22455E-10	1.1342E-09
YME1L1	-0.325910706	2.22581E-10	1.13455E-09
PHF5A	0.325880236	2.23491E-10	1.1389E-09

EIF4E3	-0.325853485	2.24292E-10	1.14269E-09
CCT2	0.325830884	2.24971E-10	1.14586E-09
CD164	-0.325787598	2.26278E-10	1.15222E-09
ERO1L	-0.325775477	2.26645E-10	1.15379E-09
NEK4	-0.325772854	2.26725E-10	1.1539E-09
DALRD3	0.32574401	2.27601E-10	1.15807E-09
HSD17B1	0.325728637	2.2807E-10	1.16003E-09
LRRK1	-0.325727538	2.28103E-10	1.16003E-09
STAT5B	-0.325675838	2.29686E-10	1.16778E-09
RLIM	-0.325647544	2.30556E-10	1.17187E-09
MYO5A	-0.325645888	2.30607E-10	1.17187E-09
GREB1L	-0.325643268	2.30688E-10	1.17198E-09
LPIN2	-0.325637841	2.30855E-10	1.17253E-09
UBE2V2	0.325556895	2.33367E-10	1.18498E-09
BACH2	-0.325541827	2.33837E-10	1.18707E-09
FRMD4A	-0.325489533	2.35477E-10	1.19509E-09
TPI1P2	-0.325469017	2.36124E-10	1.19807E-09
ENDOG	0.325391317	2.38588E-10	1.21026E-09
CAST	-0.325344785	2.40075E-10	1.21749E-09
MBOAT7	0.325328058	2.40612E-10	1.21991E-09
TRIM5	-0.325307284	2.4128E-10	1.22298E-09
PEX6	0.32526316	2.42706E-10	1.2299E-09
NAP1L1	0.325255195	2.42964E-10	1.23089E-09
CPOX	-0.325214578	2.44285E-10	1.23727E-09
FAM89B	0.325200821	2.44734E-10	1.23923E-09
RNF144B	-0.325185893	2.45223E-10	1.24138E-09
RNPC3	-0.325166706	2.45851E-10	1.24425E-09
OLA1	0.325100359	2.48038E-10	1.255E-09
RABAC1	0.325057315	2.49466E-10	1.26191E-09
NAP1L5	-0.325000944	2.51349E-10	1.27111E-09
SLC2A4RG	0.324889329	2.55119E-10	1.28962E-09
XRN2	-0.324888119	2.5516E-10	1.28962E-09
DNPEP	0.324886789	2.55205E-10	1.28962E-09
WASF3	-0.324866138	2.55909E-10	1.29285E-09
RNF146	-0.324773691	2.59082E-10	1.30855E-09
FOXF1	-0.324723227	2.6083E-10	1.31705E-09
ANKRA2	-0.324709928	2.61293E-10	1.31905E-09
FLG	-0.324625834	2.64236E-10	1.33347E-09
ZNF449	-0.324624501	2.64283E-10	1.33347E-09
C17orf62	0.324583438	2.65733E-10	1.34044E-09
MCFD2	-0.324579842	2.6586E-10	1.34075E-09
YPEL2	-0.324511567	2.68288E-10	1.35265E-09
HNRNPH2	-0.324451936	2.70427E-10	1.36308E-09
ANKRD13C	-0.324423029	2.71469E-10	1.36799E-09
RFWD2	0.324365519	2.73555E-10	1.37816E-09
PRKD3	-0.32435446	2.73958E-10	1.37984E-09

C6orf211	-0.324338354	2.74546E-10	1.38245E-09
DTX2	0.324266829	2.77171E-10	1.39531E-09
ELMO2	-0.32424076	2.78134E-10	1.39981E-09
TET3	-0.324213975	2.79126E-10	1.40445E-09
NBPF3	-0.324173925	2.80617E-10	1.41159E-09
HRH2	-0.324156713	2.8126E-10	1.41447E-09
HYI	0.324152941	2.81401E-10	1.41482E-09
S100A10	0.324132845	2.82154E-10	1.41825E-09
DNAL1	-0.324129859	2.82266E-10	1.41845E-09
PAFAH2	-0.324094926	2.8358E-10	1.4247E-09
ZNF668	0.32408901	2.83803E-10	1.42546E-09
TMEM30B	-0.324060028	2.84899E-10	1.4306E-09
GNB1L	0.324054799	2.85097E-10	1.43123E-09
FRMPD1	-0.323995488	2.87353E-10	1.4422E-09
MAGI3	-0.323984286	2.87781E-10	1.44398E-09
N4BP2L1	-0.323962631	2.88611E-10	1.44778E-09
CLNS1A	0.323941553	2.8942E-10	1.45147E-09
ARSJ	-0.323937829	2.89563E-10	1.45183E-09
SARS2	0.323930441	2.89848E-10	1.45289E-09
VPS29	0.323921359	2.90198E-10	1.45427E-09
PLEKHG5	-0.323909245	2.90665E-10	1.45625E-09
C14orf106	-0.323883018	2.91679E-10	1.46094E-09
MYL12B	0.323881255	2.91748E-10	1.46094E-09
WDR62	0.323794664	2.95123E-10	1.47747E-09
BMPER	-0.323779853	2.95704E-10	1.48E-09
ZNF628	0.323773568	2.95951E-10	1.48087E-09
C9orf72	-0.323737043	2.97389E-10	1.48769E-09
FOXN3	-0.323721288	2.98012E-10	1.49043E-09
DUSP12	0.32367048	3.00029E-10	1.50014E-09
TUBGCP2	0.323615243	3.02237E-10	1.5108E-09
TBC1D13	0.3236065	3.02587E-10	1.51218E-09
GCAT	0.323421609	3.10101E-10	1.54934E-09
MESP2	0.323388921	3.11448E-10	1.55568E-09
THADA	0.32337022	3.12221E-10	1.55915E-09
FAM54A	0.323343716	3.1332E-10	1.56425E-09
C12orf52	0.323331156	3.13843E-10	1.56646E-09
GAS6	-0.323320632	3.14281E-10	1.56825E-09
HRNR	-0.323263052	3.16689E-10	1.57987E-09
SRPR	-0.323219653	3.18515E-10	1.58852E-09
MED10	0.3232163	3.18657E-10	1.58852E-09
ACTR5	0.323216186	3.18662E-10	1.58852E-09
SNRNP70	0.323211815	3.18846E-10	1.58905E-09
NARFL	0.323208426	3.1899E-10	1.58936E-09
MEX3B	-0.323125535	3.22512E-10	1.60651E-09
DOPEY2	-0.323056153	3.2549E-10	1.62094E-09
RXFP1	-0.323039574	3.26205E-10	1.62409E-09

E2F1	0.323002706	3.27802E-10	1.63164E-09
SLC22A8	0.322893715	3.32566E-10	1.65494E-09
NHEDC2	-0.322795386	3.36922E-10	1.67619E-09
GAPVD1	-0.322705793	3.40939E-10	1.69575E-09
SSBP4	0.322650656	3.43434E-10	1.70774E-09
EXPH5	-0.322626605	3.44528E-10	1.71275E-09
CPSF3L	0.32261203	3.45193E-10	1.71563E-09
TNFRSF4	0.322486703	3.50959E-10	1.74385E-09
RSPH3	-0.322409934	3.54538E-10	1.76119E-09
PTPRK	-0.322367037	3.56553E-10	1.77076E-09
NSDHL	0.322351579	3.57281E-10	1.77394E-09
AHDC1	-0.322307423	3.59371E-10	1.78387E-09
TTC21B	-0.322203736	3.64326E-10	1.80801E-09
PRKRIP1	0.322178299	3.65551E-10	1.81364E-09
DYNC2H1	-0.322083404	3.70159E-10	1.83587E-09
PURA	-0.322082233	3.70216E-10	1.83587E-09
C12orf72	-0.322040575	3.72257E-10	1.84553E-09
CLIC5	-0.321945188	3.76971E-10	1.86843E-09
PRR7	0.321938751	3.77291E-10	1.86956E-09
LUZP6	-0.321885038	3.79973E-10	1.88238E-09
GIT2	-0.321833847	3.82546E-10	1.89466E-09
SMYD4	-0.321819645	3.83263E-10	1.89774E-09
PFDN1	0.321814684	3.83514E-10	1.8985E-09
MAN2A2	-0.321754368	3.86576E-10	1.91318E-09
DUSP19	-0.321739281	3.87345E-10	1.91652E-09
FAM32A	0.321690308	3.89853E-10	1.92845E-09
TBRG1	-0.321687279	3.90009E-10	1.92874E-09
CAT	-0.321603236	3.94351E-10	1.94973E-09
GFPT1	-0.321493534	4.00091E-10	1.97761E-09
C20orf111	0.32148985	4.00285E-10	1.97808E-09
PPP2CB	-0.321486406	4.00466E-10	1.97849E-09
ZFP3	-0.321467061	4.01488E-10	1.98304E-09
CA2	-0.321358807	4.0725E-10	2.01101E-09
TTPAL	-0.321350338	4.07704E-10	2.01275E-09
BCL9L	-0.321338325	4.08349E-10	2.01543E-09
ALKBH6	0.32129041	4.10932E-10	2.02768E-09
BRP44	0.321240318	4.1365E-10	2.04058E-09
C20orf43	0.321229173	4.14257E-10	2.04307E-09
SRFBP1	-0.321209673	4.15321E-10	2.04781E-09
DEDD2	0.321206793	4.15478E-10	2.04808E-09
NPIPL3	0.321141372	4.19068E-10	2.06527E-09
IQSEC3	-0.321071477	4.22938E-10	2.08382E-09
PIP4K2C	-0.321023875	4.25593E-10	2.09638E-09
MDFIC	-0.321007191	4.26527E-10	2.10047E-09
FAM103A1	0.320868085	4.34396E-10	2.13869E-09
MRPS28	0.320864699	4.34589E-10	2.13911E-09

AAMP	0.32082409	4.36914E-10	2.15002E-09
ABHD15	-0.320704921	4.43805E-10	2.18339E-09
HJURP	0.320676943	4.45439E-10	2.19089E-09
ACACB	-0.320626293	4.4841E-10	2.20496E-09
TARDBP	0.320609488	4.494E-10	2.20928E-09
SAE1	0.320560305	4.52311E-10	2.22304E-09
PRPF38B	-0.320460779	4.58256E-10	2.25171E-09
QTRTD1	-0.320391784	4.62422E-10	2.27161E-09
DCN	-0.320361846	4.64241E-10	2.27999E-09
MPI	0.320337835	4.65705E-10	2.28661E-09
STXBP1	-0.320279762	4.69264E-10	2.30352E-09
CIB3	0.320179876	4.75448E-10	2.3333E-09
GLS	-0.320145702	4.77582E-10	2.3432E-09
DCAF12	-0.320107406	4.79984E-10	2.35441E-09
TRPC4	-0.320066664	4.82553E-10	2.36642E-09
PPL	-0.319980524	4.88028E-10	2.39268E-09
CBX8	0.319765457	5.01962E-10	2.46039E-09
EDA2R	-0.319745733	5.03259E-10	2.46614E-09
SMC6	-0.319651434	5.09505E-10	2.49614E-09
MED1	-0.319604846	5.12619E-10	2.51077E-09
DHPS	0.319598549	5.13041E-10	2.51222E-09
CDC40	-0.319568831	5.15039E-10	2.52139E-09
HOXB3	-0.319494497	5.20068E-10	2.54538E-09
KTI12	0.319439201	5.23841E-10	2.56322E-09
SYPL1	-0.319437091	5.23985E-10	2.56329E-09
ARFGAP3	-0.319417631	5.25319E-10	2.56919E-09
PMM1	0.319410556	5.25805E-10	2.57093E-09
MMAA	-0.319384017	5.27632E-10	2.57923E-09
CEP135	-0.319346437	5.30229E-10	2.59129E-09
WBP1	0.319291095	5.34076E-10	2.60945E-09
TRIP13	0.319249711	5.36971E-10	2.62295E-09
EXOC8	-0.319231038	5.38281E-10	2.62871E-09
ACOT13	0.319190834	5.41115E-10	2.6419E-09
MPG	0.319140717	5.44667E-10	2.65859E-09
GNL3L	-0.31910109	5.47492E-10	2.67172E-09
ZNF154	-0.319085253	5.48625E-10	2.67659E-09
APOLD1	-0.319081722	5.48877E-10	2.67717E-09
C17orf81	0.319063794	5.50163E-10	2.68279E-09
POLR3K	0.319038492	5.51983E-10	2.691E-09
KIAA1147	-0.319035173	5.52222E-10	2.69151E-09
ZHX3	-0.318969435	5.56979E-10	2.71403E-09
HIST1H1A	0.318942877	5.58912E-10	2.72278E-09
ASAH2B	-0.318933345	5.59607E-10	2.7255E-09
COL6A6	-0.318839787	5.66478E-10	2.75829E-09
LTBP2	-0.318779658	5.70936E-10	2.77932E-09
ASAP2	-0.318771351	5.71555E-10	2.78165E-09

ARPC1A	0.318691248	5.77554E-10	2.81016E-09
ABI1	-0.318636127	5.81717E-10	2.82973E-09
PSMA1	0.318625586	5.82517E-10	2.83292E-09
SLC7A2	-0.318607872	5.83863E-10	2.83878E-09
PRRC1	-0.31851943	5.90629E-10	2.87097E-09
MPRIP	-0.318479752	5.93689E-10	2.88514E-09
ARNTL	-0.318434505	5.97197E-10	2.90148E-09
FMNL3	-0.31837302	6.01996E-10	2.92409E-09
ASAH1	-0.318368056	6.02385E-10	2.92526E-09
C1orf156	0.318336278	6.04882E-10	2.93667E-09
FEN1	0.318323499	6.05889E-10	2.94084E-09
OGG1	0.318273847	6.09817E-10	2.95919E-09
DEPDC5	-0.318254399	6.11362E-10	2.96596E-09
H2AFB1	0.318218633	6.14214E-10	2.97907E-09
STX11	-0.318215509	6.14463E-10	2.97956E-09
LOC375190	0.318194564	6.1614E-10	2.98696E-09
RALGDS	-0.31818426	6.16966E-10	2.99024E-09
EIF2C3	-0.31803551	6.29018E-10	3.0479E-09
BAD	0.318018989	6.3037E-10	3.05371E-09
SLIT3	-0.318010363	6.31077E-10	3.0564E-09
SNAPIN	0.317944244	6.36525E-10	3.08203E-09
LRRC57	-0.317940081	6.36869E-10	3.08295E-09
TCEANC	-0.317835112	6.45615E-10	3.12453E-09
HDDC2	0.317831921	6.45883E-10	3.12506E-09
SEMA3G	-0.317718384	6.55479E-10	3.17072E-09
TRIM54	0.317698071	6.5721E-10	3.17832E-09
GOLPH3	-0.317689958	6.57903E-10	3.1809E-09
LRRC8C	-0.317615586	6.64286E-10	3.21098E-09
MCTP1	-0.317479845	6.76092E-10	3.26726E-09
RAB14	-0.317462563	6.77609E-10	3.2738E-09
LOC145820	-0.317416874	6.81637E-10	3.29246E-09
WASL	-0.317373174	6.85512E-10	3.31037E-09
ANKRD5	-0.317369125	6.85872E-10	3.31131E-09
MAFB	-0.31734284	6.88214E-10	3.32181E-09
NUF2	0.317302424	6.9183E-10	3.33845E-09
PCDHB14	-0.31730043	6.92009E-10	3.33851E-09
CCBE1	-0.31729606	6.92401E-10	3.33959E-09
STIM2	-0.317281344	6.93724E-10	3.34516E-09
AKAP12	-0.317268014	6.94924E-10	3.35013E-09
PRDX6	0.317244081	6.97083E-10	3.35973E-09
HNRNPL	0.317194537	7.01574E-10	3.38056E-09
MTR	-0.317107085	7.0957E-10	3.41826E-09
EFNA2	-0.31710002	7.1022E-10	3.42056E-09
COPB1	-0.317073234	7.12689E-10	3.43162E-09
FBXO36	-0.3169571	7.2349E-10	3.48279E-09
NCLN	0.316952385	7.23932E-10	3.48408E-09

ERMAP	-0.316863376	7.32323E-10	3.52361E-09
CRK	-0.316819591	7.36485E-10	3.54278E-09
FAM162A	0.316774248	7.4082E-10	3.56277E-09
URB1	-0.316769393	7.41285E-10	3.56415E-09
MCCD1	0.316610606	7.5667E-10	3.63724E-09
ZNF443	-0.316594501	7.58248E-10	3.64394E-09
EMD	0.316567646	7.60886E-10	3.65574E-09
TCF21	-0.316558721	7.61764E-10	3.65908E-09
CSTB	0.316542634	7.6335E-10	3.66581E-09
PTGFR	-0.316530876	7.64512E-10	3.6705E-09
GCHFR	0.316481618	7.69396E-10	3.69306E-09
MAPKBP1	-0.316471088	7.70444E-10	3.6972E-09
CPT1A	-0.316457364	7.71811E-10	3.70243E-09
RAD54L	0.316456428	7.71905E-10	3.70243E-09
RHOC	0.316428267	7.7472E-10	3.71503E-09
SIRT3	0.316399237	7.77631E-10	3.7281E-09
ATG4C	-0.316363905	7.8119E-10	3.74426E-09
PRIM2	0.316355966	7.81992E-10	3.7472E-09
BDKRB2	-0.316342639	7.83339E-10	3.75276E-09
NR4A2	-0.316265645	7.9117E-10	3.78936E-09
HIST1H2AM	0.31624423	7.93361E-10	3.79894E-09
PAK1IP1	0.316216444	7.96213E-10	3.81168E-09
FOS	-0.316151057	8.02964E-10	3.84307E-09
PLXDC2	-0.316033278	8.15265E-10	3.90101E-09
CTR9	-0.316029018	8.15713E-10	3.90222E-09
PBRM1	-0.316011832	8.17524E-10	3.90994E-09
C12orf27	0.315992335	8.19583E-10	3.91885E-09
PLSCR1	-0.315943315	8.24783E-10	3.94276E-09
LOC100128	0.315941283	8.25E-10	3.94285E-09
DCLRE1A	-0.315935061	8.25662E-10	3.94507E-09
SGCD	-0.315930043	8.26197E-10	3.94668E-09
ZNF775	0.315900082	8.29396E-10	3.96101E-09
SYDE2	-0.315873762	8.32216E-10	3.97353E-09
HMG20A	-0.315857362	8.33978E-10	3.98099E-09
DDX60L	-0.315783248	8.41987E-10	4.01825E-09
COX4I2	0.315763598	8.44123E-10	4.02748E-09
TUBGCP3	-0.315679403	8.53334E-10	4.07045E-09
PDZRN4	-0.315674179	8.53909E-10	4.07222E-09
C6orf64	0.315617199	8.60202E-10	4.10125E-09
PTCH2	-0.315569498	8.65505E-10	4.12555E-09
MYL5	0.315557788	8.66812E-10	4.13079E-09
ZMYM5	-0.315554627	8.67165E-10	4.13148E-09
BANF2	0.315498753	8.73428E-10	4.16033E-09
EP400	-0.315472025	8.7644E-10	4.17367E-09
IQCD	0.31546131	8.7765E-10	4.17844E-09
MTCP1	-0.315455375	8.78321E-10	4.18063E-09

DNTTIP2	0.315440294	8.80029E-10	4.18776E-09
C6orf52	0.315430536	8.81135E-10	4.19202E-09
RPL23AP82	0.315400489	8.8455E-10	4.20727E-09
LOC647979	-0.315388374	8.85931E-10	4.21283E-09
GOLGA6B	-0.31534422	8.90981E-10	4.23583E-09
PRUNE2	-0.315300419	8.96019E-10	4.25876E-09
TSSK4	-0.315249656	9.01891E-10	4.28565E-09
GSK3B	-0.315242272	9.02749E-10	4.2887E-09
LARS2	-0.315192427	9.08557E-10	4.31526E-09
MAMLD1	-0.315165697	9.11686E-10	4.3291E-09
PKP2	-0.31511683	9.17435E-10	4.35535E-09
CCT6A	0.315064412	9.2364E-10	4.38377E-09
LTA4H	0.315058228	9.24375E-10	4.38621E-09
MBD3	0.31502317	9.28551E-10	4.40498E-09
SSPN	-0.314845186	9.50037E-10	4.50583E-09
C20orf7	0.314823304	9.52712E-10	4.51744E-09
FAM82A1	-0.314752213	9.61452E-10	4.5578E-09
GALK1	0.314633653	9.76201E-10	4.62662E-09
SLC35E1	-0.314603967	9.79929E-10	4.64318E-09
RASSF5	-0.314519807	9.90571E-10	4.69249E-09
SLFN11	-0.314517836	9.90822E-10	4.69256E-09
PLK2	-0.314512131	9.91547E-10	4.69488E-09
BCS1L	0.314472848	9.96558E-10	4.71748E-09
TGFBR3	-0.314445969	1E-09	4.73266E-09
TRIM39	0.314415308	1.00394E-09	4.75018E-09
KLHL36	-0.314386551	1.00765E-09	4.7666E-09
DAG1	-0.31437692	1.0089E-09	4.77136E-09
STX12	-0.314356205	1.01158E-09	4.78292E-09
FBXL17	-0.314311889	1.01735E-09	4.80848E-09
ZNF434	-0.314310202	1.01757E-09	4.80848E-09
FAM129A	-0.31430908	1.01771E-09	4.80848E-09
RAD9A	0.314268838	1.02298E-09	4.83221E-09
MRPL4	0.314217796	1.02969E-09	4.86278E-09
TRIM16	0.314206737	1.03115E-09	4.86853E-09
TNFAIP3	-0.314164709	1.03672E-09	4.89366E-09
ZNF664	-0.314142583	1.03967E-09	4.90639E-09
STOX2	-0.314104691	1.04473E-09	4.92911E-09
CRISPLD2	-0.314094466	1.0461E-09	4.9344E-09
KCNA3	-0.314018529	1.05632E-09	4.98146E-09
TXK	-0.31400798	1.05775E-09	4.98701E-09
SPRY4	-0.313992746	1.05982E-09	4.99557E-09
TMTC2	-0.313963648	1.06377E-09	5.01303E-09
BMP10	-0.31386324	1.07754E-09	5.0767E-09
VAMP8	0.313763797	1.09134E-09	5.14051E-09
CD300E	-0.313716055	1.09803E-09	5.17078E-09
UBQLN2	-0.313711813	1.09863E-09	5.17237E-09

LHFP	-0.313699498	1.10036E-09	5.1793E-09
COMTD1	0.31355387	1.12105E-09	5.27543E-09
PSMF1	0.313492226	1.12992E-09	5.31591E-09
FAM186B	-0.313459957	1.13459E-09	5.33663E-09
RNF41	-0.31343758	1.13784E-09	5.35065E-09
SLC39A9	-0.313415049	1.14112E-09	5.36481E-09
COL4A2	-0.313369551	1.14777E-09	5.39482E-09
MMRN1	-0.313338163	1.15238E-09	5.41522E-09
SCRN2	0.313308113	1.15682E-09	5.43477E-09
MN1	-0.313199009	1.17305E-09	5.50974E-09
SHANK3	-0.313151296	1.18022E-09	5.5421E-09
ANKRD36B	-0.313070046	1.19252E-09	5.59813E-09
ATXN3	-0.313068803	1.19271E-09	5.59813E-09
MAN2B2	-0.31299006	1.20476E-09	5.65334E-09
GATSL1	-0.312971144	1.20767E-09	5.66567E-09
CCDC73	0.312938805	1.21266E-09	5.68775E-09
ANGPTL7	-0.312896909	1.21916E-09	5.71688E-09
TCF20	-0.312839543	1.22811E-09	5.75751E-09
MAMDC4	-0.312803615	1.23375E-09	5.78259E-09
API5	-0.312774993	1.23826E-09	5.80236E-09
FMO4	-0.312730164	1.24536E-09	5.83425E-09
TSTD1	0.312683099	1.25286E-09	5.86797E-09
CCDC158	-0.312661564	1.2563E-09	5.88271E-09
C3orf71	0.312634883	1.26058E-09	5.90136E-09
TNKS1BP1	-0.312617458	1.26338E-09	5.91309E-09
DULLARD	0.312587294	1.26824E-09	5.93447E-09
PTAFR	-0.312577154	1.26988E-09	5.94074E-09
STK4	-0.312548171	1.27458E-09	5.96132E-09
DHDH	0.312540279	1.27586E-09	5.96591E-09
THRA	-0.312384176	1.30148E-09	6.08427E-09
SATL1	-0.312371089	1.30365E-09	6.09299E-09
TUBGCP6	-0.312369162	1.30397E-09	6.09305E-09
RNF138P1	-0.312315303	1.31294E-09	6.13353E-09
CCS	0.312299427	1.3156E-09	6.14449E-09
MRVI1	-0.312265696	1.32126E-09	6.16948E-09
SYS1	0.312217457	1.32939E-09	6.20601E-09
POLRMT	0.312176866	1.33627E-09	6.23668E-09
DTNB	0.312054415	1.35725E-09	6.33308E-09
IFIH1	-0.312045128	1.35885E-09	6.33908E-09
POLA2	0.312010573	1.36484E-09	6.3655E-09
TBX20	-0.311992702	1.36794E-09	6.37849E-09
PITPNM1	-0.311981788	1.36984E-09	6.38585E-09
C17orf56	0.311947145	1.37588E-09	6.41253E-09
LRRC61	0.311927134	1.37939E-09	6.42736E-09
RNFT1	-0.31187411	1.38871E-09	6.4693E-09
CCDC127	0.311866271	1.3901E-09	6.47423E-09

ADAMTS18	-0.311838977	1.39493E-09	6.4952E-09
ACVR1B	-0.31181316	1.39951E-09	6.51502E-09
XDH	-0.311763401	1.40838E-09	6.5548E-09
LIFR	-0.311708614	1.41822E-09	6.59902E-09
LIMCH1	-0.311562779	1.44472E-09	6.72077E-09
FZD1	-0.311495301	1.45715E-09	6.77699E-09
SAAL1	0.311464906	1.46278E-09	6.80159E-09
FERMT2	-0.311334488	1.48718E-09	6.91343E-09
SFRS18	-0.31131943	1.49002E-09	6.92502E-09
REEP2	0.311305047	1.49274E-09	6.93605E-09
ZNF638	-0.311284287	1.49667E-09	6.9527E-09
CDC37	0.311207789	1.51126E-09	7.01882E-09
RGS14	0.311172891	1.51796E-09	7.04829E-09
DYNC1I2	-0.311168937	1.51872E-09	7.05018E-09
CREB3L4	0.311154449	1.52151E-09	7.06149E-09
PSMA3	0.311144099	1.52351E-09	7.06911E-09
ZNF717	-0.311142017	1.52391E-09	7.06933E-09
ARHGEF10L	-0.31100865	1.54988E-09	7.18812E-09
KLF6	-0.310970793	1.55732E-09	7.22098E-09
PRR12	-0.310961912	1.55908E-09	7.22743E-09
SNHG12	0.310801885	1.59099E-09	7.37363E-09
CWC27	0.310710967	1.60939E-09	7.45721E-09
ABCA2	-0.310697243	1.61219E-09	7.46843E-09
GUCY1B3	-0.310661608	1.61948E-09	7.50043E-09
NECAB3	0.310619462	1.62813E-09	7.53877E-09
RGS5	-0.310519834	1.64877E-09	7.63257E-09
CNST	-0.310516636	1.64944E-09	7.63388E-09
ATP7B	-0.310466496	1.65993E-09	7.68064E-09
ETNK1	-0.310462161	1.66084E-09	7.68306E-09
TMCC3	-0.310450453	1.6633E-09	7.69265E-09
LOC80154	0.310376644	1.67888E-09	7.76294E-09
CG030	-0.310282078	1.69906E-09	7.85442E-09
EAF2	0.310253616	1.70518E-09	7.88088E-09
ZNRF2	-0.310217824	1.71291E-09	7.91475E-09
GALNTL2	-0.310153384	1.7269E-09	7.97756E-09
SPATA18	-0.310115868	1.7351E-09	8.01357E-09
NAIF1	0.310030393	1.75392E-09	8.09862E-09
ZFP92	-0.309976957	1.76579E-09	8.15152E-09
TMEM60	0.309895684	1.78398E-09	8.23362E-09
PLXNB2	-0.309879612	1.7876E-09	8.24841E-09
MUT	-0.30985756	1.79258E-09	8.26947E-09
EDEM3	-0.309797462	1.80622E-09	8.33045E-09
CLEC2L	0.309777008	1.81088E-09	8.35002E-09
FAM41C	0.309683225	1.83242E-09	8.44737E-09
CEP170L	-0.309633472	1.84394E-09	8.49853E-09
C11orf2	0.309534039	1.86719E-09	8.60367E-09

ZCCHC8	-0.309475923	1.88091E-09	8.66487E-09
HARS	0.30946047	1.88457E-09	8.67974E-09
PLDN	-0.309435714	1.89045E-09	8.70483E-09
ANKRD42	-0.309351543	1.91059E-09	8.79553E-09
SNORD17	0.309286288	1.92635E-09	8.86601E-09
TNFRSF18	0.309280328	1.92779E-09	8.87062E-09
ZNF771	0.309203447	1.94653E-09	8.95477E-09
AFG3L2	-0.309172603	1.9541E-09	8.98751E-09
EIF3C	-0.309090093	1.97449E-09	9.07917E-09
SLC4A7	-0.309061387	1.98163E-09	9.10989E-09
PIGH	0.30904463	1.9858E-09	9.127E-09
ZNF425	-0.309031571	1.98907E-09	9.13989E-09
NIPAL2	-0.309028669	1.98979E-09	9.14112E-09
DCUN1D5	0.309022088	1.99144E-09	9.14657E-09
LRRC2	-0.308860603	2.03227E-09	9.33195E-09
KANK1	-0.308856632	2.03329E-09	9.33446E-09
THBS2	-0.308815967	2.0437E-09	9.38011E-09
PIGK	-0.308811278	2.0449E-09	9.38347E-09
PGGT1B	-0.308749175	2.06092E-09	9.45478E-09
SFN	0.308720942	2.06824E-09	9.48617E-09
PCMTD2	-0.308711502	2.07069E-09	9.49524E-09
ORAI1	0.308673963	2.08047E-09	9.5379E-09
TSPYL5	-0.308669526	2.08163E-09	9.54102E-09
CCDC163P	-0.308603263	2.09902E-09	9.61851E-09
HEBP2	0.308503157	2.12556E-09	9.73787E-09
GUCY1A2	-0.308489242	2.12927E-09	9.75264E-09
TUBGCP5	-0.308483678	2.13076E-09	9.75721E-09
RARB	-0.308467404	2.13512E-09	9.7749E-09
DEAF1	0.308396688	2.15414E-09	9.85973E-09
CCDC144A	-0.308391083	2.15566E-09	9.8644E-09
UPF2	-0.308363955	2.163E-09	9.89575E-09
ABCD3	-0.308355601	2.16527E-09	9.90384E-09
PLAA	-0.308286733	2.18405E-09	9.98744E-09
IGSF10	-0.308259685	2.19147E-09	1.00191E-08
CAMLG	0.308178253	2.21395E-09	1.01195E-08
DLD	-0.308174218	2.21507E-09	1.01223E-08
ZFHX4	-0.308137775	2.22521E-09	1.01663E-08
ANKH	-0.308114859	2.23161E-09	1.01932E-08
ZNF708	-0.308102771	2.23499E-09	1.02063E-08
PUM1	-0.307941404	2.28062E-09	1.04123E-08
TXNDC12	0.307932666	2.28312E-09	1.04213E-08
PHRF1	-0.307877903	2.29882E-09	1.04906E-08
PELI1	-0.30784774	2.30752E-09	1.05279E-08
HSF2BP	0.307791065	2.32394E-09	1.06004E-08
SLFN12	-0.307778141	2.32771E-09	1.06151E-08
SYT9	-0.307760999	2.3327E-09	1.06355E-08

TMEM33	-0.307754882	2.33449E-09	1.06412E-08
CDC42EP2	0.307732808	2.34095E-09	1.06682E-08
PDE1C	-0.307712576	2.34688E-09	1.06928E-08
NHS	-0.307677807	2.35711E-09	1.07348E-08
DGCR14	0.307677573	2.35718E-09	1.07348E-08
SMAD6	-0.307670579	2.35924E-09	1.0741E-08
LOC80054	0.307669265	2.35963E-09	1.0741E-08
LOC402377	-0.307634771	2.36983E-09	1.0785E-08
PCDHGA10	-0.30760206	2.37955E-09	1.08267E-08
DLGAP2	-0.307528001	2.40168E-09	1.09232E-08
CDC37L1	-0.307527497	2.40183E-09	1.09232E-08
APPBP2	-0.307509789	2.40716E-09	1.09449E-08
SIAE	-0.307473936	2.41797E-09	1.09915E-08
SPIRE2	0.307324414	2.46357E-09	1.11963E-08
GPR37	-0.30729163	2.47368E-09	1.12396E-08
RCCD1	0.307234107	2.49151E-09	1.13181E-08
MFN2	-0.306993691	2.56742E-09	1.16602E-08
PIF1	0.306986228	2.56981E-09	1.16684E-08
MYH10	-0.306886583	2.60195E-09	1.18117E-08
HAUS5	0.306876636	2.60518E-09	1.18236E-08
GRN	0.306867521	2.60814E-09	1.18344E-08
RGS18	-0.306835862	2.61846E-09	1.18785E-08
TACO1	0.306821715	2.62308E-09	1.18968E-08
CTRL	0.306817193	2.62456E-09	1.19008E-08
C22orf27	0.306807132	2.62786E-09	1.1913E-08
POM121L9P	-0.306781011	2.63643E-09	1.19491E-08
CCRN4L	-0.306759807	2.64341E-09	1.1978E-08
SHOC2	-0.306749126	2.64693E-09	1.19913E-08
WBP11P1	-0.306745274	2.6482E-09	1.19943E-08
HEMK1	0.30667009	2.67313E-09	1.21045E-08
CEP110	-0.306493303	2.73265E-09	1.23712E-08
CDK14	-0.306460783	2.74374E-09	1.24185E-08
RPS6KB2	0.306447508	2.74828E-09	1.24363E-08
SNX1	-0.306402862	2.7636E-09	1.25027E-08
CHEK2	0.306386539	2.76922E-09	1.25253E-08
PAMR1	-0.30631353	2.7945E-09	1.26368E-08
TAS2R14	-0.306310137	2.79568E-09	1.26393E-08
SPCS2	0.306263697	2.81188E-09	1.27096E-08
CP110	-0.30625563	2.8147E-09	1.27195E-08
MGC16275	0.306241079	2.8198E-09	1.27397E-08
RABGEF1	0.306199965	2.83426E-09	1.28021E-08
DHH	-0.306153243	2.85078E-09	1.28738E-08
AGAP11	-0.306126997	2.8601E-09	1.29129E-08
PGM5P2	-0.306101532	2.86917E-09	1.2951E-08
BAG5	-0.306096207	2.87107E-09	1.29566E-08
KIF21A	-0.306021021	2.89804E-09	1.30753E-08

RABGGTB	0.306001369	2.90513E-09	1.31043E-08
NOL11	0.305992945	2.90817E-09	1.31151E-08
LRRC55	-0.305888628	2.94612E-09	1.32832E-08
SLFN12L	-0.305806669	2.97626E-09	1.34161E-08
UBOX5	0.305749004	2.99766E-09	1.3508E-08
MSL2	-0.305748083	2.998E-09	1.3508E-08
VOPP1	0.305744401	2.99937E-09	1.35111E-08
LOC100286	0.305686493	3.02101E-09	1.36055E-08
CAPN12	0.305593809	3.05597E-09	1.37598E-08
EIF5AL1	0.30551172	3.08726E-09	1.38976E-08
C19orf22	0.305441392	3.11431E-09	1.40162E-08
PDE1A	-0.305409523	3.12665E-09	1.40685E-08
ELL3	0.305287948	3.17414E-09	1.4279E-08
LHX6	-0.30526852	3.18179E-09	1.43102E-08
PTPRF	-0.305230051	3.197E-09	1.43753E-08
FBXL4	-0.305134978	3.23488E-09	1.45424E-08
PEX3	-0.305099665	3.24907E-09	1.46028E-08
KIAA1462	-0.305097613	3.24989E-09	1.46033E-08
SPRED1	-0.305057393	3.26612E-09	1.46729E-08
TIMP3	-0.30502018	3.28121E-09	1.47373E-08
UBQLN1	-0.304952415	3.30886E-09	1.48582E-08
MGC23284	0.304945379	3.31174E-09	1.48678E-08
SLC40A1	-0.304928026	3.31887E-09	1.48964E-08
TECR	0.304914526	3.32442E-09	1.49179E-08
PPP1R13B	-0.304815266	3.36551E-09	1.50989E-08
TP53TG1	0.304713081	3.40833E-09	1.52876E-08
PCDHB10	-0.304659364	3.43105E-09	1.5386E-08
FAM126A	-0.30463413	3.44177E-09	1.54306E-08
EXO1	0.30458671	3.46201E-09	1.55179E-08
RYBP	-0.304568861	3.46966E-09	1.55487E-08
SLC25A43	-0.304563889	3.47179E-09	1.55547E-08
MITF	-0.304499104	3.49971E-09	1.56763E-08
PCNA	0.30448094	3.50757E-09	1.5708E-08
ZSCAN23	-0.30443734	3.52652E-09	1.57893E-08
PPP1CB	-0.304429956	3.52974E-09	1.58001E-08
SYTL5	-0.304422152	3.53315E-09	1.58118E-08
COPS3	0.30434386	3.56749E-09	1.59619E-08
IFI30	0.304337256	3.5704E-09	1.59714E-08
FUNDC2	0.304312566	3.5813E-09	1.60165E-08
NUP160	-0.304310354	3.58228E-09	1.60173E-08
GOSR1	-0.304226932	3.61938E-09	1.61795E-08
ZC3H14	-0.304209346	3.62724E-09	1.62111E-08
PROCA1	0.304135378	3.66052E-09	1.63561E-08
C1orf124	-0.304051221	3.69873E-09	1.65232E-08
PHKG2	0.304031158	3.7079E-09	1.65604E-08
C5orf39	0.303934194	3.75251E-09	1.67559E-08

EGR1	-0.30391334	3.76218E-09	1.67953E-08
SLC5A11	0.303900631	3.76808E-09	1.68179E-08
ENTPD5	-0.303878863	3.77821E-09	1.68593E-08
KIAA1217	-0.303842804	3.79504E-09	1.69306E-08
THAP10	-0.303805121	3.81271E-09	1.70056E-08
THOP1	0.303788494	3.82054E-09	1.70367E-08
TRABD	0.303766932	3.8307E-09	1.70782E-08
FAM26E	-0.303693705	3.86543E-09	1.72292E-08
ZNF268	-0.303603215	3.90876E-09	1.74184E-08
IFT27	0.303600828	3.90991E-09	1.74197E-08
FAM65B	-0.303595913	3.91228E-09	1.74263E-08
HADHB	-0.303529846	3.94424E-09	1.75626E-08
CX3CR1	-0.303529023	3.94464E-09	1.75626E-08
TOMM40L	0.303480134	3.96846E-09	1.76647E-08
SIK3	-0.303443823	3.98624E-09	1.77399E-08
DDOST	0.303401228	4.0072E-09	1.78292E-08
DPYD	-0.303293906	4.06047E-09	1.80622E-08
ITGBL1	-0.303205548	4.10485E-09	1.82555E-08
PLAGL1	-0.30309891	4.15903E-09	1.84923E-08
LRRTM2	-0.303096427	4.1603E-09	1.84938E-08
LZTS1	-0.303090877	4.16314E-09	1.85023E-08
KPNA6	-0.303083341	4.167E-09	1.85153E-08
AP1S1	0.30288432	4.27017E-09	1.89695E-08
RABEP1	-0.302880767	4.27203E-09	1.89736E-08
ADCY4	-0.30284262	4.2921E-09	1.90584E-08
PGAM4	-0.302820064	4.304E-09	1.9107E-08
KRT8	0.302812565	4.30797E-09	1.91204E-08
HSPA1B	0.302706774	4.36429E-09	1.93661E-08
BAI3	-0.302691218	4.37263E-09	1.93988E-08
ZNF426	-0.302685093	4.37592E-09	1.9409E-08
NVL	0.302610492	4.41617E-09	1.95832E-08
FAM83C	0.302563141	4.44191E-09	1.96929E-08
OGDH	-0.302435154	4.51219E-09	2.00001E-08
KIAA0892	-0.302423499	4.51865E-09	2.00242E-08
C3orf70	-0.302349664	4.55974E-09	2.02018E-08
LAMP2	-0.302332734	4.56921E-09	2.02393E-08
ANK3	-0.302311545	4.5811E-09	2.02849E-08
NPY5R	-0.302310729	4.58155E-09	2.02849E-08
IFI16	-0.302197741	4.64543E-09	2.05632E-08
GNB4	-0.302170442	4.661E-09	2.06275E-08
GRAPL	-0.302121675	4.68893E-09	2.07465E-08
MERTK	-0.302118577	4.69071E-09	2.07498E-08
NEK2	0.302100326	4.7012E-09	2.07916E-08
HINT2	0.302092637	4.70563E-09	2.08065E-08
ZNF500	-0.302067004	4.72043E-09	2.08673E-08
HEXDC	0.302061624	4.72354E-09	2.08764E-08

TMEM38B	-0.302011278	4.75275E-09	2.10009E-08
MKKS	0.301944114	4.79199E-09	2.11696E-08
C11orf17	0.301928089	4.8014E-09	2.12064E-08
SMURF1	-0.301855028	4.84452E-09	2.13922E-08
FAM163B	-0.301849717	4.84767E-09	2.14013E-08
CGGBP1	-0.301802833	4.87556E-09	2.15197E-08
ZACN	0.30175491	4.90422E-09	2.16414E-08
PIR	0.301740831	4.91268E-09	2.16739E-08
UCN	0.301734332	4.91658E-09	2.16863E-08
HBP1	-0.30170552	4.93394E-09	2.17581E-08
SOX6	-0.301644678	4.97078E-09	2.19157E-08
GLIS3	-0.301590651	5.00372E-09	2.20519E-08
TMED1	0.301590358	5.0039E-09	2.20519E-08
AHCYL1	-0.301567466	5.01792E-09	2.21088E-08
RRS1	0.301515342	5.04999E-09	2.22452E-08
BRIP1	-0.301427074	5.10475E-09	2.24815E-08
C6orf134	0.301405169	5.11843E-09	2.25367E-08
HSPA4	0.301386134	5.13035E-09	2.25842E-08
YY1	-0.301381243	5.13341E-09	2.25927E-08
SLC30A5	-0.301339762	5.15948E-09	2.27024E-08
KLHL4	-0.301277675	5.19875E-09	2.28701E-08
FBXO43	0.301251832	5.21518E-09	2.29373E-08
C15orf28	-0.301205503	5.24476E-09	2.30603E-08
FBXO46	0.301204405	5.24546E-09	2.30603E-08
OAZ3	0.30119769	5.24976E-09	2.30742E-08
ERGIC1	-0.301190137	5.2546E-09	2.30903E-08
SSB	0.301182981	5.25919E-09	2.31054E-08
PDZRN3	-0.301153241	5.27832E-09	2.31843E-08
GPR89B	-0.301133228	5.29122E-09	2.32359E-08
COX4NB	0.301053069	5.34323E-09	2.34591E-08
COX7B	0.301038867	5.35249E-09	2.34946E-08
ITGAE	0.300990129	5.38441E-09	2.36295E-08
PCDH7	-0.30084809	5.47847E-09	2.4037E-08
C6orf174	-0.300777088	5.52608E-09	2.42405E-08
C1orf122	0.300738631	5.55204E-09	2.4349E-08
C20orf152	0.300694126	5.58223E-09	2.4476E-08
ARF6	-0.300692298	5.58347E-09	2.44761E-08
KLHL7	-0.300650604	5.6119E-09	2.45953E-08
GUSBL1	-0.300638412	5.62024E-09	2.46264E-08
IRGQ	0.300635789	5.62204E-09	2.46289E-08
ZBTB8A	-0.30061055	5.63935E-09	2.46993E-08
ZNF567	-0.300572036	5.66586E-09	2.48099E-08
ANGPT4	-0.300466137	5.73938E-09	2.51263E-08
RASSF2	-0.300407693	5.78035E-09	2.53001E-08
SGPL1	-0.300403984	5.78296E-09	2.5306E-08
KCNE1	-0.300333293	5.83291E-09	2.5519E-08

RPN2	0.300293574	5.86116E-09	2.5637E-08
BACH1	-0.300237275	5.90144E-09	2.58074E-08
MRPL19	-0.300177301	5.94463E-09	2.59906E-08
SRPX	-0.300169189	5.9505E-09	2.60106E-08
ZNF254	-0.300105519	5.99673E-09	2.62069E-08
SUPT5H	0.300097833	6.00234E-09	2.62257E-08
SIKE1	-0.300093424	6.00556E-09	2.6234E-08
C19orf60	0.300044082	6.04168E-09	2.6386E-08
ARCN1	-0.300035897	6.04769E-09	2.64064E-08
NSMCE4A	0.300027097	6.05416E-09	2.64289E-08
RIC8B	-0.299995034	6.0778E-09	2.65263E-08
CD274	-0.299990626	6.08105E-09	2.65346E-08
MFAP3L	-0.299960717	6.10319E-09	2.66254E-08
FDPS	0.299948459	6.11228E-09	2.66592E-08
PSMA2	0.299908227	6.14223E-09	2.6784E-08
ACVR1	-0.299830859	6.20021E-09	2.70309E-08
DUSP22	0.299783397	6.23604E-09	2.71812E-08
LPP	-0.299758187	6.25516E-09	2.72585E-08
SLC25A1	0.299749164	6.26201E-09	2.72824E-08
SRGAP1	-0.299746802	6.26381E-09	2.72843E-08
HTR7	-0.299735842	6.27215E-09	2.73146E-08
ZNF718	-0.299647239	6.33995E-09	2.76039E-08
PDE4DIP	-0.299618298	6.36226E-09	2.76949E-08
JUND	0.299613207	6.36619E-09	2.7706E-08
DNAJC2	0.299567884	6.40129E-09	2.78527E-08
KLHDC8B	0.299429797	6.50939E-09	2.83168E-08
PI4KA	-0.299379685	6.54905E-09	2.84832E-08
TRIM62	-0.299358108	6.5662E-09	2.85515E-08
NIPSNAP3A	-0.299310921	6.60386E-09	2.8709E-08
SULT1C4	-0.299242154	6.65912E-09	2.89429E-08
CTAGE5	-0.299198668	6.69429E-09	2.90894E-08
ATF1	-0.299186409	6.70424E-09	2.91208E-08
LDB3	-0.299186167	6.70443E-09	2.91208E-08
KIAA0922	-0.299160851	6.72502E-09	2.92038E-08
CHRM2	-0.299147534	6.73588E-09	2.92446E-08
RNF114	0.299012192	6.84717E-09	2.97213E-08
DCLK1	-0.298931428	6.91442E-09	3.00067E-08
LOC648740	-0.298885129	6.95327E-09	3.01687E-08
ZMYM1	-0.298821224	7.00723E-09	3.03962E-08
GALE	0.298818122	7.00986E-09	3.0401E-08
CYP2C8	-0.29878428	7.03861E-09	3.0519E-08
PLCXD3	-0.298739601	7.07675E-09	3.06777E-08
CDK2AP2	0.298727884	7.08678E-09	3.07145E-08
UTRN	-0.298666394	7.13966E-09	3.0937E-08
TAGAP	-0.298607805	7.1904E-09	3.11501E-08
ZNF394	0.298595134	7.20142E-09	3.1191E-08

MESTIT1	-0.298590867	7.20514E-09	3.12003E-08
SNHG1	0.298571925	7.22165E-09	3.1265E-08
KIAA0649	-0.298521375	7.26589E-09	3.14497E-08
UBD	0.298423383	7.35241E-09	3.18173E-08
IFITM3	0.298378171	7.39267E-09	3.19846E-08
RBM33	-0.298374361	7.39607E-09	3.19923E-08
MAPKAPK5	0.298367045	7.4026E-09	3.20137E-08
FAM13A	-0.298307452	7.45606E-09	3.22378E-08
CPNE1	0.29828563	7.47572E-09	3.23158E-08
CXCL17	0.298246995	7.51067E-09	3.24598E-08
GNA13	-0.298221369	7.53393E-09	3.25533E-08
ZMYM4	-0.29816333	7.58688E-09	3.2775E-08
AP1M2	0.298085393	7.65855E-09	3.30774E-08
ZNF346	0.298081411	7.66223E-09	3.30861E-08
ZNF660	-0.298057756	7.68412E-09	3.31735E-08
DNAJC14	-0.298021727	7.71758E-09	3.33107E-08
ITPKB	-0.297982296	7.75436E-09	3.34622E-08
EIF5	-0.297959069	7.77611E-09	3.35488E-08
ELTD1	-0.297938633	7.79529E-09	3.36243E-08
PAFAH1B1	-0.297926035	7.80714E-09	3.36681E-08
ARMC8	-0.297900624	7.83109E-09	3.3764E-08
JMY	-0.297843604	7.88509E-09	3.39895E-08
ALDOA	0.297839817	7.88869E-09	3.39977E-08
ZNF799	-0.297708239	8.01474E-09	3.45334E-08
B4GALT1	-0.297613812	8.10639E-09	3.49208E-08
ACP6	0.297551398	8.16753E-09	3.51766E-08
ALKBH8	-0.297547024	8.17183E-09	3.51875E-08
GPC3	0.297519628	8.19882E-09	3.52961E-08
PPP2R2A	-0.297504909	8.21336E-09	3.5351E-08
DUS3L	0.297496141	8.22203E-09	3.53807E-08
C4orf52	0.297491965	8.22617E-09	3.53908E-08
PPARA	-0.297486016	8.23206E-09	3.54085E-08
HIST1H4J	0.297431278	8.28646E-09	3.56348E-08
CST3	0.297375495	8.34225E-09	3.5867E-08
ST20	0.297352677	8.36518E-09	3.59578E-08
KIAA0556	-0.297290136	8.42834E-09	3.62215E-08
ZNF592	-0.297248904	8.47023E-09	3.63937E-08
SKP1	0.297236115	8.48326E-09	3.64418E-08
PKD1L3	-0.297215587	8.50423E-09	3.6524E-08
FAM38A	-0.297180126	8.54055E-09	3.66721E-08
CDK1	0.297163794	8.55734E-09	3.67362E-08
RALGAPB	-0.297153035	8.56841E-09	3.67734E-08
UMPS	0.297151804	8.56968E-09	3.67734E-08
ANKRD11	-0.29711588	8.60676E-09	3.69245E-08
PABPC1	0.297079797	8.64415E-09	3.7077E-08
TUBG1	0.296982617	8.74566E-09	3.75043E-08

DIXDC1	-0.296916626	8.81525E-09	3.77945E-08
TRIM65	0.296907827	8.82457E-09	3.78264E-08
DEFB132	-0.296892383	8.84095E-09	3.78884E-08
ARHGEF11	-0.296771624	8.97004E-09	3.84334E-08
BDNFOS	-0.296763584	8.9787E-09	3.84622E-08
SPCS3	-0.296745039	8.9987E-09	3.85396E-08
KCNK7	0.296695797	9.05203E-09	3.87597E-08
ALG1L	0.296670284	9.07978E-09	3.88701E-08
DIDO1	-0.296637284	9.11579E-09	3.90159E-08
AKIRIN1	-0.29661779	9.13713E-09	3.90988E-08
LOC646214	-0.296568884	9.19088E-09	3.93204E-08
TOMM20L	0.29648711	9.28144E-09	3.96993E-08
BTN2A3	-0.296470627	9.2998E-09	3.97625E-08
MEF2C	-0.296470251	9.30022E-09	3.97625E-08
MRPL18	0.296468141	9.30257E-09	3.97641E-08
CHD8	-0.296448998	9.32394E-09	3.98469E-08
KIAA0232	-0.29643829	9.33592E-09	3.98895E-08
GSTCD	-0.296408343	9.36949E-09	4.00243E-08
DBR1	-0.296399093	9.37988E-09	4.00601E-08
PITPNM3	-0.296388694	9.39158E-09	4.01015E-08
SFRS2B	-0.296372833	9.40944E-09	4.01691E-08
ANXA3	-0.296369313	9.41341E-09	4.01775E-08
C19orf18	0.296341076	9.44532E-09	4.0305E-08
POLD4	0.29633548	9.45165E-09	4.03234E-08
LRRC4	-0.2963218	9.46716E-09	4.03785E-08
CCDC123	0.296320508	9.46862E-09	4.03785E-08
CDKL1	-0.296203269	9.60252E-09	4.09408E-08
HIST1H3C	0.296177644	9.63203E-09	4.10578E-08
LOC728875	0.296162141	9.64993E-09	4.1123E-08
IL13RA1	-0.296160816	9.65146E-09	4.1123E-08
FAM66D	0.296063495	9.76456E-09	4.15821E-08
SEMA4B	-0.296062943	9.76521E-09	4.15821E-08
SLC9A3R1	0.296062708	9.76548E-09	4.15821E-08
SULF2	-0.296030591	9.80309E-09	4.17333E-08
CBY1	0.296011893	9.82505E-09	4.18179E-08
RETSAT	-0.295941309	9.90838E-09	4.21635E-08
CYB5R4	-0.29590169	9.95545E-09	4.23548E-08
KCMF1	0.295866501	9.99744E-09	4.25243E-08
TBCK	-0.295844747	1.00235E-08	4.2626E-08
XPC	-0.295774729	1.01077E-08	4.29752E-08
C14orf104	-0.295707715	1.0189E-08	4.33116E-08
AEBP2	-0.29568082	1.02219E-08	4.34418E-08
HIST1H2AH	0.295672791	1.02317E-08	4.34742E-08
PNKP	0.295654407	1.02542E-08	4.35605E-08
DERL2	0.295594712	1.03276E-08	4.3863E-08
SIPA1L3	-0.295585057	1.03395E-08	4.39042E-08

ZFYVE26	-0.295539293	1.03962E-08	4.41355E-08
THOC3	0.295533887	1.04029E-08	4.41546E-08
ELK4	-0.295525661	1.04131E-08	4.41886E-08
ROS1	-0.295489624	1.0458E-08	4.43697E-08
SLC6A1	-0.295482638	1.04668E-08	4.43973E-08
ASPA	-0.295366316	1.06131E-08	4.50086E-08
RASSF6	-0.295331052	1.06579E-08	4.51889E-08
TIGD3	0.295318913	1.06733E-08	4.52447E-08
DZIP1	-0.295289141	1.07113E-08	4.53961E-08
LRRC4C	-0.295244261	1.07689E-08	4.56302E-08
TP53BP1	-0.295240003	1.07743E-08	4.56436E-08
SEC11A	0.295235762	1.07798E-08	4.5657E-08
WAPAL	-0.29521762	1.08031E-08	4.57462E-08
RPAIN	0.295152451	1.08874E-08	4.60934E-08
ZNF558	-0.295140957	1.09024E-08	4.61463E-08
JAG1	-0.295139266	1.09046E-08	4.61463E-08
ENG	-0.295049744	1.10216E-08	4.66317E-08
PCDHGB8P	-0.29503539	1.10405E-08	4.67016E-08
FAM134C	0.295030702	1.10467E-08	4.67178E-08
UBR4	-0.294967383	1.11303E-08	4.70618E-08
FHL2	-0.294932307	1.1177E-08	4.72489E-08
FLJ13197	-0.294871472	1.12583E-08	4.75825E-08
C2orf29	0.294812049	1.13383E-08	4.79104E-08
SDK1	-0.294788121	1.13706E-08	4.80369E-08
NQO2	0.294763757	1.14037E-08	4.81663E-08
DNAJB9	-0.294752382	1.14191E-08	4.82213E-08
RPL36A	0.294610146	1.16141E-08	4.90345E-08
DNAJC3	-0.294598251	1.16306E-08	4.90935E-08
MAD1L1	0.294576688	1.16605E-08	4.92092E-08
MYH11	-0.294553076	1.16933E-08	4.93373E-08
HIST1H4C	0.294527821	1.17285E-08	4.94753E-08
IPO7	-0.294385577	1.19286E-08	5.03088E-08
C5orf25	-0.294379231	1.19376E-08	5.03362E-08
EXOSC3	0.294338792	1.19952E-08	5.05681E-08
ASAH2	-0.294309145	1.20375E-08	5.07359E-08
TMEM85	0.294295676	1.20568E-08	5.08065E-08
VGLL4	-0.294252995	1.21181E-08	5.10541E-08
DHX15	-0.294192247	1.22059E-08	5.14131E-08
APPL1	-0.29417095	1.22369E-08	5.15325E-08
CHST13	0.29406295	1.23949E-08	5.21869E-08
INTS8	0.294058061	1.24021E-08	5.22061E-08
PKD1	-0.293880232	1.26667E-08	5.3304E-08
CCDC91	-0.293879214	1.26682E-08	5.3304E-08
FAM107A	-0.293876469	1.26724E-08	5.33101E-08
GYPE	-0.293862976	1.26927E-08	5.33843E-08
MAPRE2	-0.293664222	1.29955E-08	5.46464E-08

ZNF823	-0.293652451	1.30137E-08	5.47112E-08
PLK1	0.293649306	1.30185E-08	5.47201E-08
EDNRA	-0.293635654	1.30396E-08	5.47972E-08
ATN1	-0.293624041	1.30576E-08	5.48611E-08
MYO1D	-0.293608982	1.30809E-08	5.49475E-08
OR2A4	-0.29360508	1.3087E-08	5.49614E-08
TUBB2A	0.293472828	1.32937E-08	5.5818E-08
C8orf38	0.293382986	1.3436E-08	5.64034E-08
CEP120	-0.293352196	1.34851E-08	5.65975E-08
SEC62	-0.293298224	1.35716E-08	5.69383E-08
PPP1R2P3	-0.29329796	1.3572E-08	5.69383E-08
MAP2K4	-0.293196524	1.3736E-08	5.76141E-08
C1orf25	-0.293183837	1.37566E-08	5.76885E-08
HEPH	-0.293175296	1.37705E-08	5.77347E-08
ZNF596	-0.292983263	1.4087E-08	5.90491E-08
NR4A3	-0.292974299	1.41019E-08	5.90993E-08
BAT5	0.292911791	1.42066E-08	5.95253E-08
PGAP1	-0.292887374	1.42476E-08	5.96848E-08
DLEC1	-0.29282434	1.43542E-08	6.01186E-08
ZC3H11A	-0.292806263	1.43849E-08	6.02345E-08
CHCHD10	0.29280421	1.43884E-08	6.02365E-08
USP35	-0.292780969	1.4428E-08	6.03801E-08
GABARAPL3	-0.292780498	1.44288E-08	6.03801E-08
C1QL3	-0.29270011	1.45665E-08	6.09436E-08
ZNF280C	-0.292688132	1.45871E-08	6.10171E-08
SLC35E2	-0.292629037	1.46893E-08	6.14316E-08
AIM1	-0.292586811	1.47627E-08	6.17258E-08
TMEM189	0.292473608	1.49614E-08	6.25432E-08
SHANK1	-0.292424965	1.50475E-08	6.28902E-08
DNM3	-0.292404641	1.50837E-08	6.3028E-08
ATL2	-0.292377167	1.51326E-08	6.32194E-08
ITFG1	-0.292359647	1.5164E-08	6.33369E-08
AGTPBP1	-0.292292978	1.52837E-08	6.38237E-08
COQ10B	-0.292255499	1.53514E-08	6.4093E-08
HUWE1	-0.292216132	1.54229E-08	6.43778E-08
STXBP5	-0.292201769	1.5449E-08	6.44734E-08
NASP	0.292199419	1.54533E-08	6.44777E-08
SHF	0.29210122	1.56332E-08	6.52149E-08
FCF1	-0.292029903	1.57652E-08	6.57516E-08
GP6	-0.292021293	1.57812E-08	6.58045E-08
C19orf42	0.292018253	1.57869E-08	6.58143E-08
MED11	0.291911404	1.59868E-08	6.66265E-08
GBE1	-0.291910592	1.59884E-08	6.66265E-08
AMFR	-0.291907295	1.59946E-08	6.66385E-08
LOC400927	-0.291894398	1.60189E-08	6.67258E-08
MTRR	-0.29188665	1.60335E-08	6.67728E-08

MSH5	0.291875743	1.60541E-08	6.68446E-08
C1orf97	0.291841902	1.61182E-08	6.70975E-08
LY75	-0.291820197	1.61595E-08	6.72552E-08
SLC29A3	0.291810381	1.61782E-08	6.73189E-08
SNTA1	0.291796342	1.62049E-08	6.74162E-08
ZNF267	-0.2917816	1.62331E-08	6.75192E-08
GFOD2	-0.291708155	1.6374E-08	6.80912E-08
FAR1	-0.291658013	1.64709E-08	6.84799E-08
RCSD1	-0.291644945	1.64963E-08	6.8571E-08
CELSR2	-0.291622772	1.65394E-08	6.87357E-08
BHLHE22	-0.291543104	1.66951E-08	6.93685E-08
MAPKSP1	-0.29151015	1.67599E-08	6.96233E-08
BRCA2	-0.291490314	1.67991E-08	6.97714E-08
SLC25A24	-0.291465632	1.68479E-08	6.99596E-08
GEMIN8P4	0.291462689	1.68537E-08	6.99692E-08
SLC38A2	-0.291442466	1.68939E-08	7.01212E-08
DUSP18	-0.291434086	1.69105E-08	7.01757E-08
PSMD3	0.291379731	1.70189E-08	7.06108E-08
HTR1B	-0.291344585	1.70894E-08	7.08883E-08
NCRNA0020	0.291302776	1.71735E-08	7.12227E-08
IFT81	-0.291290304	1.71987E-08	7.13123E-08
SPIN3	-0.291216767	1.7348E-08	7.19161E-08
SH3GLB1	-0.291168851	1.74459E-08	7.23069E-08
DACH1	-0.29100628	1.77821E-08	7.36851E-08
ZNF24	-0.290949103	1.79018E-08	7.41657E-08
RP1	-0.290903976	1.79969E-08	7.4544E-08
FOXH1	0.290875264	1.80576E-08	7.478E-08
GK5	-0.290821812	1.81712E-08	7.52347E-08
TOE1	0.290792828	1.8233E-08	7.54752E-08
ROBO4	-0.290783337	1.82534E-08	7.55435E-08
SAMD10	0.290774378	1.82725E-08	7.56072E-08
TNC	-0.290710263	1.84104E-08	7.61586E-08
SEMA3C	-0.290708865	1.84134E-08	7.61586E-08
RNF152	-0.290648219	1.85448E-08	7.66861E-08
PARVA	-0.290643534	1.8555E-08	7.67123E-08
METTL6	0.290591199	1.86692E-08	7.71682E-08
IFIT2	-0.290584902	1.86829E-08	7.72091E-08
KIT	-0.290571054	1.87133E-08	7.73185E-08
C6orf62	0.290559599	1.87384E-08	7.74063E-08
C4orf10	0.290476994	1.89206E-08	7.81428E-08
STXBP6	0.290452306	1.89754E-08	7.83529E-08
C4orf31	-0.290445781	1.89899E-08	7.83965E-08
KIAA0040	-0.290425666	1.90347E-08	7.85651E-08
C15orf24	0.290416129	1.9056E-08	7.86366E-08
FN1	-0.290340617	1.92252E-08	7.93186E-08
UGP2	-0.290327242	1.92554E-08	7.94131E-08

DUSP9	0.290326906	1.92561E-08	7.94131E-08
C15orf42	0.290273133	1.93777E-08	7.9898E-08
DSN1	0.290262235	1.94024E-08	7.99834E-08
CCNF	0.290237552	1.94586E-08	8.01982E-08
HERC2P2	-0.290109289	1.97528E-08	8.13939E-08
PPARGC1A	-0.290103528	1.97661E-08	8.14319E-08
DRD1	-0.290086129	1.98064E-08	8.15809E-08
GLRX2	0.290018817	1.99629E-08	8.22086E-08
C1orf52	0.290015055	1.99717E-08	8.22278E-08
HIRA	-0.289932086	2.01663E-08	8.30121E-08
FAM160A2	-0.289914531	2.02077E-08	8.31654E-08
HSPA13	-0.289887655	2.02713E-08	8.34098E-08
PTPN12	-0.289849653	2.03615E-08	8.37637E-08
PROSC	-0.289766968	2.05592E-08	8.45594E-08
NARG2	-0.289709228	2.06983E-08	8.5114E-08
CRY2	-0.289693263	2.07369E-08	8.52552E-08
CXCR7	-0.289578227	2.10173E-08	8.63902E-08
ZNF107	-0.289544353	2.11006E-08	8.6712E-08
CYTSA	-0.289542828	2.11044E-08	8.6712E-08
CLCC1	-0.289484051	2.12496E-08	8.72909E-08
C11orf42	-0.289437188	2.13661E-08	8.77513E-08
SH3BGRL3	0.289397963	2.14641E-08	8.81356E-08
RHOT1	-0.289386132	2.14937E-08	8.82391E-08
TMEM66	-0.289352762	2.15776E-08	8.8565E-08
LGTN	0.289290108	2.17358E-08	8.9196E-08
JAM2	-0.289287332	2.17428E-08	8.92065E-08
NKIRAS2	0.289246761	2.18459E-08	8.9611E-08
ME2	-0.289225779	2.18994E-08	8.9812E-08
LOC222699	0.28920249	2.19589E-08	9.00376E-08
CAMK2D	-0.289032857	2.23973E-08	9.1816E-08
YAF2	-0.288976984	2.25435E-08	9.23964E-08
PDGFB	-0.288912944	2.27122E-08	9.30688E-08
MAP9	-0.288894099	2.27621E-08	9.3254E-08
ATP8A1	-0.288855784	2.28639E-08	9.36516E-08
TEX19	0.288844965	2.28927E-08	9.37503E-08
BAZ2A	-0.288781575	2.30622E-08	9.44251E-08
KIAA1107	-0.288717187	2.32356E-08	9.51156E-08
TTC4	0.288708657	2.32587E-08	9.51905E-08
RG9MTD2	-0.288592479	2.35751E-08	9.64656E-08
TRIP4	0.288570191	2.36363E-08	9.66961E-08
MAP3K12	-0.288516565	2.37841E-08	9.72775E-08
GATA6	-0.288515093	2.37882E-08	9.72775E-08
ZNF193	0.288426866	2.40334E-08	9.826E-08
TRPV3	-0.288413181	2.40716E-08	9.83962E-08
TP53RK	0.288304016	2.43788E-08	9.96315E-08
RGL2	0.288280633	2.44451E-08	9.9882E-08

TFAP4	0.288254265	2.45201E-08	1.00168E-07
SLC16A2	-0.288162089	2.47839E-08	1.01225E-07
TAS2R20	0.288102086	2.49572E-08	1.01911E-07
BRCC3	-0.288035958	2.51494E-08	1.02675E-07
LRRC34	-0.287998012	2.52604E-08	1.03107E-07
ZNF514	-0.287990126	2.52835E-08	1.03164E-07
THAP1	-0.287989723	2.52847E-08	1.03164E-07
DOCK8	-0.28796102	2.5369E-08	1.03487E-07
DHX40	-0.287936305	2.54418E-08	1.03763E-07
EMR4P	-0.287903982	2.55374E-08	1.04131E-07
SFRS12IP1	0.287890443	2.55775E-08	1.04273E-07
C9orf47	-0.28781578	2.57999E-08	1.05159E-07
TOB1	-0.287781385	2.5903E-08	1.05557E-07
KDM5C	-0.287756552	2.59776E-08	1.0584E-07
ARID3C	-0.287740648	2.60256E-08	1.06005E-07
N4BP1	-0.287739544	2.60289E-08	1.06005E-07
HRH4	-0.287640585	2.63291E-08	1.07206E-07
RAB6C	-0.287627938	2.63677E-08	1.07341E-07
C5orf34	0.287624125	2.63793E-08	1.07366E-07
RBM26	-0.28750605	2.67425E-08	1.08823E-07
CSRNP2	-0.287460146	2.6885E-08	1.0938E-07
KCNS3	-0.287428136	2.69848E-08	1.09764E-07
C3orf36	-0.287421726	2.70049E-08	1.09823E-07
PLCG2	-0.287343693	2.72499E-08	1.10796E-07
PTPN3	-0.287261676	2.75097E-08	1.1183E-07
HAUS1	0.287216174	2.76548E-08	1.12397E-07
CITED2	-0.287182001	2.77644E-08	1.12819E-07
RRM2	0.287096862	2.8039E-08	1.13912E-07
APEX1	0.287089878	2.80617E-08	1.13974E-07
PSIMCT-1	-0.28708864	2.80657E-08	1.13974E-07
PSD4	-0.28708218	2.80867E-08	1.14036E-07
AZI2	-0.287041264	2.82198E-08	1.14553E-07
NFIB	-0.28702528	2.8272E-08	1.14741E-07
TNXB	-0.286995092	2.83708E-08	1.15119E-07
C3orf1	0.286822528	2.8942E-08	1.17413E-07
C17orf42	0.286816246	2.8963E-08	1.17474E-07
FAM65A	-0.286776789	2.90953E-08	1.17987E-07
C1orf105	0.28666771	2.9464E-08	1.19457E-07
LOC100128	0.286496298	3.00524E-08	1.21819E-07
RNF150	-0.28649104	3.00707E-08	1.21868E-07
CRTAC1	-0.286486995	3.00847E-08	1.219E-07
FAM183A	0.286418071	3.03248E-08	1.22847E-07
FN3KRP	0.286401466	3.03829E-08	1.23058E-07
ZNF121	-0.286388856	3.04271E-08	1.23212E-07
VWA3B	-0.286381571	3.04526E-08	1.2329E-07
LENG9	0.286347033	3.05741E-08	1.23757E-07

ATF6	-0.286336451	3.06114E-08	1.23883E-07
PAXIP1	-0.286303208	3.0729E-08	1.24333E-07
ZNF233	0.286298804	3.07446E-08	1.24371E-07
KIF4A	0.286283017	3.08005E-08	1.24572E-07
DIS3	-0.286200566	3.10945E-08	1.25736E-07
SMG5	0.286188505	3.11378E-08	1.25872E-07
CRTC2	0.286187621	3.11409E-08	1.25872E-07
FLJ12825	0.286185714	3.11478E-08	1.25874E-07
SYT11	-0.286138998	3.13158E-08	1.26528E-07
KIAA0391	-0.286067676	3.15741E-08	1.27545E-07
DNM2	-0.28603317	3.16998E-08	1.28027E-07
SPERT	0.286021104	3.17439E-08	1.28179E-07
TBC1D19	-0.28599356	3.18447E-08	1.2856E-07
KCNAB2	-0.285971302	3.19263E-08	1.28864E-07
RPGR	-0.285929883	3.20789E-08	1.29453E-07
LOC728855	0.285926828	3.20902E-08	1.29472E-07
HSPD1	0.285857811	3.2346E-08	1.30478E-07
MKS1	0.285805241	3.25422E-08	1.31243E-07
DPH1	-0.28575246	3.27404E-08	1.32015E-07
RBL1	-0.285731964	3.28176E-08	1.323E-07
CSRNP1	-0.285693292	3.29639E-08	1.32863E-07
TTN	-0.285679936	3.30145E-08	1.3304E-07
PLS1	-0.285678	3.30219E-08	1.33043E-07
C3orf47	0.28567054	3.30502E-08	1.3313E-07
SPON1	-0.28556743	3.34442E-08	1.3469E-07
ZSWIM3	0.285524694	3.36089E-08	1.35326E-07
ZNF33A	-0.285482533	3.37721E-08	1.35955E-07
CT45A1	0.28547869	3.3787E-08	1.35988E-07
SMC3	-0.285445219	3.39171E-08	1.36484E-07
ZBTB16	-0.285441247	3.39326E-08	1.36519E-07
EFNB3	-0.28541707	3.4027E-08	1.36871E-07
NAA35	-0.285382246	3.41633E-08	1.37391E-07
KDM4B	-0.285371572	3.42052E-08	1.37532E-07
RNPEPL1	0.285357355	3.42611E-08	1.37729E-07
RECK	-0.285343671	3.4315E-08	1.37918E-07
CABP7	0.285338843	3.4334E-08	1.37966E-07
POLR2J4	0.285332827	3.43577E-08	1.38034E-07
RANGAP1	0.285312848	3.44366E-08	1.38323E-07
TRMT12	0.285281432	3.4561E-08	1.38795E-07
SOCS2	-0.285274112	3.45901E-08	1.38883E-07
COBRA1	0.285250826	3.46827E-08	1.39227E-07
MUSTN1	0.285216646	3.4819E-08	1.39746E-07
GPR141	-0.285163226	3.50331E-08	1.40577E-07
FAM193A	-0.285161279	3.50409E-08	1.4058E-07
SH2D1B	-0.285159521	3.5048E-08	1.4058E-07
AMZ2	0.285131261	3.51618E-08	1.41008E-07

C14orf109	0.285106641	3.52612E-08	1.41378E-07
ATXN7L1	-0.285090486	3.53266E-08	1.41612E-07
SPIN4	-0.285011922	3.56463E-08	1.42865E-07
RNF13	-0.284999847	3.56957E-08	1.43034E-07
RAB22A	-0.284989711	3.57372E-08	1.43161E-07
ST6GALNAC	-0.284988611	3.57417E-08	1.43161E-07
KLHDC5	-0.284984151	3.576E-08	1.43206E-07
PWWP2A	-0.284964491	3.58407E-08	1.435E-07
PLN	-0.284941115	3.59369E-08	1.43856E-07
ZNF821	0.284925258	3.60023E-08	1.44089E-07
IVD	-0.284809888	3.64814E-08	1.45977E-07
ABCE1	-0.284806385	3.64961E-08	1.46006E-07
TPPP	-0.284739375	3.67773E-08	1.47102E-07
GTF2IRD2P1	-0.284729752	3.68179E-08	1.47235E-07
RTN4IP1	0.284701774	3.69361E-08	1.47677E-07
TRAF3IP1	-0.284666351	3.70862E-08	1.48248E-07
GLI2	-0.284661985	3.71047E-08	1.48292E-07
FIBIN	-0.284657427	3.71241E-08	1.4834E-07
SULT1B1	-0.284609566	3.73281E-08	1.49108E-07
RNF219	-0.284608842	3.73312E-08	1.49108E-07
ZNF169	-0.284604915	3.7348E-08	1.49145E-07
SERPINB9	-0.284600105	3.73686E-08	1.49197E-07
PIK3C2B	-0.284575809	3.74726E-08	1.49583E-07
CXCL12	-0.284565424	3.75172E-08	1.49731E-07
CCDC55	-0.28453202	3.76609E-08	1.50274E-07
UROS	0.284512832	3.77437E-08	1.50574E-07
ZNF774	-0.284497405	3.78104E-08	1.5081E-07
WBSCR16	0.284467775	3.79388E-08	1.51278E-07
CYB5R1	0.284466812	3.7943E-08	1.51278E-07
NR1H3	0.284412168	3.8181E-08	1.52197E-07
GDF2	-0.284409312	3.81934E-08	1.52216E-07
GRINA	0.284389604	3.82796E-08	1.52529E-07
ANAPC5	0.284386734	3.82922E-08	1.52544E-07
IRF2BP1	0.284385216	3.82989E-08	1.52544E-07
PCDHB13	-0.284317555	3.85964E-08	1.53671E-07
HDAC5	0.284317349	3.85973E-08	1.53671E-07
RPL13AP6	0.284311627	3.86225E-08	1.53741E-07
ZFPM1	-0.284306031	3.86473E-08	1.53809E-07
SLITRK6	-0.284203966	3.91009E-08	1.55583E-07
ANO3	-0.284184948	3.9186E-08	1.55891E-07
NLRP14	-0.284118043	3.94867E-08	1.57049E-07
CELF2	-0.284116653	3.9493E-08	1.57049E-07
C8orf79	-0.284102258	3.9558E-08	1.57261E-07
TXNDC9	0.284101363	3.9562E-08	1.57261E-07
RPS26P11	0.284011362	3.99709E-08	1.58855E-07
TNNC1	0.283988808	4.0074E-08	1.59233E-07

LDOC1L	-0.283889989	4.05287E-08	1.61007E-07
TNPO1	-0.283887712	4.05393E-08	1.61017E-07
DACT1	-0.28388185	4.05664E-08	1.61093E-07
EXOC3	0.283871583	4.0614E-08	1.61249E-07
CXorf61	0.283795057	4.09703E-08	1.62632E-07
CITED4	0.283780613	4.10379E-08	1.62867E-07
GORAB	-0.283720431	4.13206E-08	1.63957E-07
NAAA	-0.283705121	4.13929E-08	1.64211E-07
LYPLA2P1	0.283700684	4.14138E-08	1.64261E-07
PDGFD	-0.28364695	4.16684E-08	1.65238E-07
LOC440944	0.283540832	4.21757E-08	1.67217E-07
SEBOX	-0.283481838	4.24603E-08	1.68311E-07
PCDHGA1	-0.283439984	4.26633E-08	1.69082E-07
TOM1L1	-0.283405984	4.28289E-08	1.69705E-07
KCNJ2	-0.283391341	4.29004E-08	1.69955E-07
AURKA	0.283369684	4.30064E-08	1.70341E-07
ADAMTS7	-0.283362525	4.30415E-08	1.70446E-07
KIAA1704	-0.283324624	4.32277E-08	1.71149E-07
LOC613037	-0.283267851	4.35082E-08	1.72225E-07
TBC1D3C	0.283199656	4.38473E-08	1.73533E-07
SP2	-0.283165994	4.40157E-08	1.74165E-07
ZDBF2	-0.283144124	4.41254E-08	1.74564E-07
DCAF10	-0.283109862	4.42978E-08	1.75211E-07
FMR1	-0.283108162	4.43064E-08	1.75211E-07
PSMB10	0.283066381	4.45176E-08	1.76011E-07
NUFIP2	-0.282933144	4.51975E-08	1.78664E-07
TIMM22	0.282892926	4.54047E-08	1.79447E-07
PRR16	-0.282827211	4.57452E-08	1.80757E-07
ANXA11	0.282823908	4.57624E-08	1.80789E-07
UBE2CBP	0.282815859	4.58043E-08	1.80919E-07
CHST3	-0.28279477	4.59142E-08	1.81317E-07
C1orf54	0.282787744	4.59509E-08	1.81426E-07
MAP7	-0.282664785	4.65975E-08	1.83942E-07
SH2D3C	-0.28265647	4.66415E-08	1.8408E-07
PIGC	0.282644055	4.67073E-08	1.84303E-07
RNMT	-0.282549495	4.72117E-08	1.86256E-07
C4orf34	-0.282532664	4.7302E-08	1.86575E-07
NPY1R	-0.28252145	4.73623E-08	1.86776E-07
TOMM34	0.282465969	4.76616E-08	1.87919E-07
TRIM66	-0.282431838	4.78466E-08	1.88611E-07
ASB8	-0.28235558	4.82625E-08	1.90213E-07
EPHA2	-0.282268341	4.87425E-08	1.92067E-07
GDF6	-0.282234318	4.8931E-08	1.92741E-07
POLD2	0.282233959	4.8933E-08	1.92741E-07
GJA5	-0.282186557	4.91968E-08	1.93742E-07
MGLL	-0.282135501	4.94824E-08	1.94828E-07

THSD7B	-0.282095995	4.97045E-08	1.95664E-07
LOC643677	-0.282065079	4.9879E-08	1.96312E-07
KCNN3	-0.282055357	4.9934E-08	1.9649E-07
ANO7	0.281941116	5.05846E-08	1.99011E-07
HAND2	-0.281892258	5.08653E-08	2.00076E-07
ZC3HAV1L	-0.281883655	5.09149E-08	2.00231E-07
TLR1	-0.281860485	5.10487E-08	2.00718E-07
SMPD3	-0.281830191	5.12241E-08	2.01368E-07
OSGEP	0.281776741	5.15351E-08	2.0255E-07
VEGFC	-0.281772753	5.15584E-08	2.02602E-07
TOM1	0.281749954	5.16916E-08	2.03085E-07
BICC1	-0.281729967	5.18087E-08	2.03505E-07
PDCD2	0.281673491	5.21409E-08	2.0477E-07
FUT10	-0.281643256	5.23196E-08	2.05431E-07
LYVE1	-0.281594799	5.26073E-08	2.0652E-07
TMTC1	-0.281572022	5.2743E-08	2.07012E-07
ARL6IP1	-0.281569166	5.276E-08	2.07038E-07
TMEM188	-0.281530683	5.29902E-08	2.07893E-07
GJB5	-0.281529239	5.29989E-08	2.07893E-07
ZXDB	-0.281470574	5.33517E-08	2.09236E-07
PDXDC2	-0.281368402	5.39715E-08	2.11625E-07
CHST9	-0.281337209	5.41621E-08	2.1233E-07
KTN1	-0.281301719	5.43798E-08	2.13142E-07
SC65	0.281235552	5.47879E-08	2.14699E-07
CALM3	0.281208038	5.49584E-08	2.15325E-07
LENG8	-0.281203968	5.49837E-08	2.15382E-07
IFT80	-0.281192566	5.50546E-08	2.15617E-07
CHMP1A	0.281082632	5.57423E-08	2.18267E-07
MOBKL1A	-0.281014386	5.61734E-08	2.19912E-07
ANAPC1	-0.281004789	5.62343E-08	2.20107E-07
ZNF17	-0.280941995	5.66343E-08	2.21629E-07
43895	-0.280926466	5.67336E-08	2.21974E-07
LASS6	-0.280919673	5.67771E-08	2.22101E-07
MALT1	-0.280858744	5.71687E-08	2.23589E-07
PSMB9	0.280806342	5.75076E-08	2.24869E-07
BEST4	0.280804648	5.75186E-08	2.24869E-07
NTRK2	-0.280795926	5.75752E-08	2.25046E-07
WDR45	0.280792062	5.76003E-08	2.251E-07
ANKRD54	0.280738756	5.79476E-08	2.26413E-07
NUAK1	-0.280514544	5.94305E-08	2.32161E-07
SOX5	-0.280503711	5.95031E-08	2.32399E-07
KIFC2	0.280495061	5.95611E-08	2.3258E-07
RAB3GAP2	-0.280377837	6.03526E-08	2.35625E-07
FAM180A	-0.28037517	6.03707E-08	2.35649E-07
C20orf194	-0.280275322	6.10531E-08	2.38266E-07
NDUFA9	0.28023636	6.13214E-08	2.39266E-07

C18orf56	0.28019307	6.16208E-08	2.40388E-07
RANBP3L	-0.280163902	6.18234E-08	2.41131E-07
GDAP2	-0.280150587	6.19161E-08	2.41445E-07
NRBP1	0.280108454	6.22102E-08	2.42544E-07
POLR2E	0.280076176	6.24364E-08	2.43379E-07
ABHD5	-0.280053985	6.25924E-08	2.43939E-07
C2orf82	0.280021521	6.28213E-08	2.44783E-07
FTSJ2	0.279878305	6.38408E-08	2.48707E-07
C1orf77	0.279831395	6.41782E-08	2.49972E-07
CEP76	-0.279819297	6.42654E-08	2.50242E-07
BAT2L2	-0.279818298	6.42727E-08	2.50242E-07
MEGF10	-0.279760459	6.46916E-08	2.51824E-07
TYK2	-0.279707392	6.50783E-08	2.5328E-07
BBS5	-0.279639211	6.55784E-08	2.55177E-07
MOGAT2	-0.279602388	6.585E-08	2.56183E-07
TSGA10	-0.279570024	6.60897E-08	2.57066E-07
BZW2	0.279468948	6.68435E-08	2.59947E-07
ZNF778	-0.279425192	6.71724E-08	2.61175E-07
ZNF132	-0.279417794	6.72282E-08	2.61341E-07
NCOR2	-0.279397423	6.73819E-08	2.61887E-07
CRHR2	-0.279388298	6.74509E-08	2.62104E-07
NSUN5P1	0.279382192	6.74971E-08	2.62233E-07
ATP5J	0.279341657	6.78047E-08	2.63376E-07
EPM2A	-0.279310147	6.80446E-08	2.64257E-07
TUBB3	0.27929988	6.8123E-08	2.64484E-07
TMEM173	-0.279299009	6.81297E-08	2.64484E-07
ITGAV	-0.279162046	6.91836E-08	2.68498E-07
PAR5	-0.279161134	6.91906E-08	2.68498E-07
WDR19	-0.27915617	6.92291E-08	2.68595E-07
C2orf24	0.279093594	6.97162E-08	2.70432E-07
ATP9B	-0.279069081	6.99079E-08	2.71123E-07
RPA2	0.279058506	6.99907E-08	2.71391E-07
GCFC1	-0.278997333	7.04719E-08	2.73204E-07
GPCPD1	-0.278968393	7.07006E-08	2.74037E-07
NOV	-0.278925731	7.10391E-08	2.75296E-07
S100P	0.278868156	7.14984E-08	2.77008E-07
HSD17B4	-0.278866844	7.15089E-08	2.77008E-07
GNPAT	0.278721765	7.26791E-08	2.81487E-07
VSNL1	0.278693154	7.29121E-08	2.82334E-07
KIAA1279	-0.278683811	7.29883E-08	2.82574E-07
CASKIN2	-0.278671329	7.30903E-08	2.82914E-07
TMX1	-0.278655531	7.32195E-08	2.83359E-07
NAP1L4	0.278638323	7.33605E-08	2.8385E-07
CILP	-0.278622765	7.34883E-08	2.84289E-07
POLR3H	0.278614148	7.35591E-08	2.84508E-07
SPATA2L	0.278601567	7.36627E-08	2.84853E-07

GPRC5B	-0.278594821	7.37182E-08	2.85012E-07
C20orf196	0.278455439	7.48756E-08	2.89431E-07
HECA	-0.278450605	7.49161E-08	2.89496E-07
LOC606724	0.278449956	7.49215E-08	2.89496E-07
DNAH10	-0.27844674	7.49484E-08	2.89544E-07
ANGPT1	-0.278429936	7.50893E-08	2.90032E-07
ARHGEF15	-0.278417161	7.51965E-08	2.9039E-07
CYTH2	0.278390691	7.54192E-08	2.91193E-07
SLC25A6	0.278381415	7.54974E-08	2.91438E-07
SULT1A3	0.278360691	7.56724E-08	2.92057E-07
SPG20	-0.278216908	7.68971E-08	2.96727E-07
CD302	-0.278214882	7.69145E-08	2.96736E-07
PRICKLE3	-0.27818105	7.72055E-08	2.97801E-07
CMPK1	-0.27817794	7.72323E-08	2.97847E-07
ZNF214	-0.278096191	7.79401E-08	3.00518E-07
LIPG	-0.278093513	7.79634E-08	3.0055E-07
SOX12	0.278084345	7.80432E-08	3.00799E-07
SYNGAP1	-0.278074075	7.81327E-08	3.01086E-07
SH3BGRL	-0.278062408	7.82345E-08	3.014E-07
SSC5D	-0.278061266	7.82444E-08	3.014E-07
MAGED4	0.277939299	7.93161E-08	3.05469E-07
C14orf132	-0.277910081	7.95749E-08	3.06407E-07
SCARA5	-0.277903358	7.96346E-08	3.06577E-07
GSR	-0.277882894	7.98165E-08	3.07218E-07
OAS3	-0.277779588	8.07409E-08	3.10716E-07
IL17RA	-0.277702549	8.14369E-08	3.13334E-07
N6AMT2	0.2777002	8.14582E-08	3.13355E-07
NTF3	-0.277690492	8.15463E-08	3.13634E-07
ZNF836	-0.277651088	8.19051E-08	3.14953E-07
PDE4B	-0.277634688	8.20548E-08	3.15467E-07
CKAP4	0.277535193	8.29689E-08	3.1892E-07
DOK5	-0.277484774	8.34359E-08	3.20653E-07
EIF4G1	-0.277456498	8.36989E-08	3.21602E-07
G6PC3	0.277437401	8.3877E-08	3.22224E-07
SUV420H2	0.277410203	8.41312E-08	3.23138E-07
NGRN	0.277399178	8.42345E-08	3.23473E-07
AMBRA1	-0.277393716	8.42857E-08	3.23607E-07
POFUT1	-0.277321335	8.49671E-08	3.2616E-07
ARMCX2	-0.277245232	8.56893E-08	3.28869E-07
PCYT1A	-0.277149556	8.66057E-08	3.32259E-07
NOP2	0.277149545	8.66058E-08	3.32259E-07
RBM18	-0.277121524	8.6876E-08	3.33231E-07
PPP3CA	-0.277111319	8.69746E-08	3.33545E-07
FAM59A	-0.277047606	8.75925E-08	3.3585E-07
IRAK4	-0.27702287	8.78336E-08	3.3671E-07
ESF1	-0.276990061	8.81543E-08	3.37874E-07

IGF2BP1	0.276949978	8.85477E-08	3.39316E-07
BSND	0.276934014	8.87049E-08	3.39853E-07
GTF3C3	-0.276870041	8.93373E-08	3.4221E-07
IL1RAP	-0.276857661	8.94602E-08	3.42615E-07
C9orf69	0.276798217	9.00525E-08	3.44817E-07
CAPN10	0.276787582	9.01588E-08	3.45158E-07
WIPI2	0.27677037	9.03312E-08	3.45752E-07
PPP2R5B	-0.276747995	9.05558E-08	3.46545E-07
CALR	0.276741084	9.06253E-08	3.46744E-07
ALG10B	-0.276735829	9.06782E-08	3.4688E-07
PLA2G1B	0.276708092	9.09577E-08	3.47882E-07
IQCC	0.276679227	9.12495E-08	3.48931E-07
ASB6	0.27661665	9.18852E-08	3.51295E-07
ZNF862	-0.276457611	9.352E-08	3.57476E-07
MAGT1	-0.276427432	9.38333E-08	3.58605E-07
COL5A1	-0.27642218	9.3888E-08	3.58745E-07
YIPF6	-0.276413902	9.39742E-08	3.59005E-07
CNOT8	-0.276406196	9.40544E-08	3.59243E-07
UBR2	-0.27640094	9.41092E-08	3.59384E-07
TSR2	0.276389333	9.42304E-08	3.59777E-07
TM4SF5	0.276375766	9.43722E-08	3.60249E-07
TPX2	0.276365236	9.44823E-08	3.60601E-07
CEBPA	0.276352468	9.46161E-08	3.61042E-07
HSPA4L	-0.276319648	9.49608E-08	3.62288E-07
ARPP19	-0.276292213	9.52499E-08	3.63321E-07
TOMM20	0.276178403	9.64581E-08	3.6786E-07
TRAP1	0.27611955	9.70886E-08	3.70193E-07
LOC100133	0.276090655	9.73997E-08	3.71308E-07
GLA	0.276083029	9.74819E-08	3.71551E-07
WHAMML1	-0.27607328	9.75872E-08	3.71881E-07
BRE	0.275957704	9.88432E-08	3.76581E-07
ZNF469	-0.275956319	9.88584E-08	3.76581E-07
TIAM1	-0.275945598	9.89757E-08	3.76956E-07
PER2	-0.275889218	9.95949E-08	3.79241E-07
MAP6	-0.275874877	9.9753E-08	3.79771E-07
LRRC45	0.275827669	1.00275E-07	3.81686E-07
C11orf63	-0.2757753	1.00857E-07	3.83829E-07
RPPH1	0.275749927	1.01141E-07	3.84833E-07
PGAM5	-0.275747303	1.0117E-07	3.84871E-07
NAPG	-0.27566496	1.02095E-07	3.88316E-07
HINT3	-0.275650123	1.02262E-07	3.88878E-07
PCDHB17	-0.275590951	1.02933E-07	3.91354E-07
NID1	-0.275582555	1.03029E-07	3.91643E-07
RTF1	-0.27552518	1.03684E-07	3.94037E-07
XPO4	-0.275523906	1.03698E-07	3.94037E-07
DPY19L4	-0.275483027	1.04167E-07	3.95745E-07

RNF141	-0.275463047	1.04397E-07	3.96543E-07
FBLL1	0.275420324	1.04891E-07	3.98342E-07
KIAA1274	-0.275179045	1.07721E-07	4.09011E-07
ORC6L	0.275169805	1.07831E-07	4.0935E-07
MIA	0.27515625	1.07992E-07	4.09867E-07
LDLRAD2	0.27515492	1.08008E-07	4.09867E-07
TOR2A	0.275143217	1.08148E-07	4.10318E-07
PDLIM1	0.275136031	1.08233E-07	4.10565E-07
PAWR	-0.275131584	1.08286E-07	4.10688E-07
DCI	0.275103422	1.08623E-07	4.11887E-07
BLZF1	-0.275022446	1.09597E-07	4.15501E-07
SIN3A	-0.275000379	1.09864E-07	4.16433E-07
RPL7L1	0.274974118	1.10182E-07	4.17561E-07
BDNF	-0.274924537	1.10786E-07	4.19768E-07
LSM12	0.27491883	1.10856E-07	4.19952E-07
TSG101	0.274915004	1.10902E-07	4.2005E-07
SLC35A1	-0.274909759	1.10966E-07	4.20212E-07
LPXN	-0.274795153	1.12376E-07	4.25469E-07
CAMK1D	-0.274763008	1.12775E-07	4.26897E-07
SNORD116-	-0.274747175	1.12971E-07	4.2756E-07
LUM	-0.274742183	1.13033E-07	4.27714E-07
FAM101B	-0.274710017	1.13434E-07	4.2915E-07
C17orf57	-0.274641392	1.14294E-07	4.32322E-07
TCHH	-0.274532408	1.15673E-07	4.37454E-07
ZBTB7C	-0.274495983	1.16138E-07	4.39126E-07
SLC16A4	-0.274454987	1.16662E-07	4.41027E-07
TTC3	-0.274440122	1.16853E-07	4.4164E-07
HIST1H3I	0.2744389	1.16869E-07	4.4164E-07
TIPIN	0.274402682	1.17335E-07	4.43318E-07
TELO2	0.274376551	1.17673E-07	4.44509E-07
MED20	0.274347199	1.18053E-07	4.45861E-07
GATAD2B	-0.274308295	1.18559E-07	4.47687E-07
TNFAIP2	-0.274295688	1.18723E-07	4.48223E-07
SLC41A3	0.274283961	1.18876E-07	4.48716E-07
HP1BP3	-0.274273527	1.19013E-07	4.49145E-07
NT5C1B	-0.274200458	1.19972E-07	4.5268E-07
SNX20	-0.274155851	1.20561E-07	4.54817E-07
LPPR1	0.274114464	1.21111E-07	4.56803E-07
ANKRD6	-0.274016105	1.22426E-07	4.61675E-07
PPM1D	-0.274009483	1.22515E-07	4.61924E-07
CAV2	-0.274003396	1.22597E-07	4.62145E-07
ZNF317	-0.273978526	1.22932E-07	4.63319E-07
TADA2A	-0.27397684	1.22954E-07	4.63319E-07
PIGT	0.273969477	1.23054E-07	4.63605E-07
TMEM87B	-0.273958014	1.23209E-07	4.64083E-07
VANGL1	-0.273956642	1.23227E-07	4.64083E-07

FCGR2C	-0.273875557	1.24329E-07	4.68142E-07
C15orf37	-0.27369173	1.26861E-07	4.77586E-07
TBC1D2	-0.273683175	1.2698E-07	4.77944E-07
EFR3A	-0.273644191	1.27523E-07	4.799E-07
C9orf131	-0.273597513	1.28178E-07	4.8227E-07
TAS2R5	-0.27354949	1.28854E-07	4.84723E-07
GUSBL2	0.273414355	1.30775E-07	4.91859E-07
IFIT5	-0.273398264	1.31006E-07	4.92633E-07
PHF16	-0.27339009	1.31123E-07	4.92981E-07
ARRDC3	-0.273364772	1.31487E-07	4.94257E-07
HCST	0.273269941	1.32859E-07	4.9932E-07
ZNF16	0.273179524	1.3418E-07	5.0419E-07
IDH3G	0.273175081	1.34246E-07	5.04339E-07
LDB1	-0.273173305	1.34272E-07	5.04342E-07
VCAN	-0.273168648	1.3434E-07	5.04504E-07
DAP3	0.273136578	1.34812E-07	5.06182E-07
BAK1	0.273130732	1.34899E-07	5.0641E-07
C2orf27A	0.273002284	1.36807E-07	5.13477E-07
HPCAL1	0.272986786	1.37039E-07	5.14251E-07
TGM3	0.272927491	1.3793E-07	5.17497E-07
SRRT	0.272878068	1.38677E-07	5.20202E-07
GSS	0.272869921	1.388E-07	5.20567E-07
CHN1	-0.272863071	1.38904E-07	5.20859E-07
FOXO3	-0.272847911	1.39135E-07	5.21624E-07
CPXM2	-0.272845012	1.39179E-07	5.21691E-07
FAM72B	0.272794456	1.3995E-07	5.24482E-07
HSPB9	0.272762762	1.40435E-07	5.26202E-07
ZFP30	-0.272731375	1.40917E-07	5.27909E-07
FOXP2	-0.272600346	1.42948E-07	5.35415E-07
MIA3	-0.272582868	1.43221E-07	5.36337E-07
PRR19	0.272575917	1.43329E-07	5.36643E-07
MTUS2	-0.272510146	1.44362E-07	5.40407E-07
MOSPD3	0.27236109	1.46728E-07	5.49162E-07
ZDHHC2	-0.272335241	1.47142E-07	5.50608E-07
HEY2	-0.27233352	1.4717E-07	5.50608E-07
LOC646982	-0.272322038	1.47354E-07	5.51194E-07
GALNT7	-0.272313238	1.47496E-07	5.5162E-07
CAMK2B	-0.272300113	1.47707E-07	5.52306E-07
PLCL2	-0.272270285	1.48188E-07	5.54001E-07
GRAMD3	-0.272247742	1.48552E-07	5.5521E-07
RGMA	-0.272245678	1.48586E-07	5.5521E-07
RNF126	0.272245126	1.48595E-07	5.5521E-07
C9orf172	-0.272209368	1.49175E-07	5.57273E-07
C15orf33	-0.272195494	1.49401E-07	5.58011E-07
UPF1	-0.272150093	1.50142E-07	5.60674E-07
MED13	-0.272116796	1.50687E-07	5.62606E-07

RNF185	-0.27207582	1.51361E-07	5.65017E-07
CHMP1B	-0.272064706	1.51545E-07	5.65595E-07
KLHL3	-0.272046819	1.5184E-07	5.66489E-07
SCARNA16	0.272046764	1.51841E-07	5.66489E-07
BAHD1	-0.272024053	1.52217E-07	5.67786E-07
PDGFC	-0.271991652	1.52755E-07	5.69686E-07
TSPAN11	-0.271975591	1.53023E-07	5.70576E-07
EZH2	0.271964939	1.532E-07	5.71131E-07
DNAJB4	-0.271961599	1.53256E-07	5.71232E-07
DYRK4	0.271932928	1.53735E-07	5.7291E-07
OPCML	-0.27190894	1.54137E-07	5.74301E-07
ATP6AP1	0.271888659	1.54478E-07	5.75462E-07
TRAK2	-0.271884785	1.54543E-07	5.75597E-07
C19orf47	0.271871231	1.54771E-07	5.76339E-07
SLC37A3	-0.271861855	1.54929E-07	5.7682E-07
C7orf26	0.271855944	1.55029E-07	5.77083E-07
EOMES	-0.271838747	1.55319E-07	5.78056E-07
C6orf106	0.271770376	1.56479E-07	5.82264E-07
PVT1	0.271760938	1.56639E-07	5.82753E-07
RPN1	0.271736514	1.57056E-07	5.84194E-07
MAF	-0.271591759	1.59548E-07	5.93352E-07
MON1A	0.271516752	1.60854E-07	5.98097E-07
NME4	0.271471037	1.61655E-07	6.00964E-07
TRNT1	-0.271382098	1.63225E-07	6.06685E-07
SARS	0.271346596	1.63855E-07	6.08915E-07
MTX3	-0.271337732	1.64013E-07	6.09388E-07
C20orf29	0.271330604	1.6414E-07	6.09746E-07
SLC24A3	-0.271291818	1.64833E-07	6.12205E-07
EEF1A1	0.271250578	1.65572E-07	6.14838E-07
TMEM56	-0.271227176	1.65994E-07	6.16287E-07
AMPH	-0.27121856	1.66149E-07	6.16718E-07
LOC401463	-0.271217295	1.66172E-07	6.16718E-07
GNGT1	0.271214862	1.66216E-07	6.16766E-07
TAT	-0.271198269	1.66515E-07	6.17763E-07
RINL	0.271174773	1.6694E-07	6.19225E-07
DKFZP686I1	0.271160987	1.6719E-07	6.20037E-07
GARS	0.27112654	1.67817E-07	6.22243E-07
UTP14A	0.271110593	1.68107E-07	6.23204E-07
FANCM	-0.271104941	1.6821E-07	6.23471E-07
NOM1	-0.271080218	1.68662E-07	6.25029E-07
44082	-0.27107396	1.68777E-07	6.25337E-07
HSD17B13	-0.271015038	1.69859E-07	6.29229E-07
SQSTM1	0.270912143	1.71765E-07	6.36171E-07
GALR2	0.270898521	1.72018E-07	6.36993E-07
UBB	0.270813005	1.7362E-07	6.42805E-07
PIAS1	-0.270780932	1.74225E-07	6.44923E-07

LPIN1	-0.270757526	1.74667E-07	6.4644E-07
ABCA8	-0.270728883	1.7521E-07	6.48329E-07
OTUD5	0.270718985	1.75398E-07	6.48904E-07
GABARAPL1	-0.270707554	1.75615E-07	6.49587E-07
SH3GLB2	0.270702306	1.75715E-07	6.49836E-07
DNAJC9	0.270653675	1.76643E-07	6.53146E-07
ZNF532	-0.270643785	1.76832E-07	6.53725E-07
EPB41	-0.270634408	1.77012E-07	6.54268E-07
NCBP1	-0.270626028	1.77173E-07	6.5474E-07
KIAA0831	-0.270575096	1.78152E-07	6.58239E-07
DCBLD2	-0.270539951	1.78832E-07	6.60625E-07
NFYB	-0.270502242	1.79563E-07	6.63204E-07
ARHGAP12	-0.270485264	1.79893E-07	6.64301E-07
LOC283174	-0.270472108	1.8015E-07	6.65015E-07
PRR18	-0.270471907	1.80154E-07	6.65015E-07
ZFAND5	-0.270428828	1.80995E-07	6.67999E-07
GRIP2	-0.270426962	1.81032E-07	6.6801E-07
ACO1	-0.270406012	1.81443E-07	6.69402E-07
GPM6B	-0.270361898	1.82311E-07	6.72479E-07
ACTR1A	0.27034764	1.82592E-07	6.73393E-07
FAM134B	-0.270345355	1.82637E-07	6.73434E-07
PRDX2	0.270312536	1.83287E-07	6.75704E-07
ASB7	-0.270265909	1.84213E-07	6.78994E-07
STX10	0.270252864	1.84473E-07	6.79826E-07
TRERF1	-0.270071754	1.8812E-07	6.93137E-07
PAM	-0.270056745	1.88425E-07	6.94133E-07
GTF2F2	0.270024542	1.89082E-07	6.96424E-07
ITGB1BP2	0.270006722	1.89446E-07	6.97636E-07
ZNF146	-0.269999606	1.89592E-07	6.98044E-07
KILLIN	-0.269997089	1.89643E-07	6.98105E-07
GATAD2A	-0.26997817	1.90031E-07	6.99403E-07
WNT9A	-0.269968513	1.90229E-07	6.99959E-07
TRIM58	-0.26996739	1.90253E-07	6.99959E-07
SENP2	-0.269926912	1.91086E-07	7.02896E-07
OSBPL1A	-0.269878727	1.92083E-07	7.06432E-07
SFRS13A	-0.26987338	1.92194E-07	7.06709E-07
SYNRG	-0.269806383	1.93589E-07	7.11707E-07
GDNF	-0.269733686	1.95113E-07	7.1718E-07
TMEM177	0.269702153	1.95778E-07	7.19491E-07
EDAR	-0.269682834	1.96187E-07	7.20859E-07
SHISA3	-0.269677896	1.96291E-07	7.2111E-07
FAM189B	0.269575532	1.9847E-07	7.28981E-07
DLG2	-0.26949553	2.00189E-07	7.3516E-07
TMEM123	-0.269464294	2.00865E-07	7.37504E-07
OSBPL9	-0.269449027	2.01195E-07	7.38582E-07
KCTD11	-0.269437897	2.01437E-07	7.39332E-07

MRO	-0.269418949	2.01849E-07	7.40707E-07
ACYP1	0.269393026	2.02413E-07	7.42642E-07
SNAI1	-0.269378008	2.02741E-07	7.43707E-07
C1orf111	-0.269302466	2.04398E-07	7.49538E-07
C17orf104	-0.269302099	2.04406E-07	7.49538E-07
RORB	-0.269281544	2.04859E-07	7.51061E-07
C14orf45	-0.269275999	2.04981E-07	7.51371E-07
ATRN	-0.269247898	2.05602E-07	7.5351E-07
MYNN	-0.269224976	2.0611E-07	7.55233E-07
CCDC25	-0.269204817	2.06558E-07	7.56735E-07
C2orf63	-0.269201347	2.06636E-07	7.56878E-07
PER1	-0.269138133	2.08046E-07	7.61906E-07
ORC1L	0.269114358	2.0858E-07	7.63718E-07
CT45A3	0.269104978	2.0879E-07	7.64349E-07
SNX17	0.269089142	2.09146E-07	7.65512E-07
RBM12B	-0.269086941	2.09196E-07	7.65552E-07
AGBL5	0.269050745	2.10012E-07	7.68399E-07
RAB19	-0.269031236	2.10454E-07	7.69872E-07
TPR	-0.268991038	2.11366E-07	7.73067E-07
IRS1	-0.268952123	2.12252E-07	7.76167E-07
EMILIN1	-0.26894258	2.1247E-07	7.76821E-07
CD200	-0.268885301	2.13783E-07	7.81478E-07
BFSP1	0.268854207	2.14499E-07	7.83951E-07
KRI1	0.268846123	2.14686E-07	7.84277E-07
UTP20	-0.268845651	2.14696E-07	7.84277E-07
GPLD1	-0.268845213	2.14707E-07	7.84277E-07
TMEM2	-0.268805456	2.15626E-07	7.87492E-07
PTGER3	-0.268777386	2.16278E-07	7.89727E-07
C10orf105	-0.26873691	2.17221E-07	7.93024E-07
CTNNB1	-0.268703856	2.17993E-07	7.957E-07
ZNF750	-0.268677036	2.18623E-07	7.9785E-07
DYNLT3	-0.268659691	2.1903E-07	7.99192E-07
FLJ34503	-0.268643526	2.19411E-07	8.00434E-07
ARNT	-0.268608674	2.20234E-07	8.03289E-07
FAM131B	-0.26860228	2.20385E-07	8.03694E-07
C1orf227	0.268545804	2.21726E-07	8.08435E-07
CLK4	-0.268480123	2.23295E-07	8.13907E-07
TXNL1	0.268479566	2.23308E-07	8.13907E-07
TSPAN2	-0.268475148	2.23414E-07	8.14144E-07
FAM161A	-0.268447705	2.24073E-07	8.16396E-07
NCAPD3	-0.268382472	2.25648E-07	8.21981E-07
KIAA0240	-0.268269694	2.28394E-07	8.31835E-07
OGN	-0.268259916	2.28634E-07	8.32556E-07
C3orf64	-0.268257459	2.28694E-07	8.32623E-07
PRR14	0.26823283	2.29299E-07	8.34672E-07
CCR9	-0.268201432	2.30073E-07	8.37334E-07

CPSF7	-0.268172342	2.30791E-07	8.39797E-07
TBC1D14	-0.26814992	2.31347E-07	8.41664E-07
GPR4	-0.268147323	2.31411E-07	8.41745E-07
CDC25B	0.268133018	2.31766E-07	8.42883E-07
HAPLN4	-0.268048139	2.33884E-07	8.5043E-07
C19orf73	0.268010081	2.3484E-07	8.5375E-07
THBS1	-0.267985951	2.35448E-07	8.55804E-07
TFCP2	-0.26797853	2.35635E-07	8.56328E-07
VARS2	0.267866423	2.38482E-07	8.66514E-07
SAMD4A	-0.267805805	2.40035E-07	8.71997E-07
PTGS2	-0.267790182	2.40436E-07	8.73298E-07
ATP5SL	0.267776718	2.40783E-07	8.74398E-07
FNBP4	-0.267711455	2.42471E-07	8.80367E-07
UST	-0.267652016	2.44018E-07	8.85822E-07
WDR65	-0.267611266	2.45084E-07	8.8953E-07
FAM106C	-0.267562112	2.46376E-07	8.94056E-07
MIOS	-0.267554553	2.46575E-07	8.94616E-07
VKORC1L1	-0.267533507	2.47131E-07	8.96469E-07
HSPC157	0.267484759	2.48423E-07	9.0099E-07
HSPA1A	0.267483009	2.48469E-07	9.00994E-07
CCDC71	0.267429625	2.49891E-07	9.05986E-07
PIBF1	-0.267390505	2.50938E-07	9.09617E-07
DYSF	-0.267384169	2.51108E-07	9.10067E-07
IYD	-0.267373145	2.51404E-07	9.10974E-07
TJP2	-0.267320265	2.52829E-07	9.1597E-07
TBC1D15	-0.26730358	2.5328E-07	9.17437E-07
MDM2	-0.267295693	2.53493E-07	9.18043E-07
EWSR1	0.267263534	2.54366E-07	9.21035E-07
NAA16	-0.267245243	2.54863E-07	9.22669E-07
APOA2	0.267227038	2.55359E-07	9.24297E-07
SUOX	-0.267210079	2.55822E-07	9.25804E-07
CIAPIN1	0.267141644	2.57699E-07	9.32425E-07
TULP4	-0.267129695	2.58028E-07	9.33446E-07
BTBD10	-0.267113037	2.58487E-07	9.34937E-07
CASZ1	-0.26706872	2.59713E-07	9.392E-07
HAS2	-0.26704577	2.60349E-07	9.41333E-07
C1orf159	0.26701508	2.61204E-07	9.44249E-07
MGC87042	0.267003555	2.61525E-07	9.4524E-07
RIPK2	0.266999702	2.61633E-07	9.45457E-07
CETN2	0.266987426	2.61975E-07	9.46524E-07
GPR82	-0.266977022	2.62266E-07	9.47404E-07
WDR35	-0.266884594	2.64865E-07	9.56618E-07
SCAMP2	0.266875414	2.65125E-07	9.57381E-07
CALHM2	-0.266854922	2.65705E-07	9.5899E-07
PRKD1	-0.266854607	2.65714E-07	9.5899E-07
EHF	-0.266854572	2.65715E-07	9.5899E-07

HMGCLL1	-0.266820802	2.66673E-07	9.6226E-07
NDUFS7	0.266817853	2.66757E-07	9.6226E-07
C1orf95	-0.266817555	2.66766E-07	9.6226E-07
LOC148696	-0.266806532	2.67079E-07	9.63217E-07
POC1B	-0.266799164	2.67289E-07	9.63799E-07
C22orf9	0.266750708	2.68673E-07	9.68616E-07
ST3GAL6	-0.266724771	2.69417E-07	9.71121E-07
NMNAT2	-0.266710929	2.69815E-07	9.72379E-07
CCNL1	-0.266709198	2.69865E-07	9.72383E-07
PYY2	0.266697981	2.70188E-07	9.7337E-07
CAMK4	-0.266564538	2.74057E-07	9.87129E-07
ULK2	-0.266552136	2.74419E-07	9.88255E-07
SHISA5	0.266489089	2.76268E-07	9.94733E-07
CXorf21	-0.266487028	2.76328E-07	9.94772E-07
LCMT2	-0.266439772	2.77722E-07	9.99609E-07
MCPH1	-0.266404046	2.7878E-07	1.00324E-06
ACRV1	0.266291916	2.82127E-07	1.0151E-06
LOC653786	0.266282822	2.824E-07	1.0159E-06
GIMAP2	-0.266240586	2.83672E-07	1.02029E-06
ZCRB1	0.266230922	2.83964E-07	1.02115E-06
BHLHA15	-0.266187814	2.85269E-07	1.02566E-06
GPX2	0.266133828	2.86911E-07	1.03138E-06
PCMTD1	-0.266049307	2.89501E-07	1.0405E-06
CCDC89	-0.265999053	2.91051E-07	1.04588E-06
GJC1	-0.265956657	2.92366E-07	1.05042E-06
CD300LG	-0.265917351	2.93589E-07	1.05462E-06
P2RX3	-0.265909322	2.9384E-07	1.05533E-06
THEM5	-0.265859935	2.95385E-07	1.06069E-06
C18orf22	0.265820245	2.96633E-07	1.06498E-06
RPL9	0.26579758	2.97348E-07	1.06735E-06
AMDHD2	0.265793498	2.97477E-07	1.06762E-06
TBC1D4	-0.265728825	2.99526E-07	1.07479E-06
SKAP2	-0.265719027	2.99838E-07	1.07571E-06
NAGK	0.265702873	3.00352E-07	1.07736E-06
NFATC2IP	-0.265633844	3.02561E-07	1.08509E-06
RFK	-0.265600841	3.03622E-07	1.0887E-06
IL6	-0.265553796	3.05141E-07	1.09395E-06
43897	-0.265549308	3.05287E-07	1.09427E-06
KCNJ3	-0.265513967	3.06433E-07	1.09819E-06
SMARCAL1	0.265500682	3.06865E-07	1.09954E-06
LOC595101	-0.265420327	3.09491E-07	1.10875E-06
ZNF133	0.265414249	3.09691E-07	1.10926E-06
TOR1B	-0.265407723	3.09905E-07	1.1098E-06
AVEN	0.265406295	3.09952E-07	1.1098E-06
LAIR2	0.26540304	3.10059E-07	1.10998E-06
BNC1	-0.265392733	3.10398E-07	1.11099E-06

PDGFRA	-0.265332105	3.12399E-07	1.11796E-06
GJA1	-0.265320721	3.12776E-07	1.1191E-06
ZNF7	0.265315983	3.12933E-07	1.11947E-06
OLFM1	-0.265276952	3.1423E-07	1.1239E-06
TSC22D3	-0.26521492	3.16302E-07	1.13111E-06
C4orf36	-0.265176267	3.176E-07	1.13555E-06
GP1BA	-0.265173583	3.1769E-07	1.13567E-06
TTC5	-0.265142149	3.1875E-07	1.13925E-06
NLRC3	-0.265095907	3.20314E-07	1.14464E-06
ANXA2	0.265078208	3.20915E-07	1.14658E-06
BACE1	-0.26507547	3.21008E-07	1.14665E-06
KCNE4	-0.265074248	3.2105E-07	1.14665E-06
USP5	0.265048513	3.21925E-07	1.14957E-06
LOC283731	-0.26501465	3.23081E-07	1.15349E-06
FAM133B	-0.264967281	3.24705E-07	1.15908E-06
SCLT1	-0.264963968	3.24819E-07	1.15928E-06
CEP350	-0.264954106	3.25158E-07	1.16028E-06
CUL4A	-0.264880828	3.27689E-07	1.1691E-06
GBF1	-0.264869334	3.28088E-07	1.17031E-06
LTBP3	-0.264865682	3.28215E-07	1.17056E-06
AKT2	-0.264852992	3.28655E-07	1.17171E-06
ZNF259	0.264852953	3.28657E-07	1.17171E-06
CREB1	-0.264842206	3.29031E-07	1.17284E-06
SCARNA2	0.264794723	3.30687E-07	1.17853E-06
YWHAG	-0.264666603	3.35197E-07	1.19439E-06
ZHX1	-0.264615009	3.37029E-07	1.20071E-06
DENR	0.264601925	3.37496E-07	1.20215E-06
HAS3	-0.26457624	3.38413E-07	1.20509E-06
TNFSF15	-0.264575403	3.38443E-07	1.20509E-06
PCBD1	0.264553901	3.39213E-07	1.20762E-06
PLEKHO1	0.264456108	3.42735E-07	1.21994E-06
TC2N	-0.264452694	3.42858E-07	1.22016E-06
SYNGR4	0.264442324	3.43234E-07	1.22128E-06
FGFR1	-0.264425821	3.43833E-07	1.22319E-06
NOS2	-0.264394545	3.4497E-07	1.22702E-06
PRKCSH	0.264386587	3.4526E-07	1.22783E-06
PRAP1	0.264372232	3.45783E-07	1.22947E-06
FRMD7	-0.264338327	3.47023E-07	1.23366E-06
DDX5	-0.264320442	3.47679E-07	1.23572E-06
C11orf21	-0.26431915	3.47726E-07	1.23572E-06
NDUFV2	0.26424423	3.50486E-07	1.24531E-06
DAB1	-0.264193722	3.52359E-07	1.25174E-06
PCDHAC1	-0.264189639	3.52511E-07	1.25205E-06
TGDS	-0.264176563	3.52997E-07	1.25356E-06
FOXD4L2	-0.264142814	3.54256E-07	1.2578E-06
CPT2	-0.264116089	3.55256E-07	1.26113E-06

XPO7	-0.264078971	3.56649E-07	1.26585E-06
C6orf126	0.264013953	3.59102E-07	1.27433E-06
NSUN3	-0.26398557	3.60178E-07	1.27792E-06
DIO2	-0.263969974	3.60771E-07	1.27979E-06
C3orf32	0.263954648	3.61354E-07	1.28163E-06
C12orf5	-0.263949545	3.61549E-07	1.28209E-06
TRIB3	0.263943115	3.61794E-07	1.2827E-06
C2orf39	0.263941708	3.61847E-07	1.2827E-06
GNB2	0.263907022	3.63172E-07	1.28716E-06
C20orf4	0.263898961	3.63481E-07	1.28803E-06
TNFSF18	-0.26388901	3.63862E-07	1.28915E-06
KLHL29	-0.263885529	3.63996E-07	1.28939E-06
OXA1L	0.263883669	3.64067E-07	1.28942E-06
MAPK8IP3	-0.263822742	3.66411E-07	1.29749E-06
PCP2	0.263810193	3.66896E-07	1.29897E-06
PRMT7	0.263800274	3.67279E-07	1.3001E-06
SLC4A10	-0.263776543	3.68198E-07	1.30312E-06
TAF12	0.263628752	3.73972E-07	1.32332E-06
STX1A	0.263616566	3.74452E-07	1.32478E-06
CPSF2	-0.263591964	3.75423E-07	1.32798E-06
C16orf79	0.263569419	3.76314E-07	1.3309E-06
STRADA	0.263497628	3.79167E-07	1.34075E-06
PCSK6	-0.263377937	3.8397E-07	1.35749E-06
ALS2CR11	-0.263357233	3.84807E-07	1.36021E-06
LOC349196	-0.263335886	3.85671E-07	1.36302E-06
TTK	0.26328052	3.87922E-07	1.37074E-06
ABR	-0.263252457	3.89068E-07	1.37454E-06
COIL	0.263213516	3.90663E-07	1.37993E-06
CBLL1	-0.263187888	3.91717E-07	1.38341E-06
CT45A2	0.263183465	3.91899E-07	1.38381E-06
RGAG4	-0.263131176	3.94057E-07	1.39118E-06
PCDHB6	-0.263128988	3.94148E-07	1.39125E-06
ZBTB17	0.263111233	3.94883E-07	1.3936E-06
SGIP1	-0.263062221	3.96921E-07	1.40055E-06
WDR13	0.263011487	3.99041E-07	1.40778E-06
LPAR1	-0.26299738	3.99632E-07	1.40961E-06
IP6K2	0.262980586	4.00337E-07	1.41185E-06
ZNF780B	-0.262928203	4.02544E-07	1.41938E-06
CSTF2T	-0.262922994	4.02765E-07	1.41991E-06
FZD8	-0.262825105	4.06923E-07	1.43431E-06
CYP4A22	-0.262782523	4.08744E-07	1.44048E-06
NFKBIB	0.262774709	4.09079E-07	1.4414E-06
ZSCAN2	0.262704275	4.12112E-07	1.45183E-06
C19orf52	0.262620192	4.1576E-07	1.46443E-06
EFEMP1	-0.262587168	4.17202E-07	1.46924E-06
GNRHR	-0.262573958	4.1778E-07	1.47102E-06

BICD2	-0.262556361	4.18551E-07	1.47347E-06
PMS2L1	0.262541546	4.19201E-07	1.4755E-06
ZC3H12D	-0.262538895	4.19318E-07	1.47565E-06
MRC1	-0.262524712	4.19941E-07	1.47758E-06
KTELC1	-0.262489316	4.21501E-07	1.48281E-06
BTN3A3	-0.262470397	4.22337E-07	1.48549E-06
CDH13	-0.262403576	4.25304E-07	1.49566E-06
NEIL3	0.262349558	4.27716E-07	1.50388E-06
RNF207	-0.262300965	4.29897E-07	1.51128E-06
SMG6	-0.262283825	4.30669E-07	1.51373E-06
SERTAD4	-0.262251393	4.32133E-07	1.51861E-06
IL1RAPL2	-0.262183227	4.35227E-07	1.52908E-06
OCLM	-0.262182344	4.35267E-07	1.52908E-06
SEMA4C	-0.262166259	4.36E-07	1.53138E-06
FNDC1	-0.262148059	4.36831E-07	1.53403E-06
PRAF2	0.262140878	4.37159E-07	1.53491E-06
NDNL2	-0.262095116	4.39257E-07	1.54201E-06
CDH1	-0.262044078	4.41607E-07	1.54998E-06
HSD17B3	0.261999194	4.43685E-07	1.557E-06
PBX1	-0.261956408	4.45674E-07	1.56371E-06
SIAH1	-0.261878161	4.49333E-07	1.57627E-06
MOV10	0.261870029	4.49715E-07	1.57733E-06
FAM173A	0.261817965	4.52168E-07	1.58565E-06
NBEA	-0.261806297	4.52719E-07	1.58708E-06
PIGN	-0.261805603	4.52752E-07	1.58708E-06
LTB4R2	-0.261804319	4.52813E-07	1.58708E-06
ZNF271	0.261745792	4.55589E-07	1.59653E-06
RAB1A	-0.261710072	4.57292E-07	1.60221E-06
TRIP12	-0.261701859	4.57684E-07	1.6033E-06
GMCL1L	-0.261670911	4.59166E-07	1.60821E-06
TM6SF1	-0.261666339	4.59385E-07	1.6087E-06
UBL4A	0.261612014	4.61997E-07	1.61756E-06
LSM6	0.261583121	4.63393E-07	1.62216E-06
RHOBTB2	-0.261569836	4.64036E-07	1.62413E-06
PRIM1	0.261543948	4.65291E-07	1.62823E-06
SDPR	-0.261533528	4.65797E-07	1.62972E-06
C10orf110	-0.261520611	4.66425E-07	1.63163E-06
SYCE2	0.261465161	4.69131E-07	1.64081E-06
TTC19	-0.261450774	4.69836E-07	1.64298E-06
SPAG4	0.261444207	4.70158E-07	1.64382E-06
ZBTB22	0.261413847	4.71649E-07	1.64874E-06
KCNG2	-0.261410774	4.718E-07	1.64898E-06
HDAC1	0.261407549	4.71959E-07	1.64925E-06
FYN	-0.26133976	4.75307E-07	1.66066E-06
C10orf2	0.261284173	4.78069E-07	1.67001E-06
B9D2	0.261251787	4.79686E-07	1.67537E-06

HOXB4	-0.261244699	4.8004E-07	1.67631E-06
HEMGN	-0.261240462	4.80252E-07	1.67676E-06
C1orf135	0.261195034	4.82531E-07	1.68442E-06
RBM3	0.261164085	4.8409E-07	1.68956E-06
ZNF92	-0.261145379	4.85034E-07	1.69256E-06
ARGLU1	-0.261123287	4.86152E-07	1.69616E-06
UBAP1	-0.261102858	4.87187E-07	1.69948E-06
KISS1	0.261064353	4.89145E-07	1.70601E-06
TNIK	-0.261048642	4.89946E-07	1.7085E-06
E4F1	0.260959884	4.94494E-07	1.72406E-06
COL6A2	-0.260945301	4.95246E-07	1.72638E-06
PMS2CL	-0.260936657	4.95691E-07	1.72763E-06
BRD9	0.26092129	4.96485E-07	1.7301E-06
WNT2	-0.260916872	4.96713E-07	1.73059E-06
SUCLG2	-0.260859687	4.99678E-07	1.74061E-06
SPARC	-0.260804234	5.02569E-07	1.75038E-06
LIG1	0.260740583	5.05908E-07	1.7617E-06
NDOR1	0.260735006	5.06201E-07	1.76241E-06
MSN	-0.260730726	5.06427E-07	1.76289E-06
MCTP2	-0.260612197	5.12706E-07	1.78444E-06
DCLRE1C	0.260597209	5.13506E-07	1.78691E-06
ARL5A	-0.260589968	5.13892E-07	1.78794E-06
HAUS2	-0.260587611	5.14018E-07	1.78807E-06
C20orf107	0.260585099	5.14153E-07	1.78822E-06
LOC645752	-0.260517995	5.17751E-07	1.80042E-06
SLC9A6	-0.260453788	5.21216E-07	1.81215E-06
ITPR3	-0.260394147	5.24455E-07	1.8231E-06
S100A1	0.260330545	5.2793E-07	1.83486E-06
MSMB	0.260292719	5.30007E-07	1.84175E-06
BSG	0.26027365	5.31057E-07	1.84508E-06
OR2B6	0.260264728	5.31549E-07	1.84647E-06
PARP14	-0.260257903	5.31926E-07	1.84746E-06
ZNF764	0.26024486	5.32647E-07	1.84964E-06
RPL3	0.260235326	5.33174E-07	1.85114E-06
ZFP1	-0.260226213	5.33679E-07	1.85257E-06
PIGF	0.260208477	5.34662E-07	1.85566E-06
PSMD6	0.260202015	5.3502E-07	1.85658E-06
EXOSC9	0.260155726	5.37596E-07	1.8652E-06
SLC43A1	0.260153824	5.37702E-07	1.86524E-06
C2	0.260141811	5.38373E-07	1.86704E-06
LINGO4	-0.260141175	5.38409E-07	1.86704E-06
GP5	-0.2600965	5.4091E-07	1.87539E-06
BCL2L13	-0.25997549	5.4774E-07	1.89874E-06
IGF1	-0.259933192	5.50147E-07	1.90675E-06
PAN2	-0.259928346	5.50424E-07	1.90738E-06
C11orf75	0.259899838	5.52052E-07	1.91269E-06

E2F2	0.259847203	5.55072E-07	1.92281E-06
KLHL33	-0.259792759	5.58212E-07	1.93335E-06
FAM64A	0.259757669	5.60245E-07	1.94006E-06
KIAA1919	-0.259751235	5.60618E-07	1.94101E-06
GPN1	0.25974751	5.60834E-07	1.94142E-06
ZNF304	-0.25973605	5.615E-07	1.94339E-06
CCDC42B	0.259671564	5.65263E-07	1.95607E-06
ZNF703	-0.259668329	5.65452E-07	1.95639E-06
SRP68	0.259656918	5.66121E-07	1.95836E-06
IDH3A	-0.259634671	5.67426E-07	1.96254E-06
LRRC46	0.259622164	5.68161E-07	1.96474E-06
TAPBP	0.25961704	5.68463E-07	1.96544E-06
C10orf78	0.259601791	5.69361E-07	1.9682E-06
THAP5	-0.25958737	5.70211E-07	1.9708E-06
TMEM200A	-0.259572227	5.71106E-07	1.97342E-06
GALNTL1	-0.259571203	5.71166E-07	1.97342E-06
MOBKL2B	-0.259530098	5.73601E-07	1.98149E-06
SLC14A1	-0.259501002	5.75331E-07	1.98712E-06
OGT	-0.259484034	5.76342E-07	1.99026E-06
SLC6A6	-0.259479563	5.76608E-07	1.99084E-06
WDR17	-0.25941167	5.80672E-07	2.00452E-06
TRAF3IP2	-0.259384905	5.82282E-07	2.00918E-06
FTSJ1	0.259384584	5.82301E-07	2.00918E-06
RUFY1	0.259384196	5.82325E-07	2.00918E-06
SUSD5	-0.25934495	5.84693E-07	2.017E-06
NUP54	-0.259305568	5.87079E-07	2.02488E-06
C20orf191	0.259140721	5.97167E-07	2.05932E-06
CDK11A	-0.259128723	5.97907E-07	2.06152E-06
EIF2AK1	0.259038005	6.03536E-07	2.08057E-06
C20orf11	0.259030654	6.03995E-07	2.08167E-06
FAM186A	0.259029503	6.04067E-07	2.08167E-06
NLRC5	-0.259007031	6.0547E-07	2.08615E-06
RHEBL1	0.258996746	6.06113E-07	2.088E-06
GATA2	-0.258955729	6.08686E-07	2.0965E-06
RNMTL1	0.258862084	6.14598E-07	2.1165E-06
ZGLP1	0.2588467	6.15574E-07	2.11949E-06
PSMC2	0.258804369	6.18269E-07	2.1284E-06
FHL3	0.258798117	6.18668E-07	2.12941E-06
C21orf45	0.258674196	6.26626E-07	2.15643E-06
FAM114A2	-0.258655073	6.27863E-07	2.16031E-06
DCTN6	-0.25864129	6.28756E-07	2.16301E-06
GATM	-0.258635757	6.29114E-07	2.1636E-06
CRBN	-0.258635314	6.29143E-07	2.1636E-06
C17orf64	0.258619085	6.30197E-07	2.16685E-06
TMED7-TIC	-0.25861063	6.30746E-07	2.16836E-06
PRDM1	-0.258599607	6.31463E-07	2.17045E-06

FAM122C	-0.258558593	6.34139E-07	2.17927E-06
AHR	-0.258533539	6.35778E-07	2.18453E-06
FAM160A1	-0.2585163	6.36909E-07	2.18803E-06
EIF2AK2	-0.258502304	6.37828E-07	2.19081E-06
BCL8	-0.258490868	6.3858E-07	2.19302E-06
ATP5G3	0.258479028	6.3936E-07	2.19532E-06
C7orf11	0.2583744	6.46288E-07	2.21872E-06
CHGA	0.258366061	6.46843E-07	2.22025E-06
RHOBTB3	-0.25835877	6.47329E-07	2.22153E-06
DLX2	0.258282025	6.52465E-07	2.23877E-06
NLGN3	-0.258227209	6.56157E-07	2.25105E-06
PNMA3	0.258153431	6.61157E-07	2.26782E-06
RPF1	0.25813609	6.62338E-07	2.27148E-06
DUSP6	-0.258128203	6.62876E-07	2.27293E-06
RNF182	-0.258105883	6.644E-07	2.27776E-06
POLR1B	-0.258077444	6.66346E-07	2.28404E-06
TACR1	-0.258060241	6.67526E-07	2.28769E-06
PRKACA	-0.258048832	6.6831E-07	2.28999E-06
RFX1	-0.25804132	6.68827E-07	2.29136E-06
WBP2	0.258019621	6.70321E-07	2.29588E-06
HECTD2	-0.258018831	6.70375E-07	2.29588E-06
CDC6	0.258001337	6.71582E-07	2.29962E-06
SEPW1	0.257987326	6.72551E-07	2.30253E-06
ITGA11	-0.257884427	6.79703E-07	2.32662E-06
ASB15	-0.257845324	6.8244E-07	2.33559E-06
PPAPDC2	-0.25780571	6.85223E-07	2.34471E-06
SERTAD2	-0.257777808	6.8719E-07	2.35104E-06
FAM27C	0.257753008	6.88943E-07	2.35663E-06
THOC2	-0.257735552	6.9018E-07	2.36046E-06
RPIA	0.257720993	6.91213E-07	2.36358E-06
BMP8A	-0.257625776	6.98004E-07	2.3864E-06
OGFR	0.257623541	6.98165E-07	2.38653E-06
LRIG3	-0.257616949	6.98637E-07	2.38774E-06
CNDP1	-0.257586639	7.00815E-07	2.39477E-06
AMMECR1	-0.257577998	7.01436E-07	2.39648E-06
PANK3	-0.257559912	7.0274E-07	2.40053E-06
CBFA2T3	-0.257492856	7.07593E-07	2.41648E-06
IL2RB	-0.257492012	7.07654E-07	2.41648E-06
PAPD5	-0.257420317	7.12878E-07	2.43391E-06
OTUD1	-0.257394546	7.14765E-07	2.43993E-06
TMLHE	-0.257351184	7.17951E-07	2.45039E-06
MAP1LC3C	-0.257337071	7.18991E-07	2.45351E-06
CDR2	-0.257320051	7.20247E-07	2.45738E-06
MOSC2	-0.257299111	7.21795E-07	2.46224E-06
CT45A5	0.257289769	7.22487E-07	2.46361E-06
COG4	0.257289592	7.225E-07	2.46361E-06

ANAPC16	0.257288664	7.22569E-07	2.46361E-06
ETV4	0.257261052	7.24617E-07	2.47017E-06
NOB1	0.257221007	7.27598E-07	2.47991E-06
RTKN2	0.257207669	7.28593E-07	2.48288E-06
PRPF40A	-0.257133456	7.34155E-07	2.5014E-06
CUL9	0.257084663	7.37834E-07	2.51351E-06
SNAPC2	0.25708079	7.38127E-07	2.51407E-06
ZBTB46	-0.257062262	7.39529E-07	2.51842E-06
CSNK1A1	-0.257021997	7.42585E-07	2.52839E-06
DLGAP5	0.256846992	7.56009E-07	2.57366E-06
KIAA0114	0.256843066	7.56313E-07	2.57426E-06
MCL1	-0.256796349	7.59937E-07	2.58615E-06
SILV	-0.256793051	7.60194E-07	2.58658E-06
LRRN2	-0.256782139	7.61043E-07	2.58903E-06
COPS7B	0.256766973	7.62224E-07	2.5926E-06
POPDC3	0.256758206	7.62908E-07	2.59449E-06
COL1A2	-0.256748252	7.63686E-07	2.59669E-06
ZFC3H1	-0.256709864	7.6669E-07	2.60646E-06
TTLL1	0.256595982	7.7567E-07	2.63654E-06
UBE4A	-0.25656071	7.78472E-07	2.64561E-06
HS3ST1	-0.256444407	7.8778E-07	2.67678E-06
POLE2	0.256372548	7.93584E-07	2.69605E-06
XCL1	0.256327979	7.97204E-07	2.70788E-06
C10orf125	0.256298404	7.99615E-07	2.71561E-06
C14orf21	-0.256291466	8.00182E-07	2.71707E-06
RNF138	-0.256268837	8.02032E-07	2.72289E-06
SPDYE1	-0.256221721	8.05899E-07	2.73555E-06
CLEC16A	-0.256212425	8.06664E-07	2.73768E-06
ILDR2	-0.256198332	8.07825E-07	2.74116E-06
VAMP7	-0.256176503	8.09627E-07	2.7468E-06
DENND1C	-0.256172645	8.09946E-07	2.74741E-06
CTAGE6	-0.256086719	8.17078E-07	2.77114E-06
AIFM3	0.256027698	8.22012E-07	2.78739E-06
NUDT10	-0.256007715	8.23689E-07	2.7926E-06
LAMC2	-0.255995284	8.24734E-07	2.79567E-06
PAX8	0.255977056	8.26268E-07	2.8004E-06
TAF1B	-0.255943698	8.29083E-07	2.80946E-06
ANXA8L2	-0.25587728	8.34715E-07	2.82806E-06
DDX26B	-0.255823581	8.39296E-07	2.8431E-06
ZNF181	-0.25580123	8.4121E-07	2.8491E-06
USP51	-0.255787837	8.42358E-07	2.8525E-06
FPR3	-0.255760946	8.44669E-07	2.85984E-06
MARVELD1	-0.255746901	8.45879E-07	2.86345E-06
KLRAQ1	-0.255702884	8.4968E-07	2.87583E-06
SMCR8	-0.255691782	8.50641E-07	2.87859E-06
SGMS1	-0.25564849	8.544E-07	2.89082E-06

PAAF1	0.255623442	8.56581E-07	2.89771E-06
AKR1C2	0.255551169	8.62907E-07	2.91861E-06
RMRP	0.25552629	8.65095E-07	2.92552E-06
DIRC2	-0.255436765	8.73011E-07	2.95179E-06
ZNF516	-0.255428925	8.73708E-07	2.95364E-06
ATPAF2	0.255419069	8.74585E-07	2.9561E-06
LOC285033	-0.2553745	8.78558E-07	2.96903E-06
C21orf88	-0.255335628	8.82039E-07	2.98028E-06
RALB	-0.255286395	8.86465E-07	2.99473E-06
ZBED3	-0.255274975	8.87495E-07	2.9977E-06
FPGT	-0.25527227	8.87739E-07	2.99802E-06
GPR19	0.255122016	9.014E-07	3.04364E-06
C6orf41	0.255116833	9.01875E-07	3.04473E-06
FGF18	-0.255096278	9.0376E-07	3.05057E-06
NXNL2	-0.254923654	9.19744E-07	3.104E-06
ADAM19	-0.254865647	9.25175E-07	3.1218E-06
OTUD7A	-0.254856155	9.26067E-07	3.12428E-06
EIF2S3	0.254806258	9.30768E-07	3.13961E-06
GOLGA6A	-0.25477453	9.33769E-07	3.1492E-06
LOC728743	0.254765713	9.34605E-07	3.15148E-06
CARHSP1	0.254759268	9.35216E-07	3.15301E-06
SLC25A37	-0.254724007	9.38567E-07	3.16377E-06
BBS2	-0.254722162	9.38743E-07	3.16383E-06
GALNT4	-0.25468249	9.42528E-07	3.17605E-06
PLBD2	-0.254678716	9.42888E-07	3.17673E-06
HNRNPM	0.254640934	9.46508E-07	3.18838E-06
C16orf48	0.254629033	9.47651E-07	3.19169E-06
GRK5	-0.254623117	9.48219E-07	3.19307E-06
SIP1	0.25459919	9.50522E-07	3.20028E-06
MMP14	-0.2545749	9.52866E-07	3.20763E-06
PANK4	-0.254554479	9.5484E-07	3.21373E-06
SEC16A	-0.254527976	9.57409E-07	3.22183E-06
SLC39A8	-0.254495838	9.60532E-07	3.23179E-06
ADAMTSL2	-0.254479948	9.6208E-07	3.23646E-06
GINS1	0.254432589	9.66707E-07	3.25147E-06
AK7	-0.254408167	9.69102E-07	3.25898E-06
RPS15AP10	-0.254385375	9.71342E-07	3.26596E-06
SH3YL1	-0.254382917	9.71584E-07	3.26622E-06
UBA3	-0.254315041	9.78286E-07	3.2882E-06
TRUB2	0.254305754	9.79206E-07	3.29073E-06
P704P	-0.254297574	9.80018E-07	3.29291E-06
PNPLA3	-0.254227964	9.86948E-07	3.31563E-06
TRIOBP	-0.254197343	9.90012E-07	3.32536E-06
OCEL1	0.254119707	9.9782E-07	3.35103E-06
EFHD1	-0.254046075	1.00528E-06	3.37551E-06
KBTBD8	-0.254035773	1.00633E-06	3.37846E-06

RNF4	-0.254000452	1.00993E-06	3.38998E-06
SNX15	0.253982048	1.01181E-06	3.39572E-06
ULBP3	-0.25393327	1.01681E-06	3.41194E-06
CAPRIN2	-0.253808768	1.02969E-06	3.45456E-06
HNF1A	0.253772214	1.0335E-06	3.46676E-06
FAM102A	-0.253725612	1.03838E-06	3.48253E-06
GANAB	-0.253709589	1.04006E-06	3.48758E-06
MRPL1	0.253655441	1.04576E-06	3.50612E-06
GALNT3	-0.253619242	1.04959E-06	3.51836E-06
C8orf73	0.253538307	1.0582E-06	3.54663E-06
PRELP	-0.253529667	1.05912E-06	3.54913E-06
NUDT14	0.253455736	1.06706E-06	3.5751E-06
DDX28	0.253448878	1.06779E-06	3.57698E-06
B4GALT5	-0.253422109	1.07068E-06	3.58605E-06
BNIP3L	-0.253388736	1.07429E-06	3.59753E-06
CSTL1	0.253342354	1.07933E-06	3.6138E-06
C17orf58	0.253326979	1.081E-06	3.61879E-06
TMEM231	-0.253320757	1.08168E-06	3.62046E-06
FOXO4	-0.253302729	1.08365E-06	3.62643E-06
LAMB1	-0.253294426	1.08456E-06	3.62886E-06
GEN1	-0.25327887	1.08626E-06	3.63395E-06
ASNA1	0.253202946	1.09461E-06	3.66125E-06
WNT2B	-0.253181697	1.09695E-06	3.66848E-06
COX7A2L	0.253164189	1.09889E-06	3.67435E-06
FECH	-0.253092691	1.10684E-06	3.70029E-06
SAP18	0.253038615	1.11288E-06	3.71988E-06
EBF1	-0.253030904	1.11375E-06	3.72214E-06
TMEM120B	0.253022441	1.1147E-06	3.72469E-06
SGEF	-0.252964711	1.12119E-06	3.74578E-06
CCDC51	0.252961813	1.12152E-06	3.74624E-06
ST3GAL3	0.252950772	1.12277E-06	3.74977E-06
DQX1	0.252949132	1.12295E-06	3.74977E-06
CLCN4	-0.252936138	1.12442E-06	3.75397E-06
SRD5A1	-0.252934681	1.12459E-06	3.75397E-06
MNAT1	0.252878486	1.13097E-06	3.77463E-06
UGT1A3	-0.252825513	1.13701E-06	3.79418E-06
C7orf44	0.25281714	1.13797E-06	3.79674E-06
SMPD2	0.252758462	1.14471E-06	3.81858E-06
TCF19	0.252737697	1.1471E-06	3.82592E-06
KLHL6	-0.252713406	1.14991E-06	3.83464E-06
ZNF507	-0.252658688	1.15626E-06	3.85516E-06
WLS	-0.252584638	1.1649E-06	3.88331E-06
DGKZ	0.252555861	1.16827E-06	3.89391E-06
NCEH1	-0.252552772	1.16863E-06	3.89447E-06
TSTA3	0.252529036	1.17143E-06	3.9026E-06
MAPK10	-0.252528695	1.17147E-06	3.9026E-06

ZBTB33	-0.252504157	1.17436E-06	3.91158E-06
VIPR1	-0.25243653	1.18237E-06	3.93759E-06
TANC2	-0.25237062	1.19022E-06	3.96309E-06
C13orf33	-0.252333941	1.19461E-06	3.97705E-06
TSSK6	-0.252279809	1.20112E-06	3.99805E-06
SQRDL	0.252270377	1.20226E-06	4.00117E-06
MPZL2	-0.252246114	1.20519E-06	4.01026E-06
USP10	-0.252159171	1.21575E-06	4.04473E-06
EID1	-0.252137569	1.21839E-06	4.05283E-06
CAMSAP1L1	-0.252069115	1.22679E-06	4.08008E-06
SSH2	-0.252041966	1.23013E-06	4.09052E-06
RCAN2	-0.252019452	1.23292E-06	4.09908E-06
VPS8	-0.251962873	1.23993E-06	4.12172E-06
MYO5C	-0.251917598	1.24557E-06	4.13978E-06
LOC729234	0.251888355	1.24923E-06	4.15124E-06
PQLC3	-0.251877692	1.25056E-06	4.15498E-06
CCNB1IP1	0.251860646	1.2527E-06	4.16139E-06
ABCD2	-0.251847397	1.25437E-06	4.16623E-06
CBX7	-0.251750637	1.26659E-06	4.20611E-06
LOC100129	-0.251715944	1.27099E-06	4.22005E-06
LOC644165	0.251662901	1.27776E-06	4.24121E-06
EFCAB10	-0.251662678	1.27779E-06	4.24121E-06
FAM3A	0.251554908	1.29165E-06	4.2865E-06
GFRA1	-0.251472865	1.3023E-06	4.32112E-06
UNC13B	-0.251468731	1.30284E-06	4.32215E-06
MSTN	-0.251467162	1.30305E-06	4.32215E-06
DDHD1	-0.251435237	1.30721E-06	4.33525E-06
C6orf186	-0.251422461	1.30889E-06	4.34007E-06
IL7	-0.251348905	1.31855E-06	4.37125E-06
ATG9A	0.251347569	1.31873E-06	4.37125E-06
APLNR	-0.25133078	1.32094E-06	4.37787E-06
LOC348926	0.251303298	1.32458E-06	4.38918E-06
C10orf11	0.251277862	1.32795E-06	4.39963E-06
MLH3	-0.251260066	1.33032E-06	4.40673E-06
TNNI2	0.251252543	1.33132E-06	4.40931E-06
AASS	-0.251248673	1.33183E-06	4.41028E-06
NXF3	-0.251228677	1.3345E-06	4.41837E-06
CCAR1	-0.251219515	1.33572E-06	4.42168E-06
SCYL3	-0.25120649	1.33746E-06	4.42671E-06
NOS1	-0.251125257	1.34836E-06	4.46205E-06
PLCB4	-0.251103644	1.35127E-06	4.47095E-06
MMP2	-0.251060536	1.35711E-06	4.4895E-06
SLC9A8	-0.250984964	1.36739E-06	4.52276E-06
ART4	-0.25097975	1.3681E-06	4.52436E-06
C6orf170	-0.250940618	1.37345E-06	4.54132E-06
PABPC5	-0.250908611	1.37785E-06	4.55509E-06

MATN2	-0.250880264	1.38175E-06	4.56724E-06
C6orf81	0.250868136	1.38343E-06	4.57202E-06
RBM41	-0.250823282	1.38963E-06	4.59176E-06
ETV3L	-0.250820212	1.39006E-06	4.59241E-06
DYNC1LI1	0.250745489	1.40046E-06	4.626E-06
KIAA1984	0.250698893	1.40698E-06	4.64678E-06
WRNIP1	0.250611379	1.41932E-06	4.68673E-06
RHOB	-0.250603967	1.42037E-06	4.68942E-06
CHTF18	0.25056711	1.42559E-06	4.7059E-06
UCHL3	0.250495192	1.43585E-06	4.73896E-06
PTGDR	-0.250492356	1.43625E-06	4.73952E-06
IGF1R	-0.250480061	1.43801E-06	4.74454E-06
PLGLA	-0.250445014	1.44304E-06	4.76035E-06
PMM2	0.250373183	1.45341E-06	4.79374E-06
GPR153	-0.250325075	1.46039E-06	4.81596E-06
BUD13	0.250290616	1.4654E-06	4.83171E-06
ATXN7L3B	-0.250272097	1.46811E-06	4.83983E-06
LRRC70	-0.250261339	1.46968E-06	4.84421E-06
ING4	0.250219815	1.47577E-06	4.86347E-06
ALPL	-0.25019299	1.47971E-06	4.87566E-06
PCDHGC3	-0.250084523	1.49577E-06	4.92775E-06
AMT	0.250079718	1.49648E-06	4.92929E-06
SGTA	0.250028189	1.50417E-06	4.9538E-06
NAA20	0.25002519	1.50462E-06	4.95446E-06
CMTM7	0.24998405	1.51079E-06	4.97394E-06
VCPIP1	-0.249969631	1.51296E-06	4.98026E-06
SPAG7	0.249921222	1.52026E-06	5.00345E-06
CRISPLD1	-0.249911052	1.52179E-06	5.00769E-06
MED16	0.249892405	1.52462E-06	5.01615E-06
SLC25A30	-0.249853765	1.53048E-06	5.03462E-06
C22orf43	0.249826739	1.5346E-06	5.04732E-06
SMARCE1	0.249790126	1.54019E-06	5.06488E-06
ACTR3	-0.249739758	1.54792E-06	5.08944E-06
PGBD3	-0.249702874	1.5536E-06	5.10728E-06
PRICKLE2	-0.249680259	1.55709E-06	5.11792E-06
SIRPA	-0.249565879	1.57487E-06	5.17551E-06
ESCO1	-0.249556639	1.57632E-06	5.1794E-06
WDR25	0.249507793	1.58398E-06	5.20371E-06
TLK2	0.249481486	1.58812E-06	5.21642E-06
RNF166	0.249479892	1.58837E-06	5.21642E-06
SPHK1	0.249478094	1.58865E-06	5.21649E-06
FAM71E1	0.249448307	1.59335E-06	5.23106E-06
FKBP7	-0.249424189	1.59717E-06	5.24273E-06
SLC39A5	0.249409912	1.59943E-06	5.24929E-06
COG5	-0.249387082	1.60306E-06	5.26033E-06
HIST1H1B	0.249369782	1.60581E-06	5.26849E-06

JMJD5	-0.249367996	1.6061E-06	5.26856E-06
BCL2L11	-0.249356813	1.60788E-06	5.27353E-06
TSFM	0.249232941	1.62775E-06	5.33782E-06
DCLK2	-0.249187332	1.63512E-06	5.36111E-06
LAPTM4B	0.249174907	1.63713E-06	5.36684E-06
C19orf25	0.24915138	1.64095E-06	5.37848E-06
ALMS1	-0.249148539	1.64142E-06	5.3791E-06
CDC26	0.249060234	1.65584E-06	5.42547E-06
GNL2	0.249025864	1.66148E-06	5.44307E-06
MDH2	0.249018207	1.66274E-06	5.44631E-06
PYCARD	0.249015467	1.6632E-06	5.44689E-06
C5orf53	-0.248985485	1.66814E-06	5.46218E-06
AQP1	-0.248964445	1.67162E-06	5.47267E-06
ACBD3	-0.248955473	1.6731E-06	5.47664E-06
CSAD	-0.248946564	1.67458E-06	5.48057E-06
CIC	-0.24892285	1.67852E-06	5.49255E-06
MASP1	-0.248893001	1.68348E-06	5.50789E-06
MRPL15	0.248873472	1.68674E-06	5.51764E-06
CDCA4	0.248822573	1.69526E-06	5.54459E-06
COL18A1	-0.248820035	1.69568E-06	5.54507E-06
NCAM2	-0.248768903	1.70428E-06	5.57228E-06
ELN	-0.248741365	1.70893E-06	5.58656E-06
SNAP23	-0.248736128	1.70981E-06	5.58854E-06
C12orf65	0.248719153	1.71269E-06	5.59701E-06
ZNF493	-0.248717411	1.71298E-06	5.59705E-06
SEC31B	-0.248676782	1.71988E-06	5.61866E-06
BMPR1A	-0.248658569	1.72298E-06	5.62787E-06
SHC4	-0.248602716	1.73252E-06	5.6581E-06
C9orf169	0.248553108	1.74103E-06	5.68498E-06
ASB3	-0.248487138	1.75242E-06	5.72122E-06
EXOC1	-0.248475324	1.75447E-06	5.72696E-06
RBM17	0.248469258	1.75552E-06	5.72946E-06
ZNF347	-0.248464796	1.75629E-06	5.73104E-06
FHL5	-0.248459739	1.75717E-06	5.73297E-06
RAB5B	-0.248432524	1.7619E-06	5.74746E-06
NCRNA0018	-0.248342402	1.77765E-06	5.79789E-06
KDSR	-0.248312649	1.78288E-06	5.814E-06
AKR7A2	0.248300133	1.78509E-06	5.82023E-06
EFCAB4B	-0.248280262	1.78859E-06	5.83071E-06
POLR3F	0.248240361	1.79565E-06	5.85276E-06
ZNF765	-0.24813253	1.81486E-06	5.91439E-06
LYZL2	0.248129026	1.81548E-06	5.91547E-06
NDUFA4L2	0.248104557	1.81987E-06	5.9288E-06
LACTB	-0.248077501	1.82473E-06	5.94367E-06
MTMR15	-0.24804686	1.83026E-06	5.96068E-06
TTC22	-0.248038822	1.83171E-06	5.96443E-06

KIF5B	-0.247975326	1.84321E-06	6.00091E-06
UNC5B	-0.247954269	1.84704E-06	6.0124E-06
SH3PXD2B	-0.247945884	1.84857E-06	6.01595E-06
MAP3K1	-0.247944955	1.84874E-06	6.01595E-06
ZNF131	-0.247921575	1.853E-06	6.02885E-06
CNTD1	0.247917614	1.85373E-06	6.03021E-06
FLYWCH2	0.247905457	1.85595E-06	6.03646E-06
HERC6	-0.247877739	1.86103E-06	6.05198E-06
NAT2	-0.247793338	1.87657E-06	6.10151E-06
UBR7	-0.247791706	1.87687E-06	6.10151E-06
KGFLP2	-0.247777478	1.8795E-06	6.10907E-06
UBE2D3	-0.247710956	1.89185E-06	6.14822E-06
WSCD2	-0.247703565	1.89323E-06	6.15169E-06
C8orf80	-0.247685578	1.89659E-06	6.16159E-06
PSMD7	0.247656989	1.90193E-06	6.17795E-06
C1QBP	0.247635291	1.906E-06	6.19015E-06
FAM102B	-0.247607132	1.91129E-06	6.20632E-06
LOC100133	0.247602657	1.91213E-06	6.20804E-06
SLC25A11	0.247580099	1.91638E-06	6.22083E-06
STIM1	-0.247553103	1.92148E-06	6.23636E-06
TMCO7	-0.247500672	1.93142E-06	6.2676E-06
GJB2	-0.247418952	1.94701E-06	6.31715E-06
VRK1	0.247399939	1.95065E-06	6.32794E-06
ZNF165	0.247380713	1.95434E-06	6.33889E-06
DYNLT1	0.247366935	1.95699E-06	6.34645E-06
ARMCX5	-0.247361266	1.95809E-06	6.34895E-06
GAMT	0.247329677	1.96418E-06	6.36767E-06
COL6A1	-0.24731141	1.96771E-06	6.37807E-06
RAC3	0.24717705	1.99386E-06	6.4618E-06
C8ORFK29	0.247117068	2.00565E-06	6.49893E-06
FCN3	-0.247058868	2.01714E-06	6.53512E-06
S100A4	0.247048332	2.01923E-06	6.5401E-06
NCRNA0008	0.247047802	2.01934E-06	6.5401E-06
ABCF3	0.247045391	2.01981E-06	6.54058E-06
C11orf80	0.247035081	2.02186E-06	6.54614E-06
FAM82A2	-0.247024303	2.024E-06	6.55201E-06
MTERFD1	0.246998584	2.02912E-06	6.56751E-06
RGS6	-0.24685799	2.05731E-06	6.65768E-06
LOC100134	0.246846526	2.05963E-06	6.66409E-06
XRRA1	0.2467421	2.08083E-06	6.7316E-06
CLDND2	0.246732962	2.0827E-06	6.73655E-06
TLK1	-0.246707206	2.08796E-06	6.75248E-06
HGF	-0.246703535	2.08872E-06	6.75382E-06
TRHDE	-0.246701002	2.08923E-06	6.7544E-06
DNAJC24	-0.24669345	2.09078E-06	6.7583E-06
TMEM39B	0.246602187	2.10957E-06	6.81793E-06

RGS22	-0.246538576	2.12276E-06	6.85945E-06
SCAI	-0.246502585	2.13026E-06	6.88256E-06
ANGPTL2	-0.246468111	2.13747E-06	6.90473E-06
SGSM3	0.246453908	2.14044E-06	6.91322E-06
ESPNP	-0.246444689	2.14238E-06	6.91834E-06
CASK	-0.246431328	2.14518E-06	6.92628E-06
SEPN1	-0.246421814	2.14718E-06	6.93161E-06
LQK1	0.246410136	2.14964E-06	6.93842E-06
FIZ1	0.246385156	2.1549E-06	6.95428E-06
PPARG	0.246359937	2.16023E-06	6.97035E-06
APEH	0.246347767	2.16281E-06	6.97753E-06
PRRT3	0.246307915	2.17126E-06	7.00367E-06
CDK10	0.246259857	2.1815E-06	7.03555E-06
SLC23A2	-0.246245821	2.1845E-06	7.04408E-06
HSN2	-0.246205402	2.19315E-06	7.07085E-06
ZNF789	0.246200026	2.19431E-06	7.07342E-06
HLTF	-0.246191609	2.19611E-06	7.0781E-06
NAV3	-0.246162657	2.20234E-06	7.09703E-06
MARCKSL1	0.246148555	2.20538E-06	7.10568E-06
SMARCB1	0.246121917	2.21114E-06	7.12192E-06
SOD1	0.246121902	2.21114E-06	7.12192E-06
SIRT4	0.246049492	2.22685E-06	7.17137E-06
LOC388152	0.24600986	2.2355E-06	7.19805E-06
ATG4A	-0.245968997	2.24445E-06	7.22569E-06
GPBP1L1	-0.245908593	2.25773E-06	7.26723E-06
RPLP0P2	0.245906617	2.25817E-06	7.26723E-06
PDE5A	-0.245904869	2.25856E-06	7.26723E-06
MON1B	-0.245903732	2.25881E-06	7.26723E-06
AXL	-0.24587781	2.26454E-06	7.28448E-06
PRDM6	-0.245861139	2.26823E-06	7.29518E-06
EXOC3L2	-0.245854319	2.26974E-06	7.29886E-06
NCOA7	-0.245831334	2.27484E-06	7.31409E-06
ASPN	-0.245829326	2.27529E-06	7.31434E-06
ZNF284	-0.245813724	2.27876E-06	7.32432E-06
PDSS2	-0.245797823	2.2823E-06	7.33452E-06
CRISP2	0.245779001	2.2865E-06	7.34683E-06
USP31	-0.245768636	2.28882E-06	7.35308E-06
CHAF1B	0.245747304	2.29359E-06	7.36723E-06
VAMP4	-0.245692423	2.30591E-06	7.40562E-06
CCDC97	0.245675141	2.30981E-06	7.41693E-06
ITK	-0.245670665	2.31081E-06	7.41897E-06
GPRASP2	-0.245662998	2.31255E-06	7.42333E-06
HHIP	-0.245657324	2.31383E-06	7.42625E-06
C19orf45	0.245607981	2.325E-06	7.46089E-06
KHNYN	-0.245497243	2.35025E-06	7.54073E-06
BHLHE41	-0.245420546	2.3679E-06	7.59612E-06

ODF2L	-0.245415794	2.369E-06	7.59842E-06
TK2	-0.245376899	2.378E-06	7.62606E-06
DDA1	0.245331671	2.38851E-06	7.65735E-06
C20orf112	-0.245331602	2.38852E-06	7.65735E-06
TMEM14A	0.245252483	2.40701E-06	7.71536E-06
SMS	0.245250859	2.40739E-06	7.71536E-06
DLEU1	0.245221555	2.41428E-06	7.73618E-06
SLC7A6OS	-0.245188341	2.4221E-06	7.76E-06
PAIP2	0.245183348	2.42328E-06	7.76253E-06
RNF115	-0.245159481	2.42892E-06	7.77934E-06
HIST1H3D	0.245148059	2.43162E-06	7.78568E-06
FAM185A	-0.245147818	2.43168E-06	7.78568E-06
PHF20	-0.245027191	2.46041E-06	7.87641E-06
HINFP	0.245021274	2.46183E-06	7.87968E-06
GIYD2	0.244972226	2.47361E-06	7.91611E-06
THRSP	-0.244969043	2.47438E-06	7.9173E-06
STAG3L4	0.244960793	2.47637E-06	7.92238E-06
NPTXR	-0.244947959	2.47946E-06	7.93101E-06
ZNF34	0.244939296	2.48155E-06	7.93642E-06
C2orf48	0.244934502	2.48271E-06	7.93885E-06
RNF5P1	0.244877413	2.49654E-06	7.98179E-06
NUDCD1	0.24484046	2.50553E-06	8.00925E-06
ADAMTS3	-0.244813748	2.51205E-06	8.0288E-06
LAX1	-0.24479955	2.51552E-06	8.0386E-06
FAM190A	-0.244794595	2.51673E-06	8.04119E-06
ZNF597	-0.244786798	2.51864E-06	8.046E-06
ABCA6	-0.244771644	2.52236E-06	8.0557E-06
CLK1	-0.244771105	2.52249E-06	8.0557E-06
TUBB4	0.244755918	2.52622E-06	8.06631E-06
TGFB3	-0.244693386	2.54162E-06	8.1142E-06
SAR1A	0.244685617	2.54354E-06	8.11903E-06
NDRG2	-0.24466174	2.54945E-06	8.13659E-06
IFT140	-0.244659752	2.54994E-06	8.13686E-06
FAM104A	0.244643354	2.55401E-06	8.14853E-06
GABBR1	-0.244634954	2.5561E-06	8.15388E-06
SLC22A25	-0.24461416	2.56127E-06	8.16907E-06
ELOVL5	-0.244601258	2.56448E-06	8.178E-06
C3orf37	0.244556949	2.57555E-06	8.21197E-06
CACHD1	-0.244506486	2.5882E-06	8.25101E-06
LRRC15	-0.244464196	2.59885E-06	8.28364E-06
CREB3	0.244441806	2.60451E-06	8.30034E-06
CLEC4M	-0.244430205	2.60745E-06	8.30837E-06
PLA2G4A	-0.244363912	2.62428E-06	8.36068E-06
CXorf40A	0.24434916	2.62804E-06	8.37091E-06
UBE2E1	0.244348014	2.62834E-06	8.37091E-06
KIAA1191	-0.244311051	2.63778E-06	8.39966E-06

MRM1	0.244200513	2.66623E-06	8.48887E-06
NINJ2	0.244184662	2.67033E-06	8.50058E-06
GPR21	-0.244174874	2.67287E-06	8.50729E-06
POLR3A	-0.244172117	2.67358E-06	8.50821E-06
TMEM79	0.244157904	2.67727E-06	8.51749E-06
CYP46A1	-0.244157583	2.67735E-06	8.51749E-06
PEX12	-0.244134013	2.68348E-06	8.53561E-06
POLG2	0.24411555	2.68829E-06	8.54955E-06
FNBP1L	-0.244026004	2.71173E-06	8.62272E-06
PKHD1	-0.244013425	2.71504E-06	8.63186E-06
ODZ4	-0.243921601	2.7393E-06	8.70762E-06
SMC5	-0.243899951	2.74506E-06	8.72334E-06
SFRS13B	0.243899689	2.74513E-06	8.72334E-06
DCAF8	-0.243873456	2.75211E-06	8.74283E-06
IGDCC3	0.243873359	2.75214E-06	8.74283E-06
NCRNA0009	0.243852474	2.75771E-06	8.75914E-06
AADAT	-0.243837991	2.76158E-06	8.77004E-06
DAB2IP	-0.243810268	2.76901E-06	8.79221E-06
CPLX2	0.243798943	2.77204E-06	8.79952E-06
SELP	-0.243798392	2.77219E-06	8.79952E-06
TTYH2	-0.243773338	2.77893E-06	8.81949E-06
GGH	0.243741386	2.78754E-06	8.84541E-06
RUNX2	-0.243724101	2.7922E-06	8.85881E-06
PGRMC1	-0.243681284	2.8038E-06	8.89369E-06
APPL2	-0.243680213	2.80409E-06	8.89369E-06
DNAJB5	-0.24367113	2.80656E-06	8.9001E-06
MYO5B	-0.243640437	2.81491E-06	8.92515E-06
HDHD2	-0.243609259	2.82341E-06	8.95069E-06
PLCXD2	-0.243603342	2.82503E-06	8.95439E-06
C11orf95	-0.243577765	2.83203E-06	8.97515E-06
ZDHHC15	-0.243573649	2.83316E-06	8.9773E-06
ADD1	-0.243525529	2.84638E-06	9.01775E-06
COL10A1	-0.243520621	2.84773E-06	9.0206E-06
C9orf128	-0.243503308	2.8525E-06	9.03428E-06
NDRG1	0.243457342	2.86521E-06	9.07308E-06
AKNA	-0.243436887	2.87088E-06	9.0896E-06
FAM171B	-0.243426274	2.87383E-06	9.09748E-06
MPV17L2	0.243407641	2.87901E-06	9.11244E-06
AVP	0.243397558	2.88182E-06	9.11988E-06
SYNM	-0.243374204	2.88833E-06	9.13904E-06
KCTD1	-0.243365489	2.89076E-06	9.14528E-06
SLFN14	-0.243324619	2.90221E-06	9.18002E-06
CCR8	-0.243293922	2.91083E-06	9.2055E-06
ARL10	-0.243292651	2.91118E-06	9.2055E-06
LOC100128	0.243287524	2.91263E-06	9.2086E-06
CCDC113	-0.243271625	2.9171E-06	9.22129E-06

AGAP5	-0.243244234	2.92484E-06	9.24426E-06
IGFALS	-0.243234141	2.92769E-06	9.25181E-06
LOC220729	-0.243180177	2.94299E-06	9.29869E-06
LIPE	-0.243162228	2.94809E-06	9.31334E-06
COQ5	0.24313412	2.95611E-06	9.33717E-06
PGAP2	0.243122042	2.95956E-06	9.34659E-06
C16orf62	-0.243098057	2.96642E-06	9.36677E-06
C14orf181	0.243093557	2.96771E-06	9.36936E-06
TRADD	0.2430634	2.97636E-06	9.39518E-06
CARS2	0.242994226	2.9963E-06	9.45662E-06
VASH1	-0.242987552	2.99823E-06	9.46121E-06
SALL1	-0.242976489	3.00143E-06	9.46982E-06
AVIL	-0.242897368	3.02443E-06	9.54087E-06
SEC16B	-0.242877406	3.03026E-06	9.55775E-06
TMEM132C	-0.242850535	3.03812E-06	9.58103E-06
E2F6	0.242834078	3.04295E-06	9.59473E-06
CCDC80	-0.242818351	3.04757E-06	9.60777E-06
MAB21L2	-0.242705496	3.08091E-06	9.71134E-06
TIGD1	0.242683452	3.08746E-06	9.73045E-06
KIF11	0.242676523	3.08952E-06	9.73541E-06
KLF12	-0.24267147	3.09103E-06	9.73861E-06
SPPL3	0.242653351	3.09643E-06	9.75409E-06
FOXA3	0.242616321	3.1075E-06	9.78741E-06
PIK3C3	-0.242592391	3.11467E-06	9.80844E-06
PTPRU	-0.242588606	3.1158E-06	9.81047E-06
RNF208	0.24254034	3.13032E-06	9.85463E-06
FAM70B	-0.242477618	3.14929E-06	9.91277E-06
ADAM33	-0.242434196	3.16248E-06	9.95272E-06
UBTD1	0.24240878	3.17023E-06	9.97553E-06
CORO2B	-0.242381967	3.17842E-06	9.99973E-06
ERI1	-0.242366473	3.18316E-06	1.00131E-05
BHLHB9	-0.242358097	3.18573E-06	1.00196E-05
ZNF688	0.242352078	3.18758E-06	1.00238E-05
HMGB2	0.242308292	3.20104E-06	1.00645E-05
SCAMP1	-0.242286604	3.20772E-06	1.00839E-05
C4orf33	-0.242280643	3.20956E-06	1.00881E-05
ELP2P	0.242271247	3.21247E-06	1.00957E-05
RAD52	-0.242237566	3.22289E-06	1.01268E-05
LOC127841	0.242223577	3.22723E-06	1.01389E-05
PPM1K	-0.242189397	3.23786E-06	1.01706E-05
SLC24A5	0.242113802	3.26148E-06	1.02432E-05
WDR75	0.242105647	3.26404E-06	1.02496E-05
SLC25A15	-0.242103721	3.26464E-06	1.02499E-05
RFX3	-0.242094773	3.26745E-06	1.02571E-05
PCOTH	0.242072067	3.27459E-06	1.02779E-05
RAG1	-0.24202873	3.28826E-06	1.03192E-05

NCRNA0017	-0.241978952	3.30402E-06	1.0367E-05
SNRNP40	0.241960787	3.30979E-06	1.03835E-05
LASS4	0.241903105	3.32818E-06	1.04396E-05
POLR1A	-0.241892556	3.33156E-06	1.04485E-05
GMFG	0.241879946	3.3356E-06	1.04595E-05
PADI1	-0.241860317	3.34189E-06	1.04776E-05
FGF1	-0.241794519	3.36307E-06	1.05423E-05
BIVM	-0.241776147	3.36901E-06	1.05593E-05
SLCO3A1	-0.241756139	3.37548E-06	1.05779E-05
RNASE7	-0.241723289	3.38614E-06	1.06097E-05
KBTBD3	0.241708588	3.39092E-06	1.0623E-05
KIAA0495	-0.24169413	3.39563E-06	1.06361E-05
LOC493754	0.24168938	3.39718E-06	1.06392E-05
OR8A1	0.241663426	3.40565E-06	1.06629E-05
TM4SF1	0.241662967	3.4058E-06	1.06629E-05
NFKBIZ	-0.241576075	3.43431E-06	1.07504E-05
MTMR10	-0.241528863	3.44989E-06	1.07975E-05
TGIF2	0.241466855	3.47047E-06	1.08602E-05
MCAM	-0.241423159	3.48503E-06	1.09041E-05
DEFB126	0.241404189	3.49138E-06	1.0921E-05
STAB2	-0.241403473	3.49162E-06	1.0921E-05
RBP2	0.241402039	3.4921E-06	1.0921E-05
SEC63	-0.241386696	3.49723E-06	1.09354E-05
CASP7	-0.241333671	3.51505E-06	1.09894E-05
FAM167A	-0.241318878	3.52003E-06	1.10032E-05
TTLL9	0.241297845	3.52714E-06	1.10237E-05
KIAA1467	-0.241283721	3.53191E-06	1.10369E-05
APOBEC3C	-0.241239871	3.54678E-06	1.10816E-05
SNCA	-0.241234811	3.5485E-06	1.10852E-05
TMED10	-0.241228883	3.55051E-06	1.10898E-05
RNF123	-0.241221627	3.55298E-06	1.10957E-05
BTNL9	-0.241196151	3.56166E-06	1.11211E-05
URGCP	-0.241146657	3.57858E-06	1.11722E-05
MRPS36	0.241137539	3.5817E-06	1.11802E-05
UBXN2B	-0.241124306	3.58624E-06	1.11926E-05
DPYSL3	-0.241110692	3.59092E-06	1.12054E-05
UBE2Z	0.24110603	3.59252E-06	1.12087E-05
GZF1	-0.241095416	3.59617E-06	1.12183E-05
NHEJ1	0.241091108	3.59765E-06	1.12212E-05
ACER3	-0.241067293	3.60586E-06	1.1245E-05
CAPZB	0.24105021	3.61176E-06	1.12617E-05
HTR2A	-0.241045331	3.61345E-06	1.12652E-05
RTN4RL1	-0.24099546	3.63073E-06	1.13173E-05
TMEM209	-0.240886992	3.6686E-06	1.14335E-05
NDUFA10	0.240843163	3.68401E-06	1.14797E-05
CPSF1	0.240839356	3.68535E-06	1.14821E-05

FAM110A	0.240807493	3.69659E-06	1.15153E-05
SNORD1C	-0.240799889	3.69928E-06	1.15219E-05
BCAT2	0.240778733	3.70677E-06	1.15423E-05
TRIM32	-0.240778145	3.70698E-06	1.15423E-05
MED21	-0.240722143	3.72687E-06	1.16024E-05
HIST1H1E	0.240652667	3.75169E-06	1.16779E-05
NEDD1	-0.240629397	3.76004E-06	1.1702E-05
FAM83H	0.24039149	3.84642E-06	1.1969E-05
KCND1	-0.240383071	3.84951E-06	1.19767E-05
CKMT1A	0.240338238	3.86601E-06	1.20262E-05
RAB4B	0.240318365	3.87335E-06	1.20471E-05
CRELD2	0.240287337	3.88483E-06	1.2081E-05
BCAR3	-0.240231431	3.9056E-06	1.21436E-05
DPH2	0.240206438	3.91492E-06	1.21707E-05
COL13A1	-0.240199452	3.91753E-06	1.21769E-05
AGGF1	-0.24004883	3.97417E-06	1.23511E-05
SYTL4	-0.240006336	3.99029E-06	1.23992E-05
GYS2	-0.239974121	4.00256E-06	1.24354E-05
CACNA1D	-0.239950773	4.01147E-06	1.24611E-05
EPN1	0.239944564	4.01384E-06	1.24666E-05
ADSS	-0.239896389	4.0323E-06	1.25219E-05
MCM9	-0.239862294	4.04541E-06	1.25607E-05
SPIRE1	-0.239733933	4.09514E-06	1.27131E-05
CDCA2	0.239691284	4.11179E-06	1.27628E-05
LAMC3	-0.239686981	4.11347E-06	1.27661E-05
KIAA0652	0.239663092	4.12283E-06	1.27914E-05
FAT3	-0.239661733	4.12336E-06	1.27914E-05
THPO	0.23966123	4.12356E-06	1.27914E-05
ALG5	0.239659553	4.12422E-06	1.27914E-05
PGM5	-0.239657732	4.12493E-06	1.27917E-05
TRIM31	0.239644388	4.13017E-06	1.28059E-05
HIST1H2AB	0.239639277	4.13218E-06	1.28101E-05
NSUN6	-0.239591342	4.15106E-06	1.28667E-05
FAM129B	-0.239565603	4.16123E-06	1.28962E-05
DPH3	0.239551136	4.16696E-06	1.29119E-05
TESK2	-0.23952108	4.17888E-06	1.29469E-05
ZNF174	0.239436791	4.21249E-06	1.3049E-05
TRPC5	-0.239415214	4.22114E-06	1.30737E-05
ZNF843	-0.239348864	4.24783E-06	1.31543E-05
FAM46A	-0.239337811	4.25229E-06	1.31661E-05
SNRNP200	-0.239304051	4.26595E-06	1.32063E-05
PRSS45	0.239237143	4.29314E-06	1.32885E-05
AAK1	-0.239214962	4.30219E-06	1.33144E-05
RABEPK	0.239198911	4.30875E-06	1.33326E-05
CTSA	0.239130238	4.33693E-06	1.34177E-05
WDR26	-0.239084001	4.356E-06	1.34735E-05

C1S	-0.239081703	4.35695E-06	1.34735E-05
TTC30A	-0.239081607	4.35699E-06	1.34735E-05
YKT6	0.239045386	4.37199E-06	1.35178E-05
SLC26A2	-0.239018364	4.38321E-06	1.35501E-05
ASB1	0.239016989	4.38378E-06	1.35501E-05
CYR61	-0.238996875	4.39215E-06	1.35738E-05
HSD17B12	-0.238960789	4.40721E-06	1.36182E-05
RBMX	0.238958465	4.40818E-06	1.36191E-05
IKZF5	-0.238931698	4.41938E-06	1.36516E-05
UBE2W	-0.238919972	4.4243E-06	1.36647E-05
SNX30	-0.23889727	4.43383E-06	1.3692E-05
MPV17	0.23886674	4.44668E-06	1.37296E-05
KCNK17	-0.238852035	4.45289E-06	1.37466E-05
CCRL1	-0.2388468	4.4551E-06	1.37513E-05
PLEKHA3	-0.23883623	4.45956E-06	1.37629E-05
SLC11A2	-0.238766149	4.48928E-06	1.38525E-05
AK2	0.238750466	4.49595E-06	1.38709E-05
ZBTB47	-0.238732342	4.50368E-06	1.38926E-05
NEAT1	-0.23870549	4.51515E-06	1.39258E-05
C5orf4	-0.238685469	4.52372E-06	1.39501E-05
TTLL7	-0.238644576	4.54127E-06	1.40021E-05
OLFML1	-0.238641444	4.54262E-06	1.40041E-05
RERG	-0.238616331	4.55344E-06	1.40352E-05
C22orf36	0.238581285	4.56857E-06	1.40797E-05
FOXN2	-0.238534183	4.58899E-06	1.41404E-05
LGALS2	0.238527127	4.59205E-06	1.41477E-05
SLC26A11	0.238497937	4.60476E-06	1.41846E-05
SBDSP1	-0.238491888	4.60739E-06	1.41906E-05
GHDC	0.23848286	4.61133E-06	1.42005E-05
YARS	0.238375819	4.65827E-06	1.43428E-05
SELM	0.238358117	4.66608E-06	1.43647E-05
HNF4G	-0.238210823	4.73152E-06	1.45639E-05
CD1D	-0.238090273	4.78572E-06	1.47284E-05
SMAD4	-0.238072269	4.79387E-06	1.47512E-05
KIAA1586	0.238052994	4.80261E-06	1.47758E-05
NFKBIA	-0.238040805	4.80814E-06	1.47906E-05
C7orf13	0.237927787	4.85973E-06	1.49469E-05
KCNE1L	0.237868921	4.8868E-06	1.50279E-05
DGCR6L	0.237852579	4.89435E-06	1.50488E-05
DHRS11	0.237838717	4.90075E-06	1.50662E-05
NT5DC2	0.237767735	4.93369E-06	1.51651E-05
MARK2	-0.23776136	4.93665E-06	1.51718E-05
S100A13	0.237758387	4.93804E-06	1.51737E-05
BCL6B	-0.237730004	4.95128E-06	1.52121E-05
GBP4	-0.237686859	4.97147E-06	1.52718E-05
OAS2	-0.237671609	4.97862E-06	1.52914E-05

EMCN	-0.23763746	4.99468E-06	1.53383E-05
C20orf177	-0.237615135	5.0052E-06	1.53683E-05
C1orf43	0.237610758	5.00727E-06	1.53722E-05
NOL10	0.237560787	5.03091E-06	1.54424E-05
BSDC1	-0.237522474	5.04911E-06	1.54959E-05
UHRF2	-0.237485982	5.0665E-06	1.55469E-05
RYK	-0.237468284	5.07496E-06	1.55704E-05
KCNMA1	-0.237458151	5.0798E-06	1.55829E-05
CIB2	0.237452459	5.08253E-06	1.55889E-05
ZNF608	-0.237449223	5.08408E-06	1.55912E-05
PTPLAD2	-0.23739514	5.11004E-06	1.56684E-05
ZNF546	-0.237387419	5.11376E-06	1.56774E-05
NAA15	-0.237355016	5.12939E-06	1.57229E-05
ZBTB49	-0.237342651	5.13537E-06	1.57388E-05
LY6G6C	0.237331105	5.14095E-06	1.57535E-05
C18orf2	0.237286634	5.16252E-06	1.58172E-05
IGFBP7	-0.237272227	5.16953E-06	1.58362E-05
TAF3	-0.237263733	5.17366E-06	1.58464E-05
HPDL	0.237215305	5.1973E-06	1.59164E-05
C20orf46	0.237210949	5.19943E-06	1.59202E-05
ETAA1	-0.237209517	5.20013E-06	1.59202E-05
RAD23B	-0.237148065	5.23029E-06	1.601E-05
PTX3	-0.237025118	5.29112E-06	1.61937E-05
IRF8	-0.237004149	5.30157E-06	1.62232E-05
ISM2	0.236948379	5.32944E-06	1.63039E-05
C4orf32	-0.23694813	5.32956E-06	1.63039E-05
PTDSS1	0.236924313	5.34151E-06	1.63379E-05
BMP5	-0.236898092	5.35469E-06	1.63757E-05
ACOT7	0.236866354	5.37068E-06	1.64221E-05
C8orf45	0.236864662	5.37153E-06	1.64222E-05
SELS	0.236836236	5.3859E-06	1.64636E-05
ZNF544	0.236828082	5.39003E-06	1.64737E-05
TAF15	0.236819933	5.39416E-06	1.64838E-05
HSD11B1	-0.236807951	5.40023E-06	1.64998E-05
CDCA8	0.236794426	5.4071E-06	1.65182E-05
GPR179	-0.236738015	5.43582E-06	1.66034E-05
CTAG1B	0.236712265	5.44898E-06	1.66411E-05
PAPD7	-0.236698937	5.45581E-06	1.66594E-05
MYO1A	0.236691322	5.45971E-06	1.66687E-05
PTRF	-0.23667352	5.46884E-06	1.66941E-05
C16orf68	0.236635267	5.48852E-06	1.67495E-05
SLC28A3	-0.236634946	5.48868E-06	1.67495E-05
KCNN2	-0.236532143	5.5419E-06	1.69093E-05
PRDX4	0.236522932	5.54669E-06	1.69213E-05
TNNC2	0.236520167	5.54813E-06	1.69231E-05
THAP2	-0.236505141	5.55595E-06	1.69444E-05

TRIB2	-0.236492279	5.56266E-06	1.69608E-05
FAM192A	0.23649154	5.56305E-06	1.69608E-05
EPHB1	-0.236414541	5.60337E-06	1.70811E-05
SLC9A1	-0.236369144	5.62728E-06	1.71514E-05
NRF1	0.236313371	5.65678E-06	1.72387E-05
C10orf35	0.236287817	5.67034E-06	1.72774E-05
RCE1	0.236222316	5.70526E-06	1.73811E-05
XAF1	-0.236204898	5.71458E-06	1.74068E-05
DOCK11	-0.236157916	5.73978E-06	1.74809E-05
GAGE12J	0.236123038	5.75857E-06	1.75345E-05
MED29	0.236121995	5.75913E-06	1.75345E-05
TMEFF1	-0.236110598	5.76528E-06	1.75505E-05
AHI1	-0.236097371	5.77243E-06	1.75696E-05
NPAS1	0.236077273	5.7833E-06	1.76E-05
SLC25A28	0.236056707	5.79445E-06	1.76312E-05
SLC7A10	0.235993231	5.82899E-06	1.77336E-05
DGCR6	0.235930139	5.86351E-06	1.78359E-05
BTBD1	-0.235819576	5.92448E-06	1.80186E-05
MAGIX	0.235781243	5.94576E-06	1.80806E-05
TINAGL1	-0.235773956	5.94981E-06	1.80901E-05
COPB2	-0.235763558	5.9556E-06	1.8105E-05
NFIA	-0.235708021	5.9866E-06	1.81965E-05
THSD1	-0.235704931	5.98833E-06	1.81989E-05
MYLIP	-0.235654132	6.01684E-06	1.82828E-05
LOC90246	-0.235648906	6.01978E-06	1.82889E-05
CAGE1	0.235629674	6.03061E-06	1.83165E-05
GSK3A	0.235629499	6.03071E-06	1.83165E-05
MAVS	0.235591647	6.05208E-06	1.83786E-05
AFARP1	-0.235568273	6.06531E-06	1.8416E-05
CDR2L	-0.235517902	6.09392E-06	1.85001E-05
ZNF844	-0.235501872	6.10305E-06	1.8525E-05
C9orf139	-0.235477311	6.11707E-06	1.85647E-05
C4orf19	-0.235472928	6.11957E-06	1.85694E-05
GIPC1	0.235417712	6.15121E-06	1.86626E-05
FOXC1	-0.235389444	6.16746E-06	1.87091E-05
SENP3	0.235380717	6.17249E-06	1.87215E-05
IARS2	-0.235363442	6.18245E-06	1.87488E-05
SULF1	-0.235286734	6.22688E-06	1.88807E-05
PCDP1	-0.235279545	6.23105E-06	1.88905E-05
CEP55	0.235248254	6.24927E-06	1.89428E-05
CBX6	-0.235234893	6.25707E-06	1.89636E-05
CSNK1A1L	-0.235227503	6.26138E-06	1.89737E-05
RACGAP1	0.235187186	6.28498E-06	1.90423E-05
TMCO2	0.23516413	6.29851E-06	1.90804E-05
NUDT8	0.235103544	6.3342E-06	1.91856E-05
AMOT	-0.235052941	6.36415E-06	1.92734E-05

PAPPA2	-0.234963944	6.41716E-06	1.9431E-05
PLEKHM1	-0.234955963	6.42193E-06	1.94425E-05
SCAF1	0.234896754	6.45746E-06	1.95471E-05
NSUN5P2	0.23488299	6.46574E-06	1.95692E-05
NDST3	-0.234830901	6.49719E-06	1.96614E-05
RALA	0.234730105	6.55845E-06	1.98438E-05
CBWD2	0.234626155	6.62221E-06	2.00336E-05
FAM119A	0.234607515	6.63371E-06	2.00653E-05
ISG15	0.234557314	6.66476E-06	2.01562E-05
FAM106A	-0.234554665	6.6664E-06	2.01581E-05
NALCN	-0.234547068	6.67111E-06	2.01693E-05
LOC400027	-0.234507948	6.69543E-06	2.02398E-05
TMEM218	0.234478755	6.71363E-06	2.02917E-05
MGST2	0.234411745	6.75559E-06	2.04154E-05
NCRNA0008	-0.234389449	6.76961E-06	2.04547E-05
GPR155	-0.234358853	6.78888E-06	2.05094E-05
ATP13A4	-0.234357431	6.78978E-06	2.05094E-05
INADL	-0.234355482	6.79101E-06	2.051E-05
MFSD10	0.234334796	6.80408E-06	2.05464E-05
CDC42SE1	-0.23429448	6.82961E-06	2.06203E-05
PRAMEF10	-0.234209365	6.88383E-06	2.07809E-05
MT3	0.234194468	6.89336E-06	2.08065E-05
DTX4	-0.23411782	6.9426E-06	2.09519E-05
LOC342346	-0.234046652	6.98861E-06	2.10876E-05
HIST1H4B	0.234021747	7.00478E-06	2.1131E-05
DDT	0.234021247	7.00511E-06	2.1131E-05
EID2B	0.234004641	7.01591E-06	2.11604E-05
ZNF117	-0.233992663	7.02371E-06	2.11807E-05
CALR3	0.233930934	7.06405E-06	2.12991E-05
CENPQ	0.23387043	7.10381E-06	2.14157E-05
DLL3	0.233831878	7.12925E-06	2.14892E-05
JAM3	-0.233800531	7.15E-06	2.15485E-05
SEC22A	0.233742038	7.18887E-06	2.16623E-05
CNOT7	-0.233707478	7.21193E-06	2.17285E-05
GDPD4	-0.233692534	7.22193E-06	2.17554E-05
TERT	0.233678544	7.23129E-06	2.17803E-05
PTTG2	0.233670806	7.23648E-06	2.17926E-05
NLRP12	-0.233641033	7.25647E-06	2.18473E-05
CXCR2	-0.233640502	7.25683E-06	2.18473E-05
MCM10	0.233631793	7.26268E-06	2.18616E-05
COL1A1	-0.233605731	7.28024E-06	2.19111E-05
LOC441294	-0.233580447	7.29731E-06	2.19592E-05
KLF16	0.233513908	7.34241E-06	2.20916E-05
SCD5	-0.233508042	7.3464E-06	2.21002E-05
HRASLS2	0.233471289	7.37144E-06	2.21722E-05
LOC341056	0.23333524	7.46484E-06	2.24498E-05

C9orf40	0.233260652	7.51653E-06	2.26018E-05
RPS27	0.23323865	7.53184E-06	2.26444E-05
MCM2	0.233227255	7.53978E-06	2.26649E-05
MRE11A	-0.233208668	7.55275E-06	2.27004E-05
MOAP1	-0.233204272	7.55582E-06	2.27062E-05
ANXA1	-0.233197244	7.56073E-06	2.27176E-05
PTMS	0.23319115	7.56499E-06	2.27269E-05
NAT6	0.233137177	7.60283E-06	2.28372E-05
PDE11A	-0.233122819	7.61293E-06	2.2864E-05
TFDP2	0.233119903	7.61498E-06	2.28668E-05
TINAG	0.233117482	7.61669E-06	2.28684E-05
ANKRD31	-0.233063163	7.65502E-06	2.29801E-05
SEPX1	0.233040163	7.6713E-06	2.30255E-05
ITGB8	-0.232994296	7.70388E-06	2.31198E-05
UBN1	-0.23297416	7.71822E-06	2.31561E-05
SCXB	0.232974051	7.7183E-06	2.31561E-05
PCDH12	-0.232969642	7.72145E-06	2.3161E-05
FLG2	-0.232968481	7.72227E-06	2.3161E-05
SLC3A2	0.232961947	7.72694E-06	2.31715E-05
CRYBB2	0.232910207	7.76395E-06	2.3279E-05
C3orf49	-0.232897899	7.77278E-06	2.33015E-05
CDKN2D	0.232896475	7.7738E-06	2.33015E-05
MECR	0.232891004	7.77773E-06	2.33098E-05
ZNF45	-0.232843754	7.81173E-06	2.34082E-05
TFEC	-0.232835615	7.8176E-06	2.34222E-05
LOC399815	0.232808678	7.83707E-06	2.3477E-05
C16orf55	0.232801355	7.84236E-06	2.34894E-05
IQSEC2	-0.232759695	7.87258E-06	2.35763E-05
ZNF124	-0.232735728	7.89001E-06	2.36249E-05
NDST2	-0.23269366	7.92069E-06	2.37133E-05
EPB41L2	-0.232672487	7.93618E-06	2.3756E-05
C9orf89	0.232636105	7.96285E-06	2.38323E-05
LIN7C	-0.23261746	7.97656E-06	2.38697E-05
AHCTF1	-0.232586259	7.99954E-06	2.39349E-05
RET	-0.232560165	8.01881E-06	2.3989E-05
KCTD21	-0.232549137	8.02697E-06	2.40098E-05
ECM1	-0.232454213	8.09751E-06	2.42172E-05
HAO2	-0.232430552	8.11519E-06	2.42664E-05
PODN	-0.232415675	8.12632E-06	2.42954E-05
SLC16A6	-0.232414298	8.12735E-06	2.42954E-05
GUCA2A	0.232406395	8.13327E-06	2.43095E-05
DYRK3	-0.232355621	8.17141E-06	2.44198E-05
HOMER2	-0.232335582	8.18651E-06	2.44613E-05
GDPD3	0.23230183	8.21199E-06	2.45337E-05
NETO1	-0.232147117	8.3298E-06	2.4882E-05
ERRFI1	-0.232117034	8.35289E-06	2.49472E-05

SOCS3	-0.232049907	8.40464E-06	2.5098E-05
SAT2	0.232030742	8.41947E-06	2.51385E-05
ECE1	-0.231993647	8.44825E-06	2.52206E-05
TM4SF18	-0.231979324	8.45938E-06	2.52501E-05
FCN2	-0.231949464	8.48264E-06	2.53157E-05
ESYT1	-0.231924088	8.50246E-06	2.53711E-05
HSPA12A	-0.231890107	8.52906E-06	2.54466E-05
GLMN	0.23184828	8.56192E-06	2.55408E-05
IKBKB	-0.231844901	8.56458E-06	2.55449E-05
GALC	-0.231838799	8.56938E-06	2.55554E-05
C9orf5	-0.231827696	8.57813E-06	2.55777E-05
STBD1	-0.231824088	8.58098E-06	2.55824E-05
C4orf27	0.23182158	8.58295E-06	2.55844E-05
ARHGEF35	-0.231769222	8.62435E-06	2.5704E-05
SFRS16	0.231674499	8.69972E-06	2.59247E-05
EDEM2	0.231634856	8.73145E-06	2.60154E-05
UQCRC2	-0.231609237	8.75201E-06	2.60727E-05
SLC25A36	-0.231485275	8.85216E-06	2.63672E-05
SIX5	0.231430137	8.89706E-06	2.64969E-05
RHOT2	0.231412833	8.91119E-06	2.6535E-05
PGD	0.231404266	8.9182E-06	2.65519E-05
DHRS4L2	0.231402491	8.91965E-06	2.65523E-05
FLJ45244	0.231387914	8.93158E-06	2.65838E-05
C19orf63	0.231358063	8.95607E-06	2.66527E-05
ZNF530	0.231348081	8.96427E-06	2.66732E-05
C11orf54	-0.231313661	8.99261E-06	2.67535E-05
SLC25A33	0.231285973	9.01547E-06	2.68175E-05
C19orf39	0.231241821	9.05203E-06	2.69222E-05
DNMBP	-0.231238023	9.05519E-06	2.69276E-05
SNURF	-0.231180089	9.10339E-06	2.70669E-05
TMEM63B	0.231120186	9.1535E-06	2.72118E-05
PGC	0.231113613	9.15901E-06	2.72241E-05
UGT1A5	-0.231097173	9.17282E-06	2.72609E-05
POM121	-0.231095628	9.17411E-06	2.72609E-05
psiTPTE22	-0.231085273	9.18282E-06	2.72827E-05
NGFR	-0.23105796	9.20582E-06	2.73469E-05
B3GAT2	-0.231047503	9.21464E-06	2.73691E-05
ADCY6	-0.231001033	9.25394E-06	2.74817E-05
CLDN20	-0.230978794	9.2728E-06	2.75336E-05
DDX59	-0.230961785	9.28725E-06	2.75724E-05
SACS	-0.23094659	9.30018E-06	2.76067E-05
C13orf37	0.230906831	9.33409E-06	2.77032E-05
PNCK	0.230896263	9.34312E-06	2.77224E-05
C7orf41	-0.230896005	9.34334E-06	2.77224E-05
STK17B	-0.230883732	9.35384E-06	2.77494E-05
PHF20L1	-0.230872355	9.36359E-06	2.77742E-05

POMT1	0.230840448	9.39097E-06	2.78512E-05
PCDHGA3	-0.23074869	9.47013E-06	2.80818E-05
FRAT1	0.230726238	9.48959E-06	2.81354E-05
KDM3A	-0.230680802	9.5291E-06	2.82483E-05
DISC1	-0.230652842	9.55349E-06	2.83164E-05
PDIK1L	-0.230607472	9.5932E-06	2.84298E-05
ALG8	0.230569816	9.62627E-06	2.85236E-05
PRAMEF8	-0.230564975	9.63053E-06	2.8532E-05
ZNF543	-0.23056055	9.63443E-06	2.85393E-05
THRB	-0.230524819	9.66594E-06	2.86284E-05
MEX3A	0.230512519	9.67681E-06	2.86563E-05
CASP8	-0.230493607	9.69354E-06	2.87016E-05
HSPA12B	-0.230491059	9.6958E-06	2.87026E-05
NUBP1	0.230489976	9.69676E-06	2.87026E-05
GFM2	-0.230472805	9.71198E-06	2.87434E-05
CAV1	-0.23046781	9.71641E-06	2.87522E-05
GORASP1	-0.230316841	9.85129E-06	2.9147E-05
C14orf174	-0.230309702	9.85771E-06	2.91617E-05
ZNF566	-0.230302567	9.86414E-06	2.91763E-05
FOSB	-0.230292201	9.87347E-06	2.9198E-05
EXOC7	0.230290724	9.87481E-06	2.9198E-05
ACAD11	-0.230289557	9.87586E-06	2.9198E-05
LYRM5	-0.230230437	9.92929E-06	2.93516E-05
IL17F	0.230177215	9.97762E-06	2.94901E-05
NFE2L1	-0.230143983	1.00079E-05	2.95752E-05
GRM8	-0.230139519	1.0012E-05	2.95829E-05
LOC100124	-0.230125586	1.00247E-05	2.96161E-05
CSF2RB	-0.230058926	1.00858E-05	2.97923E-05
PFKM	-0.230046374	1.00974E-05	2.9822E-05
PPARGC1B	-0.230028546	1.01138E-05	2.98661E-05
NLRP1	-0.230021366	1.01204E-05	2.98812E-05
EPSTI1	-0.229970157	1.01678E-05	3.00166E-05
BCL7A	-0.229946714	1.01895E-05	3.00763E-05
NTRK3	-0.229912745	1.02211E-05	3.01651E-05
C10orf62	0.229888499	1.02437E-05	3.02273E-05
ZNF23	-0.229881376	1.02504E-05	3.02425E-05
UQCRFS1	0.229767246	1.03575E-05	3.0554E-05
JUN	-0.229754528	1.03695E-05	3.05848E-05
ABLIM1	-0.229724718	1.03977E-05	3.06634E-05
RAB9A	-0.229705493	1.04159E-05	3.07126E-05
ARHGAP11B	-0.229699332	1.04218E-05	3.07253E-05
RPL31P11	0.229636596	1.04814E-05	3.08957E-05
TFPI2	-0.229635306	1.04827E-05	3.08957E-05
GBP2	0.229623435	1.0494E-05	3.09245E-05
FAM122B	-0.229520327	1.05929E-05	3.12114E-05
C17orf101	0.229478108	1.06337E-05	3.13268E-05

PANK1	-0.229475263	1.06364E-05	3.13303E-05
ADAMTS10	-0.22946138	1.06499E-05	3.13653E-05
ZNF646	-0.229443909	1.06668E-05	3.14105E-05
C1orf64	0.229387604	1.07215E-05	3.1567E-05
HDAC9	-0.229384125	1.07249E-05	3.15723E-05
RWDD4A	-0.229351647	1.07566E-05	3.1661E-05
ADAM9	-0.22932479	1.07829E-05	3.17337E-05
LOC642597	-0.229270885	1.08359E-05	3.18848E-05
AJAP1	-0.229242925	1.08634E-05	3.19612E-05
EBF3	-0.229221168	1.08849E-05	3.20197E-05
HIC1	-0.229208552	1.08974E-05	3.20517E-05
DEPDC1B	0.229158536	1.0947E-05	3.21929E-05
GALNT1	-0.229141932	1.09635E-05	3.22367E-05
GAB2	-0.229112539	1.09928E-05	3.23181E-05
QRFPR	-0.229110368	1.0995E-05	3.23197E-05
RNASEH1	0.22909216	1.10132E-05	3.23684E-05
PDE3B	-0.229078313	1.10271E-05	3.24043E-05
YIPF1	0.229041651	1.10638E-05	3.25075E-05
LMOD1	-0.229039299	1.10662E-05	3.25097E-05
TIMM44	0.229005646	1.11E-05	3.26043E-05
AMICA1	0.228978367	1.11275E-05	3.26803E-05
ATXN1	-0.228953671	1.11525E-05	3.27488E-05
HLA-A	0.228929308	1.11772E-05	3.28164E-05
LBH	-0.228921139	1.11855E-05	3.28359E-05
ZBTB48	0.228913392	1.11933E-05	3.28541E-05
B3GALTL	-0.228888716	1.12184E-05	3.29229E-05
RIPPLY2	0.228886883	1.12203E-05	3.29235E-05
44076	-0.228865774	1.12418E-05	3.29818E-05
CHRM5	-0.228856678	1.12511E-05	3.30041E-05
RBBP9	-0.228830196	1.12781E-05	3.30786E-05
APOC1	0.228762606	1.13475E-05	3.32771E-05
KIAA1211	-0.228732692	1.13783E-05	3.33625E-05
C12orf48	0.228713823	1.13977E-05	3.34147E-05
TMEM89	0.228663177	1.14502E-05	3.35635E-05
FIGNL2	0.228646891	1.14671E-05	3.36081E-05
GNG4	0.228612306	1.15031E-05	3.37086E-05
LOC338651	-0.228603918	1.15118E-05	3.37293E-05
F3	-0.228597048	1.1519E-05	3.37453E-05
C16orf93	0.228582662	1.1534E-05	3.37843E-05
PEBP4	-0.228533906	1.1585E-05	3.39289E-05
GGTLC2	0.228445692	1.1678E-05	3.41959E-05
LOC339290	-0.228432369	1.1692E-05	3.42322E-05
AMIGO1	-0.228423658	1.17013E-05	3.42541E-05
RAB10	-0.228408966	1.17168E-05	3.42947E-05
SLC39A6	-0.228378634	1.17491E-05	3.43839E-05
JKAMP	-0.228349993	1.17795E-05	3.44652E-05

SLC16A13	-0.228349277	1.17803E-05	3.44652E-05
RFTN1	-0.228336986	1.17934E-05	3.44985E-05
CHRNA6	-0.22831696	1.18148E-05	3.4556E-05
LANCL1	-0.228309359	1.18229E-05	3.45747E-05
FAM72D	0.228304013	1.18286E-05	3.45863E-05
RAB27A	-0.228222872	1.19158E-05	3.4836E-05
C14orf159	-0.228192418	1.19486E-05	3.49221E-05
UGT1A6	0.228192341	1.19487E-05	3.49221E-05
PCM1	-0.228111712	1.20361E-05	3.51724E-05
PRKRIR	-0.228014075	1.21428E-05	3.54789E-05
CCDC3	-0.228010947	1.21463E-05	3.54837E-05
TMEM150A	0.227962651	1.21994E-05	3.56337E-05
EXOSC6	0.227943701	1.22203E-05	3.56895E-05
MED6	0.22781471	1.23634E-05	3.61023E-05
NDE1	-0.227744869	1.24416E-05	3.63253E-05
PCGF5	-0.227671775	1.25239E-05	3.65603E-05
CDH20	-0.227598291	1.26072E-05	3.6798E-05
FGF8	0.227592235	1.26141E-05	3.68127E-05
NPAS3	-0.227545005	1.26679E-05	3.69644E-05
ZBTB7B	-0.227542624	1.26707E-05	3.69669E-05
ARPP21	-0.227533084	1.26816E-05	3.69933E-05
C6	-0.227527902	1.26875E-05	3.70052E-05
GTF2A1L	-0.227491847	1.27288E-05	3.71202E-05
UCK1	0.227483905	1.27379E-05	3.71414E-05
HSD17B7	0.227442345	1.27857E-05	3.72753E-05
SRPK1	0.227355389	1.28863E-05	3.7563E-05
C12orf63	-0.227350705	1.28917E-05	3.75733E-05
ITM2A	-0.227292734	1.29592E-05	3.77629E-05
HIST1H2BD	0.227291592	1.29605E-05	3.77629E-05
ACAA2	-0.227242377	1.30181E-05	3.79251E-05
SPRED3	-0.22720405	1.30631E-05	3.80506E-05
PIK3R5	-0.227186655	1.30836E-05	3.81046E-05
ENTPD1	-0.227180903	1.30904E-05	3.81188E-05
ZSWIM1	0.227150256	1.31265E-05	3.82185E-05
TMBIM6	-0.227134145	1.31456E-05	3.82684E-05
NCSTN	-0.227090371	1.31974E-05	3.84138E-05
PARM1	-0.227073518	1.32175E-05	3.84665E-05
MAPK4	-0.227066436	1.32259E-05	3.84854E-05
POT1	-0.227051907	1.32432E-05	3.85301E-05
CDKN2C	0.227033189	1.32655E-05	3.85894E-05
CROCCL2	-0.227021193	1.32798E-05	3.86254E-05
LOC653566	0.227016785	1.32851E-05	3.86351E-05
ZSWIM7	0.226990518	1.33165E-05	3.87208E-05
PPYR1	-0.226966235	1.33456E-05	3.87998E-05
C17orf103	-0.226933146	1.33854E-05	3.89097E-05
HSD17B10	0.226920603	1.34005E-05	3.89479E-05

CASC2	-0.226876196	1.34541E-05	3.9098E-05
GNAT2	-0.22686994	1.34617E-05	3.91143E-05
PCDHB7	-0.226838894	1.34993E-05	3.92179E-05
H3F3B	0.226826906	1.35139E-05	3.92544E-05
DNMT3A	0.226794465	1.35533E-05	3.93633E-05
SPN	-0.226739365	1.36206E-05	3.95529E-05
BRPF1	-0.226668715	1.37073E-05	3.97989E-05
FAM151B	-0.226643537	1.37383E-05	3.98832E-05
SNORD116-	-0.226624897	1.37613E-05	3.99441E-05
OLFML2A	-0.226621116	1.3766E-05	3.99519E-05
ZNF483	-0.226589836	1.38047E-05	4.00536E-05
TIA1	-0.226589555	1.38051E-05	4.00536E-05
HTR1D	0.226492173	1.39263E-05	4.03993E-05
INSR	-0.226490115	1.39288E-05	4.04009E-05
PDHX	-0.226325057	1.41366E-05	4.09975E-05
ZCCHC10	0.226308464	1.41576E-05	4.10482E-05
LTF	-0.226308034	1.41582E-05	4.10482E-05
FRZB	-0.226291035	1.41798E-05	4.11048E-05
NRBF2	-0.226259748	1.42196E-05	4.12143E-05
RRM2B	-0.226235954	1.425E-05	4.12963E-05
PHLDB3	0.226211832	1.42808E-05	4.13796E-05
PHKB	-0.226181409	1.43198E-05	4.14866E-05
ATL1	-0.226175538	1.43273E-05	4.15024E-05
NDST1	-0.226132478	1.43827E-05	4.16567E-05
SLCO2B1	-0.226084413	1.44448E-05	4.18304E-05
ZDHHC23	-0.226080578	1.44497E-05	4.18387E-05
MOXD1	-0.22598102	1.45791E-05	4.22072E-05
GNL3	0.225944362	1.46271E-05	4.23398E-05
MTHFD1	-0.225905843	1.46776E-05	4.24799E-05
NAALADL1	0.22589783	1.46881E-05	4.24982E-05
HIST1H3B	0.225897771	1.46882E-05	4.24982E-05
PANX3	0.225836518	1.47689E-05	4.27256E-05
P2RY10	-0.225819673	1.47912E-05	4.27838E-05
FAM132A	0.225752255	1.48807E-05	4.30363E-05
DCUN1D4	-0.225730082	1.49102E-05	4.31155E-05
PSEN1	-0.225679562	1.49777E-05	4.33044E-05
GCOM1	-0.225673746	1.49855E-05	4.33207E-05
C9orf140	0.225629827	1.50444E-05	4.34848E-05
ATP6V1B2	-0.225577224	1.51153E-05	4.36833E-05
FAM99B	-0.225575017	1.51183E-05	4.36856E-05
URB2	-0.225573158	1.51208E-05	4.36865E-05
TOP2B	-0.22552698	1.51834E-05	4.38608E-05
SPEF1	0.225503149	1.52157E-05	4.39479E-05
UBE2G1	-0.225459076	1.52757E-05	4.41149E-05
HIST1H2BK	0.225455332	1.52808E-05	4.41232E-05
BTBD3	-0.225445241	1.52946E-05	4.41566E-05

TMEM41A	0.225394364	1.53643E-05	4.43513E-05
FAM86D	0.225359241	1.54125E-05	4.44841E-05
METT11D1	0.225273133	1.55314E-05	4.48209E-05
CLDN12	-0.225246372	1.55686E-05	4.49215E-05
GRLF1	-0.225100478	1.57725E-05	4.55034E-05
LRRC59	0.225062848	1.58255E-05	4.56497E-05
TXNDC6	-0.225060312	1.58291E-05	4.56534E-05
P4HA3	-0.225036666	1.58625E-05	4.57431E-05
MEP1A	0.225019223	1.58872E-05	4.58077E-05
MAP3K10	-0.224997309	1.59183E-05	4.58906E-05
POMZP3	0.224989587	1.59292E-05	4.59155E-05
GAGE2D	0.224976202	1.59482E-05	4.59637E-05
BSCL2	0.224970933	1.59557E-05	4.5977E-05
HPS6	0.224969717	1.59575E-05	4.5977E-05
TMEM40	0.224953533	1.59805E-05	4.60366E-05
PSMB8	0.224940067	1.59997E-05	4.60853E-05
PALB2	-0.224927442	1.60177E-05	4.61305E-05
C12orf56	0.224870011	1.60998E-05	4.63604E-05
CD99	0.224860497	1.61135E-05	4.6393E-05
SLC44A4	0.224813996	1.61804E-05	4.65788E-05
TMEM205	0.22478036	1.62289E-05	4.67118E-05
TYMS	0.224764675	1.62516E-05	4.67703E-05
BZW1	-0.224743399	1.62824E-05	4.68522E-05
MELK	0.2247306	1.6301E-05	4.68989E-05
TMEM216	0.224727323	1.63057E-05	4.69058E-05
ARHGDIA	0.224718017	1.63192E-05	4.69379E-05
LRIT3	-0.224706557	1.63359E-05	4.6979E-05
BUB1	0.224685665	1.63663E-05	4.70596E-05
SPATA6	-0.224550802	1.65639E-05	4.76209E-05
RBM23	-0.224522572	1.66055E-05	4.77337E-05
CDH19	-0.224478286	1.66711E-05	4.79152E-05
FBXO33	-0.22443607	1.67338E-05	4.80884E-05
ZDHHC24	0.224421675	1.67552E-05	4.8143E-05
SS18	-0.224364638	1.68403E-05	4.83808E-05
TMEM52	0.224355834	1.68535E-05	4.84117E-05
WHAMM	-0.224349433	1.68631E-05	4.84322E-05
TAOK2	-0.224315992	1.69133E-05	4.85693E-05
FCER1A	-0.224274952	1.69751E-05	4.87397E-05
SGK3	-0.224235428	1.70348E-05	4.89041E-05
CARKD	-0.224230441	1.70423E-05	4.89187E-05
GAGE4	0.224213605	1.70678E-05	4.89849E-05
TOM1L2	-0.224156945	1.71539E-05	4.92249E-05
PITRM1	-0.224145233	1.71718E-05	4.9269E-05
SPIN2B	0.224106054	1.72316E-05	4.94336E-05
FMOD	-0.224090616	1.72553E-05	4.94942E-05
RG9MTD1	0.22407727	1.72757E-05	4.95457E-05

ANAPC13	0.224047752	1.7321E-05	4.96686E-05
TRIM61	-0.224042951	1.73284E-05	4.96826E-05
NOMO2	-0.224023849	1.73578E-05	4.97597E-05
ZCCHC6	-0.224004245	1.7388E-05	4.98392E-05
VCX3A	0.223967473	1.74448E-05	4.99948E-05
TIGD4	-0.223954458	1.7465E-05	5.00454E-05
GCC1	-0.223807367	1.76943E-05	5.06952E-05
NFX1	-0.223769833	1.77533E-05	5.08568E-05
ERO1LB	-0.223757888	1.77721E-05	5.09034E-05
ACSM5	-0.22373367	1.78103E-05	5.10054E-05
FANCD2	0.22372686	1.7821E-05	5.10289E-05
CAPN1	-0.223701653	1.78609E-05	5.11356E-05
NAT10	0.223696533	1.7869E-05	5.11515E-05
SAP30	0.223601025	1.80208E-05	5.15787E-05
LRP1	-0.223584937	1.80465E-05	5.16448E-05
INTS4L1	-0.223507346	1.81709E-05	5.19934E-05
RNF128	-0.223505411	1.8174E-05	5.19948E-05
TMEM90B	-0.2234279	1.82992E-05	5.23453E-05
USP18	-0.223401308	1.83423E-05	5.24611E-05
ATP5F1	0.223345571	1.8433E-05	5.27128E-05
PARP8	-0.223330295	1.84579E-05	5.27765E-05
LOC389458	0.223295214	1.85152E-05	5.2933E-05
FNDC5	-0.223289389	1.85248E-05	5.29527E-05
C14orf145	-0.223287162	1.85284E-05	5.29555E-05
ARMC1	-0.223274699	1.85489E-05	5.30063E-05
FAM172A	-0.223253609	1.85835E-05	5.30976E-05
CLSTN2	-0.223248519	1.85919E-05	5.31139E-05
SP5	0.223210874	1.86538E-05	5.32833E-05
CCL27	0.223192623	1.8684E-05	5.3356E-05
PRKACB	-0.223192211	1.86847E-05	5.3356E-05
COLEC10	-0.22315933	1.8739E-05	5.35037E-05
FAM76A	-0.223157531	1.8742E-05	5.35045E-05
YWHAE	0.223132493	1.87835E-05	5.36153E-05
FAM189A1	-0.223111545	1.88184E-05	5.3707E-05
ZNF785	-0.223093045	1.88491E-05	5.37872E-05
C3orf22	0.223047648	1.89249E-05	5.39956E-05
ZNF696	0.223018864	1.89731E-05	5.41253E-05
APLF	-0.222991203	1.90195E-05	5.425E-05
SF3A1	-0.222962081	1.90685E-05	5.43819E-05
ASPRV1	-0.222930179	1.91223E-05	5.45275E-05
C21orf7	-0.222872357	1.92201E-05	5.47987E-05
UGT2A3	-0.222854308	1.92508E-05	5.48782E-05
HIST1H2BN	0.222844973	1.92667E-05	5.49156E-05
OSTC	0.22281533	1.93171E-05	5.50514E-05
GCK	-0.222813745	1.93198E-05	5.50514E-05
CTDSP2	-0.222794956	1.93519E-05	5.51349E-05

LOC390595	0.222778088	1.93807E-05	5.52091E-05
CDKN1A	-0.222775295	1.93855E-05	5.52148E-05
C21orf119	0.222744396	1.94384E-05	5.53577E-05
RMI1	-0.222716205	1.94868E-05	5.54876E-05
SENP1	-0.222696473	1.95208E-05	5.55763E-05
MARS	0.222633255	1.96299E-05	5.5879E-05
SNHG10	0.222609007	1.96719E-05	5.59906E-05
ARHGAP27	-0.222596569	1.96935E-05	5.6044E-05
CGNL1	-0.222570007	1.97397E-05	5.61674E-05
TICAM1	-0.222533728	1.98029E-05	5.63393E-05
LOC441454	-0.222510926	1.98427E-05	5.64445E-05
BNIPL	0.222449349	1.99507E-05	5.67435E-05
TREM2	0.222374595	2.00825E-05	5.71103E-05
GALNT2	-0.222308249	2.02002E-05	5.74367E-05
FGF12	-0.222286458	2.0239E-05	5.75388E-05
RAB11FIP3	-0.222280421	2.02497E-05	5.75612E-05
PTPN22	-0.222239313	2.03232E-05	5.77616E-05
FAM153A	-0.222236458	2.03283E-05	5.77679E-05
VPS33B	0.222214963	2.03668E-05	5.7869E-05
FAM108A1	0.222146192	2.04904E-05	5.8212E-05
CCDC50	-0.222136191	2.05084E-05	5.82549E-05
DNAJB12	0.222133338	2.05136E-05	5.82613E-05
LASS1	0.222118161	2.0541E-05	5.83308E-05
HOXB2	-0.222084408	2.06021E-05	5.84959E-05
NPB	0.222075733	2.06178E-05	5.85322E-05
MSH3	-0.222040727	2.06814E-05	5.87043E-05
ITGA2	-0.222030107	2.07007E-05	5.87508E-05
CCDC24	0.222006777	2.07432E-05	5.88631E-05
C4orf44	0.221995796	2.07632E-05	5.89115E-05
PCDHA12	-0.221943614	2.08587E-05	5.9174E-05
VGF	0.221934312	2.08758E-05	5.92139E-05
CFHR4	-0.221913188	2.09146E-05	5.93155E-05
DNM1L	-0.221893497	2.09508E-05	5.94098E-05
CCDC106	0.221886458	2.09638E-05	5.94381E-05
LIPA	-0.221839557	2.10503E-05	5.96751E-05
NAPSA	0.221782374	2.11563E-05	5.99671E-05
COBL	-0.221752347	2.12122E-05	6.01169E-05
TMEM97	0.221742757	2.12301E-05	6.0159E-05
CTBP2	-0.221740376	2.12345E-05	6.0163E-05
SCRIB	0.221735463	2.12437E-05	6.01804E-05
DUSP7	-0.221727526	2.12585E-05	6.02138E-05
USP2	-0.221699581	2.13107E-05	6.03532E-05
CYP26A1	-0.221693392	2.13223E-05	6.03774E-05
MSL3	-0.221688825	2.13309E-05	6.0393E-05
CSTF1	0.22156745	2.15593E-05	6.10312E-05
ASTN1	-0.221557763	2.15777E-05	6.10691E-05

CARD18	0.221557143	2.15789E-05	6.10691E-05
PCDH19	-0.221543138	2.16054E-05	6.11355E-05
KRTAP5-10	0.221541296	2.16089E-05	6.11366E-05
ARL6	-0.221458879	2.17657E-05	6.15715E-05
GPD1	-0.221420128	2.18398E-05	6.17724E-05
PHOSPHO2	0.221414988	2.18496E-05	6.17914E-05
ZC3H4	-0.221398287	2.18816E-05	6.18732E-05
YTHDF3	-0.221365721	2.19442E-05	6.20413E-05
ZCCHC4	-0.221358698	2.19577E-05	6.20707E-05
USP6	-0.221343961	2.19861E-05	6.21421E-05
OMG	-0.221307868	2.20558E-05	6.23302E-05
GPRC5A	-0.221291219	2.2088E-05	6.24124E-05
SLC16A7	-0.221265598	2.21376E-05	6.25438E-05
MNT	-0.221211489	2.22429E-05	6.28321E-05
GNA12	-0.221181537	2.23013E-05	6.29883E-05
PSORS1C3	0.221097183	2.24667E-05	6.34463E-05
LRRC43	-0.221083387	2.24938E-05	6.3514E-05
TRAF1	-0.221075104	2.25101E-05	6.35511E-05
GOT2	-0.221035633	2.25881E-05	6.3762E-05
SELE	-0.220995609	2.26673E-05	6.39767E-05
CATSPERB	-0.220984752	2.26889E-05	6.40285E-05
HPCAL4	-0.220973079	2.27121E-05	6.40848E-05
TTC28	-0.220931836	2.27942E-05	6.43074E-05
SPNS2	-0.220898542	2.28607E-05	6.44859E-05
TMEM135	-0.220852213	2.29535E-05	6.47386E-05
TRMT6	0.220840442	2.29772E-05	6.47961E-05
TMEM43	-0.220824569	2.30091E-05	6.48769E-05
TEKT4	0.22076336	2.31326E-05	6.52159E-05
FAM46C	-0.220717649	2.32252E-05	6.54677E-05
PRPSAP1	0.220701526	2.32579E-05	6.55508E-05
PDHA2	0.220651699	2.33594E-05	6.58275E-05
EGFLAM	-0.220601195	2.34627E-05	6.61093E-05
ZC3H3	0.22056525	2.35365E-05	6.63078E-05
WBSCR17	-0.220502859	2.36651E-05	6.66607E-05
C13orf26	-0.220500381	2.36703E-05	6.66657E-05
KIAA1804	-0.220472864	2.37272E-05	6.68166E-05
OR1L8	-0.220421956	2.38329E-05	6.71048E-05
SFXN1	-0.220410379	2.3857E-05	6.71631E-05
DDX17	-0.22040703	2.3864E-05	6.71733E-05
COL8A2	-0.220389388	2.39007E-05	6.72673E-05
CLEC1B	-0.2203353	2.40138E-05	6.7576E-05
TOR1A	-0.220268459	2.41543E-05	6.79576E-05
KCNS2	-0.220267524	2.41562E-05	6.79576E-05
FLJ10357	-0.220205447	2.42874E-05	6.83169E-05
LOC284232	-0.220191288	2.43174E-05	6.83916E-05
LMF2	0.22017214	2.4358E-05	6.84962E-05

MAGEL2	-0.220152749	2.43993E-05	6.86022E-05
C17orf51	-0.220151165	2.44026E-05	6.86022E-05
UPP1	0.220122495	2.44637E-05	6.87642E-05
ZNF572	0.220111859	2.44864E-05	6.88183E-05
ANKRD57	-0.220103054	2.45052E-05	6.88614E-05
GIT1	0.220095023	2.45223E-05	6.88999E-05
CCL23	-0.220065157	2.45863E-05	6.90697E-05
SEC14L3	-0.22003192	2.46576E-05	6.92603E-05
PARP12	0.220023123	2.46765E-05	6.93036E-05
CCDC130	0.220006457	2.47123E-05	6.93946E-05
DAPK3	0.219999899	2.47265E-05	6.94244E-05
RALGPS2	-0.219995706	2.47355E-05	6.944E-05
CD7	0.219942156	2.48512E-05	6.97549E-05
LRDD	0.219910629	2.49195E-05	6.99368E-05
SAGE1	0.21986752	2.50132E-05	7.019E-05
PBX2	0.219845316	2.50616E-05	7.03159E-05
PROS1	-0.219831832	2.50911E-05	7.03886E-05
TRPC6	-0.219828544	2.50982E-05	7.03988E-05
SPON2	0.219820005	2.51169E-05	7.04412E-05
LOC284900	0.219744722	2.5282E-05	7.08943E-05
OCRL	-0.219714974	2.53475E-05	7.10681E-05
C6orf217	-0.2197118	2.53546E-05	7.10777E-05
HDHD3	0.219675662	2.54344E-05	7.12915E-05
KIAA1199	-0.219600162	2.5602E-05	7.17511E-05
C9orf68	-0.219597253	2.56084E-05	7.17591E-05
LXN	-0.219550604	2.57125E-05	7.20407E-05
RRP7B	0.219526877	2.57656E-05	7.21793E-05
SC5DL	-0.21950443	2.5816E-05	7.23101E-05
C19orf46	0.219432208	2.59785E-05	7.27552E-05
ADCYAP1R1	-0.219400582	2.605E-05	7.29451E-05
ACTL6B	0.219392626	2.6068E-05	7.29853E-05
CLEC18A	0.219339974	2.61875E-05	7.33096E-05
USP48	-0.219322977	2.62262E-05	7.34076E-05
C6orf138	-0.21931312	2.62487E-05	7.34506E-05
ZNF625	-0.219312994	2.6249E-05	7.34506E-05
PFN4	0.219268032	2.63517E-05	7.37276E-05
BIN1	0.219265358	2.63578E-05	7.37344E-05
NECAP2	0.219243064	2.64088E-05	7.38669E-05
XPOT	-0.21921001	2.64847E-05	7.40687E-05
TGFA	-0.219143702	2.66376E-05	7.44858E-05
WSB1	-0.219129714	2.667E-05	7.45658E-05
RNASEL	-0.219107419	2.67216E-05	7.46997E-05
KIAA0753	-0.219094784	2.67509E-05	7.47711E-05
SPEG	-0.2190804	2.67843E-05	7.4854E-05
DTL	0.219052637	2.68489E-05	7.5024E-05
FASTKD5	-0.218917424	2.71656E-05	7.58982E-05

COQ3	0.218875473	2.72645E-05	7.6164E-05
TCF7L2	-0.218873347	2.72696E-05	7.61674E-05
NLRP3	-0.218851715	2.73207E-05	7.62904E-05
ZNF709	-0.218851486	2.73213E-05	7.62904E-05
CCDC14	-0.218849125	2.73269E-05	7.62954E-05
XRCC1	0.218830997	2.73698E-05	7.64046E-05
GLIPR1L2	-0.218764481	2.7528E-05	7.68354E-05
SFT2D2	0.218735269	2.75978E-05	7.70193E-05
SERPINB1	0.218709563	2.76593E-05	7.71802E-05
CYP4F3	-0.218674856	2.77426E-05	7.74017E-05
KIAA1409	-0.218633175	2.78429E-05	7.76706E-05
MAT2B	-0.21859664	2.79311E-05	7.79058E-05
TMEM39A	-0.218594828	2.79355E-05	7.79071E-05
SNORA74B	0.218528415	2.80965E-05	7.83392E-05
PTPN5	-0.218527681	2.80983E-05	7.83392E-05
FAM155A	-0.218516472	2.81255E-05	7.84023E-05
CCDC104	0.218515145	2.81288E-05	7.84023E-05
OLFM2	-0.218422814	2.83544E-05	7.902E-05
RSAD2	-0.218390462	2.84338E-05	7.92303E-05
HIBADH	-0.218373556	2.84754E-05	7.93352E-05
ERCC6L	0.218360502	2.85076E-05	7.94137E-05
FCAR	-0.218353015	2.8526E-05	7.9454E-05
ITGA3	-0.218335153	2.85701E-05	7.95657E-05
FLCN	-0.218328432	2.85867E-05	7.96008E-05
TRIL	-0.218317	2.8615E-05	7.96683E-05
CA13	-0.218296099	2.86667E-05	7.98013E-05
HOXD9	0.218274234	2.8721E-05	7.99411E-05
LOC619207	-0.21826535	2.8743E-05	7.99913E-05
KIF20A	0.218247401	2.87877E-05	8.01043E-05
PHTF1	-0.218243208	2.87981E-05	8.01221E-05
ABCA10	-0.218223339	2.88476E-05	8.02486E-05
NDUFV1	0.218220369	2.8855E-05	8.0258E-05
SLC25A34	-0.218182921	2.89485E-05	8.05069E-05
CUZD1	0.218144999	2.90435E-05	8.07598E-05
C6orf27	-0.21807807	2.92119E-05	8.12166E-05
TMEM181	-0.218063074	2.92497E-05	8.13105E-05
RCC1	0.218027424	2.93399E-05	8.15498E-05
QSER1	-0.218011866	2.93794E-05	8.1648E-05
DYDC2	0.218007583	2.93902E-05	8.16668E-05
ACOX1	-0.217992501	2.94285E-05	8.17618E-05
MAP3K9	-0.217951414	2.95331E-05	8.20409E-05
MYLK4	-0.217937315	2.9569E-05	8.21293E-05
NRN1	-0.217931961	2.95827E-05	8.21558E-05
AKAP3	-0.217909646	2.96397E-05	8.23027E-05
FLJ36031	-0.217906748	2.96471E-05	8.23118E-05
AFAP1L1	-0.217886477	2.9699E-05	8.24444E-05

DCAF7	-0.217876294	2.97251E-05	8.25053E-05
GBA3	-0.217872616	2.97346E-05	8.252E-05
SNX27	-0.217861526	2.9763E-05	8.25875E-05
ZNF324	-0.217856732	2.97753E-05	8.26102E-05
DARC	-0.217821406	2.98662E-05	8.28507E-05
MFSD2A	-0.217807316	2.99025E-05	8.29399E-05
COPS2	-0.217785935	2.99577E-05	8.30814E-05
MAPRE3	-0.217782483	2.99666E-05	8.30946E-05
FAM109A	0.217778386	2.99772E-05	8.31124E-05
LCN12	0.217765971	3.00093E-05	8.31898E-05
AIF1	0.217736664	3.00853E-05	8.33887E-05
ABCA7	-0.217704147	3.01697E-05	8.36008E-05
C10orf84	0.217703978	3.01702E-05	8.36008E-05
PAPOLA	-0.217684191	3.02217E-05	8.37319E-05
HOOK1	-0.21761876	3.03926E-05	8.41937E-05
EPHX2	-0.21759992	3.0442E-05	8.43187E-05
ZNF786	-0.21757246	3.05141E-05	8.45067E-05
GSDMB	0.217516065	3.06627E-05	8.49065E-05
KRTAP5-6	-0.217463161	3.08027E-05	8.52806E-05
DNM1P35	0.217461781	3.08064E-05	8.52806E-05
KCNJ12	-0.217293894	3.12548E-05	8.651E-05
CABC1	0.21728059	3.12906E-05	8.6597E-05
KIAA0408	-0.217274351	3.13074E-05	8.66315E-05
CNN3	-0.217197727	3.15144E-05	8.71923E-05
EBPL	0.217189718	3.15361E-05	8.72326E-05
C1orf223	0.21718912	3.15378E-05	8.72326E-05
C11orf61	-0.217180704	3.15606E-05	8.72836E-05
GOLT1A	0.217177104	3.15704E-05	8.72985E-05
OR56B4	-0.217128748	3.17019E-05	8.76501E-05
BRAP	0.217088497	3.18118E-05	8.79417E-05
SIDT2	-0.217075408	3.18476E-05	8.80285E-05
PMEPA1	-0.217037122	3.19526E-05	8.83064E-05
RGS4	-0.217025524	3.19845E-05	8.83822E-05
UBC	0.216950776	3.21906E-05	8.89394E-05
RBMXL1	-0.216941421	3.22164E-05	8.89985E-05
KISS1R	0.21693884	3.22236E-05	8.90059E-05
FAM159A	0.216929957	3.22482E-05	8.90615E-05
DMGDH	-0.21691794	3.22815E-05	8.91411E-05
EHHADH	-0.216869817	3.24152E-05	8.94979E-05
AKAP7	-0.216848801	3.24737E-05	8.96471E-05
PRPF3	0.216846313	3.24807E-05	8.96538E-05
ZCCHC24	-0.216843634	3.24881E-05	8.96621E-05
NFYC	0.216813042	3.25736E-05	8.98854E-05
CATSPER1	0.216804174	3.25984E-05	8.99414E-05
C21orf56	0.216794668	3.2625E-05	9.00024E-05
PPIG	-0.216783027	3.26576E-05	9.00799E-05

PNPLA7	-0.216704094	3.28796E-05	9.06796E-05
BOC	-0.216657613	3.3011E-05	9.10294E-05
RARG	-0.216651635	3.30279E-05	9.10635E-05
KIAA0427	-0.216636642	3.30705E-05	9.11681E-05
FGD2	-0.216623118	3.31088E-05	9.12613E-05
ZCCHC3	0.216576584	3.32412E-05	9.16135E-05
CTSL2	0.216547851	3.33233E-05	9.18268E-05
RNF149	0.216531227	3.33708E-05	9.19451E-05
ZNF623	-0.216460358	3.35741E-05	9.24926E-05
HEATR4	-0.21640009	3.3748E-05	9.29587E-05
INCENP	-0.216395501	3.37613E-05	9.29758E-05
REG4	0.216394716	3.37636E-05	9.29758E-05
PTBP1	0.216359962	3.38642E-05	9.32402E-05
LMAN2L	0.216352113	3.3887E-05	9.329E-05
CABYR	0.216323512	3.39701E-05	9.35059E-05
CDRT15P	-0.216317939	3.39864E-05	9.35377E-05
NKX3-1	-0.216272141	3.41199E-05	9.38923E-05
GNPDA1	0.216257034	3.41641E-05	9.40009E-05
MGC27382	-0.216237448	3.42215E-05	9.41456E-05
TSNARE1	0.216221181	3.42692E-05	9.42638E-05
GABRD	0.216189156	3.43632E-05	9.45096E-05
DCTN1	0.21618579	3.43731E-05	9.45238E-05
PART1	0.21617784	3.43965E-05	9.45751E-05
TTC15	0.216168456	3.44242E-05	9.4638E-05
ZMPSTE24	-0.216146176	3.44899E-05	9.48056E-05
FSHR	-0.216103992	3.46146E-05	9.51353E-05
LRRC33	-0.216089864	3.46565E-05	9.52372E-05
DGKI	-0.21605149	3.47705E-05	9.55373E-05
TGM2	-0.216043787	3.47934E-05	9.5587E-05
ATG2A	-0.216035374	3.48184E-05	9.56427E-05
PSMD5	-0.21603244	3.48272E-05	9.56535E-05
MEGF6	-0.216029965	3.48346E-05	9.56606E-05
TMEM128	-0.216024886	3.48497E-05	9.56889E-05
HNRNPD	0.215942785	3.50953E-05	9.63499E-05
H1FX	0.215940908	3.51009E-05	9.63521E-05
MEF2B	0.215934868	3.5119E-05	9.63886E-05
LOC144571	0.215907956	3.51999E-05	9.65973E-05
ABCG4	-0.215896969	3.5233E-05	9.66748E-05
AEBP1	-0.215842825	3.53965E-05	9.71098E-05
PRSS23	-0.215837533	3.54125E-05	9.71404E-05
MYL4	0.21576515	3.56322E-05	9.77296E-05
CLCN6	-0.215747688	3.56854E-05	9.78621E-05
PKNOX2	-0.215641865	3.60094E-05	9.8737E-05
TBCD	0.215625214	3.60606E-05	9.88638E-05
STX16	-0.215620343	3.60756E-05	9.88913E-05
SRXN1	0.215510588	3.64152E-05	9.98084E-05

SLC38A4	-0.215502597	3.644E-05	9.98628E-05
SH3PXD2A	-0.215500402	3.64468E-05	9.98677E-05
SLC25A10	0.215491064	3.64759E-05	9.99336E-05
C1orf101	-0.215433221	3.66563E-05	0.000100414
PRDX3	-0.215430038	3.66663E-05	0.000100428
TLR5	-0.215412238	3.6722E-05	0.000100566
TAF4B	-0.215402463	3.67526E-05	0.000100636
TRIM34	-0.215376444	3.68343E-05	0.000100846
MESDC2	0.215365618	3.68683E-05	0.000100925
LOC285733	0.215342323	3.69416E-05	0.000101112
C17orf86	0.215321624	3.70069E-05	0.00010127
BOP1	0.215320842	3.70093E-05	0.00010127
MAD2L1	0.215266643	3.71807E-05	0.000101725
BTG2	-0.215200052	3.73924E-05	0.00010229
DEM1	0.215175438	3.74709E-05	0.00010249
EVI5L	-0.215144166	3.75708E-05	0.00010275
PAPSS2	-0.215096419	3.77239E-05	0.000103154
PLCE1	-0.21505729	3.78499E-05	0.000103484
RGS16	-0.215042573	3.78973E-05	0.0001036
KIF3B	-0.215032815	3.79288E-05	0.000103672
UGT1A4	-0.214970825	3.81295E-05	0.000104206
SLC22A1	-0.214935883	3.82431E-05	0.000104502
LIPT2	0.214927744	3.82696E-05	0.00010456
WDR66	-0.214914148	3.83139E-05	0.000104667
C20orf12	-0.214905708	3.83414E-05	0.000104728
CHMP4C	0.214816657	3.8633E-05	0.00010551
ZNF445	-0.214809307	3.86572E-05	0.000105561
ZNF518B	-0.21479393	3.87078E-05	0.000105685
KCNMB3	0.214765627	3.88011E-05	0.000105925
LPCAT1	0.214681452	3.90797E-05	0.000106671
BEX2	0.214668652	3.91223E-05	0.000106773
C6orf89	0.214629915	3.92513E-05	0.00010711
FAM133A	0.214572938	3.94418E-05	0.000107608
OGDHL	-0.214572103	3.94446E-05	0.000107608
TMX2	0.214568308	3.94573E-05	0.000107628
ATAD1	-0.214556673	3.94964E-05	0.00010772
VCX	0.214482185	3.9747E-05	0.000108389
UNC45B	-0.214472757	3.97789E-05	0.000108461
HIST4H4	-0.214454155	3.98418E-05	0.00010861
CHUK	-0.214453349	3.98445E-05	0.00010861
ACVR1C	-0.214441578	3.98843E-05	0.000108704
PDIA2	0.214405123	4.0008E-05	0.000109026
WAC	-0.214381577	4.0088E-05	0.000109229
CCM2	0.214377674	4.01013E-05	0.00010925
MFAP4	-0.214289692	4.04019E-05	0.000110054
DHX37	0.214259075	4.0507E-05	0.000110325

PLD1	-0.214250173	4.05376E-05	0.000110393
BCAM	0.214198502	4.07156E-05	0.000110863
STOML1	0.214109852	4.10229E-05	0.000111684
ACTC1	-0.214053062	4.12208E-05	0.000112208
PTGER2	-0.214041903	4.12598E-05	0.000112299
C10orf114	0.214021688	4.13306E-05	0.000112476
B4GALT6	-0.214016405	4.13491E-05	0.000112511
IL9R	-0.213978631	4.14817E-05	0.000112856
MED17	-0.213956984	4.15578E-05	0.000113048
KIAA1826	-0.213894612	4.1778E-05	0.000113631
ART1	-0.213883835	4.18161E-05	0.000113719
SH3GL1	0.21386873	4.18696E-05	0.00011385
CWF19L2	-0.213863373	4.18886E-05	0.000113886
MITD1	0.21386019	4.18999E-05	0.000113901
SS18L1	-0.21385818	4.19071E-05	0.000113905
FNDC3B	-0.213832265	4.19991E-05	0.000114139
FUT8	-0.213828757	4.20116E-05	0.000114158
PNMAL2	-0.213773346	4.22091E-05	0.000114679
DIAPH2	-0.213752423	4.2284E-05	0.000114866
SCN4A	-0.21371324	4.24244E-05	0.000115232
INPPL1	-0.21370471	4.2455E-05	0.0001153
CBX5	-0.213696292	4.24853E-05	0.000115366
ELF4	-0.213668516	4.25853E-05	0.000115622
KCNH1	-0.213639766	4.2689E-05	0.000115887
MPND	0.21359471	4.2852E-05	0.000116314
PPP4R4	-0.213576964	4.29163E-05	0.000116473
NEBL	-0.213538326	4.30568E-05	0.000116838
MYOZ2	-0.213510734	4.31573E-05	0.000117095
C2CD4D	-0.213494957	4.32149E-05	0.000117235
EIF3L	0.213481529	4.3264E-05	0.000117351
C11orf86	0.213480084	4.32693E-05	0.000117351
WDR78	-0.213462664	4.33331E-05	0.000117508
PECI	0.21343576	4.34317E-05	0.000117759
CSMD2	-0.213409054	4.35298E-05	0.000118009
LNX1	-0.213402137	4.35553E-05	0.000118062
CNP	0.213340309	4.37834E-05	0.000118664
MLF1IP	0.213332836	4.38111E-05	0.000118723
MFAP5	-0.213308923	4.38996E-05	0.000118947
AK5	-0.213282243	4.39987E-05	0.000119199
DNASE1L3	-0.21325427	4.41027E-05	0.000119465
ERCC5	-0.213117517	4.46148E-05	0.000120836
TNFSF12-T	0.213093585	4.4705E-05	0.000121063
GPR64	-0.213083911	4.47415E-05	0.000121146
ACAD9	0.21296177	4.52048E-05	0.000122384
BCAS4	0.212952106	4.52417E-05	0.000122467
AQP7P3	0.212905312	4.54206E-05	0.000122934

PPP2R1B	-0.212885639	4.54959E-05	0.000123122
SLC39A3	0.212862155	4.55861E-05	0.000123349
CRIP3	0.212849647	4.56342E-05	0.000123462
ERMP1	-0.212840318	4.56701E-05	0.000123542
OR5K2	-0.212823707	4.5734E-05	0.000123699
CCDC122	-0.212814788	4.57684E-05	0.000123775
KCNJ13	-0.212812878	4.57758E-05	0.000123778
ATXN7L2	0.212786978	4.58758E-05	0.000124032
RBM44	-0.212741414	4.60523E-05	0.000124492
FOXP3	-0.212735359	4.60757E-05	0.000124538
GOLGA8A	-0.212711968	4.61666E-05	0.000124751
RENBP	0.212711929	4.61668E-05	0.000124751
DCTD	-0.212706554	4.61877E-05	0.00012479
ALDH1A2	-0.21264599	4.64238E-05	0.000125411
RRAGB	-0.212634348	4.64694E-05	0.000125517
RAD17	-0.212593163	4.66308E-05	0.000125936
RHBG	0.212562285	4.67521E-05	0.000126247
ZNF695	0.212545911	4.68166E-05	0.000126404
KCND2	-0.212525406	4.68975E-05	0.000126605
UPK3A	0.212494143	4.7021E-05	0.000126906
RNU11	0.212493961	4.70217E-05	0.000126906
LRRC1	0.212483129	4.70646E-05	0.000127004
ITPR2	-0.212478171	4.70842E-05	0.00012704
PTGIS	-0.212438822	4.72404E-05	0.000127444
LIMD2	0.212432705	4.72647E-05	0.000127492
ANK1	-0.212420908	4.73116E-05	0.000127602
MRPL46	0.212402323	4.73856E-05	0.000127784
VAV2	-0.212336246	4.76497E-05	0.000128479
TTLL4	0.212317401	4.77252E-05	0.000128665
SLC39A4	0.212299962	4.77952E-05	0.000128836
FAM66C	-0.212253316	4.7983E-05	0.000129325
HARS2	0.212247846	4.80051E-05	0.000129367
CAPN2	-0.212231082	4.80727E-05	0.000129532
EID2	0.212205972	4.81743E-05	0.000129788
CADPS2	-0.21220245	4.81885E-05	0.000129809
ADAMTS9	-0.212190792	4.82358E-05	0.000129914
C18orf1	-0.212189587	4.82407E-05	0.000129914
NFKB2	0.212184596	4.82609E-05	0.000129951
TMEM27	-0.212180265	4.82785E-05	0.00012998
ODZ3	-0.212140696	4.84392E-05	0.000130396
RRAGD	0.212115016	4.85438E-05	0.00013066
KIF18B	0.21211186	4.85567E-05	0.000130677
TCL1B	0.212096131	4.86209E-05	0.000130832
MDC1	0.212081653	4.868E-05	0.000130973
HMG20B	0.212078069	4.86947E-05	0.000130995
CXorf48	0.212017547	4.89428E-05	0.000131644

CCDC102B	-0.211987019	4.90683E-05	0.000131964
LZTS2	0.211966366	4.91535E-05	0.000132175
LOC728190	0.211945932	4.92379E-05	0.000132384
GTF2IRD2B	-0.211938138	4.92701E-05	0.000132453
LIME1	0.211928255	4.9311E-05	0.000132545
BTBD16	0.211881099	4.95065E-05	0.000133053
ARRB2	0.211815511	4.97796E-05	0.000133769
DDIT4	-0.211791656	4.98793E-05	0.000134019
C8orf55	0.211778046	4.99362E-05	0.000134153
GSTO2	0.211776476	4.99428E-05	0.000134153
ZNF248	-0.211761307	5.00064E-05	0.000134306
ZNF585B	-0.211750093	5.00534E-05	0.000134414
RSAD1	0.211714289	5.02039E-05	0.0001348
ACADSB	-0.211612533	5.06339E-05	0.000135936
WNT8B	-0.211603951	5.06703E-05	0.000136016
RGS10	0.211587416	5.07406E-05	0.000136186
SH3TC2	-0.211550952	5.08958E-05	0.000136584
MATR3	-0.211535124	5.09633E-05	0.000136747
ACCN2	0.211500393	5.11118E-05	0.000137127
THNSL1	-0.211486141	5.11729E-05	0.000137272
C17orf102	-0.211450522	5.13257E-05	0.000137664
S100A9	0.211447514	5.13386E-05	0.00013768
RNH1	0.211431229	5.14087E-05	0.000137849
ZNF548	-0.21139804	5.15517E-05	0.000138214
GRAMD4	-0.211352777	5.17474E-05	0.00013872
GLIS2	-0.211324064	5.18719E-05	0.000139035
GSTA3	0.211314824	5.1912E-05	0.000139124
FOXP4	0.211272078	5.2098E-05	0.000139603
CHST12	0.21126262	5.21392E-05	0.000139695
SSR1	0.211257895	5.21599E-05	0.000139713
FPGS	0.211257856	5.216E-05	0.000139713
PNPT1	-0.211181032	5.24963E-05	0.000140579
MAGED2	0.211180748	5.24975E-05	0.000140579
MFF	0.211101965	5.28444E-05	0.000141489
VDAC1	0.211094044	5.28794E-05	0.000141564
EFS	-0.211087782	5.29071E-05	0.000141619
PMP22	-0.211049669	5.30759E-05	0.000142052
TP53INP2	-0.211029674	5.31647E-05	0.00014227
EXOC4	0.211027067	5.31763E-05	0.000142278
SH3RF3	-0.211025779	5.3182E-05	0.000142278
IRS2	-0.211005745	5.32711E-05	0.000142498
NCKIPSD	0.210991217	5.33358E-05	0.000142651
AMY2B	-0.210951281	5.35141E-05	0.000143109
C19orf28	0.210879298	5.38367E-05	0.000143953
PLEKHA9	0.210876487	5.38494E-05	0.000143967
LOC100287	0.210815511	5.41242E-05	0.000144674

PLXNA2	-0.210814598	5.41284E-05	0.000144674
PHF19	0.210796876	5.42085E-05	0.000144869
DSCAM	-0.21069589	5.46673E-05	0.000146075
RTN4	-0.210649254	5.48804E-05	0.000146625
NANOS3	0.210598013	5.51154E-05	0.000147233
APOC2	0.210580168	5.51975E-05	0.000147433
ZBTB34	-0.210578246	5.52063E-05	0.000147437
LOC81691	0.210567664	5.52551E-05	0.000147547
PLCH2	-0.210474965	5.56837E-05	0.000148672
XRCC3	0.210433232	5.58777E-05	0.00014917
MCM6	0.210418221	5.59477E-05	0.000149336
ZNF486	-0.210416346	5.59564E-05	0.00014934
PLOD1	0.210400388	5.60308E-05	0.000149518
MMP21	-0.210384261	5.61062E-05	0.000149699
H6PD	-0.210352481	5.62549E-05	0.000150076
ACAT1	-0.210310872	5.64502E-05	0.000150577
C17orf50	0.210290802	5.65446E-05	0.000150808
LOC441204	-0.210276175	5.66135E-05	0.000150972
KIAA1244	-0.210274281	5.66225E-05	0.000150976
HIGD1B	0.210250924	5.67327E-05	0.000151249
ADAL	-0.210223211	5.68637E-05	0.000151567
PCCA	-0.210222244	5.68683E-05	0.000151567
CCDC154	0.210220874	5.68748E-05	0.000151567
VAT1	0.210197598	5.69851E-05	0.000151841
ARL13A	-0.210152619	5.71988E-05	0.00015239
LCP2	-0.210132696	5.72937E-05	0.00015261
FOXK2	0.210132087	5.72966E-05	0.00015261
SLC39A1	0.210126802	5.73218E-05	0.000152656
PPP1R3G	0.210121068	5.73492E-05	0.000152709
SYP	0.210111748	5.73937E-05	0.000152807
LOC441046	-0.210105241	5.74248E-05	0.000152869
SBF1	-0.210083789	5.75273E-05	0.000153109
LRRC49	-0.210083214	5.75301E-05	0.000153109
RTKN	0.210047514	5.77012E-05	0.000153543
CNOT1	-0.210026646	5.78014E-05	0.00015379
L3MBTL4	-0.210003492	5.79128E-05	0.000154066
MAPK14	0.2098706	5.85562E-05	0.000155756
MS4A7	-0.209798866	5.89063E-05	0.000156666
ATP8B2	-0.209714896	5.93186E-05	0.000157742
VRK2	-0.209676724	5.95069E-05	0.000158222
STARD4	-0.20962868	5.97447E-05	0.000158833
GNLY	0.20958799	5.99469E-05	0.000159349
BMP8B	-0.209546696	6.01527E-05	0.000159875
KIAA0467	-0.209537607	6.0198E-05	0.000159974
DNAJC25	-0.209519555	6.02883E-05	0.000160192
SHISA2	-0.209505944	6.03564E-05	0.000160352

TMEM132E	-0.209500744	6.03824E-05	0.0001604
SHKBP1	0.209487113	6.04508E-05	0.00016056
DTX1	-0.209470661	6.05333E-05	0.000160758
CWC22	-0.209446908	6.06527E-05	0.000161053
THEMIS	-0.209434485	6.07152E-05	0.000161198
C7orf40	0.209384544	6.09672E-05	0.000161845
JPH2	-0.20935628	6.11102E-05	0.000162203
MTO1	-0.209313917	6.13252E-05	0.000162752
COQ4	0.209249766	6.16521E-05	0.000163598
DOCK2	-0.209238722	6.17086E-05	0.000163726
PLEKHH2	-0.209221979	6.17942E-05	0.000163931
ADAT3	0.209189881	6.19588E-05	0.000164346
NCRNA0010	0.209155632	6.21348E-05	0.000164791
DKFZp686A	0.209151475	6.21562E-05	0.000164826
STARD5	-0.209088957	6.24788E-05	0.000165659
SEMA6C	0.209079541	6.25276E-05	0.000165766
GPX3	0.209026469	6.28029E-05	0.000166474
PTPRA	-0.208987653	6.3005E-05	0.000166988
C2orf55	-0.208958828	6.31555E-05	0.000167364
C1orf168	-0.20892911	6.33109E-05	0.000167754
RAB11FIP1	-0.208879332	6.35722E-05	0.000168424
OVGP1	0.208861071	6.36683E-05	0.000168656
MICA	0.208837979	6.379E-05	0.000168956
FGD1	-0.208820755	6.38809E-05	0.000169174
C3orf59	-0.208788328	6.40525E-05	0.000169601
DNAJC30	0.208787035	6.40593E-05	0.000169601
UCN2	0.208771999	6.4139E-05	0.00016979
SPR	0.208731128	6.43561E-05	0.000170342
ALOX5	-0.208702065	6.45108E-05	0.000170729
CCND2	-0.208671704	6.46729E-05	0.000171135
SPHAR	0.20865739	6.47495E-05	0.000171315
HUS1B	0.208641299	6.48356E-05	0.00017152
CCBL1	0.208634723	6.48708E-05	0.00017159
GPR45	-0.208610161	6.50026E-05	0.000171916
WBSCR27	0.208585726	6.51339E-05	0.00017224
SIGLEC6	-0.208513085	6.55259E-05	0.000173254
HHIPL2	0.208493711	6.56308E-05	0.000173508
MCF2	-0.208489896	6.56515E-05	0.00017354
GAGE12D	0.20847459	6.57345E-05	0.000173736
ASAP1	-0.208472341	6.57467E-05	0.000173745
TLR2	-0.208430958	6.59717E-05	0.000174317
GPATCH1	0.208409555	6.60883E-05	0.000174602
NT5C3	0.208402482	6.61269E-05	0.000174681
KCNA5	-0.208364924	6.63322E-05	0.0001752
MMP28	-0.208362678	6.63445E-05	0.000175209
RACGAP1P	0.208356764	6.63769E-05	0.000175271

LOC652276	-0.208315765	6.66018E-05	0.000175842
ASXL3	-0.208312465	6.66199E-05	0.000175866
SNAP47	0.208290954	6.67382E-05	0.000176155
SLC28A1	-0.208267157	6.68694E-05	0.000176478
NR2F1	-0.208249408	6.69673E-05	0.000176713
PRR11	-0.208218689	6.71372E-05	0.000177138
GTPBP5	0.20819941	6.7244E-05	0.000177396
ARHGEF3	-0.208196738	6.72588E-05	0.000177412
NCR1	-0.208183492	6.73323E-05	0.000177582
HNRNPAB	0.208116198	6.77068E-05	0.000178546
NUDT12	-0.208074439	6.79402E-05	0.000179138
CDIPT	0.208069161	6.79698E-05	0.000179192
BEND7	-0.2079993	6.83621E-05	0.000180203
NPIP	-0.207961137	6.85774E-05	0.000180746
C3orf33	0.207946566	6.86597E-05	0.000180939
PBK	0.207917432	6.88246E-05	0.00018135
MARCKS	0.207886338	6.9001E-05	0.000181791
KDM2B	-0.207874617	6.90677E-05	0.000181942
LST1	0.207863963	6.91283E-05	0.000182078
B3GNT5	-0.207820046	6.93786E-05	0.000182713
LY6H	0.207781833	6.95971E-05	0.000183264
EYS	-0.207706565	7.00294E-05	0.000184378
TAX1BP1	-0.207633255	7.04529E-05	0.000185469
GUCY2D	0.20760417	7.06216E-05	0.000185888
HULC	0.207587297	7.07197E-05	0.000186122
SAR1B	-0.207483452	7.13259E-05	0.000187692
FGL2	-0.207393035	7.18576E-05	0.000189067
SCFD1	-0.207387217	7.1892E-05	0.000189132
C1orf203	0.207367988	7.20056E-05	0.000189406
MCCC1	-0.207342655	7.21556E-05	0.000189775
H2AFY	0.207270499	7.25843E-05	0.000190878
MLYCD	-0.207252083	7.26941E-05	0.000191141
TNIP2	0.207200667	7.30015E-05	0.000191924
ZDHHC8	-0.207196087	7.30289E-05	0.000191971
SNCG	0.207156389	7.32672E-05	0.000192572
HIST1H2AJ	0.207144082	7.33412E-05	0.000192741
ID2	-0.207135684	7.33918E-05	0.000192848
ZNF658	-0.207089986	7.36674E-05	0.000193547
KIF15	0.207053603	7.38875E-05	0.0001941
PARG	-0.20705145	7.39006E-05	0.000194108
LOC338758	-0.207030702	7.40264E-05	0.000194413
FAM49A	-0.207027725	7.40445E-05	0.000194435
HSD17B8	0.20693846	7.45883E-05	0.000195837
JUNB	-0.20690402	7.47991E-05	0.000196365
PEG3AS	-0.206898686	7.48318E-05	0.000196425
FHIT	0.206891268	7.48773E-05	0.000196518

RIOK3	-0.206884004	7.49219E-05	0.000196609
TRAM2	-0.206875081	7.49767E-05	0.000196716
SLC2A3	-0.206874173	7.49822E-05	0.000196716
RAB31	-0.20686687	7.50271E-05	0.000196808
ARHGAP17	-0.206848648	7.51392E-05	0.000197076
POU5F1	0.206837445	7.52082E-05	0.000197231
LOC147804	0.206831216	7.52466E-05	0.000197306
TTF2	-0.206788374	7.55111E-05	0.000197973
PRDM9	0.206780917	7.55572E-05	0.000198068
CT45A6	0.206721664	7.59247E-05	0.000198972
TMPPE	-0.206721457	7.5926E-05	0.000198972
TRAPPC9	0.206720483	7.59321E-05	0.000198972
WDR3	-0.206701523	7.605E-05	0.000199255
TRPM5	0.206653149	7.63517E-05	0.000200019
NAA11	0.206632172	7.64829E-05	0.000200336
C1orf113	0.206569787	7.68744E-05	0.000201335
FAM175B	-0.206546522	7.70208E-05	0.000201692
NR1H2	0.206515453	7.72168E-05	0.000202179
UBAC1	0.206504612	7.72853E-05	0.000202332
GPR34	-0.206481704	7.74302E-05	0.000202684
KCNQ4	-0.206471469	7.74951E-05	0.000202827
PRKAB1	0.206436122	7.77194E-05	0.000203378
ROR2	-0.20643508	7.7726E-05	0.000203378
DHX58	-0.206384266	7.80495E-05	0.000204198
TCTEX1D2	0.206373183	7.81203E-05	0.000204356
HIST2H2AA3	0.206320509	7.84573E-05	0.000205211
LEPREL1	-0.206313527	7.85021E-05	0.000205282
CALU	-0.206313064	7.8505E-05	0.000205282
LRRCC1	-0.206301704	7.85779E-05	0.000205446
YIPF5	-0.206262742	7.88285E-05	0.000206074
LOX	-0.206260447	7.88433E-05	0.000206085
ASCL2	0.206252079	7.88972E-05	0.000206199
SEPP1	-0.206226715	7.90608E-05	0.000206594
MARK1	-0.206225457	7.90689E-05	0.000206594
ASRGL1	0.20621609	7.91294E-05	0.000206725
ZNF280A	0.206210344	7.91666E-05	0.000206795
PRDM13	0.2061659	7.94544E-05	0.000207519
KCNK9	0.206145094	7.95895E-05	0.000207845
PRKAR2B	-0.206124469	7.97237E-05	0.000208168
MAFK	-0.206003415	8.05152E-05	0.000210207
PRR5	0.205963609	8.07771E-05	0.000210863
MLF2	0.205885774	8.12916E-05	0.000212178
RASA3	-0.205783365	8.19731E-05	0.000213929
RBM20	-0.205741498	8.22533E-05	0.000214632
XRCC4	0.205738175	8.22756E-05	0.000214657
DR1	-0.205736898	8.22841E-05	0.000214657

C2orf64	0.205714072	8.24373E-05	0.000215028
NBPF7	-0.20570359	8.25077E-05	0.000215184
SHQ1	-0.205697737	8.25471E-05	0.000215258
LOC84989	0.205649857	8.28697E-05	0.000216071
NLN	-0.20564306	8.29156E-05	0.000216162
SFTA1P	0.20559651	8.32305E-05	0.000216955
PPP1R14D	0.205573434	8.33871E-05	0.000217335
KCNIP1	-0.205528579	8.36921E-05	0.000218101
DDX18	-0.205519096	8.37568E-05	0.000218241
NR4A1	-0.205513719	8.37934E-05	0.000218308
SLC24A4	-0.205414508	8.44727E-05	0.000220049
RGS11	-0.205363112	8.48267E-05	0.000220942
TMEM180	0.205347141	8.49369E-05	0.0002212
PSMD2	0.205344513	8.49551E-05	0.000221219
FREM2	-0.205334703	8.50229E-05	0.000221366
WIZ	-0.205316482	8.5149E-05	0.000221666
DUT	0.205304426	8.52325E-05	0.000221854
SAFB	0.205282091	8.53874E-05	0.000222228
B4GALNT3	-0.205254884	8.55765E-05	0.000222691
NUP153	-0.205213716	8.58634E-05	0.000223409
HBS1L	-0.20519346	8.60049E-05	0.000223747
ARFGEF1	-0.205190337	8.60267E-05	0.000223775
MPPE1	-0.205116635	8.65435E-05	0.00022509
ITPKA	0.20509858	8.66705E-05	0.000225391
TRIM9	-0.205089955	8.67313E-05	0.000225519
AK3L1	0.205067757	8.68878E-05	0.000225897
GSTM5	-0.205065666	8.69025E-05	0.000225906
CAMTA1	0.205047837	8.70285E-05	0.000226204
LAMB3	-0.20499726	8.73867E-05	0.000227105
DSTYK	-0.204975165	8.75436E-05	0.000227483
VPS45	0.204923711	8.791E-05	0.000228405
CUL2	-0.204789048	8.88759E-05	0.000230885
GGT5	-0.204772941	8.89921E-05	0.000231157
GMEB2	0.204756562	8.91104E-05	0.000231434
TBCCD1	-0.204653939	8.9855E-05	0.000233337
RAF1	-0.204645302	8.99179E-05	0.00023347
KIF2A	-0.204639903	8.99573E-05	0.000233542
DPP9	-0.204558011	9.05563E-05	0.000235066
C4orf49	-0.204554339	9.05832E-05	0.000235106
NBEAL2	-0.204550432	9.06119E-05	0.000235149
LOC148189	-0.204497359	9.10024E-05	0.000236132
TRIM7	0.204488526	9.10675E-05	0.00023627
PKD1L1	-0.204460877	9.12717E-05	0.000236769
PID1	-0.204456988	9.13004E-05	0.000236813
ARPC2	0.204387978	9.1812E-05	0.000238106
LOC285954	-0.204386502	9.1823E-05	0.000238106

FKRP	-0.20438106	9.18635E-05	0.00023818
DHX9	-0.204367479	9.19645E-05	0.000238411
EPB41L3	-0.204349415	9.20991E-05	0.000238729
NCAM1	-0.20433037	9.22412E-05	0.000239066
IL17REL	-0.204321144	9.23101E-05	0.000239213
ENKUR	-0.204256645	9.27932E-05	0.000240434
XPR1	-0.2041911	9.32865E-05	0.000241681
RSPH4A	-0.204128156	9.37626E-05	0.000242883
KIAA0895L	-0.204026464	9.45366E-05	0.000244856
DHX35	-0.204018843	9.45948E-05	0.000244975
GMFB	-0.203992195	9.47987E-05	0.000245471
IL4I1	0.203942115	9.51831E-05	0.000246434
C22orf26	0.203925201	9.53133E-05	0.000246739
LEPR	-0.203876191	9.56913E-05	0.000247686
FLT3	-0.203865054	9.57774E-05	0.000247876
RAB39	-0.203841452	9.59601E-05	0.000248317
SLC44A2	-0.20383075	9.60431E-05	0.000248499
LRRC8B	-0.203822527	9.61069E-05	0.000248632
SSRP1	0.203788659	9.637E-05	0.00024928
UBP1	-0.203774921	9.6477E-05	0.000249525
KCNK5	-0.20376242	9.65744E-05	0.000249744
MMP16	-0.203746824	9.6696E-05	0.000250026
FAM116A	-0.20373268	9.68065E-05	0.000250279
CPNE3	-0.203662045	9.73598E-05	0.000251677
CARD6	-0.203634411	9.75771E-05	0.000252206
PHLDA2	0.203631909	9.75968E-05	0.000252224
C16orf86	0.203597878	9.78651E-05	0.000252885
GGA1	0.203538318	9.83362E-05	0.000254069
SIK1	-0.203534757	9.83645E-05	0.000254109
SYCE1L	0.203519839	9.84829E-05	0.000254382
HIST1H2AE	0.203516291	9.8511E-05	0.000254422
PGPEP1	0.203475483	9.88356E-05	0.000255227
PRTG	-0.203460034	9.89588E-05	0.000255481
RGS20	0.203458478	9.89712E-05	0.000255481
MBTD1	-0.203458312	9.89725E-05	0.000255481
EPR1	0.203433939	9.91671E-05	0.00025595
MAPK3	0.203431696	9.91851E-05	0.000255964
SUMO4	-0.203358389	9.97726E-05	0.000257447
UPK3BL	0.203272745	0.000100463	0.000259195
FAM27A	0.203268538	0.000100497	0.000259249
BLVRB	0.20322047	0.000100887	0.000260221
BAG1	0.203207897	0.000100989	0.000260451
ZNF534	0.203204365	0.000101018	0.000260491
G6PC	-0.203191439	0.000101123	0.000260728
STK38	0.203179868	0.000101217	0.000260938
KIF3C	-0.203103756	0.000101839	0.000262507

LOC255167	-0.203081129	0.000102025	0.000262951
GALT	0.203072122	0.000102099	0.000263108
INSIG2	-0.203060882	0.000102191	0.000263312
VASP	0.203053207	0.000102254	0.00026344
COG8	-0.203048304	0.000102294	0.00026351
TMEM20	-0.203035312	0.000102401	0.000263751
XPO1	-0.202989328	0.000102781	0.000264694
TNFRSF10D	-0.202920944	0.000103348	0.000266119
GM2A	0.202908564	0.00010345	0.00026635
MYOZ1	-0.202865461	0.000103809	0.000267213
MGC16703	-0.202865073	0.000103813	0.000267213
HDAC6	-0.202859592	0.000103858	0.000267297
SLC25A20	-0.202836875	0.000104048	0.00026775
C21orf59	0.202823697	0.000104158	0.000267999
RHBDD3	0.20281271	0.00010425	0.000268202
SERTAD3	0.202804153	0.000104322	0.000268351
RPL23AP32	0.202796529	0.000104386	0.000268481
GAPT	-0.202780037	0.000104524	0.000268802
LYZL1	0.202748914	0.000104786	0.00026944
ZSWIM4	-0.202726802	0.000104972	0.000269884
STEAP1	0.202705689	0.000105151	0.000270308
PPAPDC1A	-0.202693239	0.000105256	0.000270543
CLEC2D	0.202681628	0.000105354	0.00027076
FAM82B	0.202661897	0.000105521	0.000271155
LOC642846	0.202658435	0.00010555	0.000271195
WDR92	-0.202644724	0.000105667	0.000271459
MTPAP	-0.202630436	0.000105788	0.000271735
LOC84931	0.202593212	0.000106104	0.000272513
NUDT22	0.20253235	0.000106624	0.000273812
ZDHHC18	0.202523985	0.000106695	0.000273961
SLCO1C1	-0.202519741	0.000106732	0.000274019
PHACTR3	-0.20251167	0.000106801	0.000274161
POMGNT1	0.202508677	0.000106827	0.000274191
TOP1P2	0.202499435	0.000106906	0.000274359
PDIA3	0.202476602	0.000107102	0.000274827
GPC6	-0.202447471	0.000107352	0.000275435
RASL10B	0.202424801	0.000107548	0.0002759
RABL2B	-0.202394729	0.000107807	0.000276531
BAP1	0.202390651	0.000107843	0.000276586
FADD	0.202367912	0.000108039	0.000277055
DENND2A	-0.202351766	0.000108179	0.000277378
FST	0.202334895	0.000108326	0.000277717
CORT	0.202267884	0.000108909	0.000279177
PCDHA13	-0.202256934	0.000109005	0.000279386
RRN3P3	-0.202241038	0.000109144	0.000279706
GPR44	0.202224108	0.000109292	0.000280038

PARS2	0.202223019	0.000109301	0.000280038
LOC400696	0.202181192	0.000109668	0.000280942
STXBP2	0.202166102	0.000109801	0.000281246
FAF2	-0.202146708	0.000109972	0.000281647
CCR1	-0.20208965	0.000110475	0.0002829
AIF1L	-0.202082608	0.000110538	0.000283023
GDF15	0.202074757	0.000110607	0.000283165
RABGGTA	0.202056956	0.000110765	0.000283532
AK1	0.202003286	0.000111242	0.000284716
CCL15	0.201982492	0.000111427	0.000285154
CYP27C1	-0.201952617	0.000111694	0.000285765
MYADML	0.201952527	0.000111694	0.000285765
USF2	0.201902493	0.000112142	0.000286874
GLYATL1	-0.201863878	0.000112489	0.000287725
CTAG2	0.201823417	0.000112854	0.00028862
GOLGA8DP	0.20172733	0.000113724	0.000290809
COQ6	-0.201701767	0.000113957	0.000291366
FUS	0.201675564	0.000114196	0.000291939
PLEKHA2	-0.201649775	0.000114431	0.000292504
NQO1	0.201639424	0.000114526	0.000292708
H2AFY2	0.201621493	0.00011469	0.00029309
CHST15	-0.2016149	0.00011475	0.000293207
FPR2	-0.201566252	0.000115197	0.000294311
OTOA	0.201529064	0.00011554	0.000295148
CEP152	-0.201460393	0.000116175	0.000296733
ANKRD29	-0.201443775	0.000116329	0.000297088
AGXT2L1	-0.201438829	0.000116375	0.00029714
SLC41A1	-0.201438405	0.000116379	0.00029714
PPA1	0.20135685	0.000117139	0.000299041
LGI2	-0.201348305	0.000117219	0.000299207
RCAN3	-0.201343941	0.00011726	0.000299273
BAMBI	0.201328287	0.000117406	0.000299608
AHNAK2	-0.201307938	0.000117597	0.000300057
FGFBP3	0.201305838	0.000117617	0.000300068
EXTL2	-0.201243654	0.000118201	0.000301522
CEP57	-0.201242016	0.000118217	0.000301522
VTI1B	0.201236674	0.000118267	0.000301612
UBE2MP1	0.201217725	0.000118446	0.000301991
ARL2BP	-0.201217712	0.000118446	0.000301991
CLEC14A	-0.201189762	0.00011871	0.000302626
MEX3D	-0.20113533	0.000119226	0.000303903
BMS1	0.201133229	0.000119246	0.000303915
CDKN2A	0.201121382	0.000119359	0.000304163
DLX1	0.201101325	0.00011955	0.000304611
PHB2	0.201098744	0.000119575	0.000304634
P4HB	0.201082971	0.000119725	0.000304979

TMEM176A	0.201076179	0.00011979	0.000305105
F11	-0.201041851	0.000120118	0.000305901
LOC148709	0.200983582	0.000120677	0.000307285
NOL4	-0.200952544	0.000120975	0.000308006
PDPN	-0.200918405	0.000121304	0.000308805
ICMT	-0.200909293	0.000121393	0.000308989
PAGE1	0.200900438	0.000121478	0.000309168
SULT1C2	0.200898765	0.000121494	0.000309169
NIPAL3	-0.200840256	0.000122061	0.000310573
HUNK	-0.200776527	0.000122682	0.000312112
DNAH1	-0.20076821	0.000122763	0.000312278
LPHN3	-0.200718504	0.000123249	0.000313476
FAM150B	-0.200699216	0.000123439	0.000313917
TPP1	-0.200667274	0.000123753	0.000314675
ZNF700	-0.200645985	0.000123962	0.000315168
RIBC2	0.200604779	0.000124369	0.000316156
CDH9	0.200603386	0.000124383	0.000316156
CATSPERG	-0.200532311	0.000125087	0.000317907
FUCA2	0.200432209	0.000126086	0.000320404
CXorf66	-0.200418589	0.000126223	0.00032071
STYX	-0.200380143	0.000126609	0.000321649
GHRL	0.200368917	0.000126721	0.000321895
CXADRP3	-0.200322177	0.000127193	0.000323051
KRTAP6-3	0.20030155	0.000127401	0.000323539
BNIP3	0.200297411	0.000127443	0.000323604
RHOQ	-0.200292466	0.000127493	0.00032369
SLC10A1	-0.200269496	0.000127726	0.000324239
TFIP11	0.200244615	0.000127978	0.000324838
DOLK	0.200208249	0.000128348	0.000325735
MGST3	0.200173777	0.000128699	0.000326586
FAM149B1	-0.200113956	0.000129311	0.000328097
TSSK3	-0.200095909	0.000129497	0.000328525
HIST1H4E	0.200034888	0.000130125	0.000330076
DOK6	-0.199988036	0.000130609	0.000331262
LOC344595	-0.199951041	0.000130992	0.000332193
BCAS1	0.199933257	0.000131177	0.000332619
LOC100130	-0.199895327	0.000131572	0.000333577
LRRN3	-0.199780686	0.000132772	0.000336577
FGF14	-0.19975331	0.00013306	0.000337264
TDO2	-0.199746948	0.000133127	0.000337391
GTF2F1	0.199729848	0.000133307	0.000337805
SUMO3	0.19972573	0.00013335	0.000337872
CDK5R2	0.199655319	0.000134096	0.000339717
LUC7L	0.199646434	0.00013419	0.000339912
MGAT3	-0.19962026	0.000134468	0.000340574
KBTBD2	-0.199531784	0.000135412	0.000342922

C19orf44	0.199526647	0.000135467	0.000343017
CKMT2	-0.199477534	0.000135994	0.000344308
PGM2L1	-0.199422696	0.000136585	0.00034576
PRPF38A	0.199372122	0.000137132	0.000347101
VCX3B	0.199303304	0.00013788	0.000348948
PIGO	-0.199278978	0.000138145	0.000349575
PRPF8	-0.199256694	0.000138388	0.000350146
ZNF100	-0.199218954	0.000138801	0.000351147
PHYHIPL	0.199192549	0.000139091	0.000351835
GREM1	-0.199143804	0.000139627	0.000353146
RS1	0.199139602	0.000139673	0.000353219
ZNF517	0.199089293	0.000140229	0.000354579
CTSS	-0.199073596	0.000140403	0.000354973
C20orf197	-0.199060583	0.000140547	0.000355293
ABCA5	-0.198952307	0.000141752	0.000358294
POSTN	-0.198896349	0.000142379	0.000359833
COL5A2	-0.198889035	0.000142461	0.000359995
PSMD14	0.198882922	0.00014253	0.000360123
DYRK2	-0.198847494	0.000142929	0.000361084
CCND1	-0.198826486	0.000143165	0.000361636
PDZD4	-0.198813344	0.000143314	0.000361931
AKTIP	-0.198812344	0.000143325	0.000361931
FRMD8	-0.19881131	0.000143337	0.000361931
LLGL1	-0.198788223	0.000143598	0.000362544
SART3	-0.198759949	0.000143918	0.000363307
C21orf2	0.198744805	0.00014409	0.000363694
ADH1B	-0.198740964	0.000144133	0.000363758
HK1	-0.198738143	0.000144165	0.000363793
CD200R1	-0.198654754	0.000145115	0.000366092
ZNF618	-0.198653712	0.000145127	0.000366092
EEPD1	-0.198653329	0.000145132	0.000366092
EPOR	-0.198602294	0.000145716	0.000367519
NRIP2	-0.198584258	0.000145923	0.000367995
ARSK	-0.198581706	0.000145952	0.000368022
DNAL4	0.198579991	0.000145972	0.000368025
PAOX	0.198565449	0.000146139	0.0003684
EPS8L3	0.198551337	0.000146301	0.000368763
ZMYND10	0.198540171	0.00014643	0.00036904
STARD6	0.198519926	0.000146663	0.000369582
ILVBL	0.198420362	0.000147817	0.000372431
TMEM207	0.198419063	0.000147832	0.000372431
ISG20L2	-0.198370413	0.000148398	0.000373812
GPR180	-0.198291347	0.000149324	0.000376095
DENND3	-0.198280782	0.000149448	0.00037636
RPRD2	-0.198207305	0.000150313	0.000378491
ARSD	-0.198187298	0.00015055	0.000379038

MKNK2	-0.19815221	0.000150965	0.000380037
C2orf60	-0.19814054	0.000151104	0.000380337
LYPLAL1	0.19811095	0.000151455	0.00038117
MTSS1	-0.198109479	0.000151473	0.00038117
C15orf21	0.198100617	0.000151578	0.000381387
CYP2A13	-0.198091392	0.000151688	0.000381615
MMADHC	-0.198076336	0.000151867	0.000382018
MIF4GD	0.198023679	0.000152496	0.000383552
ARHGAP18	-0.198016718	0.00015258	0.000383713
ZFP90	-0.198001498	0.000152762	0.000384123
RARA	-0.197955017	0.00015332	0.000385478
NCAPH	0.197947824	0.000153407	0.000385647
IL17RD	-0.197943782	0.000153456	0.000385721
FCRL6	-0.197933668	0.000153577	0.000385978
NOC3L	-0.197891781	0.000154083	0.0003872
LAG3	0.197880555	0.000154219	0.000387492
SFRS17A	-0.197878227	0.000154247	0.000387514
C2orf44	0.197826423	0.000154875	0.000389043
CCDC7	-0.197805128	0.000155134	0.000389644
CCDC17	0.19776652	0.000155604	0.000390776
RPS6KA3	-0.197756205	0.00015573	0.000391042
EHD4	-0.197671775	0.000156764	0.000393589
ATP6AP2	-0.197666512	0.000156828	0.000393702
GABRR3	0.19763917	0.000157165	0.000394496
PLA2G12B	0.197636718	0.000157195	0.000394522
ACTL8	0.197557545	0.000158173	0.000396926
TSC22D1	-0.1975372	0.000158425	0.000397509
LGALS8	-0.197530514	0.000158508	0.000397667
GDF1	0.197509448	0.00015877	0.000398274
KLF2	-0.197492186	0.000158984	0.000398762
TFAP2E	0.197454045	0.00015946	0.000399904
ANKRD13A	-0.197442833	0.0001596	0.000400205
STC1	-0.197416557	0.000159928	0.000400978
C9orf86	0.197404168	0.000160083	0.000401317
SYT7	-0.197369166	0.000160522	0.000402367
CLEC18C	0.197359598	0.000160643	0.000402617
LHB	0.197348788	0.000160778	0.000402907
HYDIN	-0.197336454	0.000160934	0.000403196
RBBP8	-0.197336412	0.000160934	0.000403196
ACPT	0.197259464	0.000161906	0.000405579
IL4R	-0.197225805	0.000162332	0.000406597
SUZ12	-0.197206281	0.00016258	0.000407167
KIAA0528	-0.197150748	0.000163288	0.000408886
C1orf173	-0.197146349	0.000163344	0.000408976
CBWD1	0.197120211	0.000163678	0.00040976
PARP15	-0.196987537	0.000165383	0.000413977

PNRC1	-0.196975499	0.000165539	0.000414315
SERAC1	-0.196862414	0.000167007	0.000417936
CFTR	-0.196826696	0.000167473	0.00041905
CCL3L1	0.19681881	0.000167576	0.000419255
SPAG5	0.19681616	0.00016761	0.000419289
CHRNB4	-0.19681419	0.000167636	0.000419301
ZCCHC16	-0.196783733	0.000168035	0.000420246
CTTNBP2	-0.196771664	0.000168193	0.000420589
CNKSR2	-0.196768626	0.000168233	0.000420636
CSPG4	-0.196765746	0.000168271	0.000420677
CENPT	0.196760481	0.00016834	0.000420797
LRRC7	-0.196752545	0.000168444	0.000421005
SRD5A2	-0.19674377	0.00016856	0.000421241
LRRC19	-0.196701676	0.000169114	0.000422573
USP7	-0.196697228	0.000169173	0.000422666
CLIC3	0.196667386	0.000169567	0.000423598
ZFP112	-0.196661225	0.000169648	0.000423748
WFDC10A	0.196623688	0.000170145	0.000424937
ADCY7	-0.196590641	0.000170584	0.000425979
SPATA9	-0.196564839	0.000170928	0.000426783
RPRML	0.196536243	0.000171309	0.000427682
C5orf44	-0.196530898	0.00017138	0.000427806
C10orf41	0.196510979	0.000171647	0.000428417
SLC6A17	-0.196478564	0.000172081	0.000429447
SND1	0.196452181	0.000172435	0.000430276
ROPN1	-0.196393874	0.000173219	0.00043218
KRTDAP	0.196386577	0.000173318	0.000432318
ZNF385B	-0.196386558	0.000173318	0.000432318
AGFG1	-0.196383352	0.000173361	0.000432372
PDIA5	-0.196344338	0.000173889	0.000433633
C3orf54	0.196336285	0.000173998	0.00043385
LOXL2	-0.196331383	0.000174064	0.000433962
FAM86C	0.196317709	0.000174249	0.000434369
NXN	-0.196306036	0.000174408	0.00043471
ERAP1	-0.196303616	0.000174441	0.000434737
MTERF	-0.196277019	0.000174802	0.000435584
TTLL5	-0.196260097	0.000175032	0.000436103
DNASE1L2	0.196229295	0.000175452	0.000437095
WDR61	0.196223274	0.000175535	0.000437194
C1orf66	0.196223174	0.000175536	0.000437194
LOC162632	-0.196202698	0.000175816	0.000437836
LYPD2	-0.196190993	0.000175976	0.00043818
ZNF429	-0.196162726	0.000176363	0.00043909
MSI1	0.196107032	0.000177129	0.00044094
C3orf19	0.196101095	0.000177211	0.000441089
ATP10A	-0.196084542	0.000177439	0.000441602

FKBP9	-0.196043045	0.000178012	0.000442973
PODNL1	-0.196024271	0.000178272	0.000443562
TEX9	-0.196022732	0.000178294	0.000443562
EPX	-0.196017779	0.000178362	0.000443678
ALOX12P2	-0.195978014	0.000178914	0.000444995
TNFAIP8L2	0.195975118	0.000178954	0.00044504
SLC5A1	-0.195938333	0.000179467	0.000446258
ADCY1	-0.195914757	0.000179796	0.00044702
APH1A	0.195881211	0.000180265	0.000448095
GBP3	-0.195880614	0.000180273	0.000448095
TMED5	-0.195804886	0.000181336	0.000450682
OCLN	-0.195791334	0.000181527	0.0004511
FYB	-0.195762308	0.000181937	0.000452061
NLGN4X	-0.195687759	0.000182992	0.000454628
EHD2	-0.195642565	0.000183635	0.000456168
LRRC28	0.195631953	0.000183786	0.000456484
ISM1	-0.195630424	0.000183808	0.000456484
CAPG	0.19561995	0.000183958	0.000456798
TMEM145	0.195577277	0.000184568	0.000458255
ZNF570	-0.19554897	0.000184973	0.000459205
FAM55B	-0.195544533	0.000185037	0.000459305
ADAMTS8	-0.19552102	0.000185374	0.000460019
CXADR	-0.195520409	0.000185383	0.000460019
SMPX	0.195519692	0.000185393	0.000460019
FLJ25363	0.195517667	0.000185423	0.000460034
TNPO2	-0.195514872	0.000185463	0.000460076
RPE	-0.195476672	0.000186013	0.000461383
INMT	-0.195443217	0.000186496	0.000462523
PPP1R2P1	-0.195400657	0.000187112	0.000463994
PXMP4	0.195379042	0.000187425	0.000464702
SECISBP2	-0.195377742	0.000187444	0.000464702
MYST2	-0.195363263	0.000187655	0.000465166
GBP1	-0.195296043	0.000188634	0.000467536
C9orf82	-0.195259045	0.000189176	0.000468819
TMTC4	-0.195253427	0.000189258	0.000468965
CMYA5	-0.195224646	0.00018968	0.000469952
ZNF70	-0.19521439	0.000189831	0.000470267
DUSP10	-0.195209079	0.000189909	0.000470402
FEV	0.195197879	0.000190073	0.000470752
GREM2	-0.195191587	0.000190166	0.000470922
P2RX2	-0.195182586	0.000190299	0.000471141
RNF170	-0.195182365	0.000190302	0.000471141
RARRES2	0.195159797	0.000190635	0.000471906
CNKSR3	-0.195113079	0.000191325	0.000473557
CENPBD1	0.19508561	0.000191732	0.000474505
CD55	-0.195071734	0.000191938	0.000474956

IMPA2	0.19506796	0.000191994	0.000475035
PPT1	0.195042186	0.000192377	0.000475924
CLEC7A	-0.195035344	0.000192479	0.000476117
KCTD7	-0.195024241	0.000192644	0.000476467
NCRNA0017	0.19498129	0.000193285	0.000477993
FAM3B	0.194967446	0.000193492	0.000478445
PHLDB1	-0.194906706	0.000194403	0.000480637
NCRNA0009	-0.194824849	0.000195637	0.000483628
MEP1B	-0.19475241	0.000196734	0.000486281
SPDYE5	-0.19474608	0.000196831	0.000486458
TPM2	0.194692393	0.000197648	0.000488419
C1orf128	0.194668003	0.000198021	0.000489279
FAM91A1	-0.194649951	0.000198297	0.0004899
DNAJC10	-0.194589806	0.00019922	0.000492119
PMS2L4	0.194581162	0.000199353	0.000492386
TMCO6	0.194565639	0.000199592	0.000492915
KCNJ16	-0.194515426	0.000200366	0.000494767
PTPRN	0.194493437	0.000200707	0.000495546
GPHN	-0.194474456	0.000201001	0.00049621
USP44	-0.194439812	0.000201538	0.000497476
SNX5	0.194434278	0.000201624	0.000497627
MAGED1	0.194366045	0.000202688	0.000500189
PLP2	0.194266488	0.000204249	0.00050398
SFPQ	0.194254884	0.000204432	0.000504368
CFL2	-0.194234761	0.000204749	0.000505088
ENOX2	-0.194228598	0.000204846	0.000505265
PRKD2	0.194221174	0.000204963	0.000505491
MMP1	0.194208456	0.000205164	0.000505924
CANT1	0.194194422	0.000205386	0.000506409
CNTLN	-0.194189732	0.00020546	0.000506529
RERE	-0.194172404	0.000205735	0.000507143
RAPGEF3	-0.194169975	0.000205773	0.000507175
CRELD1	0.194085892	0.00020711	0.000510406
ALX1	0.194046095	0.000207745	0.000511908
FAM3C	-0.194037672	0.00020788	0.000512177
NMUR1	-0.193994017	0.000208579	0.000513837
STAP2	0.193915336	0.000209846	0.000516892
CATSPER3	0.193907765	0.000209968	0.00051713
C18orf19	-0.193902593	0.000210051	0.000517271
CAPS	0.193880801	0.000210404	0.000518075
UBE2R2	0.19386956	0.000210586	0.000518459
C20orf96	0.193859683	0.000210746	0.00051875
CPAMD8	-0.193859044	0.000210756	0.00051875
DKFZP434K	-0.193818855	0.000211408	0.000520291
TIMP2	-0.193767106	0.000212251	0.0005223
BBS9	-0.193724918	0.00021294	0.000523932

CEBPD	0.193705779	0.000213254	0.000524638
TUT1	0.193702557	0.000213306	0.000524703
C20orf160	-0.193698198	0.000213378	0.000524814
PRDM16	-0.193658693	0.000214026	0.000526344
FBXO6	0.193622405	0.000214624	0.000527748
ACSBG1	-0.193575795	0.000215393	0.000529492
LOC100131	-0.193574834	0.000215409	0.000529492
CAPN13	-0.193574621	0.000215413	0.000529492
OR2A1	-0.193542244	0.000215949	0.000530744
EGLN1	-0.193513791	0.000216421	0.000531839
CCNDBP1	-0.19349981	0.000216653	0.000532345
WWC3	-0.193447754	0.000217521	0.00053441
DKK1	0.193432495	0.000217776	0.00053497
EMB	-0.193407056	0.000218201	0.000535949
KLC4	0.193401692	0.000218291	0.000536103
RAMP3	-0.193324685	0.000219584	0.000539213
BUB3	0.193309564	0.000219839	0.000539772
CBWD6	0.193304316	0.000219927	0.000539923
TSPAN14	-0.193299108	0.000220015	0.000540072
CHAF1A	0.193234274	0.000221111	0.000542696
BCAR1	-0.193193974	0.000221795	0.000544308
DCP1A	-0.193189759	0.000221867	0.000544414
MXD1	-0.193188221	0.000221893	0.000544414
MRC2	-0.193185386	0.000221941	0.000544465
C1orf103	-0.193172634	0.000222158	0.00054493
C20orf106	0.193112616	0.000223182	0.000547374
CSTF3	0.193109766	0.000223231	0.000547426
FMN1	-0.193081703	0.000223712	0.000548537
CD69	-0.193071185	0.000223892	0.000548911
C17orf68	-0.193044352	0.000224352	0.000549973
RNF44	-0.193012936	0.000224893	0.00055123
AS3MT	0.193008315	0.000224972	0.000551357
FAM66A	-0.192974473	0.000225556	0.000552719
IL17RE	0.192956768	0.000225862	0.000553401
TM7SF2	0.192918298	0.000226528	0.000554964
CTGF	-0.19291304	0.000226619	0.000555119
LOC415056	-0.19291057	0.000226662	0.000555156
NDRG3	0.192862094	0.000227504	0.000557151
ZNF263	0.192859424	0.000227551	0.000557196
ADRA2A	-0.192857307	0.000227588	0.000557218
SAMD14	-0.192796946	0.000228641	0.000559728
ZNF44	-0.192757408	0.000229333	0.000561354
TYROBP	0.192746621	0.000229523	0.000561748
BLVRA	0.192721086	0.000229971	0.000562777
C8orf77	0.192705259	0.00023025	0.000563389
RASL11B	-0.192671074	0.000230852	0.000564794

PSPH	0.192575698	0.000232541	0.000568855
AUH	-0.192559953	0.000232821	0.00056947
EXT2	-0.192541283	0.000233153	0.000570213
REEP4	0.192519733	0.000233537	0.000571082
CCDC159	0.192501152	0.000233869	0.000571823
RBM19	0.192483363	0.000234187	0.00057253
SCUBE3	-0.192407908	0.00023554	0.000575767
NPHP4	-0.192396854	0.000235739	0.000576183
FGF2	-0.192381622	0.000236013	0.000576782
SLC8A3	-0.192371823	0.00023619	0.000577143
NEURL3	0.192337074	0.000236817	0.000578604
HSP90B1	0.192333141	0.000236888	0.000578707
LOC283392	-0.192313636	0.000237241	0.000579498
P2RY13	-0.192290261	0.000237664	0.000580461
EXOC3L	-0.192275538	0.000237931	0.000581042
PLCG1	-0.192187916	0.000239527	0.000584832
TAS2R3	-0.192187088	0.000239542	0.000584832
ELOVL1	0.192177879	0.00023971	0.000585171
CTSK	-0.192155044	0.000240128	0.000586119
CUBN	-0.192093765	0.000241252	0.000588791
PPIAL4D	0.192059675	0.00024188	0.00059025
CTXN2	0.192030401	0.00024242	0.000591496
ODZ2	-0.192025166	0.000242516	0.000591659
LRRC23	0.191988473	0.000243195	0.000593243
NPEPL1	0.191975488	0.000243436	0.000593757
XKR6	-0.191955369	0.000243809	0.000594595
CMTM4	-0.191941611	0.000244065	0.000595146
BCL10	-0.191915452	0.000244552	0.000596259
ANXA8	-0.191912609	0.000244605	0.000596315
COX11	0.191904743	0.000244751	0.0005966
SIM1	-0.19189735	0.000244889	0.000596851
CYP2C19	-0.191896006	0.000244914	0.000596851
EFTUD1	-0.191894237	0.000244947	0.000596858
DNAJB13	0.191824318	0.000246254	0.00059997
CORO7	0.191785032	0.000246992	0.000601693
GPR31	-0.191712008	0.000248368	0.000604971
CLDN23	-0.19168072	0.00024896	0.000606339
IMPG2	-0.19156008	0.000251254	0.000611851
APITD1	0.191554015	0.00025137	0.000612059
PRKAR1A	-0.1915386	0.000251664	0.000612701
NUSAP1	0.191520326	0.000252014	0.000613478
AGPAT3	-0.191451032	0.000253345	0.000616641
FAM194A	0.191439047	0.000253575	0.000617127
GGPS1	0.191395499	0.000254416	0.000619097
TNFAIP8L1	-0.191341186	0.000255467	0.000621579
MKI67	0.191322653	0.000255827	0.000622379

ESAM	-0.191282417	0.000256609	0.000624206
CDK2	-0.191263713	0.000256974	0.000625017
REEP3	-0.191229536	0.000257641	0.000626564
FABP1	0.191190606	0.000258404	0.00062834
MOGS	0.191181861	0.000258575	0.000628681
ESM1	0.191165236	0.000258901	0.000629397
CGN	-0.191141182	0.000259374	0.00063047
MOGAT1	-0.191119953	0.000259792	0.000631409
C1QTNF2	-0.191039614	0.00026138	0.000635158
C9orf93	-0.191038668	0.000261399	0.000635158
FAM49B	0.19103573	0.000261457	0.000635222
NHSL1	-0.191027185	0.000261626	0.000635556
PAK2	-0.190986046	0.000262443	0.000637463
ANKRD16	0.190965289	0.000262857	0.000638389
LOC145783	0.190875863	0.000264644	0.000642651
KIF18A	0.190833727	0.00026549	0.000644627
ATP6V1C1	0.190770333	0.000266767	0.00064765
LRCH2	-0.190728613	0.000267611	0.000649567
LOC26102	0.190728063	0.000267622	0.000649567
FGR	-0.19071707	0.000267845	0.000650029
C21orf63	-0.19068614	0.000268473	0.000651473
UBE4B	-0.190677804	0.000268642	0.000651804
OSTM1	-0.190617976	0.000269861	0.000654682
COL9A2	0.190609327	0.000270038	0.000655031
HHLA3	0.190572305	0.000270795	0.000656788
FAM135B	-0.190569015	0.000270862	0.000656871
SPARCL1	-0.190545158	0.000271352	0.000657977
DNAH7	-0.190538242	0.000271494	0.000658241
C12orf12	0.190510376	0.000272066	0.000659484
ATP1A2	-0.190510076	0.000272072	0.000659484
TP53I11	-0.190486739	0.000272553	0.000660568
PAQR9	0.19048223	0.000272646	0.000660713
S100A2	0.190478441	0.000272724	0.000660806
F2RL2	-0.190476158	0.000272771	0.000660806
PRODH2	0.190475553	0.000272783	0.000660806
HIGD1A	-0.190442289	0.00027347	0.000662389
MURC	-0.19043256	0.000273671	0.000662795
GPR142	-0.190416694	0.000274	0.000663442
SECTM1	0.190416442	0.000274005	0.000663442
CNRIP1	-0.190396168	0.000274425	0.000664379
AP3S2	-0.190385333	0.00027465	0.000664842
GPR56	-0.190383197	0.000274694	0.000664868
ZNF575	0.190342515	0.00027554	0.000666834
HMGB1	0.190307634	0.000276267	0.000668512
DBC1	-0.190297654	0.000276475	0.000668935
IFIT3	-0.190243059	0.000277618	0.000671617

CNOT4	-0.19019525	0.000278622	0.000673964
PBLD	-0.190189641	0.00027874	0.000674168
KIAA1239	-0.190179749	0.000278948	0.00067459
SERP1	0.190176005	0.000279027	0.000674692
ADAR	-0.190174527	0.000279058	0.000674692
SCN3A	-0.190135637	0.000279878	0.000676593
GRAP2	-0.19012065	0.000280195	0.000677276
ARHGAP39	0.190088143	0.000280883	0.000678857
PTGER4	-0.190069835	0.000281271	0.000679692
ZNF707	0.190068631	0.000281297	0.000679692
GLT8D1	0.190037309	0.000281962	0.000681218
PYGM	-0.189993802	0.000282889	0.000683374
LAMA1	-0.189934638	0.000284154	0.000686346
ADAMTS5	-0.18992957	0.000284262	0.000686525
NCK2	-0.189845847	0.000286062	0.000690788
GRID1	-0.189811026	0.000286814	0.000692519
FBXL18	0.189792946	0.000287205	0.000693379
LARP6	-0.189790655	0.000287254	0.000693414
LOC285830	0.189772286	0.000287652	0.00069429
SLC3A1	-0.189740379	0.000288344	0.000695877
RAB11FIP4	0.189735146	0.000288458	0.000696067
HABP4	0.189732495	0.000288515	0.000696121
KDM4D	-0.189730271	0.000288564	0.000696153
DGKE	-0.189727015	0.000288635	0.00069624
PYCR1	0.189724968	0.000288679	0.000696263
C3orf17	-0.189672389	0.000289824	0.00069894
KCNC3	-0.189656454	0.000290172	0.000699695
MMS19	0.189614289	0.000291095	0.000701834
TPM3	0.189601777	0.000291369	0.000702411
ELL	-0.189571103	0.000292042	0.000703949
LRP2BP	-0.189527354	0.000293005	0.000706185
HELLS	0.18945131	0.000294686	0.00071015
CAMK1G	-0.189433682	0.000295077	0.000711006
LOC100270	0.189423678	0.000295299	0.000711455
HEXA	0.189395343	0.000295929	0.000712886
CNOT3	0.189393601	0.000295968	0.000712893
ZNF253	-0.189381973	0.000296227	0.00071343
DBNDD2	0.189379944	0.000296272	0.000713453
FUBP1	-0.189363613	0.000296636	0.000714243
FANCB	0.18933474	0.000297281	0.000715709
SRCRB4D	0.189316104	0.000297697	0.000716625
ZNF69	0.189291184	0.000298255	0.000717882
PDXK	0.18928504	0.000298393	0.000718126
GPX8	-0.189268282	0.000298769	0.000718944
INSIG1	-0.189237732	0.000299456	0.00072051
PRSS50	0.189226079	0.000299718	0.000721053

SGOL1	0.189216657	0.00029993	0.000721477
ERICH1	-0.189173784	0.000300897	0.000723717
CNFN	0.18914286	0.000301597	0.000725312
ADAM20	-0.189120173	0.000302111	0.000726461
KIAA1009	-0.189028159	0.000304205	0.000731332
CPNE9	0.189027926	0.000304211	0.000731332
ZNF238	-0.18898764	0.000305132	0.000733458
APOL3	-0.188944585	0.000306119	0.000735742
KIAA0087	-0.188908545	0.000306948	0.000737645
COG2	0.188903822	0.000307056	0.000737817
PANX1	-0.188806759	0.000309299	0.000743117
GJA9	0.1887686	0.000310185	0.000745155
UCA1	-0.18874085	0.000310831	0.000746617
COL29A1	-0.188687766	0.00031207	0.000749502
TSPAN5	-0.18861533	0.000313768	0.000753489
WWC1	-0.188597312	0.000314191	0.000754415
ARHGEF18	-0.188540286	0.000315536	0.000757552
FBXL16	-0.188528112	0.000315823	0.000758151
GRIPAP1	-0.188516165	0.000316106	0.000758738
LOC100134	0.188492328	0.00031667	0.000760002
PYGL	-0.188487325	0.000316789	0.000760195
TUG1	-0.188464498	0.000317331	0.000761403
POM121L10	0.188457416	0.000317499	0.000761714
TFDP1	-0.188429188	0.00031817	0.000763233
NR1I2	-0.188309019	0.000321043	0.000770032
EFR3B	-0.188254233	0.000322361	0.000773099
CD79B	0.188206748	0.000323507	0.000775755
TUBAL3	0.188198021	0.000323718	0.000776167
CYP3A43	-0.188194265	0.000323809	0.000776292
TRPS1	-0.188189399	0.000323927	0.000776472
METT5D1	-0.188187943	0.000323962	0.000776472
TRIP6	0.188082665	0.00032652	0.000782509
SLC25A14	0.188031976	0.000327758	0.000785382
CLDN19	0.187937937	0.000330067	0.00079082
DSEL	-0.18790698	0.000330831	0.000792554
ZNF37B	-0.187897515	0.000331065	0.000793018
FCGRT	0.187831491	0.000332699	0.000796838
C5orf48	0.187789212	0.00033375	0.000799259
LOC285359	0.187755252	0.000334597	0.000801189
RINT1	-0.187737498	0.00033504	0.000802154
CYP39A1	-0.18769093	0.000336205	0.000804847
GRHL1	-0.187564898	0.000339377	0.000812344
ASB16	-0.187553542	0.000339665	0.000812933
C21orf34	-0.187503863	0.000340924	0.000815849
FLJ36777	0.187490191	0.000341271	0.000816582
PDCD1LG2	-0.187460854	0.000342017	0.000818214

ACTA1	0.187460154	0.000342035	0.000818214
CNIH2	0.187455312	0.000342158	0.000818321
ELP4	0.187455161	0.000342162	0.000818321
CYP8B1	-0.187413747	0.000343219	0.000820749
RAB32	0.187399656	0.000343579	0.000821512
WIPF1	-0.187376043	0.000344183	0.000822809
SMTNL1	-0.187375234	0.000344204	0.000822809
PNRC2	-0.18736758	0.0003444	0.000823179
IGSF9	-0.187365283	0.000344459	0.000823221
PMPCA	0.187355016	0.000344722	0.000823751
CCNE1	0.187334993	0.000345236	0.000824881
EDN2	-0.187280559	0.000346637	0.000828128
MMP9	0.187270952	0.000346885	0.000828621
TBC1D3B	0.187254467	0.000347311	0.000829538
TAF4	-0.187231589	0.000347902	0.000830851
CKMT1B	0.187229662	0.000347952	0.000830871
AVL9	-0.187196905	0.0003488	0.000832797
HIST2H2BF	-0.187187728	0.000349039	0.000833258
SLC12A2	-0.187186237	0.000349077	0.000833258
NCBP2	0.187180602	0.000349223	0.000833507
NUP93	0.18717184	0.000349451	0.000833951
FAM99A	-0.187156035	0.000349862	0.000834831
HORMAD2	-0.187151714	0.000349974	0.000834999
ARSE	0.187145416	0.000350138	0.00083529
AP3M2	-0.187117051	0.000350877	0.000836953
GPR171	-0.187076577	0.000351934	0.000839373
TOP3A	-0.187069204	0.000352127	0.000839733
GPRC6A	0.186976899	0.00035455	0.00084541
KIAA1024	-0.18693629	0.000355621	0.000847862
SOX17	-0.18691244	0.000356251	0.000849263
KIF12	0.186909508	0.000356329	0.000849346
ALDH2	-0.186884139	0.000357	0.000850845
NT5C1A	-0.186878126	0.00035716	0.000851123
CRYBB3	0.186838111	0.000358222	0.000853553
CYP4F2	-0.186799332	0.000359255	0.00085591
NPW	0.186785213	0.000359631	0.000856705
MIP	-0.186735686	0.000360955	0.000859756
DCTN5	0.186732231	0.000361047	0.000859873
PACSIN1	0.186711311	0.000361608	0.000861106
ST8SIA4	-0.186692107	0.000362124	0.00086223
MYO1C	-0.186641669	0.00036348	0.000865357
TERC	0.186633848	0.000363691	0.000865756
KIF1A	-0.186580405	0.000365135	0.000869089
G3BP1	-0.186544885	0.000366098	0.000871275
LY96	0.186521018	0.000366746	0.000872713
MFSD6	-0.186518809	0.000366806	0.000872752

LOC283070	-0.186366963	0.000370954	0.000882518
C6orf208	-0.186360525	0.000371131	0.000882833
HMHB1	0.186355845	0.00037126	0.000883033
C3orf35	0.186352241	0.000371359	0.000883164
FBXO40	-0.186329577	0.000371982	0.000884541
CYP2A7	-0.186318128	0.000372298	0.000885185
ALOX12	-0.186308326	0.000372568	0.000885722
SNAPC1	-0.186289207	0.000373096	0.00088687
C16orf54	-0.186269773	0.000373633	0.000888041
SAPS3	-0.186256892	0.000373989	0.000888782
LHX2	-0.186202965	0.000375484	0.000892228
SUFU	-0.186143048	0.000377152	0.000896085
EVC	-0.186109342	0.000378093	0.000898214
LCN2	0.186043897	0.000379927	0.000902462
SIGLEC5	-0.185850473	0.000385395	0.000915342
MADD	-0.185808714	0.000386585	0.000918059
BIRC7	0.185794317	0.000386996	0.000918926
CXorf36	-0.185777376	0.000387481	0.000919966
ALG2	-0.18571225	0.000389348	0.000924289
RBM45	0.185662081	0.000390791	0.000927606
TRIM15	0.185659443	0.000390868	0.000927676
USP4	-0.185642057	0.000391369	0.000928756
UCHL5	-0.185638039	0.000391485	0.00092892
FAM200B	0.185618974	0.000392036	0.000930117
HSDL2	-0.185606019	0.000392411	0.000930895
TNNT1	0.185603205	0.000392492	0.000930977
ASAM	-0.185581527	0.00039312	0.000932356
C2orf53	0.185568888	0.000393487	0.000933114
CRCP	-0.18556218	0.000393682	0.000933464
DLAT	-0.185526781	0.00039471	0.000935792
MYO7A	-0.185523285	0.000394812	0.000935893
PAGE2B	0.18552209	0.000394847	0.000935893
MRS2P2	0.185515621	0.000395035	0.000936228
TREML3	0.185500332	0.000395481	0.000937172
CYP4F22	-0.185472951	0.00039628	0.000938954
YWHAZ	0.185433504	0.000397433	0.000941576
AMBP	0.185431395	0.000397495	0.00094161
GLTPD2	0.18542721	0.000397618	0.000941788
RTDR1	0.185411375	0.000398082	0.000942776
RAB27B	-0.185397979	0.000398475	0.000943594
CCDC93	-0.185394931	0.000398564	0.000943694
SOAT1	-0.185373631	0.00039919	0.000944997
ZNF615	-0.185372974	0.00039921	0.000944997
INVS	-0.185367498	0.000399371	0.000945266
CIZ1	0.185341558	0.000400134	0.000946961
PCNP	-0.185324365	0.000400641	0.000948048

WNT7A	-0.185314478	0.000400933	0.000948626
C9orf150	-0.185281363	0.000401912	0.000950829
CCDC99	0.185221051	0.000403701	0.000954947
COX7B2	0.185218273	0.000403783	0.000955029
MAP3K6	-0.185208474	0.000404074	0.000955604
RNF216L	0.185196332	0.000404436	0.000956345
ST8SIA5	0.185189848	0.000404629	0.000956688
HTR2B	-0.185166067	0.000405338	0.00095825
MYRIP	-0.185150184	0.000405812	0.000959257
RAP2A	-0.185147059	0.000405905	0.000959289
NMRAL1	0.185146499	0.000405922	0.000959289
CTH	-0.185139773	0.000406122	0.00095965
SFRS7	0.185113864	0.000406897	0.000961367
AGXT2L2	-0.18509243	0.000407539	0.00096277
TM9SF3	-0.185088406	0.00040766	0.00096294
MORF4L1	0.185085671	0.000407742	0.00096302
NCL	0.185073376	0.000408111	0.000963777
AMIGO2	-0.18505979	0.000408519	0.000964626
IKZF1	-0.185054546	0.000408677	0.000964884
PIK3AP1	-0.185029486	0.00040943	0.000966549
EPO	0.185022394	0.000409644	0.000966938
MRPS22	0.185011405	0.000409975	0.000967515
KLRC4	-0.185011066	0.000409985	0.000967515
AKR1B10	0.184992293	0.000410552	0.000968736
PPP1R3D	-0.184972359	0.000411154	0.000970042
SSR3	0.184968236	0.000411278	0.000970221
ENPP1	-0.184957764	0.000411595	0.000970853
FLJ40852	-0.184951214	0.000411793	0.000971206
ALDH8A1	-0.184938342	0.000412183	0.00097201
ART5	0.184923427	0.000412635	0.000972961
SNAI2	-0.184889886	0.000413653	0.000975247
GNB5	-0.184882915	0.000413865	0.000975631
G0S2	-0.18487193	0.0004142	0.000976303
EVI2B	-0.184848933	0.0004149	0.000977838
SETDB1	0.184823166	0.000415686	0.000979575
CLDN3	0.184812924	0.000415999	0.000980196
C14orf19	0.184724204	0.000418718	0.000986487
CST8	0.18470355	0.000419354	0.000987867
PRPSAP2	0.184690078	0.000419768	0.000988727
OR10A3	0.18468094	0.00042005	0.000989274
MAP3K11	0.184662913	0.000420606	0.000990466
GLTSCR1	-0.184655355	0.00042084	0.000990899
MUC6	-0.184586284	0.000422978	0.000995816
LOC339240	-0.184584004	0.000423049	0.000995865
C7orf46	-0.184576436	0.000423284	0.0009963
SMAD5OS	0.184571249	0.000423445	0.000996562

ANKRD10	-0.184555095	0.000423947	0.000997625
FAM47E	-0.184530947	0.000424699	0.000999276
TSLP	-0.184528728	0.000424768	0.00099932
FAM124A	-0.184520752	0.000425016	0.000999787
BMS1P5	-0.18447687	0.000426386	0.001002891
SH2B2	0.184473806	0.000426482	0.001002998
NRK	-0.184460178	0.000426908	0.001003882
KIAA1731	-0.184442519	0.000427461	0.001005064
SCOC	-0.18443739	0.000427622	0.001005323
SMCR7L	-0.184426059	0.000427977	0.00100604
TLL2	0.184422561	0.000428087	0.001006179
APOH	0.184409019	0.000428512	0.001007009
LOXHD1	-0.184408097	0.000428541	0.001007009
FAM43B	-0.184405066	0.000428637	0.001007114
THOC1	0.184397562	0.000428872	0.001007549
C14orf184	-0.18434676	0.000430472	0.001011188
STX7	-0.184309441	0.000431651	0.001013837
ORC2L	-0.18428505	0.000432423	0.00101553
C2orf34	0.184274428	0.000432759	0.001016201
KIF26B	-0.18425171	0.00043348	0.001017773
ABCB11	-0.184242532	0.000433771	0.001018338
SLC25A25	-0.184237367	0.000433936	0.001018603
CAMKK2	-0.184231176	0.000434132	0.001018892
GFPT2	-0.184230268	0.000434161	0.001018892
PALLD	-0.184213572	0.000434692	0.001020019
MFSD4	-0.184205661	0.000434944	0.00102049
USP43	-0.184201285	0.000435084	0.001020696
GNAZ	0.184165383	0.000436229	0.001023262
GEFT	-0.184147959	0.000436786	0.001024448
SLC17A7	-0.184144902	0.000436883	0.001024556
SRGAP2	-0.184118957	0.000437714	0.001026383
BLK	-0.18409623	0.000438442	0.00102797
ZNF573	-0.18407977	0.000438971	0.001029088
TP53BP2	-0.184045474	0.000440074	0.001031552
ITGA6	-0.184039106	0.000440279	0.001031798
MED22	0.184038998	0.000440282	0.001031798
AMN	0.184031679	0.000440518	0.00103223
GAGE1	0.184021909	0.000440833	0.001032846
SPAG8	0.183904247	0.000444643	0.001041649
PRDM8	-0.183872978	0.00044566	0.001043903
DSG1	-0.183871462	0.00044571	0.001043903
CLEC9A	-0.183847759	0.000446483	0.00104559
ERLEC1	-0.183831702	0.000447007	0.001046695
ANKRD35	-0.183823987	0.000447259	0.001047162
APBB1IP	-0.183812745	0.000447627	0.0010479
SLC1A4	0.183806419	0.000447834	0.001048261

SLC1A5	0.183801643	0.00044799	0.001048504
GUF1	-0.183739783	0.000450019	0.001053129
ARPC1B	0.183716129	0.000450797	0.001054826
ZNF160	-0.183696987	0.000451428	0.00105616
C2orf58	-0.183695606	0.000451473	0.00105616
GRHL3	-0.183643695	0.000453188	0.001060047
TIAM2	-0.183636351	0.000453431	0.001060491
MYCBP	0.183624802	0.000453814	0.001061261
GLS2	-0.18360599	0.000454437	0.001062595
LIM2	0.183575255	0.000455458	0.001064856
STX1B	-0.18354913	0.000456327	0.001066763
PRCP	-0.183529363	0.000456986	0.001068177
KLK2	0.183515905	0.000457435	0.001069101
TRIM68	-0.183486349	0.000458422	0.001071283
DNAH2	-0.183436649	0.000460087	0.001075048
SHCBP1	0.183427861	0.000460382	0.001075611
MPPED1	-0.18334157	0.000463288	0.001082273
LOC644172	-0.183286999	0.000465134	0.001086459
ACTB	0.183280472	0.000465355	0.001086848
LMBR1L	0.183259014	0.000466084	0.001088421
WBP4	-0.183237354	0.00046682	0.001090013
XAGE1D	0.183233431	0.000466953	0.001090196
FAM19A2	-0.183208065	0.000467817	0.001092085
GDAP1L1	0.183175017	0.000468945	0.001094589
ERBB2	-0.183148319	0.000469857	0.00109659
CHRNA7	-0.183094676	0.000471696	0.001100753
C7orf45	-0.183076822	0.00047231	0.001102056
C9orf144B	-0.183070902	0.000472513	0.001102402
PCDHB16	-0.183047951	0.000473303	0.001104115
ZNF655	-0.183037012	0.00047368	0.001104865
C4orf21	-0.183022769	0.000474171	0.001105882
TPT1	0.183013704	0.000474484	0.001106482
CASP10	-0.1829761	0.000475784	0.001109268
GSTA4	0.182975934	0.00047579	0.001109268
OR56B1	-0.182948555	0.000476739	0.001111349
COL7A1	0.1829436	0.000476911	0.00111162
C4orf48	0.182816267	0.000481348	0.00112183
CCDC47	-0.182775142	0.000482789	0.001125057
ITGA7	-0.182762135	0.000483245	0.001125945
TMED2	-0.182761066	0.000483283	0.001125945
LYRM7	-0.182717986	0.000484798	0.001129343
PIGA	-0.18270606	0.000485218	0.00113019
TCP1	0.18267743	0.000486229	0.001132295
PDDC1	0.182677236	0.000486236	0.001132295
PIK3IP1	-0.182672481	0.000486404	0.001132553
RNGTT	-0.182658753	0.000486889	0.001133551

KIAA0513	-0.182610452	0.0004886	0.001137402
STK11IP	0.182596204	0.000489106	0.001138446
EFNA1	0.182584891	0.000489508	0.001139248
POGZ	-0.182548035	0.000490819	0.001142168
NFXL1	-0.182535301	0.000491273	0.00114309
INPP4B	-0.182466519	0.000493732	0.001148677
GOLGA6C	-0.182436289	0.000494816	0.001151065
L3MBTL3	-0.182434397	0.000494884	0.001151089
PLAU	-0.182411874	0.000495694	0.001152836
MYCL1	-0.182378789	0.000496885	0.001155472
C5orf32	0.18235309	0.000497812	0.001157492
RELB	0.18233306	0.000498535	0.001159039
PHF7	0.182327817	0.000498725	0.001159345
PDCD11	0.182323616	0.000498877	0.001159563
IMPAD1	-0.18230439	0.000499573	0.001161045
GNPDA2	-0.182293257	0.000499976	0.001161847
PFKFB4	0.182266485	0.000500948	0.001163968
AGRN	-0.182249531	0.000501564	0.001165264
ICAM2	0.182244091	0.000501761	0.001165587
SLC7A5P1	-0.182231467	0.000502221	0.001166518
CASP3	-0.182106986	0.000506771	0.00117695
C16orf5	0.182087581	0.000507484	0.001178468
ZNF662	-0.182066302	0.000508266	0.001180148
DAND5	0.182011655	0.000510282	0.001184688
SYDE1	-0.181986235	0.000511221	0.001186732
FMO3	-0.181967372	0.00051192	0.001188215
GTPBP2	0.18193606	0.000513081	0.001190705
INHBB	-0.181935227	0.000513112	0.001190705
MANBA	-0.181902014	0.000514347	0.001193431
ZBED4	-0.181888827	0.000514838	0.001194431
TPBG	-0.18187216	0.000515459	0.001195733
ZNF485	0.181841681	0.000516597	0.001198233
NIT2	0.18182085	0.000517376	0.0011999
SNORA74A	0.181771359	0.000519231	0.001204062
GTPBP3	0.18175779	0.00051974	0.001205103
NADK	-0.181733644	0.000520649	0.001207068
TAF6	0.181652747	0.000523702	0.001214005
YOD1	-0.181649604	0.000523821	0.00121414
SDC4P	-0.181483478	0.000530144	0.001228653
C6orf47	0.181464261	0.00053088	0.001230216
THUMPD2	0.181447986	0.000531504	0.001231519
ICA1L	-0.181381927	0.000534044	0.00123726
SMN2	0.181345923	0.000535433	0.001240334
PPM1H	-0.181276728	0.000538112	0.001246395
TCF15	0.181241393	0.000539485	0.00124943
FAM168B	-0.181223621	0.000540177	0.001250887

PWRN1	-0.181215861	0.000540479	0.001251441
SERPINA13	0.181206619	0.00054084	0.00125213
TPCN2	-0.181201089	0.000541055	0.001252484
WISP2	-0.1811706	0.000542246	0.001255094
GNRHR2	0.181151559	0.00054299	0.001256671
RASA4	-0.181149363	0.000543076	0.001256724
RPL23AP7	0.181128332	0.0005439	0.001258484
CDK5RAP2	0.181071986	0.000546113	0.001263457
FXYD6	-0.181053367	0.000546846	0.001264999
CARD16	0.181051828	0.000546907	0.001264999
CRB1	-0.181046865	0.000547102	0.001265305
SLC16A3	0.181003784	0.000548802	0.00126909
PPP3CC	-0.180998423	0.000549014	0.001269432
SAMD13	0.180976383	0.000549886	0.001271301
PIK3CB	-0.180950233	0.000550923	0.001273549
LRRC17	-0.180945117	0.000551126	0.00127387
CDKN1B	-0.180941914	0.000551253	0.001274016
CHRFAM7A	-0.180908312	0.000552588	0.001276954
PPFIA2	-0.180805792	0.00055668	0.00128626
LOXL1	-0.180796923	0.000557035	0.001286932
PRC1	0.180792621	0.000557207	0.001287181
HERPUD2	-0.180781207	0.000557665	0.001288089
PPT2	0.180715221	0.000560318	0.001294066
PRIMA1	-0.18069561	0.000561109	0.001295732
LOC728264	-0.180694112	0.000561169	0.001295732
C15orf23	0.180662497	0.000562446	0.00129853
HPS1	0.18065614	0.000562703	0.001298942
U2AF2	0.180654859	0.000562755	0.001298942
DND1	-0.18060043	0.000564961	0.001303882
C5orf46	0.180563394	0.000566467	0.001307205
PGK2	0.180558452	0.000566668	0.001307518
MPP1	-0.18055644	0.00056675	0.001307556
SNORA57	0.180512462	0.000568543	0.001311541
MAGEA6	0.180486954	0.000569586	0.001313794
GADD45B	-0.180484537	0.000569685	0.00131387
AKR1A1	0.180473697	0.000570128	0.001314709
TRIM69	-0.180472432	0.00057018	0.001314709
TXNDC15	-0.180424593	0.000572142	0.00131908
TRIP10	0.180411637	0.000572675	0.001320155
PRDM5	-0.180401541	0.00057309	0.001320959
CCT8	0.180364499	0.000574616	0.001324323
TGFB1I1	-0.180301636	0.000577214	0.001330157
CAPZA1	-0.180266066	0.000578689	0.001333402
LCA5L	-0.180172702	0.000582577	0.001342206
DLX4	0.180141801	0.000583869	0.001345027
GLTP	0.180114353	0.00058502	0.001347521

PIGZ	0.180088369	0.00058611	0.001349877
TMEM167B	-0.180046655	0.000587865	0.001353762
RPP14	-0.179958006	0.00059161	0.001362229
NBR1	-0.179947155	0.00059207	0.001363131
CC2D1B	0.17990291	0.000593949	0.001367299
COL2A1	0.179888911	0.000594545	0.001368512
LAMB2L	0.179871469	0.000595288	0.001369987
SERPINE1	-0.179870631	0.000595324	0.001369987
TAS2R60	-0.179861826	0.000595699	0.001370693
MYOM2	-0.17983509	0.00059684	0.00137316
CCDC88B	0.179792044	0.000598682	0.001377238
RRH	-0.179789745	0.00059878	0.001377306
STEAP3	-0.179706494	0.000602358	0.001385374
MCF2L2	-0.179699172	0.000602673	0.00138594
CIAO1	0.179690285	0.000603056	0.001386661
TBCE	0.179656589	0.000604511	0.001389846
GBA2	-0.179644227	0.000605046	0.001390915
ARHGAP4	0.179631054	0.000605616	0.001392065
FGFR1OP	-0.179566636	0.000608412	0.001398258
FAM48A	-0.179565733	0.000608451	0.001398258
C1orf21	-0.179562014	0.000608613	0.001398468
GIMAP4	-0.179542467	0.000609464	0.001400262
ISL2	0.179526151	0.000610175	0.001401734
TMEM69	0.179515696	0.000610631	0.001402505
HIST2H4A	0.179515228	0.000610651	0.001402505
HDC	-0.179503712	0.000611154	0.001403498
RFFL	-0.179498776	0.00061137	0.001403814
ACSF2	0.179497329	0.000611433	0.001403814
ACTR8	0.179491521	0.000611687	0.001404235
CHRNA10	0.179385369	0.000616342	0.00141476
C6orf203	0.179377027	0.00061671	0.00141544
C10orf119	-0.179372728	0.000616899	0.001415711
PACRGL	-0.17936718	0.000617143	0.001416109
RPRD1A	-0.179345613	0.000618095	0.001418128
STXBP4	-0.179291305	0.000620495	0.001423473
C14orf126	-0.17928942	0.000620579	0.0014235
SOX8	-0.179252389	0.000622221	0.001427023
LOC550112	-0.179251558	0.000622258	0.001427023
RPS28	0.179249211	0.000622362	0.001427098
ID4	-0.179222368	0.000623556	0.001429669
SF3B2	0.179216968	0.000623796	0.001430056
C12orf68	-0.179206014	0.000624284	0.001430877
PCNT	-0.179205699	0.000624298	0.001430877
IKBKG	0.17918408	0.000625261	0.00143292
TNFAIP6	-0.179180101	0.000625439	0.001433162
TMPRSS2	-0.179146778	0.000626927	0.001436246

PNMA6A	0.179146735	0.000626929	0.001436246
NPR1	-0.179137476	0.000627343	0.00143703
TM6SF2	-0.179116587	0.000628278	0.001438859
FAM83B	-0.1791158	0.000628314	0.001438859
WDR54	0.179114798	0.000628358	0.001438859
STK16	0.179081069	0.000629872	0.001442158
SUDS3	-0.179068627	0.000630431	0.001443272
POFUT2	-0.179057397	0.000630935	0.001444262
SGOL2	0.178980976	0.000634381	0.001451983
NUDT21	-0.178963955	0.000635151	0.001453578
HHAT	-0.17883105	0.000641193	0.001467236
FKBP5	-0.178770741	0.000643952	0.00147338
WDR12	0.17869185	0.000647577	0.001481505
CES7	-0.178686942	0.000647803	0.001481852
LOC441601	0.17868358	0.000647958	0.001482036
C17orf39	-0.178666951	0.000648725	0.00148362
ATP8A2	-0.178643663	0.000649801	0.00148591
HSD3B1	-0.178608091	0.000651447	0.001489503
LMO2	-0.17855607	0.000653862	0.001494852
MSX2	-0.178531198	0.000655019	0.001497325
C19orf23	0.178525713	0.000655274	0.001497737
C5orf55	0.178501175	0.000656418	0.00150018
CCNA2	0.178466089	0.000658057	0.001503688
SIVA1	0.17846499	0.000658109	0.001503688
DUSP3	-0.178463471	0.00065818	0.001503688
PROM2	-0.178455843	0.000658537	0.001504331
GOLGA7	-0.178452286	0.000658703	0.001504538
RSU1	-0.17839775	0.00066126	0.001510206
RFX6	0.17839211	0.000661525	0.001510638
ZBTB5	-0.178388881	0.000661677	0.001510812
CCDC8	-0.178312538	0.000665275	0.001518852
TMEM54	0.178305273	0.000665618	0.001519461
PPP2CA	0.178302305	0.000665758	0.001519607
AFG3L1	-0.178283788	0.000666634	0.001521432
PGAM1	0.178276847	0.000666963	0.001522008
C12orf74	-0.178239796	0.000668719	0.001525842
TSPO	0.178231303	0.000669123	0.001526587
LOC647121	-0.178169859	0.000672047	0.001533083
SLC2A12	-0.178147785	0.0006731	0.00153531
CABIN1	-0.178121226	0.00067437	0.001538029
ANGPTL6	-0.178101383	0.000675319	0.001540019
FLJ44635	0.178055653	0.000677513	0.001544845
DCAF11	-0.178041233	0.000678206	0.001546248
HUS1	-0.178006997	0.000679855	0.001549829
C2CD2	-0.177995061	0.00068043	0.001550963
ANXA7	-0.177991031	0.000680625	0.001551229

BLM	0.177973861	0.000681454	0.001552941
C11orf49	0.177959704	0.000682138	0.001554322
BTBD12	-0.177945237	0.000682838	0.001555738
CRIP1	0.177924797	0.000683827	0.001557815
SMOC2	-0.17791061	0.000684515	0.001559204
KRTAP19-5	0.177888275	0.000685599	0.001561495
ATP6V1C2	0.177884888	0.000685764	0.001561691
C6orf120	-0.17782352	0.000688752	0.001568316
DDX46	-0.1777796	0.000690897	0.001573021
EDC4	0.177762473	0.000691736	0.00157475
GMPR	-0.177737437	0.000692963	0.001577364
LOC143188	0.177681276	0.000695723	0.001583466
MYADM	-0.177625548	0.000698472	0.001589541
NLGN2	-0.177621783	0.000698658	0.001589783
MAP1D	0.17761236	0.000699124	0.001590661
BATF3	0.17760941	0.00069927	0.001590811
LOC401093	-0.177591664	0.000700148	0.001592628
PI4KAP2	-0.177588109	0.000700325	0.001592847
NCRNA0016	0.177568417	0.000701301	0.001594885
SPATA17	0.177531544	0.000703132	0.001598867
PTPRT	-0.177480578	0.000705671	0.001604456
LOC388387	-0.177428203	0.000708288	0.001610223
OTUD3	-0.177420418	0.000708678	0.001610926
REPS1	-0.177378682	0.000710771	0.0016155
VIM	-0.177362909	0.000711564	0.001617117
DNAH8	0.177255144	0.000717001	0.001629287
TBC1D9	-0.177229699	0.00071829	0.001632031
IAH1	0.177216235	0.000718973	0.001633397
TIGD2	-0.177203844	0.000719603	0.00163464
TMEM44	0.177200888	0.000719753	0.001634794
DCAF6	-0.17719481	0.000720062	0.00163531
KCNK1	-0.17715996	0.000721835	0.00163915
OTX1	-0.177113371	0.000724212	0.001644361
IKBIP	0.177100677	0.000724861	0.001645647
ZNF75A	0.177080357	0.000725901	0.00164782
PVRIG	-0.177063164	0.000726782	0.001649632
ZNF134	-0.176991309	0.000730475	0.001657824
ZNF672	0.176983862	0.000730859	0.001658506
EIF4E	-0.17695825	0.00073218	0.001661315
SPA17	0.176954262	0.000732386	0.001661593
ZSCAN12P1	-0.176935013	0.00073338	0.001663659
TSPAN12	-0.176904023	0.000734984	0.001667108
P2RY14	-0.176848747	0.000737853	0.001673424
GPR161	-0.176843364	0.000738133	0.001673868
DDX55	0.17683791	0.000738416	0.001674321
TESC	0.176825097	0.000739083	0.001675642

ZBTB41	-0.176815307	0.000739593	0.001676608
NTSR1	-0.176717746	0.000744693	0.001687976
SRRM5	0.176701595	0.00074554	0.001689705
HMGB4	-0.176675972	0.000746886	0.001692563
PITX3	0.176665558	0.000747434	0.001693611
MAP1A	-0.176642366	0.000748655	0.001696186
KDM4DL	-0.176622781	0.000749688	0.001698332
AKR1D1	-0.176571547	0.000752396	0.001704272
TMEM53	0.176563078	0.000752844	0.001705094
TBL2	0.176549358	0.000753571	0.001706547
LIN28B	0.176511631	0.000755574	0.001710887
LCORL	-0.176413809	0.000760789	0.001722501
OR10AD1	-0.176402478	0.000761396	0.001723678
MEF2D	-0.176387352	0.000762206	0.001725315
ZRSR2	0.176382314	0.000762476	0.00172573
TMEM168	-0.176368008	0.000763243	0.00172727
SNORD115-	-0.176365154	0.000763396	0.00172742
SPDYE6	-0.176341378	0.000764672	0.00173004
CDC16	0.176340358	0.000764727	0.00173004
RBM12	-0.176319332	0.000765858	0.001732401
TMEM68	0.176278155	0.000768077	0.001737223
CISH	-0.176262513	0.000768921	0.001738936
TMEM19	-0.176228834	0.000770742	0.001742856
RAB15	0.176216994	0.000771384	0.001744108
CACNG8	-0.176193302	0.000772668	0.001746814
CREB5	-0.17616382	0.000774269	0.001750235
RASGRP1	-0.176154889	0.000774755	0.001751134
ZDHHC19	0.176132065	0.000775997	0.001753743
VWF	-0.176127202	0.000776262	0.001754143
SDCCAG3	0.176123292	0.000776475	0.001754425
IP6K3	-0.176097315	0.000777892	0.001757428
OR52W1	-0.176046014	0.000780698	0.001763481
GPC1	-0.176045078	0.000780749	0.001763481
GAB3	-0.176032348	0.000781447	0.001764857
DKKL1	0.175976908	0.000784492	0.001771533
PIP4K2A	-0.175967617	0.000785003	0.001772487
C16orf46	-0.17596563	0.000785112	0.001772533
OR2A7	-0.17596214	0.000785305	0.001772766
ZDHHC1	-0.175909604	0.000788203	0.001779107
MORF4L2	-0.175901599	0.000788646	0.001779904
NKAIN1	0.175882011	0.000789729	0.001782148
FLJ35390	-0.175877352	0.000789987	0.001782529
XIST	-0.175854049	0.000791279	0.00178524
PTCD2	-0.175813671	0.000793521	0.001790097
CYP2B7P1	-0.175802883	0.000794121	0.001791248
COPA	-0.175788923	0.000794899	0.001792798

PGBD1	0.175786331	0.000795043	0.00179292
CACNG1	0.175745535	0.000797319	0.001797849
PKD2L2	-0.175737842	0.000797748	0.001798614
SFRP4	-0.175715843	0.000798979	0.001801184
TPST1	-0.175677334	0.000801136	0.001805844
LOC400759	-0.175572756	0.000807023	0.001818908
KIAA1407	-0.17546822	0.000812948	0.001832053
FURIN	-0.17545645	0.000813617	0.001833354
CECR2	-0.175451892	0.000813877	0.001833732
SHMT2	0.175420006	0.000815694	0.001837618
MMP24	0.175416713	0.000815882	0.001837833
MRAP2	0.175403427	0.00081664	0.001839281
PAGE5	0.175399302	0.000816875	0.001839281
VIL1	0.175397865	0.000816958	0.001839281
ZNF684	-0.175397483	0.000816979	0.001839281
PRPF39	-0.175397364	0.000816986	0.001839281
GNASAS	0.175390808	0.000817361	0.001839916
MRFAP1	0.175387691	0.000817539	0.001840109
TNP1	0.175385642	0.000817656	0.001840165
ABHD11	0.175356422	0.000819328	0.00184372
C1orf83	-0.175305367	0.000822257	0.001849936
CHRNB1	-0.175305035	0.000822276	0.001849936
C9orf25	-0.175298298	0.000822664	0.001850598
CCDC68	-0.175227613	0.000826737	0.001859551
ACP1	0.175199248	0.000828377	0.001863029
VWCE	0.175174968	0.000829783	0.00186598
EFHA1	-0.175120769	0.000832929	0.001872844
UBE2Q1	0.175114317	0.000833304	0.001873477
WNT6	0.175107932	0.000833676	0.001874101
ZNF624	-0.175097648	0.000834275	0.001875213
TMEM41B	0.1750962	0.000834359	0.001875213
AGAP8	-0.17508831	0.000834819	0.001876034
ZNF611	-0.175041379	0.000837558	0.001881977
EFCAB7	-0.174999955	0.000839983	0.001887213
PKIG	0.174993669	0.000840351	0.001887828
KCTD6	0.17498559	0.000840825	0.001888679
ZNF665	-0.174902287	0.000845725	0.001899471
KCNK13	-0.174879184	0.000847089	0.001902185
SIX2	0.174878578	0.000847124	0.001902185
SYVN1	-0.174866967	0.000847811	0.001903511
F2RL1	-0.174854335	0.000848558	0.001904873
C2orf76	0.174852353	0.000848675	0.001904873
PTP4A1	-0.174851861	0.000848704	0.001904873
MTA3	0.17482606	0.000850232	0.001908087
ANKRD7	0.1747822	0.000852835	0.001913714
SFRS4	-0.174768355	0.000853658	0.001915346

ZNF860	-0.17476041	0.000854131	0.001916191
C2orf67	-0.17475837	0.000854253	0.001916247
HNRNPU	0.174738221	0.000855453	0.001918723
NR0B1	0.174729374	0.00085598	0.00191969
C8orf46	-0.174720693	0.000856498	0.001920635
ARHGAP10	-0.174710192	0.000857125	0.001921628
ARL6IP5	-0.174710044	0.000857134	0.001921628
CAPSL	0.174677374	0.000859087	0.00192579
DGCR9	-0.174648433	0.00086082	0.001929458
LARP1	-0.174622051	0.000862403	0.001932789
ABCF2	0.174618715	0.000862604	0.001933021
P2RY8	-0.174583407	0.000864727	0.00193756
C12orf43	0.174548926	0.000866805	0.001941998
GPHA2	0.17454013	0.000867336	0.001942969
ATG16L2	-0.174523115	0.000868364	0.001945053
SYT10	-0.174473799	0.000871349	0.00195152
IPCEF1	-0.174445737	0.000873052	0.001955114
RMND5B	0.174438961	0.000873464	0.001955816
PHLDA1	-0.174384431	0.000876783	0.001963028
CDKL3	0.17436824	0.000877771	0.001965018
R3HCC1	0.17430436	0.000881678	0.001973544
ZGPAT	0.174258281	0.000884506	0.001979652
C20orf165	-0.174231987	0.000886124	0.00198305
CADM1	-0.174207131	0.000887656	0.001986255
FOXM1	0.174145558	0.000891461	0.001994449
AGBL2	-0.174144275	0.00089154	0.001994449
GABRP	-0.174143019	0.000891618	0.001994449
PCNXL2	-0.174115594	0.000893318	0.001998027
FAM160B2	-0.174093961	0.000894661	0.002000806
RAB9B	-0.174088127	0.000895024	0.002001392
ZNF414	0.174053214	0.000897196	0.002006024
CAPN5	-0.174037496	0.000898175	0.002007989
BUB1B	0.174009936	0.000899895	0.002011608
UBTD2	-0.174005327	0.000900183	0.002012026
CWC25	-0.17399901	0.000900578	0.002012682
C14orf142	0.173988615	0.000901228	0.002013797
LGALS7B	0.173987798	0.000901279	0.002013797
UPF0639	-0.173940731	0.000904227	0.002020158
RXFP4	0.173925744	0.000905168	0.002022033
CPD	-0.173893185	0.000907215	0.002026378
TMEM72	-0.173877302	0.000908215	0.002028311
ZNF345	-0.173876205	0.000908284	0.002028311
FXR1	0.173849141	0.00090999	0.002031633
AGPAT2	0.173847804	0.000910075	0.002031633
NTN1	-0.173847759	0.000910078	0.002031633
SSX6	0.17383104	0.000911133	0.002033762

CCR7	-0.173823001	0.000911641	0.002034668
SLC4A8	-0.173805317	0.00091276	0.002036936
WDFY4	-0.173803043	0.000912904	0.002037029
IL12A	0.173783473	0.000914143	0.002039566
ALDH3A1	0.173764453	0.000915349	0.002042028
EP400NL	-0.173750712	0.000916222	0.002043643
LMNB1	0.173749813	0.000916279	0.002043643
IER3	0.173690846	0.000920031	0.002051782
IFNAR1	-0.173674811	0.000921053	0.002053723
CCDC141	-0.173673962	0.000921108	0.002053723
ADAM12	-0.173623242	0.00092435	0.002060721
C1orf104	-0.173616285	0.000924795	0.002061484
FMNL2	-0.173593914	0.000926229	0.002064385
OMP	-0.173592745	0.000926304	0.002064385
IGLL3	0.173576931	0.000927319	0.002066416
GALM	0.17356815	0.000927883	0.002067441
LOC100130	-0.173517422	0.000931148	0.002074483
GRPEL2	0.173479268	0.00093361	0.002079736
GNG2	-0.173460409	0.000934829	0.00208222
FUNDC1	0.17344475	0.000935843	0.002084244
C17orf99	0.173441591	0.000936047	0.002084467
CSRP2BP	0.17343852	0.000936246	0.002084677
PAQR6	0.173435551	0.000936439	0.002084872
FAM125B	-0.173425662	0.00093708	0.002086066
MAST1	0.173387139	0.000939581	0.002091399
C19orf35	-0.173384515	0.000939751	0.002091545
CES8	-0.173378541	0.00094014	0.002092176
ADAMTS17	-0.173374377	0.00094041	0.002092545
SRD5A3	0.173357913	0.000941482	0.002094695
ACTG2	0.173230929	0.000949785	0.002112931
MBNL3	-0.173216153	0.000950755	0.002114854
ZNF589	0.173206209	0.000951409	0.002116072
DHX30	0.173190556	0.000952438	0.002118125
SHH	-0.173174894	0.00095347	0.002120182
TCF7	0.173169972	0.000953794	0.002120666
ATOH8	-0.173156995	0.00095465	0.002122332
LONRF3	-0.173104682	0.000958106	0.002129647
MSGN1	0.173103959	0.000958154	0.002129647
C11orf52	0.17310055	0.000958379	0.00212991
GCH1	-0.173089932	0.000959083	0.002131235
GATAD1	-0.173071105	0.00096033	0.00213377
VRK3	0.17304064	0.000962353	0.002138025
ANKRD20A4	-0.173030846	0.000963004	0.002139233
CHRNA4	-0.173015232	0.000964043	0.002141301
OGFOD1	-0.173007175	0.000964579	0.002142254
MAP7D3	-0.172990782	0.000965671	0.00214444

ABCC6P2	0.172981742	0.000966274	0.00214554
ETHE1	0.172948391	0.000968501	0.002150244
DDC	0.17290462	0.00097143	0.002156307
SLC35E3	-0.172904359	0.000971448	0.002156307
C17orf47	-0.172887403	0.000972585	0.002158591
CCDC62	-0.17287721	0.000973269	0.002159868
LYG1	0.17287447	0.000973453	0.002160036
C5orf22	-0.172840883	0.000975711	0.002164805
PCGF3	-0.172791514	0.000979039	0.002171946
TRNP1	0.17276251	0.000980999	0.002176052
FAIM2	-0.172745891	0.000982124	0.002178304
FKBP1B	0.172734562	0.000982891	0.002179763
PHF17	-0.172725886	0.000983479	0.002180824
JAZF1	-0.172651196	0.000988554	0.002191835
KEAP1	0.172637282	0.000989503	0.002193693
DCT	-0.172616006	0.000990954	0.002196666
DKFZP586I1	-0.172568159	0.000994225	0.002203673
DGCR5	-0.172530182	0.000996829	0.002209198
POU5F1B	0.17251965	0.000997552	0.002210555
CDK15	-0.17250854	0.000998316	0.002212
KIAA0141	-0.172499008	0.000998971	0.002213206
SBDS	-0.172464884	0.001001321	0.002218164
SLC25A38	0.172461806	0.001001533	0.002218388
FOXE3	0.172452638	0.001002165	0.002219541
ADA	0.172449453	0.001002385	0.002219579
IRF7	0.17244916	0.001002405	0.002219579
SYS1-DBND	-0.172446635	0.001002579	0.002219718
CYB5R3	-0.172443955	0.001002764	0.002219763
MYLK2	0.17244311	0.001002822	0.002219763
ZC3H10	0.172378045	0.001007323	0.002229476
BGN	-0.172363249	0.001008349	0.002231499
CXCL14	-0.172350367	0.001009243	0.002233229
PRM1	0.17232855	0.001010758	0.002236335
GDPD1	-0.172304863	0.001012406	0.002239733
SLC20A2	-0.172243033	0.00101672	0.002249026
MS4A14	-0.172227345	0.001017817	0.002251203
IL28RA	-0.172219156	0.00101839	0.00225222
AFMID	0.17220656	0.001019273	0.002253921
TMEM116	0.172169059	0.001021903	0.002259487
CCR10	0.172138653	0.001024041	0.002263962
MSL1	-0.172131493	0.001024545	0.002264825
CBR3	0.172110415	0.001026029	0.002267855
LPAR6	-0.172090415	0.00102744	0.002270722
RHOXF2B	0.17207746	0.001028355	0.002272491
LOC440905	0.172060217	0.001029574	0.002274932
PLA2G16	0.172019152	0.001032481	0.002281104

CDA	-0.172001838	0.00103371	0.002283564
CORO1C	-0.171977039	0.001035471	0.002287202
BEND6	-0.171962114	0.001036533	0.002289292
DIMT1L	-0.171947136	0.001037599	0.002291393
ALDH1B1	-0.171933599	0.001038564	0.002293269
HIST1H2AL	0.171911395	0.001040148	0.002296512
EPHA8	0.171905614	0.00104056	0.002297169
RSPO1	-0.171900761	0.001040907	0.002297679
ARMCX1	-0.171892171	0.001041521	0.002298779
AGAP1	-0.171833958	0.001045689	0.002307724
ARHGAP1	-0.171825353	0.001046307	0.002308831
EIF1AX	-0.171818726	0.001046782	0.002309624
HSD3B2	-0.171808055	0.001047549	0.002311059
HEXIM1	-0.171792396	0.001048675	0.002313287
C1orf112	0.171784721	0.001049227	0.002314248
QPRT	0.171769796	0.001050301	0.002316362
GTSE1	0.171754923	0.001051373	0.002318469
FAM13AOS	-0.171740312	0.001052427	0.002320536
TMEM178	-0.171674709	0.001057171	0.002330738
LILRB5	-0.171651095	0.001058883	0.002334255
KIAA0895	-0.171644208	0.001059383	0.002335098
NRAP	-0.171641322	0.001059593	0.002335302
ACTN1	-0.171636423	0.001059948	0.002335827
MGEA5	-0.17160991	0.001061876	0.002339816
OR2B2	0.171531431	0.0010676	0.002352169
KRTAP19-1	0.171515079	0.001068796	0.002354544
GPR137B	-0.171506509	0.001069424	0.002355528
AKR1C4	0.171505748	0.001069479	0.002355528
TMEM71	-0.171485006	0.001071	0.002358615
ANGEL2	-0.171462814	0.001072628	0.00236194
FASTKD1	-0.171447511	0.001073753	0.002364155
CPZ	-0.171423834	0.001075494	0.002367728
RPS4Y2	0.171418439	0.001075891	0.002368341
ISLR	-0.171409944	0.001076517	0.002369456
ST3GAL2	-0.171407133	0.001076724	0.00236965
ATL3	-0.17140361	0.001076984	0.00236996
TOP2A	0.17138658	0.00107824	0.002372462
TYW1	-0.171298969	0.001084723	0.002386462
HNRNPUL2	-0.171285107	0.001085752	0.002388462
ENPP4	-0.17126739	0.001087068	0.002391094
C2orf16	-0.171250788	0.001088303	0.002393546
KCNQ5	-0.171243274	0.001088863	0.002394512
MME	-0.171231078	0.001089771	0.002396245
AQP5	0.171226974	0.001090077	0.002396653
KITLG	-0.171156823	0.001095317	0.002407909
PLEKHA8	0.171150954	0.001095757	0.002408609

TCEB3	-0.171148128	0.001095968	0.002408808
SCFD2	-0.171140033	0.001096575	0.002409875
XPNPEP1	0.171106433	0.001099096	0.002415149
FAM40B	-0.171100067	0.001099574	0.002415933
CCBP2	-0.171085597	0.001100662	0.002418056
PDP1	-0.171073321	0.001101585	0.002419818
CIR1	0.171066837	0.001102073	0.002420623
ZNF813	-0.171044883	0.001103727	0.002423989
C21orf33	0.171034216	0.001104532	0.002425488
TDRD7	-0.171031944	0.001104703	0.002425597
SLC22A6	0.170995128	0.001107485	0.002431436
PLK1S1	0.170949604	0.001110933	0.002438606
TP53TG3B	0.170948778	0.001110995	0.002438606
USHBP1	-0.170938483	0.001111777	0.002440052
FOXI2	-0.170920934	0.001113109	0.002442708
MBOAT2	-0.17087137	0.001116881	0.002450715
C19orf26	-0.17086255	0.001117554	0.002451921
IFNGR1	-0.170772607	0.001124433	0.002466741
FAM177A1	-0.170676953	0.001131791	0.002482611
LMNB2	0.170672651	0.001132123	0.002483065
SPINK1	0.170668496	0.001132444	0.002483495
SAG	0.170660434	0.001133066	0.002484587
ISG20	0.170584079	0.001138978	0.002497275
MAB21L1	-0.170580207	0.001139279	0.002497659
XPNPEP2	0.170576333	0.00113958	0.002498044
KATNA1	0.170562112	0.001140684	0.00250019
ZNF226	0.170538747	0.001142501	0.002503735
LOC401127	-0.170538086	0.001142553	0.002503735
SNN	-0.170526954	0.00114342	0.002505358
VPS54	-0.170508412	0.001144865	0.002508249
FRG1B	-0.170484526	0.001146729	0.002512056
TADA1	-0.17046339	0.00114838	0.002515398
SEC1	-0.170458702	0.001148747	0.002515924
MEIS2	-0.170455699	0.001148982	0.002516162
HIST1H2AG	-0.170384682	0.001154551	0.002528079
NAPA	0.17036167	0.00115636	0.002531763
ADIG	0.170348086	0.00115743	0.002533826
MYCNOS	0.170338531	0.001158183	0.002534977
KIAA1522	0.170338182	0.00115821	0.002534977
TAC3	0.170319881	0.001159653	0.002537857
PLIN3	0.170312702	0.00116022	0.002538818
MNDA	-0.17031078	0.001160372	0.002538871
RRAS	0.170273867	0.001163289	0.002544975
PRAME	0.170238693	0.001166076	0.002550791
RNF167	0.170219774	0.001167577	0.002553795
HCG9	0.170216884	0.001167807	0.002553992

CCL3	0.17021541	0.001167924	0.002553992
VMAC	0.170193865	0.001169636	0.002557455
DLEU7	-0.170191544	0.001169821	0.002557578
C2orf40	-0.170183176	0.001170487	0.002558753
RPS6KA6	-0.170178515	0.001170858	0.002559283
PFKFB2	-0.170120887	0.001175453	0.002569047
PIP5K1C	-0.170107185	0.001176549	0.002571158
PAK3	-0.170099193	0.001177188	0.002572273
IL12B	-0.170054882	0.001180738	0.002579747
CATSPER2	0.170046851	0.001181382	0.002580872
FAM120C	-0.170044672	0.001181557	0.002580971
SLC19A2	-0.170042049	0.001181768	0.002581148
GBA	0.170012863	0.001184113	0.002585987
SPTLC1	-0.169988717	0.001186057	0.002589948
KIAA1875	0.16992268	0.001191388	0.002601303
CKAP5	-0.169884621	0.00119447	0.002607747
NBN	-0.169880945	0.001194768	0.002608112
IL16	-0.169877954	0.001195011	0.002608356
SPAG6	0.169813881	0.001200218	0.002619028
C1orf161	-0.169813617	0.00120024	0.002619028
C5orf13	0.169812945	0.001200295	0.002619028
TAP1	0.16980766	0.001200725	0.00261968
DRD4	0.169802066	0.001201181	0.002620388
AP1M1	0.169746522	0.001205716	0.002629992
ASCC2	0.169741155	0.001206155	0.002630662
MIPEP	-0.169695651	0.001209883	0.002638504
DEPDC1	0.169681329	0.001211059	0.002640779
CNR2	-0.169676776	0.001211432	0.002641305
PTP4A3	0.169672952	0.001211747	0.002641701
ZNF189	-0.169658939	0.001212898	0.002643922
LAP3	-0.169642792	0.001214227	0.002646528
C12orf53	-0.169638053	0.001214617	0.002647089
SH3D20	-0.169615248	0.001216496	0.002650894
AIMP2	0.169561945	0.001220899	0.002660197
GPR146	-0.169549987	0.001221888	0.002662062
C10orf26	-0.169514505	0.001224829	0.002668177
KRT78	0.169454092	0.001229852	0.002678825
HSPA1L	0.169440873	0.001230953	0.002680931
ACER1	-0.169382046	0.001235866	0.002691336
RFX5	-0.169332292	0.001240035	0.00270012
PPIL5	0.16931606	0.001241398	0.002702792
STON1-GTF	-0.169302616	0.001242528	0.002704956
LZTFL1	-0.169288043	0.001243754	0.002707329
ZNF675	-0.169250524	0.001246915	0.002713913
LOC440925	0.169200476	0.001251143	0.002722819
C12orf42	0.169198217	0.001251334	0.002722937

GALNTL4	-0.169185755	0.001252389	0.002724935
C1orf177	0.169159439	0.00125462	0.00272949
ANKRD34C	-0.169155893	0.001254921	0.002729672
C4BPB	0.169155224	0.001254977	0.002729672
C19orf21	0.169151574	0.001255287	0.002730047
TMEM108	-0.169145267	0.001255823	0.002730914
RPL10L	0.169115417	0.001258359	0.002736131
ARNTL2	-0.169051838	0.001263778	0.002747614
AMZ2P1	0.169023983	0.001266159	0.00275249
DECR2	0.169019307	0.001266559	0.002753059
SSX4	0.169004528	0.001267825	0.002755509
CNGB3	0.168947911	0.001272682	0.002765765
CCL24	-0.168925514	0.001274609	0.002769649
BTBD18	-0.168903432	0.001276511	0.002773479
DUSP15	0.168883249	0.001278251	0.002776958
C1orf49	0.16887432	0.001279022	0.00277833
GAGE2A	0.16885237	0.001280919	0.002782146
FANCA	0.168823463	0.001283421	0.002787276
UNC45A	0.16879384	0.001285989	0.002792549
LOC442421	-0.168745105	0.001290224	0.002801441
PADI2	-0.168675074	0.001296333	0.002814398
KLK11	-0.168667648	0.001296983	0.002815501
FUT4	-0.168604272	0.001302536	0.002827249
CLIP3	-0.168589828	0.001303805	0.002829695
PICK1	0.168586708	0.001304079	0.002829982
ATP1B2	-0.16855729	0.001306668	0.00283529
MYO16	-0.168535218	0.001308613	0.002839201
SLC35B4	-0.168512429	0.001310624	0.002843254
ANXA2P3	0.168487893	0.001312792	0.002847587
LPL	-0.168486594	0.001312907	0.002847587
CLIC6	-0.168464445	0.001314867	0.002851528
BLOC1S2	0.168408369	0.001319842	0.002862006
RTN3	0.168396355	0.001320911	0.00286401
VPS53	-0.168368159	0.001323421	0.00286914
VCY	0.168365192	0.001323685	0.002869401
FAM55C	-0.168222611	0.001336447	0.002896751
LAMA3	-0.168191348	0.00133926	0.002902533
TUBA3C	0.168169987	0.001341186	0.002906389
RPGRIP1L	-0.168103164	0.001347225	0.00291916
CH25H	-0.168070586	0.001350179	0.002925241
GIPC3	-0.168060692	0.001351077	0.002926869
LOC100129	-0.168043708	0.00135262	0.002929893
LOC646627	0.168031184	0.001353759	0.002931892
RAB28	-0.168030322	0.001353838	0.002931892
RNF183	0.168016837	0.001355065	0.002934231
HIST1H2BG	0.168008501	0.001355824	0.002935555

MR1	-0.168000266	0.001356575	0.002936861
JMJD8	0.167981237	0.00135831	0.002939986
MEIG1	0.167981197	0.001358314	0.002939986
CDRT1	0.167939216	0.00136215	0.002947969
TMEM70	0.167932674	0.001362748	0.002948944
LOC100303	-0.167929244	0.001363062	0.002949303
MDM4	-0.167865739	0.001368887	0.002961379
CYP2A6	-0.167865155	0.001368941	0.002961379
TNFSF11	-0.167847985	0.00137052	0.002964473
RNF14	-0.16783691	0.001371539	0.002966356
REG1A	0.167791017	0.00137577	0.002975184
LOC100129	-0.16778763	0.001376083	0.002975402
FAM19A1	-0.16778669	0.00137617	0.002975402
SMOX	0.167755403	0.001379062	0.002981331
RAB1B	0.167703891	0.001383837	0.002991328
FXYD3	0.167698222	0.001384363	0.002991971
APOE	0.167697452	0.001384434	0.002991971
TBC1D3H	-0.167627525	0.001390942	0.003005709
EIF1B	0.167593223	0.001394145	0.003012302
TSGA10IP	0.167575603	0.001395792	0.003015455
CD163	-0.167574379	0.001395907	0.003015455
CCDC155	0.167566539	0.001396641	0.003016713
AKNAD1	0.167564823	0.001396801	0.003016733
LOC121838	-0.167533839	0.001399705	0.003022676
CHID1	0.167529215	0.001400139	0.003023278
GIP	0.167527633	0.001400287	0.003023278
HEXB	0.167520875	0.001400921	0.00302432
REXO1	-0.167507186	0.001402207	0.003026767
C11orf20	0.167503873	0.001402518	0.003027111
MED18	0.167486656	0.001404137	0.003030277
ZNF782	-0.167474721	0.001405261	0.003032372
FAM69B	0.167464866	0.001406189	0.003034046
NRL	0.167460096	0.001406638	0.003034687
ZNF831	-0.167455426	0.001407078	0.003035308
FLVCR1	0.167423045	0.001410133	0.003041569
RHOD	0.167392297	0.00141304	0.003047508
PTPRCAP	0.167350337	0.001417016	0.003055752
LOC644538	-0.167329549	0.001418989	0.003059676
NEGR1	-0.16728688	0.001423047	0.003068095
GRIA3	-0.167283267	0.001423392	0.003068505
HERPUD1	-0.167261	0.001425514	0.003072748
SH2D4A	-0.167243162	0.001427217	0.003076085
CHIC1	-0.167237445	0.001427763	0.003076929
E2F4	0.167228256	0.001428641	0.003078488
CARNS1	-0.167221579	0.001429279	0.00307953
MBD4	-0.167218389	0.001429584	0.003079854

GABARAPL2	0.167162384	0.00143495	0.003091079
C17orf76	-0.167155552	0.001435606	0.003092157
MTRF1	-0.167143587	0.001436755	0.003094298
LGI1	-0.167129211	0.001438137	0.003096939
ADD3	-0.16712109	0.001438918	0.003098286
C1orf198	-0.167074886	0.00144337	0.003107536
SAMSN1	-0.16706718	0.001444113	0.003108801
FOXJ1	0.167050817	0.001445694	0.003111867
TRIM42	0.167047449	0.001446019	0.003112231
NARS	-0.167027216	0.001447976	0.003116106
PREP	-0.166987504	0.001451824	0.003123755
SQLE	0.166987294	0.001451844	0.003123755
ADCK1	0.16694107	0.001456334	0.003133078
HOXD10	0.166838615	0.001466333	0.003154247
HELQ	-0.166832673	0.001466915	0.003155158
PSMD1	0.16681061	0.001469077	0.003159467
IL17RB	0.166790872	0.001471013	0.003163284
LPAR5	-0.166789283	0.001471169	0.003163284
B2M	-0.166784626	0.001471627	0.003163926
CFI	-0.166781311	0.001471953	0.003164285
TIGD6	0.166756946	0.001474348	0.003169092
ZNF77	-0.166747911	0.001475237	0.003170661
RAI14	-0.166734389	0.001476569	0.003173181
FOXN4	-0.166727296	0.001477268	0.00317434
AGAP3	0.166712033	0.001478773	0.003177232
SEC14L2	-0.166658568	0.001484056	0.003188239
ASNS	0.166636073	0.001486284	0.003192682
CALCOCO2	-0.166624159	0.001487466	0.003194875
ICAM3	0.166598775	0.001489985	0.003199941
LOC153910	0.166542534	0.001495582	0.003211615
RGAG1	-0.166533772	0.001496456	0.003213145
SMPD4	0.166512007	0.001498628	0.003217462
AMN1	-0.166472919	0.001502537	0.003225506
CYCSP52	-0.166439259	0.00150591	0.003232398
STK32C	0.166427269	0.001507113	0.003234632
RNASE4	-0.166414689	0.001508376	0.003236995
FAM134A	0.166391436	0.001510714	0.003241663
METAP1	-0.166379559	0.00151191	0.003243878
C12orf71	-0.166326819	0.001517228	0.003254939
SNORA45	0.166310377	0.00151889	0.003258153
CRYGC	0.166307594	0.001519171	0.003258405
HLA-H	0.166250299	0.001524975	0.003270502
FGFR2	-0.166236124	0.001526414	0.003273236
CDH6	-0.166209609	0.00152911	0.003278329
PRPF4B	-0.166209522	0.001529119	0.003278329
LONRF2	-0.166117957	0.00153846	0.003298001

CUX1	-0.166103221	0.001539968	0.003300879
TNFSF9	0.166096961	0.001540609	0.003301898
ESRRG	-0.166082096	0.001542133	0.003304807
TASP1	-0.166047387	0.001545695	0.003312085
SP100	-0.166034748	0.001546994	0.003314512
ZNF571	-0.166027557	0.001547734	0.00331574
NUDT18	0.166005121	0.001550043	0.003320179
ZNF221	-0.16600419	0.001550139	0.003320179
C9orf109	0.165980413	0.00155259	0.003325071
SEPT7P2	-0.165977189	0.001552923	0.003325426
VIPR2	-0.165947224	0.001556018	0.003331695
NKAIN2	0.165927482	0.00155806	0.003335709
GGCX	-0.165913498	0.001559508	0.00333845
ZBTB25	-0.165845708	0.001566545	0.003353153
C15orf34	-0.165832894	0.001567878	0.003355646
SAPS1	0.165792498	0.001572088	0.003364296
BBS12	-0.165754713	0.001576036	0.003372381
PRKCG	0.165725306	0.001579114	0.003378606
PRR22	0.165710215	0.001580696	0.003381627
SET	0.165707873	0.001580942	0.003381789
OR5AK2	-0.165704511	0.001581295	0.00338218
NINL	-0.165699476	0.001581823	0.003382947
C17orf91	-0.165673541	0.001584547	0.003388408
TCERG1	-0.165669306	0.001584992	0.003388997
SDR42E1	-0.165652333	0.001586777	0.00339245
FABP6	0.165626087	0.001589542	0.003397996
SCGN	0.165608654	0.001591381	0.003401561
C3orf58	-0.165598091	0.001592496	0.003403579
LOC643719	0.165580696	0.001594333	0.003407141
ZNF697	-0.165536387	0.001599023	0.003416797
ASGR1	0.16550107	0.001602771	0.003424437
RAB8A	-0.16548327	0.001604662	0.003428111
PAIP2B	-0.165480511	0.001604956	0.00342837
CDK20	-0.16547616	0.001605419	0.003428991
CACNG4	0.165404413	0.001613068	0.003444959
PHF23	0.165377669	0.001615927	0.003450696
ZNF594	-0.165355277	0.001618325	0.003455446
STK10	-0.165312869	0.001622876	0.003464791
RAB5C	-0.16529567	0.001624725	0.003468366
LTB	0.165276066	0.001626834	0.003472498
IQCF1	0.165235986	0.001631155	0.003481348
POM121L4P	-0.165222393	0.001632623	0.003484107
CNGA4	-0.16521452	0.001633474	0.003485549
FAM153B	-0.165180899	0.001637111	0.003492937
LOXL3	-0.165165894	0.001638737	0.003496031
RGS9BP	0.165162068	0.001639152	0.003496362

MBD6	-0.165161224	0.001639244	0.003496362
SPPL2A	-0.165127127	0.001642945	0.003503881
RIBC1	0.16512398	0.001643287	0.003504235
EDN1	-0.165112659	0.001644517	0.003506484
PLA2G5	-0.16509417	0.001646529	0.003510398
ZSWIM5	0.165078256	0.001648263	0.003513718
MAGEA3	0.165053922	0.001650916	0.003518998
JUP	0.165035493	0.001652929	0.003522911
PCDHA11	-0.165024455	0.001654135	0.003525105
C17orf75	0.164989436	0.001657968	0.003532895
SETD8	0.16498009	0.001658992	0.003534354
SMAGP	0.164979949	0.001659008	0.003534354
LTB4R	-0.164975639	0.00165948	0.003534983
PRKX	-0.164946593	0.001662668	0.003541395
NR6A1	-0.164906422	0.001667086	0.003550425
CDC14A	-0.164839751	0.001674443	0.003565711
ZNF235	-0.164820051	0.001676622	0.003569971
GPA33	-0.164814892	0.001677194	0.003570805
KDM5B	-0.164795716	0.001679318	0.003574946
ELMOD1	-0.164786933	0.001680292	0.003576637
ANXA9	0.164759328	0.001683357	0.003582693
WDR5	0.164758066	0.001683497	0.003582693
SEMA3D	-0.164754144	0.001683933	0.003583237
CAMK2G	-0.164709557	0.001688895	0.003593412
USP3	-0.164701944	0.001689743	0.003594833
C16orf88	0.164680646	0.001692119	0.003599503
LOC647859	-0.164672029	0.001693081	0.003601166
VEZF1	-0.164658349	0.00169461	0.003604032
TACSTD2	-0.164636732	0.001697028	0.003608789
PRM2	0.164601425	0.001700984	0.003616816
XRCC6BP1	0.164580566	0.001703325	0.003621407
SPAST	-0.164561825	0.001705431	0.003625497
SLC4A1	-0.164557105	0.001705962	0.003626239
ANKRD20A3	-0.164515113	0.001710691	0.003635535
ZW10	-0.164515024	0.001710701	0.003635535
LGALS1	0.164452129	0.001717806	0.003650246
TBC1D1	-0.164444265	0.001718696	0.003651748
BATF	0.164436923	0.001719528	0.003653125
ZNF766	0.164405954	0.001723039	0.003660195
VMO1	0.164396607	0.0017241	0.003661978
HRH3	0.164395321	0.001724246	0.003661978
NPFFR2	0.164387294	0.001725158	0.003663524
TLX1	0.164380565	0.001725923	0.003664757
DSG2	-0.164356848	0.001728621	0.003670095
F2RL3	-0.164300367	0.001735061	0.003683376
CCDC153	0.164274466	0.001738022	0.003689268

SOCS7	-0.16426177	0.001739475	0.003691959
TRPC2	0.164245126	0.001741382	0.003695612
ABCC11	-0.164205218	0.001745961	0.003704935
SETMAR	0.164136263	0.001753899	0.003721383
C10orf72	-0.164119112	0.001755879	0.003725186
TTC36	-0.164116946	0.001756129	0.00372532
KLHL18	-0.164100688	0.001758007	0.003728908
F11R	-0.164085896	0.001759718	0.003732139
LOC340508	0.1640531	0.001763517	0.003739797
GCNT2	0.164041276	0.001764888	0.003742307
ROBO3	0.16402395	0.001766899	0.003746173
NAA40	-0.163978689	0.001772163	0.003756933
ATG9B	0.163931816	0.001777629	0.00376812
IFIT1B	-0.163910044	0.001780174	0.003773112
NME5	-0.163904418	0.001780832	0.003774105
IGF2BP3	0.163885717	0.001783021	0.003778342
CCDC147	-0.163882701	0.001783374	0.003778688
NRXN3	-0.163867954	0.001785102	0.003781948
LRMP	-0.163855502	0.001786562	0.003784639
AP1S2	-0.163851685	0.00178701	0.003785185
ACADL	-0.163831791	0.001789346	0.00378973
GPR27	-0.163816095	0.001791191	0.003792859
HSPB7	-0.163815982	0.001791205	0.003792859
RNF186	0.163769437	0.001796686	0.003804062
MYOM3	-0.163754335	0.001798468	0.003807429
PRSS30P	0.163737399	0.001800468	0.003811258
PDIA3P	-0.163733204	0.001800964	0.003811903
SERPINB8	-0.16373026	0.001801312	0.003812234
SCARF2	-0.163657479	0.001809934	0.003830075
PRO0611	-0.163653889	0.00181036	0.00383057
DLGAP3	0.163609126	0.001815683	0.003841425
SPSB3	0.163604306	0.001816257	0.003842231
CTLA4	0.16355512	0.001822124	0.003854233
SCT	0.163553475	0.001822321	0.003854239
BEND4	-0.163512799	0.001827186	0.00386412
SLC13A1	-0.163496667	0.001829119	0.003867797
NR5A1	0.163467959	0.001832564	0.003874669
TLR10	-0.163457731	0.001833792	0.003876855
ABHD12B	-0.163455007	0.00183412	0.003877135
DIAPH3	0.16344543	0.001835271	0.003879158
PSD	-0.163436922	0.001836294	0.003880909
CUL1	-0.163431301	0.001836971	0.003881926
C15orf29	-0.163386193	0.001842407	0.003893001
HBQ1	0.163360095	0.001845559	0.003899247
FGFR3	0.16333458	0.001848645	0.003905353
PINX1	0.163323572	0.001849978	0.003907754

TMED6	0.163321737	0.0018502	0.003907809
PIAS4	-0.163301062	0.001852706	0.003912688
UTP15	-0.163283971	0.00185478	0.003916652
GLIPR1	-0.16327583	0.001855769	0.003918325
ZNF10	-0.163267887	0.001856734	0.003919947
KRTAP26-1	0.16322657	0.001861762	0.003930145
SFT2D3	0.163223922	0.001862084	0.003930409
MEN1	0.163221943	0.001862326	0.003930502
AMPD3	-0.163152072	0.001870859	0.003948092
C7orf28A	0.163149124	0.001871219	0.003948209
CCDC150	0.16314838	0.00187131	0.003948209
C2orf61	-0.163071042	0.001880799	0.003967808
DDX1	0.163060268	0.001882124	0.003970184
PMS2L11	-0.163050239	0.001883359	0.003972367
MCART1	-0.163027528	0.001886157	0.003977848
KCTD10	-0.162998595	0.001889727	0.003984956
SFRP2	-0.162980337	0.001891984	0.003989291
SEC23B	-0.162961594	0.001894302	0.003993757
MTHFSD	0.162958689	0.001894662	0.003994092
AP1S3	-0.162881683	0.001904218	0.004013812
TBXA2R	-0.162865454	0.001906237	0.004017527
RFPL4B	0.162863374	0.001906496	0.004017527
WDR33	0.162862654	0.001906586	0.004017527
CCDC28B	0.162848661	0.001908329	0.004020775
STARD10	0.16283658	0.001909835	0.004023522
TPRG1	-0.16282231	0.001911615	0.004026847
SRGN	-0.162815651	0.001912446	0.004028172
C19orf34	-0.162812293	0.001912866	0.004028629
NOTCH2NL	0.162763492	0.001918969	0.004041057
SCN2B	-0.162751632	0.001920455	0.004043759
PAGE2	0.162737654	0.001922208	0.004047021
PROX2	-0.162708187	0.001925908	0.004054382
SYT3	-0.162694028	0.001927688	0.004057701
PABPC4	0.162620972	0.001936897	0.004076654
RGR	0.162614584	0.001937704	0.004077922
PLA2G4E	-0.162581241	0.001941922	0.004086366
MYBPC1	0.162550277	0.001945846	0.004094192
PROL1	-0.162537394	0.001947481	0.004097199
SFT2D1	0.162517176	0.001950049	0.004102169
MACC1	-0.162496015	0.001952741	0.004107397
SDR39U1	0.162484679	0.001954184	0.004109998
SYBU	-0.162456756	0.001957743	0.004117049
PRKAG1	0.162445601	0.001959166	0.004119607
XG	-0.162443323	0.001959457	0.004119784
IL32	0.162408841	0.001963864	0.004128613
LBXCOR1	-0.162366543	0.001969282	0.004139567

SIRT5	0.162331354	0.0019738	0.004148626
INHBE	-0.162329617	0.001974023	0.004148657
NUP205	-0.162286829	0.00197953	0.004159791
DVL2	0.162241625	0.001985363	0.004171609
VSTM2B	0.162231803	0.001986633	0.004173836
CRTAP	0.16216502	0.001995284	0.004191569
CENPN	0.162156329	0.001996412	0.004193498
SCARNA17	0.162028665	0.002013055	0.00422801
PHGDH	0.162022858	0.002013815	0.00422916
ZBTB2	-0.161953995	0.002022848	0.004247586
DNAJC11	-0.161952717	0.002023016	0.004247586
KLHDC9	0.161887618	0.002031589	0.004265138
PTPRR	-0.161885045	0.002031929	0.004265401
EIF2B2	0.161845482	0.002037156	0.004275924
WNK4	0.161842245	0.002037585	0.004276372
ELOVL6	-0.161821962	0.00204027	0.004281223
SLC27A2	-0.161821542	0.002040326	0.004281223
GTSF1	0.16176756	0.002047488	0.0042958
C1orf220	-0.161708894	0.002055299	0.004311732
SH2D2A	0.161706491	0.002055619	0.00431195
CDH18	0.161682823	0.002058778	0.004318122
FBXO24	0.161670396	0.002060439	0.00432115
SPANXE	0.161656615	0.002062282	0.004324559
NPPB	0.16163255	0.002065503	0.004330859
ARRDC1	0.161608538	0.002068722	0.004337152
HADH	-0.16160096	0.002069739	0.004338827
RHPN1	0.161583782	0.002072046	0.004343206
MGC3771	0.161573093	0.002073483	0.00434576
FAM111A	-0.1615379	0.002078219	0.004355228
CHP	-0.161518707	0.002080806	0.004360191
ZNF322B	0.161500104	0.002083317	0.004364992
WDR67	0.161495217	0.002083977	0.004365916
WDR73	0.161444508	0.002090836	0.004379825
ABTB1	0.161432569	0.002092454	0.004382753
HNRNPF	-0.16141597	0.002094705	0.004387007
TBC1D22B	0.161410307	0.002095474	0.004388155
BEAN	-0.161392686	0.002097867	0.004392705
HTATIP2	0.161380409	0.002099536	0.004395737
ZNF829	-0.161355377	0.002102942	0.004402406
MYO15A	-0.161336969	0.00210545	0.004407194
RTN1	-0.16129623	0.002111011	0.00441837
NAT15	0.161260792	0.002115859	0.004428051
CD96	-0.161254921	0.002116663	0.004429269
KSR1	-0.161248116	0.002117596	0.004430602
RABL3	-0.161247025	0.002117745	0.004430602
TRIM38	-0.161222254	0.002121143	0.004437244

C19orf69	0.161178098	0.002127212	0.004449472
ZNF498	-0.161156907	0.00213013	0.004455108
DNAJC19	0.161127808	0.002134143	0.004463032
TCIRG1	0.161086279	0.002139882	0.004474565
C17orf60	0.161022109	0.002148778	0.004492695
B3GAT1	-0.161018637	0.00214926	0.00449281
ZNF323	0.161018463	0.002149285	0.00449281
COPS7A	0.161006298	0.002150975	0.004495872
RBP7	0.160980415	0.002154576	0.004502926
C7orf29	0.16097066	0.002155935	0.004505293
PPP2R3C	0.160924332	0.002162398	0.004518325
ADRA2B	-0.1609131	0.002163967	0.00452113
C13orf23	-0.160856313	0.002171919	0.004537198
TMEM62	0.160854924	0.002172114	0.004537198
RAB11FIP5	-0.160848663	0.002172992	0.004538557
HAUS4	0.160836599	0.002174686	0.004541617
OAF	-0.160820099	0.002177004	0.004545982
EXD3	0.160800472	0.002179764	0.004551268
TTC13	0.160788369	0.002181468	0.004554348
PIWIL4	-0.160784745	0.002181978	0.004554936
PCDH9	-0.160775753	0.002183245	0.004557103
RPL39L	0.160758003	0.002185748	0.004561849
TMUB2	0.160754558	0.002186234	0.004562385
HIST1H2AC	0.160735962	0.002188859	0.004567386
GTF2B	0.160715091	0.002191809	0.004572275
ZP1	0.160714926	0.002191832	0.004572275
CYP21A2	0.160714509	0.002191891	0.004572275
C1QTNF1	-0.160712887	0.002192121	0.004572275
HEYL	-0.160569925	0.002212428	0.004614148
C9orf156	0.1605538	0.002214729	0.004618464
ABCA4	0.160551856	0.002215007	0.004618559
CMTM8	0.160518922	0.002219714	0.00462789
RMND1	0.160502132	0.002222117	0.004632345
AGPAT5	-0.160500743	0.002222316	0.004632345
ISOC1	-0.160494901	0.002223153	0.004633605
CCDC41	-0.160471756	0.002226471	0.004640035
MRPL50	0.160446119	0.002230152	0.00464722
MAGEE1	-0.160417381	0.002234285	0.004655345
NIF3L1	0.160369491	0.002241188	0.004669239
RNF39	-0.160360344	0.002242508	0.004671501
TCF3	0.16033095	0.002246757	0.004679862
TRAT1	-0.16032306	0.002247898	0.00468175
ZC3H18	-0.16031836	0.002248579	0.004682677
ANKRD36	-0.160308254	0.002250042	0.004685235
CR1	-0.160303683	0.002250704	0.004686124
DIS3L2	0.160288387	0.002252922	0.00469025

IL17B	0.160285269	0.002253374	0.004690701
PI16	-0.160253349	0.002258008	0.004699857
IL20	-0.160244841	0.002259245	0.00470194
MEFV	-0.160216243	0.002263406	0.004709686
PKP1	-0.160216015	0.00226344	0.004709686
PLIN4	-0.160210248	0.00226428	0.004710941
GPR89A	0.160201293	0.002265585	0.004713164
VWA5B2	0.16016984	0.002270174	0.004721435
DNASE1	0.160169372	0.002270242	0.004721435
C1QA	0.160169168	0.002270272	0.004721435
SEC22C	-0.160142884	0.002274113	0.004728931
POLR1E	0.160075459	0.002283995	0.004748983
UGT1A1	-0.160060948	0.002286127	0.00475292
SORCS2	-0.16004718	0.002288151	0.004756632
C6orf25	-0.160027058	0.002291113	0.004762291
C14orf180	-0.160024218	0.002291531	0.004762663
LRRC39	-0.1600107	0.002293523	0.004766306
EMR3	-0.159999533	0.00229517	0.00476923
LRRN4CL	-0.159993086	0.002296121	0.004770709
SLC27A1	-0.159990142	0.002296555	0.004771113
FUCA1	-0.159981614	0.002297814	0.004773231
CD151	0.15997869	0.002298246	0.00477363
SCRN1	-0.159964557	0.002300334	0.004777469
BMP3	-0.159947545	0.00230285	0.004782195
PPP4R1L	-0.159937593	0.002304322	0.004784754
POLR3GL	0.159917269	0.002307333	0.004790071
NUP210L	-0.159917059	0.002307364	0.004790071
NCOA3	-0.159871995	0.002314052	0.004803455
GBP7	-0.159863775	0.002315274	0.004805491
C7orf42	-0.159841127	0.002318644	0.004811983
CHST2	-0.159799026	0.002324919	0.004824504
MCART6	-0.159796118	0.002325353	0.004824902
MVK	0.159793068	0.002325809	0.004825344
MKX	-0.159722145	0.002336419	0.004846853
SNX16	-0.159709158	0.002338367	0.004850388
SFI1	0.159676935	0.002343206	0.00485992
C14orf162	-0.159664243	0.002345115	0.004863109
ERI2	-0.159663461	0.002345232	0.004863109
PPFIA3	0.159638659	0.002348966	0.004870344
PKIB	0.159588198	0.002356579	0.004885621
MSL3L2	0.159478016	0.002373281	0.004919735
CELSR1	-0.159459662	0.002376074	0.004925012
PGBD2	-0.159457414	0.002376416	0.004925209
SNORA39	-0.15944523	0.002378272	0.004928542
FAM48B1	-0.159442777	0.002378646	0.004928803
C14orf70	0.15942346	0.002381591	0.004934393

ALG10	-0.159370658	0.002389659	0.004950594
KIAA1045	-0.159360932	0.002391148	0.004953027
DIAPH1	-0.159359735	0.002391331	0.004953027
PPIAL4E	0.159331676	0.002395631	0.004961418
CHTF8	-0.159317067	0.002397873	0.004965544
GRIP1	-0.159209123	0.002414496	0.004999126
RDH13	0.159208502	0.002414591	0.004999126
SMC2	-0.159138034	0.0024255	0.005021189
F9	-0.15912681	0.002427242	0.005024272
44083	0.159124761	0.00242756	0.005024408
HSPB6	-0.159099891	0.002431424	0.005031882
MPPED2	-0.159090477	0.002432887	0.005034388
PRDM4	-0.159082342	0.002434153	0.005036484
TNFRSF11B	-0.159052011	0.002438878	0.005045735
CYB5RL	-0.159046699	0.002439706	0.005046924
DKFZp761E	-0.159029859	0.002442333	0.005051835
CYP3A4	-0.159014853	0.002444677	0.005056157
IFI44L	-0.159005682	0.00244611	0.005058596
NCRNA0018	-0.158967223	0.002452129	0.005070517
ZCWPW1	0.158964556	0.002452547	0.005070854
SLC30A8	0.158945544	0.002455527	0.00507649
LPPR4	-0.158938843	0.002456579	0.005078137
C16orf11	0.158897558	0.002463066	0.005091017
NDRG4	-0.158877	0.002466301	0.005097177
MGC42105	-0.158853605	0.002469989	0.005104267
EPB42	-0.158841966	0.002471825	0.005107532
CDK16	0.158835865	0.002472788	0.005108992
TCTEX1D1	-0.158822891	0.002474837	0.005112695
GALNTL6	-0.158805604	0.00247757	0.005117809
ST5	-0.158803079	0.002477969	0.005118103
NFIL3	-0.158763194	0.002484285	0.005130617
GPR128	-0.158745859	0.002487035	0.005135763
BET3L	-0.158742752	0.002487528	0.005136249
PWP2	0.158723358	0.002490608	0.005142076
PABPC1L	0.158711062	0.002492563	0.005145578
PALMD	-0.158695906	0.002494974	0.005150021
C20orf108	-0.158685685	0.002496601	0.005152846
GPR77	-0.158661212	0.002500502	0.005160362
OSTBETA	0.158653164	0.002501785	0.005162476
CLEC17A	-0.158624644	0.00250634	0.005171339
FCGR2B	-0.158600092	0.002510267	0.005178904
PRKG2	-0.158588646	0.002512099	0.005182148
RHOA	0.15858608	0.00251251	0.005182459
ARGFXP2	-0.158577263	0.002513923	0.005184836
WDR52	-0.158555745	0.002517374	0.005191415
AKAP5	-0.158546216	0.002518903	0.005194032

OR8B12	0.158525293	0.002522265	0.005200424
ZNF410	-0.15852273	0.002522677	0.005200735
OLAH	-0.158517906	0.002523452	0.005201796
SCARNA5	0.158515345	0.002523864	0.005202107
CSK	0.158503148	0.002525827	0.005205613
REG3A	0.158479807	0.002529586	0.005212822
LOC389791	0.158476444	0.002530128	0.005213062
CETN1	0.158475836	0.002530226	0.005213062
TNRC6C	-0.158469532	0.002531243	0.005214617
GPR112	-0.158462079	0.002532445	0.005216554
SCPEP1	-0.158390126	0.002544078	0.005239975
ARHGAP26	-0.158348894	0.002550766	0.005253207
DSCR8	0.158329588	0.002553903	0.005259124
TMEM45A	-0.158322952	0.002554983	0.005260802
LOC646999	-0.158301379	0.002558494	0.005267487
ZNF702P	-0.158294686	0.002559584	0.005269187
BTN1A1	0.15829082	0.002560214	0.005269939
CARTPT	0.158287375	0.002560775	0.005270549
LOC100132	-0.158283731	0.002561369	0.005271227
CENPJ	0.158242247	0.00256814	0.005284615
FCER1G	0.158232026	0.00256981	0.005287507
GLCE	-0.158212357	0.002573028	0.005293581
CELSR3	0.158197103	0.002575527	0.005298173
CSNK1D	0.158151094	0.002583074	0.005313151
LOC441089	-0.158114288	0.002589127	0.005325051
PTPLAD1	-0.158107524	0.002590241	0.005326792
MXRA8	-0.158077668	0.002595162	0.005336361
EPT1	-0.158036069	0.002602033	0.005349938
NME7	0.158024677	0.002603918	0.00535326
SSBP2	-0.15798372	0.002610704	0.005366438
TSN	0.157982735	0.002610867	0.005366438
BASP1	-0.157962475	0.00261423	0.005372796
RWDD3	0.157935704	0.002618679	0.005381385
KRTAP20-4	0.157894418	0.002625555	0.005394957
C20orf70	0.157885885	0.002626978	0.005397324
FAM169B	-0.157852602	0.002632535	0.005408185
NKG7	0.157830747	0.002636191	0.005415135
POLE3	0.157799931	0.002641352	0.005425178
TMEM165	-0.1577918	0.002642716	0.005427419
AES	0.157778246	0.00264499	0.005431529
HIRIP3	0.157775826	0.002645396	0.005431804
C6orf154	0.157768481	0.002646629	0.005433776
RUNX1	-0.157751133	0.002649544	0.0054392
PXDNL	-0.15767849	0.002661783	0.005463761
SUV39H2	0.157641104	0.002668101	0.005476166
ABI2	-0.157616054	0.002672343	0.005484306

JPH4	-0.157575085	0.002679293	0.005498002
LGALS7	0.157490259	0.002693734	0.005526962
DACT3	-0.157488936	0.00269396	0.005526962
OR2W3	-0.157482681	0.002695028	0.005528583
CRYGA	0.157474177	0.00269648	0.005530992
HLA-DMA	0.15747101	0.002697021	0.005531533
PLA2G2E	0.15743474	0.002703225	0.005543685
ALK	-0.157423439	0.00270516	0.005547083
LMOD2	-0.157417591	0.002706163	0.005548567
LGALS13	0.157407456	0.0027079	0.005551558
PTPRD	-0.157379911	0.002712627	0.005560677
SULT6B1	0.157368588	0.002714573	0.005564092
SLC26A10	-0.157344467	0.002718722	0.005572022
PPP2R5C	-0.157335805	0.002720213	0.005574505
ATP6V1H	0.157331898	0.002720886	0.00557531
MED25	0.157324904	0.00272209	0.005577205
LOC348840	-0.157312285	0.002724265	0.005581087
SERBP1	0.157236537	0.002737355	0.005607326
FAM178A	-0.157221789	0.00273991	0.005611983
LOC642826	-0.157217304	0.002740688	0.005612998
EYA2	-0.157211725	0.002741655	0.005614402
FER1L6	-0.157201661	0.002743401	0.0056174
DHRS3	0.15712337	0.002757018	0.00564429
GPBP1	0.157122895	0.0027571	0.00564429
ARHGAP25	-0.157108692	0.002759577	0.00564878
WEE1	-0.157101844	0.002760772	0.005650645
S100A16	0.157083382	0.002763996	0.005656662
TMEM217	-0.157078986	0.002764764	0.005657653
ZBBX	0.157056367	0.00276872	0.005665165
CECR4	0.157027996	0.002773688	0.005674749
TMEM49	0.156981857	0.002781786	0.005690731
NPY	0.156960941	0.002785464	0.005697097
USP14	-0.156960905	0.00278547	0.005697097
CD3D	0.156954048	0.002786677	0.00569898
SLC36A1	-0.156943298	0.00278857	0.005702266
PPBP	-0.156918582	0.002792926	0.005710587
GPR183	-0.156913563	0.002793811	0.005711251
PZP	-0.156913491	0.002793824	0.005711251
ANKRD34A	-0.156911322	0.002794207	0.005711447
LNP1	-0.156874896	0.002800641	0.005724011
FAM36A	0.156798684	0.002814146	0.005751023
GGA2	-0.156790112	0.002815669	0.005753545
INPP5D	-0.15677495	0.002818364	0.005758461
C10orf129	0.156757141	0.002821533	0.005764344
ACR	-0.156733193	0.002825799	0.005772468
COL19A1	-0.156724682	0.002827316	0.005774975

ZNF814	0.156722717	0.002827667	0.005775099
SLC34A2	-0.156716139	0.002828841	0.005776903
LOC100130	0.156667324	0.002837563	0.005794122
CYS1	-0.156654386	0.002839879	0.005798257
SPSB4	-0.156615736	0.002846808	0.005811807
SLC25A16	-0.156588364	0.002851724	0.005821247
SUCLG1	0.156567229	0.002855526	0.00582841
CDX2	0.156552819	0.00285812	0.005833107
DHFR	-0.156526544	0.002862856	0.005842174
POLR3B	-0.156491473	0.002869189	0.005854498
AP4B1	0.156478291	0.002871572	0.005858761
FAM127A	0.156476525	0.002871892	0.005858812
MZF1	-0.15643097	0.002880144	0.005875045
TIMM17A	0.156395096	0.002886657	0.005887728
KCNF1	0.156357234	0.002893546	0.005901175
PDHB	0.156305077	0.002903061	0.005919972
GCM1	0.156225946	0.00291755	0.00594891
GAL	0.156212722	0.002919977	0.005953251
LYN	-0.156204689	0.002921453	0.00595565
DLL4	-0.156185394	0.002925001	0.005962271
WIPF3	-0.156182375	0.002925556	0.005962793
ADPRHL1	0.156168591	0.002928093	0.005967353
TLN2	-0.156158439	0.002929962	0.005970552
LOC401010	0.156131541	0.002934922	0.005979507
SLC45A1	-0.156131345	0.002934958	0.005979507
HMGN4	0.156125542	0.002936029	0.005981077
SOX2OT	-0.156109599	0.002938973	0.005986462
JAG2	-0.156083833	0.002943736	0.005995552
SERPINI1	0.156080704	0.002944315	0.005996118
KCTD17	0.156050139	0.002949976	0.006007032
C9orf43	0.156045987	0.002950746	0.006007985
SLC38A5	0.156023735	0.002954874	0.006015776
ITIH5	-0.156017779	0.00295598	0.006017413
C15orf58	-0.155949123	0.002968756	0.006042506
TMEM63A	-0.155947261	0.002969103	0.006042506
CDH10	0.155946646	0.002969218	0.006042506
LHX9	-0.155938273	0.002970779	0.006045067
FBN2	-0.155934453	0.002971492	0.006045899
SST	0.155924265	0.002973394	0.006049151
GPR119	0.155901153	0.002977712	0.006057317
ZDHHC4	0.155892985	0.002979239	0.006059804
NPTX1	-0.155879275	0.002981804	0.006064403
C16orf92	0.155798388	0.00299698	0.006094646
GCA	-0.155753741	0.003005387	0.006111117
TAF1A	-0.1557216	0.003011452	0.006122825
C7orf61	0.155669046	0.003021393	0.00614241

BFSP2	0.15566312	0.003022516	0.006144065
ZNF583	-0.155655212	0.003024015	0.006146485
ANKAR	-0.15561351	0.003031931	0.006161946
PKP4	-0.155570251	0.003040162	0.006178044
SLC15A4	-0.155542906	0.003045376	0.006188007
SNORD15B	0.155473788	0.00305859	0.006214224
CAMKK1	-0.155468576	0.003059589	0.006215619
GRB10	-0.155415018	0.003069867	0.006235475
THG1L	0.155414383	0.003069989	0.006235475
FER1L4	0.155396831	0.003073364	0.006241694
ZFP28	-0.155390505	0.003074582	0.00624353
PCSK1N	0.155361082	0.00308025	0.006254402
ITFG2	-0.155343457	0.003083649	0.006260666
NPRL3	0.155315641	0.003089021	0.006270934
WBSCR26	0.155283404	0.003095258	0.006282955
CD34	-0.155278552	0.003096198	0.006284222
CUL7	0.155239267	0.003103816	0.006299042
KCNAB3	-0.155223597	0.003106859	0.006304575
RBM24	0.155208604	0.003109774	0.006309847
CIITA	-0.155181038	0.003115139	0.006320089
GOLGA2B	0.15517128	0.00311704	0.006323301
ZNF471	-0.155160872	0.003119069	0.006326773
TFCP2L1	-0.155115608	0.003127907	0.006344053
YY1AP1	0.155112881	0.00312844	0.006344488
BRD2	-0.155096085	0.003131725	0.006350024
SNAP29	0.155095668	0.003131807	0.006350024
FOXO3B	-0.155076528	0.003135555	0.006356976
LOC723809	0.155066481	0.003137524	0.006360321
MST1R	-0.155053325	0.003140104	0.006364903
NUDCD2	0.155044866	0.003141764	0.00636762
CCDC45	0.155041825	0.003142361	0.006368182
NFKBID	-0.155006026	0.003149396	0.00638179
VIPAR	-0.155000072	0.003150568	0.006383514
ARL4A	0.154972841	0.003155931	0.006393729
KHDRBS3	0.15488341	0.003173601	0.006428875
C9orf91	-0.154875944	0.00317508	0.006431217
OR10J5	-0.154852944	0.003179641	0.0064398
CDC27	-0.154824117	0.003185366	0.006450739
HMX2	0.154819466	0.003186291	0.006451955
LYG2	0.154812242	0.003187727	0.006454208
UGGT2	-0.154798924	0.003190377	0.006458916
TPM4	-0.154749297	0.003200269	0.006478283
TSEN2	0.154718907	0.00320634	0.006489913
HOXB13	0.154671068	0.003215918	0.006508638
HIST1H4H	0.154665127	0.003217109	0.006510388
CCDC135	0.154617778	0.003226618	0.006528967

SH3GL2	-0.154612776	0.003227624	0.006530339
FOXG1	0.154548933	0.003240489	0.006555216
PLEKHF1	0.154548493	0.003240577	0.006555216
GATSL2	-0.154519666	0.003246402	0.006566331
DAGLB	0.154513853	0.003247577	0.006568042
CSRP1	-0.154492979	0.003251802	0.006575918
GGTLC1	0.154486421	0.003253131	0.006577937
REEP1	-0.154466071	0.003257256	0.006585609
PPP1R3A	0.154457528	0.003258989	0.006588062
SCNN1A	-0.154456829	0.003259131	0.006588062
C21orf96	-0.154450965	0.003260321	0.006589799
FAM43A	-0.154396741	0.003271345	0.00661141
SETD3	-0.154374765	0.003275822	0.006619787
KIF23	0.154349111	0.003281056	0.006629691
VEGFB	0.154307101	0.003289643	0.006646368
CREB3L1	-0.154302591	0.003290566	0.006647559
TMEM5	0.154281516	0.003294883	0.006655604
LOC150197	0.154233179	0.003304803	0.006674965
SNCAIP	-0.154214855	0.003308571	0.006681897
SCRG1	-0.15420636	0.003310319	0.006684749
LOC100133	0.154181703	0.003315397	0.006694326
TLR8	-0.154143999	0.003323176	0.006709353
VWA2	-0.154139608	0.003324083	0.006710154
METTL7A	-0.154138816	0.003324247	0.006710154
TTC35	0.154103244	0.003331604	0.006724322
LASS3	0.154061602	0.003340234	0.006741059
C20orf186	0.154051326	0.003342367	0.00674468
KLRF1	-0.154040657	0.003344583	0.006748467
KIN	0.154036653	0.003345415	0.006749462
TAS2R4	-0.154028138	0.003347185	0.006752349
MXI1	-0.154026254	0.003347577	0.006752455
DBP	0.153989407	0.003355246	0.006767239
TMEM132B	-0.153966987	0.00335992	0.00677598
PDSS1	0.153922976	0.003369112	0.006793831
TP63	-0.153862825	0.003381713	0.006818074
TMEM194A	-0.153862316	0.00338182	0.006818074
NPAS2	-0.153846103	0.003385223	0.006824246
COL11A1	-0.153831928	0.003388202	0.006828957
GOLGA8C	0.153831717	0.003388246	0.006828957
SLC39A2	-0.15381278	0.003392229	0.006836293
SH3BGRL2	-0.153805671	0.003393725	0.006838616
LRCH4	-0.153798185	0.003395302	0.0068411
PTPRZ1	-0.153764107	0.003402485	0.006854881
PTH1R	-0.153732738	0.00340911	0.006867533
PINK1	-0.153726979	0.003410328	0.006869291
KREMEN1	-0.153724118	0.003410933	0.006869815

VTCN1	-0.153721824	0.003411418	0.006870097
VPS26B	-0.153711463	0.00341361	0.006873403
SLC17A3	0.153710802	0.00341375	0.006873403
CLDN10	-0.153675241	0.003421284	0.006887875
MASTL	-0.153642256	0.003428285	0.006901273
PCDHB19P	-0.153635853	0.003429646	0.006903314
ATXN2	-0.153588446	0.003439735	0.006922922
FPR1	-0.153564508	0.003444839	0.006932495
TAS2R50	-0.153496317	0.003459418	0.006961131
RNF213	-0.15345965	0.003467281	0.006976247
CPEB1	-0.153455877	0.00346809	0.006977172
MREG	-0.153434217	0.003472743	0.006985827
SNORA8	-0.153426307	0.003474444	0.006988542
ZNF404	0.153406059	0.003478801	0.006996598
RPA4	-0.153375681	0.003485346	0.007009055
C13orf16	0.153370354	0.003486496	0.007010658
C5orf62	-0.153334859	0.00349416	0.007025361
C6orf122	-0.153329241	0.003495375	0.007027094
ELANE	-0.153306861	0.003500217	0.007036118
SDHAP1	-0.153269796	0.00350825	0.007051072
SCUBE1	-0.153269267	0.003508364	0.007051072
MAP6D1	0.153250931	0.003512344	0.007058359
GPR81	-0.153236965	0.003515379	0.007063744
HIST2H2AB	0.153231621	0.00351654	0.007065365
HSP90B3P	-0.153221895	0.003518655	0.007068901
EPHA7	-0.153212098	0.003520787	0.00707247
DACH2	0.153203731	0.003522608	0.007075416
SDC3	-0.153157643	0.003532657	0.007094883
C22orf41	0.153113644	0.003542274	0.007112971
MTHFD1L	0.153113173	0.003542377	0.007112971
FAM40A	-0.153080259	0.003549587	0.007126729
CCND3	0.153067781	0.003552324	0.007131505
POTEF	-0.153054217	0.003555301	0.007136763
TMEM87A	-0.153029195	0.003560799	0.007147079
SOX15	-0.152976446	0.003572415	0.007169671
44088	0.152969255	0.003574001	0.007172132
C21orf122	0.15295748	0.0035766	0.007176624
GALNT9	-0.15295047	0.003578148	0.007179007
CHURC1	-0.152942223	0.00357997	0.007181938
S1PR2	0.152917114	0.003585522	0.00719163
TSPAN33	0.152917108	0.003585523	0.00719163
TTC31	-0.152911601	0.003586742	0.00719335
SCARNA9	0.152870879	0.003595765	0.007210721
NOXO1	0.152851764	0.003600008	0.007218502
ROPN1B	-0.152831956	0.003604409	0.0072266
DHRS4	0.15280337	0.00361077	0.007238623

ABHD3	-0.152771902	0.003617783	0.007251953
ABHD6	-0.152761786	0.00362004	0.0072553
PCDHA10	-0.152760123	0.003620412	0.0072553
TPH1	-0.152759521	0.003620546	0.0072553
VPREB3	0.152755871	0.003621361	0.00725557
FAP	-0.152755654	0.003621409	0.00725557
CDCA7	0.152749371	0.003622812	0.007257651
TRPV1	-0.152644108	0.003646392	0.007304154
RADIL	-0.152565417	0.00366411	0.007338907
KRT81	-0.152550828	0.003667404	0.007344765
PRMT3	-0.152548076	0.003668025	0.007345271
EFNB1	-0.152482465	0.003682872	0.007374261
ACTBL2	-0.152478442	0.003683784	0.007375346
ARPC5	0.152466541	0.003686484	0.007380008
LAT	0.152453935	0.003689345	0.007384994
KRTAP10-6	-0.152442866	0.003691859	0.007389284
SPP1	0.152430579	0.003694652	0.00739413
SSX2	0.152413333	0.003698575	0.007401238
MEMO1	0.152379141	0.003706363	0.007416078
RCVRN	-0.152369383	0.003708589	0.007419786
MTVR2	-0.152332754	0.003716954	0.007435775
C20orf200	-0.152278844	0.003729296	0.007459716
MIAT	-0.152264376	0.003732615	0.007465604
C9orf106	-0.152259038	0.00373384	0.007466683
SLC7A9	0.152258608	0.003733939	0.007466683
ULK1	-0.152257125	0.003734279	0.007466683
GAS7	-0.152192739	0.003749086	0.007495538
ARID5A	0.152188752	0.003750005	0.007496622
CRLS1	0.152146837	0.003759675	0.007515199
ZNF251	0.152144193	0.003760286	0.007515665
CLIP2	-0.152141756	0.003760849	0.007516036
WNT5A	-0.152138134	0.003761686	0.007516955
TOPBP1	-0.152126416	0.003764394	0.007521613
PROC	0.152114411	0.003767171	0.007526406
CARD8	-0.152102674	0.003769888	0.007531078
HIATL2	0.152094528	0.003771775	0.007534092
SETDB2	-0.152089467	0.003772947	0.00753497
C17orf73	0.152089364	0.003772971	0.00753497
BPIL1	0.152058832	0.003780052	0.007548354
GAGE2B	0.15205429	0.003781106	0.007549702
RBM14	0.152039786	0.003784475	0.007555671
INTS10	-0.152027761	0.00378727	0.007560493
ZNF142	-0.151978281	0.00379879	0.007582731
ADPRH	-0.151967218	0.00380137	0.007587121
FLII	-0.151942657	0.003807104	0.007597804
PGBD5	-0.15194004	0.003807716	0.007598263

GZMH	0.151937736	0.003808254	0.007598575
CMTM5	-0.151928238	0.003810474	0.007602243
REM2	0.151855143	0.003827598	0.007635643
PLP1	-0.151850856	0.003828605	0.007636885
MECP2	-0.151838795	0.003831438	0.007641771
KLHL34	-0.151833875	0.003832594	0.007643312
CCDC19	0.15181626	0.003836736	0.007650594
PTCRA	0.151815078	0.003837014	0.007650594
DGKA	-0.151727934	0.003857566	0.007690803
UGT3A1	-0.151713091	0.003861077	0.007697032
CHI3L1	-0.151696182	0.003865079	0.007704239
S100A5	0.151682139	0.003868407	0.0077101
CD4	-0.151663462	0.003872835	0.007718154
SNRPN	0.151657417	0.00387427	0.007720241
C20orf72	0.151634544	0.003879702	0.007730292
SERINC4	-0.151601937	0.003887458	0.00774497
SMAD2	-0.151588645	0.003890623	0.007750501
MEOX1	-0.151584971	0.003891499	0.00775147
AGPHD1	0.151573275	0.003894287	0.007756248
ARID3A	0.151502467	0.003911204	0.007789162
PARK2	-0.151488586	0.003914528	0.007795003
NUP214	-0.15143875	0.003926483	0.007818028
ATF3	-0.151410655	0.003933238	0.007830694
DNAH11	-0.151358	0.003945925	0.007855168
UGT2B15	-0.151351963	0.003947382	0.007857283
ZYG11A	0.151340024	0.003950265	0.007862236
GPR126	-0.15133699	0.003950998	0.007862908
CLK2P	-0.151315645	0.003956158	0.00787239
NUMBL	-0.151312076	0.003957021	0.007873321
BAI2	-0.151289547	0.003962475	0.007883385
LRP5L	-0.151275628	0.003965847	0.007889307
SNX21	0.151272094	0.003966704	0.007890223
HCG27	0.15126976	0.00396727	0.007890561
TCEA1	0.151246608	0.003972888	0.007900793
MATN4	0.151245288	0.003973208	0.007900793
BCL7B	0.15118297	0.003988366	0.007929735
UROC1	-0.151182177	0.003988559	0.007929735
SHMT1	-0.151154872	0.003995217	0.007942179
ANKRD13D	0.15113587	0.003999857	0.007950608
NCRNA0016	0.151129844	0.004001329	0.007952741
TAF9B	-0.151068609	0.004016318	0.007981736
PAK4	0.151062301	0.004017865	0.007984013
FAM7A2	-0.151042728	0.004022669	0.007992761
GPR107	-0.151039021	0.004023579	0.007993772
KLF4	-0.150985462	0.004036752	0.008019144
DPT	-0.150980107	0.004038071	0.008020964

FLJ25328	0.150947648	0.004046076	0.008036063
C11orf87	-0.150942827	0.004047266	0.008037625
SLC7A4	-0.15093319	0.004049646	0.00804155
TMEM25	-0.150878997	0.004063054	0.008067369
ANKS1A	-0.150855678	0.004068835	0.008077863
LOC284788	0.150854409	0.00406915	0.008077863
WDR86	-0.150844618	0.00407158	0.008081881
FLRT3	-0.15083777	0.00407328	0.00808445
CPLX3	0.150829239	0.004075399	0.00808785
PGM3	-0.150817933	0.004078209	0.00809262
MGAT4A	-0.15081339	0.004079339	0.008094055
HS1BP3	0.150803382	0.004081828	0.008098187
TLE2	-0.150795706	0.004083738	0.00810117
MAGEA4	0.150783944	0.004086667	0.008106172
CTBP1	0.150763184	0.004091841	0.008115626
HIST1H4D	0.150749415	0.004095275	0.00812163
CD3G	-0.150724937	0.004101388	0.008132149
BBC3	0.150724904	0.004101396	0.008132149
LOC647309	-0.15070996	0.004105132	0.008138746
NOL9	-0.150644928	0.004121425	0.008170234
CECR7	0.150636571	0.004123523	0.008173579
SCARNA18	0.150623752	0.004126743	0.008179148
FSCN1	-0.1506149	0.004128968	0.008182744
BEX5	0.150584098	0.004136718	0.008197287
EFHD2	0.150551199	0.00414501	0.008212902
LIX1	0.150485576	0.004161596	0.008244944
SMEK3P	0.150480808	0.004162803	0.008246515
UNC93B1	0.150473728	0.004164596	0.008249248
UBE2J1	-0.150429396	0.004175842	0.008269905
KCNQ3	-0.150429343	0.004175856	0.008269905
SUSD2	-0.150424098	0.004177188	0.008271721
ZNF780A	0.150353328	0.004195202	0.008306567
DLX6	0.150344605	0.004197427	0.008310147
PRRX1	-0.150339782	0.004198658	0.008311757
KIAA1429	0.150316909	0.0042045	0.008322494
C3orf67	-0.150299975	0.004208829	0.008330236
C6orf123	-0.15027191	0.004216014	0.008343626
KCNJ5	-0.150251783	0.004221173	0.008353006
MAS1L	-0.150235682	0.004225304	0.00836035
ALG6	0.150195398	0.004235656	0.008380001
SYT2	-0.15018909	0.004237279	0.008382379
SIRT2	0.150147015	0.00424812	0.00840299
GCNT7	-0.150140249	0.004249865	0.008405122
MAPK7	-0.150139564	0.004250042	0.008405122
INPP5E	-0.150118009	0.004255607	0.008415293
BRF2	0.150105409	0.004258864	0.008420897

KCNK10	0.15009761	0.004260881	0.008424048
C8A	-0.150073502	0.00426712	0.008435547
PRRT2	-0.149965397	0.004295201	0.008490216
LOC100130	0.149942637	0.004301134	0.008501101
PPP2R2D	0.14992819	0.004304904	0.008507708
PDIA6	0.149906065	0.004310684	0.008518284
PCDHB3	-0.149852141	0.004324799	0.008545329
DKK3	-0.14981786	0.004333794	0.008562253
RAD1	0.14981614	0.004334246	0.008562296
C14orf101	-0.149801733	0.004338031	0.008568703
DPPA4	-0.149800522	0.00433835	0.008568703
DDN	0.149751404	0.00435128	0.008593389
C19orf30	0.149744461	0.00435311	0.008596151
LOC285780	-0.149723751	0.004358574	0.008606089
ADM2	0.149716477	0.004360495	0.008609027
RRP15	0.149695268	0.004366099	0.008619238
ERLIN1	-0.149689082	0.004367735	0.008621613
DIO3	-0.149637267	0.00438146	0.008647846
TAS2R10	-0.149632885	0.004382622	0.008649283
TSHB	-0.149604113	0.004390261	0.008663501
ACAT2	0.149598386	0.004391784	0.008665647
TDRD12	-0.149577096	0.004397446	0.00867596
SYT5	0.149491658	0.004420236	0.008720059
HSP90AB4P	0.149480864	0.004423122	0.008724889
ITPRIPL2	-0.149472883	0.004425258	0.008728237
BRI3BP	0.14941995	0.004439445	0.008755353
CHRDL1	-0.149385601	0.004448673	0.008772684
KLK3	0.149381148	0.004449871	0.008774176
EPHB6	-0.149372882	0.004452095	0.008777693
L3MBTL2	0.149322858	0.004465575	0.008803398
CDK3	0.149290102	0.004474421	0.008819965
ECH1	0.149269881	0.004479891	0.008829872
PHF14	0.149193301	0.004500658	0.008869926
OCIAD1	-0.149161612	0.004509277	0.008886033
SPINK4	0.149152555	0.004511743	0.008890013
TMEM17	-0.149140388	0.004515057	0.008895664
FUNDC2P2	0.149131228	0.004517555	0.008899703
FDX1	-0.149081542	0.004531121	0.008925546
RNF133	-0.149078851	0.004531856	0.008925902
NOD2	-0.149077603	0.004532198	0.008925902
C14orf1	0.149065559	0.004535492	0.008931507
TTC9	-0.149057047	0.004537822	0.008935212
BREA2	0.149054097	0.00453863	0.008935918
NDST4	0.149049117	0.004539994	0.00893772
TBX10	0.14904553	0.004540976	0.008938771
ZNF282	0.149042829	0.004541716	0.008939344

INPP5B	-0.149037181	0.004543264	0.008941506
FCRL1	-0.14900479	0.004552149	0.008957563
STAT6	-0.14900416	0.004552322	0.008957563
IDH2	-0.148960229	0.004564398	0.008980437
C5orf43	-0.148953986	0.004566117	0.008982592
GAGE8	0.148952974	0.004566395	0.008982592
PLEKHA6	-0.148930773	0.004572512	0.008993735
SOX30	-0.148921942	0.004574946	0.00899731
GPR65	-0.148920903	0.004575233	0.00899731
MTMR9L	-0.148884367	0.00458532	0.009016256
TGFB2	-0.148873575	0.004588303	0.009021232
CD9	-0.148793912	0.004610379	0.009063741
SEMA6A	-0.148740585	0.00462521	0.009092001
DKFZP434L1	0.14873346	0.004627195	0.009095005
GDF3	-0.148719291	0.004631145	0.00910187
FZD10	-0.148712581	0.004633016	0.009103943
RAP1A	-0.148712231	0.004633114	0.009103943
CXorf65	0.148654232	0.004649318	0.009134883
TFAM	-0.148614977	0.004660315	0.009155585
ZNF804A	-0.148609444	0.004661866	0.00915773
TSPAN8	0.148598185	0.004665026	0.009163032
MUTED	0.148586136	0.004668409	0.009167657
EVI2A	-0.148585479	0.004668593	0.009167657
IP6K1	0.148584881	0.004668761	0.009167657
ZNF649	-0.14856353	0.004674762	0.009178535
ZFP14	-0.148530243	0.004684131	0.009196024
ABCB7	-0.148513532	0.004688841	0.009204363
PDLIM7	0.148506094	0.004690939	0.009207574
TSPAN4	0.148459434	0.004704118	0.009232532
IFT57	-0.148409655	0.004718214	0.009259286
MFGE8	-0.148290134	0.004752216	0.009325095
IL10RA	-0.148250942	0.004763414	0.009346146
DET1	-0.148222775	0.004771476	0.009361043
ZNF496	-0.148203396	0.00477703	0.009371016
GPR68	-0.148168657	0.004787001	0.009389651
DBF4	0.148126013	0.004799266	0.009412782
BMP1	-0.14812094	0.004800727	0.00941472
TMEM80	0.148113617	0.004802837	0.00941793
NAGA	-0.148046506	0.00482221	0.009454988
CCDC46	-0.148029444	0.004827146	0.009463735
SNORA12	0.148011368	0.004832381	0.009473065
TAS2R31	-0.14800554	0.00483407	0.009475443
NKPD1	0.147959182	0.004847523	0.00950012
VPS37D	0.147958872	0.004847613	0.00950012
ZCCHC9	0.147953967	0.004849039	0.009501978
FAM157A	-0.147942164	0.00485247	0.009507767

GTF2IRD2	-0.147935684	0.004854355	0.009510524
HIST1H2BH	0.147922838	0.004858094	0.009516913
FOXP1	0.147895001	0.004866204	0.009531863
CDON	-0.147880908	0.004870315	0.009538977
TBC1D10A	-0.147860517	0.004876268	0.009549697
PLD2	-0.147780467	0.004899702	0.009594647
ZNF550	-0.147769419	0.004902944	0.009600052
GDA	-0.147739463	0.004911745	0.009616339
GRB7	0.147709829	0.004920465	0.009632464
WDR6	0.147678017	0.004929842	0.009649872
CD70	0.147647569	0.004938831	0.009666518
YPEL5	-0.147640229	0.004941	0.009669814
C12orf69	-0.147638134	0.00494162	0.009670075
ST6GAL1	-0.147590719	0.004955655	0.009696588
ANKK1	0.147581793	0.004958302	0.009700813
RIPK3	-0.147574645	0.004960421	0.009704007
LOC92249	-0.147553582	0.004966674	0.009715284
FAM138F	0.147524617	0.004975282	0.009731167
SSH3	-0.147521028	0.00497635	0.0097323
NFATC1	-0.147417218	0.005007321	0.009791908
C14orf73	0.147410141	0.005009438	0.009795087
LOC100131	-0.147396451	0.005013537	0.009802139
C12orf39	0.147394236	0.005014201	0.009802474
AP3M1	-0.147379074	0.005018744	0.009810393
CORO1B	0.147367064	0.005022346	0.00981647
C22orf13	-0.147344947	0.005028984	0.009828481
HOXA1	-0.147307185	0.005040337	0.009849702
ASCC1	-0.147267577	0.00505227	0.009872053
PIH1D2	0.147262198	0.005053892	0.009874254
P2RY6	0.147257665	0.00505526	0.009875957
FEZ1	-0.147204263	0.005071397	0.009906511
GOLGA6L9	0.147191698	0.005075201	0.009912969
PTPRS	-0.14717618	0.005079902	0.009921178
HOXD11	0.14717168	0.005081266	0.009922869
GHRLOS	0.147158318	0.005085318	0.009929808
KDELR2	0.147152124	0.005087198	0.009932504
YBX2	0.147122966	0.005096053	0.009948819
SLC30A3	0.147118618	0.005097375	0.009950423
C2CD4C	-0.147073893	0.005110988	0.00997602
POM121L8P	-0.147069026	0.005112472	0.009977937
GTPBP1	-0.147060956	0.005114932	0.009981761
CCDC88C	-0.147058161	0.005115784	0.009982446
CCNC	-0.147043785	0.005120171	0.009990027
ALDH16A1	0.147017745	0.005128125	0.010004565
HPGD	-0.146984735	0.005138224	0.010023286
WIF1	0.146969903	0.005142767	0.010031166

ADAM21P1	-0.146962352	0.005145082	0.010034698
SNAR-C4	0.146957691	0.005146511	0.010036502
MRPL30	0.146952902	0.005147979	0.010038383
ATP6V0A1	0.146855307	0.005177993	0.010095921
AGER	0.146839397	0.005182901	0.0101045
GART	-0.146819173	0.005189145	0.010115684
C6orf162	0.146810989	0.005191674	0.010119623
TMEM198	0.146770414	0.005204228	0.010143101
C2orf62	0.146759494	0.005207612	0.010148702
PCK1	-0.146749954	0.005210569	0.010153472
ACOT2	-0.146739604	0.005213779	0.010158734
GCSH	-0.146732789	0.005215894	0.01016186
TDRD1	-0.146710866	0.005222702	0.010174128
SLC22A2	-0.146698922	0.005226415	0.010180365
ASFMR1	-0.146683966	0.005231067	0.010187854
C1orf38	-0.146683272	0.005231283	0.010187854
SUZ12P	0.146679692	0.005232397	0.010189028
TAF7L	-0.146676456	0.005233404	0.010189993
GPATCH2	-0.146588833	0.005260746	0.010242177
C1orf126	-0.146587272	0.005261234	0.010242177
RAB4A	0.146549092	0.005273189	0.010264447
BCAP29	-0.146526547	0.005280259	0.010277205
XCL2	0.14652031	0.005282217	0.010279476
CPM	-0.146519541	0.005282458	0.010279476
LRRC47	0.14649469	0.005290265	0.010293662
LOC644936	-0.146422784	0.005312912	0.010336013
VSIG4	-0.146422287	0.005313069	0.010336013
NPEPPS	-0.14640633	0.005318106	0.010344802
EEF1A1P9	0.146367238	0.005330466	0.010367831
OR7E5P	0.146362375	0.005332005	0.010369813
GFOD1	-0.146350118	0.005335887	0.010376348
C9orf103	0.146288527	0.005355429	0.010413335
GYG1	0.146277544	0.005358921	0.010419107
ZNF473	0.146207085	0.00538137	0.010461732
AAA1	0.146202247	0.005382915	0.010463714
TSPY2	0.146177405	0.005390852	0.010478119
STK36	-0.146154066	0.005398318	0.010491608
FAM189A2	-0.146150967	0.00539931	0.010492512
SERPINF2	0.146131654	0.005405497	0.01050351
TUBB2B	0.146083681	0.005420892	0.010532396
LIPM	-0.146075625	0.005423481	0.010536384
LOC100130	0.146074002	0.005424002	0.010536384
SIGLEC8	-0.146045564	0.005433152	0.010553128
IQCK	-0.146043134	0.005433934	0.010553618
ATP9A	-0.146023465	0.005440271	0.010564895
MAPK8IP1	-0.14600491	0.005446255	0.010575485

INTS1	0.14594023	0.005467161	0.010615045
USH2A	-0.145930035	0.005470463	0.010620421
MRPS6	0.14591459	0.005475469	0.010629102
UTS2	0.145901374	0.005479755	0.010636387
PCDH1	-0.145890398	0.005483318	0.010642264
GRB2	0.145874345	0.005488531	0.010651345
RICH2	-0.145768205	0.005523116	0.010717417
ERAS	0.145750866	0.005528784	0.01072737
SIPA1	0.145744954	0.005530718	0.010730077
OR52N4	-0.1456846	0.005550495	0.010767398
BTF3L4	0.145624279	0.005570325	0.010804813
MMGT1	-0.14560941	0.005575223	0.010813261
MIER2	0.145602331	0.005577556	0.010816733
RAPSN	0.145595154	0.005579922	0.010820267
C1orf27	-0.14558047	0.005584766	0.010828607
PTN	-0.145536789	0.005599199	0.010855535
NGFRAP1	0.145529857	0.005601493	0.010858924
SLC26A4	-0.145521117	0.005604386	0.010863475
BLMH	0.145514023	0.005606735	0.010866971
ABCC6	-0.145484332	0.005616576	0.010884986
CMTM3	0.145476558	0.005619156	0.010888925
FAIM3	-0.145464258	0.005623239	0.010895777
MTSS1L	-0.145446329	0.005629196	0.010906258
HIST1H2AK	-0.145417141	0.005638905	0.010924006
BCL6	-0.145409164	0.005641561	0.010928089
CDCA7L	0.145405584	0.005642753	0.010929336
SFRS5	-0.145394874	0.005646322	0.010935114
ZXDC	-0.145391757	0.005647361	0.010935114
TNFRSF12A	0.145391688	0.005647384	0.010935114
CHST8	-0.145389476	0.005648121	0.010935479
ADCYAP1	-0.145378364	0.005651827	0.01094159
LOC100129	0.145336886	0.005665679	0.010967341
CHMP2B	-0.145334309	0.005666541	0.010967943
CSNK1G1	-0.145307802	0.00567541	0.010983449
OSBPL10	-0.14530707	0.005675655	0.010983449
AQP7P1	0.145291465	0.005680883	0.010992497
MYO3B	-0.145271257	0.005687659	0.01100454
TMEM86B	0.145263885	0.005690133	0.011008256
WDR76	0.145257871	0.005692151	0.011011092
LONP1	0.14524855	0.005695281	0.011016077
C20orf3	-0.145190739	0.005714729	0.011052621
CCR2	-0.145173857	0.00572042	0.01106254
ASPM	0.145172229	0.005720969	0.01106254
EDA	-0.145167613	0.005722526	0.011064476
B3GALT5	-0.145156334	0.005726332	0.01107076
RBPJL	0.145150896	0.005728168	0.011073235

C3orf48	-0.145142949	0.005730852	0.011077347
GABRG1	-0.145135592	0.005733337	0.011081076
RNASE1	0.145112327	0.005741203	0.011095203
ZNF431	-0.145101485	0.005744873	0.011101217
ITM2C	0.145074429	0.005754038	0.011117849
TMCO3	-0.145048984	0.00576267	0.011133447
FSD2	-0.145012184	0.005775174	0.011156523
TEAD4	0.14500895	0.005776274	0.011157566
TAF5	0.144999692	0.005779425	0.011162568
TNFRSF13B	0.144958378	0.005793501	0.01118867
MYF6	0.144928984	0.005803535	0.011206962
CTNNBIP1	0.144911435	0.005809533	0.011217457
CHRM3	-0.144908486	0.005810541	0.011218316
RHOU	-0.144906749	0.005811135	0.011218375
NKD2	-0.144868702	0.005824161	0.011242432
ZNF461	-0.144855288	0.00582876	0.011250219
DDX58	-0.144851527	0.00583005	0.011251618
FAM187B	0.144844886	0.005832329	0.011254925
CXorf42	0.144771225	0.005857654	0.011302701
CPNE7	0.144762522	0.005860653	0.011307392
NFYA	0.144755554	0.005863055	0.01131093
CCL11	-0.144737632	0.005869237	0.011321759
ETV6	-0.144701445	0.005881736	0.011344772
BIK	0.144698599	0.00588272	0.011345572
C21orf62	-0.144663062	0.005895021	0.011368194
FAM20B	-0.144661128	0.005895691	0.011368385
PLCB2	-0.14465638	0.005897336	0.011370457
RAPGEFL1	0.144611229	0.005913003	0.01139956
FASN	-0.144589647	0.005920505	0.011412918
EIF4A2	-0.144577945	0.005924576	0.01141966
UTP3	-0.144508838	0.00594867	0.011464992
SAMM50	0.144480353	0.005958627	0.01148307
FAM57B	0.144471729	0.005961644	0.011487774
FAM48B2	-0.14446699	0.005963303	0.011489858
LIMS2	-0.144452286	0.005968452	0.011498667
PYHIN1	-0.144431457	0.005975753	0.011511619
PEX7	-0.144429175	0.005976553	0.011512047
PSORS1C1	0.144406017	0.005984681	0.011526588
LGALS9B	-0.144386931	0.005991386	0.011538387
TYR	-0.144351865	0.006003725	0.01156103
CSDE1	-0.14428077	0.00602881	0.011608069
FAM123B	-0.144279333	0.006029318	0.011608069
ENOX1	-0.144266787	0.006033755	0.011615488
LYZ	0.144226097	0.006048165	0.011642103
ZBTB32	0.144220148	0.006050274	0.011645038
C11orf82	0.14418306	0.006063439	0.01166925

DCPS	0.144174664	0.006066423	0.011673864
RCC2	0.144111436	0.006088937	0.011716055
PREB	0.144098549	0.006093535	0.01172377
PEX2	0.144062424	0.00610644	0.011747464
DNMT1	-0.144056086	0.006108706	0.01175069
KLHDC2	-0.144048995	0.006111244	0.01175324
RANBP3	0.144047567	0.006111755	0.01175324
STK35	-0.144047432	0.006111803	0.01175324
CDC42BPG	-0.144018093	0.00612231	0.011772016
BTN3A1	-0.144016869	0.006122749	0.011772016
NOL8	-0.143996052	0.006130215	0.011785233
NEUROD4	0.143991318	0.006131914	0.011787361
GNS	-0.143984303	0.006134432	0.011791064
ENDOU	-0.143978145	0.006136643	0.011794176
LOC339535	0.143949477	0.006146948	0.01181284
GOLGA6D	-0.143944887	0.006148599	0.011814873
KCNK15	-0.143915998	0.006159001	0.011833719
PTPN14	-0.143891612	0.006167794	0.01184947
GRIN2A	-0.143800502	0.006200745	0.011911222
YIPF2	0.143799433	0.006201132	0.011911222
VPS33A	0.143797142	0.006201963	0.011911669
UBR5	-0.143763711	0.006214095	0.01193382
LOC401588	-0.143732262	0.006225528	0.011954623
INPP5K	0.143725192	0.006228101	0.01195841
HNRNPK	-0.143707957	0.006234376	0.011969306
PVRL2	0.143702364	0.006236414	0.011972064
PIM2	0.14368379	0.006243186	0.011983909
HOXC9	0.14367923	0.006244849	0.011985946
RNF19B	-0.143666883	0.006249355	0.011993439
MYST1	0.14365867	0.006252355	0.011998038
ABCC1	-0.143649935	0.006255546	0.012003005
IGFBP2	0.143633702	0.00626148	0.012013234
ATP2A3	-0.143616905	0.006267625	0.012023866
TRPM8	-0.14354773	0.006292991	0.012071366
SVOP	0.143534455	0.00629787	0.012079534
PDC	-0.143532843	0.006298463	0.012079534
VSIG2	-0.143506391	0.006308194	0.012096754
NAP1L3	-0.143505136	0.006308656	0.012096754
HBD	-0.143464746	0.006323543	0.012124131
SNORD116-	-0.14344355	0.006331367	0.012137965
CAMKV	0.143383928	0.006353424	0.012179078
LST-3TM12	-0.143369056	0.006358937	0.012188472
LIPI	-0.143361792	0.006361631	0.012192463
CYBA	0.143335043	0.00637156	0.012210318
DNAJA3	-0.143331901	0.006372728	0.01221138
ZFP41	0.14328744	0.006389265	0.012241891

SAMD12	-0.143282074	0.006391264	0.012244542
SEPHS2	0.143252033	0.006402463	0.012264818
TFE3	-0.143209038	0.006418521	0.012294398
LOC283267	-0.143207058	0.006419262	0.012294633
CA9	0.143195697	0.006423512	0.01230159
CYGB	-0.143188077	0.006426363	0.012305868
GABRA1	0.143171326	0.006432636	0.012316696
SCARNA10	0.14316687	0.006434306	0.012318709
DAPP1	-0.143147114	0.006441713	0.012331704
FXYD1	0.1431217	0.006451253	0.01234878
GJB4	-0.143111301	0.00645516	0.012355071
RNF157	0.143077522	0.006467867	0.012378202
CPVL	0.143042071	0.006481226	0.012402577
ADAM28	-0.143040011	0.006482003	0.012402872
TEKT5	0.143020509	0.006489364	0.012415764
KRTAP5-7	0.143006914	0.006494499	0.012424395
ZNF260	-0.142993256	0.006499662	0.012433078
RYR2	-0.142957902	0.006513044	0.012457479
PRR23C	0.14295531	0.006514026	0.01245816
UBE2B	0.142933273	0.00652238	0.012472941
RABEP2	0.14291616	0.006528875	0.012484162
ENHO	0.142883624	0.006541239	0.012506602
ALG12	0.142851744	0.006553373	0.0125286
ANKRD26P1	0.142832477	0.006560717	0.012541435
ZNF620	0.142823421	0.006564171	0.012546834
CDC42EP1	0.142813505	0.006567955	0.012552862
TRPA1	-0.142811705	0.006568642	0.01255297
PARP1	0.142755737	0.006590039	0.012592652
MRPL16	0.142738564	0.006596616	0.012604011
NOMO3	0.142727598	0.006600819	0.012610833
HMMR	0.142718239	0.006604409	0.01261648
CPA2	0.142712776	0.006606505	0.012619273
CLUAP1	0.142708608	0.006608105	0.012621118
IWS1	-0.142606727	0.00664731	0.01269478
PCGF2	-0.142588005	0.006654537	0.012707365
KCTD5	-0.142586139	0.006655257	0.012707522
C17orf97	-0.142551778	0.006668541	0.012731666
GLB1L3	0.142540898	0.006672752	0.01273794
SLA	-0.142539982	0.006673107	0.01273794
MAGEA9B	0.142504621	0.00668681	0.012762874
MAOA	-0.142475189	0.006698235	0.012783456
SDHAP3	0.142470134	0.006700199	0.012785979
ICOSLG	-0.142437762	0.006712789	0.012808777
VEPH1	-0.142423709	0.006718261	0.01281799
ARL6IP6	-0.142395543	0.00672924	0.012837707
SKA2	0.14238981	0.006731477	0.012840744

SYNCRIP	-0.142368141	0.006739937	0.012855651
KLHDC7A	-0.142302426	0.006765653	0.012903464
COL16A1	-0.142290062	0.006770501	0.012911474
C16orf74	0.142288267	0.006771204	0.012911579
EPHA1	-0.142230471	0.00679391	0.012952759
RABL5	0.142229986	0.006794101	0.012952759
TBC1D26	0.142222973	0.006796861	0.01295678
P2RY11	0.142186227	0.006811338	0.012983134
RASGRP4	-0.142170111	0.006817695	0.012994009
C5orf60	0.142144986	0.006827618	0.013011676
ZNF419	0.142081801	0.006852629	0.013057397
TTTY10	-0.142081066	0.00685292	0.013057397
LOC284837	-0.142076722	0.006854643	0.013059429
LOC678655	0.142036726	0.006870522	0.013088429
DKK4	0.142022954	0.006875996	0.013097606
KRTAP10-4	-0.142011706	0.006880471	0.013104876
ELOVL3	0.141988628	0.00688966	0.013121122
TRMT61B	-0.141927621	0.006914004	0.013166225
PLA1A	-0.141918935	0.006917476	0.013171578
MGC45800	-0.141911578	0.006920418	0.01317592
WDR91	-0.141899058	0.006925428	0.013184198
NRGN	0.141840333	0.006948968	0.013227748
GLRX5	0.141834654	0.006951248	0.013230824
APBB3	-0.141829792	0.006953201	0.013233276
FAM27B	0.141818933	0.006957564	0.013240314
SPOP	0.14178969	0.006969326	0.013261431
FAM83F	-0.141755243	0.006983204	0.013286568
SLC25A13	-0.141729463	0.006993606	0.013305089
CD52	0.141647218	0.007026886	0.013367125
STAU1	-0.141612545	0.007040958	0.013392614
PRELID2	0.141577869	0.007055056	0.01341815
SCG3	0.141569284	0.007058551	0.013423514
APOD	-0.141564524	0.007060489	0.013425917
WWP2	-0.141561573	0.007061691	0.01342662
HEATR7B2	0.141560307	0.007062206	0.01342662
BDH2	-0.141537448	0.007071523	0.013443048
MEG8	0.141530546	0.007074338	0.013447116
FAT2	-0.141434404	0.007113655	0.013520561
C15orf54	-0.141419086	0.007119937	0.01353121
C4orf47	0.141413476	0.007122239	0.013534294
CREG1	-0.141411276	0.007123142	0.013534718
CST4	0.141366597	0.007141502	0.013568309
PIWIL2	-0.141317521	0.007161717	0.013605419
CCL22	-0.141310273	0.007164707	0.0136098
CEP250	0.141306672	0.007166193	0.013611325
CXXC4	-0.141289499	0.007173284	0.013623492

HAUS6	-0.141268932	0.007181783	0.013638333
LIN7A	0.141265791	0.007183081	0.013639499
GTDC1	-0.141256999	0.007186718	0.013645104
IGF2BP2	0.141248732	0.007190139	0.013650297
PDLIM2	-0.141222	0.007201211	0.013667771
PCBD2	-0.141221906	0.00720125	0.013667771
GJB3	-0.14122154	0.007201402	0.013667771
DPRXP4	-0.141218276	0.007202755	0.013669036
ZNF227	-0.141194433	0.007212645	0.013686501
BCAN	0.141169045	0.00722319	0.013705204
LOC100132	0.141139141	0.007235627	0.013727496
CLK3	0.141092974	0.007254867	0.013762685
RAB34	0.141090039	0.007256091	0.013763698
GPR15	-0.14108294	0.007259055	0.013768007
RAB37	0.14106893	0.007264905	0.013777791
FAM138E	0.141056736	0.007270001	0.013786142
TMED3	0.141049257	0.007273128	0.013790759
PTGIR	-0.141045848	0.007274553	0.013791331
UBASH3B	-0.141045224	0.007274814	0.013791331
ERCC3	-0.141035003	0.00727909	0.013798123
ADH1C	-0.141026233	0.007282761	0.013803768
LYRM1	0.140940204	0.007318858	0.013870866
SDC2	0.140929835	0.00732322	0.013877812
ASB11	-0.140921139	0.007326879	0.013883425
C14orf33	0.140912955	0.007330325	0.013888633
FHOD1	-0.140909658	0.007331713	0.013889942
CHST11	-0.140895606	0.007337634	0.013899837
PABPC1L2A	0.140892896	0.007338777	0.013900679
SKP2	-0.14087458	0.007346501	0.013913987
CIDEC	0.140853299	0.007355485	0.013929677
LILRA6	-0.14082209	0.007368678	0.013953335
PRKCB	-0.140808074	0.00737461	0.013963241
GDI2	0.140792292	0.007381295	0.013974568
ZMYND8	-0.140789585	0.007382442	0.013975412
KCNAB1	-0.140737396	0.007404588	0.014014794
SPTBN2	-0.140737243	0.007404653	0.014014794
C17orf87	-0.140728807	0.007408239	0.014020248
C4orf38	-0.140714263	0.007414424	0.01403062
LOC149134	0.14067344	0.007431809	0.014062183
CYP4B1	-0.140664255	0.007435725	0.014068256
IGFN1	-0.140645259	0.007443831	0.014082255
CEPT1	-0.140628776	0.007450871	0.014094235
CCDC9	0.140609358	0.007459172	0.014107488
GRPR	-0.140609072	0.007459295	0.014107488
PLEKHG3	-0.140575207	0.007473792	0.014132624
OXSM	0.140574712	0.007474004	0.014132624

C8orf83	-0.140526348	0.007494752	0.014170511
C3orf34	-0.140523024	0.00749618	0.014171866
ESRRB	-0.140486222	0.007512006	0.014200437
CHMP5	-0.140461055	0.007522845	0.014219577
PSPN	0.140445911	0.007529374	0.014230568
C15orf2	-0.140422322	0.007539554	0.014248456
CN5H6.4	0.140391057	0.007553066	0.014272636
SH2D6	0.140367697	0.007563176	0.014290384
RBKS	0.14027767	0.007602251	0.014362852
MINA	0.140270962	0.007605169	0.014367003
PRSS37	-0.140267078	0.00760686	0.014368833
IFI44	-0.140240888	0.007618267	0.014389016
RIN3	-0.140237535	0.007619728	0.014390412
DDX47	0.140233358	0.00762155	0.014392486
ZNF276	-0.140215209	0.007629467	0.014406071
GNG8	0.140157945	0.007654494	0.014451958
NACAD	-0.140153801	0.007656308	0.014454013
LEMD1	0.140141086	0.007661877	0.014463154
STAT1	-0.140125518	0.007668699	0.014474661
C14orf143	0.140090546	0.007684045	0.014502252
TNIP1	0.140059269	0.007697793	0.014526822
IQUB	-0.14003427	0.007708798	0.014546162
METTL4	-0.14003267	0.007709503	0.014546162
PPP1R1B	0.139997019	0.007725221	0.014573934
C20orf134	0.139995968	0.007725685	0.014573934
CUL3	-0.139977538	0.007733823	0.014587903
GABRG2	0.139973786	0.00773548	0.014589648
TIGD7	0.139892318	0.00777155	0.01465629
FCGR3B	-0.139848696	0.007790925	0.014691438
ZCCHC18	-0.139818001	0.007804584	0.014715802
CNPY4	0.13980634	0.007809779	0.014724203
CMAS	-0.139706098	0.007854563	0.014807234
TNFRSF11A	-0.139675374	0.007868335	0.014831792
HIST1H4A	0.139668433	0.007871449	0.014836258
ODAM	0.139657786	0.007876228	0.014843861
MORC4	-0.139641277	0.007883643	0.014855931
ARHGAP28	-0.139638739	0.007884784	0.014855931
ALG1	0.139638548	0.00788487	0.014855931
POP1	0.139636537	0.007885774	0.014856228
ATP2B2	-0.139626289	0.007890381	0.014863502
DEFA6	0.139612981	0.007896368	0.014873372
INF2	-0.13960821	0.007898515	0.01487601
TIFAB	0.139591959	0.007905833	0.014888385
ALDOB	-0.139540179	0.007929191	0.014930961
ALMS1P	-0.139522485	0.007937187	0.014944604
NCRNA0008	-0.139507617	0.007943911	0.014955851

FZD3	-0.139472108	0.007959991	0.014984708
FOLR2	0.13944947	0.007970258	0.015002616
MPHOSPH6	0.139416354	0.007985297	0.015029505
CCDC88A	-0.139404241	0.007990805	0.01503845
FAM182A	0.139391414	0.007996641	0.015048011
PGAP3	0.139385513	0.007999327	0.015051643
CA5B	-0.139376877	0.008003259	0.01505762
FBXW10	0.139373288	0.008004894	0.015059273
TRIT1	0.139302377	0.008037254	0.015118723
C11orf90	-0.139293321	0.008041395	0.015125084
B3GNT1	0.139287299	0.00804415	0.015128836
EEF2	-0.139275048	0.008049757	0.015137952
C1orf63	-0.139235254	0.008067993	0.015170814
DTHD1	-0.139227943	0.008071348	0.015175688
GPR113	-0.139200844	0.008083792	0.015197651
ANP32C	0.139161637	0.008101826	0.015230118
AVPR2	-0.139153997	0.008105345	0.015235295
OR14J1	0.139129565	0.008116606	0.015255022
CBFB	-0.139117079	0.008122366	0.015263324
USP30	-0.139116667	0.008122556	0.015263324
CYP7B1	-0.139111777	0.008124814	0.015266125
PRPS2	-0.139049856	0.008153442	0.015318471
MFSD7	-0.139011922	0.008171024	0.015350056
AP3B1	-0.138995588	0.008178605	0.015362849
CD8B	0.138988134	0.008182067	0.015367903
FMNL1	-0.138984963	0.00818354	0.01536922
SLC22A17	-0.138913355	0.008216868	0.015430357
POTEE	-0.138890763	0.008227408	0.015448693
GRAP	-0.13886744	0.008238302	0.01546769
ZFYVE20	-0.138865196	0.008239351	0.015468201
PYY	0.138847371	0.008247686	0.015482389
RYR1	-0.138822823	0.008259177	0.015502499
PHLPP1	-0.13879271	0.008273293	0.015527531
ZNF395	-0.138771864	0.008283078	0.01554443
UBXN8	-0.138752573	0.008292142	0.015559974
CCDC76	-0.138739526	0.008298276	0.015570018
ABCB4	-0.138728819	0.008303314	0.015578003
ZNF324B	-0.138678362	0.008327091	0.015619703
CNBP	0.138678323	0.00832711	0.015619703
TYW1B	-0.138655507	0.008337882	0.015638436
FLJ45079	-0.138642584	0.008343988	0.015648415
GCLM	-0.138636887	0.008346681	0.015651992
LRP12	-0.138634063	0.008348016	0.015653022
VAMP1	-0.138598591	0.008364806	0.015683027
MRPS27	0.138556452	0.00838479	0.015719015
HHEX	0.138535418	0.008394781	0.015736264

C13orf35	0.138532395	0.008396218	0.015737476
S100A6	0.138522746	0.008400806	0.015744593
SIGLECP3	-0.138497965	0.008412598	0.015764024
UBFD1	-0.138497632	0.008412756	0.015764024
CA1	-0.138465147	0.008428237	0.015791546
ZNF223	-0.138439239	0.008440602	0.015813225
LOC100272	-0.138433383	0.008443399	0.015816977
PRMT5	0.138385262	0.008466414	0.0158586
BTRC	-0.138372319	0.008472614	0.015868721
GJB7	0.138357793	0.008479577	0.015880269
LOC338588	-0.138340943	0.00848766	0.015893912
TSPAN17	0.138322491	0.00849652	0.015909008
FLVCR2	-0.13831429	0.008500461	0.015914889
STAC3	0.13830337	0.00850571	0.01592322
CDK5R1	-0.138297073	0.008508739	0.015927392
GEMIN8	0.138252217	0.008530338	0.015966324
PAPSS1	-0.138196148	0.008557406	0.016015482
LETMD1	0.138187696	0.008561493	0.016021625
SMARCD1	0.138168264	0.008570896	0.016037714
EXOSC10	0.138153558	0.008578018	0.016048757
UPP2	-0.138152751	0.008578409	0.016048757
PRKCA	-0.138148683	0.00858038	0.016050937
HBEGF	-0.138124763	0.008591978	0.016071123
NICN1	0.13810403	0.008602043	0.016088438
ZNF311	-0.138087225	0.008610208	0.016102198
OSTF1	-0.138068412	0.008619358	0.016117794
ZNF747	-0.138058901	0.008623987	0.016124936
FGF13	-0.138045495	0.008630514	0.016135626
LAMC1	-0.138023507	0.008641231	0.016154147
ELF3	0.137982896	0.008661056	0.016189687
KRT24	0.137962393	0.00867108	0.016206903
ZNF835	-0.137958961	0.008672759	0.01620852
FHOD3	-0.137954769	0.00867481	0.016210832
UBTF	0.13795041	0.008676943	0.016213163
SFMBT1	-0.137948893	0.008677685	0.016213163
WBP2NL	-0.137893868	0.008704657	0.01626203
LOC253039	0.137873433	0.008714692	0.016279251
PRKAB2	-0.137871511	0.008715636	0.016279488
CADM3	-0.137831776	0.008735181	0.016314465
APOA1	0.137824729	0.008738652	0.016319415
S100Z	-0.137810892	0.00874547	0.016330617
WBP11	-0.137804237	0.00874875	0.01633521
ZFP91-CNT	-0.137759242	0.00877096	0.016375145
PLVAP	-0.137736471	0.008782219	0.016394628
RGMB	-0.137717312	0.008791702	0.016410792
LRRC41	-0.13768292	0.008808748	0.01644107

TM9SF1	-0.137675813	0.008812274	0.016446109
NTM	-0.137664924	0.008817679	0.016454655
ARMCX3	-0.137607851	0.008846057	0.016504605
UPK1A	0.137607757	0.008846104	0.016504605
RGS19	0.137593634	0.008853138	0.016516182
UBE2G2	-0.137571286	0.00886428	0.016535419
TUBB6	0.1375479	0.008875953	0.016555643
GOLGA6L1	0.137528918	0.008885437	0.016571782
C6orf163	0.137480763	0.008909539	0.016615177
IFNG	0.137451455	0.008924236	0.016641026
HEATR1	-0.13741989	0.008940089	0.016669027
KMO	-0.137411689	0.008944212	0.016675153
GJA4	-0.137396609	0.008951798	0.016687733
CCDC108	0.137370466	0.008964962	0.016710709
AGFG2	0.137360378	0.008970047	0.016718622
CYP4X1	-0.137352851	0.008973842	0.01672413
CACNA1H	-0.137338811	0.008980925	0.016735765
ZNF605	0.137312869	0.008994025	0.016758609
WDR63	-0.137285268	0.009007983	0.016783046
SLC29A2	0.137282945	0.009009158	0.016783047
MAGEC2	0.137281937	0.009009669	0.016783047
C19orf33	0.137250233	0.009025727	0.016811387
SV2C	-0.137240661	0.00903058	0.016818854
KIAA1310	-0.137205308	0.009048526	0.0168507
TSEN15	0.137189223	0.009056701	0.016864348
CDKN2BAS	0.137163203	0.009069939	0.01688742
PHF21A	-0.137098534	0.009102917	0.016946634
OXCT1	-0.137097502	0.009103444	0.016946634
DDIT4L	-0.137075189	0.009114848	0.016966277
CNTROB	0.137044939	0.009130328	0.016993503
CCDC160	-0.137036832	0.009134481	0.016999643
LOC285696	-0.137017832	0.00914422	0.017015369
MRPL42	0.137017013	0.00914464	0.017015369
MBOAT1	-0.137010946	0.009147752	0.01701957
FAM127C	-0.136986686	0.009160204	0.017041146
NLRP11	-0.136976817	0.009165275	0.017048987
LGI4	0.136946811	0.009180706	0.017076095
AOX1	-0.136920606	0.009194201	0.017099599
IFNGR2	0.136854852	0.00922814	0.017161118
CYP20A1	-0.136848162	0.009231599	0.017165948
GLRA1	-0.136833169	0.009239357	0.017178768
DCLRE1B	-0.136827076	0.00924251	0.017183028
PTF1A	0.136814294	0.00924913	0.017192823
IDO2	-0.136813569	0.009249506	0.017192823
BARX1	0.136796249	0.009258483	0.017207903
GLG1	-0.136769827	0.009272192	0.017231775

VAPA	0.136730569	0.009292595	0.017268081
LZIC	0.136705221	0.009305789	0.017290319
MOGAT3	0.136704243	0.009306298	0.017290319
FXYD2	0.136691148	0.009313122	0.017301383
GSPT2	-0.13664777	0.009335757	0.017341814
FRMD1	0.136628494	0.00934583	0.017358907
NONO	0.136590006	0.009365973	0.017394699
LOC286367	-0.136586973	0.009367562	0.017396027
MEG3	0.136582964	0.009369663	0.017398306
RARRES3	0.13655589	0.009383861	0.017422173
FAM78B	0.136555117	0.009384266	0.017422173
DNHD1	-0.13654347	0.00939038	0.017431899
LPCAT2	-0.136538059	0.009393221	0.017435549
SHISA6	-0.13653029	0.009397303	0.017441499
IRAK1BP1	-0.136439233	0.009445254	0.017528863
SLC22A15	-0.136427867	0.009451254	0.017538365
ZBTB3	-0.136425516	0.009452496	0.017539034
C1orf212	0.1364007	0.009465611	0.017561484
PDE1B	-0.136399287	0.009466358	0.017561484
ALX3	-0.136396495	0.009467835	0.017562587
CALY	0.136391148	0.009470663	0.017566197
SMC1B	0.136312027	0.009512605	0.017642348
EIF2C2	0.136254378	0.009543269	0.017697569
HNRNPR	-0.136244226	0.009548678	0.017705952
RBM15	0.136234904	0.009553647	0.017713516
C12orf34	0.136223174	0.009559903	0.017723465
SLC9A3R2	0.136183276	0.009581209	0.017761312
RCOR3	-0.136170456	0.009588064	0.017772365
TMEM232	-0.136148449	0.009599841	0.01779254
ACOT12	-0.136131723	0.009608801	0.017806137
MGC12982	0.136131416	0.009608966	0.017806137
CYB561	-0.136086988	0.009632801	0.017848645
PMS2L3	0.136042691	0.009656619	0.017891113
BARHL2	0.136034047	0.009661273	0.01789807
MAOB	-0.13603082	0.009663011	0.017899624
DEFA4	-0.136022421	0.009667536	0.01790634
ANAPC2	0.135993934	0.009682895	0.017933122
TMEM104	0.135979239	0.009690827	0.017946143
LRRN4	-0.135975895	0.009692633	0.017947819
DCAF4L2	0.13595937	0.009701561	0.017962681
SLC45A3	-0.13594665	0.009708438	0.017973742
RUNDC3A	0.13592499	0.009720159	0.017993769
TCEB3C	-0.135895866	0.009735939	0.018021306
GATA1	-0.135874253	0.009747663	0.018041331
CITED1	0.135803354	0.009786213	0.018110997
ATCAY	0.135798539	0.009788836	0.018114168

LOC152024	0.135794492	0.009791041	0.018116565
PXT1	-0.135775405	0.009801447	0.018134134
ART3	0.135764748	0.009807261	0.018143205
C7orf60	-0.135735401	0.009823288	0.018171167
LAPTM4A	-0.13571539	0.00983423	0.018189717
SFRS8	0.135713391	0.009835323	0.018190051
ZNF439	-0.135670989	0.009858546	0.018231307
PPAPDC3	-0.135665038	0.009861809	0.018235648
CSRNP3	-0.135658719	0.009865275	0.018240364
TNNI3	0.135642143	0.009874372	0.01825413
SAA3P	0.135641811	0.009874554	0.01825413
MEIS1	-0.135608731	0.009892731	0.018286035
RFWD3	-0.135593611	0.00990105	0.018299713
HOXA2	-0.135582306	0.009907273	0.018309516
SPINT3	-0.135560837	0.009919101	0.018329675
IGJ	-0.135554102	0.009922815	0.018334835
GH2	0.135505266	0.009949776	0.018382947
SNX10	-0.135495534	0.009955157	0.018391182
ANKRD2	0.135487737	0.00995947	0.018397443
HOMER3	0.135479825	0.009963847	0.018403823
DDX4	0.135459873	0.009974895	0.018422521
C11orf93	0.135434636	0.009988885	0.018446647
GKAP1	0.135428623	0.00999222	0.018451096
FAM138D	0.13539241	0.010012331	0.018486516
ANXA4	-0.135380928	0.010018715	0.018496589
FAM166B	0.1353273	0.010048579	0.018550005
KIAA1683	-0.135284573	0.010072429	0.01859231
SPSB1	-0.135269897	0.010080633	0.018605728
TST	0.135223695	0.010106498	0.018651739
MAK16	-0.135197587	0.010121141	0.018677031
TMEM164	0.135151376	0.010147103	0.018723205
CCDC54	-0.135135005	0.010156314	0.018738466
MRI1	-0.13511154	0.010169531	0.018761113
FRMPD2	-0.135101955	0.010174934	0.018769342
43901	0.135098826	0.010176698	0.018770857
C9orf50	0.135038505	0.010210765	0.018831948
FAM71F2	-0.134989336	0.010238608	0.018881552
HPS3	0.134986185	0.010240394	0.018883097
TMX4	-0.134954919	0.010258137	0.018914064
LHFPL4	0.134943962	0.010264361	0.018923787
C7orf49	0.134934345	0.010269827	0.018932112
CPN1	0.134924488	0.010275432	0.018940691
F13A1	-0.134911499	0.010282822	0.018951875
VCX2	0.134910479	0.010283403	0.018951875
KIAA0226	-0.13489975	0.010289511	0.018961377
DDX11	0.134889036	0.010295614	0.018970868

NUP155	-0.134870659	0.010306089	0.018988413
OPN1LW	0.134857847	0.010313397	0.018998944
KANK3	-0.134857294	0.010313713	0.018998944
CTPS	-0.134850681	0.010317487	0.019004139
MYOZ3	-0.134824686	0.010332335	0.019029728
TCHP	-0.13481176	0.010339725	0.019041578
CLLU1	-0.134721597	0.010391405	0.019134982
HEPN1	-0.134694455	0.010407007	0.01916194
C17orf55	0.134674388	0.010418555	0.01918143
SYNPO2L	-0.134668471	0.010421963	0.019185929
ACLY	-0.134665371	0.010423748	0.019187443
RAD54B	0.134645308	0.010435311	0.019206951
TMEM201	0.134608461	0.010456576	0.019244313
SLC39A12	-0.13451708	0.010509479	0.019339889
C17orf70	0.134499284	0.01051981	0.019357111
MBL1P	-0.134492073	0.010523998	0.019363028
C17orf96	0.134458686	0.010543409	0.019396951
JAKMIP3	-0.134445031	0.010551358	0.019409781
CLECL1	0.13442413	0.010563534	0.019430385
NKX2-5	0.134404907	0.010574744	0.019449208
DYNC2LI1	0.134391651	0.01058248	0.019461639
LIN28A	0.134389389	0.010583801	0.019462271
EIF3IP1	0.134372943	0.010593407	0.019478136
AGR2	0.134336978	0.010614441	0.01951501
MTL5	0.134330687	0.010618124	0.019519979
LOC143666	0.134318969	0.010624987	0.019530779
LOC653113	0.134317308	0.01062596	0.019530779
FBXO39	-0.134293648	0.010639831	0.01955447
C1orf127	0.134285455	0.010644638	0.019561499
CLCN1	-0.134280511	0.01064754	0.019565026
TUB	-0.134273176	0.010651846	0.019571133
PIK3R2	0.134255917	0.010661984	0.019587953
EPB49	-0.134235551	0.010673959	0.019608144
SCG2	-0.134137695	0.010731661	0.019711787
KIRREL2	0.134136517	0.010732358	0.019711787
MSLNL	0.134112398	0.010746623	0.019734791
SCAMP5	0.13411199	0.010746865	0.019734791
FAM22G	0.134099953	0.010753991	0.01974532
C10orf88	0.134098955	0.010754582	0.01974532
GLOD4	-0.134071912	0.010770607	0.019772919
RCL1	-0.134044328	0.010786975	0.019801143
HSD17B6	-0.134037524	0.010791015	0.019806734
C21orf82	-0.134022382	0.010800012	0.019821421
LOC100134	0.134019208	0.010801899	0.019823057
PTCD3	-0.134008781	0.010808099	0.019832608
NLGN1	-0.13399268	0.01081768	0.01984836

OR7C1	0.133983831	0.010822949	0.019856198
ZBTB45	0.133944831	0.010846196	0.019895893
KLF5	-0.133944182	0.010846584	0.019895893
DGCR8	-0.133913327	0.010865008	0.019926726
FAM22A	0.133912681	0.010865394	0.019926726
SPINK9	0.133910693	0.010866582	0.019927071
LY6G6D	0.133900341	0.01087277	0.019936582
ZNF826	0.133884593	0.010882191	0.019952019
CD1E	-0.133878587	0.010885785	0.019955786
CEP68	-0.133877812	0.010886249	0.019955786
PBXIP1	0.133825109	0.010917838	0.020011445
KHDC1	0.133823803	0.010918622	0.020011445
ZNF302	-0.133819627	0.010921128	0.020014196
BDKRB1	-0.133788009	0.010940123	0.020047162
TRIM63	-0.133758925	0.010957622	0.02007738
MAPK13	0.133729325	0.010975456	0.020108207
ZNF416	0.133701855	0.01099203	0.02013672
PRR4	0.133660085	0.011017275	0.02018111
S100A7	0.133656104	0.011019684	0.020183666
TMEM151B	-0.133645735	0.011025959	0.020193303
LGALS14	0.133606662	0.011049637	0.020233829
DPEP3	0.133605866	0.011050119	0.020233829
GAGE13	0.133594859	0.011056798	0.020244196
FAM171A1	-0.133591196	0.011059021	0.020246406
PRSS8	-0.13357275	0.011070223	0.020264204
CLNK	-0.133571836	0.011070778	0.020264204
ZNF239	0.133543032	0.011088291	0.020294396
GCET2	-0.133519836	0.011102413	0.020318374
EML3	-0.13351735	0.011103927	0.020319278
FBP1	-0.133494288	0.011117984	0.020343131
SOX10	-0.133491597	0.011119625	0.020344266
MRAP	-0.13346453	0.011136145	0.020372619
FABP3	0.133461458	0.011138022	0.02037418
DMRTA2	0.133457976	0.011140148	0.020376199
OR6B1	0.133453198	0.011143068	0.020379421
POM121L1P	-0.133451744	0.011143956	0.020379421
PAQR4	0.133437944	0.011152393	0.020392977
CLN5	-0.133425734	0.011159862	0.020404761
PCDH11X	-0.13331394	0.011228454	0.02052829
TTTY9B	0.13329914	0.011237563	0.020543058
TCEAL6	-0.13321924	0.01128685	0.020630207
AWAT1	0.1332185	0.011287308	0.020630207
TM4SF19	0.13317413	0.011314762	0.020678487
ATG3	0.13316112	0.011322822	0.02069132
GTPBP4	0.133144531	0.011333108	0.020708217
AKD1	-0.133135365	0.011338795	0.020716707

C10orf75	0.13310703	0.011356391	0.020746006
SNORA72	0.133106187	0.011356915	0.020746006
TP53I13	0.133095041	0.011363843	0.020756758
BAALC	-0.133090512	0.01136666	0.020759999
CLCN5	-0.133075247	0.011376156	0.020775438
ST6GALNAC	-0.133064946	0.011382569	0.020785243
SCUBE2	-0.133047754	0.011393279	0.020802893
IRX4	0.133041463	0.0113972	0.020808145
SELT	-0.132994423	0.011426558	0.020858192
ZNF273	-0.132994184	0.011426707	0.020858192
TRIM37	-0.132892468	0.011490418	0.020972567
LOC100127	0.132884802	0.011495232	0.020979431
PNPLA1	0.132878361	0.011499278	0.020984892
ABCG1	-0.132847332	0.01151879	0.021018572
FBXL20	-0.132805781	0.011544962	0.0210644
LOC286467	0.132793667	0.011552603	0.02107641
PKDREJ	-0.132736247	0.011588879	0.021140655
C14orf139	-0.132724279	0.011596453	0.021152534
HIST1H2BI	0.132708935	0.01160617	0.021168319
UGT2B10	-0.132647546	0.011645117	0.021237408
LOC146880	0.13263911	0.011650478	0.02124524
OCM	0.132590643	0.01168132	0.021299532
KIAA1543	0.132584335	0.01168534	0.021304911
AP3S1	0.13257732	0.011689811	0.021311112
GSTA1	0.132554755	0.011704204	0.021335398
PORCN	-0.132548613	0.011708125	0.021340592
MOBKL2A	0.132531304	0.01171918	0.021358787
DTWD1	0.132494495	0.011742718	0.021399729
ZNF167	-0.132476825	0.011754034	0.02141839
ZMYND12	-0.132443142	0.011775628	0.021455777
CLTC	-0.132403816	0.011800886	0.021499831
UBA7	-0.132401989	0.01180206	0.021500003
RTTN	-0.132383982	0.011813642	0.021519134
HSD11B2	0.132374702	0.011819616	0.021528045
SLC22A11	0.132361468	0.011828137	0.021541596
SNORA9	0.132358109	0.011830301	0.021543567
SLC5A8	-0.132346714	0.011837645	0.021554969
RAB25	-0.132327967	0.011849736	0.021575013
CUEDC1	-0.1323219	0.01185365	0.021580167
C8orf56	0.132305957	0.011863944	0.021596933
NHEG1	0.132297539	0.011869382	0.02160427
CADM4	-0.13229636	0.011870144	0.02160427
KCTD16	-0.132269294	0.011887646	0.021634146
FGF3	0.132231654	0.011912022	0.021676528
PPWD1	-0.132227797	0.011914522	0.021679097
LOC220594	-0.132221646	0.011918511	0.021684373

OR7E37P	0.13221513	0.011922737	0.021690081
ZNF749	-0.132207944	0.0119274	0.021696581
C1orf204	-0.132177438	0.011947211	0.021730634
SLFNL1	-0.132158658	0.011959422	0.021750857
CSF3	-0.132154403	0.011962189	0.021753904
KIAA0146	0.132117408	0.011986281	0.021795725
FKSG29	-0.132085551	0.01200706	0.021831517
ZFP36L2	-0.132076016	0.012013286	0.021840842
ALPI	0.132068929	0.012017915	0.021847263
C12orf32	0.132065261	0.012020311	0.021849626
CMAH	-0.131998205	0.012064196	0.021927395
ANO5	-0.131979012	0.012076784	0.02194827
CAMP	-0.131974891	0.012079487	0.021951181
TMEM159	-0.131960627	0.012088852	0.021966194
SHISA7	-0.131940523	0.01210206	0.021988187
PARD3	0.131933495	0.012106681	0.021994576
SLCO6A1	0.131913104	0.012120095	0.022016938
MRPS31	0.131905765	0.012124927	0.022023706
DAO	-0.13190071	0.012128255	0.022027744
MAGEA2	0.131854317	0.012158843	0.022081283
PLOD3	0.131842476	0.012166661	0.022093467
HAGHL	0.131819858	0.012181606	0.022116825
C2orf42	-0.131819648	0.012181745	0.022116825
ASMT	0.131803351	0.012192524	0.022134377
CTNNA3	-0.131796457	0.012197086	0.022138936
TCF25	0.131796196	0.012197259	0.022138936
OMA1	-0.131791101	0.012200632	0.02214304
NFATC3	-0.131775828	0.012210747	0.022159378
HCN3	0.131749392	0.012228272	0.022189161
HSPA2	-0.131742512	0.012232838	0.022195423
ZBTB12	0.131717086	0.01224972	0.02222403
LOC148145	-0.1317068	0.012256556	0.022234406
FSTL3	-0.131701796	0.012259882	0.022238415
TTYH3	-0.131696445	0.012263441	0.022242844
GPRIN2	-0.131694401	0.0122648	0.022243283
PFAS	-0.131661244	0.012286871	0.022281282
TRA2A	-0.13164749	0.012296037	0.022295873
SLC25A45	0.131645625	0.01229728	0.022296097
PIP5K1P1	-0.131643862	0.012298455	0.022296198
RPP25	0.131635419	0.012304086	0.022304375
SLC12A9	0.131607058	0.012323015	0.022336657
MALAT1	-0.131598987	0.012328407	0.022344396
SH3BP2	-0.131577797	0.012342573	0.022368035
SLC2A2	-0.131573634	0.012345357	0.022371045
POLQ	0.131540318	0.012367663	0.022409426
C9orf46	0.131523392	0.012379008	0.022427943

ZNF746	-0.13152121	0.012380471	0.022428554
CIDEB	-0.131517564	0.012382917	0.022430943
RAGE	0.131513809	0.012385436	0.022433466
PIGB	-0.131487581	0.012403043	0.022463315
TCAP	0.131455252	0.012424776	0.022500629
OSGIN1	0.131439015	0.012435704	0.022518371
IL10	-0.131413387	0.01245297	0.022547586
KIF5C	-0.131396073	0.012464646	0.022566676
PCDHB9	-0.131368558	0.012483222	0.022598252
SFTPD	-0.131353491	0.012493404	0.022614629
ODZ1	-0.131346031	0.012498448	0.022621703
MB	-0.131323649	0.012513593	0.022647056
LIAS	0.131320292	0.012515866	0.022649111
YTHDF1	0.131311213	0.012522015	0.022657085
SLC34A3	0.131310427	0.012522548	0.022657085
ZNF833	-0.131252169	0.012562068	0.022726525
PIGP	0.131248336	0.012564672	0.022729172
ITGAD	-0.13124511	0.012566864	0.022731072
SLC35A4	0.131235715	0.01257325	0.022740557
SIDT1	-0.131233524	0.01257474	0.022741187
ZNF318	-0.131183029	0.012609114	0.02279949
PROCR	-0.131182802	0.012609268	0.02279949
RTN2	0.131168414	0.012619078	0.022815155
PITPNC1	0.131114149	0.012656137	0.022880081
ZIC5	0.131102573	0.012664055	0.022892317
CWH43	-0.131074617	0.012683195	0.022924834
ZDHHC7	-0.131064091	0.012690407	0.022935789
PNPLA5	-0.131039056	0.012707578	0.022964738
MIS12	-0.131033221	0.012711583	0.022969891
REXO1L1	0.130994328	0.012738305	0.02301609
MTHFD2	-0.130932179	0.012781108	0.023091334
VAX2	0.130858418	0.012832075	0.023181311
CCDC144C	-0.130832606	0.012849952	0.0232115
WWP1	-0.130803326	0.012870258	0.023246071
HNRPLL	-0.130789747	0.012879684	0.023260988
SMPDL3A	-0.13074216	0.012912768	0.023318623
ZNF48	0.130717322	0.012930065	0.023347427
LSAMP	-0.13071589	0.012931063	0.023347427
AMAC1L3	-0.130691109	0.012948343	0.023376507
MSI2	-0.130684706	0.01295281	0.023382453
DCAF12L1	0.130675061	0.012959544	0.023392487
WNT3A	0.130638996	0.012984746	0.023435855
ZNF365	-0.13061022	0.013004887	0.023470079
KPNA7	0.130557307	0.013041993	0.023534912
HMOX2	0.130532629	0.01305933	0.023564062
SLC25A42	-0.130522724	0.013066295	0.023574493

CKM	0.130454199	0.013114566	0.023659248
NLRC4	-0.130452668	0.013115646	0.023659248
GHITM	-0.130429697	0.013131864	0.023686358
ZNF738	0.130402926	0.013150787	0.023718341
JRK	0.130393374	0.013157545	0.02372838
SCLY	0.130352226	0.01318669	0.023778787
TMEM50A	0.130347669	0.013189921	0.023782461
CSAG1	0.130340212	0.01319521	0.023789843
WDR8	0.130326078	0.01320524	0.023805771
ARL17B	-0.130317356	0.013211433	0.023813103
ARL1	-0.130316982	0.013211698	0.023813103
SPANXC	0.130311824	0.013215362	0.02381755
TBX15	-0.130294712	0.013227522	0.023837309
CXCL6	-0.130291369	0.013229899	0.023839435
ZNF841	-0.130287921	0.013232351	0.023840079
CCDC67	0.1302875	0.01323265	0.023840079
GPR149	-0.130283877	0.013235227	0.023842565
DCXR	0.130267582	0.013246822	0.023861294
XRCC5	0.130239225	0.013267021	0.023895517
RHD	-0.130192474	0.013300382	0.023953437
CALML6	0.130162741	0.013321637	0.023989547
GPC4	-0.130142485	0.013336135	0.024013484
IL1F7	0.130133201	0.013342784	0.024023284
TMEM37	0.130125552	0.013348265	0.024030979
ARAP1	0.130111434	0.013358386	0.024047026
ZSCAN20	-0.130100776	0.013366031	0.024058614
AMBN	0.130087427	0.013375612	0.024073683
PSTPIP1	0.13005477	0.013399075	0.024112283
ATP2B3	-0.130054207	0.013399479	0.024112283
BAGE	0.130048241	0.01340377	0.024117825
DHRS4L1	0.130009215	0.013431865	0.024166194
CD248	-0.12996272	0.013465406	0.02422435
GATC	-0.129958947	0.013468131	0.024227064
EMR2	-0.129939791	0.013481974	0.024249775
DNAH9	-0.129929677	0.013489287	0.024260739
C7orf64	-0.129914699	0.013500124	0.024278036
C5orf28	-0.129908066	0.013504926	0.024284479
DHRS7C	-0.129878729	0.013526182	0.024320504
IGHMBP2	-0.129842125	0.013552744	0.024366065
BPHL	0.129823038	0.013566613	0.024388797
ABHD14B	0.12981834	0.013570028	0.024392735
CDK9	0.129810121	0.013576006	0.024401278
SEC61A2	-0.129806291	0.013578792	0.024404082
ZNF705A	-0.129721396	0.013640678	0.024513093
SNORA10	0.129718587	0.01364273	0.024514569
ZNF480	0.129674891	0.013674683	0.024569769

RTBDN	0.129665841	0.01368131	0.024579458
CPT1B	0.129663237	0.013683217	0.024580667
NLRX1	-0.129641228	0.013699346	0.02460742
TRPV2	0.129638476	0.013701364	0.024608825
BBOX1	-0.129631795	0.013706263	0.024615406
MANSC1	-0.129625167	0.013711126	0.024621918
DKFZp779M	0.129604882	0.013726017	0.024646437
C1orf194	0.12955687	0.013761319	0.024707598
IL20RB	0.12955059	0.013765942	0.02471367
HSDL1	0.129511553	0.013794713	0.02476309
SERPINB2	-0.129507752	0.013797517	0.024764325
C4orf7	0.129507249	0.013797888	0.024764325
KCNC1	0.129502294	0.013801545	0.024768655
ARHGEF37	-0.12948187	0.013816625	0.024793484
SORCS3	0.129473867	0.013822538	0.02480186
C8orf22	0.129456438	0.013835423	0.024822744
PPP1R12C	0.129444322	0.013844387	0.024836588
OR2A14	-0.129435174	0.013851158	0.024846496
PCDHB2	-0.129432904	0.013852838	0.024847273
ETV1	-0.129397043	0.013879413	0.024892697
FAM182B	0.129386877	0.013886955	0.02490398
ELFN2	-0.129364048	0.013903903	0.024932129
NUP50	-0.129354513	0.013910987	0.024942587
CSNK2A2	0.129337413	0.0139237	0.024963133
GPR156	-0.129328468	0.013930354	0.024972167
GALNT12	-0.129327269	0.013931247	0.024972167
ANKRD32	-0.129315747	0.013939822	0.02498529
OR6C2	0.129313279	0.01394166	0.024985872
DISP2	-0.129311941	0.013942656	0.024985872
GNGT2	0.129292537	0.013957111	0.025009526
C1orf229	0.129289332	0.0139595	0.025011556
HSF4	0.129223456	0.014008682	0.025097418
SLC6A20	-0.129171471	0.0140476	0.025164879
ACTRT1	0.129153334	0.0140612	0.025186976
C14orf166B	0.129130297	0.014078492	0.025215682
TP53I3	0.129128126	0.014080123	0.025216335
GRM4	0.129111028	0.01409297	0.025237073
BANK1	-0.129073874	0.014120922	0.025284856
SLC37A1	-0.129068513	0.014124959	0.02528981
ZSCAN4	-0.129054324	0.01413565	0.025306676
FAM84B	-0.129030826	0.014153369	0.025336121
SLCO1B1	-0.129005109	0.014172785	0.025368597
F13B	-0.128951699	0.014213183	0.025438621
C3orf15	-0.128895614	0.014255713	0.025512448
LOC168474	-0.128883391	0.014264996	0.025526767
SGK223	-0.128847532	0.014292263	0.025573263

LRRC20	0.128842348	0.014296208	0.025577487
LRRC36	0.128841056	0.014297192	0.025577487
TAAR1	-0.128834379	0.014302275	0.025584282
GPR25	-0.128822636	0.01431122	0.025597983
NXT2	-0.128742599	0.014372314	0.025704952
SLC7A8	-0.128723548	0.014386891	0.025728711
PSG3	-0.128714545	0.014393783	0.025738726
LASP1	0.128690147	0.014412476	0.025769839
IHH	0.128688117	0.014414033	0.025770309
JAKMIP2	-0.128684169	0.01441706	0.025773407
PRNP	-0.128677699	0.014422023	0.025779965
PROKR1	-0.128622231	0.014464626	0.025853799
USP17L2	-0.128619114	0.014467024	0.025855764
PPP6C	-0.128611174	0.014473132	0.025864359
GEMIN5	-0.128599613	0.01448203	0.025877939
BBS4	-0.128507424	0.014553158	0.026002703
KLHL31	0.128504364	0.014555524	0.026004599
TMEM110	0.128480381	0.01457408	0.026035416
C1orf125	0.128461622	0.01458861	0.026059033
PDE9A	0.128416197	0.014623845	0.026118908
TMEM134	0.128415028	0.014624753	0.026118908
MAPKAPK3	0.128402911	0.014634164	0.026133374
ERBB4	-0.128338375	0.014684383	0.026220702
LCE2D	0.128334202	0.014687636	0.026224159
LOH12CR1	0.128318074	0.014700212	0.02624426
M6PR	-0.128304268	0.014710985	0.026261139
PLIN1	-0.128297727	0.014716091	0.026267899
ETV5	0.128252604	0.01475136	0.026328493
NPLOC4	0.128241262	0.014760237	0.026341977
SERPINA9	0.128235317	0.014764892	0.02634602
PTEN	-0.128234989	0.014765149	0.02634602
PLCD4	0.12820708	0.014787017	0.026382677
WFDC1	-0.128192769	0.014798243	0.026400341
ACVR2B	0.12816905	0.014816863	0.026431192
ARL4C	-0.128124394	0.014851976	0.026491456
NEUROG1	0.128108822	0.014864238	0.026509562
FLJ36000	0.128108121	0.014864789	0.026509562
HIST1H1D	0.128055256	0.014906484	0.026581538
SLC9A3	-0.128041419	0.014917414	0.026598647
LOC100132	0.128009672	0.014942517	0.02663928
C7orf70	0.128009218	0.014942877	0.02663928
HAND1	0.127991869	0.014956611	0.026661378
FBXL7	-0.127979318	0.014966555	0.026676715
DNAJC6	-0.127973533	0.014971139	0.026682499
CDCP2	-0.127961645	0.014980565	0.026696908
GRID2	0.12793267	0.015003561	0.026735496

DEF6	0.127902959	0.015027172	0.026775175
VPS4A	0.127900378	0.015029225	0.026776437
TBC1D10B	0.127887461	0.015039501	0.026792349
WDR90	-0.127880801	0.015044803	0.026799396
DTD1	0.127871858	0.015051923	0.026809682
SYAP1	-0.127837182	0.015079562	0.026856508
CYTIP	-0.127814589	0.015097593	0.026886217
DPCR1	-0.127782051	0.015123595	0.026930115
JOSD1	-0.127779509	0.015125629	0.026931327
SLC9A9	-0.127777004	0.015127632	0.026932487
MORN1	0.127740305	0.015157013	0.026982383
CHAC2	-0.127710573	0.015180853	0.027022407
PDCL2	0.127701358	0.01518825	0.027033157
SNORA21	0.127690413	0.015197037	0.027046381
NAA25	-0.127687681	0.015199232	0.027047869
MSR1	-0.12768256	0.015203346	0.027052772
CCDC43	0.127676855	0.01520793	0.027057962
AOC2	-0.127675549	0.015208979	0.027057962
LRRN1	-0.127664208	0.015218096	0.027071763
NRG3	-0.127613328	0.015259058	0.027139824
RAB11A	0.127613299	0.015259081	0.027139824
C6orf218	0.1275579	0.015303791	0.027216914
PLA2G15	0.127532389	0.015324418	0.027251164
LOC100188	0.127508851	0.015343472	0.027282611
NCRNA0010	0.127496024	0.015353864	0.027298651
TCEAL2	0.127483884	0.015363705	0.02731371
AURKAPS1	0.127436164	0.015402441	0.027380132
GLT6D1	0.12741305	0.015421234	0.027411093
ATG12	0.127410014	0.015423705	0.027413037
ATP6V1G3	0.127402028	0.015430203	0.02742214
STOM	-0.127372231	0.015454473	0.027462821
ZC3HAV1	-0.127366354	0.015459264	0.027468882
XAGE5	0.127349468	0.015473036	0.027490901
DNAH14	0.127343043	0.015478279	0.027497763
MORC2	0.127340338	0.015480486	0.027499232
GTF2H2B	0.127329843	0.015489055	0.027511999
MAPK12	0.127310993	0.015504456	0.027536898
CLPTM1L	0.127290475	0.015521234	0.027563664
ACSL6	-0.127289179	0.015522295	0.027563664
ABCC3	-0.127280255	0.015529597	0.027574174
CHST4	-0.127273216	0.01553536	0.027581947
GNAI1	-0.127254161	0.015550969	0.027607198
KRTAP10-3	-0.127218722	0.015580036	0.027656334
UBE2E3	0.127212732	0.015584953	0.027662597
PDE6B	0.127188442	0.015604909	0.027695549
TBL1X	-0.127178802	0.015612835	0.027707147

RAB3A	-0.12717187	0.015618536	0.027714795
MARK4	0.127165969	0.015623391	0.027720939
USP1	-0.127127189	0.015655329	0.027775135
DRAM2	0.127107703	0.0156714	0.02780117
CT47A2	0.127101965	0.015676135	0.027807092
TCEB3B	-0.127093359	0.015683238	0.027817215
PRSS22	-0.127087945	0.015687709	0.027822667
TCP11L1	-0.127004897	0.015756423	0.027942045
FAM84A	-0.126999339	0.015761031	0.027947728
LOC284551	0.126971359	0.015784247	0.027986404
PLEKHH3	-0.12696531	0.01578927	0.027992818
LSG1	-0.126946623	0.015804796	0.028017849
KCNU1	0.12691417	0.015831792	0.028063207
UCN3	0.126906966	0.01583779	0.028071341
C6orf97	-0.126874178	0.015865115	0.02811727
INSM1	0.126846673	0.015888068	0.028154574
SLC39A10	-0.126845568	0.015888991	0.028154574
ZFAND1	0.126829338	0.01590255	0.028176092
MLL4	-0.126798938	0.015927973	0.028218627
TPK1	-0.126758055	0.015962219	0.028276784
SCTR	-0.126752556	0.015966831	0.028281064
CGB7	-0.126751787	0.015967476	0.028281064
NARS2	-0.126663551	0.01604163	0.02840947
CAPZA2	-0.12666213	0.016042827	0.02840947
SSX1	0.126637988	0.01606317	0.028442965
TRPM6	0.126635003	0.016065686	0.028444892
ABL2	-0.126621797	0.016076826	0.028462084
GPR135	-0.126599714	0.016095467	0.028492553
ING1	-0.126593404	0.016100797	0.028499456
MOCS1	0.126583697	0.016109	0.028511441
KCNQ1	-0.12651101	0.016170538	0.028617815
HOXA11	0.126501296	0.016178778	0.028629853
CIDEA	-0.1264992	0.016180556	0.028630455
GPR35	0.126478847	0.016197833	0.02865848
RTCD1	0.126468223	0.016206858	0.028671901
RBM15B	0.126463174	0.016211149	0.028676944
CD80	-0.126449143	0.016223078	0.028695124
DHRS7B	0.126447696	0.016224308	0.028695124
SPTA1	-0.126439308	0.016231444	0.028705195
PITPNB	-0.126430555	0.016238893	0.028715818
LOC154761	-0.126378973	0.01628285	0.028790993
HBE1	0.126372995	0.016287951	0.028797455
MPZ	0.126356681	0.016301879	0.028819522
KCNV1	0.126264811	0.016380507	0.028955955
UGDH	0.126232429	0.0164083	0.029002512
GRM6	-0.126228454	0.016411714	0.029005972

PLXNB3	-0.126217833	0.016420841	0.029019527
CCL2	-0.126182704	0.016451059	0.029070349
INPP5A	0.126173596	0.016458901	0.029081627
MS4A2	-0.126140147	0.016487732	0.029129598
TGM5	-0.126138704	0.016488976	0.029129598
MBL2	-0.126119455	0.016505589	0.029153759
GRID2IP	-0.12611834	0.016506551	0.029153759
SNORA20	0.126117769	0.016507044	0.029153759
LOC388692	-0.126112281	0.016511783	0.029159543
AMIGO3	0.126076726	0.016542517	0.029211227
MRPL39	-0.126043778	0.016571041	0.029259001
TRIM36	-0.126032679	0.016580659	0.029273389
UHRF1	0.126025191	0.01658715	0.029281617
KGFLP1	-0.126023911	0.01658826	0.029281617
RCN3	0.126017418	0.016593892	0.029288962
PMAIP1	-0.1259922	0.016615779	0.029324995
RANBP9	-0.125963097	0.01664107	0.029367028
ATF5	-0.125945573	0.016656315	0.029391326
SLC2A8	0.125932287	0.01666788	0.029409129
PEMT	0.125915329	0.016682654	0.029432588
PTGS1	-0.125897502	0.016698195	0.029457398
GDI1	0.125879863	0.016713586	0.029481937
RPS6KL1	0.125844676	0.016744326	0.029533545
SDSL	0.125811007	0.016773786	0.029582885
TTC39A	0.125798597	0.016784655	0.029599434
CCDC15	-0.125769867	0.016809843	0.029641086
FOXA2	0.125768262	0.016811251	0.029641086
HMGB3L1	0.125750116	0.016827178	0.029666542
AMPD1	-0.125731423	0.016843599	0.029692862
PSG5	-0.125694133	0.016876398	0.02974805
FABP5	0.125680904	0.016888048	0.02976595
TROVE2	-0.12562019	0.016941602	0.029857699
ACOT9	0.125595514	0.01696341	0.029893488
PAH	-0.125585982	0.016971842	0.029905701
CECR1	-0.125577702	0.016979168	0.029915962
C22orf39	0.125550675	0.017003102	0.029955482
PEX19	-0.125542317	0.017010509	0.029965549
KCNV2	-0.125539322	0.017013164	0.029965549
LYPD4	0.125539135	0.01701333	0.029965549
YEATS2	-0.125492936	0.017054331	0.030035108
FAM164C	-0.125444915	0.017097041	0.030105942
ZNF335	0.125444315	0.017097575	0.030105942
MYH15	-0.125426748	0.017113223	0.030130832
PRDM7	0.125422546	0.017116968	0.030134762
LOC641367	-0.125392929	0.017143383	0.030178597
SNORA38B	0.125359457	0.017173279	0.030228553

SIGIRR	0.125355718	0.017176621	0.030231763
C1R	-0.125321939	0.017206842	0.030282279
ORMDL1	0.125301828	0.017224857	0.030311304
ALS2CL	-0.125288277	0.017237005	0.030330002
CEP70	-0.125278478	0.017245794	0.030342785
DNMT3L	-0.125261692	0.017260859	0.030366609
TDRD9	-0.125241011	0.017279436	0.030394824
LOC401397	0.12524044	0.017279949	0.030394824
ZNF639	-0.125223025	0.017295606	0.030419677
PAGE4	0.125214474	0.017303299	0.030430519
RIMS1	-0.125204686	0.017312107	0.03044332
KCNMB1	-0.125188403	0.01732677	0.030466415
HDAC2	0.12512268	0.017386063	0.030567974
C10orf55	-0.125116971	0.017391221	0.030574343
KIRREL3	-0.125077498	0.017426927	0.030634411
SVIP	0.125068351	0.017435209	0.030645249
AFM	-0.125067291	0.01743617	0.030645249
DERL3	0.125043341	0.017457874	0.030680689
FAM58B	-0.12501976	0.017479268	0.030715576
CNTNAP4	0.124999705	0.017497482	0.030743243
C1orf180	0.124999024	0.0174981	0.030743243
C14orf79	0.124996825	0.017500099	0.030744042
C6orf127	0.124982542	0.017513081	0.030764136
DOK2	0.124950502	0.017542236	0.030812632
C2orf84	-0.124941178	0.017550727	0.030824829
ZNF492	0.124924366	0.017566048	0.030849017
NCRNA0011	-0.124912206	0.017577137	0.030865769
NAP1L6	0.124840401	0.017642742	0.030978241
HNRNPA1L2	0.124814536	0.017666427	0.031017093
PPP1R9B	-0.124793643	0.017685577	0.031047979
SNORA36A	0.12477588	0.017701874	0.031073849
C12orf70	0.124741965	0.017733026	0.031125789
MAP3K8	-0.124730135	0.017743903	0.031142138
EBAG9	0.124720019	0.017753209	0.031155724
HYOU1	-0.124681367	0.017788806	0.031215443
TRAF3	-0.124663714	0.017805084	0.031241255
LAMA4	-0.124627203	0.017838793	0.031297644
ITGB1BP3	-0.124601848	0.017862234	0.03133601
LOC440173	0.124600017	0.017863928	0.031336222
EIF2A	0.124587898	0.017875142	0.031353132
LOC91450	0.124571495	0.017890332	0.031377011
CACNA1A	0.12456168	0.017899426	0.031390196
TTC9C	0.124554561	0.017906024	0.031399003
SLC16A14	-0.124516767	0.017941091	0.031457724
LOC115110	-0.124486149	0.017969543	0.031504838
SLC7A13	0.124447619	0.018005404	0.031564931

C14orf149	-0.124427975	0.018023711	0.031594244
SCHIP1	-0.124416354	0.018034549	0.03161046
GAGE10	0.124400753	0.018049108	0.031633194
RASA4P	0.12438808	0.018060942	0.031651149
ABCC4	-0.124348512	0.018097932	0.031711461
ZNF71	0.12434786	0.018098542	0.031711461
DZIP1L	-0.124296303	0.018146843	0.031793294
FAM129C	-0.124284125	0.018158267	0.031810511
RPL32P3	-0.124247173	0.018192973	0.031868507
OR51E1	-0.124243133	0.018196771	0.031872357
PRAMEF12	-0.124239576	0.018200115	0.03187541
DVL3	-0.124199119	0.018238192	0.031939289
RNF130	-0.124160019	0.018275058	0.032001036
LOC92973	-0.124147632	0.01828675	0.032018695
PRY2	-0.124132025	0.018301492	0.032041688
HPS4	-0.124128309	0.018305004	0.03204502
DGCR11	-0.124119626	0.018313211	0.032056569
LOC651250	-0.124109826	0.018322478	0.032069972
NXF2	0.124107819	0.018324376	0.032070476
C20orf54	-0.124059148	0.018370465	0.032148313
MED12L	-0.124031982	0.018396233	0.03219058
RSPH9	0.124015289	0.018412083	0.032215484
KCNIP2	0.123987896	0.018438119	0.032258033
ZNF701	-0.123986296	0.018439641	0.032258033
HN1L	0.123971821	0.018453412	0.032279288
RNF145	-0.123949965	0.018474223	0.032312853
GCN1L1	0.123946549	0.018477477	0.032315708
TSHR	-0.123936986	0.018486591	0.032328807
MUM1	-0.123927756	0.018495391	0.032340572
MAPRE1	0.123926523	0.018496566	0.032340572
MYL12A	0.123918348	0.018504363	0.032351365
CXorf22	-0.123907455	0.018514758	0.032366697
MUM1L1	-0.123858164	0.018561857	0.032446185
C10orf140	0.123807075	0.018610784	0.032528854
CCL14	-0.123781948	0.018634888	0.032568127
C9orf71	-0.123777735	0.018638933	0.032572338
SLBP	0.12375582	0.018659983	0.032606261
C2orf81	0.123737542	0.018677555	0.032634102
ZNF883	0.123723249	0.018691305	0.032655263
AATK	-0.123704329	0.018709522	0.032684223
HYAL3	0.123695166	0.01871835	0.032696776
LYPD5	-0.123688512	0.018724763	0.032705109
ADRB2	-0.123658052	0.018754143	0.032753552
LILRA2	-0.123649157	0.01876273	0.032765676
PARP16	-0.123639264	0.018772285	0.032779487
MYH2	-0.123600231	0.018810025	0.032842507

PLAC1	0.123572383	0.01883699	0.032886706
KCTD14	0.123538269	0.01887007	0.032939973
GLYAT	-0.123537507	0.018870809	0.032939973
SLU7	0.123530804	0.018877315	0.032945589
SMTNL2	0.123529535	0.018878546	0.032945589
CLSTN3	-0.123529079	0.018878989	0.032945589
CHMP7	-0.123525136	0.018882817	0.032949383
XIRP1	-0.123523365	0.018884537	0.032949497
NEK6	0.123516582	0.018891124	0.032958103
SGK494	0.123495694	0.018911424	0.032989208
ANKHD1	-0.123494827	0.018912266	0.032989208
EEF1A2	-0.123458562	0.018947555	0.033047868
F5	-0.123456428	0.018949634	0.0330486
SAP130	-0.123443235	0.018962488	0.033068121
MMP8	-0.123437724	0.018967859	0.033074592
SOHLH2	0.123433577	0.018971901	0.033078744
ZNF737	-0.123412846	0.018992123	0.033111103
RBMS1	-0.123387707	0.019016669	0.033150994
OR2C1	-0.123341604	0.019061756	0.033226684
SPANXA2	0.123293837	0.019108569	0.033305369
AGAP2	-0.123282159	0.01912003	0.033322428
SNORA79	0.123265637	0.019136253	0.033347785
DEFB4A	-0.123260812	0.019140994	0.033353127
CCDC64B	-0.123200572	0.01920026	0.033453471
PWWP2B	0.123182342	0.019218228	0.033481848
CLEC3B	-0.123143013	0.019257039	0.03354653
LIX1L	0.123112048	0.019287644	0.033596907
CC2D2B	-0.123099574	0.019299985	0.033614729
ACAD10	-0.123098294	0.019301251	0.033614729
SAMD3	-0.12309578	0.01930374	0.033616122
TIAL1	0.123088601	0.019310846	0.033625558
LRRC37A2	-0.123080051	0.019319314	0.033637361
LOC100129	0.123056182	0.019342968	0.033675602
SH3BP5L	0.123011892	0.019386927	0.033749183
ING2	0.122966058	0.01943251	0.033825579
VGLL1	-0.122948766	0.019449731	0.033851586
KCNJ4	-0.122947642	0.019450851	0.033851586
LBR	0.122932372	0.01946607	0.033875113
HNMT	-0.12292184	0.019476574	0.033888656
PRRG3	-0.122921156	0.019477256	0.033888656
POU6F1	-0.122884572	0.01951378	0.033948508
HILS1	0.122883285	0.019515065	0.033948508
ZNF195	-0.122879476	0.019518872	0.033952164
D4S234E	-0.122872691	0.019525654	0.033960995
EMX2OS	-0.122845636	0.019552716	0.034005095
C1orf213	0.122831754	0.019566615	0.034026295

MMACHC	-0.122826573	0.019571805	0.034032349
C5	-0.122799812	0.019598628	0.034076015
RGS3	-0.122771327	0.019627215	0.03412274
AGPS	-0.122765947	0.019632617	0.034129153
C4orf43	0.12274774	0.019650913	0.034157976
C1orf131	0.122739961	0.019658735	0.03416859
PLEKHB2	-0.122680083	0.019719028	0.034270394
GTF2E1	-0.12266243	0.019736835	0.034298348
STAT2	-0.122618223	0.019781487	0.034372944
SLC35F1	-0.122606317	0.019793528	0.034390866
TMEM107	0.122596845	0.019803112	0.034404516
AP1B1	-0.122567874	0.01983245	0.034452482
C11orf65	-0.122558869	0.019841577	0.034465331
LOC729375	0.122555195	0.019845301	0.034468793
PRAM1	-0.122518622	0.019882413	0.034530239
HEPHL1	0.122508611	0.019892582	0.034544888
NFAM1	-0.122504993	0.019896258	0.034548259
LOC100270	0.122493098	0.019908349	0.03456624
SUPT6H	0.122443108	0.01995923	0.034651562
TUSC3	-0.122437975	0.019964461	0.034657622
APOC1P1	0.122417475	0.019985364	0.034690885
C17orf98	0.122409832	0.019993162	0.034701396
NCF4	0.122399017	0.020004201	0.034717531
CNTN2	-0.122396474	0.020006798	0.034719012
ZNF652	-0.122368408	0.020035472	0.034765743
LBX2	0.122347848	0.020056502	0.034798937
SESTD1	-0.122346289	0.020058097	0.034798937
TMIE	0.122343487	0.020060965	0.034800881
OR2A9P	-0.12233349	0.020071198	0.034815601
PDCL	-0.122320216	0.020084794	0.03483615
C1orf106	0.122240273	0.020166844	0.034975415
CUX2	-0.122224827	0.02018273	0.034999918
DMKN	0.122214824	0.020193024	0.035014722
ZNF560	0.122198787	0.020209537	0.035039693
RBBP7	0.122197421	0.020210944	0.035039693
MARCO	-0.122194693	0.020213754	0.035041514
DPF1	0.122160017	0.020249507	0.035097408
SRP72	-0.122160003	0.020249521	0.035097408
CPA6	0.122151387	0.020258413	0.035109764
PLK3	-0.122136117	0.02027418	0.035134031
COX7A1	0.122092835	0.02031893	0.035208518
PRPF40B	0.122085164	0.02032687	0.035219211
ZNF33B	-0.122063068	0.020349756	0.035255797
TULP3	-0.122058241	0.020354758	0.035260103
LTBR	0.122057253	0.020355782	0.035260103
LRRC29	0.122012592	0.02040212	0.035337296

SGCA	-0.121979617	0.020436392	0.035391391
FCAMR	-0.121977966	0.02043811	0.035391391
PPHLN1	0.121977414	0.020438684	0.035391391
FBRSL1	0.121958787	0.020458068	0.035421877
FABP7	0.121933759	0.020484137	0.035463931
ANXA10	-0.121926854	0.020491335	0.035473308
CENPF	0.121924439	0.020493853	0.035473487
KIAA0319	0.121923338	0.020495001	0.035473487
GABRB1	0.121889492	0.020530318	0.035531527
JPH1	-0.121872381	0.020548192	0.035559372
PADI3	-0.121801893	0.02062197	0.035683947
GAL3ST1	0.121789192	0.020635288	0.035703888
SELL	-0.121777457	0.0206476	0.035722087
TP53INP1	-0.121749743	0.020676702	0.035769329
OSR1	-0.121717118	0.020711005	0.035824655
SYT17	-0.121715905	0.020712281	0.035824655
QRSL1	0.121710335	0.020718143	0.035831682
GSTP1	0.121704766	0.020724006	0.03583871
DZIP3	-0.121652851	0.020778724	0.035930216
CETN3	0.121638548	0.020793822	0.0359532
ZNF714	-0.121629102	0.020803797	0.035967326
SPZ1	0.12162222	0.020811068	0.03597391
LINS1	-0.121622077	0.020811219	0.03597391
B3GNT7	-0.121570958	0.020865294	0.036064254
C21orf99	0.121567355	0.02086911	0.03606772
G6PC2	-0.121554798	0.020882414	0.036087581
AKR7A3	-0.121537091	0.020901187	0.036116888
HDHD1A	0.121490228	0.020950941	0.036199722
MRPL49	-0.121487336	0.020954015	0.036201892
PHF10	-0.12148146	0.020960261	0.036209542
LOC152225	-0.121472568	0.020969717	0.036222736
KRBA1	0.121462608	0.020980313	0.036237514
NEUROD1	0.121461105	0.020981912	0.036237514
KRTAP4-4	0.121412186	0.021034025	0.036324369
COL11A2	0.121407799	0.021038704	0.036329298
COL17A1	-0.121391864	0.021055708	0.036355508
CCDC21	-0.121387789	0.021060058	0.036359867
DBN1	0.121377934	0.021070581	0.036374881
TFEB	0.121373037	0.021075812	0.036380757
EMP3	0.121355929	0.021094095	0.036409162
DVL1	0.121330505	0.021121291	0.036452943
CTXN1	0.121318692	0.021133938	0.03647161
GTF2IP1	-0.12126386	0.021192725	0.036569891
CSDC2	-0.121247442	0.021210355	0.036597143
CAND2	-0.121233553	0.021225279	0.03661972
UBE2O	0.12122663	0.021232721	0.036629387

SNORA40	0.12122265	0.021237001	0.036633596
DGKQ	-0.121191293	0.021270744	0.036688626
FAM118A	0.121181816	0.021280952	0.036703054
LOC100287	0.121179897	0.021283019	0.03670344
ANKRD40	-0.121177707	0.021285379	0.036704331
EFEMP2	-0.121170689	0.021292942	0.036714194
GRPEL1	0.121164853	0.021299233	0.036721862
C20orf135	0.121143303	0.021322477	0.036758754
MIMT1	0.121101983	0.021367107	0.036832505
RNF135	-0.12109504	0.021374614	0.036842257
PTX4	-0.121045637	0.021428097	0.036931246
ATXN7L3	-0.121043123	0.021430821	0.036932745
GPR50	-0.120998978	0.021478715	0.03701208
ZNF630	-0.120967218	0.02151323	0.037068348
ZNF777	-0.120964509	0.021516177	0.037070219
SLC25A23	-0.120956931	0.021524419	0.037081212
GRHL2	-0.120949792	0.021532187	0.037091386
PLEKHB1	0.120942832	0.021539764	0.037101228
SP110	0.120935396	0.021547861	0.037111965
GLRX	0.120923119	0.021561234	0.037131786
CCBL2	-0.120877246	0.021611267	0.037213345
MAPK9	-0.120876272	0.02161233	0.037213345
MAGEA12	0.1208706	0.021618524	0.037220792
TXNL4B	0.120850766	0.021640194	0.037254881
PI4KAP1	-0.120840547	0.021651366	0.037270892
MALL	-0.120826073	0.021667199	0.037294925
RBMY1A1	0.120817111	0.021677008	0.037308583
DPY19L2	-0.120807454	0.021687581	0.037323555
C11orf41	-0.120778798	0.021718982	0.037374365
HRH1	-0.120767081	0.021731834	0.037390276
GJC3	-0.120766945	0.021731983	0.037390276
LOC387646	-0.120753729	0.021746486	0.037408871
LOC285074	0.120753673	0.021746548	0.037408871
PCSK9	0.120724289	0.021778823	0.037461156
ZNF341	0.120698362	0.021807336	0.03750696
SHOX2	0.120689031	0.021817605	0.037521383
CRYBA4	0.120674255	0.021833877	0.037545982
SLC22A5	-0.120672618	0.02183568	0.037545982
SUN3	0.120641868	0.021869577	0.037601021
FBRS	0.120617657	0.021896297	0.037643712
METTL10	-0.120588259	0.021928781	0.037696303
LRRIQ4	-0.1205837	0.021933822	0.037701715
MUSK	-0.120517866	0.022006732	0.037823775
SNORA25	0.120501055	0.022025384	0.037852565
BIN3	0.120489806	0.022037873	0.037869128
KAAG1	0.120487698	0.022040213	0.037869128

UPF3A	0.120487237	0.022040726	0.037869128
PIAS3	-0.120481793	0.022046772	0.037876248
CDH4	-0.120466111	0.022064198	0.037902915
CHKB	0.120460926	0.022069962	0.037909547
MRAS	-0.120440262	0.022092948	0.037945758
C17orf93	0.120429263	0.022105191	0.037963511
EFCAB2	0.120404697	0.022132556	0.038007231
KPNA5	-0.120398901	0.022139017	0.038015048
METTL7B	0.120336242	0.022208968	0.038131873
CALCR	0.120302147	0.022247112	0.038194071
TMEM185B	-0.12027122	0.022281759	0.03824982
CROT	-0.120269734	0.022283425	0.03824982
GAGE2C	0.120256032	0.022298792	0.038272898
TWISTNB	-0.120248213	0.022307565	0.038284657
CEACAM19	0.120241239	0.022315391	0.038294789
IQCG	-0.120224358	0.022334348	0.038324017
FLJ39582	0.120139702	0.022429624	0.038484187
ST8SIA2	-0.120088579	0.022487329	0.038579873
FANCC	-0.120068065	0.022510521	0.038616334
ST6GALNAC	0.119995087	0.022593192	0.038754816
CTSZ	0.119993191	0.022595344	0.038755169
CDADC1	-0.119973181	0.022618058	0.038790788
OR5K1	-0.119968161	0.022623761	0.038797226
TMC3	-0.119949155	0.022645359	0.038830922
LOC440040	0.119927549	0.022669935	0.038869716
PAPLN	-0.119911286	0.022688448	0.03889811
C8orf42	-0.119900524	0.022700706	0.038914636
MAGEA11	0.119899392	0.022701996	0.038914636
DLX6AS	0.119865535	0.022740599	0.038977453
P4HA1	0.119851756	0.022756326	0.039001053
VWA1	0.119843711	0.022765513	0.03901344
LOC728613	0.11977183	0.022847736	0.039150978
UNC5A	-0.119728655	0.022897247	0.039232442
C1orf107	-0.119715381	0.022912488	0.039255179
ZNF648	-0.119700837	0.022929197	0.039280427
CLEC12B	-0.119691357	0.022940093	0.039295713
RORC	-0.119681491	0.022951439	0.039311767
FUK	0.119549674	0.023103486	0.039565998
OR52D1	-0.119549366	0.023103841	0.039565998
AQP9	-0.11953047	0.023125709	0.039600043
STYK1	-0.119515574	0.02314296	0.039626176
UGT2B7	-0.119501428	0.023159353	0.039650835
COCH	0.11948281	0.023180942	0.039684386
C14orf115	0.119473246	0.02319204	0.039699972
NXF1	-0.119469788	0.023196053	0.03970343
IL1A	0.11945854	0.023209114	0.03972237

OTUD7B	-0.119456363	0.023211642	0.039723284
LINGO2	-0.119452934	0.023215624	0.039726685
SLC39A13	0.119446151	0.023223505	0.039736756
LOC286359	0.119440181	0.023230443	0.039745212
ZNHIT6	-0.119410616	0.023264826	0.03980062
ST7OT1	0.119367418	0.023315145	0.039883276
SPRR2G	-0.119334555	0.023353487	0.039945245
PCDHGA8	0.119332933	0.023355382	0.039945245
VNN2	0.119324817	0.02336486	0.039958024
ZNF557	-0.119309947	0.023382235	0.039984304
TRHR	-0.11930762	0.023384955	0.039985523
C22orf28	0.119277631	0.023420032	0.040042062
HES1	-0.119272642	0.023425873	0.04004861
CCL13	0.119268992	0.023430147	0.040052478
ATG10	0.119265487	0.023434251	0.040056056
LOC255025	0.119249049	0.023453507	0.04008553
FBXO22	-0.11922794	0.023478256	0.040124385
GMEB1	-0.119219541	0.02348811	0.040137781
PSORS1C2	-0.1192139	0.023494729	0.040145648
HAS1	-0.119173349	0.023542365	0.040223592
S100PBP	-0.119151154	0.023568473	0.040264746
PLA2G7	0.119083426	0.023648295	0.040397649
PCDHA8	-0.119033588	0.023707182	0.040494771
C14orf72	0.11902636	0.023715733	0.040505903
BRPF3	-0.119000037	0.023746897	0.040555651
CUL4B	-0.118976075	0.023775295	0.040600669
KLK1	0.118926384	0.023834281	0.040697909
ZNF234	-0.118907831	0.023856337	0.040732077
C1orf53	0.118894849	0.02387178	0.04075495
GAGE12F	0.118875426	0.023894902	0.040790929
NSUN2	-0.118865982	0.023906152	0.040803553
CLEC4G	-0.118865777	0.023906395	0.040803553
GAL3ST4	-0.118853143	0.023921453	0.040825754
EPYC	-0.118806709	0.023976861	0.04091681
LGSN	-0.11878896	0.023998069	0.040949493
FSTL5	0.118781754	0.024006684	0.040960684
LOC339524	-0.118735936	0.024061524	0.041050737
CT45A4	0.118691819	0.024114431	0.041137476
SNAR-B2	0.118686844	0.024120403	0.041144139
S100A3	0.118681448	0.024126882	0.041151666
TTLL6	0.118554033	0.024280315	0.04140982
RUFY3	-0.118526133	0.024314024	0.04146376
ADAMTS16	-0.118514969	0.024327524	0.04148323
MS4A1	-0.11842497	0.02443659	0.041665641
MIR17HG	0.118408575	0.024456504	0.041696026
MLEC	0.118385504	0.02448455	0.041740269

SNCB	0.118351322	0.024526154	0.041807615
FOXR1	0.118346495	0.024532035	0.041814061
PASD1	0.118338695	0.02454154	0.04182668
C19orf40	0.118325512	0.02455761	0.041850489
TGM4	0.118276095	0.024617933	0.041949699
VBP1	0.118272773	0.024621992	0.041953025
ZBED2	0.118269339	0.024626189	0.041956587
VN1R1	-0.118257972	0.024640087	0.041976675
MTCH2	0.118236585	0.024666253	0.042017657
CLRN1OS	-0.118230272	0.024673982	0.042027228
BPIL3	0.118226627	0.024678445	0.042031235
CACNG6	0.118193225	0.024719377	0.042095277
CYP2B6	-0.118192493	0.024720275	0.042095277
OR10J3	-0.118190323	0.024722936	0.042096209
ANKRD27	-0.118165096	0.024753892	0.042145316
SLC24A6	-0.118157734	0.024762932	0.042157104
KIR3DL1	-0.118138895	0.024786079	0.042192903
N4BP2L2	-0.11813638	0.024789171	0.042194559
SHC2	0.118121519	0.024807446	0.042222057
MGC23270	0.118114112	0.024816558	0.042233956
RASD2	-0.118105965	0.024826585	0.04224741
NPC1	-0.118103096	0.024830116	0.042249808
C16orf72	-0.118091686	0.024844166	0.042270104
GSG1	-0.118089522	0.024846831	0.042271027
STK3	0.1180633	0.024879149	0.042322392
ESPL1	0.118040668	0.02490707	0.042366272
ARHGAP30	-0.117974066	0.024989397	0.042500886
RIN1	0.117973193	0.024990478	0.042500886
BTNL2	0.117957839	0.025009492	0.04252959
SSX8	0.117915683	0.025061758	0.042614832
STMN2	-0.117901915	0.025078848	0.042640251
HTATSF1	-0.117877782	0.025108829	0.042686671
ZNF716	0.117876488	0.025110437	0.042686671
TMEM189-	-0.117871665	0.025116433	0.04269322
NR2E3	-0.11785889	0.02513232	0.042716579
SLC46A2	-0.117852003	0.025140888	0.042727495
FABP9	0.117807547	0.025196258	0.042817944
NPHS1	0.117756951	0.025259404	0.042921589
TNNT2	0.117719274	0.025306515	0.042997973
SP7	0.117715521	0.025311211	0.043002284
HLA-C	0.117704705	0.025324751	0.043021618
PFKFB1	-0.117674536	0.025362552	0.043082159
RUNX3	-0.11766992	0.025368341	0.043088316
MAL2	0.117665128	0.02537435	0.043094847
IL11RA	0.117630313	0.02541805	0.043165385
LOC550643	0.117528175	0.02554663	0.043380042

SAMD5	-0.117505287	0.025575518	0.043425394
PHYH	-0.117500173	0.025581978	0.04343266
KIAA1529	0.117491223	0.025593284	0.043448151
GZMM	0.117468847	0.025621572	0.043492466
RNF20	-0.117460877	0.025631655	0.043505872
ZUFSP	-0.117456731	0.025636901	0.043511069
ZNF79	-0.117454856	0.025639274	0.043511388
NUAK2	-0.117449826	0.02564564	0.043518483
SLC16A8	-0.117431532	0.025668806	0.043554084
CD53	-0.117428735	0.025672349	0.043555693
MYH16	-0.11742733	0.025674129	0.043555693
ZFP37	-0.117418549	0.025685257	0.04357086
PDK1	-0.117409495	0.025696735	0.043586618
MUC12	0.117393405	0.025717144	0.04361752
ATP2B1	-0.117368876	0.025748285	0.043666618
KRT222	-0.117360057	0.025759489	0.043681899
RIT2	-0.11735335	0.025768012	0.043692631
RAB40B	0.117347363	0.025775622	0.043701815
CELF3	0.117282146	0.025858651	0.043838539
FER1L5	0.117279469	0.025862064	0.043838539
C10orf120	0.117278839	0.025862867	0.043838539
TFF2	0.117259289	0.025887805	0.043877075
PM20D1	-0.117253172	0.025895612	0.043886572
MAPKAP1	0.117240766	0.025911451	0.043909679
CDAN1	0.117221604	0.025935934	0.043947428
KLKB1	-0.117212279	0.025947855	0.043963887
LCAT	-0.117209433	0.025951493	0.043966311
SYCP2	-0.117204114	0.025958297	0.043974096
PHC2	0.117202132	0.025960832	0.043974651
LIF	-0.117196663	0.025967828	0.043982761
TBC1D17	0.117187444	0.025979626	0.043994311
CNTNAP3	-0.117187223	0.025979909	0.043994311
CABP2	-0.117186156	0.025981275	0.043994311
GPR18	-0.117113122	0.02607491	0.04414911
ERBB3	0.117105757	0.026084368	0.044161369
XYLT2	0.117082929	0.026113703	0.044207277
FSD1L	-0.117016138	0.026199697	0.044349084
NRTN	0.116996174	0.026225448	0.04438613
RGL3	-0.116995716	0.02622604	0.04438613
B3GNT6	-0.116980283	0.026245961	0.04441607
ADAMTS13	-0.11697043	0.026258688	0.044433832
FMO6P	0.116959943	0.026272238	0.044452983
KLRD1	-0.116952302	0.026282114	0.044465917
MRS2	0.116947932	0.026287764	0.044471698
OR5M11	0.116914621	0.026330868	0.044540833
TTLL2	-0.116878812	0.026377272	0.044615541

MESDC1	-0.116786154	0.026497671	0.044815381
WNT7B	-0.116770772	0.026517704	0.044845455
GALNT5	-0.116758794	0.026533313	0.044868042
C13orf30	-0.116751165	0.026543259	0.04488105
MFAP1	0.116739671	0.026558249	0.044902583
KIAA1609	-0.116715621	0.026589638	0.044951836
ST3GAL5	-0.11667399	0.026644049	0.045039999
SMNDC1	-0.116669802	0.026649527	0.045045437
ZNF667	-0.11665287	0.026671688	0.045079069
C18orf32	0.116637075	0.026692376	0.045110206
CLCN7	0.116613251	0.026723604	0.045159149
LOC100271	0.116582045	0.026764558	0.045224518
GRAMD1B	-0.116525811	0.026838493	0.0453456
EGFL7	0.116498652	0.026874263	0.045402184
TPRXL	0.116486736	0.026889971	0.045424868
OR1C1	0.116476278	0.026903763	0.045443808
DLK1	0.116474774	0.026905746	0.045443808
GCGR	-0.116465047	0.02691858	0.045461629
GIMAP1	-0.116461642	0.026923075	0.045465364
GRAMD2	-0.116448196	0.026940827	0.045491485
CXorf49B	0.11642824	0.026967192	0.045531904
TMEM220	-0.116426618	0.026969337	0.045531904
C10orf58	0.116405786	0.026996885	0.04557455
APOO	0.116385503	0.027023732	0.045616004
HS2ST1	-0.116377104	0.027034855	0.045630912
ASCL1	-0.116357281	0.027061124	0.04567138
DMBT1	0.116349616	0.027071286	0.04568466
NPNT	-0.116345601	0.027076612	0.045689775
TMEM175	-0.116340295	0.027083651	0.04569778
C2orf54	0.116337955	0.027086755	0.04569834
MAGEA10	0.116336585	0.027088572	0.04569834
C1orf88	0.116322755	0.027106928	0.045722234
ADSSL1	-0.116322455	0.027107327	0.045722234
SRMS	-0.116303782	0.027132127	0.045760189
MS4A10	-0.116286456	0.027155157	0.045795153
CCDC114	0.116275688	0.027169478	0.045815424
ZC3H12A	-0.116239055	0.027218248	0.045893777
GUCA2B	0.116186909	0.0272878	0.046007157
LAMB2	-0.116168154	0.027312853	0.046045497
EZH1	-0.116151244	0.027335457	0.046079704
ALDH5A1	-0.116142365	0.027347332	0.046095821
C7orf51	-0.116130576	0.027363107	0.046116614
CCL8	0.116129685	0.027364299	0.046116614
SMG7	-0.116078844	0.027432421	0.046227506
PLSCR5	0.116043066	0.027480447	0.046304519
MCHR1	0.116036137	0.027489757	0.046316287

PLXND1	-0.11602426	0.02750572	0.046339262
FAM105A	-0.115938857	0.027620743	0.046529107
GPR108	0.115920502	0.027645517	0.046566902
USP27X	0.115859338	0.027728212	0.046702245
KIFAP3	-0.115838436	0.02775652	0.04674597
CASC3	0.115821113	0.02778	0.046781558
CFD	0.115800603	0.027807822	0.046821076
TPSG1	-0.115800347	0.027808169	0.046821076
EHD1	0.115776365	0.027840731	0.046871939
GAGE2E	0.115755994	0.027868417	0.046914583
RBPMS2	-0.115695151	0.027951245	0.047050042
TLR7	-0.115675863	0.027977547	0.047090336
ZCCHC7	0.115672337	0.027982357	0.047094451
FIGN	-0.115596147	0.028086476	0.04726569
SLC14A2	-0.115586498	0.028099686	0.047283924
TRPV6	-0.115578213	0.028111032	0.04729902
DDAH1	-0.115574196	0.028116535	0.047304283
SELV	0.115553973	0.028144253	0.047346916
SPAG1	-0.115542153	0.028160463	0.047370186
USP21	0.115528657	0.028178983	0.047394274
RCOR2	0.115527414	0.028180689	0.047394274
GPRIN1	0.115526515	0.028181923	0.047394274
DFFB	-0.115523472	0.0281861	0.047397296
ANO4	-0.115516634	0.02819549	0.047409084
CLDN11	-0.115511687	0.028202284	0.047416503
CCK	0.115505382	0.028210946	0.047427063
OR4K2	-0.115493345	0.028227488	0.047448154
CNIH	-0.115492786	0.028228257	0.047448154
ZNF467	-0.11548286	0.028241904	0.047467088
ACTN4	-0.115436193	0.028306145	0.047571044
LOC349114	-0.115429568	0.028315276	0.047582374
AURKC	0.115409216	0.028343339	0.047625514
CCL20	0.115396109	0.028361425	0.047651884
RBP5	0.11537599	0.028389206	0.047694536
ANGEL1	-0.115369664	0.028397945	0.047705194
STK32A	-0.115329397	0.028453631	0.047794708
ASB5	-0.115298891	0.02849588	0.047861639
DKC1	0.115296993	0.028498512	0.047862022
KNDC1	-0.115283245	0.028517571	0.047889992
ABCA17P	0.115256729	0.028554362	0.047947734
RASL10A	0.115253893	0.0285583	0.047950303
FAM70A	-0.115247186	0.028567614	0.047961897
ATRNL1	-0.115237605	0.028580922	0.047980196
PPPDE2	0.115210788	0.028618202	0.048038731
PIGV	-0.11520175	0.028630777	0.048055787
RBM4B	0.115188401	0.028649356	0.048082375

MTAP	-0.1151869	0.028651446	0.048082375
TIMD4	-0.115174552	0.028668643	0.048105911
LOC401387	0.115173361	0.028670302	0.048105911
QRICH2	0.115160218	0.028688617	0.04813166
SNORA4	-0.11515888	0.028690481	0.04813166
ZNF815	0.115128876	0.028732331	0.048197808
C20orf85	0.115082364	0.028797311	0.048302003
OR5B2	-0.115080945	0.028799296	0.048302003
PCDH11Y	-0.115069265	0.028815635	0.048324938
TDRD5	0.1150677	0.028817824	0.048324938
TERF2	-0.115064329	0.028822541	0.048328779
FBXW8	-0.11498595	0.028932408	0.048508917
TLE3	-0.114928456	0.029013229	0.048636381
XPNPEP3	-0.114928393	0.029013317	0.048636381
RTN4RL2	-0.114874942	0.029088627	0.048758523
SYNGR1	0.114868374	0.029097893	0.048769949
DNAJC4	0.114865958	0.029101302	0.048771559
C20orf56	-0.114848694	0.029125672	0.048808293
RHBDL1	0.114815981	0.029171896	0.048881642
CD300LF	0.114810879	0.02917911	0.048889616
PGM1	-0.114803414	0.029189669	0.048903194
GNPNAT1	-0.11480014	0.029194302	0.048906841
CCL21	-0.114793291	0.029203994	0.048918962
MLLT3	-0.11477368	0.029231761	0.048961356
VIP	-0.114766793	0.029241518	0.04897358
PAG1	-0.114742	0.029276665	0.049028319
ADAMTS6	-0.114728649	0.029295607	0.049055916
BAAT	-0.114711068	0.029320565	0.049093581
NCRNA0009	-0.114688884	0.029352084	0.049142223
KCNK4	-0.114565664	0.029527685	0.049432066
TMEM125	-0.114560561	0.029534977	0.049440116
C8orf51	0.114537912	0.029567358	0.049490161
NUS1	-0.114533822	0.029573209	0.049495795
RFPL3	-0.114526827	0.029583218	0.049508385
CBLC	0.11449513	0.029628608	0.04958018
RAB3IL1	0.114486277	0.029641295	0.049597244
ODF3L2	0.114474804	0.029657745	0.049618067
MED28	0.114474123	0.029658723	0.049618067
GSPT1	-0.114449621	0.029693881	0.049672636
FEZ2	-0.114446479	0.029698391	0.049672636
SYT6	-0.114446178	0.029698824	0.049672636
TGS1	-0.114438143	0.029710363	0.049687762
LOC400931	0.11443545	0.029714231	0.04969006
MAGEB2	0.114387306	0.029783458	0.049801642
IL13RA2	-0.114384756	0.029787128	0.049803598
MRPS35	0.114359046	0.029824156	0.049861018

IMPDH1	0.114357435	0.029826478	0.049861018
SYT15	-0.114315151	0.029887468	0.049958781
CCDC66	-0.114301383	0.029907349	0.049987819
C7orf43	-0.114298545	0.029911449	0.049990476
NINJ1	0.114281285	0.029936393	0.050027966
GATA4	-0.114275651	0.029944539	0.050037379
LOC399744	-0.114202801	0.030050038	0.050209456
C16orf87	-0.114136642	0.030146124	0.050365777
KIAA1324L	-0.114123778	0.030164838	0.050392815
FAM55D	-0.114067388	0.030246985	0.05052581
GAS2L1	-0.114010331	0.030330301	0.050660736
LOC221710	0.114006022	0.030336601	0.050667009
ZNF367	-0.113966719	0.030394115	0.050758809
CLEC5A	-0.113961165	0.030402251	0.050768139
KLC1	-0.113955386	0.030410718	0.05077802
CFH	-0.113944973	0.030425978	0.050799242
RUSC1	0.113938274	0.0304358	0.05081138
MKL1	-0.113926652	0.030452844	0.050835574
MMP20	0.113905738	0.030483539	0.050882548
KCNT1	-0.113870479	0.030535344	0.050964749
ZBTB40	-0.113839163	0.030581421	0.051037375
C9orf173	-0.113836997	0.03058461	0.05103842
PASK	-0.11381767	0.030613078	0.051081647
ZNF334	-0.113768274	0.030685942	0.051198938
SLC25A44	-0.113733815	0.030736859	0.051279597
CHST7	-0.113723335	0.030752358	0.051301157
FKBP1AP1	-0.113672239	0.030828025	0.051423077
IGSF1	0.113659292	0.030847223	0.051450792
C1orf216	0.113652981	0.030856584	0.051462095
ZNF321	0.113631999	0.030887727	0.051509721
HSPA9	-0.113598739	0.030937147	0.051587816
DHX57	0.113585107	0.030957422	0.051617303
ZIC2	0.113582564	0.030961207	0.051619291
SNHG3-RC	-0.113579565	0.030965669	0.051622409
PPP2R4	0.113548117	0.031012497	0.051696147
ASMTL	-0.113542645	0.031020651	0.051705412
C15orf51	-0.113532322	0.031036039	0.051726731
SERPINA4	0.113505076	0.031076685	0.05179014
LOC646762	-0.113485699	0.031105618	0.05183402
HOMER1	-0.113461856	0.031141252	0.051889059
RSPH6A	0.113448349	0.031161455	0.051918378
FAM127B	0.113424601	0.031197002	0.051973256
HCG4P6	0.113416624	0.031208951	0.051988813
FLJ44606	0.113397722	0.031237277	0.052030258
MRGPRE	-0.113395929	0.031239967	0.052030258
C15orf32	-0.113394793	0.031241669	0.052030258

SSX3	0.11338411	0.031257691	0.05205259
NCS1	-0.113362003	0.031290868	0.052103482
GPER	0.113356094	0.031299741	0.052113899
44089	0.113344022	0.031317876	0.052139734
LOC100128	-0.113338589	0.03132604	0.052148966
ARID5B	-0.113330433	0.031338299	0.052165014
LOC389634	0.113322281	0.031350557	0.052181056
RFC1	-0.113311856	0.031366237	0.052202792
DYDC1	0.113306836	0.031373791	0.052211
ADRA1D	0.113296493	0.031389358	0.052232541
KCNJ6	-0.113279505	0.031414942	0.052270747
DGKD	-0.113277579	0.031417844	0.052271208
ITGB6	-0.113256407	0.031449755	0.052317044
OTC	-0.113255815	0.031450648	0.052317044
SPIN2A	-0.113251111	0.031457743	0.052324475
TDRKH	0.113215416	0.031511619	0.052409713
GPR150	-0.113200439	0.031534249	0.052442969
PRLHR	0.113194771	0.031542816	0.052452838
GFAP	-0.113190901	0.031548666	0.052458186
LPA	-0.113188276	0.031552636	0.052460406
LOC285456	-0.113171706	0.031577701	0.052497697
GALNS	-0.113161006	0.031593895	0.052520236
KYNU	0.113144898	0.031618288	0.052556397
METRNL	-0.113139452	0.031626538	0.052565724
KRT7	-0.113136218	0.03163144	0.052569484
CNTN1	-0.113123289	0.031651037	0.052597663
GEMIN4	0.113114648	0.03166414	0.052612712
SLC38A9	0.113113833	0.031665376	0.052612712
ZNF687	-0.113085812	0.031707902	0.052678974
KIAA2013	0.113076482	0.031722072	0.052698121
BPIL2	-0.113071922	0.031728999	0.052705232
SLC26A9	-0.113068741	0.031733834	0.052708865
PGLYRP2	-0.11305691	0.031751816	0.052729963
TAGLN2	0.1130569	0.031751831	0.052729963
LRRC66	-0.112967787	0.031887555	0.052950943
ABCA11P	0.112960115	0.031899264	0.05296597
TNR	-0.112919864	0.03196075	0.05306344
LTA	0.1129182	0.031963295	0.05306344
SLC44A3	0.112886566	0.032011693	0.053139356
OXCT2	0.112883892	0.032015786	0.053141722
AKAP1	-0.112867784	0.032040456	0.053178238
SPATA22	-0.11286585	0.03204342	0.053178726
TATDN2	0.112863796	0.032046567	0.053179517
FLT3LG	0.112840905	0.03208166	0.053233316
OR3A1	0.112835255	0.032090327	0.053243261
ALOX15	-0.112801081	0.032142791	0.053325864

GATSL3	-0.112767688	0.032194126	0.053406583
MMD2	0.112753562	0.032215862	0.053438189
MARVELD3	-0.112707291	0.032287152	0.05355198
SENP5	-0.112701147	0.032296628	0.053563236
TBX18	-0.112690699	0.032312749	0.05358551
PABPC3	-0.112667268	0.032348923	0.053641033
IQGAP3	0.112658211	0.032362916	0.053659768
C8orf75	-0.112646651	0.032380783	0.053684923
ADHFE1	-0.112634479	0.032399606	0.053711659
MDGA1	-0.112631246	0.032404606	0.053715477
FLJ37453	0.112618242	0.032424729	0.053744359
RPL29P2	0.112600184	0.032452688	0.053786225
LOC100130	0.112586774	0.032473464	0.05381618
SCN3B	-0.112571602	0.032496983	0.053850676
FAM23A	-0.112563207	0.032510004	0.05386777
TREML2	-0.112546968	0.032535203	0.053905039
CD207	-0.112527697	0.032565127	0.05395013
CDKL4	0.112517732	0.03258061	0.053971291
PPAT	-0.112506823	0.032597568	0.053994891
ADRBK1	-0.112500969	0.032606671	0.054005477
ZNF547	0.112492994	0.032619076	0.054021529
FOXD1	0.11249021	0.032623407	0.05402421
44075	0.112448067	0.032689031	0.054125611
C10orf91	0.112447397	0.032690076	0.054125611
PCMT1	0.11243252	0.032713269	0.054159511
DIRC1	-0.112393328	0.032774437	0.054256268
PPY	0.112384528	0.032788184	0.054271045
RNASE13	-0.112384126	0.032788813	0.054271045
ZNF845	-0.11237444	0.032803951	0.054291588
CPE	-0.112364591	0.03281935	0.05431256
MED31	0.112346105	0.032848268	0.0543559
CNGA1	-0.112333208	0.032868457	0.054384789
LAMP1	-0.112329932	0.032873587	0.054388759
C3orf38	-0.112309817	0.0329051	0.05443546
CRISP1	0.112308426	0.032907281	0.05443546
IL19	0.112297958	0.032923692	0.054458083
44086	0.112294623	0.032928921	0.054462209
CHRNA3	-0.112269508	0.032968328	0.054522857
SCRT1	0.112266307	0.032973353	0.05452664
PHC1	-0.112248594	0.033001174	0.054568114
C6orf105	-0.11220962	0.033062458	0.054664911
RELN	0.112198449	0.033080041	0.054689442
IFIT1	-0.112170178	0.033124576	0.054758523
PCDHA7	-0.11215421	0.033149753	0.054795595
C10orf137	-0.112130271	0.033187528	0.054853484
APH1B	-0.11209115	0.033249339	0.054951085

PDZD7	0.112084603	0.033259693	0.054963637
HIF1AN	0.11207863	0.033269141	0.05497308
STL	-0.112077501	0.033270928	0.05497308
DCAF15	0.112052088	0.033311155	0.05503498
IFFO1	0.112049392	0.033315424	0.055037468
NOX1	0.112046481	0.033320035	0.055040519
CALCA	0.112017804	0.033365489	0.055111032
BEGAIN	-0.112004025	0.033387348	0.055142565
POLE	-0.111985052	0.033417467	0.055187731
GPAM	-0.111954175	0.033466532	0.055264178
GPR101	-0.111928919	0.033506711	0.055325938
ABHD8	0.11189808	0.033555827	0.055402445
MRGPRF	-0.111888801	0.033570617	0.05542227
POU4F3	-0.111861178	0.03361468	0.055490414
RNF121	0.111852532	0.033628483	0.055508598
C5orf54	0.11183439	0.033657458	0.055551821
CD1C	-0.11182336	0.033675086	0.05557631
HCP5	-0.111813537	0.03369079	0.055597621
RFTN2	-0.111797669	0.033716173	0.055634898
LOC100130	-0.111777832	0.033747929	0.055682685
LRRC27	0.111769906	0.033760623	0.055699015
ANAPC4	0.111744277	0.033801701	0.055759581
OR7E91P	-0.111743508	0.033802934	0.055759581
DSCR10	0.111731548	0.033822119	0.055786607
BCMO1	-0.111712052	0.03385341	0.055833596
ORC5L	0.111707988	0.033859936	0.055839735
STX18	0.111693879	0.033882601	0.055872485
HOXA11AS	0.111650137	0.033952951	0.055983856
INHA	0.111636143	0.033975483	0.056016371
SFRP5	-0.111619766	0.034001869	0.056055233
PNPLA4	-0.111601496	0.034031325	0.05609915
VAV3	-0.1115901	0.03404971	0.056124812
TDP1	0.111567586	0.034086055	0.056180069
LEP	-0.111562816	0.03409376	0.056188118
PAR4	-0.111548673	0.034116612	0.056221128
RTP3	-0.111535753	0.0341375	0.056250895
COMP	-0.111496952	0.034200297	0.056349707
BEND3	0.111470314	0.034243466	0.056416167
UGT2B28	-0.111463477	0.034254552	0.056429763
PTPRO	-0.111448932	0.03427815	0.056463966
RPRM	0.111411766	0.034338508	0.056558713
RPS4Y1	0.111406641	0.034346838	0.056567754
LOC100101	0.111404629	0.03435011	0.056568464
CTDSP1	0.111391742	0.034371066	0.056598295
ULBP1	-0.111386566	0.034379485	0.056607478
PRKCD	0.111380272	0.034389726	0.056617988

CRYGD	0.111379149	0.034391554	0.056617988
CCDC64	-0.111293241	0.034531601	0.056843845
GTF3C1	-0.111207993	0.034671052	0.057064977
KSR2	-0.111207525	0.034671819	0.057064977
DAZAP2	-0.11120587	0.034674531	0.057064977
PIGQ	0.111198256	0.03468701	0.057080797
HOXB7	0.111193753	0.034694393	0.057088228
KIAA1614	-0.111154926	0.034758103	0.057185644
OSM	-0.111154173	0.034759338	0.057185644
HSPB8	-0.111151104	0.03476438	0.057189213
RXRA	-0.111139645	0.034783203	0.057215452
PPPDE1	-0.111124013	0.034808896	0.057252987
VKORC1	0.111114551	0.034824456	0.05727385
LOC338799	0.111094187	0.034857965	0.057324226
ZNF415	-0.111051709	0.034927949	0.057434572
LETM2	-0.11103686	0.034952442	0.057470102
GNG7	-0.11101841	0.034982894	0.057515425
MTCP1NB	0.111013901	0.03499034	0.057522917
HPSE2	0.111009495	0.034997618	0.057530133
MGC12916	0.110986268	0.035036001	0.057588475
ERMN	-0.110976064	0.035052873	0.057611453
RER1	0.110971887	0.035059784	0.057618055
HSD11B1L	0.110961838	0.035076409	0.057640621
CDC42SE2	-0.110950739	0.035094782	0.057664573
AP2A1	-0.110949535	0.035096776	0.057664573
DUSP4	-0.110917296	0.035150189	0.057741588
PEPD	-0.110916047	0.03515226	0.057741588
CCT6B	-0.110915994	0.035152348	0.057741588
ZNF442	-0.110873234	0.035223303	0.057853367
SNORA80	0.110845273	0.035269766	0.057924904
FAM154B	-0.110794278	0.035354639	0.058059506
HEATR6	-0.110759146	0.035413213	0.058150901
JSRP1	0.110734031	0.035455135	0.058214941
NEFL	-0.110716266	0.035484815	0.058258871
PITX1	0.110709672	0.035495838	0.058272163
FBXO45	0.110693224	0.035523342	0.058312509
INTS4	0.110638256	0.035615394	0.058458796
HOXA5	-0.110590316	0.035695842	0.058586014
KIAA1598	-0.110582589	0.035708824	0.058602492
CYP2R1	0.110503768	0.03584147	0.058815332
RUNDC3B	-0.110484079	0.03587467	0.058864962
APOL1	-0.110464889	0.035907052	0.058913243
CRYZ	-0.110453427	0.035926405	0.05894014
ARHGEF2	0.110426013	0.035972732	0.059011281
SYTL3	-0.110412617	0.035995387	0.059043583
BAGE2	-0.110404402	0.036009286	0.059054798

LPPR3	-0.110403968	0.036010021	0.059054798
ACSM2A	-0.110403318	0.036011121	0.059054798
SCN1A	0.110394814	0.036025515	0.059072413
FAM138B	0.110393467	0.036027794	0.059072413
CYP4F12	0.110359869	0.036084712	0.059160866
C7orf33	0.110332739	0.03613073	0.059231436
SENP8	-0.110327394	0.036139801	0.059241431
C21orf58	0.110315877	0.036159356	0.059268607
FCGR1B	0.110295731	0.036193581	0.059319823
SNRNP27	-0.110271251	0.036235206	0.059383158
RFPL3S	0.110264774	0.036246227	0.059396332
NAPB	0.110232876	0.036300541	0.059480441
TMSL3	0.110223492	0.036316534	0.05950175
SPRYD5	-0.110203893	0.036349953	0.059551606
CDSN	-0.110197111	0.036361523	0.059563477
SH3KBP1	-0.110196139	0.036363181	0.059563477
C8G	0.110192248	0.036369821	0.059569453
GABRA2	0.110156662	0.036430595	0.059664088
HMGA2	0.110144142	0.036451998	0.059694231
FABP4	-0.110128953	0.036477977	0.059731863
SSTR1	-0.110115033	0.036501798	0.059765955
TSPY3	0.110080386	0.036561151	0.059858214
TAF8	0.110077062	0.036566849	0.059862622
RASSF10	-0.110073501	0.036572954	0.059867694
ABCG2	-0.110018767	0.036666909	0.06001656
MUC2	-0.110008886	0.036683891	0.060039422
LOC647946	0.109954021	0.036778312	0.060189012
TMEM144	-0.109943074	0.036797176	0.060214935
PTK7	-0.109910592	0.036853197	0.060301653
BAG3	-0.109885127	0.036897169	0.060368643
CYB5B	0.10986612	0.036930017	0.060417422
TMEM206	0.109849514	0.036958735	0.060459438
CNTNAP2	-0.109839059	0.036976826	0.060484065
GDEP	0.109786159	0.037068478	0.060629002
TCAM1P	0.109783586	0.03707294	0.060631322
CYFIP2	-0.10976887	0.037098473	0.060668098
C17orf71	-0.109765321	0.037104634	0.06067319
IER5L	0.10973159	0.037163224	0.060764006
STH	0.10971859	0.037185824	0.060795968
KLK10	-0.10966074	0.037286542	0.06095563
CTAGE9	-0.109645112	0.03731379	0.060995168
TARP	-0.109641279	0.037320476	0.06100109
LOC220930	-0.109625764	0.037347549	0.061040332
TEAD3	0.109610468	0.037374255	0.061078967
WNT4	-0.109606986	0.037380336	0.061083892
TREH	-0.109598233	0.037395628	0.061103867

XKR5	-0.109571525	0.03744232	0.061175142
ADH4	-0.109507212	0.037554959	0.061354143
CNDP2	-0.109501644	0.037564724	0.061365063
KLF14	0.109498747	0.037569806	0.06136833
LOC145837	-0.109462448	0.037633529	0.061467377
C20orf132	-0.109449329	0.037656581	0.061499985
INPP1	-0.109444259	0.037665494	0.061509497
PRAMEF2	-0.109442493	0.037668599	0.061509523
C15orf27	0.109427821	0.0376944	0.061546608
LIMK2	-0.109416815	0.037713766	0.06157318
ATP8B3	-0.109381976	0.037775123	0.061668298
RTN4R	-0.1093496	0.037832217	0.061751451
RDH11	-0.109349576	0.03783226	0.061751451
SLC12A5	0.109331882	0.037863492	0.061795319
LPIN3	-0.109330447	0.037866028	0.061795319
GDAP1	0.109329078	0.037868444	0.061795319
tAKR	-0.109316749	0.037890224	0.061825794
TPTE	0.109309161	0.037903633	0.061842606
TOP3B	0.109288054	0.037940953	0.061898426
SCEL	-0.109268669	0.037975256	0.061949314
TMEM30C	0.109253647	0.038001857	0.061987631
TSPAN9	-0.109197659	0.038101139	0.062144486
HOXC13	0.109172347	0.038146094	0.062211466
ZNF491	-0.109171021	0.038148452	0.062211466
AMACR	-0.109169179	0.038151725	0.062211709
CT62	-0.109156394	0.038174453	0.062243674
ATP6V0A2	-0.109151163	0.038183755	0.062253744
KCNT2	-0.109141987	0.038200077	0.062275257
B3GALT2	-0.109131342	0.03821902	0.062301038
ITIH1	0.10911215	0.038253191	0.062351637
PLD5	-0.109069131	0.038329882	0.062471529
DNAJB3	-0.109042012	0.038378296	0.062545317
TMEM119	-0.109039447	0.038382877	0.062547663
CRAT	-0.109035373	0.038390156	0.062554406
GPR132	-0.109021695	0.038414599	0.062589114
C21orf29	-0.10901383	0.038428661	0.062606903
HDAC8	0.109003441	0.038447242	0.062628273
TMEM229B	-0.10900298	0.038448067	0.062628273
ZIK1	-0.108979628	0.03848986	0.062687167
SOST	-0.10897926	0.038490518	0.062687167
C2orf66	-0.108965266	0.038515583	0.062722859
PPM1J	-0.108951063	0.038541035	0.062759176
PCK2	-0.108922451	0.038592353	0.062837601
DTX3L	-0.108886387	0.038657119	0.062937911
RNF212	-0.108872178	0.038682662	0.062974349
PIGX	0.108863885	0.038697575	0.062993478

PTGFRN	-0.108854274	0.038714867	0.063016475
BOLL	0.108793169	0.03882495	0.063190494
CACNB3	-0.108774303	0.038858992	0.063240731
TNFRSF1B	-0.10876822	0.038869973	0.063253433
LRFN4	-0.108737644	0.038925211	0.063338147
ALDH3B2	-0.108716919	0.03896269	0.063393952
LOC100128	-0.108704418	0.038985313	0.063425578
C22orf29	0.108665317	0.03905614	0.063535617
C3orf62	0.10861157	0.039153676	0.063687387
GUCY1B2	0.108610384	0.039155831	0.063687387
PGF	0.108593078	0.039187281	0.063733337
CYBB	-0.10858445	0.03920297	0.063753646
LINGO3	-0.108579211	0.039212498	0.063763933
ADAMTSL4	-0.108550821	0.039264166	0.063842739
TTL	-0.108543624	0.039277273	0.063858836
SIRPG	0.108524931	0.039311334	0.063908998
DVWA	0.108511701	0.039335457	0.063942995
KLC3	0.10848527	0.039383685	0.064016168
ANAPC10	-0.108477103	0.039398598	0.064035181
CES3	-0.108474773	0.039402854	0.064036872
DAK	-0.108451266	0.039445807	0.064101449
NKAPL	-0.108441295	0.039464037	0.064125841
NLGN4Y	-0.108430977	0.039482912	0.064151276
BIN2	-0.108400475	0.03953875	0.06423676
CYP26C1	-0.108359248	0.039614327	0.064354296
DEDD	0.108354888	0.039622328	0.064358368
GCNT3	0.10835436	0.039623297	0.064358368
DNAH12	0.108343201	0.039643777	0.064386383
SNORA65	0.108305733	0.039712612	0.06449292
HES7	0.108303606	0.039716524	0.064494013
ODC1	0.108300491	0.039722252	0.064498055
FAM150A	-0.108278848	0.039762068	0.06455209
PLEKHM2	-0.108277457	0.039764629	0.06455209
CHIC2	-0.108277046	0.039765384	0.06455209
TOX3	0.108275356	0.039768496	0.06455209
TMEM74	0.108256889	0.0398025	0.064602019
FAM90A1	0.108196524	0.039913826	0.064777431
SERPINA1	0.108191212	0.039923635	0.064785954
GPX5	0.108190157	0.039925584	0.064785954
HAR1A	-0.108186567	0.039932215	0.064791434
TPTE2P3	0.108168938	0.039964788	0.064839003
HOXC10	0.108164039	0.039973846	0.064842446
CA3	-0.108162712	0.039976298	0.064842446
CRB3	0.108162507	0.039976678	0.064842446
KIAA0406	-0.108159014	0.039983136	0.06484562
ESPN	0.108157926	0.039985148	0.06484562

KAZALD1	0.108141838	0.040014908	0.064888599
MCM3AP	-0.108121347	0.040052838	0.064944819
DHX8	-0.10811257	0.040069094	0.064965888
SLC25A31	0.108099962	0.040092456	0.064998475
PPAP2C	0.10808793	0.040114761	0.065027466
MEGF11	-0.108086793	0.040116869	0.065027466
TESK1	0.108069635	0.040148696	0.065073759
ZNF222	0.108050359	0.040184478	0.065126455
DPP4	-0.108030087	0.040222138	0.065182186
OR1L6	0.108027641	0.040226684	0.065184248
RHCG	-0.108011784	0.040256164	0.065226712
COQ9	-0.107994403	0.040288501	0.065273795
FADS3	0.107985205	0.04030562	0.065296219
LOC732275	0.107978464	0.040318172	0.065311241
LIPC	0.107961844	0.040349132	0.065356077
CD40LG	-0.107937544	0.040394434	0.065419846
C14orf129	-0.107937202	0.040395071	0.065419846
GPR143	-0.107923084	0.040421412	0.065457182
PPFIA4	0.107908124	0.040449339	0.06549708
FAM100A	0.107870942	0.040518819	0.065604251
WDR59	-0.107848379	0.04056103	0.065667255
MYOM1	0.107820455	0.040613323	0.065746571
ZNF501	-0.10781657	0.040620603	0.065753012
PRL	0.107785077	0.040679657	0.065843251
UTF1	0.107775234	0.040698128	0.065867794
PCDH20	-0.10776322	0.040720683	0.065898943
KIAA1524	0.107758127	0.040730248	0.065907935
KRTAP19-3	0.107756738	0.040732858	0.065907935
ZFYVE21	-0.107746602	0.040751901	0.06593339
C6orf182	-0.107708373	0.040823788	0.066044333
CAMK2N1	0.107696343	0.040846433	0.066075601
CD27	0.107665615	0.040904321	0.066163869
RFX2	-0.107639724	0.04095315	0.066237471
WFDC5	-0.107548528	0.041125535	0.066507628
MSX1	0.107547828	0.041126861	0.066507628
CD44	-0.107517336	0.041184636	0.06659565
PLAC9	0.107492168	0.041232377	0.066667434
AMZ1	-0.107452466	0.041307779	0.06677944
FBXO32	0.107452162	0.041308357	0.06677944
SLC2A7	0.107437241	0.041336726	0.066819878
TMEM179	-0.107377868	0.041449773	0.066997177
CXorf27	0.107367972	0.04146864	0.067022234
ZNF626	-0.107363047	0.041478031	0.067031972
NCOA5	0.10735515	0.041493096	0.067050877
MLXIPL	0.10735335	0.04149653	0.067050986
ZPBP2	0.107347635	0.041507436	0.067063166

OXER1	0.107329687	0.041541701	0.067113082
LRRC3B	-0.107302086	0.04159444	0.067192835
FOXRED1	0.107283074	0.041630801	0.067246119
OR2AT4	0.107235289	0.041722307	0.067388463
ENPP5	-0.107230357	0.041731763	0.067397844
GATA3	-0.107228729	0.041734884	0.067397844
CDH23	-0.107200291	0.041789437	0.06748047
BRAF	-0.107189421	0.041810305	0.067508693
NUP62	0.107182725	0.041823162	0.067523979
CYTSB	-0.107149471	0.041887073	0.067621682
SMARCC2	-0.107138269	0.041908623	0.067650986
SLC19A3	-0.107131604	0.041921448	0.067666204
TNFAIP8	-0.107108161	0.041966583	0.067733568
DLGAP4	0.107099021	0.041984192	0.067756497
KLC2	-0.107071436	0.042037373	0.067836827
SETD1A	-0.107058659	0.042062025	0.067871108
GZMB	0.107025934	0.042125221	0.067967574
JUB	-0.107013464	0.042149324	0.06799777
NEB	-0.107012719	0.042150764	0.06799777
VAC14	0.106986416	0.042201642	0.068074332
AKR1B15	0.106980989	0.042212147	0.068085762
TTBK1	-0.106976914	0.042220035	0.06809075
B4GALNT1	0.106974842	0.042224047	0.06809075
DEF8	0.106974093	0.042225496	0.06809075
TEX10	0.10696917	0.042235029	0.06809535
SERPINB11	0.106969088	0.042235188	0.06809535
C2orf77	-0.106962799	0.042247369	0.068109476
DNAJA4	-0.10694769	0.042276644	0.068151154
DSCR4	0.106919223	0.04233185	0.068234625
KCNIP3	-0.106915882	0.042338334	0.068239553
SMYD2	0.106912945	0.042344033	0.068243216
FAM83D	0.106890098	0.042388393	0.06830918
RPL21P44	-0.106861858	0.04244328	0.068392097
PLEKHG7	-0.106851005	0.042464389	0.068420576
BMPR1B	-0.106819835	0.042525066	0.068512798
INE2	0.1068166	0.042531368	0.068517407
LOC645676	0.106778545	0.042605553	0.068631368
DDX19A	-0.106756808	0.042647978	0.068694152
RFPL4A	-0.106744658	0.042671706	0.068726812
ANXA13	0.106735542	0.042689518	0.068749939
LOC388428	0.106670935	0.042815925	0.068947937
CCDC148	-0.106659532	0.042838268	0.068978339
PRRT1	-0.106648733	0.042859437	0.069006846
TMEM191A	0.10664188	0.042872876	0.069022905
CAPN14	-0.106636619	0.042883194	0.069033935
TMEM8A	-0.106621161	0.042913526	0.069073038

KRT86	-0.106619855	0.042916089	0.069073038
NR5A2	-0.106618938	0.042917889	0.069073038
SLC35F2	-0.106573039	0.043008065	0.069212574
PRSS21	0.106555328	0.043042905	0.069263046
FTSJD2	0.106496887	0.04315803	0.069437463
SNORD15A	0.106496006	0.043159768	0.069437463
PELO	0.106494998	0.043161755	0.069437463
C3orf57	0.106491728	0.043168205	0.06944223
EXOC2	-0.106457209	0.043236341	0.069546218
NLRP5	0.106434615	0.043280988	0.069612412
C9orf110	-0.106397812	0.043353796	0.069723883
FLJ13224	-0.106374298	0.043400366	0.069793144
GAL3ST2	-0.106365442	0.043417917	0.069814979
TMCO1	0.106363909	0.043420955	0.069814979
DMWD	0.106344376	0.04345969	0.069871617
PSMA8	0.106338796	0.043470761	0.069883775
VSX2	0.106325117	0.043497911	0.069917052
NPC1L1	0.106324829	0.043498482	0.069917052
BRDT	0.106322338	0.043503427	0.069919356
GPR37L1	0.106315958	0.043516095	0.069934072
CHRDL2	0.106274969	0.04359756	0.070059341
CCDC103	0.106267407	0.043612604	0.07007786
GPR123	-0.106241024	0.043665124	0.07015659
UXS1	0.106238698	0.043669756	0.070158372
LRRC31	-0.10622314	0.043700754	0.070200919
CBLN2	-0.106221868	0.043703289	0.070200919
ENC1	-0.106186532	0.043773764	0.070308454
WTAP	-0.106183466	0.043779885	0.070312614
SCAND3	-0.106181094	0.04378462	0.070314548
METT10D	0.10616345	0.043819852	0.070362672
CSTF2	0.106162549	0.043821653	0.070362672
C9orf163	-0.106153358	0.043840016	0.070381038
FEM1A	-0.106153286	0.04384016	0.070381038
GOLGA6L6	0.106116193	0.043914338	0.070494441
SLC7A6	0.10608894	0.043968904	0.070576346
CCDC6	-0.106062675	0.044021547	0.07065515
SIPA1L2	-0.106060631	0.044025647	0.070656037
LOC400794	0.106050088	0.044046793	0.070684278
KERA	-0.10603721	0.044072637	0.070720052
C14orf147	-0.106031	0.044085104	0.070734358
ASGR2	0.10601652	0.044114183	0.070775313
LRIT1	0.10599456	0.044158317	0.070840413
SEMA4D	-0.10597995	0.0441877	0.07088184
ALAS2	-0.10595853	0.044230807	0.070943913
C21orf131	0.105957182	0.044233521	0.070943913
C1orf150	-0.10595265	0.044242646	0.070952834

MLH1	-0.105948727	0.044250547	0.070959791
FREM1	-0.105920266	0.0443079	0.071046042
AKR7L	-0.105917825	0.044312823	0.071048214
FNTB	0.105868683	0.044412008	0.071201509
LOC407835	0.105863654	0.044422169	0.071212065
HTR3E	0.105844504	0.044460877	0.071268381
OR4F5	-0.105836805	0.044476449	0.071287605
NMNAT1	-0.105819901	0.04451065	0.071336681
REG3G	0.105745486	0.044661482	0.071572657
RAB3C	0.105741578	0.044669416	0.071579611
TMCC2	-0.10569906	0.044755801	0.071712267
POLB	0.105648074	0.044859575	0.071872763
MS4A6E	-0.105641787	0.044872385	0.071887503
LOC25845	0.105637716	0.044880682	0.071895012
PNMAL1	-0.105627934	0.044900623	0.071921169
ENPP3	0.105590418	0.044977168	0.07203429
TAL2	0.105589776	0.044978478	0.07203429
SERPINA12	0.105586124	0.044985937	0.072040442
C6orf223	0.105565437	0.045028199	0.072100687
C18orf54	0.105564165	0.045030798	0.072100687
ARNT2	-0.10555504	0.045049451	0.072124755
FMO9P	0.105550504	0.045058728	0.072133808
GNG10	0.105532896	0.045094747	0.072185667
LRRC42	0.105524402	0.04511213	0.072207689
TNRC18	-0.105518129	0.045124973	0.072222442
C19orf2	0.105514805	0.045131779	0.072227529
MORC1	-0.105478388	0.045206401	0.07234114
TCF23	-0.105474918	0.045213517	0.072346712
ITGAM	-0.105461254	0.045241548	0.072385749
LRRC16A	-0.105456699	0.045250895	0.072394889
TAGLN3	0.105437227	0.045290871	0.072453024
C4A	0.10543527	0.045294891	0.072453216
ZYX	0.105433626	0.045298267	0.072453216
DDO	-0.105428015	0.045309793	0.072465831
KLF15	-0.105423931	0.045318184	0.072469528
RASL11A	-0.105423348	0.045319383	0.072469528
LOC284661	0.105412095	0.045342511	0.07250069
XKR8	0.105397603	0.04537231	0.072539463
SLC16A9	-0.10539676	0.045374044	0.072539463
MAGEB1	0.105374587	0.045419672	0.07260658
PVALB	-0.105323231	0.045525501	0.072769913
HMGB3	0.105308377	0.045556147	0.072813056
APBB1	0.105297552	0.045578493	0.072842925
RIMBP2	-0.105287231	0.045599808	0.072871142
FAM89A	-0.105276136	0.045622729	0.072901922
ZNF837	-0.105261157	0.04565369	0.072945187

SPINK13	0.105259493	0.04565713	0.072945187
NRN1L	0.105225572	0.045727314	0.073051456
TCFL5	0.105221459	0.04573583	0.073059201
S100A8	0.105210434	0.045758666	0.073089816
BTN2A1	-0.105207063	0.045765648	0.073095107
FAH	0.105200718	0.045778796	0.073110243
CD72	0.105186474	0.045808323	0.073151531
TKTL2	0.105127899	0.045929909	0.073339813
MRGPRD	-0.105105234	0.045977027	0.073409164
LARS	0.105062827	0.046065298	0.073544205
KCNS1	-0.105050596	0.046090783	0.073578994
KRTAP1-1	-0.105035943	0.046121333	0.073621861
MARK3	-0.105031182	0.046131262	0.073631809
LILRA3	-0.105027746	0.046138429	0.073637346
EXD1	0.105025173	0.046143796	0.073640011
FAM5C	0.105021196	0.046152092	0.073647348
C11orf57	-0.104973977	0.046250702	0.073798793
DNAJC1	0.104954141	0.04629218	0.073859058
CDC42	0.10494943	0.046302035	0.073868863
ANKRD46	-0.104921655	0.046360175	0.073955694
ZNF187	0.104897551	0.046410681	0.074030334
MGC14436	-0.104861062	0.046487224	0.07414649
AHRR	-0.104804281	0.046606546	0.074330853
FOLH1	-0.104793106	0.04663006	0.074362399
AGT	0.104790173	0.046636233	0.074366288
PARD6B	-0.104776524	0.046664969	0.074406154
PRSS53	-0.1047732	0.04667197	0.074411358
LRRC10B	-0.104719966	0.046784202	0.074584325
PPID	-0.104673796	0.046881725	0.074733816
TGM6	-0.104651941	0.046927947	0.074801511
LOC100130	-0.104627901	0.046978836	0.074876632
EFHB	-0.10459275	0.047053327	0.074989356
COG1	0.104582182	0.04707574	0.075019073
IRX2	-0.104576716	0.047087338	0.075031552
C21orf129	0.104569166	0.04710336	0.075051077
AHSG	0.104557082	0.047129014	0.075085945
HSD17B14	0.104526364	0.047194278	0.07518391
EMP2	0.104521077	0.047205521	0.075195804
C5AR1	-0.104486123	0.047279892	0.075308249
IFFO2	-0.104465468	0.047323885	0.075372295
FANK1	-0.104460337	0.047334821	0.075383684
TARM1	-0.104433553	0.047391931	0.075468599
ARSF	-0.104391582	0.047481541	0.075605252
PPFIBP2	-0.104385515	0.047494505	0.075619849
CNN1	-0.104378814	0.047508828	0.075636607
MPEG1	-0.104361572	0.0475457	0.075689258

C11orf58	-0.104353737	0.047562464	0.075709892
SIGLEC16	0.104351669	0.047566888	0.075710884
ABCC10	0.104338636	0.047594784	0.07574923
ZKSCAN3	0.104332995	0.047606863	0.0757624
TMC6	0.104311154	0.047653655	0.075830806
RRP1B	-0.104295061	0.047688155	0.075879643
CRMP1	-0.104258223	0.047767209	0.075996808
ATG5	-0.104257192	0.047769422	0.075996808
PANX2	0.104252586	0.047779315	0.076006475
FGF17	0.104228353	0.047831392	0.07608324
EPB41L4B	-0.104203163	0.047885572	0.076163338
HTR3B	0.104127552	0.048048513	0.076416399
LOC221122	-0.104118055	0.048069012	0.076442895
RAG2	-0.104105669	0.048095757	0.07647932
PRPH	0.104101635	0.048104471	0.076483313
TTC18	-0.104100951	0.048105949	0.076483313
GUCY2GP	-0.104098649	0.048110923	0.076485114
HYAL2	-0.104095073	0.048118647	0.076491288
TRIM3	-0.104090899	0.048127667	0.07649952
ABCG8	-0.104083596	0.048143452	0.076518503
NGB	0.10407404	0.048164112	0.076545043
PAIP1	-0.104072317	0.048167837	0.076545043
SNPH	-0.104069925	0.04817301	0.076547156
LRRC4B	-0.104060897	0.04819254	0.076571219
STEAP2	0.10405937	0.048195844	0.076571219
LOC727924	0.104054399	0.048206599	0.076582196
SLC25A29	0.104046199	0.048224347	0.076604281
SNORA11B	0.104014188	0.048293681	0.076707561
TMEM163	-0.104012626	0.048297068	0.076707561
KCTD4	-0.103976909	0.048374532	0.076824466
CENPL	0.103944847	0.048444158	0.076928906
PDE4D	-0.103942	0.048450344	0.076932595
LOC286135	0.103927933	0.048480923	0.076975013
AARS	0.103919653	0.048498928	0.076997461
SLC6A12	-0.103905507	0.048529704	0.077040181
RGS9	-0.103889757	0.048563988	0.07708846
APEX2	0.103876421	0.048593032	0.077128416
ZC3H12B	-0.103867774	0.048611874	0.077152173
IL1R2	0.103850249	0.048650077	0.077206652
DNAJC5	0.103762369	0.048842027	0.077505096
FANCL	0.103751628	0.048865531	0.077536217
OR52K2	-0.103679095	0.049024501	0.07778226
NIP7	0.103610362	0.04917554	0.078015683
ATP6V1D	0.103602675	0.049192456	0.078036304
FMO1	-0.103595534	0.049208175	0.078055023
MOBKL3	-0.103589325	0.049221846	0.078070489

DSTN	-0.103552992	0.049301907	0.078191245
FAM47C	-0.103538471	0.049333935	0.078235811
C6orf147	0.103511654	0.049393128	0.078323446
FABP2	0.103477804	0.049467933	0.078435818
C7orf23	0.10346319	0.049500256	0.078480822
LOC148413	0.103410678	0.049616549	0.078658938
C22orf31	0.103395592	0.049650001	0.078705705
B3GALNT2	-0.103391875	0.049658245	0.078712508
SORD	-0.103376064	0.04969333	0.078761852
DHX32	-0.103372705	0.049700787	0.078767402
LAMP3	-0.103310542	0.049838944	0.078980072
OR13C2	-0.103306662	0.049847579	0.07898747
MMP11	0.103302601	0.049856615	0.078995504
TLX2	0.103299998	0.04986241	0.0789984
NIPA2	-0.103286445	0.049892586	0.079039922
XPO6	0.103280309	0.049906252	0.079055282
KRTAP5-8	0.103220535	0.050039552	0.079260136
TSPY1	0.103203913	0.050076675	0.079312626
ZNF768	0.10320213	0.050080657	0.079312626
LOC100240	0.103194312	0.050098126	0.079333984
OR7E24	-0.103192441	0.050102308	0.079334298
DEFA5	0.103167398	0.050158301	0.079416646
NCRNA0005	-0.103160856	0.050172937	0.079433504
NKRF	-0.103155571	0.050184764	0.079445913
TICAM2	-0.103126923	0.05024891	0.079541137
TRAM1L1	0.103122267	0.050259343	0.0795486
ATXN2L	-0.103121254	0.050261613	0.0795486
LRPAP1	0.10308976	0.050332227	0.079654029
HNRPDL	0.103015986	0.050497962	0.079909965
PALM	0.103012896	0.050504913	0.079914615
LIPH	-0.10300336	0.050526372	0.079942218
C1orf96	-0.102999673	0.050534671	0.079948997
CRYBA2	0.102986007	0.050565441	0.079991322
PCBP2	0.102958169	0.050628167	0.080084189
GRIK5	0.102945373	0.050657022	0.080123469
C17orf82	0.102943246	0.050661821	0.080124695
PPDPF	0.102848451	0.050876025	0.080457083
TGM1	0.102839664	0.050895918	0.080482151
VPS26A	0.102789571	0.051009454	0.080655281
PPEF2	-0.102770193	0.05105343	0.080718408
C2orf78	-0.102759229	0.051078327	0.08075136
PPM1F	0.102737386	0.051127957	0.080823405
SMAP2	-0.102730608	0.051143364	0.080841344
SLC12A1	-0.102706441	0.051198333	0.08092181
TTC24	-0.102692963	0.051229013	0.080963876
WEE2	-0.102681	0.051256255	0.081000502

C14orf105	-0.102669387	0.051282712	0.08103588
DEFB1	-0.102661237	0.051301286	0.081058799
STK17A	0.102612439	0.051412619	0.081228267
GLYCTK	0.102591834	0.051459688	0.081296183
C11orf9	-0.10258586	0.051473342	0.081311304
SCML2	0.102579017	0.051488986	0.081329565
KIF21B	-0.102563322	0.051524882	0.08137981
ZBED5	-0.102544315	0.051568377	0.081442049
RPH3AL	-0.102527733	0.051606351	0.081495558
PTPN18	0.10250542	0.051657485	0.081569839
METTL13	0.102487493	0.051698597	0.081622461
INGX	0.102487315	0.051699007	0.081622461
ADRB1	-0.102473954	0.051729665	0.081664391
PHF13	-0.102469721	0.051739382	0.081673256
CARD11	-0.102464679	0.051750958	0.081685054
TPTE2P1	0.102442472	0.051801967	0.081759088
HDAC10	0.102433528	0.051822524	0.081785051
LMOD3	-0.102412441	0.051871014	0.081855091
PHIP	-0.102409866	0.05187694	0.081857956
TUBB8	0.102401861	0.05189536	0.081880533
TTLL13	0.10237902	0.051947948	0.081951253
IL27	-0.102378818	0.051948412	0.081951253
PIRT	0.102374341	0.051958725	0.08196103
C3orf52	-0.10229142	0.052150045	0.082256309
NFE2	0.102255625	0.052232816	0.082380339
LYSMD1	0.102117259	0.052553793	0.082880012
FKBP6	0.102115256	0.05255845	0.082880793
C18orf62	-0.102092035	0.052612483	0.08295943
UBQLNL	-0.102089877	0.052617507	0.082960783
MCM3APAS	-0.102059821	0.052687517	0.08306459
FAM153C	-0.102050389	0.052709503	0.083092674
PLG	-0.102005668	0.052813851	0.083250582
HIST1H3F	0.102002893	0.052820333	0.083254208
SH3BP1	0.101991876	0.052846068	0.083288181
SIGLEC10	-0.101982943	0.052866942	0.083314485
PHLDA3	-0.101961969	0.052915982	0.083385171
C7orf69	-0.101959668	0.052921363	0.083387052
SNORA71A	0.10191337	0.053029759	0.083551238
NUBPL	-0.101858043	0.053159532	0.083749076
PDE6G	0.101811391	0.053269164	0.083915156
NEURL	0.101788669	0.053322627	0.083985388
MAS1	-0.10178739	0.053325638	0.083985388
SNTB1	0.101787067	0.053326399	0.083985388
FBXO2	-0.101784783	0.053331776	0.083987215
ZNF788	-0.101752621	0.05340754	0.084099878
DDX19B	-0.101746573	0.053421796	0.084115676

WDFY2	-0.10168991	0.053555523	0.08431957
NCRNA0011	0.101685345	0.053566311	0.084329888
TMSB15B	-0.101670881	0.053600497	0.084377037
LRFN3	-0.101660797	0.05362434	0.084401565
OR11L1	0.101659626	0.053627109	0.084401565
IL24	-0.101658915	0.053628793	0.084401565
PCDHA5	-0.101649147	0.053651899	0.084431258
CYP4A11	-0.101627348	0.053703497	0.084505779
ZFP64	-0.101607493	0.05375053	0.084573106
LSM11	0.101593975	0.05378257	0.084616834
CBR1	0.101551842	0.053882538	0.084767418
C4orf42	-0.101524965	0.05394639	0.084861166
TMEM184B	-0.101504459	0.053995147	0.084931154
C13orf34	0.101494797	0.054018132	0.084958745
CXCR1	-0.1014935	0.05402122	0.084958745
ACADS	-0.101459213	0.054102859	0.08508042
CD59	-0.101452812	0.054118112	0.085097687
STX6	0.101437477	0.054154666	0.085148442
CD5	-0.101415949	0.054206019	0.085222458
WDR93	0.101393129	0.054260499	0.085301377
DIRC3	-0.1013887	0.054271076	0.085307645
ZNF527	-0.101387308	0.054274401	0.085307645
ANKRD58	0.101386079	0.054277337	0.085307645
KAT5	0.101318378	0.054439273	0.085555407
C3orf50	0.101311069	0.054456778	0.085576165
PSIP1	-0.101279503	0.054532438	0.0856883
ZNF184	-0.10120011	0.054723122	0.085979599
CCNJ	-0.101198723	0.054726458	0.085979599
ACVR2A	-0.101177454	0.054777638	0.086053219
SEMA3B	0.101172803	0.054788834	0.086064018
FAM178B	0.101163971	0.0548101	0.086090634
FAM86B2	-0.101161904	0.054815081	0.086091667
P2RX6	0.101152329	0.054838147	0.086121102
TTC32	0.101145528	0.054854536	0.086140047
LCN15	0.101130737	0.05489019	0.086189241
MBD1	-0.101128241	0.054896209	0.086191896
KLRG1	-0.10112116	0.054913288	0.086209501
POLD3	-0.101120003	0.05491608	0.086209501
VDAC3	0.101081325	0.055009448	0.086349268
R3HDML	-0.101074179	0.055026713	0.086369561
ARHGEF38	-0.101054586	0.055074072	0.086437082
DNAJC12	0.101046347	0.055093996	0.086460785
AANAT	0.101044751	0.055097857	0.086460785
EXTL3	-0.10085336	0.055562431	0.087182935
LOC100133	-0.100770194	0.055765314	0.087494385
ZNF274	-0.100762173	0.055784915	0.087518243

GSTM3	0.100694595	0.055950271	0.087770747
ZNF559	-0.100688177	0.055965997	0.087788503
LOC728723	-0.100681179	0.055983147	0.087808487
NUCB1	0.100678321	0.055990152	0.087811742
CSF1R	-0.100676735	0.05599404	0.087811742
LMX1A	0.100669458	0.056011881	0.087832805
TMC2	0.100665125	0.056022508	0.087842551
NAT8B	0.100660442	0.056033996	0.087853647
TFG	0.100656946	0.056042571	0.087855287
PCA3	0.100656418	0.056043865	0.087855287
TMEM8B	0.100646609	0.056067936	0.087886103
TNFRSF9	-0.100624102	0.056123194	0.087965796
PTK6	-0.100543962	0.056320318	0.088267816
LTC4S	-0.100469056	0.056505088	0.088550427
CLEC18B	0.100456411	0.056536331	0.088592417
LOC285401	0.100361366	0.056771608	0.088954096
HIBCH	-0.100359511	0.056776207	0.088954304
ALDOC	0.100344305	0.056813927	0.089006399
VWA5A	-0.100326665	0.056857709	0.089067984
UBE2QL1	-0.100317369	0.056880792	0.089097135
AMELY	0.100306237	0.056908446	0.089133442
RUNDC1	-0.100298007	0.056928898	0.089158462
C8orf39	-0.100289451	0.056950165	0.089184756
HSFYL1	-0.100285395	0.056960249	0.089193534
HBB	-0.100207364	0.057154549	0.089487626
C9orf135	0.100206362	0.057157048	0.089487626
NTAN1	0.10019542	0.05718434	0.089523318
TMEM126B	0.100181042	0.057220215	0.08957244
LCE1F	0.100137694	0.057328495	0.089734887
SVOPL	0.100119377	0.057374298	0.089799525
CD22	-0.10011468	0.057386049	0.089810858
SGSH	0.100111392	0.057394275	0.089816673
CLRN3	-0.100104398	0.057411777	0.089837003
TRPM2	0.100101319	0.057419484	0.089842003
C3orf66	0.100073796	0.057488412	0.089942787
SLC25A12	-0.100064081	0.057512758	0.089973808
FDXR	0.100062195	0.057517485	0.089974135
BHMT	-0.100026159	0.057607872	0.09010845
SLC29A4	0.100024146	0.057612926	0.090109276
LCE3D	-0.100015381	0.057634929	0.090136612
GAS2	0.09999161	0.057694643	0.090222914
HIST1H3G	0.099987673	0.057704538	0.090231302
HSH2D	0.099984229	0.057713194	0.090237752
IARS	-0.09997418	0.057738459	0.090270169
ANKRD24	-0.099941646	0.057820316	0.09039105
CD68	0.099927666	0.05785552	0.090438985

MAGEA8	0.099897009	0.057932783	0.090552654
RHBDF1	-0.099879684	0.057976484	0.09061385
ASPHD1	0.099874656	0.057989172	0.090626569
HLA-DOB	0.09987176	0.05799648	0.090630877
KCNC2	0.099852552	0.058044978	0.090699547
ACN9	0.099841802	0.058072134	0.090734861
SDHA	-0.099826465	0.058110895	0.0907883
NUB1	0.09978787	0.058208531	0.090933707
CCKBR	0.099778097	0.058233277	0.090965229
ALG13	-0.099745018	0.058317096	0.091089017
C17orf108	-0.099720195	0.058380064	0.091174857
PPAN-P2RY	-0.099719743	0.058381209	0.091174857
DUXA	0.099710053	0.058405806	0.091206118
C6orf165	-0.099698362	0.058435491	0.09124532
SAPS2	0.099694695	0.058444805	0.091247055
SERPING1	-0.099694316	0.058445766	0.091247055
CCR5	-0.099639732	0.058584549	0.091456557
MGP	-0.099624033	0.058624518	0.091511778
NRSN2	0.099617659	0.058640749	0.091529942
FBLN7	-0.099607649	0.058666251	0.09156257
TMEM176B	0.099603359	0.058677182	0.091572453
CYP4Z2P	-0.099584448	0.058725393	0.09164051
CST9	0.099548329	0.058817559	0.091777143
NUP210	0.099531784	0.058859817	0.091835887
C7orf10	0.099464229	0.059032627	0.092098297
C3orf51	0.099452005	0.059063942	0.092139935
SLITRK4	-0.099442452	0.059088423	0.092170904
KRTAP4-8	-0.099431135	0.059117436	0.092204092
TMPO	-0.09942885	0.059123295	0.092204092
YARS2	0.099428736	0.059123588	0.092204092
DNALI1	0.099425928	0.059130789	0.092208102
WFDC6	0.099422958	0.059138407	0.09221276
MIOX	0.099413862	0.059161742	0.092241924
DDX12	0.099389704	0.059223752	0.092331377
YPEL1	-0.099371084	0.059271584	0.092398716
MAPK8IP2	0.09934379	0.059341756	0.092499983
B3GALT1	-0.099342205	0.059345834	0.092499983
NRARP	-0.099325264	0.059389426	0.092560684
MS4A8B	0.099317741	0.059408791	0.092583619
AMD1	-0.099302472	0.059448114	0.092637652
CCDC84	0.099300119	0.059454177	0.092639851
CFHR5	-0.099296355	0.059463873	0.09264771
LHX4	0.099275816	0.059516813	0.092722939
LITAF	-0.099259025	0.059560121	0.092783152
SLC17A4	-0.099254505	0.059571785	0.092793793
C10orf99	-0.099252767	0.059576271	0.092793793

ULK3	-0.099245997	0.059593743	0.092813748
POU3F1	-0.09923758	0.059615472	0.092840328
MED24	0.099234166	0.059624287	0.092846797
ISX	0.099230468	0.059633839	0.09285441
POPDC2	-0.099211404	0.059683091	0.092923833
NRD1	0.099181244	0.059761082	0.093037988
CRYAB	0.099170788	0.059788138	0.093072835
EFCAB4A	0.09915474	0.059829686	0.093130232
RPGRIP1	-0.099148867	0.059844897	0.09314009
C9orf24	0.099148683	0.059845372	0.09314009
CARM1	0.099146777	0.059850311	0.093140497
C10orf131	-0.099139317	0.059869638	0.093163294
TMEM129	-0.099128612	0.059897381	0.093199077
C7orf68	0.099126833	0.059901993	0.093199077
ARVCF	0.099111664	0.059941325	0.093252987
SUGT1P1	-0.099088534	0.060001341	0.093339065
AQPEP	0.099082571	0.060016821	0.093355853
SLFN13	-0.09901828	0.060183937	0.093608489
TMEM9B	-0.099001039	0.060228818	0.093670981
STK32B	-0.098993167	0.060249318	0.093695546
MMP19	0.098969199	0.060311775	0.093778172
HIST1H2BA	-0.098969164	0.060311867	0.093778172
CENPE	0.098950219	0.060361271	0.093847663
COL23A1	-0.098935564	0.060399511	0.093899787
NUDT11	-0.098925333	0.060426219	0.093933976
NKX2-4	-0.098898649	0.060495925	0.094034994
GBP6	-0.098864809	0.060584416	0.094165195
LILRA4	-0.098842846	0.060641908	0.094247199
CREM	-0.098783392	0.060797764	0.09448205
UPB1	-0.098774195	0.060821904	0.094512189
LOC158696	-0.098769058	0.060835391	0.094525771
SMARCD2	0.098755876	0.060870008	0.09457218
TWIST1	-0.098738773	0.060914948	0.094634619
PIP5K1B	-0.098733242	0.060929486	0.094649822
RDH16	-0.098699837	0.061017358	0.094778932
ZC3H15	-0.098693579	0.06103383	0.094797125
ARMC3	0.098677858	0.06107523	0.094854028
REP15	0.098670107	0.06109565	0.094878343
TEAD2	0.098659513	0.061123568	0.094914297
DLG5	-0.098657314	0.061129366	0.094915899
FXYD4	0.098634094	0.061190601	0.095003572
BTLA	-0.098608001	0.06125947	0.095103082
PCDHA1	0.098593073	0.0612989	0.095156878
TNFSF13B	-0.098526007	0.061476304	0.095424831
VSIG10	-0.098490021	0.061571668	0.09556541
RAP1GDS1	-0.09847723	0.061605594	0.095610615

FAM75C1	0.098454846	0.061665002	0.095695357
SH2D5	0.098437965	0.061709837	0.095757472
SPPL2B	0.098430408	0.061729916	0.095781168
C3orf31	0.098422514	0.061750896	0.095806256
SNORA67	0.098408875	0.061787159	0.09585505
PCOLCE2	0.0983836	0.061854404	0.095951897
CLDN18	0.098375641	0.061875594	0.095977292
HDAC4	-0.098355689	0.061928734	0.096052237
NOL6	-0.09834346	0.061961326	0.096095305
SYN3	0.098326523	0.062006485	0.096157853
REG1B	0.098311808	0.062045744	0.096211243
DUSP5	-0.098302686	0.062070089	0.096241499
SFRS1	-0.098299242	0.062079285	0.096248265
ELAVL4	-0.098277243	0.062138038	0.096331857
SPDEF	0.098197377	0.06235173	0.096655616
OPRK1	0.098183757	0.062388233	0.096703133
PIM1	0.098182316	0.062392094	0.096703133
MAPK15	0.098141388	0.062501895	0.096865778
KIF25	0.098103202	0.062604485	0.097017222
C11orf94	0.09809482	0.06262702	0.097044592
C1orf226	-0.098090883	0.062637609	0.097053449
KIR3DX1	-0.098088628	0.062643674	0.097055296
CST7	0.098055562	0.062732665	0.097185611
LOC100128	-0.098051008	0.06274493	0.097190606
FAM177B	-0.09805074	0.06274565	0.097190606
SLC22A18AS	0.098033015	0.062793404	0.097250435
CLSPN	-0.098032779	0.062794042	0.097250435
TAB1	0.097987177	0.062917041	0.097433349
MYO7B	-0.097983505	0.062926953	0.097441122
LOC727896	-0.097973593	0.062953718	0.097474987
IPO4	0.097967913	0.06296906	0.097491162
OR6A2	-0.097921025	0.06309582	0.097679824
METTL8	-0.09790542	0.063138053	0.097734966
LHCGR	-0.097904238	0.063141254	0.097734966
PHYHD1	-0.097899652	0.06315367	0.097746587
ASB14	-0.097897733	0.063158867	0.097747035
ANKRD33	0.097894645	0.063167229	0.097752379
SPDYA	0.097873762	0.063223806	0.097832331
FRMPD4	-0.097871749	0.06322926	0.097833169
LOC100128	-0.097851259	0.063284817	0.097911524
SLC2A11	-0.097841832	0.063310392	0.097943483
TBC1D29	-0.09782604	0.063353252	0.098002176
C22orf46	-0.097803632	0.063414108	0.098088696
C6orf94	0.097779675	0.063479226	0.098181794
PPP1R15A	-0.097775	0.06349194	0.098193832
FNBP1	-0.097728926	0.063617346	0.09838014

SLITRK3	-0.097701815	0.063691235	0.098486757
SPATS2	0.097689197	0.063725648	0.098532319
KIAA1328	-0.097680931	0.063748201	0.098559538
RPS2P32	0.09767845	0.063754971	0.098562354
PKHD1L1	0.097676194	0.063761127	0.09856422
AGAP6	-0.097663919	0.063794636	0.098608365
FAM53A	0.09765906	0.063807903	0.098621217
CHRM1	0.097620882	0.063912231	0.098774801
IL12RB2	-0.097599001	0.063972087	0.098859635
TSGA13	0.097593169	0.063988048	0.098876627
LACTB2	-0.097569958	0.064051606	0.098962986
UTS2D	-0.09756913	0.064053873	0.098962986
SAT1	-0.097544257	0.064122041	0.099060134
SHISA9	-0.097542558	0.064126701	0.099060134
ZCWPW2	-0.097531853	0.064156059	0.099097799
FCGBP	0.097526482	0.064170794	0.099112871
GNB1	-0.097518988	0.064191357	0.099136942
HOXA13	-0.097483712	0.064288225	0.099278846
KIAA0802	-0.09746982	0.064326404	0.099330103
FAM18A	-0.097464803	0.064340197	0.099335895
DMRT2	0.097464482	0.064341082	0.099335895
CYCS	0.097463013	0.064345119	0.099335895
TRPC4AP	0.097448836	0.064384112	0.099384619
NOX5	-0.097447909	0.064386661	0.099384619
FLJ35220	0.097439975	0.064408493	0.099410613
C10orf57	-0.097435819	0.064419929	0.099420559
CENPO	0.097427178	0.064443718	0.099449566
IFT88	-0.097423588	0.064453602	0.099457112
SNORA18	-0.097399283	0.064520554	0.099552711
SMAD3	-0.097397129	0.064526491	0.099554157
DCAF8L1	0.097361979	0.064623427	0.099695991
P2RX5	0.09733978	0.064684708	0.099782801
LOC100131	-0.097331274	0.0647082	0.099811308
FAM116B	0.097314622	0.064754216	0.099874551
ATP13A1	0.097304399	0.064782477	0.099910402
KRTAP19-8	-0.097254308	0.064921103	0.100116445
LOC100128	-0.097245589	0.064945256	0.100145937
TERF2IP	-0.097240608	0.06495906	0.100159467
INSC	-0.097226502	0.064998159	0.100211995
KRTAP12-2	-0.097198006	0.065077208	0.100326102
DNAJB6	0.097191625	0.065094919	0.100345639
ST6GALNAC	0.097145348	0.065223487	0.100531545
PDLIM3	-0.097143546	0.065228498	0.100531545
S100A11	0.097142768	0.065230662	0.100531545
GJA10	-0.097135009	0.065252241	0.10055702
ZNF502	-0.097118275	0.0652988	0.100620842

PIWIL1	-0.097115129	0.065307556	0.100620842
PEX5L	-0.097114678	0.065308812	0.100620842
CLPB	-0.097110734	0.065319791	0.100629971
PQLC1	0.097058658	0.065464897	0.100845716
NUDT16P1	-0.097042595	0.065509708	0.10090694
DNAJC28	-0.097029981	0.065544916	0.100953364
KIAA0195	0.096987475	0.06566367	0.101128449
ITGB7	-0.096930144	0.06582412	0.10136418
C12orf76	0.096929148	0.065826911	0.10136418
BRD7P3	-0.096909834	0.065881039	0.101439686
RPS27L	0.096885466	0.065949384	0.101537069
DCTN4	-0.096876516	0.065974502	0.101567889
PHTF2	-0.096872061	0.065987005	0.101579285
OR8B3	0.096860183	0.066020357	0.10161649
TSPAN10	0.096859819	0.066021379	0.10161649
GUSB	0.096805992	0.06617269	0.101841508
PRB3	0.096798302	0.066194328	0.101866938
C2orf89	0.096760961	0.066299491	0.10202089
HERC5	-0.096743324	0.066349209	0.102089507
LOC572558	-0.0967258	0.066398639	0.102157672
ANLN	0.096723825	0.066404212	0.102158353
INTU	-0.096711744	0.066438307	0.102202912
WNT16	-0.09666262	0.0665771	0.102408507
CFB	0.096616441	0.066707791	0.10260161
TAX1BP3	0.096599546	0.066755655	0.102667301
CCDC18	-0.096570865	0.066836977	0.102784432
RILP	0.09655302	0.066887618	0.102854367
ZFP62	-0.096549324	0.066898109	0.102862558
RAB23	-0.096539247	0.066926722	0.102898608
GSX1	0.096516524	0.066991279	0.102989913
C19orf20	0.096491429	0.067062636	0.103091656
TWIST2	-0.096483585	0.067084951	0.103117999
FAM92A1	-0.096481615	0.067090557	0.103118658
CIRH1A	0.096476977	0.067103755	0.103130984
FCGR1A	0.096459126	0.067154577	0.103201127
LOC147727	0.096432271	0.067231094	0.103310744
PLK4	0.096428485	0.067241886	0.103319355
DUSP11	-0.096426402	0.067247825	0.103320509
RTEL1	0.096405388	0.06730776	0.103404616
KREMEN2	0.096399467	0.067324656	0.103422595
AOAH	-0.096366845	0.067417803	0.103553187
SH2D3A	0.096366053	0.067420066	0.103553187
GABRA3	0.096348463	0.067470339	0.10362241
RD3	-0.096316341	0.067562221	0.103755523
LOC400657	0.096301081	0.067605906	0.103814606
CDK19	-0.096293077	0.067628829	0.103841799

CA5BP	-0.096288409	0.067642201	0.103854324
FLJ37543	-0.096216259	0.067849157	0.104164042
CXCL9	-0.096165528	0.067994981	0.10437987
LDHD	-0.096149507	0.068041089	0.104442599
CYB5D2	-0.096137279	0.068076295	0.104483501
ARRDC2	-0.096136608	0.068078228	0.104483501
MGC16121	0.096125794	0.068109378	0.104523253
MLX	0.096119821	0.068126586	0.104541606
PSME4	-0.096110519	0.068153395	0.104574687
CCDC120	-0.096094458	0.068199706	0.104637685
UBLCP1	-0.096085273	0.0682262	0.10467027
LRRC8D	-0.096082487	0.068234238	0.104674538
GYPA	-0.09606743	0.068277692	0.104733131
C1orf189	0.096061742	0.068294115	0.104743988
C22orf23	-0.096060053	0.068298994	0.104743988
CALN1	-0.096059514	0.068300549	0.104743988
SCARNA21	0.096045161	0.068342006	0.104799496
CCNG1	-0.096027336	0.068393518	0.104870412
GIPR	-0.096021735	0.068409711	0.104887165
SSX2IP	-0.095962241	0.068581908	0.105143087
SMAP1	0.09594148	0.068642081	0.105227238
POLR2J3	0.095909467	0.068734953	0.105356315
TOR3A	0.095908809	0.068736862	0.105356315
UOX	-0.095892727	0.068783558	0.105419776
ACTR1B	-0.095889526	0.068792856	0.105425912
ADPGK	-0.095886208	0.068802493	0.105432567
FBXO7	-0.095864847	0.06886457	0.105519574
LOC285692	0.095847631	0.068914634	0.105588163
C21orf54	-0.095835147	0.068950956	0.105629833
FAM123C	0.095834637	0.068952439	0.105629833
NAT8	0.095828847	0.068969289	0.105647519
PRKCQ	-0.095786241	0.069093396	0.105829487
ZNF846	0.095764527	0.069156717	0.10591285
LOC100289	0.09576393	0.069158459	0.10591285
PRKCI	-0.095752772	0.069191016	0.105954562
PCDH8	0.095733569	0.069247076	0.106032256
PSTPIP2	-0.095722342	0.069279869	0.106074313
LOC400891	0.095674733	0.069419071	0.106279274
CACNG2	0.09566126	0.069458503	0.10633147
PMS1	-0.09561993	0.069579589	0.106505604
CTDSPL	0.095618784	0.069582948	0.106505604
ERVFRDE1	-0.095614305	0.069596081	0.106517519
GPR12	-0.095581556	0.069692167	0.106656382
PFKP	-0.095566015	0.069737801	0.10671802
SLITRK2	-0.095514929	0.069887987	0.106939628
IL5RA	-0.095505997	0.069914272	0.106971629

ICAM4	0.095504114	0.069919813	0.106971889
IRF4	-0.095498931	0.069935073	0.106987016
HIST2H2BE	-0.09546709	0.070028863	0.107122268
METTL9	0.095460246	0.070049038	0.107144899
SCGB3A1	0.095455091	0.070064235	0.107159444
VPS35	-0.09545337	0.070069308	0.107159444
PCYOX1L	-0.095429771	0.070138921	0.107257669
CDH3	-0.095420141	0.070167347	0.107292899
HMGN3	0.095412289	0.070190527	0.107320103
ARTN	0.095401871	0.070221295	0.107358904
CRABP2	-0.095395479	0.070240178	0.10737953
MS4A13	0.095393037	0.070247395	0.10738232
HS3ST4	0.095387671	0.070263252	0.107398316
VANGL2	-0.095376046	0.070297612	0.10744259
PIGW	-0.095373599	0.070304849	0.107445405
LRRC24	0.09533804	0.070410051	0.107597926
SULT1A2	0.095329949	0.070434006	0.10762222
HES3	0.09532902	0.070436757	0.10762222
CCDC74A	-0.095316183	0.07047478	0.107672056
OLR1	-0.095263166	0.070631988	0.107903962
PSG10	-0.095204267	0.070806975	0.108155389
MOCOS	-0.095204115	0.07080743	0.108155389
ZNF80	-0.095194535	0.070835925	0.108190616
SCN10A	-0.095180191	0.070878607	0.108247504
HBM	-0.095173923	0.070897266	0.108267698
C4orf6	0.095171271	0.07090516	0.108271451
CACNA1E	0.095164228	0.070926134	0.10829407
MYLPF	0.095161245	0.070935017	0.10829407
TCP10	0.095160819	0.070936286	0.10829407
HMGN5	-0.095157637	0.070945764	0.108300236
CD109	-0.095140571	0.070996612	0.108369551
TM2D1	-0.095130026	0.071028049	0.108409227
CDHR5	0.095120536	0.071056348	0.108444109
ODF3B	0.095096468	0.071128162	0.10854539
LOC392196	-0.095079561	0.071178643	0.108614104
POLR3G	0.095065171	0.071221632	0.108671376
RB1CC1	-0.095043422	0.071286647	0.108762244
KRTAP3-2	0.095039297	0.071298983	0.108772733
BAI1	-0.095019122	0.071359343	0.108856478
FAM188B	0.095008074	0.071392414	0.108898586
RAD9B	-0.094997249	0.071424833	0.108939692
SLC35C1	-0.094977023	0.071485434	0.109023772
SEL1L3	-0.094934437	0.071613165	0.109210214
PPP4R1	-0.094929663	0.071627497	0.109223706
MMP3	0.094926537	0.07163688	0.10922965
GPR88	-0.094872088	0.07180051	0.109470767

MGAT5B	-0.094858427	0.071841613	0.109523009
PTS	0.094857044	0.071845774	0.109523009
CACNA1I	-0.094844572	0.071883316	0.109571851
PTK2	0.094813715	0.071976272	0.109705147
GRRP1	-0.094807583	0.071994754	0.109724919
TMOD1	-0.094795265	0.072031895	0.109773125
PEG10	0.094781793	0.072072537	0.109826656
LOC282997	0.094777076	0.072086771	0.109839942
PAX5	-0.094764961	0.072123339	0.109887254
WHSC1	-0.094753431	0.072158154	0.109929797
SH2D1A	-0.094752058	0.072162302	0.109929797
CPNE2	-0.094730455	0.072227575	0.110020816
APBA2	-0.094718189	0.072264656	0.110068882
RASIP1	-0.094695921	0.072332016	0.110159546
SAMD7	-0.094694854	0.072335244	0.110159546
MET	-0.094675281	0.072394499	0.110235828
CERK	-0.094674651	0.072396404	0.110235828
SCARB1	0.094637911	0.072507737	0.11039691
GNAI3	-0.094597271	0.072631048	0.110576203
ZNF528	-0.094563195	0.072734574	0.110725351
TMPRSS4	-0.094550234	0.072773983	0.110776876
DNAH6	-0.094544916	0.072790156	0.110793027
IRF5	0.09454108	0.072801824	0.110802318
A4GALT	-0.094516875	0.072875492	0.110905963
SLC16A10	0.094502426	0.072919494	0.110964447
ASAP1IT1	-0.094450322	0.073078353	0.111197692
SLC6A14	0.09440439	0.073218627	0.111402624
OR5M8	0.09440062	0.073230148	0.111411642
FGF5	-0.094397943	0.073238331	0.111415581
PBOV1	-0.094388188	0.073268158	0.111444476
CDH7	-0.094388071	0.073268517	0.111444476
OR8B2	0.094384401	0.07327974	0.111453033
PNPLA6	0.094366446	0.073334672	0.111528064
LOC150185	0.094350869	0.073382353	0.111592055
ALOX15B	-0.09431065	0.073505585	0.111770919
LGR5	0.094291413	0.073564585	0.11184844
TFAP2B	0.094290366	0.073567799	0.11184844
NIPSNAP1	0.09428426	0.073586535	0.111868384
HOXB1	0.094271008	0.07362721	0.111921677
PTPN6	0.094263094	0.073651512	0.111950073
MACROD1	0.09426091	0.073658221	0.111951726
ALAS1	-0.094247842	0.073698366	0.112004193
NSL1	0.094229608	0.073754409	0.112080813
PRR15L	0.094200945	0.073842575	0.112206233
C1orf187	-0.094185533	0.073890019	0.112269759
CD160	0.094183132	0.073897413	0.112270309

C1orf175	-0.094181754	0.073901656	0.112270309
F10	0.094140134	0.074029917	0.112456583
MRPL34	0.094134186	0.074048261	0.112475869
DCAKD	-0.094105034	0.074138227	0.112603935
GYG2	0.094073682	0.074235078	0.112742437
KIAA1908	-0.094066948	0.074255896	0.11276079
ACCN1	-0.094064543	0.07426333	0.11276079
FBF1	-0.094064278	0.074264148	0.11276079
FOXD4L5	-0.094060905	0.074274578	0.112768028
LOC126536	0.094049444	0.074310026	0.112813247
SMPDL3B	0.094045069	0.074323561	0.112825193
TBKBP1	0.094035437	0.074353366	0.112861835
SNORA41	0.09402238	0.074393783	0.112914579
ZNF143	-0.093974509	0.074542121	0.113131104
TMEM105	0.093957311	0.074595472	0.113203446
PI4KB	0.093947677	0.074625371	0.113240191
ARHGEF4	-0.093909673	0.074743415	0.113410674
MSTO2P	0.093907655	0.074749687	0.113411551
APOBEC1	-0.093862637	0.074889722	0.113615359
OR1J4	-0.093852441	0.074921466	0.113654859
CA12	-0.093842218	0.074953307	0.113694502
PGA3	0.093803781	0.075073122	0.113867572
CCDC42	-0.093784052	0.075134682	0.113951914
SDS	-0.093782293	0.075140172	0.113951914
APOB	-0.09377261	0.075170403	0.113989079
PEBP1	0.093756886	0.075219513	0.114054865
TMPRSS11B	0.093746285	0.075252637	0.114096404
GPR85	-0.093723105	0.07532511	0.114197592
CPN2	-0.093700851	0.07539474	0.114294454
LRRC67	0.093641594	0.075580401	0.114567185
HIST1H2BL	0.093637018	0.075594754	0.114580221
FLNB	-0.093633393	0.075606125	0.114588735
NCRNA0011	0.093615257	0.075663041	0.114666271
CANX	-0.093599086	0.075713816	0.114734488
NANOS2	0.093590269	0.075741511	0.114767724
C10orf90	-0.093581889	0.075767844	0.11479889
LOC286002	-0.093574183	0.075792065	0.114826853
MLN	0.093568686	0.075809346	0.114844296
LOC100188	-0.093561509	0.075831915	0.114869748
PKN3	0.093548217	0.075873724	0.11492434
ZNF74	0.093544096	0.075886688	0.114935234
EBI3	0.09353634	0.075911097	0.11496346
SCARNA14	0.093506022	0.076006572	0.115099299
LIPN	-0.093495685	0.076039146	0.115139871
GPR120	-0.093468495	0.07612488	0.115259036
DNM1	0.093467057	0.076129418	0.115259036

ZSCAN22	-0.093464148	0.076138596	0.115264168
ANKS4B	0.093426862	0.076256312	0.115433601
HOXB6	-0.093421881	0.07627205	0.115448649
GSTTP2	-0.093407122	0.076318692	0.11551047
CC2D1A	-0.093394892	0.076357363	0.115560215
ID3	-0.093367956	0.076442587	0.115680404
OR56A5	0.093343412	0.076520308	0.115789221
CD177	0.093298674	0.076662143	0.115995029
PPM1M	0.093287488	0.076697641	0.116039924
USP54	-0.09326857	0.076757704	0.116113626
ENTPD8	-0.093268472	0.076758016	0.116113626
OR52E4	-0.093244432	0.076834395	0.116220338
TRIM43	-0.093238656	0.076852755	0.116239281
ENOPH1	-0.093236196	0.076860576	0.116242283
GULP1	-0.09319796	0.076982223	0.116411387
HOXD8	0.093197376	0.07698408	0.116411387
ANK2	-0.093193359	0.076996869	0.116421885
NCCRP1	0.093183521	0.077028201	0.116460419
ROM1	0.093175041	0.077055212	0.116492412
RPL13P5	-0.093149341	0.077137125	0.116607397
ZNF805	-0.093135097	0.077182556	0.116667218
PGK1	-0.093093576	0.077315109	0.116858712
CPS1	-0.093060552	0.077420666	0.117009378
GRSF1	-0.093051695	0.077448999	0.117043316
GBP5	-0.093017148	0.077559584	0.117201541
MARVELD2	-0.092991411	0.077642051	0.117317257
SNORA27	0.09297241	0.077702979	0.117400412
CA7	0.092931356	0.077834757	0.117590592
CCDC115	-0.092925928	0.077852191	0.11760801
C7orf31	0.092888826	0.077971461	0.117779252
SNORA62	0.092871011	0.078028782	0.117856898
TRIM40	0.09283975	0.07812945	0.117996168
GDF10	0.092838699	0.078132838	0.117996168
EIF4ENIF1	-0.092832446	0.078152986	0.118017647
ZNF653	0.092779543	0.078323629	0.118266363
CLP1	0.092736009	0.078464274	0.118469752
NRG2	-0.092728412	0.078488839	0.118497856
CCKAR	0.09269331	0.078602422	0.118660343
C3orf23	-0.092635106	0.078791055	0.118936091
OR11H4	0.09261699	0.078849841	0.119015809
MYO18B	0.09260016	0.078904486	0.119089264
GPR152	-0.092590771	0.078934984	0.119123806
NXPH4	0.092589431	0.078939336	0.119123806
HS6ST3	-0.092551526	0.079062566	0.119300728
R3HDM1	-0.092541729	0.07909444	0.119339782
ADCY10	-0.092523161	0.079154881	0.119421928

FOXD4L6	-0.092473702	0.079316061	0.119656038
PECR	-0.092463922	0.079347962	0.119695097
BAIAP2L1	0.092445173	0.079409153	0.119778329
FAHD1	0.092434085	0.079445355	0.119823861
DDX50	0.092431211	0.079454742	0.119823971
LHPP	0.092430378	0.079457462	0.119823971
INCA1	0.092424813	0.07947564	0.119842308
NCKAP1L	-0.09240106	0.079553269	0.119950284
FAM92A3	-0.092395156	0.079572572	0.119970306
SCIN	-0.092385765	0.079603286	0.120007528
MMP12	0.092377093	0.079631657	0.120041212
CES2	-0.092360887	0.079684698	0.120112077
RNF26	0.092349537	0.079721861	0.120159
HOXD1	0.092342965	0.079743387	0.120182349
RAB3IP	-0.09233651	0.079764535	0.120205125
MEGF8	-0.092333269	0.079775154	0.120212031
ANKLE1	0.092317993	0.079825224	0.12027838
RNF144A	-0.09231042	0.079850054	0.120306689
RAD51AP1	0.092281219	0.079945858	0.120441921
SLC22A23	0.092247085	0.080057964	0.12060169
NUDT16	0.092241466	0.080076432	0.120620387
OR11H6	0.092221332	0.080142629	0.12071097
SFXN3	-0.092212471	0.080171778	0.120745743
ST6GALNAC	0.092208174	0.080185916	0.120757904
TCP10L2	0.092197129	0.080222264	0.120797349
ARHGAP11A	0.092196529	0.08022424	0.120797349
C1orf133	-0.092181009	0.08027534	0.120852367
SNX14	-0.092180783	0.080276085	0.120852367
IRAK2	-0.092179903	0.080278984	0.120852367
FSCN2	-0.092147539	0.080385631	0.121003768
KRT17	0.092131886	0.080437254	0.121072324
RASGRP2	-0.092108185	0.080515469	0.121173353
UMOD	0.092107859	0.080516544	0.121173353
SUSD1	-0.092059205	0.080677301	0.121406109
ZNF549	-0.092055301	0.080690212	0.121416363
ZNF670	-0.092018965	0.080810452	0.121588105
TAS1R3	-0.091982465	0.080931381	0.121760856
TTC26	-0.091952138	0.08103197	0.121902982
MIXL1	0.091941427	0.081067519	0.12194725
PGA5	0.091910488	0.081170279	0.122092606
SLC12A7	-0.091904792	0.08118921	0.122111858
GMIP	-0.09190133	0.081200715	0.122119939
NEK3	0.09189877	0.081209227	0.122123518
NMU	0.091885869	0.081252122	0.122178798
DDB1	-0.091855439	0.081353378	0.122321821
PARD6A	0.091849973	0.081371576	0.122339947

LOC642852	-0.091846656	0.081382621	0.122347316
LOC100130	0.091842841	0.081395326	0.1223518
DARS	-0.091842071	0.081397891	0.1223518
TNFRSF19	-0.091839795	0.081405472	0.12235396
ACTR6	0.091776164	0.081617638	0.122663592
PAK1	0.091722335	0.081797472	0.122924588
CST1	0.091716455	0.081817133	0.122944859
CHRD	0.091709954	0.081838879	0.122968256
GGA3	0.091705269	0.081854553	0.122982528
TPRX1	0.091666218	0.081985287	0.123169658
SEC61A1	0.091648984	0.082043036	0.12324712
KIAA0020	0.091641035	0.082069686	0.123277854
USP19	-0.091563424	0.082330231	0.123659896
ZMIZ2	-0.091524225	0.082462077	0.123848586
RNF32	0.091476076	0.08262426	0.124082809
SPATA5	-0.09146871	0.082649094	0.124110746
SNORA13	0.091420461	0.082811913	0.124345868
FLJ46361	0.091418385	0.082818925	0.124347022
ECHDC2	-0.091394677	0.08289903	0.124457911
HIST1H2BM	0.091381695	0.082942918	0.124514414
C1QB	0.09136072	0.08301387	0.124611534
MDGA2	0.091356523	0.083028074	0.124623462
CPA5	0.091353565	0.083038084	0.124629093
OR1K1	-0.0913376	0.083092135	0.124694016
ABHD10	0.09133709	0.083093863	0.124694016
MAGEB3	0.091333263	0.083106823	0.124704068
CCL4L2	0.091300593	0.083217533	0.124860783
PRODH	-0.091293343	0.083242119	0.124888263
TEKT1	-0.091289348	0.083255668	0.124899181
C11orf16	-0.091276964	0.083297679	0.124940423
KIF17	-0.091275814	0.083301582	0.124940423
LOC402644	0.091275697	0.08330198	0.124940423
NT5DC3	0.091253234	0.083378232	0.125028847
UCMA	0.091253149	0.083378522	0.125028847
PLAGL2	-0.091252781	0.08337977	0.125028847
ZRANB2	-0.09124995	0.083389384	0.12503385
ESX1	0.091229194	0.083459903	0.125130163
FOXA1	-0.091221929	0.083484594	0.12515776
TCL6	0.091203784	0.083546293	0.125233993
RWDD2B	-0.091203276	0.08354802	0.125233993
ALDH1L1	0.091195352	0.083574978	0.125264972
LRP10	-0.091163606	0.083683046	0.125417509
TCERG1L	-0.091111916	0.083859242	0.125667495
CAPN11	-0.091110971	0.083862466	0.125667495
PMFBP1	0.091089095	0.083937129	0.125769913
DUOX2	-0.091058937	0.084040143	0.125914795

PDGFA	-0.091051036	0.08406715	0.125945783
OR4D1	-0.091017184	0.084182935	0.12610976
C10orf107	-0.09101448	0.084192188	0.126114136
UBTFL1	0.091005482	0.084222991	0.126150787
PEX14	0.090991804	0.084269828	0.126211448
DNAJC21	-0.090988199	0.084282177	0.126220451
PRO0628	-0.09097807	0.084316879	0.126262926
AAGAB	0.090959622	0.084380112	0.126348116
GLYATL2	0.090920627	0.0845139	0.126538931
43894	-0.090916029	0.084529688	0.126553055
ZNF772	-0.090903679	0.084572103	0.126598471
STARD3NL	0.090903494	0.084572737	0.126598471
TMEM99	0.090886741	0.0846303	0.126675117
ZNF22	-0.090851603	0.084751135	0.126840456
TTLL11	0.090850916	0.0847535	0.126840456
BCAR4	0.090845659	0.084771589	0.126854129
COL5A3	-0.090844559	0.084775376	0.126854129
DPP6	0.090824808	0.084843372	0.126946338
FAM22D	-0.090820867	0.084856944	0.126957106
CXorf40B	0.090802613	0.084919835	0.127041656
MPP6	0.090770604	0.085030208	0.127195978
HMGCS2	-0.090768994	0.085035765	0.127195978
SPATA20	-0.090752284	0.085093432	0.127272678
MYBBP1A	0.090747363	0.08511042	0.127288527
ENTPD3	-0.090732993	0.085160044	0.127353178
BEX1	0.090698585	0.085278963	0.127521441
DUSP8	-0.090681439	0.085338275	0.127600552
TRIM49L	0.090677216	0.085352888	0.12761282
PPP1R3C	-0.090665365	0.085393906	0.127664563
BMP6	0.090630419	0.085514952	0.127835931
NUDT9	-0.090618202	0.085557302	0.12788964
CCL18	-0.090604106	0.085606185	0.127953105
MAL	0.090601927	0.085613744	0.1279548
LUZP4	0.09059186	0.085648673	0.127997397
PLD4	-0.090577768	0.085697588	0.128060888
TRIM46	0.09055683	0.085770302	0.128159931
LPAL2	-0.090553807	0.085780807	0.128166011
DKFZp434L1	0.090541509	0.085823543	0.128220245
NPSR1	0.090504354	0.085952767	0.128403673
C21orf67	0.090492068	0.085995532	0.128457922
L1TD1	-0.09048435	0.086022403	0.128488424
PCBP3	-0.090478296	0.08604349	0.128510282
MRPL35	-0.090453825	0.086128757	0.128627986
IDI1	0.090450259	0.086141186	0.128636902
DPF2	0.090442918	0.086166781	0.128665475
GDPD2	-0.090440181	0.086176326	0.128670081

TPD52	0.090434683	0.086195501	0.128689062
ATP1B3	0.090432578	0.086202845	0.128690379
RWDD2A	0.090403158	0.086305517	0.128833998
CD99L2	-0.090385628	0.086366741	0.128915728
FBXO10	-0.090383215	0.086375171	0.128918648
LRAT	-0.090355425	0.086472309	0.129050783
IMPA1	-0.09035418	0.086476661	0.129050783
OR6W1P	0.090351861	0.086484771	0.129053216
SFRS6	-0.090327128	0.086571306	0.129172665
GSTM4	0.090313617	0.086618609	0.129233563
OPLAH	-0.090306137	0.086644807	0.129262966
LYPD6	-0.090298033	0.086673195	0.129295632
VNN3	0.090287364	0.086710579	0.12934171
C14orf182	-0.090283745	0.086723265	0.129350945
APOA5	-0.090227192	0.086921672	0.129637168
LDLRAP1	-0.090217665	0.086955133	0.129677361
SERTAD1	0.090184951	0.087070107	0.1298391
RAB33A	0.090173279	0.087111157	0.129890588
SNORA42	0.090147788	0.087200865	0.130014617
ITGA5	-0.090119447	0.08730069	0.130153709
BZRAP1	-0.090109926	0.087334248	0.130193992
MAN2B1	0.090104664	0.087352798	0.130211899
EARS2	-0.090087767	0.087412386	0.130290971
ZNF200	-0.09007051	0.087473277	0.130371974
RAMP1	0.090063499	0.087498024	0.130399099
EGOT	-0.090061271	0.087505888	0.13040106
LSM14A	-0.090022437	0.08764308	0.130595731
RSPO4	0.089990431	0.087756279	0.130751992
SNORA6	0.089989075	0.087761079	0.130751992
CD2AP	0.089973078	0.087817703	0.130826567
LRRFIP2	-0.089934573	0.08795412	0.131019994
TMIGD1	0.089888217	0.088118578	0.131255159
CES4	-0.089885248	0.08812912	0.131261045
C1orf183	-0.089858919	0.088222646	0.131390517
H1F0	-0.089851409	0.088249341	0.131420446
CLIC2	-0.089813737	0.088383329	0.131610139
C15orf48	0.089793399	0.088455735	0.131708109
NLK	-0.089783105	0.088492403	0.131752856
FOXF2	-0.089747807	0.088618221	0.131923649
CCNH	0.089747206	0.088620366	0.131923649
FOLR1	0.089733164	0.088670459	0.131988354
HECTD3	-0.089723703	0.088704221	0.132028742
SPAG11A	0.089718801	0.08872172	0.132044919
HSPA5	0.089704369	0.088773252	0.13211174
SHD	0.089695883	0.088803565	0.132146978
IPO9	-0.089681868	0.088853647	0.132211624

C5orf35	-0.089676557	0.088872629	0.132229989
LGI3	0.08966579	0.088911124	0.132274504
RETN	0.089664474	0.088915831	0.132274504
PVRL1	-0.089647933	0.088974999	0.132352637
USH1G	0.089644405	0.088987622	0.132354591
TREML4	0.089643852	0.088989605	0.132354591
CCL5	0.089608125	0.089117527	0.132534952
MAGEB4	0.089524685	0.089416864	0.132970195
BCDIN3D	0.089499159	0.089508598	0.133096672
UCP2	0.089484801	0.089560231	0.133163506
CCNL2	-0.089476816	0.089588958	0.133193636
SERPINE2	-0.08947545	0.089593871	0.133193636
NAGS	0.0894697	0.089614561	0.13321445
SSX5	0.089466319	0.089626728	0.133222593
RBM5	-0.089420777	0.089790757	0.133456446
HS3ST2	-0.089377864	0.089945538	0.133676522
C16orf71	-0.089354589	0.090029575	0.133791433
RIMS2	0.089333615	0.09010536	0.133894064
ZNF563	0.0893156	0.090170493	0.133980852
NXF2B	0.089302628	0.090217417	0.134040575
ORAI3	0.089293286	0.09025122	0.134080795
PTH2	0.089283526	0.09028655	0.134123277
FEZF2	0.089245626	0.090423843	0.13431721
NCRNA0016	-0.089201245	0.090584824	0.134546298
SC4MOL	-0.08919191	0.090618713	0.134586596
CHRM4	-0.089186918	0.090636842	0.134603484
C1orf26	-0.089175574	0.090678043	0.134654629
SERPINA5	-0.089140531	0.090805422	0.134833729
LRRC37A	-0.089121437	0.090874886	0.134926813
SLC22A7	-0.089108346	0.090922536	0.134987498
TDRD10	-0.089031504	0.09120264	0.135392545
LRP2	0.089029773	0.091208957	0.135392545
CDO1	-0.089001593	0.091311857	0.13553519
GNPTG	0.088949551	0.091502135	0.135807498
PLRG1	0.088931859	0.091566893	0.135893485
KRTAP5-1	-0.08891862	0.091615375	0.135955304
RNU4ATAC	0.088913299	0.091634866	0.135974097
SIGLEC14	-0.088908564	0.091652214	0.135989706
DUSP27	-0.088882855	0.091746454	0.136119394
KIAA1949	-0.088879971	0.091757029	0.136124942
TMEM151A	0.088872637	0.09178393	0.136154709
COL6A4P2	-0.088867093	0.091804269	0.136174738
GFRA4	0.088854788	0.091849423	0.136231567
PCSK7	-0.088841402	0.091898566	0.136294306
IL17RC	-0.088827969	0.091947901	0.13635732
C12orf24	0.088818475	0.091982783	0.136398011

ST3GAL4	0.088816773	0.091989037	0.136398011
TTR	0.088797147	0.092061183	0.136490593
ZNF468	-0.088795464	0.092067372	0.136490593
NIPAL4	-0.088794195	0.092072038	0.136490593
PXN	-0.088785282	0.092104819	0.136529026
IGSF8	0.088778527	0.09212967	0.136555699
LPPR5	0.088738138	0.092278372	0.136765928
TTC25	-0.088730106	0.092307965	0.1367934
MFSD11	-0.088729379	0.092310645	0.1367934
MADCAM1	-0.088723029	0.092334047	0.136817898
ACVRL1	-0.088716807	0.092356981	0.136841699
TACR2	0.088687163	0.092466318	0.136993508
PCSK4	-0.088682795	0.09248244	0.137003233
TEC	-0.088681656	0.092486642	0.137003233
MYOT	-0.088663718	0.092552864	0.137091133
SNAR-G1	0.08865358	0.092590309	0.137131686
PMS2L2	-0.088652577	0.092594014	0.137131686
H2BFWT	0.088650554	0.092601487	0.137132556
OR4F17	-0.088591256	0.092820764	0.137447061
C15orf39	0.088552572	0.092964038	0.137648982
OPRD1	0.088541688	0.09300438	0.137698477
ZNF600	0.088537267	0.09302077	0.137712506
LIPT1	0.088526141	0.093062029	0.137763347
SH3TC1	-0.088492581	0.093186573	0.137937462
TMEM167A	-0.08848701	0.09320726	0.137957828
SEC14L5	-0.088483888	0.093218855	0.137964737
FAM115C	-0.088479982	0.093233362	0.137975953
PRDXDD1P	0.088462463	0.093298456	0.138055453
TSPYL2	0.088461793	0.093300946	0.138055453
KRTAP5-5	0.088453119	0.093333188	0.138092901
TXLNB	-0.088414837	0.093475596	0.138293329
EN2	-0.088377226	0.09361568	0.138490288
HS6ST1	-0.088370914	0.093639204	0.1385148
PLIN2	-0.088367045	0.093653624	0.13852584
NWD1	-0.088360229	0.093679039	0.138553142
KCNJ1	-0.088336604	0.093767161	0.138673178
DAPK2	0.088318539	0.093834589	0.138762593
RABL2A	-0.088279532	0.093980317	0.138967777
SKINTL	-0.088249871	0.094091252	0.139121486
NPHS2	0.08823243	0.094156533	0.139207672
MNX1	0.088211558	0.0942347	0.139312898
LRRC52	0.088207064	0.094251535	0.139327444
ANKRD9	-0.088201216	0.094273449	0.139348429
SPINT2	-0.088199542	0.094279725	0.139348429
LGR6	-0.088145689	0.094481725	0.139636627
SMCR5	-0.088123436	0.094565298	0.139749771

FLJ42393	0.088113016	0.094604448	0.139797253
IRAK1	0.088107045	0.094626891	0.139813072
WHSC2	0.088106432	0.094629194	0.139813072
CKB	0.088098706	0.094658241	0.139845614
TBX2	-0.088096688	0.094665829	0.13984645
RNU6ATAC	0.088066987	0.094777558	0.140001117
DYRK1B	0.08806139	0.094798625	0.14002185
C12orf23	-0.088058634	0.094808997	0.140022723
ADAD2	-0.088057497	0.094813277	0.140022723
GSTM2P1	-0.088040961	0.094875549	0.140104297
C1QTNF8	0.088027624	0.094925793	0.1401681
EVPL	0.087989642	0.095069008	0.140369165
SLC38A8	0.087982807	0.095094796	0.140396832
SLC9A5	-0.08797287	0.095132302	0.140441794
OTP	0.087964831	0.095162649	0.140476181
SULT2B1	0.087959005	0.095184651	0.140498246
VEGFA	-0.087939008	0.095260192	0.140599327
SLC7A11	0.08785821	0.095565906	0.141035279
FGF23	-0.087856641	0.095571852	0.141035279
GPR52	-0.087855333	0.095576808	0.141035279
CBX1	0.087850569	0.095594857	0.141051461
IL1RN	0.087828488	0.095678561	0.141164509
PRAMEF11	-0.087790292	0.095823491	0.141367865
LOC285768	-0.087747899	0.095984551	0.141585092
LOC646813	0.087747793	0.095984952	0.141585092
C9orf125	0.087710444	0.096127027	0.141784162
HOXD13	0.087692535	0.096195212	0.141874225
DNAJB1	0.087679411	0.096245204	0.141937443
VSIG10L	0.087672939	0.096269867	0.141963303
IGFBP4	-0.087646921	0.096369055	0.142099047
CYP11A1	-0.087619356	0.096474231	0.1422436
NOG	-0.087575009	0.096643629	0.142482815
ACE2	-0.087532416	0.096806554	0.142712452
TBX4	0.087521919	0.096846736	0.142761121
LILRB1	-0.087504355	0.096914009	0.142849714
SUPT16H	-0.087448661	0.097127561	0.143153892
PATE1	0.087413308	0.097263313	0.143343364
C17orf48	0.08739307	0.097341091	0.143447376
COQ2	-0.08738352	0.097377812	0.143490871
CCL17	0.087345335	0.097524746	0.143696753
PCDHA4	-0.087336503	0.097558757	0.143736231
LYPLA1	-0.087308689	0.097665922	0.143880765
CLCA4	0.08730669	0.097673628	0.143880765
LOC645332	0.087305419	0.097678531	0.143880765
CAV3	-0.087296358	0.097713464	0.143921576
TRIM17	0.087282872	0.097765481	0.143987543

BTBD2	0.087262598	0.097843718	0.144092114
APOBEC3A	0.087243691	0.097916726	0.144188968
MAGED4B	0.087226753	0.097982167	0.144274665
PTCD1	0.087215448	0.098025867	0.144328341
SLC35F4	-0.087171799	0.098194729	0.144566276
A2BP1	0.087160457	0.098238644	0.144620239
TSC22D4	0.08713136	0.098351378	0.144775497
CTSC	0.087125946	0.098372366	0.144795688
PARP6	-0.087123878	0.098380382	0.144796786
LOC100189	0.087120636	0.098392953	0.144804585
ZSCAN18	0.087117941	0.098403403	0.144809264
EZR	-0.087115895	0.098411338	0.14481024
GPR55	-0.087103243	0.09846041	0.144871745
TRPV5	-0.087095463	0.098490598	0.144905456
CCDC36	-0.087039216	0.098709062	0.145215009
DUSP21	0.087037539	0.098715581	0.145215009
ALS2CR12	-0.086993347	0.098887499	0.145457164
ITFG3	0.086979011	0.098943323	0.145527372
HLA-DQB1	0.086977337	0.098949844	0.145527372
EFCAB1	-0.086973562	0.098964546	0.145538247
ABCC6P1	-0.086931931	0.099126822	0.145766127
SYNPR	0.086910891	0.099208914	0.145876072
TRIM60	0.086899546	0.099253201	0.145930417
POLR3E	-0.086849216	0.099449868	0.146208779
LEF1	0.086845868	0.099462958	0.14621723
MOCS3	0.086826421	0.09953904	0.146318275
SPC24	0.086820231	0.099563265	0.146340959
HOXC11	0.086818723	0.099569169	0.146340959
SV2B	-0.086808408	0.099609552	0.146389508
ZNF513	0.086790461	0.099679844	0.146482002
TNMD	-0.086763848	0.099784152	0.146624465
CPA3	-0.086734382	0.099899737	0.146783479
VPS18	0.086732321	0.09990783	0.146784539
OR4K14	0.086707453	0.100005468	0.14691715
IGSF5	-0.086663104	0.100179791	0.147162391
KCNE3	0.086655203	0.100210872	0.147186711
C21orf15	0.086655137	0.100211128	0.147186711
GLB1	-0.086652876	0.100220025	0.147188923
OPN1SW	0.086642757	0.100259845	0.147236547
DMRTB1	0.086620531	0.100347355	0.147354193
TAS2R1	0.086597941	0.100436359	0.147471387
SGK2	-0.086596516	0.100441973	0.147471387
METTL11B	0.08658892	0.100471919	0.147504479
C18orf16	-0.086574045	0.100530575	0.147579714
WARS	0.086569138	0.100549931	0.147597248
GSG2	0.08656311	0.100573713	0.147621278

CES1	0.086523979	0.100728204	0.147837143
FUT7	0.086510603	0.100781054	0.147903809
CEACAM6	-0.08650094	0.100819249	0.14794896
MUC15	0.086474464	0.10092396	0.148091709
SDF4	-0.086439023	0.101064261	0.148286654
KRTAP3-1	0.08643667	0.101073579	0.148289401
SEMG1	-0.086422565	0.101129465	0.148360464
SNORA66	-0.086416988	0.101151569	0.14838196
TEX261	-0.086376844	0.101310784	0.148601506
IKBKE	0.08637549	0.101316156	0.148601506
C11orf84	0.08633335	0.101483508	0.148836001
SYTL1	0.086316893	0.101548921	0.148920969
BMF	0.086288044	0.101663674	0.149078276
SEMA3A	-0.08626651	0.101749397	0.149192994
CLDN25	-0.086245799	0.1018319	0.149302974
PRCD	-0.086217579	0.101944397	0.14945691
CLPS	0.086213867	0.101959201	0.14946761
FZD5	-0.086193545	0.102040287	0.149575468
NFRKB	-0.086188719	0.10205955	0.149592695
HCG4	-0.086186208	0.102069571	0.149596373
CPA4	-0.086179028	0.102098239	0.149627377
OR13C5	0.086166356	0.102148849	0.149690532
RBMY2FP	0.086153695	0.102199432	0.149753637
RND2	0.086141438	0.102248423	0.149814401
LRRC30	0.086133789	0.102279005	0.149848186
SLC23A1	0.086110772	0.102371072	0.149972039
TMEFF2	0.086059152	0.10257779	0.150263823
LOC285501	0.086052738	0.102603502	0.150290434
IL8	-0.086025995	0.102710746	0.150436457
GALR3	0.085968241	0.102942662	0.150765047
RG9MTD3	-0.085945974	0.103032183	0.15088506
UBE2NL	-0.085932154	0.103087778	0.150955375
C1QL2	-0.08592239	0.10312707	0.150993385
SCARNA12	0.085921936	0.103128899	0.150993385
ULBP2	-0.085884915	0.103277991	0.151200558
GLYATL3	-0.085853827	0.10340332	0.151369886
NGF	-0.085852455	0.103408852	0.151369886
EDARADD	0.085848493	0.103424833	0.151382151
NUFIP1	0.085814298	0.103562849	0.151573024
C1GALT1C1	-0.085797463	0.103630853	0.151661408
GPNMB	-0.085764227	0.103765209	0.151846876
NKX3-2	0.085756908	0.103794811	0.151879034
NDN	0.085746779	0.103835793	0.151927839
ARF3	-0.085723243	0.103931074	0.152053126
LPCAT4	0.085721856	0.103936691	0.152053126
FAM108C1	-0.085701234	0.104020233	0.152164165

PDCD1	0.085686477	0.104080045	0.152240477
ARMC2	-0.085676413	0.104120855	0.152288986
TMSB15A	0.085669794	0.104147702	0.152317066
ZNF57	-0.085659312	0.104190227	0.152368069
HAL	-0.085652606	0.104217439	0.152396674
TPM1	-0.085646778	0.104241093	0.15241747
NPTX2	-0.085645331	0.104246968	0.15241747
FLNC	0.08563334	0.104295652	0.152477456
PRKRA	0.085629069	0.104312996	0.152491619
GPR32	0.085620602	0.104347388	0.152530698
KIF1C	-0.085601684	0.104424268	0.152631874
HCFC1	-0.085589322	0.104474524	0.152694126
ADCY2	-0.085586861	0.104484534	0.152696253
C5orf27	-0.085585193	0.104491315	0.152696253
SCARNA15	0.085571851	0.104545586	0.152764351
IVNS1ABP	-0.08555742	0.104604314	0.152838952
YJEFN3	0.085547368	0.104645236	0.152887197
RASSF4	0.085545538	0.104652688	0.152887197
NCRNA0018	0.085533019	0.104703673	0.152950463
TBX6	0.085516337	0.104771641	0.153038523
FAAH2	-0.085501673	0.104831421	0.153114613
USP29	0.085492808	0.104867572	0.153156182
KRTAP4-12	0.085454212	0.105025076	0.153374965
FAM9B	-0.085451747	0.105035141	0.153378415
CTNS	0.085436838	0.105096037	0.153452197
ATP1A4	-0.085435605	0.105101078	0.153452197
CCDC65	0.085429176	0.105127345	0.153479296
CYP4F11	-0.085386331	0.105302546	0.153723809
FRMPD2L1	-0.085363275	0.105396919	0.1538503
LOC285847	0.085352958	0.105439172	0.153896001
ENOSF1	-0.085351857	0.105443682	0.153896001
RPS6KC1	-0.085349402	0.105453739	0.1538994
RNF17	0.08532273	0.105563043	0.154043758
MMP23A	-0.085321491	0.105568125	0.154043758
ADI1	0.08526711	0.105791273	0.154358063
C6orf227	0.085251847	0.105853968	0.154438225
FUZ	-0.085247867	0.105870325	0.154450774
KIAA1797	-0.0852198	0.105985708	0.154607777
LOC283761	-0.085217034	0.105997083	0.154613046
LOC201651	-0.085206229	0.106041534	0.154666556
CCDC144NL	0.085203793	0.106051555	0.154669843
WDR88	-0.085199717	0.10606833	0.154682981
C19orf41	0.085196397	0.106081993	0.154691579
CCDC87	-0.085174778	0.106171002	0.154810038
CAB39L	-0.085164884	0.106211756	0.154850188
USP40	-0.085164318	0.106214089	0.154850188

SKIV2L2	-0.085150075	0.106272778	0.15492441
KRT1	-0.085141689	0.106307346	0.15496346
RNF24	0.085109554	0.106439893	0.155145317
SCN2A	-0.085105733	0.106455662	0.155156946
C2orf18	0.085081479	0.106555801	0.155291531
TPTE2	0.085049473	0.106688055	0.155472898
CDRT4	0.084997381	0.106903591	0.155775592
CSNK1A1P	-0.084976379	0.106990588	0.155890954
RIPK4	-0.084970988	0.107012927	0.155912096
MLC1	-0.084957109	0.107070456	0.155984501
CLDN14	-0.084924123	0.107207286	0.156172417
NTS	0.084915386	0.107243551	0.156213818
HCN4	-0.084908634	0.107271584	0.156242973
GYPB	-0.084906785	0.107279257	0.156242973
DSE	-0.084894898	0.107328629	0.156303448
PSG8	-0.084879753	0.107391551	0.156383646
ZNF763	-0.08486724	0.107443564	0.156447947
IFI6	0.084859384	0.10747623	0.15648407
SETD6	0.084839904	0.107557258	0.156590598
MYB	0.08483412	0.107581327	0.15661419
MAGEC1	0.08481935	0.107642806	0.156692236
IL18	0.084817188	0.107651808	0.156693887
MST1	0.084799885	0.107723871	0.156787319
VENTXP1	0.084778287	0.107813875	0.156897106
C1orf200	0.084778004	0.107815058	0.156897106
STRADB	-0.084774438	0.107829922	0.156907271
ALB	-0.084737055	0.107985866	0.157122709
KCNJ8	-0.084730148	0.108014698	0.157153179
PNOC	0.08469417	0.108164982	0.157360334
C16orf7	0.084655733	0.108325718	0.157582664
MAPT	0.0846499	0.108350128	0.157606662
CSN1S2A	0.084645054	0.108370411	0.157624652
KRTAP10-5	-0.084607308	0.10852849	0.15784305
C11orf24	0.084581495	0.108636701	0.157980931
SLAMF9	0.084580909	0.108639159	0.157980931
CLCNKB	0.084569247	0.108688076	0.158040525
HIST1H2BF	-0.084562959	0.108714458	0.158067345
SLC1A6	0.084539786	0.108811729	0.158197225
SNORD22	0.084528807	0.108857839	0.158252709
LPGAT1	-0.084525835	0.108870322	0.158256543
TEX13B	0.084524395	0.10887637	0.158256543
SLC10A5	0.084519164	0.108898349	0.158276939
FAM171A2	-0.084501807	0.108971297	0.158371407
PLEK	-0.084488561	0.109026994	0.15844079
L1CAM	-0.084475394	0.10908238	0.158509711
FSTL4	0.084470176	0.109104335	0.158530048

CYP3A7	-0.08445447	0.10917044	0.158614526
MUL1	-0.084388994	0.109446366	0.159003821
BARX2	-0.084383733	0.109468558	0.159014546
SLC27A3	0.084383458	0.109469718	0.159014546
HDLBP	0.084374599	0.109507101	0.159057248
CIT	0.084368188	0.109534157	0.159081678
C4orf37	-0.084366828	0.109539897	0.159081678
RAD21L1	-0.084291758	0.109857142	0.15953077
GMPS	0.084281619	0.109900043	0.159581433
EHBP1L1	-0.084276362	0.109922292	0.159602104
SNORA26	0.0842549	0.110013169	0.159722407
MKNK1	-0.084246855	0.110047246	0.159760235
CYTL1	-0.084215114	0.110181784	0.159921593
P4HA2	0.084215063	0.110181999	0.159921593
DUSP2	0.084214949	0.110182484	0.159921593
SCCPDH	0.084210441	0.1102016	0.159937681
SLC22A10	-0.084208534	0.110209689	0.159937766
VDR	-0.084204758	0.110225705	0.159949353
OPTN	0.084199374	0.110248547	0.159970842
CSF3R	-0.084169938	0.110373487	0.160140463
SLPI	-0.084138268	0.110508035	0.160323998
SNORA63	0.084120607	0.110583123	0.160421248
ZSCAN12	-0.084101946	0.110662509	0.160524719
LPHN1	-0.084083549	0.110740809	0.160615715
ATP5L2	0.084083418	0.11074137	0.160615715
LRRC37A3	0.084032627	0.110957782	0.160917873
ACSS3	-0.084007298	0.111065826	0.161062836
GPR98	0.084002556	0.111086064	0.161080455
HAUS3	-0.083911929	0.111473384	0.161630319
SCN1B	0.08390606	0.111498506	0.161654976
ZNF396	-0.083858908	0.111700476	0.161936011
BCAS2	0.083829749	0.111825523	0.162102873
SAP30L	-0.083828273	0.111831854	0.162102873
SLC22A12	0.083780648	0.112036334	0.162387452
TMEM200B	0.083765066	0.112103302	0.162468394
GALNT8	-0.08376233	0.112115064	0.162468394
MCHR2	0.08376196	0.112116652	0.162468394
H1FOO	0.083737146	0.112223368	0.162611203
CCDC30	-0.083704669	0.112363161	0.162801918
GPR62	0.083702574	0.112372187	0.16280315
TUBA4A	0.083669407	0.112515098	0.16299834
MICALL1	-0.083615758	0.112746568	0.16331353
HAR1B	-0.083615178	0.11274907	0.16331353
POU3F2	0.083586504	0.112872941	0.163481064
PDILT	-0.083562555	0.112976479	0.163619124
NXF4	0.083553731	0.113014648	0.163662501

ZNF197	-0.083522977	0.113147754	0.163837255
SPAG11B	0.083522048	0.113151775	0.163837255
CILP2	-0.083506013	0.113221227	0.163925899
DPYSL5	0.083476974	0.113347084	0.164096189
C15orf50	-0.083444236	0.113489111	0.164289862
CDC42EP4	0.083440964	0.11350331	0.164298475
COG7	-0.083411581	0.11363091	0.164471224
KRTAP20-2	0.083395824	0.113699383	0.164558374
MMP23B	-0.083363225	0.113841148	0.164751579
RILPL2	0.083326962	0.113999008	0.164968046
OPA3	0.083319379	0.114032038	0.165003854
MYL7	-0.083307871	0.114082184	0.165064422
PEG3	-0.083303247	0.114102339	0.165081589
H19	0.083241364	0.114372313	0.165456476
CDHR2	0.083240046	0.114378072	0.165456476
ADARB2	-0.083230742	0.114418704	0.165503232
NUDT4	-0.083224143	0.114447533	0.165532909
SRPX2	-0.083207499	0.114520272	0.165626087
CNTNAP1	-0.083204237	0.114534527	0.165634675
VSX1	-0.083200957	0.114548868	0.165643386
ENTPD2	0.083176271	0.114656838	0.165787478
SMYD1	-0.083173909	0.114667174	0.165790385
PROZ	-0.083153419	0.114756853	0.165908001
CSN1S1	0.083133811	0.114842728	0.166020102
ZNF230	-0.083073987	0.115105041	0.166387231
P2RY4	-0.083071227	0.115117152	0.166392662
RBM6	0.083030289	0.11529694	0.166640435
ACP2	0.08302056	0.1153397	0.16669014
MAPK1IP1L	-0.082998932	0.115434803	0.166815478
TRIM24	0.082967984	0.115570987	0.167000161
ESR2	-0.082947176	0.115662624	0.167120449
HNF4A	-0.082919952	0.115782603	0.167281519
KIR2DL3	-0.082918071	0.115790898	0.167281519
WNT8A	0.082912349	0.11581613	0.167305834
RLBP1	-0.082907547	0.115837307	0.167324288
CSTA	0.082904906	0.115848956	0.167328979
GABRA6	0.082902054	0.115861536	0.167335013
ATG4D	0.082891806	0.115906747	0.167388171
SLED1	0.082881575	0.115951901	0.167441238
IL13	-0.082870892	0.115999062	0.167497196
FGF22	0.082859053	0.116051345	0.16756054
MUC1	0.082847783	0.116101129	0.167620269
UBA5	-0.082834715	0.116158881	0.167691491
FBXL13	-0.082825745	0.116198535	0.167736578
LOC285629	0.082784704	0.116380098	0.167986494
FUT9	0.082769863	0.116445808	0.168069159

AQP11	0.082748267	0.116541481	0.168195058
SLC36A3	0.082727019	0.11663567	0.168313268
XKR9	-0.082725977	0.116640292	0.168313268
SMO	-0.082721697	0.116659271	0.168328459
HES4	0.0827186	0.116673005	0.168336079
SPATA16	0.082708438	0.116718086	0.168388922
PKDCC	-0.082693357	0.11678501	0.168473269
FLJ32063	-0.082685723	0.116818898	0.168509949
ACTR3B	0.08267148	0.116882149	0.168588977
ATPBD4	0.082664831	0.116911683	0.168619363
PPP1R10	0.082644289	0.117002967	0.1687388
MAGEC3	0.082641919	0.117013505	0.168741779
ESRP1	-0.082630093	0.117066084	0.168805376
PCDH10	-0.082617996	0.117119891	0.168870738
CTCFL	-0.082582265	0.117278929	0.16908219
KCNE2	0.082581234	0.117283524	0.16908219
NT5M	0.082571612	0.117326379	0.169131729
C8orf31	-0.08255196	0.11741395	0.169245716
ZCCHC5	-0.082537547	0.117478208	0.169326085
IFT74	-0.082516714	0.117571137	0.169447763
RNF43	0.082491496	0.117683705	0.169597727
LRFN1	0.082480444	0.117733064	0.169656583
TCEAL7	-0.082476277	0.117751679	0.16967113
FAM46D	0.08246592	0.117797955	0.16972553
PSG2	-0.082462803	0.117811886	0.169733322
GNRH1	-0.082458689	0.117830271	0.16974753
OGFRL1	-0.082438556	0.117920289	0.169864922
SLC17A2	0.082426803	0.117972861	0.169928361
LAD1	0.082422991	0.117989917	0.169940638
SLC35F3	-0.082365044	0.118249423	0.170302086
PRRX2	0.082346705	0.11833164	0.170408172
TAS2R19	-0.082343867	0.118344372	0.170414184
CSRP3	-0.08233043	0.118404647	0.170488652
SLC27A4	0.082328205	0.118414629	0.170490698
TFF3	0.082314423	0.118476484	0.170567424
PCGEM1	0.082302702	0.118529104	0.170630845
PCYT1B	-0.08227153	0.118669146	0.170820097
CYP19A1	0.082257382	0.118732746	0.170899294
KCNJ9	0.082240632	0.118808081	0.17099537
PRKCZ	-0.082236253	0.118827782	0.171011365
FIGLA	0.082229733	0.118857121	0.171041228
GNRH2	-0.082213964	0.118928102	0.171131008
CRH	0.082186869	0.119050135	0.17129423
SLC22A3	0.082181235	0.119075526	0.171318385
HAT1	0.082171061	0.11912138	0.171371977
DNAJC5G	0.082134279	0.119287283	0.171598255

C1orf61	0.082103318	0.119427067	0.17178693
RLN2	0.082085908	0.119505728	0.171887663
NEK10	-0.082027927	0.119767987	0.172246041
RDH10	-0.082025729	0.119777938	0.172246041
STAB1	-0.082025088	0.11978084	0.172246041
NADSYN1	0.082006058	0.11986702	0.172346979
BANP	0.082004106	0.119875863	0.172346979
LY86	0.082003856	0.119876995	0.172346979
NEIL2	-0.081945711	0.120140629	0.172713536
E2F8	0.08192797	0.12022116	0.172816832
STRA8	0.081897917	0.120357672	0.17300058
PIGG	-0.081851784	0.120567462	0.173289622
MAFF	-0.08183913	0.120625055	0.173359888
CXCL11	-0.081829864	0.120667243	0.173408005
GPR83	-0.081827483	0.120678083	0.17341107
LOC100132	0.081824157	0.120693229	0.173420322
RNF126P1	0.081803628	0.120786751	0.173542179
LOC100128	-0.081798809	0.120808715	0.173561215
RTL1	0.081791115	0.120843784	0.173599073
SFTA2	-0.081777723	0.120904845	0.173674264
MST4	-0.081767163	0.120953015	0.173730926
GNB3	0.081751362	0.121025114	0.173821948
IL2RA	-0.081745156	0.121053439	0.173850093
SLC22A13	-0.081723918	0.121150419	0.173976824
SLC11A1	0.081718169	0.121176681	0.17400199
RHBDD2	0.081709203	0.121217648	0.174036857
CLEC4C	-0.081709029	0.121218441	0.174036857
PDE4A	-0.081687788	0.121315537	0.174156569
ARHGEF1	0.081686962	0.121319311	0.174156569
ZNF28	-0.081682707	0.12133877	0.174171947
CCDC37	0.081643556	0.121517922	0.174416534
MYO3A	-0.081627269	0.121592507	0.174511011
AMY1A	0.081621231	0.121620167	0.174535107
LOC100133	0.081619778	0.121626825	0.174535107
ADAM15	-0.081605097	0.121694103	0.174616494
AQP6	0.081603576	0.121701077	0.174616494
FAM157B	-0.081594017	0.1217449	0.174666788
SPATA21	0.081570057	0.121854798	0.174811865
GINS4	0.081563485	0.121884957	0.174842535
LOC100129	0.081518934	0.122089544	0.175123399
RABGAP1L	-0.081489508	0.122224822	0.175304814
ACCSL	-0.081466164	0.122332223	0.175446221
NT5C2	-0.081447101	0.122419982	0.17555944
DHODH	-0.081434601	0.122477555	0.175629357
CNOT6	-0.081431988	0.122489592	0.175633971
CAMK2N2	0.08142305	0.122530772	0.175680326

CD276	0.081421143	0.122539564	0.175680326
C10orf68	-0.081416272	0.122562009	0.175699857
FASTKD2	-0.081401245	0.122631288	0.175786519
C16orf81	-0.081386571	0.122698962	0.175870868
MLLT11	-0.081361061	0.122816687	0.17602694
ORM2	0.081284675	0.123169719	0.176520218
LHFPL3	0.081280972	0.123186854	0.176532071
FZD6	-0.08122744	0.123434759	0.176874603
IL1RAPL1	-0.081216411	0.123485884	0.176935131
C11orf66	-0.08120953	0.123517787	0.17696811
CCL26	0.081181352	0.123648503	0.177142647
TMEM214	0.081169015	0.123705763	0.177211932
LOC388242	0.081165403	0.123722534	0.177223208
GLDC	-0.081150109	0.123793559	0.177312193
LOH3CR2A	-0.081124663	0.123911804	0.177468794
POR	-0.081085847	0.124092339	0.177714579
ADAMTS19	0.081071568	0.124158804	0.177796979
F7	-0.08106236	0.124201683	0.17784179
KLK14	-0.081061012	0.124207957	0.17784179
GABPB2	-0.081054818	0.124236809	0.177870313
SLC6A9	0.0810485	0.124266242	0.177899662
TINF2	-0.08103878	0.124311529	0.177951704
PLD6	0.081017813	0.12440927	0.178078819
PRMT2	0.080989364	0.124541981	0.178255969
KCNC4	-0.080971982	0.12462312	0.178359284
ILF3	0.080957887	0.124688947	0.178439351
LOC100129	0.080956166	0.124696985	0.178439351
C20orf30	0.080947557	0.124737207	0.178484083
SLC1A3	-0.080923723	0.12484861	0.178630654
MGAT4C	-0.080887934	0.125016041	0.178857361
OR4A47	-0.080881411	0.125046573	0.17888819
ALOX12B	0.080866066	0.125118429	0.17897813
MVP	0.080850041	0.125193501	0.179072654
ABLIM3	-0.080838209	0.125248951	0.179139102
TMPRSS9	-0.080783657	0.125504864	0.179492235
CHST14	-0.08075407	0.125643831	0.179678077
RBMY1J	0.080749977	0.125663063	0.179691551
NDFIP1	-0.080748225	0.125671299	0.179691551
LOC151300	0.080745481	0.125684195	0.179697088
KRT27	-0.08072696	0.125771268	0.179808672
NUP35	0.080697078	0.125911852	0.179996736
CHI3L2	0.080683068	0.125977804	0.180078092
RRAS2	-0.080663716	0.126068951	0.180195447
OR1Q1	-0.080656465	0.126103114	0.180231341
MYEF2	0.080654487	0.126112436	0.18023173
LOC441869	0.080650669	0.126130428	0.180244509

C20orf201	0.080610718	0.126318826	0.180499102
LUC7L3	0.080609046	0.126326713	0.180499102
TPH2	-0.080598541	0.126376293	0.18055699
CDC7	0.080596586	0.126385518	0.180557217
OLIG3	0.080591991	0.126407212	0.180575255
FZD9	0.080576636	0.126479718	0.180665872
SLC26A8	-0.080574453	0.126490032	0.180667645
LHFPL5	0.080555669	0.126578779	0.180781439
PRAMEF6	-0.080528716	0.126706208	0.180950457
C7orf54	0.080522412	0.126736026	0.180979011
LYZL4	0.080520647	0.126744377	0.180979011
ATP1A3	-0.080518007	0.126756863	0.180983864
ZNF138	-0.080511095	0.126789571	0.181017586
METTL2B	-0.080498464	0.126849352	0.181089953
VSTM2L	-0.080454044	0.127059761	0.181377331
CDHR3	0.080434918	0.127150442	0.181493768
MYO15B	-0.08042185	0.127212429	0.181569234
RAB36	-0.080380842	0.127407096	0.181834049
APOA4	0.080370233	0.127457498	0.181892947
DISP1	-0.080357545	0.127517794	0.181965954
BTBD11	-0.08035102	0.127548811	0.181997175
IL3	-0.080330322	0.12764724	0.182118267
C13orf27	0.080329329	0.127651965	0.182118267
EMILIN2	0.080318979	0.127701207	0.182175469
PRKAA2	0.08027788	0.127896887	0.182441552
KCNK6	-0.080266265	0.127952233	0.182507429
GPR109A	-0.08023265	0.128112507	0.182722952
NF2	0.080210483	0.128218283	0.182860721
FAM26F	0.080200582	0.128265553	0.182913928
SLC25A17	-0.080198821	0.12827396	0.182913928
BACE2	-0.08018658	0.128332421	0.182984188
TNN	-0.080182277	0.128352974	0.183000393
GLI1	-0.080168736	0.128417677	0.183079536
SMC4	-0.080166422	0.128428735	0.183082194
WDR48	-0.080131135	0.128597471	0.183309616
SLC22A18	0.08012405	0.12863137	0.183344814
EYA1	0.080117216	0.128664075	0.183378305
DEGS2	-0.080107015	0.128712907	0.183434775
LCE5A	0.080097707	0.128757476	0.183485162
GRIK4	-0.080082447	0.128830568	0.183576185
CEND1	-0.080080024	0.128842181	0.183579597
WDR64	-0.080074543	0.12886844	0.183603877
LCN1	0.080072273	0.128879323	0.183606246
LY6G5B	0.080062008	0.128928519	0.183663195
SLC1A7	0.080057547	0.128949909	0.183680526
DNTT	0.080035066	0.129057724	0.183820954

KLF17	-0.080022072	0.129120074	0.183896608
C6orf57	0.079968456	0.129377595	0.1842502
SCARNA8	0.079965829	0.129390219	0.184255003
MAP3K15	0.079960512	0.129415779	0.184278224
HACL1	0.079918267	0.129619012	0.184554416
ARHGAP33	0.079900382	0.129705124	0.184663821
CD19	0.079895929	0.129726568	0.18468115
SCAND2	0.079873607	0.129834121	0.184813251
IGFL3	-0.079872819	0.129837921	0.184813251
SNORA50	0.079842369	0.12998475	0.185009026
CGB8	-0.079834831	0.130021122	0.185047569
UBE2D4	0.07983051	0.130041972	0.185064019
C10orf67	-0.079820321	0.130091149	0.185120774
OR1E2	0.079797875	0.130199532	0.185261767
UGT2A1	0.079793246	0.130221897	0.185280351
LOC158572	-0.079786168	0.130256093	0.185315766
DCP2	0.079782944	0.130271669	0.185324687
MORN4	0.079778449	0.130293393	0.185342352
TULP1	0.079773233	0.130318605	0.185364976
C17orf72	-0.079717857	0.130586484	0.185732741
SRC	0.079708364	0.130632451	0.18578485
DPP10	-0.079678459	0.130777336	0.185977625
OR2A25	-0.07967314	0.130803118	0.186001006
GSTT1	-0.079671061	0.130813195	0.186002055
UNC119	0.079644017	0.13094435	0.18617525
C16orf3	-0.079610596	0.131106577	0.186392596
CCT8L2	0.079594522	0.131184652	0.186490281
DNASE2B	-0.079587424	0.13121914	0.186525994
CSDAP1	-0.079552106	0.131390856	0.186756756
C2orf47	0.079548527	0.131408267	0.186768173
FSIP1	-0.079531625	0.131490512	0.186871729
LAMB4	-0.079522639	0.131534252	0.186920553
HRASLS5	-0.079502621	0.131631738	0.187045742
LPAR2	0.079497359	0.131657373	0.18706882
DEK	0.079454655	0.131865553	0.187351251
DMC1	0.079422866	0.13202069	0.187558285
SDK2	-0.079364995	0.132303469	0.187946614
ECHS1	0.079362188	0.132317199	0.187952712
C9orf122	-0.079354841	0.132353136	0.18799035
PLXDC1	-0.079352021	0.132366933	0.187996539
PTPRH	0.07930594	0.132592517	0.188303499
FAM27L	0.079289828	0.132671463	0.188402181
TMEM138	0.079286908	0.132685774	0.188409069
KRT5	-0.079271601	0.132760817	0.188502187
AGTR2	0.079265933	0.13278861	0.188528208
DHRSX	0.079246436	0.132884254	0.18865055

PODXL2	0.079242728	0.132902451	0.188662934
ACOT6	0.079215651	0.133035386	0.188838183
GPR63	-0.079198452	0.133119871	0.18894464
LOC100302	0.079183077	0.133195437	0.189038422
KCNQ1DN	-0.07917334	0.133243311	0.189092891
TMEM84	0.079169877	0.133260341	0.189103583
C3	-0.079135491	0.13342952	0.189330167
FBXL2	-0.079101035	0.133599212	0.189557446
CD81	-0.079082441	0.133690855	0.18967396
NAE1	0.07907492	0.133727939	0.189713058
GABBR2	-0.07904067	0.133896907	0.189939233
FLJ26850	0.079033262	0.133933474	0.189977574
TDRD6	-0.079026588	0.133966427	0.190010782
CCDC57	0.079015027	0.134023523	0.190078227
APLP1	-0.079009559	0.13405053	0.190102992
FAM78A	-0.078986289	0.134165527	0.190252525
TPPP2	-0.078963069	0.134280351	0.190375317
C15orf17	0.078961875	0.134286256	0.190375317
FCRLB	-0.078961077	0.134290205	0.190375317
RAET1E	-0.078961046	0.134290357	0.190375317
MGC15885	-0.078933665	0.134425863	0.190553852
HSPA7	-0.078906842	0.134558706	0.190726329
HOXC5	0.078905231	0.134566691	0.190726329
NCRNA0018	0.078898834	0.134598389	0.19075768
GLRB	-0.078890765	0.134638379	0.190791468
ITM2B	-0.078890158	0.13464139	0.190791468
OS9	0.078882	0.134681834	0.190835198
ACTG1	0.078878499	0.134699189	0.190846211
C10orf93	-0.078870015	0.134741266	0.190892246
ZNF614	-0.078861516	0.134783424	0.190938391
SYT16	-0.078844969	0.13486553	0.191041114
GABRR1	0.078792916	0.135124072	0.191393733
RBM39	-0.078775963	0.135208359	0.191499499
PPAPDC1B	-0.078769902	0.135238502	0.191528571
BRSK1	0.07875724	0.135301492	0.191604154
GAD2	0.078739024	0.135392152	0.191718907
PADI4	-0.078695479	0.135609055	0.192012395
PTPRVP	-0.078689643	0.13563815	0.192039937
SDC1	-0.078684884	0.135661873	0.192059871
LOC286238	-0.078641573	0.135877947	0.192352098
NCRNA0011	-0.078594536	0.136112906	0.192671016
PPP3R2	-0.078584285	0.13616415	0.192729855
DNAJB7	-0.078569835	0.136236416	0.192818437
WARS2	0.078535262	0.136409433	0.193049592
SCAMP4	0.078506447	0.136553766	0.193240123
LOC145474	0.078485001	0.136661263	0.193378503

FLJ30679	-0.078473687	0.136718	0.193445042
RAB35	-0.078470758	0.136732691	0.193452084
BTF3L1	0.078450778	0.136832942	0.193580168
TAF1C	-0.078365169	0.137263117	0.194174952
F2	0.078361851	0.137279811	0.194184774
C9orf53	0.078346728	0.137355915	0.194278626
PILRB	-0.078315426	0.137513545	0.194487767
ZNF177	-0.07830236	0.137579383	0.194566195
PTPLA	-0.078300544	0.137588537	0.194566195
CCDC53	0.078293428	0.137624402	0.194603094
DPYS	-0.078286588	0.13765889	0.194638039
LOC91149	0.078278426	0.137700046	0.19468241
KRT71	-0.078250468	0.137841101	0.194868
C5orf40	-0.078232662	0.137930993	0.194981242
FNDC7	0.078196467	0.138113862	0.19522589
IL31RA	0.078171313	0.138241055	0.19539181
IFITM2	0.07816847	0.138255437	0.19539827
RIOK2	0.07815573	0.1383199	0.195475504
ZNF3	0.078124089	0.138480096	0.195688008
PXMP2	0.07809226	0.138641383	0.195902026
RBM38	0.07808298	0.13868844	0.195954613
IL3RA	0.078025527	0.138980017	0.196352657
MEIS3	-0.078021872	0.13899858	0.196364951
CCDC152	0.07801825	0.139016982	0.196377018
MTMR11	0.07800242	0.139097417	0.196476704
SPP2	-0.077997291	0.139123491	0.196499598
TMEM106A	-0.077986422	0.139178747	0.196558896
RERGL	-0.07798515	0.139185215	0.196558896
PTPN7	0.077982426	0.139199068	0.196564522
ARHGAP36	-0.077972248	0.139250834	0.196623678
SCARNA3	0.077961965	0.139303145	0.196683597
ARGFX	0.077937081	0.139429806	0.196848475
FAM120AO	0.077930143	0.139465138	0.1968844
PMCH	0.077918415	0.139524871	0.196954764
TH	0.077865756	0.139793338	0.197319748
GPN3	0.077831767	0.139966828	0.19755063
USP39	0.077811757	0.140069048	0.197680893
TRIM49	0.077796257	0.140148262	0.197778673
FBXO27	-0.077783746	0.14021223	0.197850462
LMCD1	0.077782354	0.140219349	0.197850462
GNAI2	-0.077780479	0.140228936	0.197850462
PRDM11	-0.077766001	0.140302991	0.197940923
MAP3K4	-0.077759734	0.14033506	0.197972142
ZNF682	0.077755535	0.140356548	0.19798843
LOC100132	-0.077747651	0.140396898	0.198031321
TRPM3	-0.07774178	0.140426955	0.19805969

RGS12	0.077734725	0.140463078	0.198096607
CASQ1	-0.077730162	0.140486444	0.198115531
SERPINI2	0.077685685	0.140714362	0.198422894
BTBD17	0.077681304	0.140736828	0.198440522
GUCY2C	0.077676579	0.140761066	0.198460646
BEST3	-0.077641091	0.140943172	0.198703332
LCTL	-0.077639057	0.140953615	0.198703988
CASP1	-0.077633743	0.140980897	0.198728381
CTSE	-0.077624554	0.14102809	0.19877775
GPR137C	-0.077623037	0.141035883	0.19877775
AG2	-0.077620979	0.141046455	0.198778583
PHKA2	0.077614593	0.141079261	0.198810748
SLC5A9	0.077612279	0.141091152	0.198813436
ZSCAN5A	0.07758432	0.141234868	0.199001867
NOSTRIN	-0.077557176	0.1413745	0.199184518
LEFTY2	0.077552826	0.141396885	0.199197859
C6orf167	-0.077551449	0.141403974	0.199197859
LOC441666	0.07753625	0.141482215	0.199293982
SIM2	-0.077511207	0.141611209	0.199461576
PPP2R3A	-0.077468598	0.141830891	0.199756873
FCRL5	-0.077464736	0.141850815	0.199770807
ANP32E	0.077455526	0.141898339	0.199823605
NPFFR1	-0.077427202	0.142044566	0.200001256
FAM175A	0.0774272	0.142044578	0.200001256
ARMC5	0.07742223	0.142070246	0.200023256
IL1F9	-0.077416109	0.142101868	0.200053634
ELMO1	-0.077410762	0.142129492	0.20007838
KRTAP5-11	0.077408345	0.14214198	0.200081816
CPLX4	0.077399309	0.142188682	0.200133409
NRG1	-0.07739343	0.142219071	0.200162036
KRTAP4-1	0.077387848	0.142247931	0.200188505
PKM2	0.077384655	0.142264438	0.200197589
SLC6A19	0.07738159	0.142280286	0.200205744
PRSSL1	0.077358945	0.142397427	0.20035642
ATAD2	0.077356513	0.142410013	0.200359972
OR9A2	-0.077338827	0.142501559	0.200474608
CMKLR1	-0.077331555	0.142539211	0.200513412
USP13	-0.077313445	0.142633017	0.2006312
SESN2	-0.077262401	0.142897666	0.200985238
NR1I3	0.077259773	0.142911302	0.200985238
PGLYRP1	-0.077259063	0.142914986	0.200985238
PATL2	-0.077255857	0.142931626	0.200994445
TRIM13	-0.077248835	0.142968069	0.201031497
HFM1	0.077227401	0.143079351	0.201172909
PABPC1L2B	0.077225574	0.14308884	0.201172909
MPL	-0.077218513	0.143125518	0.201210271

TRPV4	-0.077211786	0.143160463	0.20124519
MIPOL1	-0.077199172	0.143226016	0.201323131
NRADDP	0.077185182	0.143298741	0.201411139
SDR16C5	-0.077182535	0.143312503	0.201416267
FAM71F1	0.077174079	0.14335648	0.201463856
PRAMEF9	-0.077166626	0.143395249	0.20149034
LOC389033	-0.077166566	0.143395561	0.20149034
MS4A6A	-0.077162783	0.143415243	0.201503779
CHD4	-0.077101003	0.143736943	0.201941533
LOC157381	-0.07709557	0.143765261	0.201967069
DNAJA2	-0.077089078	0.143799104	0.202000364
ITPRIPL1	-0.077081793	0.143837088	0.202017419
IL1B	-0.077080984	0.143841305	0.202017419
ACMSD	-0.077080913	0.143841677	0.202017419
MOBKL2C	-0.077045471	0.14402658	0.20226284
LOC100190	-0.077042685	0.144041126	0.202269005
SLC5A7	-0.077019786	0.144160696	0.202410303
C10orf71	-0.077019522	0.144162075	0.202410303
SLC2A9	-0.077001979	0.144253732	0.202524714
TMEM130	-0.076988557	0.144323888	0.202608927
PLEC	-0.076986528	0.144334495	0.202609534
C12orf29	-0.076980235	0.144367403	0.202635592
ZNF607	0.076979086	0.144373408	0.202635592
LOC100130	0.076946994	0.144541305	0.202856947
SCGB1A1	0.076939495	0.144580555	0.202897735
MDS2	0.076887499	0.144852949	0.203265676
TTC7A	-0.076881279	0.144885561	0.203297115
L3MBTL	0.076848792	0.145055984	0.203521905
CCNI2	0.076828418	0.14516294	0.203657622
RRAGA	-0.076808466	0.145267737	0.203790291
CD47	-0.076787946	0.145375581	0.203927217
LOC100101	-0.07676833	0.145478728	0.204057534
ETFA	-0.076686571	0.145909247	0.204646993
ZNF287	-0.076676032	0.145964814	0.204710512
IL2	-0.076659984	0.146049459	0.204814799
SPATC1	-0.076652751	0.146087617	0.204853886
ZNF474	-0.076617928	0.146271454	0.205097234
EML4	-0.07657049	0.146522168	0.205434313
PSG4	-0.076555629	0.146600776	0.205530056
PSG6	-0.076549447	0.146633485	0.205561443
C6orf222	0.076546614	0.146648478	0.20556799
C1orf230	0.076542853	0.146668381	0.20558142
DDX11L2	0.076530063	0.146736088	0.205661847
SLC25A35	0.076507143	0.146857472	0.205817491
REM1	-0.076497759	0.146907192	0.205872685
DCAF4L1	-0.076461449	0.1470997	0.206127957

KRT77	-0.076435865	0.147235457	0.206303674
ATP13A5	-0.076411343	0.147365666	0.206471594
SCO1	-0.076406509	0.147391342	0.206493041
TMEM229A	-0.076373061	0.147569112	0.206726495
HNRNPA2B	0.076371251	0.147578739	0.206726495
DCAF4	0.076356602	0.147656651	0.206821086
MCEE	-0.076340969	0.147739829	0.206923039
FAM92B	0.076329366	0.147801592	0.206994987
KIAA0748	-0.076291285	0.148004429	0.207264483
INS	0.076274988	0.148091299	0.207371553
PLXNA1	-0.076270175	0.148116962	0.207392905
ACY1	0.076257378	0.148185207	0.207473875
TNF	-0.076228344	0.148340145	0.20767082
C8orf86	-0.076227111	0.148346728	0.20767082
CDH26	-0.076195478	0.148515673	0.207892713
GZMA	0.076188423	0.148553376	0.207929453
ITIH4	-0.076186659	0.148562801	0.207929453
CASP12	-0.076177059	0.148614115	0.207983284
TMC5	0.076175556	0.14862215	0.207983284
MGC34034	0.07616085	0.148700788	0.20807871
ARL4D	0.076158676	0.148712415	0.208080358
RNASE12	-0.076120736	0.148915445	0.208349802
C2CD4B	-0.076102979	0.14901054	0.208468206
DAB2	-0.076094968	0.149053461	0.208513605
C1orf163	-0.076076999	0.149149761	0.208633665
ACOT4	0.076064667	0.149215875	0.208711487
RAVER1	0.076059235	0.149245006	0.208737573
THBS3	-0.076052981	0.149278553	0.208769831
ZAR1L	0.076046085	0.149315546	0.208806903
TNFRSF14	0.076015466	0.149479888	0.209022045
LOC646498	0.075998378	0.149571663	0.209135693
LPPR2	-0.07596132	0.149770845	0.209399493
SPATA5L1	0.075935931	0.14990742	0.20957573
ZNF556	0.07592932	0.149942996	0.209610751
B3GALNT1	-0.075926888	0.149956086	0.209614336
PACRG	-0.07588069	0.150204909	0.209947414
OR4A16	0.075874871	0.150236268	0.209963325
KIAA1755	-0.075874666	0.150237378	0.209963325
CSMD1	0.075858019	0.150327127	0.210074011
CNGA2	-0.075832517	0.150464699	0.210251507
HES2	-0.075821855	0.150522244	0.21031716
SGSM2	-0.075799461	0.150643162	0.210471346
KCNA4	0.075780329	0.150746525	0.21058321
PCID2	0.075779123	0.150753046	0.21058321
DSC2	0.07577877	0.15075495	0.21058321
ANKRD49	-0.075772506	0.150788808	0.210615732

CRYBB1	0.07575745	0.150870207	0.210714648
HTR2C	0.075743881	0.150943591	0.210802358
PAGE3	0.07572584	0.151041209	0.210923895
SH2B1	-0.075722677	0.151058325	0.210933006
AMTN	0.075714375	0.151103264	0.210980965
LYL1	0.075710274	0.151125469	0.210996912
DPM1	0.075708352	0.151135875	0.210996912
FGF16	-0.075703613	0.151161539	0.211017949
C16orf90	0.075700148	0.151180303	0.21102935
GFRA3	0.07568484	0.151263229	0.211130306
SERPINB7	-0.075681637	0.151280583	0.211139731
FLJ43950	-0.07565385	0.151431206	0.211335141
HRG	-0.075639342	0.151509894	0.211430141
COL20A1	0.075603346	0.15170526	0.211687938
GRIK3	-0.075591687	0.15176858	0.211761457
OSBPL2	-0.075579671	0.151833861	0.2118377
ELL2	-0.075575914	0.151854277	0.211839123
FLJ10038	0.075575064	0.151858894	0.211839123
KLRC2	0.075573598	0.151866864	0.211839123
MAFG	0.075571654	0.151877429	0.211839123
TMEM88B	0.075541542	0.152041145	0.212052622
PTGDS	-0.075533251	0.152086251	0.212100679
IFNA21	0.075529206	0.152108258	0.212116516
MIDN	-0.075509069	0.152217856	0.212254489
INPP5J	0.075477149	0.152391705	0.212482027
VAX1	0.075473422	0.15241201	0.212495461
DOT1L	-0.075463354	0.152466883	0.212557086
EML2	-0.075436821	0.152611561	0.212743892
TMEM14E	-0.075415676	0.152726933	0.212889821
BAIAP2L2	0.075410875	0.15275314	0.212911449
SCN5A	-0.075398557	0.152820387	0.212990273
RFX4	0.075386059	0.152888645	0.213070494
TRDMT1	-0.075382262	0.152909385	0.213084487
PVRL4	-0.075353716	0.153065386	0.213286955
CDC5L	0.075342974	0.153124122	0.213353871
B3GNT4	0.075340779	0.153136122	0.213355664
TMEM150B	0.075293576	0.15339444	0.213700613
OR2T10	0.075274424	0.15349934	0.213831796
OR2AG1	-0.075269231	0.153527792	0.21384219
UCHL1	0.075269142	0.153528277	0.21384219
LOC100130	-0.075262418	0.153565125	0.213878556
PAQR7	0.075259485	0.153581204	0.213885991
TRIM41	-0.075255243	0.153604453	0.213903412
LOC389493	0.075243869	0.153666813	0.213975288
OVCH1	-0.075239641	0.153689995	0.213986601
C14orf68	-0.075238468	0.153696427	0.213986601

PTCHD1	-0.075226795	0.153760453	0.214060778
DYNLRB2	0.075223056	0.153780968	0.214074372
KCNK3	-0.075218522	0.153805839	0.21409403
BRCA1	0.075208238	0.15386228	0.214157626
RPL13AP17	-0.075185703	0.153986001	0.214314851
OR7E156P	0.075160423	0.154124879	0.21449315
C10orf96	0.075154772	0.154155941	0.214521387
IL5	0.075141176	0.154230681	0.214610398
C1orf170	0.075134831	0.154265575	0.214643956
CYP2C18	-0.075125405	0.15431742	0.214701091
FGF21	-0.075110211	0.154401015	0.21480239
NCRNA0002	-0.075096011	0.154479176	0.214896116
GGTA1	0.075091946	0.154501556	0.214912236
C21orf81	0.075086491	0.154531589	0.214939
RNF214	-0.075071727	0.154612904	0.215037083
OLFML3	-0.075067433	0.154636564	0.21505497
DPY19L2P1	0.075056068	0.154699187	0.215127038
ADAM22	-0.075047042	0.154748933	0.215181189
TREM1	0.075035663	0.154811674	0.215253402
TECTB	-0.075014647	0.154927591	0.215381047
LY6G6E	0.075013835	0.154932068	0.215381047
GPD2	-0.075013137	0.154935922	0.215381047
PSPC1	0.074961257	0.155222365	0.215764179
GUCY2F	-0.074956194	0.155250341	0.215788006
KIAA0174	0.074934565	0.155369899	0.215939112
LCE3E	-0.074923787	0.155429503	0.216006878
OR4C6	0.074881958	0.155660977	0.216313471
SRI	0.074872182	0.155715115	0.216373606
OR2L13	-0.074845732	0.155861661	0.216562128
FAM136B	0.074809047	0.156065081	0.216829642
CYLC2	0.074787334	0.156185577	0.216981916
PLAG1	0.074771269	0.156274776	0.217090692
MS4A15	0.074754768	0.156366435	0.21720287
MRPL32	0.074737349	0.156463233	0.217322171
SOD3	-0.074721959	0.156548798	0.217425853
SNORA47	0.074691683	0.15671723	0.217644604
IL34	-0.0746853	0.156752753	0.217678758
TAGLN	-0.074681948	0.156771411	0.217689489
CARD14	0.074676873	0.156799669	0.2177036
PSCA	0.074674906	0.156810616	0.2177036
SLC23A3	-0.074674232	0.156814368	0.2177036
SLC6A4	0.07467154	0.156829363	0.21770924
CSH2	0.074668562	0.156845945	0.217717083
CBFA2T2	-0.074659743	0.156895063	0.217756767
KPNB1	-0.074659502	0.156896402	0.217756767
BTD	-0.07465689	0.156910951	0.217761784

GPT2	-0.074653697	0.156928739	0.217771294
GJB1	0.074641413	0.156997187	0.2178511
UNC5CL	0.074624457	0.157091702	0.217967063
C18orf34	-0.074536372	0.157583387	0.21863405
FAM109B	-0.074484664	0.157872561	0.219019997
SLC17A1	0.074470722	0.1579506	0.219111469
RBMY1A3P	0.074468954	0.1579605	0.219111469
TLX3	0.074440442	0.158120186	0.219317698
TTLL12	0.074431657	0.158169413	0.219370699
SDCBP	-0.074396575	0.158366115	0.219628216
LOC158376	-0.074378332	0.158468474	0.219754868
STK11	0.074355643	0.158595846	0.219916187
PRB2	0.074350219	0.158626306	0.21994311
TAS2R42	-0.074327234	0.158755443	0.22010684
SCGB2A1	0.074321326	0.158788646	0.22012861
RHBDL2	-0.074320507	0.158793252	0.22012861
BCKDHA	-0.074317684	0.158809119	0.220135283
ARAF	0.074315315	0.158822435	0.220138419
LOC100128	0.074304188	0.158884996	0.22020045
KIAA0368	-0.074303422	0.158889303	0.22020045
NCRNA0015	0.07429469	0.158938414	0.220253184
LOC144742	-0.074213399	0.159396151	0.220866569
C2orf43	-0.074212145	0.159403223	0.220866569
EIF4B	0.074205159	0.159442609	0.220905772
OR3A3	0.07418631	0.159548905	0.221023435
HSFY2	-0.074186164	0.159549731	0.221023435
HAPLN1	0.074163827	0.159675772	0.221180998
FAM119B	-0.074162071	0.159685684	0.221180998
ECSCR	0.074079744	0.160150902	0.221809946
SERPINE3	-0.074034301	0.16040813	0.222150759
C9orf98	0.07402313	0.160471412	0.222222944
SBK1	-0.074005185	0.160573104	0.222348308
MAP4K4	-0.073970945	0.160767272	0.222601699
SNORA52	0.073953494	0.160866306	0.222723338
FN3K	0.073932713	0.160984293	0.222871199
SPATS2L	0.073899503	0.161172979	0.223098601
OR8G2	0.073898212	0.161180319	0.223098601
C6orf132	-0.073897888	0.161182158	0.223098601
FDFT1	0.073883124	0.161266099	0.223199274
DOLPP1	0.073858789	0.161404531	0.223375346
KHDC1L	0.073845379	0.161480849	0.223465437
RBM11	-0.073842198	0.161498959	0.22347497
CD5L	-0.073838856	0.161517989	0.223485774
RHEB	0.073834411	0.161543294	0.22350526
BST1	-0.073830287	0.161566778	0.223522222
PPIL6	0.073824979	0.161597005	0.223548511

FLJ41941	0.073803503	0.161719359	0.223702232
KRT33B	-0.073768331	0.161919882	0.223964054
ADRA2C	0.073745232	0.162051678	0.224130785
NKIRAS1	-0.073739563	0.162084038	0.224159973
DDX10	0.073707641	0.162266337	0.224396507
ZNF257	0.073702552	0.162295415	0.224421135
ROD1	-0.073690426	0.162364714	0.224501373
IER5	-0.073680455	0.162421709	0.224564588
ELMOD2	-0.073664371	0.162513687	0.22467616
C9orf123	0.073662257	0.162525778	0.224677278
HSP90AA1	0.073649441	0.162599101	0.224763039
LOC285370	0.073643685	0.162632035	0.224792961
MAFA	-0.073590862	0.162934546	0.225195466
ATP2C2	-0.073588302	0.162949219	0.225200117
AP3D1	-0.073560111	0.163110847	0.225407849
PPARD	-0.073550124	0.163168131	0.225467706
ZNF285	0.073548613	0.163176804	0.225467706
PGBD4	-0.073536017	0.163249081	0.225551925
C15orf60	0.073525144	0.163311493	0.225622504
CSH1	0.073507767	0.163411273	0.225744694
MMD	0.073499238	0.163460261	0.225796706
MAEA	-0.073495919	0.163479331	0.225807385
SRCIN1	-0.073467994	0.163639823	0.22601339
FZD7	-0.073454388	0.163718062	0.226102908
EFCAB3	0.073452775	0.163727343	0.226102908
CRTC1	-0.07343819	0.163811246	0.22620309
SEL1L2	-0.073432203	0.163845701	0.226234981
TRIM21	-0.073425589	0.163883767	0.226258829
AACSL	0.073425254	0.163885695	0.226258829
FGF19	-0.073414663	0.163946664	0.226327313
SNORA32	0.073412314	0.163960187	0.226330292
NAPSB	-0.073406245	0.163995132	0.226362841
GTSF1L	0.073393678	0.16406752	0.226447063
ADH7	-0.073372067	0.16419205	0.226603235
CCDC28A	0.073361815	0.164251149	0.226669089
SH2D7	-0.073303131	0.164589752	0.227120629
OR8D1	0.073294367	0.164640365	0.22717473
GPR1	-0.073275471	0.164749536	0.227309617
SIGLEC15	-0.073245301	0.164923944	0.227534489
SPACA4	-0.073241838	0.164943974	0.227546359
LOC400804	-0.073216955	0.165087939	0.227729189
C1D	0.073198027	0.165197513	0.227864557
KCNK2	-0.07319098	0.165238323	0.227905063
PIK3CD	-0.073169328	0.165363755	0.22806227
SLAMF6	-0.073166	0.165383044	0.228073078
GK2	0.073163328	0.165398526	0.228078635

SIGLEC7	-0.073149585	0.165478194	0.228172695
TKTL1	0.073144513	0.165507602	0.228191625
CYP2C9	-0.073143265	0.165514839	0.228191625
TF	0.073140783	0.165529228	0.228195665
KBTBD10	-0.073135937	0.165557333	0.228218612
CEP72	0.073126128	0.165614232	0.228281246
UGT1A10	0.073124149	0.165625716	0.228281274
QRFP	0.073112267	0.165694662	0.228360498
RAD51L1	-0.07309838	0.165775267	0.228452499
CXADRP2	-0.073096814	0.165784359	0.228452499
STXBP5L	0.073093452	0.16580388	0.22846359
MXD4	0.073088696	0.165831498	0.228485837
GAL3ST3	0.073083179	0.165863536	0.228514169
PRSS35	-0.073049719	0.166057954	0.228766198
ARIH2	0.073036419	0.166135283	0.228856897
LOC442459	0.073030398	0.166170295	0.228889295
SH3BGR	-0.072980583	0.16646021	0.229272777
FBXO48	-0.072965472	0.166548229	0.229378145
FMO5	-0.072935146	0.166724974	0.229605688
AGXT	-0.072916464	0.166833921	0.229739837
CT47A9	-0.072874602	0.167078255	0.230060391
NCRNA0015	0.072850426	0.16721948	0.230238934
UCP1	0.072837355	0.167295874	0.230328194
GRIA1	-0.072828358	0.16734847	0.23038468
GGT1	0.072815073	0.167426155	0.230475694
PLAC4	-0.072802805	0.167497922	0.230558551
CD97	0.072795021	0.167543471	0.230596808
IL23A	-0.07279221	0.167559919	0.230596808
CAD	0.072792119	0.167560453	0.230596808
C10orf108	-0.072785406	0.167599745	0.230625189
TAS2R40	-0.072784638	0.167604236	0.230625189
EN1	0.072772957	0.167672621	0.230703347
MUC7	-0.072767939	0.167702004	0.230727835
CYP27A1	0.072748789	0.16781418	0.230866219
HYAL4	0.072732572	0.167909212	0.230981001
KCP	0.072709524	0.168044347	0.23115093
RAB7L1	-0.072695699	0.168125447	0.231246514
HTRA1	-0.072670661	0.168272396	0.231432649
CAPRIN1	-0.072654637	0.168366492	0.231546073
HIST1H2BB	0.072648401	0.168403121	0.231580455
FLJ23867	-0.072644008	0.168428929	0.231599953
PEX11A	-0.072641424	0.168444105	0.231604829
IL18BP	0.072637551	0.168466865	0.231620132
EGFL6	-0.072631855	0.168500335	0.231650157
NEUROD6	-0.072624377	0.16854429	0.23169459
MX1	-0.072544354	0.169015155	0.232325843

SFTPC	0.072524208	0.169133849	0.232472953
TSPAN19	0.07250047	0.169273792	0.232649246
CRYM	0.072498096	0.169287792	0.232652431
LOC100287	0.072467222	0.16946994	0.232886686
PIK3R6	0.072436435	0.169651724	0.233120409
TMOD4	0.072422069	0.169736597	0.233220942
ZNF599	-0.072313934	0.170376469	0.234083986
SLC35B3	0.072305715	0.170425175	0.234134752
CEBPB	0.07230344	0.170438664	0.234137131
ADD2	-0.072254015	0.170731806	0.234523652
ZC3H8	-0.072240265	0.170813425	0.234619585
MYADML2	0.072215326	0.170961534	0.234806824
LMF1	-0.072206118	0.171016244	0.234865767
SNORA84	0.072195615	0.171078665	0.234924981
POM121L2	-0.072194894	0.171082952	0.234924981
APOBEC3B	0.072190695	0.171107911	0.234943054
NHLH1	0.072170788	0.17122628	0.235089374
ZNF574	0.072132227	0.171455746	0.235388196
HMGCL	0.072091315	0.171699448	0.235706522
PRPS1	-0.072088609	0.171715575	0.235712412
STRN4	0.072077778	0.17178014	0.235784788
TBX19	0.072075285	0.171795005	0.23578894
FLJ14107	-0.072071428	0.171818002	0.235804253
C6orf176	-0.072031309	0.17205736	0.236116478
LOC149620	0.072024292	0.172099249	0.23615769
ASB10	-0.07201995	0.172125173	0.23617699
DSG4	-0.072002334	0.172230385	0.236305071
PNLIPRP2	0.071972986	0.172405766	0.236529404
BCAS3	-0.071961188	0.172476304	0.236609878
WNT11	-0.071950801	0.172538426	0.236678795
FCGR2A	-0.071947299	0.172559379	0.23668059
CNNM3	-0.071946609	0.172563503	0.23668059
GOLPH3L	0.071939698	0.172604849	0.236720996
C6orf201	-0.071927252	0.172679332	0.236806838
PRND	-0.071925161	0.172691849	0.236807697
JPH3	-0.071921065	0.172716364	0.236825005
GABPB1	-0.071886858	0.172921229	0.237089589
GABRB2	-0.071877325	0.172978354	0.237151584
ATP10B	-0.071848772	0.173149532	0.237369927
DSC3	-0.0718365	0.173223141	0.237454492
HCLS1	-0.071827344	0.173278083	0.237513457
CYP1A2	-0.071822358	0.173308	0.237538116
KCNN1	0.071801712	0.173431939	0.237691629
PAICS	-0.071793587	0.173480732	0.23774214
PTPRQ	-0.071743638	0.17378092	0.238137138
TSPYL6	-0.071718831	0.173930149	0.238325231

ZNF185	-0.071658214	0.174295195	0.238808999
LOC100133	-0.071634078	0.174440703	0.238991924
LHX1	0.071569776	0.174828806	0.239507166
UBXN10	-0.071567074	0.17484513	0.239513053
CER1	0.071552971	0.174930339	0.239609417
MKRN2	-0.071551449	0.174939539	0.239609417
RAB11B	0.071514158	0.175165014	0.239901744
DCST1	-0.071506984	0.175208415	0.239944683
HTRA3	-0.071493186	0.175291911	0.240042523
KLHL32	-0.071483519	0.175350432	0.24010615
SNORA7B	-0.071448534	0.175562324	0.240379765
ZNF541	-0.071444234	0.17558838	0.240398913
C13orf39	-0.071425865	0.175699725	0.24053482
SNORA71D	0.071400716	0.175852251	0.240727082
NPPC	-0.071395998	0.17588088	0.240749723
ZNF761	-0.07136199	0.176087316	0.241015732
ZDHHC13	-0.071359375	0.176103192	0.241020896
CR1L	-0.071350831	0.176155092	0.241075361
MT1H	0.071338096	0.176232461	0.241136934
TARS	0.071338055	0.176232715	0.241136934
PHACTR1	-0.071337447	0.176236408	0.241136934
WFS1	0.07132506	0.17631169	0.241223366
CHST1	-0.071299134	0.17646934	0.24142247
GSG1L	-0.071286291	0.176547473	0.241512772
COMT	0.071266467	0.176668124	0.241661219
HOXA3	0.071241738	0.176818715	0.241850598
CHD7	-0.07122601	0.17691454	0.241965048
CGB1	-0.071217083	0.176968953	0.242022847
GLOD5	0.071197503	0.177088331	0.242169478
DDX24	-0.071192514	0.177118756	0.242194454
KRT2	-0.071181436	0.177186331	0.242270221
GALNTL5	0.071143353	0.177418793	0.242571417
GGT3P	0.071132807	0.177483205	0.242642825
TMEM171	-0.071130648	0.177496391	0.242644194
GPBAR1	-0.071119919	0.177561944	0.242717147
DENND1A	0.071115647	0.177588049	0.242736169
ASAP3	-0.071106012	0.17764694	0.242800001
SUMO1P1	0.071099817	0.177684811	0.242835095
C19orf36	0.071097757	0.177697402	0.242835638
TEX12	0.071089857	0.177745708	0.242884985
PLA2G2F	-0.071079821	0.177807091	0.242952194
RAB40AL	0.071041613	0.178040919	0.243255001
GAD1	0.071024931	0.178143079	0.243377885
PCSK2	-0.071021232	0.17816574	0.243392146
IFNK	-0.070965857	0.178505206	0.243839164
C10orf82	0.070951526	0.178593135	0.243927268

C16orf70	-0.070951352	0.1785942	0.243927268
NBAS	0.070948407	0.178612273	0.243935223
INPP5F	-0.070923127	0.178767477	0.244130447
ITPKC	0.070915884	0.178811963	0.244174454
LOC390858	-0.070912584	0.178832237	0.244185397
KCNG1	-0.070898085	0.178921316	0.24429028
CBX2	0.070850551	0.1792136	0.244672574
KCNN4	0.070795456	0.179552819	0.245106231
AP2A2	0.070794964	0.179555853	0.245106231
AKR1CL1	-0.07078192	0.179636229	0.245199143
ASH2L	-0.070761825	0.179760114	0.245351427
FAM155B	0.070732456	0.179941292	0.245581094
LDHB	-0.070730551	0.179953046	0.245581094
CXCL10	-0.070725813	0.179982287	0.24560417
BTBD6	-0.070719924	0.18001864	0.245636946
SPINK2	0.07071458	0.180051629	0.245665126
CRLF2	-0.070687088	0.180221421	0.24587814
KLHL12	-0.070685304	0.180232442	0.24587814
SH2D4B	-0.070669269	0.180331539	0.24599648
NUPL2	-0.070658649	0.18039719	0.246069181
SEMA4A	0.070641485	0.180503332	0.246182425
SNORA38	0.070641226	0.180504935	0.246182425
LOC729668	-0.070603205	0.180740221	0.246476809
APCDD1L	-0.070601447	0.180751107	0.246476809
CFLP1	-0.07059998	0.180760188	0.246476809
HTR3D	0.070598349	0.180770287	0.246476809
TRH	0.070593509	0.180800261	0.2465008
MC2R	0.070588066	0.180833974	0.246529887
VNN1	-0.07058435	0.180856992	0.24654439
SPANXB2	0.070551836	0.181058472	0.246802154
IQCF3	0.070494943	0.181411433	0.247266353
ZNF266	-0.070487162	0.181459744	0.247315275
PDE4C	0.070484571	0.181475833	0.247320277
FBP2	-0.070451178	0.181683295	0.24758607
GRM3	-0.070441626	0.181742671	0.247650037
ANKMY2	-0.07040071	0.181997169	0.247979859
TMEM55B	0.070389382	0.182067669	0.248058947
C11orf34	-0.070386763	0.182083979	0.248060345
KLK5	0.070385216	0.182093607	0.248060345
PIGR	-0.070369957	0.182188619	0.248172801
HPR	0.070334486	0.182409633	0.248456866
CEACAM4	0.070301253	0.182616885	0.248722149
UNK	0.070294348	0.182659963	0.248763809
LOC648691	0.07027779	0.182763306	0.24888753
C10orf79	0.070270426	0.182809281	0.248933116
DOK7	-0.070235184	0.183029422	0.249215844

KLF1	0.070216516	0.183146106	0.249348485
RAP2B	-0.070215593	0.183151877	0.249348485
OR52B6	0.070202456	0.183234027	0.249443273
FAM179A	-0.070162761	0.18348242	0.249760875
HLA-DRB1	0.070161165	0.183492411	0.249760875
MST1P9	0.070154314	0.183535308	0.249802191
FAM71E2	-0.070149118	0.183567851	0.24981732
PURB	-0.070147538	0.183577744	0.24981732
XAGE3	0.07014653	0.183584056	0.24981732
AP3B2	0.070132163	0.183674055	0.249922711
SNORA24	0.070123584	0.183727811	0.249978777
LDHC	0.07011551	0.183778416	0.250030548
C19orf76	0.070104875	0.183845086	0.250104166
NPM2	0.070102866	0.183857683	0.250104218
ALX4	-0.070084488	0.183972939	0.25024391
C13orf15	-0.070073379	0.184042636	0.250321615
RPA1	-0.07006717	0.184081599	0.250357511
HIST3H3	-0.070025065	0.184345979	0.250699958
NOX3	0.070012638	0.184424062	0.250773796
CSAG3	0.070010494	0.184437533	0.250773796
C5orf49	0.070010412	0.184438051	0.250773796
C5orf30	0.069973001	0.184673274	0.251076478
GRIN2D	0.069923638	0.184983989	0.25148175
PDK3	-0.069919562	0.185009664	0.251499486
TMC7	0.069914736	0.185040066	0.251523645
TAPBPL	-0.069908294	0.185080648	0.251561638
BET1	-0.069888338	0.185206421	0.251715408
OSBPL7	0.069878577	0.185267962	0.251781865
ZNF470	-0.069868624	0.18533073	0.251849983
KIAA0196	-0.069862216	0.185371149	0.25188772
HESX1	-0.069854454	0.185420115	0.251937066
LRFN2	0.069815368	0.185666841	0.252255091
TMEM114	-0.069757834	0.186030459	0.252731877
PKNOX1	0.069739351	0.186147382	0.252873473
ICAM5	-0.069727116	0.186224809	0.252961399
TCP10L	0.069705209	0.18636351	0.25313254
NIT1	0.069691594	0.186449746	0.253232401
WSCD1	0.06966213	0.186636471	0.25346872
EMX2	-0.069624026	0.186878152	0.253779639
SPRR2B	-0.069596526	0.187052724	0.253999386
LZTR1	-0.069589966	0.187094382	0.254038632
ATP6V0D1	0.069584098	0.187131658	0.254071923
DPPA3	0.069569507	0.187224359	0.254180456
HEY1	0.069559235	0.187289639	0.254251749
SERPINH1	0.069555363	0.187314251	0.254267828
C19orf38	0.069550828	0.18734308	0.254289629

TNFSF10	-0.069519095	0.187544897	0.254546215
FJX1	-0.069485181	0.187760765	0.254821837
RBP1	0.069475534	0.187822202	0.254887847
C9orf116	0.069464024	0.187895525	0.254969977
SLAIN1	-0.069454166	0.187958336	0.255037832
LMX1B	-0.069448041	0.187997371	0.255073419
OR2T33	-0.069439659	0.188050806	0.255128537
PLGLB2	-0.069435806	0.188075364	0.255144474
DNAH3	0.069387665	0.188382479	0.255541268
MTIF2	0.069385936	0.188393517	0.255541268
CEACAM1	-0.06938183	0.188419733	0.255559421
HOXA6	-0.06937882	0.188438945	0.255568073
C10orf122	-0.069368918	0.188502177	0.255636422
ZNF676	0.069321038	0.188808131	0.256033905
ADC	-0.069315837	0.188841387	0.256061566
IVL	-0.069289118	0.189012307	0.256275877
EMID2	0.069285044	0.189038379	0.256293778
ZNF586	0.069269416	0.189138407	0.256411939
C22orf45	0.069246156	0.189287366	0.256596413
TMEM158	-0.069242525	0.189310625	0.256604415
ACBD4	-0.069241212	0.189319039	0.256604415
OCIAD2	0.069220004	0.189454941	0.256771142
P2RX1	-0.069214095	0.189492824	0.256805008
WFDC8	0.069209195	0.189524237	0.256830103
DDB2	-0.069202192	0.189569143	0.256873476
LRRTM1	-0.069166026	0.189801169	0.257170382
DGKB	-0.069160519	0.189836521	0.257200784
SLC22A20	-0.069139409	0.189972072	0.257366927
GAP43	0.069097059	0.190244219	0.257718088
RAET1L	-0.069079832	0.190355003	0.257850625
SLC5A5	0.069072878	0.19039974	0.257893683
FBXO44	0.069059409	0.190486403	0.25799352
INSL3	0.069055011	0.190514707	0.258014308
IFNA7	-0.069050067	0.19054653	0.258039858
TUBA3E	0.069024673	0.190710047	0.258243733
ANKRD45	-0.06900708	0.190823388	0.258379641
PCSK1	-0.068976085	0.191023194	0.258632599
FNTA	-0.068971994	0.191049573	0.25865073
RGSL1	-0.068951065	0.191184593	0.258815931
PLCZ1	-0.06894742	0.191208109	0.258830172
SULT1A1	0.068933027	0.191301014	0.258938333
WDR24	-0.068928779	0.191328437	0.258957852
NKX6-2	-0.068842319	0.19188725	0.25969654
C1orf51	0.068831418	0.19195779	0.259774354
SKAP1	0.068787125	0.192244612	0.260129121
ZNF343	0.0687869	0.192246066	0.260129121

TMEM22	-0.068781407	0.192281661	0.260130438
KRT25	0.068781321	0.192282216	0.260130438
C22orf15	0.068780703	0.192286225	0.260130438
SLC29A1	0.068756689	0.19244188	0.260323328
TET1	0.068752739	0.192467494	0.260331295
WNT5B	-0.068751749	0.192473913	0.260331295
ZNF19	-0.068746128	0.192510361	0.26036291
CDH15	0.068737238	0.192568028	0.260423216
UAP1	-0.068710449	0.192741867	0.260640611
SLC17A9	0.068649918	0.193135077	0.261150086
LSM1	0.068648416	0.193144846	0.261150086
C12orf41	-0.068645353	0.193164761	0.261151767
UBE2N	0.068644191	0.193172316	0.261151767
ELAC1	0.068638436	0.193209736	0.261184625
DTNA	-0.068633929	0.193239047	0.261206517
TAS2R38	-0.068604793	0.193428596	0.26144499
CXorf59	-0.068580163	0.19358894	0.261643959
ODF3L1	0.068553987	0.193759461	0.261856654
C15orf59	-0.068543456	0.193828094	0.261931634
TPD52L3	-0.068521354	0.193972194	0.262108579
CLGN	0.068503818	0.194086576	0.262245347
SLC4A3	-0.068501388	0.194102432	0.262248978
N6AMT1	-0.068480187	0.194240797	0.262418118
CCL16	0.068453768	0.194413325	0.262633385
CLDN1	-0.068433546	0.194545454	0.262788348
ATP4B	0.068430445	0.194565721	0.262788348
EIF2C1	-0.068430154	0.194567622	0.262788348
C17orf69	0.068427668	0.194583873	0.262792475
DAZ2	-0.068423982	0.194607966	0.262793672
RAB6B	0.068423495	0.194611151	0.262793672
RIC3	-0.068402683	0.194747237	0.262959606
GOLM1	-0.068386429	0.194853571	0.263085347
ARL8A	-0.068375222	0.19492691	0.263166525
OR10H1	-0.068356172	0.195051618	0.263317039
PDZD3	0.068287606	0.195500972	0.263905772
TSPAN13	-0.068282796	0.195532519	0.263930468
LOC100271	0.068279052	0.195557082	0.263945733
MT1DP	0.06827364	0.195592594	0.263975773
C9orf167	-0.068247021	0.195767305	0.264193663
SIAH2	0.068239868	0.195814269	0.264237048
CCDC157	-0.068238083	0.19582599	0.264237048
KHK	0.068220795	0.195939548	0.264372365
SAMD1	-0.068209517	0.196013654	0.264454435
IQSEC1	-0.068206331	0.196034586	0.264464759
MYH6	0.068203144	0.196055535	0.264475105
C3orf65	0.068178651	0.196216563	0.264674401

MOSPD1	0.068168148	0.196285647	0.264749656
CYP26B1	-0.068155786	0.196366982	0.264841423
SR140	-0.068146019	0.196431257	0.264898665
CT47B1	0.068145294	0.196436027	0.264898665
NEU2	-0.068126791	0.196557845	0.265044992
DFFA	0.068075636	0.196894907	0.265481522
ROBO2	-0.068070345	0.196929793	0.26549434
TFR2	-0.06807015	0.196931077	0.26549434
LOC254559	0.068065956	0.196958731	0.265513649
IL1F5	-0.068018894	0.197269271	0.265914278
SNORA31	0.068002733	0.197375992	0.266040127
TMEM50B	-0.06799716	0.197412804	0.266071737
PRKACG	-0.067991773	0.197448394	0.266101697
SLC27A6	0.067988731	0.197468489	0.26611077
PJA1	-0.067980811	0.19752082	0.266163282
ZKSCAN4	0.067933803	0.19783165	0.266564094
RPS6KA1	-0.067919683	0.197925084	0.266671947
TTC29	0.067901241	0.198047166	0.266818381
NYX	0.067896256	0.198080173	0.266844798
GUCA1B	0.067890667	0.198117188	0.26687661
CAP1	-0.067875941	0.198214736	0.266989954
DNAI1	0.067873266	0.198232459	0.266995767
ATP1B4	-0.067862827	0.198301629	0.267070869
IFNW1	-0.067795688	0.198746958	0.267652533
LOC100272	-0.067766452	0.1989411	0.267895868
C17orf46	-0.067712918	0.199296958	0.268356924
WNT1	-0.067701402	0.199373571	0.268441935
ANKRD52	-0.067685645	0.199478427	0.268564959
TMCO4	-0.067630793	0.199843767	0.269017049
HSPA6	0.067630424	0.199846226	0.269017049
WDSUB1	-0.06762873	0.199857517	0.269017049
LOC340017	-0.067627119	0.199868253	0.269017049
HNRNPA0	0.067593618	0.200091643	0.269299525
HLA-DPA1	-0.067566297	0.200273963	0.269526691
PPIEL	-0.067560788	0.200310741	0.26955797
TMEM215	-0.067558559	0.200325623	0.269559783
CLK2	0.067546894	0.200403517	0.269646378
YTHDF2	0.067522171	0.200568674	0.26983833
FAM55A	-0.067521483	0.200573276	0.26983833
BCL11B	-0.067519219	0.200588403	0.269840451
CDNF	-0.067510389	0.200647421	0.269901614
KCTD15	-0.06749704	0.200736662	0.269998909
SLC47A2	-0.067495514	0.200746867	0.269998909
CEACAM7	-0.067438879	0.201125832	0.270490339
LOC643923	0.067432205	0.201170526	0.270532178
SPACA5	0.067415402	0.201283075	0.270665256

EPB41L1	-0.067411748	0.201307561	0.270679905
RDH12	0.067407905	0.201333312	0.270696253
MGC21881	-0.067404189	0.201358213	0.270711456
OTX2	0.067399886	0.201387053	0.270731953
NCRNA0016	0.067394312	0.201424414	0.270763902
SLC17A5	-0.067391507	0.201443215	0.270770898
ZNF677	-0.067388095	0.201466088	0.270783367
PROK2	-0.067382344	0.201504651	0.27081692
CPNE4	0.067377801	0.201535109	0.270839577
ENAM	-0.067365838	0.201615343	0.270929119
S100A14	0.067349946	0.201721964	0.271054106
PSMC6	0.067345918	0.201748996	0.271072139
DKFZp566F	0.067335128	0.201821415	0.271151149
VWA5B1	0.067330225	0.201854333	0.271177081
CCDC85C	-0.067314135	0.201962371	0.271283871
CCDC70	-0.067313254	0.201968292	0.271283871
TLE1	0.067312301	0.20197469	0.271283871
TDGF3	-0.06730763	0.202006067	0.271307717
ANKRD30A	0.067301987	0.202043976	0.271340331
CHPF2	-0.067294336	0.20209538	0.271391065
CENPK	0.067260924	0.202319974	0.27167435
NRCAM	0.067254055	0.202366173	0.271718064
LOC145845	0.067211766	0.202650752	0.272081826
MUC16	-0.067200855	0.202724221	0.272162118
C14orf23	0.067182029	0.202851038	0.272314016
C21orf121	-0.067165671	0.202961273	0.272443634
DEFA1B	-0.067128711	0.2032105	0.272759797
CIRBP	0.067116769	0.203291073	0.272849557
GIPC2	-0.067111058	0.203329616	0.272882897
SLCO1B3	-0.067106762	0.203358614	0.272903423
CLEC4D	-0.067104478	0.203374032	0.272905724
OTUD6B	-0.067098825	0.20341219	0.272938536
OR6C70	0.06709398	0.203444902	0.272964038
MBD2	0.067086911	0.203492638	0.273009693
RPS6KB1	-0.0670811	0.203531881	0.273043948
TMEM169	0.067068498	0.203617011	0.273136794
OR8G5	0.067066794	0.203628521	0.273136794
KIR2DL4	0.067051187	0.203733985	0.273259854
C2orf74	0.067013727	0.203987289	0.273581175
GDE1	-0.067006557	0.204035798	0.273627806
DCST2	0.067000785	0.204074854	0.273661754
TG	-0.066993113	0.204126776	0.273712951
SLC25A5	0.066985866	0.204175831	0.273760295
TMEM190	0.06696408	0.204323347	0.273939643
LOC283314	-0.066947152	0.204438019	0.274074935
DLEU2L	-0.066929092	0.204560416	0.274210869

LOC387647	0.066928128	0.204566954	0.274210869
HPGDS	0.066922637	0.20460418	0.274242311
TMEM95	0.066865087	0.204994625	0.274747154
CDH8	-0.066848753	0.20510554	0.274860291
TM7SF4	-0.066848591	0.205106642	0.274860291
PDCD10	-0.066829977	0.205233091	0.275011237
TBPL2	0.066821447	0.205291055	0.275070401
A2M	-0.066814313	0.205339541	0.275116857
ARID3B	-0.066774683	0.205609052	0.275459419
ACCN3	0.066764518	0.205678222	0.275533553
NCRNA0002	-0.066760674	0.205704384	0.275550064
C19orf77	0.066719759	0.20598299	0.275904712
POGK	-0.066689909	0.206186429	0.276158635
MUS81	0.066687809	0.206200746	0.276159238
LRP3	0.066684337	0.206224418	0.276172368
TXNRD3IT1	-0.066677122	0.206273615	0.276219678
IRF9	-0.06664295	0.206506754	0.27651328
SLC25A4	-0.066635608	0.206556872	0.276561792
NMNAT3	-0.066613911	0.206705028	0.276741555
PCDHA2	0.066602798	0.206780939	0.276824577
CLYBL	-0.066588106	0.206881333	0.276940361
RBM47	-0.066552826	0.207122546	0.277244623
MTMR4	-0.066472526	0.207672327	0.277961852
RPS10P7	0.066457708	0.207773891	0.278079102
BCHE	-0.066438215	0.207907558	0.278220142
MGAT1	0.066437207	0.20791447	0.278220142
ELP2	0.066436229	0.207921184	0.278220142
MAP2K5	0.066428251	0.207975907	0.278274669
PM20D2	-0.06639578	0.208198754	0.278554127
HIST1H2BJ	0.066365659	0.208405632	0.278812183
CHD1L	0.066357329	0.208462873	0.278870029
SLC15A3	-0.066349178	0.208518887	0.278926225
MTNR1A	-0.066320412	0.208716675	0.279172046
KLK8	0.066317545	0.208736392	0.279179667
C11orf36	0.066312069	0.208774061	0.279198385
SELO	0.066311435	0.208778426	0.279198385
ZSWIM2	-0.066307089	0.20880832	0.279219613
TSNAXIP1	-0.066287468	0.208943354	0.279381422
MS4A4A	-0.066282951	0.208974442	0.27940423
FOXL1	-0.066273722	0.209037985	0.279470427
ADCK2	0.066260766	0.209127208	0.279570944
HOXA9	0.066231609	0.209328106	0.27982073
DAP	0.066211824	0.209464504	0.279984267
SPIC	-0.066206719	0.20949971	0.280012532
CD28	-0.066197322	0.209564524	0.280080364
ZNF595	-0.066189927	0.209615546	0.280129754

PRG4	-0.066183085	0.20966275	0.280174037
ACIN1	-0.066163294	0.20979935	0.280337766
SOCS1	0.066159084	0.209828415	0.280357792
NUDT17	0.066151745	0.209879095	0.280406694
STT3A	0.066110385	0.210164851	0.280759052
CCDC109B	-0.066109492	0.210171024	0.280759052
NCF1C	-0.066067109	0.210464143	0.28113176
ORM1	0.066037498	0.210669109	0.281386674
SCNN1G	-0.066033047	0.210699932	0.281408969
POF1B	0.066025596	0.210751531	0.281459009
CASP2	-0.066006437	0.210884265	0.281617391
ABLIM2	-0.065985525	0.211029208	0.281792053
TMEM90A	-0.06598072	0.211062522	0.281817641
FRMD5	-0.065977629	0.211083954	0.281827363
SCN9A	-0.065935606	0.211375493	0.28219769
SPOCK1	-0.065928319	0.211426078	0.282246303
PHOX2B	0.065924683	0.211451321	0.28226108
PAX2	-0.065884655	0.211729358	0.282613282
USP11	0.065879976	0.211761874	0.28263774
OBFC1	0.065872982	0.21181049	0.282683683
DLG3	-0.065853026	0.211949244	0.282849909
RNF148	0.065748343	0.212678169	0.283803654
PTHLH	-0.065676892	0.213176736	0.284449895
CFP	-0.065653845	0.21333773	0.284644065
TRIM55	0.065651968	0.213350839	0.284644065
MLANA	-0.065603429	0.213690209	0.28507774
GUCA1C	-0.065597253	0.213733421	0.285116289
CASR	-0.065588522	0.213794509	0.285173068
RNF31	0.065587077	0.213804623	0.285173068
FLJ46321	0.065564015	0.213966065	0.285369286
NAT8L	0.065560165	0.213993021	0.285386126
OR10A6	0.06552827	0.214216458	0.285664976
LLGL2	0.065515701	0.214304557	0.285763325
BCL2L15	-0.06550565	0.214375019	0.285838144
TMEM61	0.065488237	0.214497144	0.285981832
OR1E1	-0.065479856	0.214555935	0.286041067
C17orf67	0.065471195	0.214616707	0.286102933
CSPP1	-0.065451048	0.214758119	0.286272285
HTR1A	0.065447519	0.214782902	0.286286158
SYK	-0.065440277	0.214833748	0.286334767
SFTA3	-0.065421629	0.214964733	0.286490173
ABCD1	0.065419104	0.214982477	0.286494648
RNASE6	0.065399523	0.21512008	0.286658842
EXOC6	0.065375158	0.215291397	0.286867936
C5orf52	0.065357669	0.215414426	0.287012664
C10orf54	-0.065352205	0.215452876	0.287044689

SOX1	-0.065332223	0.215593519	0.287212852
TUBA4B	0.065327857	0.21562426	0.287228076
FMR1NB	0.06532607	0.215636847	0.287228076
CCR3	0.065324455	0.215648215	0.287228076
ARC	-0.065320052	0.215679222	0.287250164
CCNK	-0.065309422	0.215754094	0.287330666
GRM1	-0.065306693	0.215773317	0.287333116
C7orf63	-0.065305064	0.21578479	0.287333116
TSNAX	0.065285413	0.215923256	0.287498272
LDHAL6A	-0.065281411	0.215951468	0.287516612
REN	-0.06527024	0.21603022	0.287602234
ZNF554	0.065267783	0.216047538	0.287606065
COL9A1	0.065261078	0.216094824	0.287649785
DAZ3	-0.06524661	0.21619687	0.287766386
SNORA2B	-0.065236053	0.216271347	0.28784628
DGKK	0.065211586	0.216444041	0.288052464
KLK6	0.065209449	0.216459129	0.288052464
C12orf50	0.065207959	0.216469655	0.288052464
ALLC	-0.065205124	0.216489668	0.288059847
ARL11	-0.065195605	0.216556895	0.288130048
IFT52	0.065180817	0.216661355	0.288249775
FAM86B1	-0.065168571	0.216747891	0.288345642
SPDYE8P	0.065135156	0.216984135	0.288640641
CPT1C	-0.065128283	0.217032752	0.288686031
OR6C68	-0.065114506	0.217130224	0.288796287
DFNB59	0.065112469	0.217144645	0.288796287
DOC2A	0.065072192	0.217429798	0.289156222
BCKDK	-0.065048016	0.217601093	0.289364702
INTS9	-0.065035532	0.217689578	0.289463039
CYP2E1	0.06502022	0.217798151	0.289588075
TRIM67	0.065011216	0.217862013	0.289650175
SCARNA11	0.065008225	0.217883226	0.289650175
NDUFAF1	-0.065007484	0.217888489	0.289650175
IFNAR2	-0.064998832	0.217949872	0.289712435
APOL4	-0.064996462	0.217966689	0.289714458
OBSCN	-0.064994517	0.217980489	0.289714458
KBTBD13	-0.064991691	0.218000542	0.289721775
TTLL10	-0.064964117	0.218196285	0.289962566
ZNF280B	-0.064941284	0.218358464	0.290158725
C7orf52	0.064925943	0.21846748	0.290284218
SPINK5	0.064888656	0.218732611	0.290607051
HCRTR1	0.064887669	0.218739628	0.290607051
DIRAS2	-0.064866395	0.218891006	0.290788766
KIAA1257	0.064846302	0.219034047	0.290959382
C14orf28	0.064842607	0.219060362	0.290974928
C5orf38	-0.064833452	0.219125564	0.291025863

IL22	0.064833119	0.219127934	0.291025863
TSR1	-0.064819395	0.219225701	0.291132593
NET1	-0.064815979	0.219250039	0.291132593
OR9I1	-0.064815683	0.219252153	0.291132593
PHAX	-0.064810146	0.219291606	0.291165567
GPI	0.064791128	0.219427171	0.29132614
PDCD4	-0.064786246	0.219461981	0.291352933
DCHS2	-0.064780326	0.219504194	0.291375302
OR2AE1	-0.06477978	0.219508092	0.291375302
HOXC8	0.064776807	0.219529292	0.291384021
FHDC1	-0.064765148	0.21961246	0.291474985
BCORL1	-0.064731455	0.219852918	0.291755427
GABRB3	-0.064731435	0.21985306	0.291755427
IDH1	-0.064712557	0.219987869	0.291914873
DCAF16	-0.06467774	0.22023666	0.292225537
TBL1XR1	-0.064675209	0.220254757	0.292230079
PPP3CB	-0.064665624	0.220323284	0.292301526
CXorf64	0.064659311	0.220368433	0.292341949
PCDHB5	-0.064590936	0.220857806	0.292971638
RLN1	0.064587827	0.220880075	0.292981663
LOC100128	0.064580827	0.220930225	0.293028667
FIGF	0.064573845	0.220980252	0.293075501
ZSCAN10	0.064557631	0.221096459	0.293210093
ZFP82	0.064554901	0.221116025	0.293216516
LOC151162	-0.064548438	0.221162368	0.293258443
EFHC1	0.06454159	0.221211468	0.29330402
HSPBAP1	0.064523813	0.221338972	0.29345354
ZG16	0.064511046	0.221430581	0.293546907
PABPC1P2	0.06450989	0.221438874	0.293546907
NGEF	0.064503333	0.221485936	0.29358975
RAP1GAP2	-0.06449534	0.221543305	0.293646251
DOCK3	0.064488419	0.221592994	0.293692565
C9orf44	-0.06446517	0.221759967	0.293894307
GALR1	-0.064441289	0.221931573	0.294102161
ORAI2	-0.064425417	0.222045677	0.294233792
PRSS38	0.06442313	0.22206212	0.294236003
MTERFD2	0.064405432	0.22218941	0.294385077
DERL1	0.064403231	0.22220524	0.294386466
PDYN	0.064383199	0.222349384	0.294557838
HLA-DOA	-0.064366011	0.222473121	0.294702156
VAPB	-0.064359125	0.222522708	0.294729771
CCL25	0.064359005	0.222523567	0.294729771
MEI1	0.064354911	0.222553051	0.294749219
PHOX2A	0.064334065	0.222703225	0.294928496
SLC9A11	-0.064306711	0.222900385	0.295169969
DPY19L2P4	-0.064301205	0.222940085	0.295202912

AGRP	0.064255038	0.223273178	0.295624316
KIAA0090	-0.06424884	0.223317923	0.295663904
KRT37	0.064234906	0.223418534	0.295777446
FAM26D	-0.064228971	0.223461402	0.295814533
HHIPL1	-0.064222978	0.223504692	0.295852173
DFNA5	-0.064217861	0.223541662	0.295881443
HS3ST6	0.064204018	0.223641688	0.29599368
CACNB1	-0.064202013	0.223656184	0.29599368
LOC100130	-0.064190284	0.223740966	0.296086207
CFC1B	0.064178995	0.22382259	0.296174544
IL12RB1	-0.064173364	0.223863309	0.296208743
ARL9	0.064167826	0.223903366	0.296242063
SNAPC4	0.064156682	0.223983981	0.296329037
SLC7A3	0.064153771	0.224005045	0.296337218
LOC388946	-0.06409015	0.22446571	0.296926909
SDHAP2	-0.064083148	0.22451645	0.29695587
C18orf10	0.064083014	0.224517426	0.29695587
UNC13A	-0.064072537	0.224593363	0.29703658
CHP2	0.064047755	0.224773062	0.297254501
RAD51L3	-0.064010249	0.225045215	0.297594651
TTLL3	-0.064006524	0.225072258	0.297610652
ACTL7B	0.063999396	0.225124009	0.297659317
CCDC92	-0.063991157	0.225183841	0.297718662
SLC22A9	0.063981127	0.225256696	0.297795214
OR1N1	-0.06396043	0.225407078	0.297974242
C1orf94	-0.063950245	0.225481113	0.298052328
ANO10	0.06392173	0.225688469	0.298306621
ANKRD37	0.06391523	0.225735752	0.298347623
LOC284578	-0.063913347	0.225749452	0.298347623
ZNF157	-0.06390037	0.225843879	0.298452611
CXCL2	-0.063883517	0.225966554	0.298594912
FCGR1C	0.063840719	0.226278303	0.298987022
CSF2	-0.063827671	0.226373405	0.299092838
OXNAD1	-0.063812826	0.226481639	0.29921599
ZNF362	-0.063784512	0.226688184	0.299469001
HSP90AB2P	-0.063779407	0.226725438	0.299498349
ZFAND6	-0.063775985	0.226750414	0.299511474
TAP2	0.063752968	0.226918443	0.299713542
GABRG3	0.063736813	0.227036433	0.299849497
SLC37A2	-0.063722266	0.227142716	0.299969973
MOG	-0.063710166	0.227231151	0.300066864
FCGR3A	-0.063694526	0.227345492	0.30019795
TSPAN3	-0.063682555	0.227433034	0.300293634
PON1	-0.063662207	0.227581895	0.300470263
INA	-0.063655879	0.227628204	0.300510901
PARVB	0.063653878	0.227642855	0.300510901

C6orf145	0.063648183	0.227684538	0.300546004
MINPP1	-0.063630681	0.22781267	0.300695209
CHST10	0.063628449	0.227829015	0.300696855
NXNL1	-0.063620017	0.227890771	0.300758429
SLC44A5	0.063612403	0.227946547	0.300812105
ZNF853	-0.06360242	0.228019685	0.300888684
C14orf178	-0.063585003	0.228147333	0.301037178
CLEC10A	-0.063567002	0.228279316	0.301191372
CYP17A1	0.063545316	0.228438384	0.30138128
HBBP1	0.063503282	0.22874694	0.301768369
LOC374491	-0.063498999	0.228778394	0.301789873
PPP2R2B	0.063431825	0.229272149	0.302421172
IL23R	-0.063423725	0.229331734	0.302479733
SDCCAG8	-0.063420988	0.229351871	0.302486259
NTSR2	-0.063418879	0.229367391	0.302486696
DNAJC5B	0.063411831	0.229419258	0.302535063
SREBF2	-0.063389798	0.229581446	0.302728894
SLC32A1	0.063360368	0.229798207	0.302994656
OR2C3	0.063344482	0.229915278	0.303128948
AMAC1L2	-0.063329671	0.230024464	0.303252827
HBA1	-0.063287569	0.230335031	0.303642162
SUMF2	0.063268202	0.230477991	0.30381051
PRAMEF17	0.063238818	0.23069502	0.304076466
BCL2L10	-0.06322074	0.230828613	0.304232418
HNRNPUL1	-0.063151634	0.231339794	0.304885977
C15orf53	0.063148827	0.231360575	0.304893187
KEL	-0.063144898	0.231389665	0.304911347
PARP10	0.063118538	0.231584891	0.305148413
AKAP14	0.063107741	0.231664891	0.305233629
OSBPL3	-0.063094178	0.231765411	0.305345869
TMEM55A	-0.063053634	0.23206608	0.305721768
MCART2	-0.063039187	0.232173283	0.305842766
FLJ22536	-0.063031403	0.23223106	0.305898641
PMCHL2	-0.06302064	0.232310962	0.305983651
CRX	0.063015706	0.232347601	0.306011671
RHBDF2	-0.062969919	0.232687776	0.30643943
STOX1	0.062967609	0.232704948	0.30644178
C9orf4	0.062965312	0.232722022	0.306444001
AADAC	-0.062953874	0.232807064	0.306535714
C22orf25	0.062934791	0.232948995	0.306702316
IGF2	-0.06292087	0.233052576	0.306818407
KLHL17	0.062913225	0.233109471	0.306873024
SOX21	-0.062907897	0.233149128	0.306904943
OR2T34	-0.06285898	0.233513456	0.307364209
C9orf9	0.062828225	0.23374272	0.307645645
ADAM18	0.062802072	0.233937806	0.307882064

ELAC2	0.062793647	0.234000673	0.307944453
BTBD9	0.062732497	0.234457345	0.308525045
OR9A4	0.062723814	0.234522245	0.308590057
OR51B2	-0.062691029	0.234767389	0.308878876
TSPY4	0.062690311	0.234772762	0.308878876
CD24	0.062658892	0.235007865	0.309167765
C19orf51	-0.062654415	0.235041383	0.309191436
METTL3	-0.062636586	0.235174886	0.309346622
OR2A5	-0.062588969	0.235531695	0.309795502
EPHX4	0.062586853	0.235547558	0.309795906
TMEM187	0.062583777	0.235570619	0.309805776
ARF4	-0.062576793	0.235622993	0.309854193
WDR41	-0.062550752	0.235818339	0.310061987
LCN10	0.062550012	0.235823897	0.310061987
ARF1	0.062547888	0.23583983	0.310061987
GNAT1	0.062546906	0.235847201	0.310061987
FTMT	0.062545354	0.235858852	0.310061987
ZIC4	0.062542546	0.235879923	0.310069219
KRT15	0.062527417	0.235993487	0.310198026
FBXW5	0.062493294	0.236249768	0.310514396
CNTD2	-0.062482401	0.236331624	0.310601484
CYTH4	0.062479993	0.236349719	0.310604768
KIAA1751	0.062425043	0.236762958	0.311127306
CC2D2A	-0.062416118	0.236830126	0.311195036
CDX1	-0.062386161	0.237055669	0.311470849
PRH2	-0.062379676	0.237104516	0.311514477
MANEAL	0.062359375	0.237257472	0.31169397
GPRC5C	0.062357389	0.237272437	0.31169397
TRIM71	0.062351722	0.237315152	0.31172952
SNORD115-	-0.062333531	0.237452288	0.311889086
LOC389705	0.062311728	0.237616728	0.312084492
FCER2	0.062301121	0.237696759	0.312169015
PLAC8	-0.062288618	0.237791118	0.312269586
FOXS1	0.062284788	0.237820023	0.312269586
ZNF732	0.062284741	0.237820377	0.312269586
COPS4	-0.062275154	0.23789276	0.312344035
LARP4B	-0.062269707	0.237933888	0.312377439
CRYBA1	0.06226519	0.237967993	0.31240162
KIR2DL1	-0.062239843	0.238159463	0.312632371
GPR39	-0.0622103	0.238382769	0.31290488
SPACA3	0.062194577	0.23850167	0.313040319
TEX11	0.062180615	0.238607298	0.31315832
HIC2	0.062171245	0.238678197	0.313230728
CGB2	0.062153498	0.238812524	0.313386361
OTUB2	0.062120378	0.23906336	0.313694855
SIX1	0.0621021	0.239201871	0.313855927

CLDN4	0.062088723	0.239303275	0.313968293
APOBEC3F	-0.062084462	0.239335575	0.313989986
CCDC116	-0.062072548	0.239425924	0.314087825
TSC1	-0.062057243	0.239542023	0.314200697
ALDH7A1	-0.062056279	0.239549337	0.314200697
ALDH3B1	0.062054967	0.239559296	0.314200697
IGSF6	-0.062046873	0.239620708	0.314260547
LOC400752	0.062024594	0.239789822	0.314461629
VAMP2	-0.062006359	0.239928298	0.314608178
ANTXRL	-0.062005718	0.239933167	0.314608178
NUP43	0.061981455	0.240117504	0.314829158
SLC6A11	-0.061970303	0.240202267	0.314919563
SPATA2	0.061965614	0.240237914	0.314945563
ZNF135	-0.061958554	0.240291592	0.314995198
PTBP2	-0.06195325	0.240331924	0.315027333
SSX7	0.061870925	0.24095853	0.315827901
KIAA0664	-0.061860653	0.241036799	0.315909698
NFATC4	0.061845292	0.241153866	0.316042329
SLITRK5	-0.061841881	0.241179872	0.316055613
FBXO18	-0.061831362	0.241260068	0.316139904
RNF175	-0.061826478	0.241297311	0.316167902
NCRNA0011	-0.061822518	0.241327509	0.316186668
OSR2	0.061812102	0.241406961	0.316269958
TM9SF4	-0.061801341	0.241489059	0.316356704
LGALS9	0.061794243	0.241543224	0.316406847
CAPN8	0.061785244	0.241611904	0.316475998
KIAA2022	-0.061781145	0.24164319	0.31649616
FOXN1	-0.061760082	0.241804012	0.31667851
GLT25D1	0.061758746	0.241814215	0.31667851
ZNF585A	0.061755693	0.241837532	0.31668822
NCAPD2	0.061724628	0.242074887	0.316978195
ACO2	-0.061718152	0.242124382	0.317022161
TMPRSS11D	-0.061711943	0.242171851	0.317063468
FFAR2	0.061671212	0.242483387	0.317450476
TSKU	-0.061649674	0.242648241	0.317645417
KRTAP5-9	0.061646876	0.242669665	0.317652582
BEND5	0.061634268	0.242766206	0.317758067
FIGNL1	-0.061609549	0.242955564	0.317972867
SPG7	-0.061608677	0.242962246	0.317972867
FAM46B	0.061604714	0.242992617	0.317984785
PRAMEF4	-0.061603321	0.243003286	0.317984785
KLHL35	0.06156515	0.243295941	0.318346824
FBXL22	-0.061554472	0.243377854	0.318423185
MC5R	-0.061553374	0.243386278	0.318423185
WWOX	-0.061523986	0.243611818	0.318697323
OR52R1	-0.061518764	0.243651909	0.318728834

CERCAM	-0.06151473	0.243682882	0.318748414
TLR9	0.06150686	0.243743315	0.318806523
EPDR1	-0.061444967	0.244218976	0.31940769
C1orf115	0.061433556	0.244306744	0.319501497
TBC1D10C	0.061407904	0.244504124	0.319738633
TMEM202	0.061399531	0.244568568	0.319801906
NR0B2	0.061335188	0.245064238	0.320424727
HK2	-0.061333526	0.24507705	0.320424727
PRSS3	0.061330909	0.245097227	0.320430071
WDR69	0.061320583	0.245176846	0.320513121
PNLDC1	0.06131703	0.245204247	0.320527901
ACTA2	-0.061312775	0.245237066	0.320549762
SNORD115-	-0.061297989	0.245351122	0.320677798
CNNM2	0.061282967	0.245467045	0.320808256
SAA2	-0.061261182	0.24563522	0.321006982
SPINK14	-0.061255782	0.245676916	0.321040406
LMO4	-0.061194799	0.246148167	0.321632653
C9orf144	-0.061192954	0.246162435	0.321632653
C2orf15	-0.061173343	0.246314121	0.32180973
KLHL21	-0.061168014	0.246355353	0.321842486
GIMAP5	-0.061161659	0.246404529	0.321875404
FAM71D	0.06116058	0.246412875	0.321875404
GH1	0.061148853	0.246503644	0.321961163
FIP1L1	-0.06114792	0.246510862	0.321961163
TMPRSS5	0.061130754	0.246643766	0.322082428
C6orf115	-0.061129748	0.246651552	0.322082428
NHLRC4	0.06112834	0.246662459	0.322082428
SOLH	-0.061127573	0.246668401	0.322082428
FOXB1	-0.061103401	0.246855643	0.322305784
C17orf88	-0.061075743	0.247070006	0.322564518
PUS7	-0.06102952	0.247428558	0.323011454
FAM19A5	-0.061009485	0.247584076	0.323193292
C7orf28B	-0.061002908	0.247635145	0.32323877
OCM2	0.060997873	0.247674248	0.323268624
KIF19	-0.060995026	0.247696363	0.323276301
C3orf14	0.060972815	0.247868908	0.323480295
RYR3	0.060940566	0.248119588	0.323786226
ARMS2	0.060933077	0.248177824	0.323813636
ZNF208	-0.060931877	0.248187157	0.323813636
CXCR5	-0.060931592	0.248189372	0.323813636
C5orf58	0.060887243	0.248534463	0.324242635
CDKN1C	0.06088363	0.248562597	0.324258097
CXCR3	0.060860483	0.248742848	0.324471987
C6orf35	0.060854274	0.248791223	0.324513833
ACTL7A	-0.060839981	0.248902587	0.324622782
LGMN	0.06083937	0.24890735	0.324622782

LOC100270	0.060832554	0.248960473	0.324670802
TTTY14	0.06082831	0.248993553	0.32469268
DGKH	0.060818721	0.249068309	0.324768897
PTH2R	0.060801065	0.249205988	0.324927147
C1orf174	0.060797667	0.249232492	0.324933602
FAM45A	-0.060796247	0.249243571	0.324933602
NKX6-3	0.060785099	0.249330541	0.325025707
MYH13	-0.060756643	0.249552637	0.325293936
CPB1	-0.060743435	0.249655768	0.325397632
TMEM98	0.06074227	0.249664867	0.325397632
ACYP2	-0.060719231	0.249844835	0.325610882
EIF1AY	0.060699625	0.249998061	0.325789254
ACCN4	0.060695959	0.250026718	0.32580528
SEMA3F	0.060658973	0.250315976	0.326148122
IPO13	0.060658129	0.250322573	0.326148122
C10orf53	0.060645754	0.250419408	0.326252944
GLE1	0.060641491	0.250452772	0.326266003
ACPP	0.060640287	0.250462197	0.326266003
B3GALT4	0.060628465	0.250554738	0.326365204
ELAVL1	-0.060587551	0.250875196	0.326761251
RAX2	-0.060555381	0.251127368	0.32706831
TMEM82	0.060482128	0.251702232	0.327795577
HMP19	0.060477787	0.251736326	0.3277975
C1QTNF3	0.060477749	0.251736629	0.3277975
GTF2IRD1	0.060470238	0.251795626	0.327839885
TRIM6-TRI	-0.060467664	0.25181585	0.327839885
CTF1	-0.060467318	0.251818565	0.327839885
C2CD2L	-0.060462143	0.251859228	0.32787139
44079	0.060457157	0.251898406	0.327900958
FA2H	-0.060450247	0.251952706	0.327931294
OR1B1	-0.060450001	0.251954644	0.327931294
FERMT1	0.060446457	0.251982492	0.327946106
SYN2	-0.060439714	0.2520355	0.327993661
SUPT7L	0.060435361	0.252069722	0.328016761
C19orf66	0.060426752	0.252137409	0.328083404
ENAH	-0.060406528	0.25229647	0.328268928
KCTD13	-0.060367473	0.252603838	0.328647379
C17orf77	-0.060327373	0.252919696	0.329020428
SFTPA2	-0.060326055	0.252930086	0.329020428
HCG22	-0.06032478	0.252940133	0.329020428
LOC400043	0.06031041	0.253053395	0.329146259
EED	0.060289226	0.253220429	0.329342009
OR5H6	0.060278051	0.253308575	0.329435138
LRRC6	-0.060249138	0.253536725	0.329710323
NUDT9P1	-0.060237485	0.25362872	0.32980842
AQP12A	0.060229539	0.253691465	0.329868472

HTR1F	-0.060209285	0.253851449	0.330040511
SLC22A16	0.060206751	0.253871465	0.330040511
KIR3DP1	-0.06020422	0.253891463	0.330040511
PRKDC	-0.060202712	0.25390338	0.330040511
CCNB3	-0.0602023	0.253906637	0.330040511
ICOS	0.060180379	0.254079885	0.330244153
GJB6	0.060157839	0.25425811	0.330454236
C6orf192	-0.060145786	0.254353454	0.33055658
NCRNA0009	-0.060138779	0.254408891	0.330607051
ZNF212	0.060127139	0.254501006	0.330705177
NCRNA0009	0.06009944	0.254720284	0.330968517
VPS37B	-0.060094584	0.254758741	0.33099689
DARS2	-0.060073035	0.254929452	0.331193771
PPP5C	-0.060071257	0.254943536	0.331193771
DDX42	-0.060064206	0.254999414	0.331244755
LRRC26	0.060061342	0.255022115	0.331244913
LBX1	0.060059993	0.255032802	0.331244913
TRIM47	0.060042104	0.255174614	0.331407489
HIPK4	0.060032202	0.255253136	0.331487852
FGFRL1	-0.06002084	0.255343251	0.331583258
OSBPL6	-0.059972696	0.255725354	0.332057797
DNAJA1	0.059960419	0.255822856	0.332162745
TXLNA	-0.059946609	0.255932557	0.332283517
GNAL	-0.059918492	0.256156021	0.332538826
TAS1R2	-0.059917665	0.256162598	0.332538826
PAX6	0.059909702	0.256225909	0.332599334
CCDC78	-0.059894927	0.256343405	0.332730164
AIRE	0.059880665	0.256456863	0.332855737
PPP2R2C	0.059859843	0.256622572	0.333049106
CGB	-0.059856731	0.256647343	0.333049464
PECAM1	0.059855606	0.256656295	0.333049464
AQP2	-0.059847879	0.256717817	0.333107592
SYNGR3	0.059831831	0.256845613	0.333251702
ZNF329	0.059821577	0.25692729	0.33333596
CERKL	0.059816006	0.256971674	0.333371825
NCRNA0023	0.059780125	0.257257663	0.333721102
HLA-F	0.059775219	0.257296785	0.333750112
SNORA71B	-0.059751133	0.257488905	0.333977566
C9orf117	0.059741612	0.257564875	0.334054346
EEF1DP3	0.059738035	0.257593428	0.334069622
POU2AF1	0.059734502	0.257621618	0.334084426
TSNAX-DIS	-0.059723249	0.257711446	0.334168974
CHADL	0.059722131	0.257720375	0.334168974
HOMEZ	0.059717435	0.257757861	0.33419582
ZNF101	0.059702829	0.2578745	0.334325283
COQ7	0.059691362	0.257966098	0.334422266

VAMP3	-0.059666037	0.258168469	0.33466283
CA8	-0.059653111	0.2582718	0.334764104
PF4	-0.059652058	0.258280214	0.334764104
TMED8	-0.059637831	0.258393983	0.334889768
WTIP	-0.059622537	0.258516326	0.335020749
SUSD3	0.059620991	0.258528691	0.335020749
KNCN	-0.059576052	0.25888841	0.335465072
C2orf3	0.05954752	0.259116975	0.335739399
ATP5A1	-0.059540195	0.259175685	0.335793622
TTYH1	0.059535144	0.259216163	0.335824218
C7orf53	-0.059532928	0.259233931	0.335825391
LYRM2	0.059512498	0.259397723	0.336015718
HABP2	-0.059507898	0.259434615	0.33604165
TCEAL3	-0.059491299	0.259567765	0.336192251
MID1	0.059422127	0.260123138	0.336889659
PELI3	0.059382228	0.260443849	0.337261493
MATK	0.059382195	0.260444113	0.337261493
SYT1	0.05937602	0.260493778	0.337303874
TRIM53	0.059365102	0.260581595	0.337395649
C1orf144	0.059304227	0.261071626	0.338008155
CYP2D6	0.059297462	0.261126119	0.338056728
CHN2	-0.059276192	0.261297509	0.338256624
C3P1	-0.059273603	0.261318374	0.338261646
LOC728392	0.059268845	0.261356726	0.338289302
ZDHHC6	-0.059241326	0.261578626	0.338554516
CXorf51	0.059237607	0.261608621	0.338571333
C10orf81	0.059231659	0.261656606	0.33861143
TMEM195	-0.05922939	0.261674908	0.338613112
SEH1L	0.059221041	0.26174227	0.338678273
ALOXE3	-0.059205879	0.261864634	0.338800759
NCAPG2	-0.059205096	0.261870955	0.338800759
FCRL3	-0.059202742	0.261889955	0.33880333
OPN3	0.05919706	0.261935828	0.338840663
DHX40P1	0.059179506	0.262077573	0.339002005
CKAP2L	0.059173396	0.262126922	0.339043817
KLHL14	-0.0591636	0.262206058	0.339115028
RASGRF1	-0.059162365	0.262216034	0.339115028
SCRT2	0.059152032	0.262299525	0.339200976
GTF2H2	-0.059105218	0.262678018	0.33966838
ZDHHC9	-0.059099834	0.262721572	0.339702643
FBLIM1	-0.059080008	0.262881997	0.339888006
PRAMEF1	-0.059060004	0.263043926	0.340051353
PON2	0.059058779	0.263053843	0.340051353
KRT23	0.059058073	0.26305956	0.340051353
CA10	-0.059050126	0.263123913	0.340112464
DUOX1	0.059025084	0.263326768	0.340352582

GSTTP1	0.059021618	0.263354856	0.340366796
CALML5	-0.059009612	0.263452154	0.340470451
ANO9	0.058995198	0.263569005	0.34059936
UTS2R	-0.058989615	0.263614273	0.340635754
KY	-0.058966582	0.263801087	0.340855034
DMRTC2	0.058952275	0.263917175	0.340982907
SIGLEC11	0.058920174	0.264177773	0.34129746
BET1L	0.058910275	0.264258168	0.341379179
LOC729609	0.058878072	0.264519818	0.341695024
WNK2	0.058863152	0.264641105	0.341829524
SNORD94	0.058857231	0.264689251	0.341869539
TSPAN32	-0.058839374	0.264834486	0.342034941
GRM7	-0.058827738	0.264929144	0.342135005
ADRB3	-0.058821431	0.264980466	0.342179094
SLA2	-0.058796687	0.265181874	0.342416976
OR52K1	-0.058786788	0.265262484	0.342479339
GSTA2	0.058786532	0.265264564	0.342479339
MBOAT4	-0.05877191	0.265383666	0.342610174
SIX3	0.058769867	0.265400309	0.342610174
EGR4	-0.058764384	0.265444975	0.342645624
SPAG17	-0.058733304	0.265698282	0.342950372
DNAJC25-G	-0.058717751	0.265825102	0.343079057
TUSC5	-0.058716852	0.265832434	0.343079057
OR4D9	-0.058702759	0.265947389	0.343205175
NAP1L2	-0.058692272	0.266032946	0.34329334
SPG21	-0.058663436	0.266268311	0.343574797
OR5AN1	0.05863872	0.26647017	0.343812986
ATP1A1	-0.058635367	0.266497562	0.343826053
LEPROTL1	-0.058630742	0.266535341	0.343852517
KRTAP19-4	0.058626205	0.266572412	0.343878066
GC	-0.058601634	0.26677324	0.344114843
PPEF1	0.058584084	0.266916739	0.344277646
LENEP	-0.058565536	0.267068461	0.34443228
C20orf118	0.0585652	0.267071216	0.34443228
NACC1	-0.058532475	0.267339044	0.344755363
OR4D6	-0.058511041	0.267514569	0.344959378
LRRIQ1	0.058475672	0.267804383	0.345310734
DLGAP1	-0.058457613	0.267952438	0.34547927
TMEM132A	0.058453699	0.26798454	0.345498291
FOXD4L1	-0.058430987	0.268170832	0.345716088
POTEC	0.058405637	0.268378881	0.345961902
HCG2P7	-0.058363576	0.26872431	0.346384766
SNORA37	0.058340459	0.268914287	0.346607211
ANGPTL4	-0.058326293	0.269030755	0.346734887
PDZK1	0.05832042	0.26907905	0.346770848
LYPD3	0.058318665	0.269093481	0.346770848

ODF3	0.058298802	0.269256864	0.346958941
C12orf75	0.058292283	0.269310505	0.34700561
SIT1	0.05828859	0.269340891	0.347022309
SCGB1C1	-0.058278558	0.269423457	0.347106232
ANPEP	-0.05826425	0.269541239	0.347235511
REEP5	0.058254274	0.269623381	0.347299353
CYB5A	-0.058253996	0.269625674	0.347299353
RPTOR	-0.058251858	0.26964328	0.347299569
CTRC	0.058246576	0.269686783	0.347333136
NUDT19	0.058224037	0.269872467	0.347549805
ITIH2	-0.05821853	0.269917847	0.347585769
NDUFS2	0.058192323	0.270133883	0.347841476
SPINK8	-0.058180998	0.270227276	0.347939239
C1orf84	-0.058139908	0.270566315	0.348353256
SEMG2	-0.058123012	0.270705803	0.348484337
RGS8	-0.0581224	0.270710864	0.348484337
RRAD	-0.058121218	0.270720622	0.348484337
ANXA8L1	-0.058098126	0.270911359	0.348707324
LOC441177	-0.058086085	0.271010855	0.348812846
ZNF300	0.058078993	0.271069462	0.348865733
BHMT2	-0.058075037	0.271102163	0.348885272
C12orf4	0.058033064	0.271449256	0.349309377
DMRTA1	-0.058026683	0.271502051	0.349354742
FOXD4	-0.057991421	0.271793915	0.349707703
C12orf26	0.057986489	0.271834756	0.349737656
LRRIQ3	-0.057981091	0.271879462	0.349772577
C11orf88	-0.057950858	0.272129926	0.350064352
ACTL9	-0.057949472	0.272141416	0.350064352
TEKT2	0.057938999	0.272228216	0.350153388
LOC284379	0.057931639	0.272289226	0.350209244
KCNMB4	0.057921525	0.272373084	0.350294477
PAR-SN	-0.057908857	0.272478148	0.350392309
NCR3	0.05790811	0.272484343	0.350392309
HDAC7	-0.057850625	0.272961436	0.350972639
SLC2A1	0.057849487	0.272970887	0.350972639
POM121C	-0.057817252	0.273238679	0.351294273
DPPA5	0.057789361	0.273470524	0.351569653
ESD	0.05778581	0.273500046	0.35158491
PLLP	0.057770866	0.27362434	0.351721986
CLCA2	-0.057768042	0.273647829	0.351729477
C16orf78	-0.057745391	0.273836294	0.351949003
IL1RL2	-0.057724917	0.274006728	0.352145327
OR9Q1	-0.057722172	0.274029586	0.352151979
PRPF18	0.057718887	0.274056941	0.352164407
PON3	-0.057690452	0.274293792	0.35244602
DNA2	-0.05768781	0.2743158	0.352451559

CA11	0.05768568	0.274333546	0.352451621
ITGAX	-0.057620319	0.274878557	0.353129046
C2CD4A	-0.057607275	0.274987411	0.353246101
NUP188	-0.057575001	0.27525687	0.35355163
EPS15L1	-0.057574535	0.27526076	0.35355163
USP37	-0.057516236	0.275747962	0.354148831
RAB17	0.057514644	0.275761281	0.354148831
DEXI	0.057511765	0.275785354	0.354156315
ABP1	-0.057509694	0.275802676	0.354156315
TAF7	0.057504687	0.275844546	0.354187243
HERC2P4	0.057500192	0.275882148	0.354212686
SEZ6	-0.05749694	0.275909354	0.35422478
C2orf80	0.057477594	0.276071222	0.354409745
RARRES1	-0.057457503	0.276239384	0.354602768
ERCC8	-0.057397182	0.276744714	0.355228552
ENPP2	-0.057343942	0.277191245	0.355778786
KIAA0319L	-0.057324699	0.277352751	0.35596314
PPIL3	0.057311317	0.277465116	0.356084404
IFRD1	0.057305049	0.277517753	0.356129006
NCF1B	-0.057295723	0.277596078	0.356206565
NECAB1	0.057276187	0.277760217	0.356394223
MSH4	-0.05727318	0.277785485	0.356403681
TACR3	0.057258946	0.277905126	0.356534213
SLC25A41	0.057251753	0.277965596	0.35658882
CRCT1	-0.057235778	0.278099931	0.356738172
RASSF9	-0.05722355	0.278202781	0.35684712
C1QL1	-0.057217038	0.278257563	0.356894401
FARP1	-0.057208756	0.278327251	0.356960793
GPAT2	0.057198046	0.278417389	0.357053403
DMPK	-0.057093665	0.279296886	0.358158241
TMEM92	0.05708645	0.279357749	0.358213224
ZNF540	-0.05707636	0.279442875	0.358278256
COLEC11	-0.057076174	0.279444447	0.358278256
GATS	-0.05707354	0.279466666	0.358283678
C3AR1	-0.057062734	0.279557865	0.358377527
GRIN2C	-0.057046723	0.279693024	0.358527668
MGAT4B	-0.057044595	0.279710991	0.358527668
ADAMTS20	0.057041136	0.279740197	0.358536804
LCNL1	-0.057039486	0.279754125	0.358536804
TAS2R9	-0.05703477	0.279793952	0.358564772
TMEM121	-0.057028733	0.279844937	0.35859575
CEACAM5	-0.057027644	0.279854138	0.35859575
SELENBP1	-0.057004961	0.280045769	0.358818213
C17orf80	0.057001506	0.280074958	0.358832527
NAALAD2	-0.056973167	0.280314516	0.359116346
NUP62CL	0.056968783	0.280351585	0.359140732

GRK7	-0.056953357	0.280482056	0.359284759
ZMYM3	0.056945607	0.280547616	0.359345624
DLEU2	0.056931712	0.280665192	0.359461095
PLSCR2	0.056930687	0.280673865	0.359461095
HPN	0.056927141	0.280703878	0.359476415
NDUFA5	-0.056915998	0.280798193	0.359574075
DOK1	0.056908956	0.280857811	0.359627294
NRXN1	-0.056902731	0.280910522	0.359671663
GFI1B	-0.056871928	0.281171435	0.359982587
CALHM3	0.056838382	0.281455776	0.360323462
BDH1	-0.056813591	0.281666029	0.360535532
CST11	0.056812905	0.281671846	0.360535532
SLC36A2	0.056812446	0.281675739	0.360535532
KIAA1143	-0.056808352	0.281710468	0.36055681
SFXN2	0.056778729	0.281961875	0.360855391
USP9Y	-0.056765898	0.282070809	0.360971608
TFAP2D	0.056750944	0.282197806	0.361110924
MGC4473	0.056747181	0.282229775	0.361128629
OOEP	0.056731315	0.282364568	0.361277891
DSC1	-0.056709546	0.282549589	0.361491396
LOC121952	-0.056688727	0.282726616	0.361694646
HESRG	0.056676858	0.28282757	0.36179102
NSUN4	0.056675598	0.282838282	0.36179102
PRB4	-0.056666931	0.282912025	0.361862105
GPR87	-0.05665716	0.282995162	0.361945197
HLA-DQA2	-0.056645041	0.283098318	0.362053879
OR6T1	-0.056640106	0.283140326	0.362084351
PDK2	-0.056617541	0.283332466	0.362306797
ELOVL2	0.056610713	0.283390621	0.362357895
SOD2	-0.056593131	0.283540414	0.362526152
DAD1L	0.05658188	0.283636297	0.362625465
CLCF1	-0.05657907	0.283660251	0.362632809
PALM3	0.056563456	0.283793358	0.362760121
DDX52	0.056563115	0.283796269	0.362760121
MAGEB16	0.05652045	0.284160207	0.363202011
OR52H1	-0.0565051	0.28429122	0.363346146
KDM1B	-0.056485331	0.284460013	0.363538545
WDR81	-0.056476844	0.284532491	0.363607839
IL2RG	0.056468604	0.284602874	0.363674446
FBXO5	-0.056464318	0.284639494	0.363697904
SERPINA10	-0.056448958	0.284770745	0.363842265
TFB1M	0.056434116	0.284897606	0.363981
RIMKLA	-0.056392503	0.285253493	0.364412298
NCRNA0020	0.056365247	0.285486754	0.364686896
PRAMEF5	-0.056341744	0.285688003	0.364920568
OR13C4	0.056337842	0.285721417	0.364939842

KDELC1	-0.056308678	0.285971292	0.365235572
ST18	0.056272923	0.286277834	0.365603633
LOC554202	-0.056270459	0.286298972	0.365607184
C2orf14	0.05626097	0.286380364	0.365677034
ARL5C	-0.056259801	0.286390394	0.365677034
CXCR4	-0.056231953	0.286629359	0.365958693
FAM90A7	-0.056213829	0.286784963	0.366126795
CELA3A	0.056212335	0.286797791	0.366126795
IL11	-0.056208951	0.286826847	0.366140418
LOC728606	-0.056194183	0.286953692	0.366278861
ZFAT	-0.05618597	0.287024245	0.366342596
PRUNE	0.056184088	0.287040415	0.366342596
CASP9	-0.056135186	0.287460781	0.366855589
KRT83	-0.056115267	0.287632132	0.367050744
DCAF8L2	0.056106622	0.287706515	0.36712214
THTPA	0.056080229	0.287933692	0.367388486
C9orf30	0.05607042	0.288018158	0.367472715
GYS1	0.05602269	0.288429383	0.36797381
TRIM45	0.056000656	0.288619356	0.368192587
ZNF568	-0.055998248	0.288640122	0.368195492
TDRG1	-0.055983038	0.288771316	0.368339253
POLR2J2	0.055973025	0.288857702	0.368425844
CNTFR	-0.055968451	0.288897167	0.368429461
ZNF2	-0.055968408	0.288897538	0.368429461
PDXP	0.055930022	0.289228905	0.368828433
PPCS	0.055919458	0.289320147	0.368909912
CMBL	-0.055916268	0.289347699	0.368909912
HIST1H3E	-0.05591619	0.289348372	0.368909912
MFAP2	0.05586852	0.289760353	0.369411524
MGC29506	0.055856984	0.289860115	0.369515055
CP	-0.055842432	0.289985985	0.369651852
PAX4	0.055811737	0.290251609	0.369966769
SPOCK3	-0.055808283	0.29028151	0.369981202
SNORA16B	-0.055799561	0.290357019	0.370053758
LOC286094	-0.055737308	0.290896366	0.370717421
GLB1L2	-0.055719514	0.291050652	0.370890308
UBAP2	-0.05571222	0.291113908	0.37094718
OR2B11	-0.055685087	0.291349317	0.371223394
PTGR1	0.055678782	0.291404037	0.371269363
ZNF155	0.055673063	0.291453675	0.37130885
C2orf71	-0.05565567	0.291604677	0.37147746
OR6C3	0.055653079	0.291627172	0.371482354
WFIKKN2	0.055637817	0.291759724	0.371627433
C9orf152	0.055633071	0.291800957	0.371656181
UGT1A8	-0.055623444	0.29188459	0.371738928
CABP5	-0.05561264	0.29197848	0.371834725

MCART3P	-0.055609877	0.292002494	0.371841529
ZNF256	0.055585894	0.292210984	0.372083232
NGLY1	0.055582539	0.292240155	0.372096586
ZNF418	0.055574709	0.292308252	0.372159496
ZFP2	-0.055556221	0.292469074	0.372333023
SPACA1	-0.055554743	0.292481939	0.372333023
IQCE	-0.055533937	0.292663004	0.372539708
MPZL1	0.055476042	0.293167234	0.373157706
CLDN9	0.05546277	0.293282908	0.373281084
HMHA1	0.055457642	0.293327611	0.373314121
DNAJC18	-0.055423604	0.29362444	0.373668012
OR1L3	-0.055411491	0.293730121	0.373778617
HLA-B	0.055371649	0.294077901	0.374197266
GRM5	-0.055366386	0.294123857	0.37423183
CCDC11	0.055356981	0.294206001	0.374312433
COMMD8	0.055339329	0.294360218	0.374484715
PHEX	-0.055325928	0.294477339	0.374609786
VTA1	-0.055312518	0.294594558	0.374734965
BSPRY	0.055298363	0.294718335	0.374868471
MUC17	-0.055285611	0.294829864	0.37498638
GOLGA8B	0.055264039	0.295018604	0.375202471
KCNH7	0.055260133	0.295052786	0.375221981
ATP12A	-0.055252564	0.295119035	0.375282267
MORN5	0.055249758	0.295143593	0.375289532
PCDHA6	-0.055235592	0.295267618	0.375423266
AMH	-0.055233178	0.295288751	0.375426167
AGAP7	0.055218982	0.295413073	0.375560252
TNFSF12	0.05518144	0.295742022	0.375954448
MPP3	0.055173701	0.295809862	0.376016685
C21orf71	0.055159362	0.29593559	0.376152493
PLEKHN1	-0.055150128	0.296016571	0.376231413
ADIPOQ	-0.055142336	0.296084923	0.376294272
44077	-0.055136704	0.296134331	0.376333048
LCE2B	-0.055118176	0.296296907	0.376506823
CBWD5	-0.055116811	0.296308885	0.376506823
ADAM6	-0.055096502	0.296487167	0.376709324
LRSAM1	-0.055085671	0.296582274	0.376806124
RAB20	-0.055062024	0.296789987	0.377045967
SPRR2A	-0.055051131	0.296885708	0.377143514
GPR61	-0.055043058	0.296956656	0.377187549
LOC283050	0.055041235	0.296972679	0.377187549
CTSL1	0.055039641	0.296986692	0.377187549
RIMBP3C	0.055038567	0.296996132	0.377187549
SLCO4C1	-0.055027668	0.29709195	0.37728518
C2orf52	0.05501985	0.297160686	0.377348407
GPSM3	0.055011086	0.297237764	0.377422218

RBMY2EP	0.055008758	0.297258238	0.377424151
WDR38	0.054996726	0.297364073	0.377532205
SLC19A1	0.054994773	0.297381256	0.377532205
MGC2752	-0.054977794	0.297530657	0.377697797
H2BFM	0.054972825	0.297574393	0.377729239
CLEC6A	-0.054966314	0.297631703	0.377777907
ZBTB8B	0.054961568	0.29767348	0.377806855
ITIH5L	0.054959051	0.297695643	0.377810906
PFN3	0.054955739	0.297724803	0.377823835
RAB40A	0.054948698	0.297786795	0.377878426
ARHGEF19	0.054941771	0.297847797	0.377915619
TTTY20	0.05494106	0.297854058	0.377915619
MFSD6L	-0.054924567	0.297999339	0.378075863
CT47A10	-0.054919583	0.298043244	0.378107479
SIGMAR1	0.054900556	0.298210919	0.378296098
TTTY4C	-0.054893553	0.298272651	0.378350309
FOLH1B	-0.054886168	0.298337759	0.378408794
OR1D4	-0.054812145	0.29899086	0.379213029
SMARCA4	0.054808745	0.299020878	0.37922695
RELL1	-0.054801918	0.299081167	0.379279256
TMEM146	0.054775267	0.299316592	0.379553641
HSPB2	-0.054752528	0.299517545	0.379784281
ANXA5	-0.054736976	0.299655048	0.379934442
NMT2	-0.054728149	0.299733102	0.380009214
SCARA3	0.054723125	0.299777537	0.380041063
UNC50	-0.054720994	0.29979639	0.380041063
PLXNB1	0.054716214	0.299838667	0.380057936
ZNF577	0.054715174	0.299847868	0.380057936
TAS2R46	-0.054699131	0.299989808	0.380213646
ECEL1	0.054696393	0.300014038	0.380219007
ANKDD1A	0.054694339	0.300032222	0.380219007
SERPIND1	0.054686888	0.300098158	0.380278367
TMC1	-0.054662125	0.300317396	0.380531967
H3F3C	-0.05465615	0.300370308	0.380574799
MYH14	0.054622595	0.30066758	0.380927213
FOXI3	-0.054612727	0.300755044	0.381013785
TUBG2	0.054605046	0.300823132	0.381075802
PKD1L2	-0.054566316	0.301166619	0.381486657
EXTL1	-0.054559907	0.301223477	0.381534411
GYPC	0.054540315	0.301397359	0.381730374
GK	-0.054534028	0.301453171	0.381776782
CD14	-0.05453117	0.301478536	0.381784627
AGPAT4	0.054508215	0.301682386	0.382018485
RSPH10B2	-0.054499033	0.301763948	0.382097471
BCL2L14	0.054426275	0.302410762	0.382892131
ZNF398	-0.05441006	0.302555039	0.383050452

CNNM1	0.054398118	0.302661317	0.383160647
ACTN2	0.054392	0.302715784	0.383205241
ITLN2	0.054358249	0.303016339	0.383561329
SSBP3	-0.054345389	0.303130911	0.383681969
OR8I2	-0.054336719	0.30320817	0.383755367
LCN6	-0.054316101	0.303391951	0.383963567
C20orf203	-0.054308573	0.303459069	0.384024106
ECHDC3	0.054296699	0.303564958	0.384123832
ITGA2B	-0.054295411	0.303576449	0.384123832
ISCA1P1	0.054282762	0.303689276	0.384242181
ZNF211	-0.054269134	0.303810873	0.384371611
GLIS1	-0.054264758	0.303849922	0.384396595
SPRR1B	-0.054244757	0.304028455	0.38457859
ROGDI	0.054244314	0.304032404	0.38457859
CTSH	-0.054240983	0.304062149	0.384591788
FBXL12	-0.054228658	0.304172204	0.384686827
HSPC072	0.054226412	0.304192258	0.384686827
C11orf1	0.054226079	0.304195237	0.384686827
DNAH17	0.054205139	0.304382289	0.384898932
TAC4	-0.054189218	0.304524559	0.385054387
SLC5A10	-0.054183661	0.304574228	0.38509274
FXYD5	0.054181474	0.30459377	0.385093
OBP2B	-0.054170981	0.304687574	0.385187142
NRBP2	-0.054153107	0.304847411	0.385364745
PACS2	0.054142364	0.304943501	0.385461747
GPR84	0.054139162	0.304972147	0.38547349
VENTXP7	0.054136904	0.304992347	0.385474557
ACP5	0.054128599	0.305066649	0.385543997
ELAVL2	-0.054052722	0.305746087	0.386378154
OXTR	-0.054005277	0.306171443	0.386891135
NEK8	-0.053990364	0.306305219	0.387035622
PAK6	0.053956096	0.30661277	0.387399652
KCNQ1OT1	-0.053950672	0.306661463	0.387436594
C4orf17	0.053918976	0.30694614	0.387771655
LOC100286	-0.053897511	0.307139029	0.387990724
ORMDL3	0.053883524	0.307264764	0.388115392
ENPP7	0.053882197	0.307276695	0.388115392
NCRNA0003	-0.053853737	0.307532638	0.388414033
SOX9	0.053850628	0.307560605	0.388424723
CD300C	-0.053837219	0.307681245	0.388552442
RPUSD4	0.053806926	0.307953917	0.388872124
FBXO41	-0.053801772	0.30800032	0.38890606
SLC25A22	0.053791251	0.308095069	0.389001033
PLBD1	0.053786887	0.308134374	0.389025995
LECT1	0.053776395	0.30822889	0.389120656
CR2	-0.05377279	0.308261368	0.389136989

ARMC10	0.053769542	0.308290633	0.389149265
SFRS2	-0.053716413	0.308769573	0.389724854
C11orf71	-0.053714619	0.308785763	0.389724854
CD3EAP	0.053699356	0.308923451	0.3898706
ALDH4A1	-0.053697006	0.308944649	0.3898706
HLF	-0.053695308	0.30895997	0.3898706
ACOX3	-0.05369223	0.308987743	0.389880941
CHRNB3	-0.053671183	0.309177702	0.390095916
ZIC1	0.053664473	0.309238274	0.390147623
EAPP	0.053656049	0.309314339	0.390218869
ETF1	-0.053646875	0.309397185	0.390298659
DEFB123	0.053623507	0.309608285	0.39054022
KLHL22	0.053576157	0.310036308	0.391035501
STOML3	0.053575727	0.310040199	0.391035501
SLC9A10	-0.053570003	0.310091966	0.391053846
LINGO1	-0.053567938	0.310110638	0.391053846
AFF3	-0.053567605	0.310113653	0.391053846
FCRL4	0.053559944	0.310182954	0.391112515
BRS3	0.05355812	0.310199456	0.391112515
RNLS	-0.053546649	0.310303244	0.391218606
PRX	0.053539322	0.310369545	0.391273362
CD37	-0.053536801	0.310392366	0.391273362
C1orf162	0.053535337	0.310405616	0.391273362
NOL3	0.05352422	0.310506246	0.391375436
MEST	-0.053470689	0.310991089	0.391961745
C5orf33	-0.053445389	0.311220419	0.392225962
DUOXA1	-0.053441982	0.311251304	0.392240063
OR2J2	-0.053438112	0.311286399	0.392259469
MUC4	-0.053398955	0.311641592	0.392682209
FCRL2	-0.053393177	0.311694032	0.392723437
BMP15	0.053388635	0.311735248	0.39275052
HIST1H1T	-0.053380914	0.311805338	0.392813974
AWAT2	-0.053328497	0.312281388	0.393388819
KRT76	-0.053321901	0.312341331	0.393439443
ORC3L	-0.053314461	0.312408945	0.393499724
VAT1L	-0.053305169	0.312493416	0.393581228
LOC100130	0.053300249	0.312538141	0.393612665
SERPINA6	0.053283848	0.312687273	0.393775581
BEST2	-0.053274969	0.312768032	0.393852378
PIP4K2B	0.053272747	0.312788244	0.393852927
KLHL13	-0.053258866	0.312914523	0.393987021
CYP4F8	-0.053213317	0.313329148	0.394484131
LOC340074	-0.053207431	0.31338275	0.394526674
FAM151A	-0.05315804	0.313832796	0.395068277
TIMP4	-0.053152528	0.313883049	0.395106562
GPR78	-0.053142759	0.313972127	0.395175974

LILRB2	-0.053142128	0.313977878	0.395175974
ZNF705D	-0.05313605	0.314033311	0.395220765
LAPTM5	0.053129886	0.314089529	0.395266538
OR13D1	-0.053124311	0.314140375	0.395291903
CCDC13	0.053123324	0.314149382	0.395291903
SLC13A4	-0.053107677	0.314292142	0.395446551
CTDP1	0.053098468	0.314376177	0.395527296
TM4SF4	-0.053063312	0.314697141	0.3959061
NMI	0.0530586	0.314740174	0.395935226
CORO1A	0.053043108	0.314881692	0.396088232
TMPRSS6	-0.053036322	0.314943696	0.396141206
RPE65	-0.053032337	0.31498011	0.396156554
LETM1	0.053030633	0.314995684	0.396156554
SIX4	0.053027822	0.315021371	0.396163842
KRT3	-0.053020914	0.315084502	0.396218214
PLCD3	-0.052999701	0.315278428	0.396416372
HMX1	-0.052999322	0.315281894	0.396416372
C7orf71	0.052996148	0.315310917	0.396427836
IBSP	0.052974803	0.315506143	0.396648244
FRG2	-0.05295439	0.315692919	0.396858003
IL22RA2	-0.05293146	0.315902816	0.397096798
CDH16	0.052925567	0.315956765	0.397139547
BRF1	0.05292095	0.315999045	0.397167623
FSCB	0.052902642	0.316166726	0.397353297
NOXA1	0.052892088	0.316263415	0.397449733
MICAL1	0.052886222	0.316317172	0.397492205
CTSB	-0.052872439	0.316443482	0.39762584
COX10	-0.052850021	0.31664901	0.397858993
ZNF555	-0.052815893	0.316962053	0.398227196
ZBTB24	-0.052782749	0.317266264	0.398584257
FAM21C	0.052773004	0.317355744	0.398671522
ADIPOR1	-0.052749802	0.317568854	0.398914074
SCGBL	-0.052747402	0.317590902	0.398916608
VCAM1	-0.05273128	0.317739052	0.39907438
B3GNT9	-0.052727272	0.317775884	0.39907438
LOC348021	0.052727191	0.317776626	0.39907438
PPIL2	-0.052695485	0.318068128	0.39941527
QRICH1	-0.052658803	0.31840559	0.39979998
C19orf12	-0.05265782	0.318414636	0.39979998
TARSL2	-0.052591883	0.319021828	0.400537114
GJD4	0.052581962	0.319113259	0.400626651
RAVER2	-0.052561641	0.319300571	0.40083654
GSTA5	0.05255854	0.319329164	0.400841579
CCDC90B	0.05255684	0.319344841	0.400841579
RHOXF1	0.052548428	0.319422412	0.400913678
PRAMEF20	-0.052506307	0.319811005	0.401376113

FAM174B	-0.052493657	0.319927765	0.40149735
TTF1	-0.052490495	0.319956962	0.401508689
PARP2	0.052466995	0.32017396	0.401755682
ANKRD13B	0.05245093	0.320322358	0.401916567
ACADVL	-0.052429276	0.320522463	0.402142307
DPP7	-0.052415483	0.320649959	0.402276927
B4GALT4	-0.05240909	0.320709073	0.402325744
KCNA6	-0.052394316	0.32084569	0.402471776
TMED10P1	-0.05236786	0.321090437	0.402753421
RASL12	-0.052328938	0.321450731	0.403179954
SYN1	-0.052288183	0.321828268	0.403623983
MYBPH	-0.052286347	0.321845285	0.403623983
MORF4	-0.052196581	0.322677888	0.404642664
C14orf102	-0.052184001	0.322794682	0.404763638
PDE6A	0.052180515	0.322827046	0.404778734
COL4A5	-0.052150263	0.32310804	0.405105554
GLDN	0.052144989	0.323157048	0.405141493
HHLA2	0.052131422	0.323283123	0.405274039
ABCA3	-0.052116051	0.323426007	0.405427641
OR10Q1	-0.052057653	0.323969236	0.40608304
MAP1LC3B	-0.052037413	0.324157644	0.406293629
GZMK	-0.052034383	0.32418586	0.406303424
NUP88	0.052023489	0.324287303	0.406404986
LOC257358	-0.052010104	0.324411978	0.40653565
PLB1	-0.052005826	0.324451831	0.406560009
ZNF673	-0.051999391	0.324511778	0.406609542
OR5C1	0.051976857	0.324721782	0.406847077
CPB2	-0.051966707	0.324816394	0.406940016
IFT46	-0.051963824	0.324843273	0.40694809
C14orf183	-0.051913702	0.3253108	0.407508151
FAM156A	-0.051897797	0.325459249	0.407668467
MS4A3	-0.051881727	0.325609287	0.407830751
TRPM4	-0.051850487	0.325901079	0.408170554
IL18RAP	-0.051833259	0.326062067	0.408346502
LOC283867	0.051819582	0.326189906	0.408480915
RFXAP	-0.051815407	0.326228938	0.408504106
CHD3	-0.051785689	0.32650685	0.408826402
TTTY2	0.051762302	0.326725666	0.409074666
SHROOM1	0.051751025	0.326831214	0.409181092
TBR1	-0.051732908	0.327000825	0.409367704
S100G	0.051724949	0.327075358	0.409435273
HRNBP3	0.051687173	0.327429245	0.409852509
MRPL42P5	-0.051669583	0.327594111	0.410033104
HOXC6	0.051665737	0.327630168	0.410045263
CPSF4L	0.051662613	0.327659458	0.410045263
TRIM72	-0.051661958	0.327665594	0.410045263

YWHAB	0.051642111	0.327851715	0.410252398
ACTR10	-0.051631589	0.327950407	0.41035011
OR52I1	-0.051626635	0.327996885	0.410382482
CBWD3	0.051615194	0.328104235	0.410491006
PKIA	0.051612023	0.328133996	0.410502452
SCML4	-0.051601384	0.328233845	0.410601572
PLCH1	-0.051583123	0.32840528	0.410790224
ARSH	-0.051576323	0.328469129	0.410822678
LOC731779	-0.051575966	0.328472483	0.410822678
JARID2	0.051569777	0.328530602	0.410863062
CDV3	-0.051568134	0.328546034	0.410863062
TMEM185A	0.051551124	0.328705815	0.411037066
TXNDC5	0.051544064	0.328772146	0.411094197
SPANXN3	0.05153508	0.328856568	0.411173943
GDF5	-0.051498571	0.329199789	0.411577237
RNF122	-0.051464344	0.329521761	0.411953916
HIST1H2BO	0.051441771	0.329734218	0.412193644
TAF6L	0.0514348	0.329799853	0.412249817
ANKFN1	0.051425922	0.329883446	0.412311001
LOC151658	0.05142401	0.329901453	0.412311001
SLC6A8	0.051423005	0.329910911	0.412311001
OR4M2	0.051415233	0.329984107	0.412376599
FLJ42627	-0.051407932	0.33005288	0.412406035
RRN3P1	0.051407314	0.330058705	0.412406035
NKAIN3	-0.051406137	0.330069786	0.412406035
RHBDL3	0.051397402	0.330152083	0.412482982
BIRC3	-0.051388683	0.330234232	0.412559735
KIF7	0.051354212	0.33055916	0.412939762
NPDC1	-0.051348746	0.330610705	0.412978247
PER4	-0.051327368	0.330812337	0.413204195
TXNDC11	-0.051305441	0.331019231	0.413436688
C1orf185	0.051268779	0.331365347	0.413834488
ZNF391	-0.051267303	0.331379291	0.413834488
ABCG5	-0.051262695	0.331422811	0.413862885
SEMA5B	-0.051256612	0.33148027	0.413908682
TBP	0.051228929	0.331741828	0.41420931
NXPH3	0.05122498	0.331779146	0.414229935
DCDC2B	-0.051183463	0.332171701	0.414694047
ST3GAL1	-0.051173864	0.332262501	0.414781402
ZNF582	0.051158393	0.332408877	0.414938122
C5orf15	-0.05113468	0.332633326	0.415192271
SPRR2E	-0.05111066	0.332860776	0.415447491
LRRTM3	-0.051108681	0.332879519	0.415447491
RFC5	0.051082257	0.333129864	0.41573388
OR1S2	0.051064376	0.333299335	0.415919309
43892	0.051053642	0.333401098	0.41602023

DAGLA	-0.05103279	0.333598835	0.416240887
EI24	-0.051024286	0.333679496	0.416315447
FBXL21	0.051012177	0.333794378	0.416413846
C6orf114	-0.051011566	0.333800183	0.416413846
CHERP	-0.050994947	0.33395789	0.41658449
OR4F4	-0.050990208	0.334002872	0.41660103
FHAD1	-0.050988384	0.33402019	0.41660103
PLEKHO2	-0.050986939	0.334033906	0.41660103
CCL4	0.050960477	0.334285162	0.416888284
SLC7A1	-0.050937174	0.334506529	0.41713823
LAYN	-0.050928738	0.334586682	0.417212058
LOC731789	0.05092366	0.334634947	0.417235712
MOS	-0.050922333	0.334647554	0.417235712
HYMAI	-0.050916348	0.334704441	0.417280514
MCM8	-0.050887946	0.334974474	0.417591027
EFCAB5	-0.050876899	0.33507954	0.417695858
RAB2A	0.050874219	0.335105034	0.417701493
CACNB4	-0.050866861	0.335175029	0.417762592
SURF4	-0.05086435	0.335198912	0.417766214
FABP5L3	0.050823356	0.335589072	0.418226306
VTN	0.050799942	0.335812049	0.418462226
SNX32	0.050799065	0.335820401	0.418462226
IGSF11	0.050785698	0.335947743	0.418594714
TNFRSF17	0.050774392	0.336055475	0.418702752
FCRLA	-0.050756328	0.336227642	0.418891054
NEUROD2	0.05074079	0.336375785	0.419049403
FOXQ1	0.050720687	0.336567513	0.419252492
SLC44A1	-0.050719282	0.336580911	0.419252492
DPYSL4	0.050710406	0.33666559	0.419295968
FLJ37201	-0.050709742	0.336671923	0.419295968
SLCO1A2	-0.050709003	0.336678977	0.419295968
ARHGDIG	0.050704141	0.336725363	0.419327514
SYT4	0.050698946	0.336774931	0.419363018
EMID1	0.050694576	0.336816638	0.41938873
EPHA6	-0.050661392	0.337133423	0.419756931
CTNNA1	-0.050654144	0.337202638	0.419816862
EVL	-0.050646481	0.337275826	0.419881732
CCDC48	-0.050624942	0.337481597	0.420092385
LOC653544	0.050624354	0.337487224	0.420092385
PSMD11	0.050594708	0.337770576	0.420418814
GAB4	0.050589555	0.33781985	0.420453866
ZNF830	-0.050581593	0.337895986	0.420522344
HSFX2	0.050577795	0.337932301	0.42054126
CA6	-0.050563882	0.338065384	0.420680588
PLA2G4F	0.050545795	0.338238432	0.420869626
CEBPG	0.050534727	0.338344353	0.420958224

C13orf18	-0.050533937	0.33835191	0.420958224
WIT1	0.050531678	0.338373534	0.420958829
GKN2	0.050511928	0.338562608	0.42116774
SLC4A2	0.050496308	0.338712186	0.421327495
ACSM2B	-0.050492221	0.338751337	0.421349876
HPVC1	-0.050461969	0.339041184	0.421684061
HIP1R	-0.050451033	0.339145999	0.421788083
OR2T3	-0.050435412	0.339295754	0.421930233
HLA-DQB2	-0.050434691	0.33930267	0.421930233
CORO2A	0.050420147	0.339442141	0.422077312
SCG5	0.050414992	0.339491582	0.422112435
NKX2-8	-0.050402754	0.339608983	0.422232045
LOC84856	-0.050392764	0.339704837	0.422301115
MBD3L5	-0.050392544	0.339706948	0.422301115
CD33	-0.050387471	0.339755622	0.42233526
KRT34	-0.050382586	0.339802502	0.422367171
LMAN1L	-0.050356385	0.340054032	0.422653438
CALML3	-0.050350531	0.340110235	0.422696913
OBFC2A	-0.050341269	0.34019919	0.422781084
C13orf36	-0.050314462	0.340456719	0.423074726
ANKRD43	0.050300057	0.340595156	0.423220349
SLC10A2	-0.050294203	0.340651426	0.42326386
OR10C1	-0.050289408	0.340697519	0.423294721
KCNJ14	0.050261484	0.340966039	0.423601913
ARSG	-0.050255227	0.341026227	0.423650259
FOSL1	-0.050244902	0.341125559	0.423747225
BPY2	-0.050236115	0.341210099	0.423825804
KLK4	0.05022951	0.34127366	0.423878318
TNNI1	-0.050214693	0.341416281	0.424029013
PSG9	-0.050210602	0.341455669	0.424051487
FTHL17	0.05019767	0.341580181	0.424179667
GJC2	0.050182605	0.341725263	0.424333372
MUC5B	-0.050162387	0.341920044	0.424548767
SERPINB5	-0.050134865	0.342185289	0.424851622
ENO3	0.050117971	0.342348184	0.425027372
LOC116437	-0.050112921	0.342396882	0.425061332
TACC2	-0.050104364	0.342479413	0.425117444
CYP2F1	0.050103808	0.342484774	0.425117444
FGF9	0.050092372	0.342595088	0.42522787
CALML4	0.050086479	0.342651951	0.425271943
HS6ST2	0.05006471	0.342862034	0.425506164
ZNF565	0.05005516	0.342954215	0.425594043
MTFR1	0.050050142	0.343002666	0.425627646
WFDC13	0.050046335	0.34303942	0.425646732
SRP9	0.050011167	0.343379098	0.426041663
KBTBD6	-0.049996196	0.343523763	0.426194601

ACY3	-0.04998437	0.343638065	0.426309853
MYO1F	-0.04997313	0.343746722	0.426406452
HSPA14	0.049971886	0.343758754	0.426406452
GSC	0.049963399	0.343840815	0.426481678
CDHR1	-0.049946945	0.343999941	0.426652477
ATP2A1	0.049941206	0.344055458	0.42669476
LSP1	0.049919202	0.344268366	0.426932219
HEATR7A	0.049890426	0.344546912	0.427251043
RLN3	0.049886159	0.34458823	0.427275674
PLIN5	0.04987763	0.344670826	0.427351482
PUS3	-0.049866389	0.344779708	0.427459869
CABP1	0.049848511	0.344952912	0.427647986
LRRC25	0.0498423	0.345013099	0.427695977
LTV1	0.04982354	0.345194929	0.427894748
ALPP	0.049815817	0.345269797	0.427960914
C4orf3	0.049808376	0.345341944	0.4280237
IQCA1	-0.049768524	0.345728517	0.42847616
IL21	-0.049763059	0.345781555	0.428515225
ARSB	-0.049713703	0.346260736	0.429082357
TAC1	0.049708127	0.346314902	0.429122777
HGFAC	-0.049659492	0.346787551	0.429658115
ANGPTL5	-0.049659231	0.346790085	0.429658115
IQCJ	-0.049649052	0.346889057	0.429754002
GPX7	0.049646643	0.346912491	0.429756299
PCDHA3	-0.049641795	0.346959636	0.429783278
AQP3	-0.049639965	0.346977431	0.429783278
SOX4	0.049617706	0.347193961	0.430006631
ETV7	0.049616064	0.347209937	0.430006631
ZNF781	0.04961477	0.347222527	0.430006631
POTEH	0.04960568	0.34731098	0.430089427
DRGX	-0.049598636	0.347379542	0.430147583
IL15RA	0.049596035	0.347404854	0.430148691
KRT80	-0.049592871	0.34743565	0.430148691
HMGCS1	-0.04959152	0.347448802	0.430148691
IMMT	-0.049589668	0.347466834	0.430148691
HLA-DRA	-0.049584419	0.347517934	0.43018521
CACNA2D2	-0.049533599	0.348012909	0.430749338
C10orf76	0.049533188	0.348016915	0.430749338
EYA4	0.049512661	0.348216971	0.430970169
NKAIN4	0.049509878	0.348244102	0.430976963
ZNF99	-0.049491245	0.34842577	0.431174995
PRTFDC1	0.049486675	0.348470331	0.431203345
ZNF219	0.049451772	0.348810819	0.431597855
HAAO	0.04944464	0.348880425	0.431657161
SCML1	-0.049439032	0.348935154	0.431698055
C1QTNF6	0.04943611	0.348963681	0.431706529

DHRS1	-0.049433009	0.348993946	0.431717153
LOC149837	-0.049415039	0.349169389	0.431907353
CBX4	0.049411301	0.349205896	0.431925682
FAM83E	-0.049395529	0.349359935	0.432089374
NOMO1	0.049356465	0.349741645	0.432534613
C4BPA	-0.049349927	0.349805551	0.432586783
OR52I2	-0.049334319	0.349958163	0.432748638
OR2A12	-0.049330903	0.349991563	0.432763068
WFIKKN1	-0.049323252	0.350066391	0.432828719
NRIP3	-0.049303239	0.350262163	0.433043889
ADK	0.049278532	0.350503945	0.433315914
RFESD	0.04927301	0.350557998	0.43333541
OR10G2	-0.049272475	0.350563233	0.43333541
ZNF793	-0.049245562	0.350826757	0.43363424
DDI1	-0.049234968	0.350930523	0.433735578
GPR97	-0.049186173	0.351408716	0.43428095
CCL7	-0.049185492	0.351415391	0.43428095
SLC26A1	-0.049161891	0.351646827	0.434539995
RAB3B	-0.049139101	0.351870412	0.434789307
PDE7A	0.049128681	0.351972664	0.434888671
CMA1	-0.049118778	0.352069857	0.434973844
ABCB5	0.049117207	0.352085281	0.434973844
DHRS2	0.049108376	0.352171973	0.435046025
STRA6	-0.049106805	0.352187397	0.435046025
FGGY	-0.049098788	0.352266114	0.435116273
HSD17B11	-0.049089169	0.352360576	0.435205959
C18orf55	-0.049074856	0.352501156	0.43535259
WDR49	0.049066501	0.352583236	0.435426959
LOC146481	-0.049063817	0.352609603	0.43543252
FAM66E	-0.049026051	0.35298079	0.435856869
IGSF3	-0.049024402	0.35299701	0.435856869
MGC26647	-0.04901654	0.353074306	0.435925282
CDKL2	-0.049013733	0.353101916	0.435932345
OR5P3	-0.04900078	0.353229311	0.436062592
RNPEP	0.048979841	0.353435302	0.436289843
FLJ90757	0.048924637	0.353978761	0.43693362
BTK	-0.048919208	0.354032237	0.436972544
SPDYE4	0.04891211	0.354102158	0.437031759
PTGR2	-0.048908243	0.354140259	0.437051698
44166	-0.048891212	0.354308071	0.437231704
PRSS16	-0.048883012	0.354388884	0.437286917
REC8	0.048882217	0.354396728	0.437286917
SLC47A1	-0.048878957	0.354428861	0.437299471
TBC1D16	0.048876396	0.354454098	0.437303516
SLC16A11	-0.048860286	0.354612934	0.43745064
LHX3	-0.048859845	0.35461728	0.43745064

LACE1	-0.04884008	0.354812202	0.437663983
ZNF141	-0.048831494	0.354896899	0.437741344
CROCCL1	-0.048824619	0.354964731	0.437797895
SNORA64	-0.048806999	0.355138602	0.437985215
SCNN1B	-0.048797492	0.355232443	0.438073819
SLC18A2	-0.048792251	0.355284181	0.438110493
LOC100272	0.048773761	0.35546675	0.438308484
RBMXL3	0.048711008	0.356086795	0.439045847
HSPA8	-0.048699074	0.356204789	0.439164141
ZNF536	0.04869197	0.356275037	0.439223559
LRRC48	0.048664304	0.356548702	0.439523161
RNF10	0.04866294	0.356562198	0.439523161
NELL1	0.048648487	0.356705215	0.439672239
STMN3	-0.04862501	0.356937612	0.43993146
SLC7A14	0.048617039	0.357016544	0.440001515
SLC38A1	-0.048611345	0.357072931	0.440043776
SNORA34	0.048595451	0.357230353	0.440210536
LRRC8E	-0.048579464	0.357388741	0.440378467
OR4E2	0.04856335	0.357548437	0.440547988
ICA1	0.048543978	0.357740473	0.440757333
UGT3A2	0.048539111	0.357788728	0.440776929
TOX	-0.04853791	0.357800645	0.440776929
LOC440461	0.048526395	0.357914837	0.440870435
FSD1	0.048525791	0.357920823	0.440870435
IL15	-0.048518299	0.357995136	0.440934698
ZNF137	0.048512798	0.358049704	0.440974636
DGKG	0.04850232	0.358153662	0.441075393
ZNF18	-0.048496545	0.358210963	0.441118682
SERPINA3	0.048474482	0.358429936	0.441361045
RAB41	-0.048448701	0.358685913	0.44164894
ASNSD1	0.04843627	0.358809388	0.441773661
HIST1H2AA	-0.048430519	0.35886652	0.441816688
C14orf148	0.048424056	0.358930727	0.44186842
SLC26A3	-0.048421126	0.358959834	0.441876938
CPNE5	-0.048418809	0.358982865	0.441877975
MSH2	-0.048409618	0.359074189	0.441963071
ANKRD55	-0.048377265	0.359395806	0.442331591
SMCP	0.048372927	0.359438948	0.442346414
ZNF876P	-0.048371587	0.359452273	0.442346414
TMEM196	0.048363545	0.359532253	0.442391793
PCP4	0.048363412	0.359533576	0.442391793
OSTCL	-0.048339966	0.359766813	0.442651432
MFI2	-0.048327252	0.359893333	0.442779745
ANKS6	0.048323561	0.359930066	0.442797582
TBC1D3P2	0.048309944	0.360065609	0.44293697
LIPF	-0.048248793	0.360674707	0.44365885

CREG2	0.048218187	0.360979798	0.444006713
ABCA13	-0.048199238	0.361168763	0.444188953
LOC729991	-0.048198857	0.361172569	0.444188953
TRA2B	0.04816976	0.361462865	0.444518523
LSS	0.048158953	0.361570719	0.444623702
POU2F3	-0.048123786	0.361921831	0.445027986
CCDC112	-0.048087857	0.362280772	0.445441846
C6orf141	-0.048075928	0.362399994	0.445544254
UMODL1	0.048075047	0.362408806	0.445544254
DDX53	-0.048071059	0.362448671	0.445565759
NMT1	0.047988884	0.363270689	0.446548718
KIAA1210	0.047935819	0.363802137	0.447174396
CD2	0.04792748	0.363885695	0.447249498
GAK	-0.047907038	0.364090583	0.447473708
IL31	0.047897078	0.364190438	0.447563389
CHPF	-0.047895276	0.3642085	0.447563389
C1QL4	0.047887674	0.364284727	0.44762944
TNFRSF13C	-0.04784832	0.364679494	0.448086879
AFF2	-0.04784265	0.36473639	0.448129138
NCRNA0020	-0.047830089	0.364862468	0.448256388
UBXN6	0.047827081	0.364892656	0.448265823
ZNF114	-0.047820575	0.36495797	0.448318405
PIGL	0.04779955	0.365169082	0.448550068
FLJ33360	0.047774842	0.365417274	0.448827249
CHRNB2	0.047765052	0.365515653	0.448894135
ACAA1	0.047764936	0.365516811	0.448894135
MAK	-0.047759078	0.365575679	0.448938746
MGC57346	0.04774302	0.365737084	0.449109263
TCN1	-0.047729627	0.365871741	0.449246916
TUBB1	0.047709358	0.366075575	0.449469487
ARMC4	-0.047695363	0.366216357	0.449614621
LOC29034	-0.047678094	0.366390123	0.449787054
ZNF433	-0.047676917	0.366401977	0.449787054
TMEM48	-0.047663763	0.366534372	0.449902254
ISCA1	-0.04766142	0.366557955	0.449902254
KLRK1	-0.04766086	0.366563594	0.449902254
ZNF727	-0.047646671	0.366706444	0.450049844
CLEC4GP1	0.04763628	0.36681109	0.450150533
SNX33	0.047608351	0.367092425	0.45046803
WDR5B	-0.04759731	0.367203688	0.450576798
GRHPR	-0.047593225	0.367244856	0.450599551
OR2L3	-0.047585977	0.367317902	0.450661411
SLC16A12	-0.047569122	0.367487818	0.450842105
SLC4A11	0.047563102	0.367548517	0.450888796
CTXN3	0.04755833	0.367596643	0.450920059
LOC642929	0.047526462	0.367918099	0.451286582

C8B	-0.047511201	0.368072093	0.451447665
ZSCAN21	-0.047504102	0.368143752	0.451494133
SP9	0.047502955	0.368155321	0.451494133
UNC119B	-0.047498261	0.368202709	0.451524443
OR4N4	-0.047483929	0.368347401	0.451674065
FOXR2	0.047467601	0.368512296	0.45184844
PNMA5	0.047451406	0.368675886	0.452021195
HSD3B7	0.047437367	0.368817728	0.452142824
CDH17	-0.047437093	0.368820496	0.452142824
NUP107	-0.047416696	0.369026651	0.452367706
LOC151174	0.047412619	0.369067865	0.452390381
POTED	0.047402872	0.369166405	0.452483318
MAT2A	-0.047389118	0.369305491	0.452625937
SMARCC1	0.04738577	0.369339357	0.452639588
CEP164	-0.04738113	0.369386288	0.452669246
MEPE	0.047358531	0.36961491	0.452913823
SALL4	-0.047356906	0.369631351	0.452913823
PATZ1	0.047328247	0.369921426	0.453241368
F8A1	-0.047309268	0.370113588	0.453448915
ELSPBP1	0.047291071	0.370297907	0.453646826
ZNF790	0.04725576	0.370655718	0.454057245
PEA15	0.047252701	0.370686728	0.454067301
ODF2	0.047242638	0.370788743	0.454164329
CPLX1	0.047238056	0.370835199	0.454193295
CCNE2	0.047213257	0.371086694	0.454465441
ATHL1	-0.047211646	0.371103038	0.454465441
TXNDC2	-0.047207991	0.371140111	0.454482894
IL17C	0.047185753	0.371365749	0.45473124
UHRF1BP1	0.047178281	0.371441578	0.45478318
TXNRD1	0.047177073	0.371453839	0.45478318
LRIT2	-0.0471514	0.371714468	0.455072412
MUC21	-0.047148692	0.371741966	0.455072412
FCN1	0.047147051	0.371758628	0.455072412
POU3F4	-0.047144225	0.371787333	0.455079578
PSMD12	0.047133818	0.37189303	0.455180979
MYOC	0.04711807	0.372053003	0.455348793
GPR3	-0.047113406	0.372100392	0.455378806
CX3CL1	-0.047068985	0.372551903	0.455901259
ADAMDEC1	-0.047065466	0.372587686	0.455901259
ADAM11	0.047064651	0.372595977	0.455901259
ZNF14	0.047061052	0.372632579	0.455918032
OR5H2	0.04704917	0.372753426	0.456037872
POU4F2	0.047022713	0.37302259	0.456324714
LOH12CR2	0.047021621	0.37303371	0.456324714
INTS12	-0.047017604	0.373074589	0.456346689
LOC100131	0.046992291	0.373332254	0.456621846

LASS2	-0.046990694	0.37334851	0.456621846
A1CF	0.046988748	0.373368322	0.456621846
KNG1	-0.046955495	0.373707001	0.457007978
GRIK1	-0.046929991	0.373966884	0.457297709
RAP1GAP	0.046907878	0.374192307	0.457545269
RASD1	-0.046897761	0.374295464	0.457616897
GNA15	0.046897626	0.374296843	0.457616897
CLRN2	0.046880464	0.374471876	0.457794132
LRTOMT	0.046878904	0.374487784	0.457794132
H2AFV	0.046862876	0.374651305	0.457965918
DHRS9	0.046857905	0.374702027	0.457999808
C16orf80	-0.046850656	0.374776004	0.458062116
SLC24A2	0.046838413	0.374900969	0.458186732
ZNF320	0.046829276	0.374994244	0.458272605
RDH5	-0.04681939	0.375095181	0.45836783
OR3A4	0.046815027	0.375139734	0.458394147
TARS2	-0.04680694	0.375222318	0.458447684
DHX38	-0.046806228	0.375229588	0.458447684
OR56A3	0.046798806	0.375305396	0.458512174
SEMA4F	-0.046770899	0.375590516	0.45883236
IGFBP1	0.046763356	0.375667607	0.458898387
ITGB2	-0.04675093	0.375794622	0.459025386
ATP13A2	0.046738165	0.375925129	0.459156636
C9orf85	0.046727809	0.376031027	0.459257813
MYL9	-0.046696586	0.376350417	0.459619706
RAB42	0.04668001	0.376520049	0.459798674
TDGF1	0.046671755	0.376604541	0.459873654
NAA50	-0.046655448	0.376771488	0.460049306
ELOVL7	-0.046633102	0.377000332	0.46030051
IFT172	0.046612348	0.377212948	0.46053187
IL17A	0.046604727	0.377291034	0.460598967
LOC729121	0.04656134	0.377735809	0.461113684
ZNF26	0.04653701	0.377985374	0.461390053
DMRTC1B	-0.046523303	0.378126007	0.461533429
FFAR3	-0.046508717	0.378275702	0.461687847
ZNF286A	-0.046497885	0.378386895	0.461795258
MKRN1	0.046437464	0.379007483	0.462524298
SERHL2	-0.046424718	0.379138479	0.46265581
PRPF4	0.046415913	0.379228987	0.462737901
MSX2P1	-0.046396952	0.379423931	0.462947408
KIAA1383	-0.046386666	0.379529711	0.463048104
AGXT2	-0.046374896	0.379650782	0.463167442
CSNK1E	-0.046369616	0.379705097	0.46320533
TM4SF20	-0.046365023	0.379752347	0.463234594
FBN3	0.046333977	0.380071853	0.463595942
ANP32D	0.046285545	0.380570608	0.464175873

FMN2	0.04625314	0.380904531	0.464554703
MLLT6	-0.046248083	0.380956664	0.464589833
GRXCR2	-0.046229703	0.381146164	0.464792472
CALM2	0.046193551	0.381519059	0.465218715
ZNF519	-0.046180104	0.381657823	0.465359428
C14orf138	-0.046135569	0.382117599	0.465891514
COX8C	0.046119136	0.382287342	0.466069937
EIF4EBP2	0.046116702	0.382312482	0.466072055
EML5	0.046084773	0.382642432	0.466445739
OTOP2	0.046074957	0.3827439	0.466540872
YSK4	-0.04606628	0.382833612	0.466621664
HKR1	0.046032184	0.383186244	0.467010023
MED15	0.046030938	0.383199136	0.467010023
RGS1	-0.045984869	0.383675921	0.467562476
AVPI1	0.045978745	0.383739328	0.467611131
ZDHHC22	0.045965591	0.383875551	0.467748507
DONSON	0.045951094	0.384025707	0.467902842
NOVA2	-0.045931587	0.384227813	0.468120451
SNORA70	-0.045919332	0.384354825	0.46824655
SNX3	0.045901731	0.384537271	0.468440161
DGCR2	-0.045896887	0.384587502	0.468472696
MT1IP	-0.045877405	0.384789523	0.468690113
PARVG	-0.045871149	0.384854413	0.468740483
NOD1	-0.045856697	0.385004339	0.468894412
KRTAP11-1	0.045852884	0.3850439	0.468913917
PPP1R8	-0.045847887	0.385095755	0.468948389
OR2V2	0.045844601	0.385129851	0.468961234
WDR43	-0.045835992	0.385219201	0.469041354
C6orf59	-0.045827719	0.385305061	0.469117215
CNOT2	0.045812734	0.385460633	0.469277938
CST9L	0.045799796	0.385594981	0.469387908
SNORA48	0.045799495	0.385598101	0.469387908
PRR25	-0.045778761	0.385813465	0.469620224
DAZ1	-0.045776581	0.385836109	0.469620224
LFNG	-0.045766182	0.385944154	0.469723024
FAM50B	0.045762209	0.38598544	0.469744563
C14orf165	-0.045744737	0.386167015	0.469936823
KRT9	-0.04574196	0.386195888	0.469943243
LOC93432	-0.045736925	0.386248225	0.469978212
SRRM4	0.045719929	0.38642493	0.470164497
FGF4	0.045715835	0.386467504	0.47018757
LOC55908	0.045632977	0.38732975	0.471207817
DRD3	0.045581572	0.38786527	0.471830484
DPAGT1	0.045577631	0.387906337	0.471851619
LRRC37B2	0.045525104	0.388454046	0.472488996
RFX8	-0.045513725	0.388572762	0.472604529

OR52E6	0.045498857	0.388727903	0.472764347
EPPK1	0.045492178	0.388797616	0.472798044
PIK3C2G	-0.045491653	0.388803091	0.472798044
CACNA2D3	-0.045458976	0.389144245	0.473184005
PMP2	-0.04544482	0.389292084	0.473334871
ACCN5	-0.045440099	0.389341403	0.473361893
ATXN10	0.045438142	0.389361846	0.473361893
VCP	0.04543178	0.389428309	0.473413794
RNASEN	0.045428308	0.389464582	0.47342899
RNASE8	0.045409854	0.389657418	0.473612361
ADH5	0.045409321	0.389662995	0.473612361
KLK12	-0.045396317	0.38979891	0.473735882
PAEP	-0.045395046	0.389812197	0.473735882
UCKL1AS	0.045359153	0.390187526	0.474163081
ADM	-0.045352041	0.39026192	0.474224549
C3orf74	-0.045349071	0.390292988	0.474233365
CHAD	0.045344973	0.390335865	0.474256529
FAIM	0.04533797	0.390409131	0.474316608
C4orf50	0.045326831	0.390525696	0.474429283
SEPHS1	-0.045303131	0.390773775	0.4746685
HECW1	-0.04530046	0.390801736	0.4746685
TSPAN1	0.045298825	0.39081885	0.4746685
CNR1	-0.045296783	0.390840231	0.4746685
GGN	-0.045295288	0.390855885	0.4746685
CDKN2B	-0.045294359	0.390865616	0.4746685
QDPR	0.045267823	0.391143529	0.474977036
ABCB8	0.045248058	0.391350608	0.475171447
ADAP2	-0.045247987	0.391351347	0.475171447
FAM123A	0.045241454	0.39141981	0.4752256
MTNR1B	-0.045238445	0.391451342	0.475234911
CDH2	-0.045235008	0.391487363	0.47524967
CAPZA3	0.045217742	0.391668355	0.475440406
A1BG	0.04519921	0.39186267	0.475624067
EPN2	-0.045198757	0.391867421	0.475624067
AKIRIN2	0.045194202	0.391915191	0.475653057
AHSA2	-0.045166033	0.392210691	0.475981174
EPHA10	-0.045163874	0.392233344	0.475981174
SPTAN1	-0.045159006	0.392284421	0.476014151
LOC344967	-0.045152712	0.392350472	0.476065293
REEP6	-0.045141206	0.392471237	0.476182813
ME3	-0.045133184	0.392555449	0.476255971
HMOX1	0.04512715	0.392618795	0.476287856
TSKS	0.04512606	0.392630245	0.476287856
FXYD7	0.045121697	0.392676047	0.476287856
PDE6C	0.045121569	0.392677394	0.476287856
OR7A5	0.04511322	0.392765062	0.476365178

ZNF84	-0.045075261	0.393163815	0.476819766
C8orf71	0.045051513	0.393413411	0.477093417
CD38	-0.045047193	0.393458826	0.477119437
PGA4	-0.045009244	0.393857894	0.477574279
TCN2	0.045001025	0.393944353	0.477650033
TREX2	-0.044985044	0.3941125	0.477824817
BCL9	-0.044978921	0.394176942	0.477873854
TFAP2A	0.044972593	0.394243535	0.477925493
IL1F8	-0.044958173	0.394395335	0.478080412
MAGEH1	0.044944789	0.39453625	0.47822212
HYLS1	0.044916253	0.394836802	0.478557295
MYO1H	0.044901547	0.394991753	0.478715966
CSPG5	0.044898237	0.395026632	0.478729105
VSIG8	0.044895711	0.395053244	0.478732223
ALS2CR4	0.044892782	0.395084113	0.478740499
PATE4	-0.044880139	0.395217367	0.478872832
PML	-0.044870744	0.395316408	0.478963696
MYC	0.044852503	0.395508745	0.479162159
HIST1H3J	-0.044848397	0.395552051	0.479162159
NMBR	-0.044848005	0.39555618	0.479162159
NKX1-2	-0.044846083	0.395576453	0.479162159
ACOX2	-0.04484134	0.395626484	0.479172995
PDGFRL	0.044840672	0.395633521	0.479172995
CHCHD3	0.044835925	0.3956836	0.479204506
WDR1	-0.044801606	0.396045709	0.479613882
C8orf58	-0.044784023	0.396231308	0.479809467
DYNC1H1	-0.044767187	0.396409081	0.479995552
PSMD10	-0.044757088	0.39651573	0.48009314
SULT1C3	0.04475499	0.396537889	0.48009314
SASH3	-0.044733402	0.396765942	0.480340045
OR1N2	0.044720055	0.396906983	0.480454385
RILPL1	0.044719898	0.396908639	0.480454385
CTSW	0.044672669	0.397407955	0.481001084
STK31	0.04467261	0.397408579	0.481001084
DCC	-0.044653105	0.39761489	0.481221546
TRIM29	-0.044630919	0.397849653	0.481476412
IFLTD1	0.044626041	0.39790128	0.48150963
CD83	0.044608631	0.398085574	0.481703378
RHOF	0.044590333	0.398279321	0.481908542
FLJ43860	0.044556381	0.398638964	0.482314396
LOC389332	0.044545827	0.3987508	0.482420399
PSG1	-0.044526929	0.398951107	0.482633416
C18orf8	-0.044519745	0.399027259	0.48269622
MBD3L1	0.044480445	0.399444053	0.48317106
HIST3H2BB	-0.044466524	0.399591745	0.483320352
TRIM74	-0.044445115	0.39981895	0.483565796

TMEM174	-0.044435043	0.399925875	0.483665743
GRIA2	0.044424769	0.400034953	0.483768284
STRC	0.044409556	0.400196503	0.48391606
OBP2A	-0.044408685	0.40020575	0.48391606
ATP6V1G1	0.044403494	0.400260885	0.483953343
CLDN7	0.044392216	0.400380691	0.484060779
OTOP1	0.044390553	0.400398353	0.484060779
FGD3	0.044384932	0.400458078	0.484103594
SLC36A4	-0.044375207	0.400561407	0.484180202
NOVA1	-0.044374391	0.400570074	0.484180202
ENGASE	-0.044350262	0.400826533	0.484460788
CASP4	0.044346215	0.400869558	0.484483387
AZIN1	-0.044341764	0.400916871	0.484489439
LEFTY1	0.044341167	0.400923221	0.484489439
PGLYRP4	-0.044331062	0.401030662	0.48458987
C19orf75	0.044327727	0.401066134	0.484603328
MAGEB18	0.044311659	0.401237023	0.484763865
ZNF215	0.044310657	0.40124768	0.484763865
ABHD4	-0.044290279	0.401464484	0.484996373
EDN3	-0.04427973	0.401576746	0.485102565
TBX21	-0.044274503	0.40163237	0.485140332
GRIN1	0.044264818	0.401735455	0.485235419
LTK	-0.044244711	0.401949534	0.48546455
FAM8A1	-0.044241389	0.401984903	0.485477826
LIN9	0.044224717	0.402162466	0.485662817
OVOL1	-0.044210981	0.402308796	0.48581007
MGST1	0.044197156	0.402456103	0.485958485
BIRC8	0.044192876	0.402501714	0.485974788
C7orf65	0.044191309	0.40251841	0.485974788
GBX2	-0.044180207	0.40263674	0.48606465
ECD	-0.044179746	0.402641653	0.48606465
FAM181A	0.04416864	0.402760042	0.48613953
FLJ25758	0.04416839	0.402762714	0.48613953
C21orf130	-0.044167058	0.402776914	0.48613953
LOC360030	0.044158132	0.402872079	0.486224922
LOC100133	0.044148601	0.402973722	0.486318124
NCRNA0023	-0.044132366	0.403146877	0.486497609
SCGB2A2	-0.044126395	0.403210576	0.486544996
KCNH6	-0.044121551	0.403262251	0.486577869
CST5	-0.044117525	0.403305208	0.486600219
HAP1	-0.044111179	0.403372919	0.48664618
RANBP17	0.044108363	0.403402972	0.48664618
TTTY1B	-0.044107085	0.40341661	0.48664618
C17orf28	0.04410406	0.403448886	0.48664807
GOLGA8F	0.044102359	0.403467049	0.48664807
KBTBD12	-0.044077348	0.403734019	0.486940588

OR2D2	0.044029257	0.404247664	0.487530565
NPPA	0.044014819	0.404401936	0.487687088
TMC4	-0.043995472	0.404608731	0.487906925
LOC643486	-0.043978498	0.404790219	0.488096222
LYZL6	0.0438969	0.40566331	0.489119382
DIO3OS	-0.043870709	0.405943798	0.489404734
LOC727677	-0.043870211	0.405949124	0.489404734
PNLIPRP1	0.04385745	0.406085831	0.489539911
MTTP	0.043850826	0.406156794	0.489595821
DUS4L	0.043835283	0.406323353	0.489766952
GRK1	0.043828453	0.406396555	0.489813049
OTOP3	-0.043827125	0.406410787	0.489813049
HCK	-0.043815652	0.406533782	0.489931635
PTPN20B	0.043792404	0.406783054	0.49020238
MOBP	-0.043785678	0.406855191	0.490259644
SMN1	-0.04378245	0.40688981	0.490271695
GALK2	-0.043754254	0.407192316	0.490606508
GOLGA2P3	-0.043747696	0.407262685	0.490661607
ADAMTSL5	-0.043742432	0.40731918	0.490699986
SMR3A	-0.043738933	0.407356742	0.490715553
KCNA2	-0.043712632	0.407639099	0.49101715
TNFRSF6B	0.043711019	0.407656417	0.49101715
FLJ40504	-0.043704389	0.407727609	0.491073198
FASLG	-0.043684217	0.407944281	0.491304448
SGPP2	-0.04367837	0.408007095	0.491331747
GCM2	-0.043677514	0.408016291	0.491331747
NAGLU	0.043664735	0.408153599	0.491467376
C8orf48	-0.043647259	0.408341419	0.491663806
GAA	-0.043616001	0.408677477	0.492038689
RPL3L	0.043606316	0.408781647	0.492134355
IRF2BP2	-0.043602313	0.408824695	0.492156428
CLEC11A	-0.043595432	0.408898719	0.492203182
PAQR5	-0.043594108	0.408912963	0.492203182
OR5V1	-0.043586134	0.408998742	0.49227668
PURG	-0.043579086	0.409074586	0.492338211
DSCAML1	-0.043559623	0.409284034	0.492560523
IL29	0.043554067	0.409343844	0.492602734
LPO	0.043549743	0.409390394	0.492628985
NPY6R	-0.043540877	0.409485837	0.492710482
TMC8	-0.043538856	0.409507602	0.492710482
HMX3	-0.043534851	0.409550727	0.4927326
PPP1R1C	0.043528461	0.409619529	0.492760938
KRTAP1-5	-0.043528068	0.409623767	0.492760938
ALKBH1	-0.043518394	0.409727952	0.492856497
RNF113B	-0.043502302	0.409901287	0.493035219
KRTAP10-12	-0.043495976	0.409969437	0.493081756

ATAD5	-0.043494115	0.409989495	0.493081756
GPR114	-0.04347146	0.410233627	0.493345572
GALNT14	-0.04343789	0.410595554	0.493751008
DHRS12	0.04340974	0.410899188	0.494086302
AACS	0.043403355	0.410968069	0.494139292
KRT74	0.043396634	0.41104059	0.494196653
SLC13A3	0.043393045	0.411079318	0.494213379
FADS2	-0.043368676	0.411342347	0.494499749
MT1G	0.043362062	0.411413749	0.494555732
LAT2	-0.043343071	0.41161882	0.494772381
MAGEB6	0.043311839	0.411956206	0.495148038
WDR27	-0.043300804	0.41207544	0.495261459
BLNK	-0.043294562	0.412142908	0.495292694
C7orf66	0.043293797	0.412151169	0.495292694
FAM174A	0.04328412	0.412255772	0.495388506
SIGLEC9	-0.04326154	0.412499889	0.495651943
GOT1	-0.043217046	0.412981194	0.496200331
CYP4Z1	-0.043173415	0.413453479	0.496737816
OR2Z1	0.043169814	0.413492464	0.496754685
MMP25	0.04314134	0.413800872	0.497052508
MSC	-0.043139365	0.413822271	0.497052508
LRRC16B	0.043139	0.413826224	0.497052508
SYT14L	-0.043136416	0.413854215	0.497052508
C1orf114	-0.043135406	0.413865162	0.497052508
GRTP1	0.043127115	0.413955	0.497130425
ZDHHC11	-0.043118323	0.414050267	0.49721485
PADI6	0.04311509	0.414085308	0.497226947
FBXL19	0.043108032	0.414161811	0.497288826
SCARNA1	0.043092268	0.414332695	0.497464015
CELA3B	0.043020949	0.415106343	0.498362842
ZNF506	-0.043009972	0.415225492	0.498475838
LOC100133	-0.043001183	0.415320905	0.498560326
ABCC13	0.04298269	0.41552171	0.498771313
LOC284023	-0.042929641	0.41609806	0.499433032
APOBEC4	-0.042923222	0.416167831	0.499486673
LOC285205	0.042889166	0.416538116	0.499900963
CLVS2	0.042885424	0.416578807	0.499919671
SCARNA7	0.042870756	0.416738361	0.500054639
ZNF711	0.042870468	0.416741493	0.500054639
ALOX5AP	-0.042844648	0.417022435	0.500361598
SYT13	-0.042831917	0.417161011	0.500497712
NKX2-1	-0.04281619	0.417332221	0.500672963
DYX1C1	0.04280748	0.417427062	0.500756577
NEU4	0.042766738	0.417870849	0.501258761
HHLA1	0.042759664	0.417947929	0.501320145
FITM2	-0.042757421	0.417972367	0.501320145

SPRYD4	-0.04275042	0.418048663	0.501363259
SNORA49	-0.042749503	0.418058663	0.501363259
WNT10A	0.042734117	0.418226378	0.501534191
KRTAP12-3	-0.042724368	0.418332662	0.501609917
LOC374443	0.042723704	0.4183399	0.501609917
OAT	-0.042720344	0.418376532	0.501623638
PLSCR3	0.042706504	0.418527463	0.501774392
CSF2RA	-0.042680823	0.418807596	0.502064591
ACPL2	-0.04267698	0.418849523	0.502064591
CNGA3	0.042675525	0.418865393	0.502064591
C7orf4	-0.04267507	0.418870358	0.502064591
DEPDC6	-0.042664256	0.418988365	0.502175812
DCX	-0.042657517	0.41906191	0.502233732
PSAPL1	0.042641666	0.419234939	0.502410868
CTSD	0.042632827	0.419331434	0.50249627
NPHP1	0.042628519	0.419378481	0.502521977
PROK1	-0.042626241	0.419403353	0.502521977
C10orf28	-0.042622948	0.419439305	0.502534819
OR5K4	0.042606433	0.419619673	0.502720676
VWC2	-0.042600407	0.419685493	0.502769285
LOC100130	0.042568548	0.420033596	0.503156034
PGAM2	0.042560664	0.420119773	0.503228996
C8orf85	0.042537381	0.420374311	0.503503603
CXCL3	0.042514643	0.420622975	0.503723043
HES5	0.042514498	0.420624559	0.503723043
SP140	0.04251369	0.420633402	0.503723043
C9orf11	0.042501231	0.420769686	0.503851897
PPRC1	0.042499228	0.420791601	0.503851897
NANOG	-0.042488397	0.420910106	0.503963492
BPESC1	0.042470657	0.421104256	0.504165157
BST2	0.042468381	0.421129168	0.504165157
HAVCR2	-0.042460177	0.421218965	0.504242347
IGFBP6	0.042441612	0.421422225	0.504455347
TNFRSF1A	-0.042397924	0.421900781	0.504997839
C12orf77	0.042375941	0.422141698	0.50525584
NR1H4	-0.042354122	0.422380894	0.505511749
OR10A2	-0.042350831	0.422416983	0.505524561
CCDC79	-0.042342984	0.422503027	0.505597151
PERP	0.042328914	0.422657348	0.505751432
DHCR7	0.04232627	0.42268635	0.505755747
PSG7	0.042316525	0.422793249	0.505853263
STAC2	-0.042309149	0.422874173	0.505919689
PLOD2	0.042304253	0.422927897	0.505953569
MED26	0.042299588	0.422979091	0.505984418
CATSPER2P	-0.042293387	0.423047143	0.506035429
RAB7A	-0.042288951	0.423095831	0.506063272

PYROXD2	-0.042283082	0.423160246	0.506109921
RASGEF1A	0.042272195	0.423279755	0.506222455
PRAMEF14	-0.042261071	0.423401894	0.50633812
HIST2H3D	-0.042228738	0.423756999	0.506732355
COX6A2	0.042223309	0.423816637	0.506773241
CST2	0.042217022	0.423885713	0.506825407
FIG4	-0.042195602	0.424121102	0.507076409
PRDM14	0.042187165	0.424213835	0.507156833
PLCB1	0.042156786	0.424547859	0.507525699
TSPAN6	0.042115657	0.425000313	0.508036088
MMP17	0.042078937	0.425404494	0.508488715
SLMO2	-0.042067492	0.425530517	0.508608826
CCT6P1	-0.04206297	0.425580315	0.508637819
OR2T8	-0.04203905	0.425843797	0.508922181
LOC285045	-0.042015617	0.426102006	0.509185714
PEX11B	0.042014397	0.426115446	0.509185714
RNASE10	-0.04200487	0.426220452	0.509280633
SLC6A15	0.042001608	0.426256406	0.509293036
SLC6A18	-0.041988248	0.426403699	0.509436908
FNDC4	-0.04198549	0.426434101	0.509436908
ESRP2	-0.041983725	0.426453563	0.509436908
CLEC4A	-0.041978883	0.426506952	0.509452528
SLC17A8	-0.041977899	0.4265178	0.509452528
LGALS9C	0.041974386	0.426556546	0.509468251
SREBF1	-0.041971628	0.426586959	0.50947402
ZPBP	-0.041957125	0.426746918	0.509634498
PSMB11	0.041930877	0.427036505	0.509920607
MCOLN2	-0.041930768	0.427037705	0.509920607
SLC38A11	-0.041927587	0.427072807	0.509931947
SNORA16A	-0.041923178	0.427121461	0.509959466
PMCHL1	0.041919869	0.427157981	0.509972495
ANKMY1	0.041901782	0.427357628	0.510180264
ST7L	-0.041881002	0.427587076	0.510423582
XKR7	0.04185239	0.427903109	0.510770224
DFNB31	-0.04184885	0.427942211	0.510786283
C12orf11	-0.041834501	0.42810077	0.510944914
CD180	-0.041826109	0.428193511	0.511024975
TES	-0.041810664	0.428364228	0.511198083
FLJ44054	-0.041785963	0.428637341	0.511493119
SNAP91	0.041783659	0.428662825	0.511493119
SLC16A1	-0.041775568	0.428752308	0.511569242
GLTPD1	-0.041764642	0.42887317	0.511653772
BPGM	-0.041764518	0.428874538	0.511653772
KPNA4	-0.041751582	0.429017652	0.511793851
IRX5	-0.041747333	0.429064671	0.511819282
CALB1	0.041721786	0.429347406	0.512125874

TRIM48	-0.041708153	0.429498335	0.512275219
VN1R5	-0.041697555	0.429615672	0.512384483
TARBP1	0.04168209	0.429786951	0.512558065
GHRH	0.041667215	0.429951719	0.512705872
CTRB1	0.041666253	0.429962379	0.512705872
CHODL	0.041651364	0.430127341	0.512871871
RAET1K	-0.041628814	0.430377268	0.513139154
DRD2	0.041615552	0.43052429	0.513283718
CD300LD	-0.041606899	0.430620225	0.513367362
LCE1E	0.04159923	0.430705275	0.513438019
TRMT5	0.041596539	0.430735115	0.513442857
KRTAP8-1	0.041591858	0.430787025	0.513474
MTRF1L	-0.041572024	0.431007034	0.513705493
C8orf74	-0.04156714	0.43106122	0.51373933
DBX1	0.041557126	0.431172339	0.513841011
TNIP3	-0.041546769	0.431287265	0.513947217
KNTC1	0.041490957	0.431906948	0.514654872
SNORA46	-0.041474125	0.432093927	0.514846868
FLJ42709	-0.041457075	0.432283381	0.515040733
SLC2A4	-0.041454828	0.432308355	0.515040733
NXF5	0.041432027	0.432561799	0.515311852
SCGB1D2	0.041416236	0.432737369	0.515490172
KCNK12	-0.041411944	0.432785094	0.515516188
CST6	-0.04138172	0.43312128	0.515882947
LRP4	-0.041379606	0.433144803	0.515882947
CCDC38	0.04136644	0.433291297	0.516026562
ZAP70	-0.041356454	0.433402432	0.516128053
ASTE1	0.041349486	0.433479984	0.51618954
MYT1	0.041336491	0.433624642	0.516330927
GTPBP10	-0.041326765	0.433732925	0.516428984
AZGP1	0.041303667	0.433990165	0.516680067
TPRG1L	0.041301622	0.434012933	0.516680067
SLITRK1	0.041300841	0.434021634	0.516680067
RAB39B	-0.041267338	0.43439491	0.517093522
DEFB109P1B	-0.041236965	0.434733473	0.51746561
TMEM155	0.041230817	0.434802029	0.517516281
LEPREL2	-0.041225969	0.434856083	0.517549686
CRADD	-0.041220637	0.434915546	0.517589524
TCTN3	-0.041213542	0.434994674	0.517639248
NCKAP5L	0.041212229	0.435009312	0.517639248
LOC285735	-0.041206996	0.435067682	0.517677773
TCEAL8	-0.041197486	0.435173764	0.517773062
FAM95B1	-0.041158669	0.435606928	0.51825748
OR6F1	0.041130449	0.435921993	0.518601344
FOXD4L3	-0.041117083	0.436071263	0.518724293
XKR4	-0.04111653	0.436077435	0.518724293

TNFRSF25	0.041107323	0.436180276	0.518815637
UBXN2A	-0.041066018	0.436641819	0.519333601
CGRRF1	0.041043848	0.436889666	0.519597354
LCN8	-0.041036009	0.436977318	0.519670566
FAM163A	-0.04103236	0.437018125	0.519688062
GIF	0.041030004	0.437044468	0.519688355
SNORA14B	-0.040994286	0.437444016	0.520132401
MYBPC3	0.040990838	0.437482597	0.520147219
SPANXN1	0.040980097	0.437602796	0.520259069
RELT	-0.040936432	0.438091628	0.520809143
PLUNC	0.040924616	0.438223969	0.520935016
C1orf93	0.040922307	0.438249825	0.520935016
HCRTR2	-0.040919156	0.438285128	0.520945886
BLCAP	0.040916313	0.438316966	0.520952637
SPATA3	0.040880926	0.43871348	0.521392788
LGALS4	0.040834059	0.439238934	0.521966063
FUT5	0.040833226	0.439248268	0.521966063
CSNK1G2	-0.040822527	0.439368279	0.522077522
FAHD2B	0.040811402	0.439493076	0.522194655
CARD9	0.040805628	0.439557866	0.522240478
ADAM21	-0.040795062	0.439676425	0.522350176
SLC45A4	-0.040771806	0.439937437	0.522608585
FAM35B2	-0.040770154	0.439955982	0.522608585
MCM4	-0.040768669	0.439972661	0.522608585
LOC100128	-0.040746078	0.440226308	0.52285139
ISCU	0.040745786	0.440229581	0.52285139
NEK11	0.040733955	0.440362456	0.52297538
GAST	0.040731815	0.440386499	0.52297538
KRT6B	-0.040729384	0.440413799	0.522976615
FDXACB1	-0.040714214	0.440584216	0.523143245
VLDLR	-0.040712216	0.44060666	0.523143245
SGCE	-0.040705961	0.440676946	0.523184913
C12orf36	-0.040704417	0.440694296	0.523184913
RXFP3	-0.040702025	0.440721172	0.52318563
ZNF681	0.040671715	0.441061842	0.523558835
GRIK2	-0.040650329	0.441302303	0.52381305
HOOK2	-0.040640737	0.441410173	0.523909863
ATPAF1	-0.040610456	0.441750824	0.524281888
BASE	0.040608195	0.441776267	0.524281888
CACNA1B	-0.040595157	0.44192299	0.524406735
UFSP2	0.040594167	0.441934131	0.524406735
CLEC2B	0.04058563	0.442030212	0.524489495
MYL3	0.040564609	0.442266867	0.524739032
RFPL1	-0.040546036	0.442476015	0.524955906
MYT1L	-0.040538814	0.442557365	0.525021143
TP53	-0.04053056	0.442650339	0.525082015

RASGEF1C	0.040529577	0.442661408	0.525082015
C6orf225	0.040519915	0.442770257	0.52517985
MTERFD3	0.040517213	0.442800706	0.525184685
GPR133	-0.040502938	0.442961563	0.525344184
SGTB	-0.040476746	0.443256793	0.525663016
SRR	-0.040465012	0.443389088	0.525788596
LOC285796	-0.040452352	0.443531849	0.525898664
ACTR3C	0.040452098	0.44353472	0.525898664
LPAR3	-0.040441713	0.443651852	0.526006229
SLC27A5	-0.040403166	0.444086762	0.526490527
CFHR1	0.040392841	0.444203301	0.526597341
CCNA1	-0.04038519	0.444289663	0.526623306
C15orf57	-0.040384693	0.444295273	0.526623306
PPIF	-0.040383873	0.444304533	0.526623306
TAAR5	-0.040380945	0.44433758	0.526631132
CYP7A1	-0.040366058	0.444505669	0.526793518
OR6M1	0.040364125	0.444527495	0.526793518
YIPF7	0.040297707	0.445277858	0.527651347
TMPRSS11B	0.040275953	0.445523784	0.527911356
C19orf6	-0.040248386	0.445835534	0.528249326
RCN1	0.040236371	0.445971451	0.528378931
KLRC3	0.040224994	0.446100166	0.528483293
SERPINB4	-0.040223894	0.446112611	0.528483293
SLC43A2	-0.040216578	0.446195395	0.528549923
CADPS	-0.040201344	0.446367811	0.528722712
TSC2	-0.040188058	0.446518204	0.528869396
AIG1	0.040181373	0.446593893	0.528927587
CASP14	-0.040159262	0.446844271	0.529192654
CCDC117	-0.040099478	0.447521653	0.529963353
NES	-0.0400928	0.447597348	0.530021477
MYBPHL	0.04008489	0.44768702	0.530075085
GFRA2	-0.040084111	0.447695854	0.530075085
C7orf57	0.040072254	0.447830296	0.530202743
PRSS33	0.040066137	0.447899667	0.530253351
TMEM109	0.040030839	0.448300069	0.530695826
GRP	-0.040026478	0.448349556	0.53072286
KIAA0907	-0.03997826	0.448896884	0.531284614
MRGPRX1	0.039977816	0.448901919	0.531284614
SIX6	0.039975682	0.448926156	0.531284614
MAN2C1	0.03997527	0.44893083	0.531284614
AMY2A	-0.039961791	0.44908391	0.531434194
OTOL1	-0.039958235	0.449124295	0.531450406
ITGB4	-0.039953761	0.449175117	0.531478964
SERHL	0.039909524	0.449677761	0.5320421
LRGUK	0.03989597	0.449831831	0.532192771
TRIM59	0.03989024	0.449896972	0.53223822

ZP2	-0.039883496	0.449973649	0.532297311
C17orf54	-0.039877496	0.450041875	0.532324405
TMEM63C	0.039876513	0.45005306	0.532324405
EVPLL	-0.03987443	0.450076743	0.532324405
SF3B3	-0.039868934	0.450139243	0.53236671
SPNS3	-0.03984849	0.450371779	0.532610094
C22orf42	-0.039831456	0.450565586	0.532807651
OR5E1P	-0.039827177	0.450614274	0.532833587
CLEC12A	-0.039798939	0.450935672	0.533181969
KIF24	-0.039793414	0.450998568	0.533224679
ZNF385D	-0.039747703	0.45151916	0.533808494
UPK3B	0.039743435	0.451567784	0.533834288
DYM	0.039733145	0.451685027	0.533941195
USP20	0.039689413	0.452183493	0.53449871
BTN3A2	-0.039671061	0.452392768	0.534714343
OR4N2	-0.039664609	0.452466359	0.534769587
OR10G3	-0.039620511	0.452969486	0.535315924
BCL3	0.039619382	0.452982373	0.535315924
APOC4	0.039602241	0.453178029	0.535515366
CEACAM18	-0.039576793	0.45346859	0.535805308
SNORD97	0.039576039	0.453477201	0.535805308
SLC7A5	-0.03957281	0.453514076	0.535817087
FBXO9	0.039565537	0.453597137	0.53588343
POU1F1	0.039516872	0.454153174	0.536498297
RFPL2	0.039515272	0.454171468	0.536498297
TNK2	0.039492837	0.454427932	0.536769411
ZNF140	-0.039471045	0.454677132	0.537031913
USP36	-0.039440593	0.455025485	0.537411489
TDG	-0.039412808	0.455343464	0.537755149
ACSM3	-0.039379176	0.45572853	0.538177993
FAM162B	-0.039376616	0.455757846	0.5381807
ABCA12	-0.039372465	0.455805386	0.538204925
TUSC1	-0.039365283	0.455887646	0.538270141
ICAM1	-0.039350798	0.456053571	0.538431562
ZNF512B	-0.039348628	0.456078434	0.538431562
OR52B4	-0.039336313	0.45621954	0.538538002
SF1	-0.039336039	0.456222678	0.538538002
AGBL3	-0.039311661	0.456502068	0.538835863
ZNF331	0.039252343	0.457182307	0.539606806
PDIA4	-0.039236106	0.4573686	0.539794694
GPR115	-0.039231271	0.457424079	0.53982818
ID2B	-0.039225996	0.457484615	0.539854411
UTP23	0.039223359	0.457514879	0.539854411
CSAG2	0.039222248	0.45752763	0.539854411
CHD5	0.039217531	0.457581771	0.539886306
RBP4	0.039196313	0.457825346	0.540141692

MGC16025	0.039190211	0.457895412	0.540192354
IL28A	-0.039166112	0.458172173	0.54048684
SORBS3	-0.039131623	0.458568414	0.540922227
PAK7	0.039087424	0.459076489	0.541489474
PRR5-ARHG	-0.039078087	0.459183869	0.541584054
GOLGA3	0.039068231	0.459297221	0.541685666
LOC100128	0.039018057	0.459874522	0.542334405
CLCN2	-0.03901405	0.459920645	0.542336029
GINS3	0.039013206	0.459930364	0.542336029
SPRR2F	0.038997064	0.460116184	0.54252302
FTCD	0.038976614	0.460351668	0.542768543
PEX5	-0.038958071	0.460565252	0.542988219
IL28B	-0.038929024	0.460899935	0.54335063
C4orf51	0.038920851	0.460994123	0.543429499
EPS8L1	0.038911839	0.461098001	0.543511204
ZNF808	-0.038910102	0.461118017	0.543511204
SYT8	0.038904786	0.461179299	0.543551266
FOXL2	0.038872681	0.461549501	0.543955398
CHRND	-0.038859415	0.461702509	0.544103525
LY9	-0.038856886	0.461731682	0.544105707
ACOT1	0.038841109	0.461913709	0.544288002
CLEC4F	-0.038800866	0.462378179	0.544803066
UBE2A	-0.038782984	0.462584648	0.545014096
ANKRD30B	0.038775557	0.46267042	0.54508058
ASTL	-0.038773358	0.462695817	0.54508058
ARHGEF33	-0.038761823	0.462829046	0.545144614
UNKL	-0.038760218	0.462847588	0.545144614
JAK3	0.03876011	0.462848831	0.545144614
PITX2	-0.038757041	0.462884278	0.545144614
RMST	-0.038756802	0.462887041	0.545144614
ARMC6	0.03870997	0.463428212	0.545749681
CCDC140	0.038705511	0.463479763	0.545778115
EVC2	-0.038700671	0.463535709	0.545811722
FAM183B	-0.038695572	0.463594664	0.545848866
C15orf44	-0.03869018	0.463657011	0.545890001
UNC13D	-0.038681085	0.463762173	0.545981537
PLA2G2D	0.038666233	0.463933939	0.546151469
UNG	0.038647615	0.464149309	0.546372708
CRYAA	-0.03864101	0.464225737	0.546430377
KCNG4	0.038626222	0.46439685	0.546599484
C2orf83	0.038617661	0.464495935	0.546683798
SDC4	-0.038588847	0.464829492	0.547044045
FANCF	-0.038584043	0.464885124	0.547077187
ST7OT3	-0.038578835	0.464945432	0.547115829
E2F5	0.038535306	0.465449658	0.547658151
LRRC18	0.038534301	0.465461304	0.547658151

KLHL1	0.038530967	0.465499944	0.547671258
OR52A4	0.038506989	0.465777849	0.547965848
TUFT1	0.038499724	0.465862068	0.548032553
ZER1	0.038480388	0.46608626	0.548263902
NEUROG2	-0.038467166	0.466239592	0.548411876
CAPN3	-0.038455904	0.466370222	0.548533131
CSNK2A1	-0.038409237	0.466911732	0.549136341
FGB	0.038406954	0.466938228	0.549136341
AMPD2	-0.038393304	0.467096686	0.549290257
CFHR3	-0.038387963	0.467158701	0.549322569
BOK	0.038386186	0.46717933	0.549322569
PIWIL3	0.038369852	0.467369006	0.549513151
PPM1N	0.038365324	0.467421598	0.549542543
RNASE9	-0.0383521	0.4675752	0.549690679
CCDC125	-0.038295688	0.468230766	0.550403482
CDRT15	-0.038295169	0.468236796	0.550403482
KHDRBS2	-0.038290501	0.468291071	0.550434792
GCNT1	-0.038266225	0.468573353	0.550734084
CDK18	-0.038250041	0.468761602	0.550922827
CREB3L3	-0.038239971	0.46887875	0.55102799
FAM24A	-0.038230162	0.468992883	0.551102501
LCLAT1	-0.038229765	0.468997499	0.551102501
KCNH3	-0.038195977	0.469390742	0.551532045
TTTY8	-0.038164885	0.469752776	0.551904032
TAS2R30	-0.038162762	0.469777503	0.551904032
VGLL2	0.038161649	0.469790467	0.551904032
SLC6A5	-0.038155819	0.469858371	0.551951245
PNMA1	-0.038152933	0.46989199	0.55195818
OR1G1	-0.03814638	0.46996832	0.552015281
TMPRSS11A	-0.038132604	0.47012881	0.552171223
EML6	-0.038089432	0.470631973	0.552729594
FAM176A	0.038086072	0.470671152	0.552743011
NOP14	0.038076428	0.470783592	0.552842457
C12orf54	0.038072166	0.470833298	0.552868227
KRT35	-0.038046412	0.47113366	0.553183029
MRFAP1L1	-0.038044416	0.471156944	0.553183029
WT1	-0.038036078	0.471254225	0.553232599
ZPLD1	-0.038036035	0.471254723	0.553232599
ZFP42	0.038014381	0.471507395	0.553496597
MYCN	-0.037993738	0.471748333	0.553746791
SLN	0.037978185	0.471929907	0.553927276
PTPN23	0.037968971	0.472037497	0.554020907
ZNF43	-0.037958428	0.472160619	0.554132755
CXorf58	-0.037955342	0.472196663	0.554142401
HAMP	-0.037939548	0.472381152	0.554326241
APLN	0.037934692	0.472437881	0.554360146

MC4R	-0.037929752	0.472495591	0.554362887
MAGEA1	0.037929727	0.47249589	0.554362887
CHRNA1	0.037920686	0.472601521	0.554454155
KATNAL2	-0.037908669	0.472741953	0.554586239
XYLB	0.037870628	0.473186648	0.555044336
KRT6C	0.037869656	0.473198018	0.555044336
OR4D11	0.037868113	0.473216057	0.555044336
UBA1	-0.037832265	0.47363535	0.555464143
SLC39A11	-0.037832223	0.473635834	0.555464143
PABPN1L	-0.037830359	0.473657649	0.555464143
LOC643008	0.037821329	0.473763302	0.555499193
NAB2	-0.037820925	0.473768028	0.555499193
C9orf45	-0.037820652	0.473771218	0.555499193
ARMCX6	-0.037802568	0.473982844	0.555714608
HIF3A	0.037781116	0.474233947	0.55597628
KRT6A	-0.0377623	0.474454258	0.556201822
UPK1B	0.037755161	0.47453786	0.556267083
LUZP2	-0.037710191	0.475064669	0.556851847
KRT31	0.037677159	0.475451836	0.557272869
NISCH	0.037650619	0.475763037	0.557604228
SMR3B	-0.037647457	0.475800118	0.557604228
LCE6A	-0.037645887	0.475818541	0.557604228
WDR72	0.037622282	0.476095426	0.557895875
CNGB1	-0.037617203	0.476155022	0.557932882
ITIH3	0.037590149	0.476472506	0.558272046
TMEM132D	-0.037546562	0.476984244	0.558825879
PC	0.037545109	0.477001311	0.558825879
NLRP7	0.037530493	0.477172986	0.558994119
FABP12	0.037515708	0.477346676	0.559164699
RNF222	-0.037507636	0.477441521	0.559242906
CEBPZ	-0.037492627	0.4776179	0.559416602
VSTM1	-0.037484503	0.477713384	0.559495532
NKX2-2	0.037460795	0.477992083	0.559789023
MT1F	0.037423437	0.478431441	0.560270619
APCS	0.037405272	0.478645155	0.560487933
MTMR8	-0.03738326	0.478904189	0.560758286
BFAR	0.037374094	0.479012085	0.560851647
TMSB4Y	0.03737136	0.47904427	0.560856358
AICDA	-0.037360572	0.479171272	0.560972069
FBXO31	0.037354843	0.47923872	0.561018052
RBM46	0.037343727	0.479369614	0.561138298
MORN3	-0.03733349	0.479490179	0.561246439
TRY6	0.037322651	0.479617847	0.561362881
FAM47B	0.037310554	0.479760351	0.561485912
MTA1	-0.037308941	0.479779351	0.561485912
MYO19	-0.037302751	0.479852281	0.561538263

IFITM5	-0.037277204	0.480153341	0.561836578
RBMY1E	0.037276236	0.480164759	0.561836578
CD6	0.037272109	0.480213404	0.561836578
HPYR1	-0.037271545	0.480220047	0.561836578
HIST1H4F	0.037266692	0.48027725	0.561869632
LOC100233	-0.037264362	0.480304726	0.561869632
KPRP	0.037233478	0.480668876	0.562251677
PHF21B	0.037231176	0.480696021	0.562251677
PSD2	-0.037229481	0.480716008	0.562251677
TNK1	-0.037215972	0.480875349	0.562391888
LEKR1	-0.037212946	0.480911048	0.562391888
OR10H5	-0.037212136	0.480920605	0.562391888
C9orf170	-0.037200688	0.481055668	0.562516801
FXN	0.037171633	0.481398543	0.562884687
SNX7	-0.037155511	0.481588851	0.563074148
UGT2B11	0.037151713	0.481633688	0.563093513
TCTE1	0.03713752	0.481801263	0.563256364
IGDCC4	-0.037125967	0.481937705	0.5633828
VPS11	-0.037117142	0.482041938	0.563453165
HIST1H4K	0.03711608	0.482054483	0.563453165
FAM25A	-0.037108915	0.482139119	0.563513275
PKP3	0.037106935	0.482162501	0.563513275
ISYNA1	0.037104154	0.482195356	0.563518603
C6orf221	0.03709298	0.482327373	0.56363981
OTOS	0.037072494	0.482569456	0.563889616
CEACAM3	0.037061384	0.482700772	0.564009966
COPG	-0.03705587	0.482765947	0.564033261
DDX43	-0.037054906	0.482777352	0.564033261
DKFZp434J0	-0.037051987	0.482811858	0.564040485
43899	0.037037751	0.482980173	0.564182524
MID1IP1	0.037036911	0.4829901	0.564182524
LOC100132	-0.036995372	0.483481405	0.564711996
P2RY2	-0.036993793	0.483500087	0.564711996
EXOG	-0.036981084	0.48365046	0.564854498
TRAM1	0.036969906	0.483782731	0.564953256
B4GALNT4	0.036969144	0.483791757	0.564953256
XKRY2	0.036945963	0.484066137	0.565240523
C12orf49	0.036933359	0.484215362	0.565381622
CYP27B1	0.036927557	0.484284059	0.565428683
ACSF3	0.03690941	0.484498973	0.565646445
ADAM5P	0.036894273	0.484678281	0.565822614
KCNB2	0.036847423	0.485233458	0.566437533
C15orf26	-0.036842629	0.485290283	0.566470663
MAP1LC3B2	-0.036802329	0.485768159	0.566995246
PEX11G	-0.036786699	0.485953562	0.567178409
LOC284688	0.036778432	0.486051634	0.567259628

FAM74A3	-0.036774008	0.486104132	0.567287652
GAS8	0.036755038	0.486329242	0.567517102
CHRNA2	0.03672519	0.486683561	0.567897295
FAM131C	0.036716739	0.486783896	0.567981093
ALKBH5	-0.036669822	0.487341171	0.568584107
RBMY3AP	0.036668422	0.487357806	0.568584107
SHC1	0.036664484	0.4874046	0.56860539
BEST1	0.03664741	0.487607499	0.568808771
SDR9C7	-0.036626842	0.487851994	0.569060649
RHO	-0.036620134	0.487931743	0.569120338
OR2L1P	-0.036612803	0.4880189	0.569175175
FNDC8	0.036611372	0.488035917	0.569175175
OR5K3	-0.036608584	0.488069076	0.569180514
CELF4	0.036605029	0.488111345	0.569196477
ALDH1A1	0.03659274	0.488257492	0.569333565
OR12D2	0.036575192	0.488466224	0.569543609
MYOG	0.036551587	0.488747075	0.569837714
GGT6	-0.036546437	0.488808365	0.56987581
TIPRL	0.036532195	0.488977872	0.570040058
GNG11	-0.036519915	0.489124049	0.570177091
HAO1	-0.036511156	0.489228336	0.57026528
GOLGA8E	-0.036501335	0.48934527	0.570368199
ZNF679	-0.036496407	0.489403959	0.570403222
CXCL13	-0.036470028	0.48971814	0.570735999
FAM71A	0.036420924	0.490303294	0.571384522
KLHL30	-0.036397263	0.490585377	0.5716798
GABRA5	-0.036379548	0.49079663	0.57189251
C9orf153	0.036373427	0.490869643	0.571944123
ZNF384	-0.036369971	0.490910861	0.571958685
MED9	0.036336967	0.491304621	0.572383967
MIB2	0.036325751	0.491438475	0.572506419
ADRBK2	-0.036308402	0.491645559	0.572704263
OBSL1	-0.036306705	0.491665819	0.572704263
C14orf50	-0.036283167	0.491946856	0.572998107
C9orf66	-0.036271639	0.492084536	0.57312495
SLIT1	-0.036258623	0.492239998	0.573272489
NETO2	-0.036199285	0.492949092	0.574064743
MMEL1	-0.036193762	0.493015124	0.574108069
ZNF283	0.036184581	0.493124883	0.574202306
NUDT15	0.036179927	0.493180534	0.574233532
E2F7	-0.036174417	0.49324643	0.574276683
CEACAM8	-0.036163244	0.493380052	0.574398675
MAP4	-0.036149495	0.493544498	0.574539626
PRDM12	0.036148298	0.493558821	0.574539626
SOX18	0.036123075	0.493860601	0.574855143
DACT2	-0.036120365	0.493893028	0.574855143

HYAL1	-0.036118407	0.493916462	0.574855143
MTF2	-0.036113781	0.493971825	0.57488598
TTC12	-0.036106791	0.494055485	0.574949745
CXCR2P1	-0.036096555	0.494178003	0.57505872
C1QTNF9	0.036077451	0.494406719	0.575291253
GGT8P	-0.036052929	0.49470037	0.575597332
VIT	-0.036050658	0.494727569	0.575597332
LANCL2	0.036030125	0.494973541	0.57584987
ARV1	-0.036019223	0.495104168	0.575968193
RIMS3	0.035992344	0.495426302	0.576309276
C3orf43	-0.035974015	0.495646021	0.57653119
SPAM1	0.0359582	0.495835659	0.576673512
SLC18A1	0.035957626	0.495842542	0.576673512
PLA2G3	0.035956566	0.495855246	0.576673512
TRDN	-0.035939027	0.496065608	0.576884472
MSLN	0.035916597	0.49633468	0.577163678
FAM196A	-0.035900589	0.496526774	0.577353343
OR52N2	0.035896121	0.496580391	0.577381976
S1PR4	0.035879973	0.49677421	0.577543596
AP1G2	0.035879708	0.496777394	0.577543596
RDH8	0.035828685	0.497390063	0.578222118
RIMBP3	-0.035822579	0.497463421	0.578273641
SHBG	0.035814398	0.497561701	0.578354128
POTEG	0.035803415	0.497693653	0.578473742
LOC728643	-0.035798423	0.497753634	0.578480386
PRSS27	-0.035798105	0.497757464	0.578480386
COMMD2	0.035768519	0.498113043	0.57885985
GLP1R	0.035756113	0.49826219	0.578999389
ARMC9	0.03571525	0.498753614	0.579536625
OR52B2	-0.035693166	0.499019307	0.579811523
AMHR2	-0.035682738	0.499144787	0.579906809
LRRTM4	0.035681511	0.499159554	0.579906809
CECR6	0.035643585	0.499616082	0.580403329
SRGAP3	-0.035620921	0.499889001	0.580686506
SNORA5B	0.035587457	0.500292115	0.581120879
ABCD4	0.035560268	0.500619771	0.581467557
CCNY	-0.035513628	0.501182084	0.582086732
GATA5	-0.035490784	0.501457624	0.582372789
ESRRA	0.035438694	0.502086225	0.583068819
GADD45G	-0.035429963	0.502191633	0.583157223
DCDC1	-0.035415141	0.502370589	0.583331018
MCOLN1	0.035407898	0.502458048	0.583346291
KRT36	0.035407438	0.50246361	0.583346291
ERAP2	0.035406775	0.502471618	0.583346291
TYRO3	0.035402506	0.502523173	0.583372136
GPSM1	0.035376961	0.502831735	0.583696317

PDLIM4	-0.035371242	0.502900824	0.583742491
FBXO4	0.035359721	0.503040031	0.583870044
S100B	0.035356129	0.503083432	0.583870955
MACROD2	-0.035354804	0.503099452	0.583870955
FUT2	-0.035331129	0.503385583	0.584168981
SLC34A1	0.035312375	0.503612319	0.58439805
TRIML1	-0.035302113	0.503736395	0.584473944
OR52E8	-0.035299724	0.503765286	0.584473944
KIF5A	-0.035299684	0.503765767	0.584473944
MAP2K7	0.035292228	0.503855945	0.584544515
GPR158	0.035284909	0.503944461	0.584613149
GP9	-0.035260607	0.504238434	0.584920108
PDE6H	-0.035250148	0.504364986	0.585032833
HIST1H3A	0.035236644	0.504528404	0.585188303
TMEM184A	-0.035229191	0.504618616	0.585258852
C6orf168	-0.035225886	0.504658618	0.585271161
NCDN	-0.035205018	0.50491123	0.585530027
TFF1	0.035185489	0.505147711	0.585765294
RNF151	0.035183406	0.505172931	0.585765294
C10orf111	0.035170746	0.505326261	0.585908971
UBA2	0.035155624	0.505509446	0.586087245
CALM1	0.035152737	0.505544419	0.586093673
PPIC	0.035120314	0.505937315	0.586515026
SEMA6B	-0.035071693	0.506526791	0.587164205
CD74	0.035066294	0.506592261	0.587190637
CT47A11	0.03506495	0.506608563	0.587190637
RXFP2	-0.035060866	0.506658101	0.587213878
TPMT	-0.035058433	0.506687616	0.587213912
TJP3	-0.035035371	0.506967379	0.587503948
CYP11B2	-0.035031085	0.507019388	0.587530031
SCAP	0.035016487	0.507196527	0.587701102
COLEC12	0.035005556	0.507329197	0.587820628
RGNEF	-0.034996008	0.507445096	0.587907818
KIR3DL3	-0.034994493	0.507463489	0.587907818
SNTG2	-0.034977972	0.507664058	0.588105969
GPX6	0.034963208	0.50784334	0.588254485
C1orf130	0.034962549	0.507851336	0.588254485
FOXD2	0.034955914	0.507931921	0.58831361
RBMY1B	0.034938233	0.508146681	0.588528128
ACSM1	0.034930564	0.508239845	0.588571198
T	0.034930306	0.508242977	0.588571198
PRSS55	0.034921033	0.508355651	0.588667449
CA4	-0.034903445	0.508569366	0.588880686
TFAP2C	-0.034893401	0.508691441	0.588987794
REPIN1	0.034889542	0.508738352	0.589007865
GNG3	-0.034867634	0.509004682	0.589281958

LRG1	0.034860013	0.509097343	0.589354972
SLC12A3	0.034854238	0.509167577	0.589402017
LIMS3-LOC	0.034829639	0.509466752	0.589714058
ADAM32	-0.034809493	0.509711845	0.589963466
GXYLT2	-0.034801605	0.509807829	0.59004027
ELAVL3	-0.034798768	0.509842353	0.590045935
SLC10A3	0.03478877	0.509964028	0.590152456
GLIPR1L1	-0.034782381	0.510041789	0.590208148
NXPH1	0.034773697	0.510147482	0.590296154
FAM73B	0.034762747	0.510280784	0.590416094
ERGIC2	0.034753586	0.510392325	0.590510844
YRDC	-0.034737303	0.510590596	0.59069897
C10orf95	-0.034735361	0.510614249	0.59069897
ZAN	0.034722885	0.510766201	0.590840433
F12	-0.034687893	0.511192513	0.591299233
SNORA55	0.03460439	0.512210576	0.592442422
OR51M1	0.034597775	0.512291279	0.592501354
ZNF542	0.034593963	0.512337786	0.592510581
KRTAP5-3	-0.034589855	0.512387896	0.592510581
QSOX2	-0.034589805	0.512388513	0.592510581
PNLIP	-0.03457598	0.512557198	0.592671231
ZNF792	-0.034569446	0.512636934	0.592729014
FLJ40330	-0.034552167	0.512847824	0.592938428
FLJ35024	0.034519539	0.513246162	0.593323445
UBE2E2	0.034518363	0.51326052	0.593323445
DHDPSL	0.034517569	0.513270214	0.593323445
NEK5	-0.03449446	0.513552458	0.593615254
KLRB1	0.034481733	0.513707923	0.593760493
FERMT3	0.034474671	0.513794201	0.59382575
TREX1	-0.034468669	0.513867541	0.59387605
CDC20B	-0.034458616	0.513990386	0.593983551
HKDC1	0.034431767	0.514318537	0.594328285
C10orf46	-0.03439269	0.514796347	0.594837747
SYCP1	0.034390825	0.514819152	0.594837747
CISD2	0.034384312	0.514898814	0.594869073
HLA-DRB6	0.034383724	0.514906004	0.594869073
PTH	0.03434388	0.515393472	0.595397703
PRLR	0.034334684	0.515506019	0.595493177
KRT13	0.034322068	0.515660444	0.595635168
NELL2	-0.034319755	0.515688755	0.595635168
ATP6V1G2	-0.034287923	0.516078491	0.596050754
IGFBPL1	0.034245023	0.516603974	0.596623066
CPO	-0.034216492	0.516953611	0.596992239
MIR155HG	0.034197326	0.517188546	0.597228917
IGFL4	-0.034182094	0.517375306	0.597409939
OTOR	-0.034144197	0.517840103	0.597911969

CCDC129	-0.034097618	0.518411669	0.598537213
FGL1	0.034085149	0.518564731	0.598679225
SAFB2	0.034063374	0.518832082	0.598944023
MT1B	-0.034061569	0.518854245	0.598944023
STAT4	-0.034044883	0.519059173	0.599145855
ACSL3	-0.034020289	0.519361279	0.599451094
AGMAT	0.034018455	0.519383812	0.599451094
UBAP2L	0.033995747	0.519662849	0.599738388
SNORA51	-0.033987219	0.51976767	0.599824601
MLLT1	-0.033974661	0.519922029	0.599967969
ZNF322A	0.033971331	0.519962962	0.599980438
ASB13	-0.033954322	0.520172086	0.60018697
HCG26	-0.033941128	0.520334325	0.600339383
C2orf73	-0.033930736	0.52046213	0.600452051
LANCL3	-0.033917811	0.520621117	0.60060068
RESP18	0.033903854	0.520792818	0.600763958
LOC154822	-0.033900044	0.5208397	0.600783239
NCRNA0020	-0.03387046	0.521203759	0.601168358
ZNF382	-0.033848878	0.52146943	0.601439955
SLAMF8	0.033829368	0.521709668	0.601682189
FLJ16779	0.033821851	0.52180224	0.601754104
NXPH2	-0.033805242	0.522006801	0.60195515
NEFM	0.033801217	0.522056381	0.601977466
PRKAG3	0.033773568	0.522397048	0.602335411
LOC646851	-0.03375259	0.522655577	0.602580262
TMBIM1	-0.033751427	0.52266992	0.602580262
KIAA1967	-0.033746693	0.522728275	0.602612654
TIMELESS	0.03368975	0.523430432	0.603387187
NUP133	0.033687287	0.523460821	0.603387293
HCN1	-0.033672582	0.523642226	0.603540268
CEP63	-0.033671616	0.523654145	0.603540268
C10orf113	0.03364652	0.523963823	0.603822764
MRGPRX3	-0.033645887	0.523971645	0.603822764
ANGPTL3	-0.033644383	0.523990208	0.603822764
MKRN3	0.033638967	0.524057046	0.603864842
CXCR6	-0.033616778	0.524330964	0.604144142
LIG3	0.033614417	0.524360105	0.604144142
STK24	0.033607243	0.524448687	0.604211246
MAX	0.033601216	0.52452311	0.604262032
FAM71C	-0.033584504	0.524729493	0.604431492
SLC30A10	-0.033584389	0.52473091	0.604431492
KRT38	0.033576439	0.524829107	0.60450964
ZNF816A	0.033567345	0.52494145	0.604604071
LYPD1	0.033533723	0.525356871	0.605047542
SPINK6	0.033495404	0.525830544	0.605550007
PAR1	-0.033493509	0.525853971	0.605550007

GLUD1	0.03348831	0.525918256	0.605589018
RIPPLY1	0.033485574	0.525952095	0.605592968
GGNBP1	0.033462001	0.526243627	0.605893614
IL21R	-0.033459381	0.526276036	0.605895899
CCDC40	-0.033420447	0.526757735	0.606415417
HTT	0.033413965	0.526837955	0.606472709
TGFB1	-0.033407333	0.526920038	0.60653214
ADH1A	-0.033404032	0.526960892	0.606544108
FZR1	0.033396698	0.527051671	0.606581829
OR8D2	-0.033396463	0.527054581	0.606581829
VSTM2A	-0.033392307	0.527106026	0.606605982
RNF139	0.03338251	0.527227318	0.606680633
SLC35A2	-0.033380518	0.52725198	0.606680633
LHX5	-0.033379686	0.527262284	0.606680633
SNX31	-0.033376659	0.527299762	0.606688703
PLA2G2C	0.033372664	0.527349229	0.606710566
C6orf118	0.033340935	0.527742169	0.607127566
LIPK	0.033329883	0.527879083	0.607249996
BBS7	-0.033305239	0.528184436	0.607566166
LDHA	0.033291846	0.528350418	0.607721992
RLTPR	0.03326722	0.528655674	0.608025097
HPCA	0.033265661	0.528674997	0.608025097
SLC35D3	0.033254355	0.528815186	0.608151206
LCE3C	0.033240506	0.528986919	0.608313575
ZFY	-0.033188628	0.529630492	0.609018491
FAM19A4	0.033164057	0.529935455	0.609333983
TOX2	-0.033127779	0.53038587	0.609816674
MYO9B	-0.033120242	0.53047947	0.609889081
C17orf78	0.033115233	0.530541676	0.609925387
FBXW12	0.033111518	0.530587817	0.609943222
PROKR2	-0.033100329	0.530726801	0.610067777
NBL1	0.033088555	0.530873079	0.610200702
MEOX2	-0.033049205	0.531362077	0.610676048
SOHLH1	0.033048586	0.531369771	0.610676048
IL4	0.033047874	0.531378621	0.610676048
HOPX	-0.033041875	0.531453186	0.610726497
SMPD1	-0.03303215	0.53157409	0.610830188
BRD8	0.03302907	0.531612374	0.610838935
ILDR1	-0.033025894	0.53165186	0.610849062
TLE6	0.032937673	0.532749306	0.612074675
TMEM31	0.032924464	0.532913721	0.612228252
RRN3P2	-0.032919304	0.532977966	0.612231727
PLCB3	-0.032918132	0.532992552	0.612231727
XRCC2	-0.032916039	0.533018612	0.612231727
SPANXN5	0.032914344	0.533039715	0.612231727
PFKL	0.032908971	0.533106597	0.612273234

CECR5	0.032890908	0.533331517	0.612496232
OR51B6	-0.032883534	0.533423353	0.612566374
IL20RA	-0.032875316	0.533525706	0.612648587
IL10RB	0.032861828	0.53369371	0.612806171
TTC38	-0.032836879	0.53400456	0.61312775
IDUA	-0.032821038	0.534201965	0.613319044
OR52M1	0.032815086	0.534276158	0.613368864
HTR3A	0.032804335	0.534410164	0.613487342
C1orf74	-0.032786281	0.534635246	0.613710353
TYRP1	-0.032774279	0.534784898	0.613846757
MRPL3	-0.032757567	0.534993322	0.614036363
APIP	0.03275609	0.535011748	0.614036363
LOC541473	-0.032750937	0.535076017	0.614074735
GSX2	-0.032739294	0.535221254	0.61420602
EMX1	-0.032734742	0.535278041	0.614229081
SRRM3	0.032732739	0.535303034	0.614229081
OR3A2	-0.032724756	0.535402641	0.614307979
NOTUM	0.032713199	0.535546845	0.614418424
C18orf20	-0.032712097	0.535560603	0.614418424
OR2T2	0.032676917	0.535999709	0.614872253
COL21A1	-0.032675457	0.536017935	0.614872253
IFI27	0.032667927	0.536111946	0.614944672
DDX23	0.032664673	0.536152569	0.61495585
CLSTN1	-0.032607668	0.536864585	0.615737054
NHLH2	0.032599854	0.536962213	0.615755193
SIGLEC1	-0.032599569	0.536965784	0.615755193
SLC37A4	-0.032598978	0.536973158	0.615755193
C3orf77	0.032568006	0.537360251	0.616163599
HIST1H2AD	0.032533807	0.537787831	0.61661838
GABRQ	-0.032508205	0.538108026	0.616949991
PI15	-0.032486306	0.538381996	0.617228568
COL4A6	-0.03247406	0.538535231	0.617368704
C9orf70	0.032459007	0.538723613	0.617549114
MUC13	0.03244072	0.538952525	0.61777596
NKX2-3	-0.032404676	0.539403837	0.618257692
CACNG7	0.032390743	0.539578354	0.618405083
CBLN1	-0.032389451	0.539594534	0.618405083
LOC729156	0.032368166	0.539861186	0.618675078
AGPAT9	-0.032346263	0.540135643	0.618953986
LOC283404	0.032291999	0.540815921	0.619663486
ASTN2	-0.032291911	0.540817025	0.619663486
SPDYE2	-0.032284331	0.540912082	0.619736745
CAPN6	-0.032274537	0.541034913	0.619841816
MPP4	0.032256437	0.541261967	0.620066272
CCDC74B	-0.032241182	0.541453356	0.620249846
MYH1	-0.032229652	0.54159804	0.620379902

FGF11	0.032191383	0.542078401	0.620894425
OPN5	-0.032183826	0.542173278	0.620951228
TBC1D21	0.03218174	0.542199464	0.620951228
IRX3	-0.032179983	0.542221535	0.620951228
HMSD	0.032161385	0.542455073	0.621182956
FAAH	-0.032151686	0.542576899	0.621286738
CCR6	-0.032144998	0.542660902	0.621347201
C15orf43	-0.032138852	0.542738112	0.62139988
TAAR6	-0.032119839	0.542976986	0.621577443
RPAP1	-0.032119308	0.542983654	0.621577443
C1orf110	-0.032119055	0.542986833	0.621577443
SALL3	0.032110617	0.543092863	0.621663086
MGC2889	0.032083693	0.543431252	0.622010285
BEYLA	-0.032081187	0.543462752	0.622010285
SLC15A1	0.032079029	0.54348988	0.622010285
GSTK1	0.032074994	0.543540612	0.6220326
ATP6AP1L	-0.032038335	0.544001564	0.622524343
CARD10	0.032035462	0.544037702	0.622529926
NOTO	0.032025513	0.544162838	0.622637341
GPC5	-0.032002844	0.544448025	0.62290661
PGCP	0.032001834	0.544460726	0.62290661
RGL4	0.031997471	0.544515631	0.622908693
LRFN5	-0.031996718	0.544525103	0.622908693
SFXN5	-0.031982432	0.544704879	0.623078557
PPME1	0.031975498	0.544792143	0.623142584
IFNA14	0.031971882	0.544837656	0.623158851
ZNF497	0.031938233	0.545261251	0.623607524
OR51B5	0.031921226	0.545475414	0.623816634
BTG4	-0.031907321	0.545650548	0.623981088
MEAF6	-0.031895828	0.54579532	0.624110806
MAPK11	-0.031883909	0.545945468	0.624246655
OR51I2	-0.031876374	0.546040411	0.624308
ALDH18A1	0.031874164	0.546068259	0.624308
CLDN6	0.031872187	0.546093164	0.624308
CSDA	0.031861324	0.546230063	0.624428661
SERINC2	0.031858008	0.546271852	0.624440588
LOC84740	-0.031822041	0.546725224	0.624922966
C3orf79	-0.03180985	0.546878936	0.625062787
PRAC	0.031779066	0.54726719	0.625451984
FXR2	-0.031777871	0.547282263	0.625451984
COL22A1	0.031774728	0.547321901	0.62546139
DYSFIP1	0.031767116	0.547417936	0.625486559
HP	0.031764745	0.54744785	0.625486559
DEGS1	-0.03176303	0.547469479	0.625486559
TBX22	-0.031763024	0.547469556	0.625486559
PVR	0.031758253	0.547529746	0.625519441

RHCE	-0.031753243	0.547592961	0.625555774
AOX2P	-0.031708228	0.548161104	0.626168888
C18orf18	-0.031654497	0.548839617	0.626907999
KCNJ10	0.031647222	0.548931512	0.626977006
FADS6	0.03162906	0.549160976	0.627203122
GCKR	-0.031619911	0.549276588	0.627299188
HSD17B2	0.031591367	0.549637353	0.627675204
HSPC159	-0.031583473	0.549737147	0.627730608
SNORD89	-0.031582542	0.54974891	0.627730608
HTR4	-0.031572034	0.549881764	0.627846309
HRC	0.031556345	0.550080156	0.628036823
CALB2	0.031551169	0.550145612	0.628074355
FITM1	0.031548758	0.550176104	0.628074355
KLHDC8A	0.031544366	0.55023165	0.62810176
SPATA4	-0.031539996	0.550286921	0.628128849
IGLL1	0.031502701	0.550758741	0.628614118
KRT28	-0.031501401	0.550775181	0.628614118
EPHB2	0.031487998	0.550944798	0.628771671
TPCN1	-0.031472887	0.551136056	0.628953903
DCAF12L2	0.031460333	0.551294968	0.629099203
CSN2	0.031452193	0.551398028	0.629180756
C4orf40	0.031440271	0.55154897	0.629316932
HPD	-0.031437664	0.551581981	0.629317691
SLCO4A1	-0.031435228	0.551612836	0.629317691
IGSF21	0.031428159	0.551702356	0.629383767
MRPL44	0.031407046	0.551969765	0.629652759
CYorf15B	0.031341937	0.552794805	0.630557794
MLPH	-0.031335836	0.552872153	0.630609905
LOC401431	-0.031324557	0.553015151	0.630720701
NYNRIN	-0.031323178	0.553032632	0.630720701
FAM81A	-0.031301178	0.553311609	0.630973144
ADAM23	-0.031300725	0.553317348	0.630973144
PLEKHA4	0.031292204	0.553425428	0.631036748
LOC150527	-0.031291331	0.553436497	0.631036748
KCNH2	0.031272781	0.553671801	0.631236647
LOC154449	-0.031270046	0.553706495	0.631236647
KCNA1	0.031270014	0.553706903	0.631236647
DBF4B	0.031260921	0.553822272	0.631332029
SLC26A7	-0.031235003	0.554151162	0.631670791
ST8SIA3	-0.031226763	0.554255751	0.631753851
TTTY5	0.031216391	0.554387403	0.631847822
KCNJ15	0.031213945	0.554418449	0.631847822
AXIN2	-0.031212769	0.554433377	0.631847822
HVCN1	-0.031159814	0.555105836	0.632545112
CRNN	0.031159583	0.555108759	0.632545112
C12orf40	-0.031145552	0.555287004	0.632682307

MYL10	0.031145104	0.555292697	0.632682307
ZAR1	0.031127973	0.555510353	0.632894089
ZNF389	0.031104439	0.555809435	0.63319861
ZNF642	0.031055396	0.556432944	0.633872673
FAM110B	0.031015294	0.556943026	0.634414531
KIR3DL2	0.031012992	0.556972315	0.634414531
C12orf60	-0.030999765	0.557140621	0.634569944
SPESP1	0.030956459	0.557691807	0.635161405
SULT4A1	-0.030915509	0.558213262	0.63571894
GOLT1B	-0.030899717	0.558414417	0.63591166
RIMS4	-0.030894195	0.558484764	0.635923875
TMEM194B	0.030892031	0.558512338	0.635923875
RAD21	-0.030889558	0.558543838	0.635923875
MOSC1	-0.030888849	0.558552871	0.635923875
GTF2E2	0.0308703	0.558789213	0.636153689
SNORA59B	-0.030865662	0.558848322	0.636153689
FAM200A	0.030865487	0.558850554	0.636153689
STAC	0.030851706	0.559026193	0.636317252
C11orf45	-0.030834132	0.5592502	0.636535848
TMEM35	-0.030831349	0.559285675	0.636539846
FAM81B	0.030820112	0.559428943	0.636666518
GRIN2B	-0.030817149	0.559466727	0.636673135
GSTT2	-0.030790759	0.559803255	0.637019703
TBL1Y	-0.030787161	0.559849151	0.63703553
NLRP10	-0.030782237	0.559911957	0.637070594
SNORA11	0.030772336	0.560038251	0.637177889
HR	-0.030763303	0.560153495	0.637272599
IRGC	0.030721298	0.560689521	0.637845985
HMGXB3	-0.030703797	0.560912927	0.638063685
RNF215	0.030690386	0.561084146	0.638222
TFRC	-0.030683366	0.561173782	0.638287502
EPGN	-0.030651426	0.561581703	0.638714999
NHEDC1	-0.030643978	0.561676845	0.638760312
CD300LB	-0.030643285	0.561685693	0.638760312
P4HTM	-0.030625136	0.561917573	0.638953739
GPT	0.03062495	0.561919949	0.638953739
ADAP1	-0.030607029	0.562148956	0.639143911
CEACAM21	0.030606839	0.562151381	0.639143911
IFNA1	0.030593026	0.56232793	0.639308142
ASZ1	0.030589885	0.562368071	0.639317282
LCE1C	0.030581357	0.562477086	0.639404713
CGA	0.030549363	0.562886165	0.639833217
DENND4B	-0.030518309	0.563283345	0.640248149
WFDC10B	-0.03051567	0.563317101	0.640249975
ZNF569	0.03049197	0.563620325	0.640558053
PCLO	-0.030489404	0.563653166	0.640558822

NSMAF	-0.030484228	0.563719405	0.640597544
ASIP	-0.03048134	0.563756357	0.640602981
CTNNAL1	0.030450256	0.564154228	0.640984251
CTPS2	0.030450097	0.564156262	0.640984251
AGBL1	-0.030437096	0.564322705	0.641136783
ZNF98	-0.030411061	0.564656097	0.64147896
TRPC3	-0.03040776	0.564698381	0.641490403
C15orf55	-0.03040368	0.564750635	0.64151317
POU2F2	-0.030392072	0.564899321	0.641645467
NPR2	-0.030374875	0.565119637	0.641859107
TUBBP5	0.030371162	0.565167204	0.641876525
LOC347376	0.03036798	0.565207985	0.641886235
PSAP	0.030352462	0.565406835	0.642075447
SBSN	-0.030338392	0.565587156	0.642243597
LOC284009	-0.030325157	0.565756815	0.642399622
C1orf210	-0.030291455	0.566188929	0.642853622
SLC43A3	-0.030253181	0.566679841	0.643374324
C9orf57	-0.030246622	0.566764003	0.643433196
KBTBD5	0.030240006	0.56684888	0.643487661
CDK11B	0.030237846	0.566876602	0.643487661
KRTAP4-7	0.030233526	0.566932037	0.643492988
DGAT2L6	-0.030232444	0.566945919	0.643492988
THBS4	-0.030219498	0.567112058	0.643644876
KIF2B	0.03020913	0.567245116	0.643753028
CD58	0.030207036	0.567272001	0.643753028
SOX14	0.030203207	0.567321143	0.643772112
CHAT	0.030177391	0.567652571	0.644111501
PPY2	-0.03017019	0.56774504	0.644177239
C1orf228	0.030167841	0.567775198	0.644177239
LOC200726	0.030136953	0.568171896	0.644578157
PSG11	-0.030135287	0.568193299	0.644578157
KRTCAP3	-0.030129212	0.56827134	0.644629969
CT47A6	0.030122129	0.568362337	0.644696471
LOC146336	0.030118718	0.56840616	0.644709458
LY6G6F	0.030112166	0.568490334	0.644735038
LOC100134	-0.030111923	0.568493461	0.644735038
TUBA1A	0.030090223	0.568772311	0.645014553
ARHGEF5	-0.030055704	0.569216023	0.645444185
SLC5A2	0.030053441	0.569245116	0.645444185
SAA4	0.030051263	0.569273119	0.645444185
CEACAM20	0.030050666	0.569280799	0.645444185
LOC340094	0.03004227	0.569388754	0.645529832
CEACAM16	0.030017771	0.569703814	0.645836737
OLIG2	0.030016177	0.569724318	0.645836737
SPOCD1	0.030006862	0.569844132	0.64593579
ATP1B1	-0.030001213	0.569916794	0.645981386

PP14571	0.029992129	0.570033661	0.646053212
KRT72	-0.029991244	0.570045043	0.646053212
RPAP3	-0.029977732	0.570218896	0.64621347
NCRNA0017	0.029968182	0.570341789	0.646315962
CMTM2	0.029915636	0.571018166	0.647045621
ADORA3	-0.029878697	0.571493881	0.647514288
IFNA5	-0.029878471	0.571496794	0.647514288
FKBP10	0.029871847	0.571582123	0.647548455
APOF	0.029871082	0.571591981	0.647548455
TBC1D3	-0.029863984	0.57168342	0.647615205
C4orf22	-0.029856095	0.571785047	0.647693488
COX6B2	0.029843458	0.571947883	0.647841093
SULT2A1	-0.029805206	0.57244089	0.648362642
GTF3C2	-0.029791452	0.572618204	0.648526588
EIF4E1B	-0.029788762	0.572652888	0.648528988
ZNF512	-0.029780202	0.57276326	0.6486171
TRIM10	-0.029776385	0.572812475	0.64863595
C4orf23	0.029769011	0.572907573	0.64870675
TEF	-0.029758786	0.573039432	0.648819165
TRAPPC2	-0.029742537	0.573249022	0.649019573
FAM173B	-0.029728063	0.573435745	0.649170082
PRTN3	-0.029727179	0.573447154	0.649170082
ARHGAP22	0.02969222	0.573898276	0.649643845
C1orf92	0.029676166	0.574105489	0.649841469
PPM1E	-0.029671173	0.574169948	0.649877494
SV2A	0.029667748	0.574214162	0.649890602
LEO1	0.029664165	0.574260421	0.649906023
KRTAP12-1	0.029652817	0.574406939	0.650034901
NPVF	-0.029640691	0.574563513	0.650139197
NTF4	-0.029640623	0.574564392	0.650139197
OR11A1	0.029626358	0.574748617	0.650310705
MAP1LC3A	0.029621302	0.574813917	0.65034764
HOXB5	0.029595981	0.575141019	0.650680759
KLK7	-0.029553647	0.575688084	0.651262678
HBG1	-0.02954371	0.575816522	0.651370976
OR51I1	-0.029528043	0.576019071	0.651563092
CNN2	0.029523633	0.576076092	0.651569397
C18orf26	0.029521311	0.576106109	0.651569397
C17orf105	-0.02952002	0.576122797	0.651569397
BRSK2	0.029514573	0.57619324	0.651584542
PTPN2	0.029513924	0.576201625	0.651584542
TRIM6	-0.029511053	0.57623875	0.651589524
LHX8	0.029503443	0.576337157	0.651663799
PLCXD1	0.029498642	0.576399251	0.651697008
TRPC7	-0.029486384	0.576557789	0.651834974
HOXA7	-0.029484146	0.576586739	0.651834974

HLA-G	0.02946149	0.57687983	0.652129296
CYP2D7P1	0.029456071	0.576949946	0.652171539
CACNG3	0.029452995	0.576989748	0.652179513
SNORA5C	-0.02944121	0.57714224	0.652314853
HFE2	0.029426972	0.577326515	0.652486099
FLJ10661	0.029408202	0.577569471	0.652722585
CHST5	-0.029405742	0.577601312	0.652722585
MTHFD2L	-0.029392193	0.577776726	0.652883766
LOC144776	0.029370546	0.578057035	0.653163452
CCL19	-0.029357978	0.578219804	0.653310303
SPTBN4	-0.029329949	0.578582897	0.653683463
MAP7D1	-0.029319188	0.578722324	0.653803896
CDS2	-0.029295217	0.579032964	0.65411773
TMEM117	0.029269969	0.579360231	0.654450312
ADAT2	0.02924963	0.579623937	0.65471106
GAPDHS	0.029245834	0.579673158	0.654729522
MLNR	-0.029231519	0.579858796	0.654902054
HTN1	0.029221599	0.579987468	0.655004585
DNMT3B	0.029217845	0.580036153	0.655004585
NEUROG3	0.029216913	0.580048248	0.655004585
ARR3	0.029169015	0.580669697	0.655669162
TMEM45B	0.029156207	0.580835933	0.655819685
KRT16	-0.029148476	0.580936273	0.655895792
PLEKHG2	-0.029142973	0.581007712	0.655939262
IGFBP3	-0.029131015	0.581162945	0.656058477
CLEC3A	-0.029128564	0.581194776	0.656058477
MBD3L2	-0.029127226	0.581212137	0.656058477
OPN1MW	-0.029117454	0.581339029	0.656164518
MT1L	0.029097037	0.581604156	0.656426567
ABCC12	0.029008266	0.582757608	0.657678336
VMA21	0.029006597	0.582779292	0.657678336
LCP1	-0.029003064	0.582825231	0.65769291
OR10W1	0.028957712	0.583414947	0.658321076
REG1P	0.028930771	0.583765404	0.658642508
OR1I1	0.028930728	0.583765951	0.658642508
SRY	0.028917705	0.583935396	0.658796363
LIMK1	-0.02891261	0.584001686	0.658815057
STARD7	0.028911347	0.584018128	0.658815057
PSAT1	0.028875096	0.584489924	0.65928435
MASP2	0.028874295	0.584500351	0.65928435
ZNF839	-0.02886876	0.584572412	0.659328289
AQP7	-0.028846148	0.584866807	0.659622974
PSEN2	0.028821853	0.585183202	0.659942435
ZSCAN5B	-0.028816045	0.585258851	0.659990375
TPST2	-0.028794942	0.585533748	0.660262986
GLRA2	0.028779163	0.585739347	0.660457428

GPR111	0.028767255	0.585894514	0.660594987
ACAP1	0.028764116	0.585935429	0.660603717
SIRPD	-0.02875859	0.586007437	0.660634508
GLRA4	-0.028756929	0.586029085	0.660634508
SPANXN4	-0.028735803	0.586304451	0.66090752
DECR1	0.028713638	0.586593429	0.661195842
SLC2A6	-0.028708383	0.586661952	0.661235655
IPO5	0.028704421	0.586713625	0.661256471
ATOH7	0.028693417	0.586857121	0.661354274
ECT2L	0.028692673	0.58686682	0.661354274
SERP2	-0.028681599	0.587011257	0.661479612
SPTBN5	-0.02863506	0.587618418	0.662126333
POC5	0.028602339	0.588045492	0.66257007
INTS5	0.028598594	0.588094379	0.662587665
CDHR4	0.028582641	0.588302649	0.662784819
NOX4	0.028576851	0.588378255	0.662832501
PNLIPRP3	-0.028542272	0.588829833	0.663303701
ZNF767	-0.028528302	0.589012318	0.663471737
ERP27	0.028501899	0.58935729	0.663822772
C6orf191	0.028477116	0.589681177	0.664150019
C19orf57	-0.02846904	0.589786733	0.66423134
KRT20	0.02846507	0.58983864	0.664252235
OR10A7	0.028435587	0.590224098	0.664648737
KIAA1377	-0.028425953	0.590350077	0.664753013
ZNF479	0.02841878	0.590443885	0.664821053
HCN2	-0.028415701	0.590484161	0.664828814
PRSS41	0.028408608	0.590576921	0.664895663
ABCC8	-0.028391718	0.590797854	0.665093366
FOLR3	-0.028390078	0.590819319	0.665093366
OR51Q1	0.028373944	0.591030398	0.6652597
METAP2	-0.028373677	0.591033888	0.6652597
TAAR8	0.028369842	0.591084076	0.66527859
ADAM2	0.028364363	0.59115577	0.665321681
SERPINB10	0.028351006	0.591330566	0.665480799
TCTE3	0.028346123	0.591394463	0.6655151
SNORA2A	-0.028334193	0.591550613	0.665653207
BARHL1	-0.028319299	0.591745579	0.665834975
ZNF584	-0.028310631	0.591859065	0.665889597
GGNBP2	-0.028310484	0.591860997	0.665889597
PATE2	-0.028291379	0.592111151	0.666133408
CLDN8	-0.028286985	0.592168698	0.666160516
KIAA1539	0.028283715	0.59221152	0.666171059
C13orf38	-0.028278139	0.592284547	0.666204566
CLU	0.028276332	0.592308212	0.666204566
APBA3	-0.028263445	0.592476997	0.666329391
C7orf34	0.02826275	0.592486109	0.666329391

TMEM86A	-0.02825898	0.592535493	0.666347301
SORCS1	0.028244473	0.592725545	0.666523389
KLKP1	-0.028239932	0.592785027	0.666552641
AQP10	0.028234853	0.592851581	0.666567504
TMCC1	0.028232709	0.592879671	0.666567504
PPP1R14A	-0.02823126	0.592898657	0.666567504
GIN1	-0.028199421	0.59331592	0.666978264
SPEF2	-0.02819827	0.593331003	0.666978264
RPAP2	-0.028195052	0.59337319	0.666988038
MGAM	-0.028136346	0.594142919	0.667815568
B3GNT8	0.028121533	0.594337222	0.667973383
LRRC37B	-0.028120528	0.594350407	0.667973383
OR2S2	-0.028102261	0.594590052	0.668205004
ZFATAS	-0.028066664	0.595057172	0.668657676
OR56A1	0.028066448	0.595060004	0.668657676
PPP1R1A	0.028047778	0.595305066	0.668895305
CD164L2	0.02804471	0.595345348	0.668898237
WSB2	0.028042462	0.59537485	0.668898237
TCTEX1D4	-0.02795189	0.596564427	0.670196907
FAM54B	0.027944472	0.596661901	0.670268601
IFITM1	0.027908309	0.597137208	0.670764708
LOC440896	0.027891581	0.597357119	0.670973887
MYH4	-0.027874381	0.597583281	0.671190065
FLRT1	-0.027808452	0.598450536	0.672126237
GYLTL1B	-0.027805042	0.598495412	0.672138732
S100A12	-0.027800796	0.598551282	0.672163572
LCE2A	-0.027766862	0.598997919	0.672627209
LOC100133	0.027762661	0.599053226	0.672651387
C5orf20	0.027756922	0.599128784	0.672698299
EFNA5	0.027740773	0.59934141	0.672865913
OR2F1	0.027740452	0.59934564	0.672865913
LOC100192	0.027707499	0.599779621	0.673315172
HTR6	-0.027661407	0.60038687	0.673952181
KRTAP10-2	0.027659292	0.600414742	0.673952181
LOC285419	-0.027645563	0.600595683	0.674117287
OR52N1	-0.027589774	0.601331184	0.674904786
COMMD10	-0.027569568	0.601597663	0.675165819
MAPKAPK2	0.0275273	0.60215529	0.675753556
MYO1G	-0.027504886	0.602451081	0.676047404
TTLL8	0.027497725	0.602545598	0.67611537
PROM1	-0.02748539	0.60270842	0.676250791
KCTD19	-0.027483162	0.602737838	0.676250791
PKD2L1	0.027480865	0.602768154	0.676250791
C10orf47	0.027441831	0.603283564	0.676790907
OPRL1	0.027433357	0.603395477	0.676878327
FZD2	0.027414241	0.603647974	0.677123432

DDX3Y	0.02740903	0.603716811	0.677162506
DAZL	-0.027399789	0.603838898	0.677242226
CTAGE4	-0.027398502	0.603855898	0.677242226
LOC100129	0.027391312	0.603950905	0.677310636
C4orf14	0.027375808	0.604155775	0.677502239
SNORA54	0.027371608	0.604211272	0.677526322
COL9A3	-0.027363326	0.604320733	0.677610911
ZNF669	-0.02735566	0.604422056	0.677686367
ARSA	0.027314763	0.604962713	0.678254373
OR2H1	0.027288728	0.605307019	0.678602189
OR11G2	0.02726797	0.605581588	0.678871789
CACNA2D4	-0.027244066	0.605897851	0.679188095
PPP1R9A	0.027236058	0.606003818	0.679268646
TMEM57	0.027232404	0.606052175	0.679284616
WNT10B	0.027207014	0.606388211	0.679623007
ADH6	0.027203099	0.606440041	0.679642849
C2orf49	0.027195397	0.606541989	0.679718851
PNMT	0.027188939	0.606627493	0.679739992
SLC5A12	0.027188816	0.606629118	0.679739992
OR5A1	-0.027181289	0.606728763	0.679813397
CCRL2	0.027153425	0.607097741	0.680188552
ITGB5	0.027148811	0.607158852	0.680218752
TTTY6B	-0.027140495	0.607268992	0.680303875
RAET1G	-0.0271343	0.607351054	0.680357534
CCDC83	0.027061946	0.608309785	0.681393182
PCOLCE	-0.027015317	0.608928015	0.682047324
NEDD4L	0.027009522	0.609004857	0.68209503
RP1-177G6.	-0.026985867	0.609318616	0.682359535
CYB561D1	0.026984362	0.609338572	0.682359535
KLHL25	-0.026983968	0.60934381	0.682359535
TRAFD1	0.026974643	0.60946751	0.682459683
CDY2B	-0.02696342	0.609616403	0.682588028
MICALL2	-0.026922561	0.610158634	0.683156754
NCR2	0.026915253	0.610255631	0.683226944
SPIB	0.026909149	0.610336657	0.683279247
OR1L4	0.026833316	0.611343704	0.684368177
LOR	-0.026829782	0.611390651	0.684382261
ADO	0.026809483	0.611660349	0.684645675
SPRR2D	0.026798061	0.611812128	0.684777076
DUOXA2	0.026792887	0.61188089	0.684815551
C10orf27	-0.026786005	0.611972354	0.684879429
KRT32	-0.026761406	0.612299319	0.685206841
YWHAQ	0.026735534	0.612643296	0.685553252
C1orf59	-0.026725679	0.612774355	0.685628549
ZNF8	-0.026725296	0.61277944	0.685628549
IKBKAP	0.026676633	0.613426726	0.686314227

XKRX	0.02663536	0.613975949	0.68689012
TBC1D20	-0.026621577	0.614159416	0.687036671
OR2H2	0.026620335	0.614175941	0.687036671
TRIM8	0.026600321	0.614442388	0.687296122
CHST6	-0.026588251	0.614603104	0.687437283
SOX13	-0.026576829	0.614755203	0.68756879
HOXB8	0.026568148	0.614870802	0.687659461
POLR2C	0.026560288	0.614975496	0.687737927
SLC8A2	0.026537207	0.615282943	0.688038088
SFTPA1	-0.026534951	0.615312997	0.688038088
SNORA1	-0.026528626	0.615397276	0.688093692
DPRX	0.026516678	0.615556469	0.68823305
RALYL	0.026503561	0.615731251	0.68838982
RAX	0.026485815	0.615967765	0.68855083
SYCN	0.026485707	0.615969204	0.68855083
CELP	0.026484973	0.61597899	0.68855083
WDR55	0.026467087	0.616217408	0.688778676
NKX2-6	-0.026462055	0.616284485	0.688814991
C1orf201	0.02645533	0.61637414	0.688876536
IGFL2	-0.026439647	0.616583256	0.689058153
PISD	-0.026437953	0.616605842	0.689058153
IPO11	-0.02641456	0.616917818	0.689368103
ACRBP	0.026411786	0.616954811	0.689370761
LOC153328	0.026406975	0.617018989	0.689403791
KCNMB2	0.026393186	0.617202922	0.689570614
C9orf6	-0.026381476	0.617359143	0.689706459
SNORA56	-0.026362307	0.61761491	0.689953493
USP22	0.026347159	0.617817066	0.690140615
MAGEF1	0.02633886	0.617927832	0.690204269
PRO1768	0.026337696	0.617943365	0.690204269
CLCNKA	0.026317714	0.618210096	0.690463466
DCP1B	-0.026312732	0.618276606	0.690499024
DDR1	-0.026304045	0.618392582	0.690589821
ZNF878	-0.026291352	0.618562065	0.690740357
RAMP2	0.026285349	0.618642222	0.690791133
APCDD1	-0.026276401	0.618761725	0.690853557
EBF4	0.026275968	0.618767507	0.690853557
C9	-0.026272208	0.61881772	0.690870888
CRABP1	-0.026240354	0.61924321	0.691307166
MGC16142	0.026233235	0.619338322	0.691340694
MLKL	-0.026231413	0.619362655	0.691340694
HIST3H2A	-0.026229176	0.619392544	0.691340694
C9orf171	-0.026227712	0.619412102	0.691340694
CSTT	-0.026217507	0.619548459	0.691446243
FCHO1	-0.026214416	0.61958976	0.691446243
SPAG16	0.02621284	0.619610829	0.691446243

ST13	0.026186343	0.619964952	0.691789099
C9orf79	-0.026182491	0.620016429	0.691789099
SNAPC5	-0.026182054	0.620022276	0.691789099
GALNT6	-0.026172858	0.620145194	0.691887481
A4GNT	-0.026157495	0.620350584	0.69207786
ANKLE2	0.026154139	0.620395462	0.692089156
ME1	-0.026144936	0.620518506	0.692187646
DEFB124	-0.026141704	0.620561725	0.692197085
LOC284632	0.026127419	0.620752758	0.69236513
PPBPL2	0.026125239	0.620781912	0.69236513
ADCY8	0.026116822	0.620894482	0.692451902
GPR162	-0.026105309	0.621048474	0.692584856
RAP1B	0.026097428	0.621153898	0.692663636
LYPD6B	-0.026076934	0.621428082	0.692930585
SMCR7	0.026068583	0.621539812	0.693016368
43900	-0.026030793	0.622045566	0.693508818
ZBTB42	-0.026030379	0.622051118	0.693508818
ATAD3C	0.026015241	0.622253754	0.693680893
UGGT1	-0.026013645	0.622275127	0.693680893
SMU1	0.026000526	0.622450772	0.693837855
ZNF85	0.025973841	0.622808109	0.694197318
PNKD	0.025961507	0.622973304	0.694342587
LOC723972	0.025953947	0.623074562	0.694416582
AIFM1	-0.025928428	0.623416434	0.694758718
GABRE	0.025916967	0.623570007	0.694883838
GSN	-0.025914841	0.623598491	0.694883838
PRPH2	-0.025908227	0.623687124	0.694943718
CHIT1	-0.025903361	0.623752329	0.694951362
CLDN2	0.025902507	0.623763776	0.694951362
STC2	-0.025884526	0.624004766	0.695180965
LCE1B	-0.025860963	0.624320638	0.695482797
FAM170B	-0.025859106	0.624345541	0.695482797
NFE2L3	-0.025845004	0.624534614	0.695654502
PI3	0.025827428	0.62477031	0.695878116
H1FNT	0.025819926	0.62487092	0.695951253
LOC256880	0.025814575	0.624942688	0.695992262
C8orf84	0.02579367	0.625223092	0.696265609
PRSS12	0.025774024	0.625486678	0.696520197
INSRR	-0.025736912	0.625984703	0.697035804
C11orf35	-0.025731548	0.6260567	0.697076997
C3orf39	0.02571526	0.62627535	0.697281467
OR2L2	0.025712563	0.626311556	0.697282796
MTFMT	-0.025707744	0.626376253	0.697315841
OR52L1	0.025702369	0.626448413	0.697338415
ZNF525	-0.025701018	0.626466562	0.697338415
ZIM3	0.025689961	0.626615027	0.697464691

STK40	-0.025686413	0.626662659	0.697478727
FASTKD3	0.025628153	0.627445203	0.698302148
TRPC1	-0.025624733	0.62749115	0.698302148
FOXE1	-0.025623504	0.62750767	0.698302148
43893	0.025610638	0.627680543	0.698437469
SSTR2	0.025608047	0.627715363	0.698437469
ELMOD3	0.025606624	0.627734484	0.698437469
C5orf47	0.025561906	0.628335536	0.699067163
SYPL2	-0.025519778	0.628901985	0.699658289
LOC100240	-0.025513067	0.628992243	0.699719613
PRG3	0.025491201	0.629286363	0.700007704
WDHD1	-0.02548738	0.629337773	0.70002579
ZFYVE27	-0.025476696	0.62948151	0.700146566
DNAJB8	-0.025423424	0.630198402	0.70090479
OSTN	0.025400235	0.630510571	0.701212823
BRP44L	-0.025387225	0.630685741	0.701368468
CA14	0.025381613	0.6307613	0.701413328
UBE2Q2	0.025360086	0.631051213	0.701666303
SLC13A2	0.025359488	0.631059259	0.701666303
SLC9A4	-0.025321932	0.63156517	0.702158093
TTC21A	0.025321419	0.631572077	0.702158093
TRIM77	0.025296594	0.631906588	0.702490773
LOC91316	0.025260472	0.632393446	0.702962112
SLC45A2	0.025259519	0.632406287	0.702962112
SNHG4	-0.025257281	0.632436463	0.702962112
LOC113230	-0.025236742	0.63271336	0.703230638
TMEM211	0.025224136	0.632883352	0.703380321
UAP1L1	0.025213789	0.633022887	0.703496138
MYL1	-0.025209455	0.633081337	0.703521836
DEFB118	0.025205744	0.633131378	0.703538187
WBSCR28	0.025200471	0.633202499	0.70357796
FAM60A	-0.025154176	0.633827045	0.704225518
OR6S1	-0.025152029	0.633856009	0.704225518
ADAM7	0.025132892	0.634114256	0.704473134
C20orf141	0.025129892	0.634154753	0.704478826
CEP78	-0.025124667	0.63422527	0.704517863
C9orf95	-0.025120295	0.634284278	0.704544113
APOL2	0.025092249	0.634662882	0.704925337
GNMT	0.025080574	0.634820503	0.705061085
CACNA1G	0.025076815	0.634871258	0.705078134
CBLN4	-0.025070066	0.634962399	0.705140031
ACSL4	0.025055283	0.635162034	0.705322399
IFITM4P	0.025044994	0.635300986	0.705403469
HTR5A	-0.025044632	0.635305881	0.705403469
RND1	-0.02503707	0.63540802	0.705477545
OLFML2B	-0.025033211	0.635460146	0.705496087

PIPOX	0.024982894	0.636139985	0.706211483
PRB1	-0.024956286	0.636499611	0.706571335
SOX2	0.024951768	0.636560678	0.706599738
NPY2R	0.024933118	0.636812803	0.706840206
LSR	0.02492903	0.636868081	0.706862166
SPATA19	0.024915477	0.637051329	0.707026149
FAM193B	0.024907758	0.63715571	0.707102589
KRTAP10-7	-0.024892804	0.637357952	0.707287619
NCRNA0005	-0.024886602	0.637441835	0.707317748
FLJ37307	0.024885545	0.637456135	0.707317748
SSTR3	0.024881428	0.637511823	0.707340127
HBA2	0.024875523	0.637591695	0.707389338
CD244	-0.024858551	0.637821291	0.707604646
C21orf128	0.02484913	0.63794875	0.707706626
PRRT4	-0.024836537	0.638119143	0.707856221
SPRR1A	-0.024801882	0.638588162	0.708334062
MYCBPAP	0.024797143	0.638652308	0.708334062
PIP	0.024796825	0.638656611	0.708334062
SLC6A10P	0.024791638	0.638726818	0.708348353
ACSS1	-0.024790618	0.638740634	0.708348353
FOLR4	0.024787827	0.63877841	0.708350801
PRSS48	-0.024771127	0.639004512	0.708562075
TOX4	0.024766563	0.639066304	0.708591139
DMP1	-0.024759929	0.639156125	0.708651277
IFT122	0.024740702	0.639416503	0.708900499
MYLK3	-0.024731794	0.63953715	0.708994786
GNAS	0.024727336	0.639597531	0.709022256
BTNL8	0.024724642	0.639634024	0.709023243
PIGS	0.024702402	0.639935291	0.709317711
TMEM136	0.02468556	0.640163474	0.709531143
PLA2G2A	-0.024654601	0.640583016	0.709936522
MFRP	0.024650681	0.640636149	0.709936522
FADS1	0.02465068	0.640636165	0.709936522
SNORA5A	0.024634942	0.640849496	0.710133414
LY6E	0.024627182	0.640954684	0.710165319
DPPA2	0.024625851	0.640972728	0.710165319
SPATA7	0.024624926	0.640985268	0.710165319
CDCP1	-0.024610183	0.641185155	0.710347261
CDK2AP1	0.02455845	0.641886742	0.711084967
PRLH	-0.024553689	0.641951329	0.71111696
PLK5P	0.024540706	0.64212746	0.711272505
NRAS	-0.024522018	0.642381019	0.711513793
HDGFL1	0.024517344	0.642444445	0.711544471
CCDC138	0.024510631	0.64253555	0.711605799
ESPNL	0.02449717	0.642718227	0.711733613
MAP3K14	-0.02449686	0.642722435	0.711733613

GUCY2E	0.024448803	0.643374818	0.71241643
SIAH3	0.024434377	0.643570699	0.71259371
C21orf94	-0.024430304	0.643626011	0.712615334
ATF6B	0.024426782	0.643673835	0.712626915
KLK13	-0.024424264	0.643708038	0.712626915
LHFPL1	-0.024414075	0.643846415	0.712740487
MAGEE2	0.024373696	0.644394944	0.71330806
EIF5A2	0.02434714	0.6447558	0.713667838
C14orf48	0.02434426	0.644794935	0.713671491
TMEM102	-0.02430276	0.645359028	0.714256143
OAZ2	-0.024292347	0.64550061	0.71437314
OR13F1	-0.02428304	0.645627156	0.714473484
C20orf151	0.024246458	0.646124651	0.714984299
CCDC151	0.024232815	0.646310242	0.715149931
PI4K2A	0.024208346	0.646643141	0.715478535
ZNF606	0.024199077	0.646769252	0.715578315
CCDC136	-0.024184436	0.64696849	0.715758984
OR2F2	0.024174023	0.64711021	0.715876004
IER2	-0.02415108	0.647422513	0.716181711
OR6K3	-0.024144099	0.647517537	0.716239788
CLCA1	-0.024141939	0.647546944	0.716239788
POLDIP3	-0.024125232	0.647774412	0.716451594
NTRK1	-0.024111405	0.647962694	0.716620039
SLC12A8	-0.024103829	0.648065873	0.71669435
MS4A12	-0.024100958	0.648104966	0.716697784
KIAA1841	0.024064897	0.648596149	0.717201127
ENO2	0.024044492	0.64887416	0.717432957
SLC7A7	-0.024044221	0.648877852	0.717432957
PPP2R5A	0.024005256	0.649408864	0.71798021
KIAA1324	0.023973357	0.649843711	0.71842109
KCNQ2	0.023941294	0.650280908	0.718864518
RIPK1	-0.023936687	0.650343746	0.718894077
PGLYRP3	-0.023923046	0.650529785	0.7190209
SNORA58	-0.02392298	0.650530684	0.7190209
IZUMO1	0.023910686	0.650698388	0.719166346
RNASE3	-0.023906133	0.65076049	0.71919507
CCDC69	0.023898899	0.650859184	0.719249944
KCNA7	-0.023897199	0.650882375	0.719249944
C12orf59	-0.023892949	0.65094036	0.719274109
TERF1	-0.023873384	0.651207314	0.719529164
SCMH1	-0.023839957	0.651663504	0.71999327
OR2K2	0.023837237	0.651700631	0.719994345
FETUB	-0.023825723	0.651857803	0.720071882
AQP8	-0.023825478	0.651861151	0.720071882
EMR1	-0.023824149	0.651879285	0.720071882
FAM25B	0.023801703	0.652185733	0.720370431

HIST1H2BC	0.02377377	0.65256717	0.720751771
NLRP9	-0.023766861	0.652661533	0.720816018
PGPEP1L	-0.023760699	0.652745688	0.720843772
ZNF529	-0.023758256	0.652779053	0.720843772
MMP7	-0.023757071	0.652795251	0.720843772
NBPF22P	-0.023734714	0.653100643	0.721141013
GCLC	-0.023699771	0.653578097	0.721628197
NUCB2	0.023692166	0.653682031	0.721702941
C1orf69	0.023681763	0.65382421	0.721819898
CALHM1	-0.023673742	0.653933838	0.721885408
CLTCL1	-0.023672117	0.653956045	0.721885408
SNORA15	-0.02366071	0.654111979	0.722017519
SEZ6L	0.023646805	0.654302068	0.722187313
KRT79	0.023629432	0.654539607	0.722409459
LOC283999	-0.023588962	0.655093083	0.722980258
CYP1A1	0.02358501	0.655147135	0.722999845
NTN5	-0.023548533	0.655646183	0.723510485
NPAS4	-0.023525637	0.655959504	0.723816131
ZNF735	-0.02352023	0.656033496	0.72385767
XKR3	-0.023475245	0.656649299	0.724496996
FOXI1	0.023449336	0.657004068	0.724848264
C15orf62	0.023443938	0.657077995	0.724889667
OR2A2	-0.023412551	0.657507901	0.725323761
C14orf64	0.023356644	0.658273934	0.72612858
LRP8	-0.023345429	0.65842765	0.726257915
MT1X	-0.023340134	0.658500218	0.726289123
C1orf129	-0.023335872	0.658558637	0.726289123
C8orf40	0.023335383	0.658565352	0.726289123
C1orf89	-0.023328183	0.658664044	0.72635774
SDCBP2	-0.023321805	0.658751475	0.726400278
OSCAR	0.023318139	0.658801739	0.726400278
C21orf49	0.023317387	0.658812042	0.726400278
SNORA22	0.023312967	0.658872649	0.726426884
KRT12	-0.023303444	0.659003213	0.72650245
BMP2	0.023302646	0.659014148	0.72650245
TLX1NB	0.023280239	0.659321413	0.726800949
VPREB1	-0.023272105	0.659432957	0.726862766
CCIN	-0.023270827	0.659450487	0.726862766
HPRT1	0.023239898	0.659874725	0.727290119
PRAMEF16	-0.023234885	0.659943503	0.72732567
DDX25	0.023226407	0.660059821	0.72741361
ALPK3	-0.023223156	0.660104415	0.7274225
ZWILCH	0.02318518	0.660625551	0.727956501
FAM7A3	-0.023165803	0.660891505	0.728209268
DIRAS1	0.023157332	0.661007795	0.728297109
RAB26	0.023142409	0.661212675	0.728482542

GSTM2	0.023136668	0.66129149	0.728529071
HLA-DQA1	-0.023121374	0.661501501	0.728720123
RXRG	0.023108723	0.661675221	0.728871177
GRB14	0.023085245	0.661997688	0.72918606
SNORA71C	0.023079912	0.662070946	0.729226419
MX2	-0.023075699	0.662128811	0.729249821
FAM47A	0.023070115	0.662205521	0.729293975
LOC100128	-0.023055115	0.662411606	0.729480597
CNTN6	-0.023049556	0.66248798	0.729524364
AZU1	-0.023025098	0.662824069	0.729854104
KRTAP7-1	0.023019101	0.662906493	0.729904506
UBIAD1	-0.023002556	0.663133891	0.73011452
INSL6	-0.022994125	0.663249784	0.730201749
CPSF6	-0.022986613	0.663353046	0.730263503
C21orf84	0.02298471	0.663379213	0.730263503
TAS2R13	-0.022980809	0.663432841	0.730282171
STK33	0.022977376	0.663480037	0.730293757
LY6K	0.022956758	0.663763526	0.730565415
FLJ45983	0.022950665	0.6638473	0.730617241
PRHOXNB	0.022946085	0.663910279	0.730646177
CDS1	-0.022915686	0.664328379	0.731065904
GCG	0.022898496	0.66456485	0.731285722
STIL	0.022887046	0.664722375	0.731418647
RAB38	-0.022860166	0.665092242	0.731785194
FGG	0.022813469	0.665734971	0.732451906
FLJ33630	-0.022790303	0.66605392	0.732762337
EPN3	-0.022731932	0.666857841	0.733606247
NR1D1	-0.022704055	0.667241905	0.73398821
STAR	0.022654333	0.667927157	0.734701426
SULT1E1	-0.022637731	0.668156027	0.734912586
BTN2A2	-0.02263056	0.668254889	0.734980732
ZNF250	0.022625906	0.668319056	0.735010714
RPH3A	0.022619344	0.668409532	0.735051186
SNORA75	0.022617883	0.668429675	0.735051186
ZDHHC14	0.02258812	0.668840113	0.73546192
NKD1	0.022569629	0.669095156	0.735701747
GPRC5D	-0.02255072	0.669356001	0.735947925
MUC20	-0.022544387	0.66944337	0.736003352
SHISA4	-0.022526608	0.66968869	0.736232418
PLXNA3	-0.022518755	0.669797052	0.736305368
SNORA61	0.02251644	0.669828991	0.736305368
MATN3	0.022506742	0.669962838	0.736411851
SLC4A9	0.022458884	0.670623445	0.737097297
LOC642587	-0.022452	0.670718486	0.737161073
RGS21	-0.022435057	0.670952426	0.737377493
ITGAL	0.02237223	0.671820219	0.738283294

FAM69A	-0.022369212	0.671861906	0.738283294
DNAJC15	0.022366693	0.671896711	0.738283294
OLFM3	-0.022363927	0.671934937	0.738283294
GSDMC	0.022361969	0.671961988	0.738283294
TCTN2	-0.022339099	0.672278003	0.738554769
CCDC102A	-0.02233872	0.672283247	0.738554769
ZNF438	-0.022325901	0.672460402	0.738708639
CCL14-CCL	-0.022301338	0.672799912	0.73904083
ASB9	-0.022273618	0.673183156	0.739421022
ELOVL4	-0.022266593	0.673280282	0.73948692
BSN	0.022258514	0.673392	0.739568835
LRP1B	-0.022236251	0.673699882	0.739866171
C1orf9	0.022232135	0.673756808	0.739887887
TNS4	0.022133997	0.675114706	0.741319518
ABCC2	-0.022132536	0.675134928	0.741319518
ZNF383	-0.022120197	0.675305738	0.741466191
LOC153684	0.022101972	0.675558054	0.741702334
BMP4	-0.022075485	0.675924806	0.742001948
CHML	0.022075258	0.675927955	0.742001948
ZNF93	-0.022074192	0.675942724	0.742001948
HGC6.3	0.022067579	0.676034296	0.742061567
ADORA2A	-0.022055765	0.676197921	0.742200264
CETP	0.022045964	0.676333672	0.742308353
LOC100130	-0.022023358	0.676646832	0.742611136
OR8K3	-0.02201096	0.676818598	0.742742596
DRD5	-0.022006785	0.676876451	0.742742596
ERF	-0.022006637	0.676878502	0.742742596
GNG13	0.022003578	0.676920879	0.742748171
C20orf195	-0.021995277	0.677035907	0.742798998
KCNH4	-0.021994852	0.677041798	0.742798998
ADAMTS14	-0.021964559	0.677461621	0.743186863
HAGH	0.021963957	0.677469962	0.743186863
ORAOV1	0.021952825	0.677624272	0.743315196
ATOH1	0.021947536	0.677697586	0.743354672
TFB2M	0.021934877	0.67787308	0.743506217
TM2D2	-0.021927065	0.677981378	0.743584048
LOC100131	-0.021911895	0.678191718	0.74377378
FAM74A1	-0.021889204	0.678506376	0.74407789
TEDDM1	0.021876882	0.678677271	0.744224319
GBAS	0.021873736	0.67872091	0.744231192
RNF34	-0.021828659	0.679346259	0.744875888
SLC25A18	0.021807055	0.67964604	0.745163559
APOBEC3G	-0.021797219	0.679782542	0.74527219
NODAL	-0.021759931	0.680300121	0.745798575
OXGR1	-0.021734181	0.680657628	0.746149428
GJA8	-0.021729092	0.680728292	0.746185817

ELP3	-0.021705914	0.681050168	0.746489106
SARDH	-0.021703771	0.681079943	0.746489106
TGIF2LY	0.021694949	0.68120247	0.746582311
NRSN1	0.021682401	0.681376765	0.74673224
SARM1	-0.021672586	0.68151311	0.746840565
ITLN1	-0.021660932	0.68167501	0.74697688
SGSM1	-0.021651128	0.681811218	0.747077735
FRG2B	-0.021648908	0.681842075	0.747077735
LOC339047	0.021640044	0.681965239	0.747171576
CTHRC1	-0.021635503	0.682028331	0.747199593
ECHDC1	-0.021630768	0.682094132	0.747230576
CTRB2	0.021623821	0.682190673	0.747295228
SOSTDC1	0.021614995	0.682313332	0.747388483
CENPI	0.021600865	0.682509731	0.747562495
S1PR5	-0.021594169	0.68260281	0.747623327
ZNF804B	0.021563968	0.683022666	0.748042036
FBXO25	0.021559982	0.683078078	0.748061585
C10orf10	0.021550855	0.683204984	0.748159422
SLAMF7	0.021515533	0.683696209	0.748656183
LOC100133	-0.021481494	0.684169715	0.749133488
C4orf26	0.021471813	0.684304404	0.749239772
FAM20C	0.021463141	0.684425059	0.749330679
FAM75A3	0.021455316	0.684533943	0.749408689
C4orf46	-0.021429232	0.684896944	0.749726105
TMEM8C	-0.021429072	0.684899173	0.749726105
MC1R	0.021405006	0.685234153	0.750051565
LOC284749	0.021393908	0.685388658	0.750155339
ProSAPiP1	-0.021392785	0.685404296	0.750155339
C2orf85	-0.021379786	0.685585266	0.750312171
ITPK1	0.021372337	0.685688982	0.750384442
C11orf53	-0.021351853	0.685974236	0.750655361
ATP5B	0.021329665	0.686283253	0.750952251
KHSRP	0.021322957	0.686376695	0.751013234
SBK2	-0.021313228	0.686512217	0.751120251
BTG1	-0.021310007	0.686557088	0.751128079
DRAM1	0.021291586	0.686813718	0.751367567
BEND2	0.021282954	0.686933983	0.751457856
TBC1D3G	-0.021265248	0.68718071	0.751686468
SPO11	0.021262378	0.687220715	0.75168894
IRX6	-0.021257548	0.687288027	0.751721279
DRP2	0.021250251	0.687389712	0.751771442
PRSS36	-0.021248839	0.687409388	0.751771442
SNX2	-0.021245137	0.687460987	0.751786589
GPR144	0.021237156	0.687572229	0.751866953
CLC	-0.021233022	0.687629847	0.751888674
SERPINF1	0.021215183	0.687878519	0.752119287

OR10G4	-0.021207906	0.687979971	0.752188917
PRAMEF18	0.021203487	0.688041586	0.752214985
LOC645323	0.021141674	0.688903573	0.753116027
FAM105B	0.021138018	0.68895457	0.753130434
EVX1	0.021125339	0.689131443	0.753282435
CKAP2	-0.021112643	0.689308567	0.753434693
LOC440563	-0.021097483	0.689520083	0.753624524
TCTN1	-0.021084389	0.689702785	0.753741894
C14orf39	0.021084362	0.689703165	0.753741894
KCNK16	-0.021014406	0.690679634	0.754767609
PRR5L	-0.021001769	0.690856071	0.754918993
ADORA1	-0.020995355	0.690945629	0.754975431
BAT2	0.020972683	0.691262253	0.755242424
CTSF	-0.020972313	0.691267418	0.755242424
ERP44	0.020968046	0.691327013	0.755242424
CAMK2A	0.020964379	0.691378225	0.755242424
CYP51A1	0.020964281	0.691379595	0.755242424
UNC13C	0.020942545	0.691683208	0.75553264
KRT82	0.020939776	0.69172189	0.755533452
FLJ45445	0.020906206	0.692190917	0.756004284
NLRP2	0.02090138	0.692258346	0.756036466
NR2E1	-0.020875991	0.692613161	0.756331183
SGCG	-0.020875545	0.692619398	0.756331183
FOXK1	-0.020873918	0.692642135	0.756331183
TMPRSS11F	-0.02086975	0.692700393	0.756353326
C14orf86	0.020851777	0.692951619	0.756586156
C1orf70	-0.02084208	0.693087169	0.756692669
NUDT7	-0.020807198	0.693574876	0.757142227
ACCS	-0.020807191	0.693574976	0.757142227
SLCO5A1	0.020797342	0.693712702	0.757251067
IQCF2	0.020747183	0.69441428	0.757975357
PDE6D	0.020723746	0.694742198	0.75827465
GMPPB	-0.020721783	0.694769653	0.75827465
SPHK2	-0.020716854	0.694838623	0.75827465
OLIG1	0.020715169	0.6948622	0.75827465
PRMT8	-0.020713979	0.694878853	0.75827465
PYROXD1	-0.020701105	0.695059021	0.758429698
EPS8L2	0.020651369	0.695755167	0.759147718
KIAA1549	-0.020646284	0.695826358	0.759183801
FAM113B	0.02063576	0.695973711	0.759302972
LMTK3	0.020615709	0.69625446	0.759567657
WDR89	-0.020612601	0.696297991	0.759573536
BCL2L1	-0.020608697	0.696352661	0.759591568
CHGB	0.020604833	0.696406776	0.759608991
EPHA5	0.020595113	0.696542898	0.759715856
UQCC	-0.020577868	0.69678444	0.759937685

AQP12B	0.020557796	0.697065614	0.76020271
PCCB	-0.020554906	0.6971061	0.760205234
SLC22A24	-0.020549274	0.697185003	0.760249649
FAM53C	0.020538794	0.69733183	0.760368123
LOC150622	-0.020517758	0.697626598	0.76064789
ASCL4	0.020510267	0.697731579	0.760708347
DDX31	0.02050835	0.697758442	0.760708347
CYP24A1	-0.020491223	0.697998478	0.760928383
LY6D	-0.020472455	0.698261548	0.761173503
HIST1H4I	0.020461529	0.698414715	0.761298799
ACTN3	0.020452518	0.698541045	0.761394829
AMELX	0.020373852	0.699644264	0.762555578
DSCR6	0.020338475	0.700140607	0.76305479
ZNF643	0.02032033	0.700395221	0.763290513
MTA2	0.020310822	0.700528663	0.763394162
KCNH5	-0.020297051	0.700721937	0.763563
NBPF6	-0.020293042	0.70077822	0.763582549
AIPL1	-0.020283249	0.700915681	0.763690545
LOC90784	0.020276092	0.701016146	0.763758222
OTUD6A	0.020269386	0.70111029	0.763781391
OR14I1	-0.020265601	0.701163429	0.763781391
PLEKHF2	-0.020263319	0.701195467	0.763781391
LRRC56	0.020262568	0.701206004	0.763781391
NCRNA0015	-0.020260918	0.701229171	0.763781391
UBE2U	0.020228835	0.701679652	0.764230258
C2orf27B	0.020221726	0.701779482	0.764297188
RPTN	0.020213148	0.701899962	0.764386599
LOC285627	-0.020205216	0.702011368	0.764457523
CYB5R2	0.020203045	0.70204186	0.764457523
GTF2H2C	-0.020189555	0.702231344	0.764622046
OR52E2	-0.020176321	0.702417238	0.764782642
SEMA4G	0.020169166	0.702517754	0.764850267
OR4N3P	-0.020159613	0.702651974	0.764954576
ARSI	-0.020150485	0.70278022	0.765052371
IQCF6	-0.020142924	0.702886464	0.765126206
ZNF83	-0.020133483	0.703019135	0.765190037
ALKBH3	0.020133283	0.703021948	0.765190037
OR4K1	-0.020125395	0.703132794	0.76526886
CLPTM1	-0.020115677	0.703269365	0.765375671
CBARA1	0.020083633	0.70371977	0.765824001
ASB12	0.020065776	0.703970816	0.766055177
C1QTNF9B	-0.020063051	0.704009131	0.766055177
C11orf85	0.020057922	0.704081244	0.766091787
FLJ39609	-0.020051769	0.70416776	0.766144063
PMS2L5	0.02002465	0.704549105	0.766465315
LOC285740	0.020024379	0.704552916	0.766465315

MTMR7	0.020022561	0.704578485	0.766465315
IL1F6	-0.019971412	0.705297968	0.767206087
RETNLB	0.019967616	0.705351363	0.767222262
KLK9	-0.019932988	0.705838621	0.76771033
SUSD4	-0.019906224	0.706215296	0.768070366
HK3	0.019903988	0.706246776	0.768070366
GFRAL	0.019890717	0.706433579	0.768231569
RIT1	-0.019873116	0.706681362	0.768440321
HLA-DRB5	0.019871599	0.706702711	0.768440321
H2BFXP	-0.019858266	0.706890445	0.76860249
TIGIT	0.01985186	0.706980645	0.768658598
LOC100134	0.019848677	0.707025456	0.768665354
ARL14	-0.019827305	0.707326425	0.768906083
XIRP2	0.019823487	0.70738019	0.768906083
MSMP	-0.019822212	0.707398149	0.768906083
DPEP1	0.019819591	0.707435072	0.768906083
TPI1P3	0.019819246	0.707439928	0.768906083
FUT3	0.019801291	0.707692814	0.769109038
TGFBI	-0.019800504	0.707703898	0.769109038
ZNF564	-0.019795164	0.707779131	0.769148827
CPXCR1	-0.019790994	0.707837872	0.769170689
SASS6	0.019764909	0.708205358	0.769454218
MNS1	-0.019764538	0.708210585	0.769454218
ANKRD1	0.019764246	0.708214703	0.769454218
PRKAR1B	0.019757272	0.708312956	0.769512913
IL25	-0.019754927	0.708346007	0.769512913
KCNJ11	0.019749534	0.708422001	0.769553491
MAP4K2	-0.019739528	0.708563	0.769664675
SLC22A14	0.019736126	0.708610931	0.769674759
CLDN15	-0.019728822	0.708713868	0.769744584
C15orf5	-0.019715508	0.708901513	0.769906399
LOC643955	-0.019700522	0.709112736	0.770093802
TEX15	-0.019681839	0.709376115	0.770337823
ZSCAN1	-0.019675957	0.709459033	0.770385858
LOC729678	0.019655672	0.709745043	0.77065441
OSTalpha	-0.019650937	0.709811815	0.770684892
SERPINB13	0.019619496	0.710255198	0.771104947
TMEM67	0.019618012	0.710276131	0.771104947
TECRL	0.019614059	0.710331878	0.771123432
HNRNPH1	-0.019601367	0.710510902	0.771275733
MYEOV	0.019596799	0.710575343	0.771303644
PLA2G4D	-0.019584943	0.710742589	0.771443136
FAM184A	0.019581832	0.710786486	0.771448736
NMUR2	-0.019547265	0.71127419	0.771935994
CDH22	0.019531732	0.711493389	0.77213181
GAS2L2	0.019518061	0.711686333	0.772297257

DBX2	0.019515435	0.711723402	0.772297257
LOC100144	0.019507044	0.711841841	0.772383691
INSM2	-0.019479914	0.712224807	0.772757125
UGT1A9	-0.01946306	0.712462769	0.772973199
DYNC1I1	0.019448088	0.712674166	0.773160431
FAM72A	0.019437004	0.712830689	0.773288112
NBPF4	-0.019425853	0.712988169	0.773416819
PCGF6	0.019413788	0.713158574	0.773559531
EXT1	0.01940741	0.713248654	0.773615105
LOC100302	-0.019382273	0.713603746	0.773944128
ACAP3	0.019380433	0.713629728	0.773944128
NCRNA0018	0.019375671	0.713697003	0.773957508
OASL	-0.019374059	0.713719791	0.773957508
C11orf46	-0.019368901	0.713792661	0.773994384
FER	0.019363348	0.713871119	0.774037314
LOC100128	0.019357472	0.713954136	0.774043891
ADAM29	0.019357417	0.71395492	0.774043891
LOC728276	-0.019312664	0.714587357	0.774687383
PILRA	0.019290531	0.714900211	0.774984361
POLH	-0.019280425	0.715043074	0.77509704
NF1P1	-0.019262603	0.715295039	0.775298539
C8orf34	-0.019259157	0.715343772	0.775298539
LOC729467	0.019257672	0.715364757	0.775298539
CABLES1	-0.019256263	0.715384683	0.775298539
FAM74A4	-0.019241851	0.715588472	0.775442133
RHPN2	-0.019241386	0.715595055	0.775442133
VSIG1	-0.019236089	0.715669953	0.775481098
CBS	0.019212174	0.716008173	0.775805373
FLJ43390	0.019201499	0.716159171	0.775914403
CDYL	0.019199551	0.716186721	0.775914403
IMP5	0.019192355	0.716288512	0.775967712
ANXA2P2	0.019190563	0.716313854	0.775967712
PNPLA2	-0.0191522	0.716856616	0.776413009
CAPN9	0.019152073	0.71685841	0.776413009
OR11H1	-0.019151751	0.716862958	0.776413009
CELF5	0.019150486	0.716880864	0.776413009
EPHB3	-0.019139643	0.717034296	0.776529511
GUSBP1	0.019137373	0.717066418	0.776529511
UBE2DNL	0.019119894	0.717313783	0.776733183
DDTL	0.019118571	0.717332498	0.776733183
MT4	0.019113084	0.717410154	0.776775035
KLHL10	-0.019104689	0.717528975	0.776861451
PLEKHH1	-0.019084658	0.717812525	0.7771262
GIGYF1	-0.01908141	0.717858502	0.777133728
CCDC33	-0.019062333	0.718128584	0.777383853
SLC25A32	0.019057785	0.718192972	0.777411297

GOLGA7B	-0.019054425	0.718240543	0.777420535
HRSP12	-0.019049356	0.718312319	0.77745597
KANK4	0.019036166	0.71849909	0.777615857
MPV17L	-0.019026888	0.71863048	0.777715792
DEFB131	-0.019020127	0.718726231	0.777777149
CTSG	0.019010679	0.718860046	0.777804993
EPHX1	0.019010337	0.718864892	0.777804993
VN1R2	-0.019010038	0.718869129	0.777804993
SNTN	0.019005607	0.718931886	0.777830635
OR10H4	-0.018993751	0.719099809	0.777970051
S100A7A	0.01898942	0.719161167	0.777994168
TMEM59L	0.018947427	0.719756094	0.778570952
TMPRSS7	-0.018946267	0.719772523	0.778570952
AADACL3	-0.018937231	0.719900563	0.778667158
STMN4	-0.018932149	0.719972576	0.778702757
D2HGDH	-0.018926296	0.720055521	0.778750173
C17orf63	0.018905897	0.720344631	0.779020545
ADAD1	0.018902943	0.720386486	0.779023505
PRR15	-0.01889124	0.720552374	0.779160587
PRRG2	-0.018886322	0.720622086	0.77919366
TBX5	0.018880278	0.720707762	0.779243992
USP17	-0.01885562	0.721057339	0.779543992
FBLN1	0.018851807	0.721111397	0.779543992
SPDYC	0.018850337	0.72113224	0.779543992
LOC649330	-0.018849663	0.721141801	0.779543992
A2ML1	-0.018834824	0.721352209	0.779709608
ECT2	0.018833335	0.721373313	0.779709608
GHRHR	-0.018822797	0.721522756	0.779828812
SNAP25	0.018802193	0.721814963	0.780062796
CHRNE	0.018802008	0.721817585	0.780062796
C18orf45	0.018796707	0.721892782	0.780063925
PPP1R13L	-0.018796411	0.721896969	0.780063925
POU4F1	-0.018781932	0.722102353	0.780243523
GPR148	0.018757622	0.72244722	0.780540033
SLC26A5	0.018757063	0.722455155	0.780540033
C2orf57	-0.018742552	0.722661037	0.780707633
PARD6G	-0.018740603	0.722688688	0.780707633
HOXA10	0.018728186	0.722864884	0.780855617
CASP6	-0.01872223	0.722949397	0.780904553
SPEM1	0.018701366	0.723245507	0.78118203
GLIPR2	-0.018691228	0.723389396	0.781295071
SEZ6L2	0.018681305	0.723530243	0.781404815
DHDDS	-0.018655923	0.723890566	0.781751566
CSPG4PY2	-0.018651873	0.723948064	0.781753747
OR2B3	-0.01865025	0.723971095	0.781753747
TMIGD2	0.018631947	0.724230978	0.781991972

INSL4	0.018618304	0.72442471	0.782158747
LOC100268	0.018606803	0.72458803	0.782292672
SPI1	0.018601342	0.724665593	0.782333999
MFNG	-0.018576921	0.725012449	0.78266603
CXCL1	-0.0185536	0.725343743	0.782981226
ZKSCAN5	-0.018545938	0.725452598	0.783056287
GPD1L	-0.01853823	0.725562108	0.783132046
APOBEC3D	-0.018530476	0.72567228	0.7832064
ARL8B	-0.018527846	0.725709651	0.7832064
FAM170A	0.018516702	0.72586801	0.783334855
PHGR1	-0.018509909	0.725964546	0.783396582
WRB	-0.018498348	0.726128838	0.783531416
CLUL1	-0.018483358	0.726341885	0.783718841
CYP2S1	-0.018478052	0.726417306	0.783757755
AADACL4	0.018461244	0.726656216	0.78397305
SLC25A2	0.018450062	0.726815186	0.784102081
MTX2	-0.018444786	0.726890191	0.78414052
ANGPT2	0.018427993	0.72712895	0.784355598
LOC100130	0.018398662	0.727546038	0.784763004
WBP5	0.01835007	0.72823719	0.785465968
AGAP4	-0.018340819	0.728368794	0.785565368
CD101	-0.018329517	0.728529591	0.785696241
ACAN	0.01831958	0.728670967	0.785806157
PWRN2	-0.018306111	0.728862622	0.785970279
CATSPER4	-0.018273679	0.729324178	0.786425416
ACSBG2	-0.01824954	0.72966778	0.786753321
NRG4	-0.018245066	0.729731472	0.786779398
LOC100190	-0.018240707	0.729793519	0.786803699
IQCH	0.018230863	0.729933659	0.786912186
SLC5A6	-0.018223385	0.730040125	0.786984361
FAM169A	-0.018216254	0.730141662	0.787051215
STRBP	-0.018207621	0.730264576	0.787141104
FAM96A	0.018200835	0.730361213	0.787202661
SLC17A6	-0.018192255	0.730483384	0.78729173
BCL2A1	0.018184535	0.730593335	0.787367621
GDF9	0.018142862	0.731186878	0.787964647
PLEKHG6	0.018117986	0.731541273	0.788303904
RAC2	0.018105209	0.731723308	0.788457401
WFDC12	-0.018084587	0.732017164	0.788731365
C6orf70	0.018067749	0.732257124	0.788947232
C10orf50	-0.018024638	0.732871631	0.789566596
PPP1R14C	-0.017995008	0.733294079	0.789978989
TMPRSS15	0.017954714	0.733868686	0.790555249
LOC729082	-0.01793962	0.734083968	0.790744386
LOC150568	-0.017924757	0.734295982	0.79091693
TMPRSS13	-0.017922823	0.734323577	0.79091693

C6orf15	-0.017903855	0.734594168	0.791165586
C1orf172	0.017872825	0.735036915	0.791599619
CSRP2	0.017864181	0.735160266	0.7916781
COLQ	0.017862147	0.735189294	0.7916781
SLC7A5P2	-0.01783917	0.735517216	0.791988395
C3orf42	0.017831758	0.735623012	0.792059488
BECN1	0.01781354	0.735883059	0.792294448
C1orf141	-0.017810897	0.735920798	0.792294448
PDX1	0.017793343	0.736171396	0.792521399
DSG3	-0.017772931	0.736462839	0.792753665
OR51B4	0.017772656	0.736466762	0.792753665
FGA	-0.017756671	0.736695031	0.79295652
DOK4	0.017737687	0.73696614	0.793167424
C1QTNF4	-0.017737373	0.736970626	0.793167424
SP6	-0.017716	0.7372759	0.793408212
IQCF5	0.017714144	0.737302408	0.793408212
STAT5A	-0.017713341	0.737313873	0.793408212
PRAMEF13	-0.017690615	0.737638524	0.793714675
SOX11	0.017667878	0.737963372	0.794021316
B3GNT3	0.017663944	0.738019588	0.794038903
TCEAL4	-0.017649955	0.738219473	0.794211053
HAPLN3	0.017638457	0.738383792	0.794344924
KRT84	-0.017631321	0.73848578	0.794411727
IGFL1	-0.017603813	0.738878943	0.794791733
APOC3	-0.017600554	0.738925529	0.794796445
C2orf88	0.017597923	0.738963143	0.794796445
NSF	-0.017588816	0.739093322	0.794893531
HPSE	-0.017574641	0.739295968	0.795029921
ZCCHC12	-0.01757436	0.73929998	0.795029921
FAM5B	-0.017566175	0.73941701	0.795112838
DES	-0.017561699	0.739481009	0.795138724
ABCB1	-0.017547472	0.73968443	0.795314514
PAX1	-0.017538881	0.739807282	0.795403662
TSPAN16	0.017522664	0.740039188	0.795610043
GALP	0.017509073	0.740233577	0.79577607
ORC4L	0.017501575	0.740340821	0.795848399
NUDCD3	0.017470843	0.74078042	0.796277976
NCRNA0020	0.017437845	0.74125255	0.79670336
ALG1L2	0.017437592	0.741256167	0.79670336
PAX3	0.017431314	0.741346	0.796756912
KIFC3	0.017427518	0.741400316	0.79677229
MYH3	0.017413454	0.741601583	0.796945581
FLJ43663	-0.017392064	0.741907721	0.797231547
NPL	0.017387086	0.741978965	0.797257542
DAPL1	-0.017379673	0.742085083	0.797257542
MTDH	0.017379204	0.742091796	0.797257542

KRTAP2-1	0.017375939	0.742138524	0.797257542
DNAH5	0.017375852	0.742139772	0.797257542
LOC100302	0.017373593	0.74217211	0.797257542
AMAC1	-0.017343558	0.742602093	0.797676412
IFNB1	-0.017330289	0.74279207	0.797801761
OR2AG2	0.017329812	0.742798908	0.797801761
ZIC3	0.017313631	0.7430306	0.798007572
GRIA4	-0.017306247	0.743136346	0.798078102
PTCHD2	0.017265528	0.743719541	0.798661346
LOC730668	0.017261586	0.743776011	0.79867892
LOC157627	0.017257953	0.743828052	0.798691737
ACSM4	-0.017224913	0.744301392	0.799156902
LILRB3	0.017216952	0.744415461	0.799236288
NCALD	-0.017201773	0.744632961	0.799426707
MXRA7	-0.017197947	0.744687791	0.799432156
C3orf16	0.017193621	0.744749788	0.799432156
NKX6-1	0.017190737	0.744791122	0.799432156
LOC148824	0.017190215	0.744798605	0.799432156
MT1E	-0.017183322	0.744897382	0.799467519
DHRS7	0.017181197	0.744927835	0.799467519
IL22RA1	0.017179512	0.744951984	0.799467519
TREML1	-0.017160572	0.745223447	0.799675022
FAM98A	-0.017160419	0.745225646	0.799675022
LOC219347	0.017145265	0.74544286	0.799840224
FBXO42	-0.017142101	0.745488211	0.799840224
HOXC4	0.01713895	0.745533381	0.799840224
ACSS2	0.01713641	0.7455698	0.799840224
CA5A	-0.017130605	0.745653023	0.799840224
STON2	0.017130221	0.745658528	0.799840224
ENPP6	0.017130067	0.745660738	0.799840224
TAS2R43	0.017123012	0.745761878	0.799905628
CDC23	-0.017101272	0.746073572	0.800196854
KLK15	0.017086161	0.746290267	0.800386163
RNASE11	-0.017082653	0.746340566	0.800397004
LALBA	0.01705122	0.746791369	0.800837333
MATN1	-0.017040083	0.746951119	0.800951119
C10orf116	0.017035964	0.7470102	0.800951119
DNASE2	0.017035411	0.747018132	0.800951119
JAKMIP1	0.017028788	0.747113136	0.801009858
OVOL2	-0.017008204	0.747408433	0.801283319
FAM118B	0.017005161	0.74745209	0.801286987
GOT1L1	-0.01699587	0.747585391	0.801386751
ANKRD20B	-0.016992607	0.747632215	0.801393808
FAM184B	0.016979011	0.747827292	0.801559769
MAP1S	0.016957163	0.748140827	0.801852676
RAD51AP2	-0.01694674	0.748290409	0.801969836

CDR1	0.016943252	0.748340473	0.801980332
LOC654433	0.016920459	0.748667634	0.802287771
C4orf35	0.016903293	0.748914046	0.802468307
C19orf59	0.016903109	0.748916694	0.802468307
GOSR2	0.016884579	0.749182724	0.802710171
OC90	0.016849171	0.749691154	0.803211713
TBC1D28	0.016838369	0.749846288	0.803334705
LILRP2	0.016832809	0.749926129	0.803377023
MMP15	-0.016791301	0.750522351	0.803918075
NRXN2	-0.016790727	0.750530591	0.803918075
AIM2	-0.016787262	0.750580375	0.803918075
ONECUT3	-0.016786001	0.750598489	0.803918075
ST8SIA1	-0.016783597	0.750633021	0.803918075
LOC100240	0.016779566	0.750690939	0.80393687
CHDH	-0.016771191	0.75081126	0.80402249
IL1F10	0.016761611	0.750948898	0.804087657
CRTAM	0.016761335	0.750952866	0.804087657
VILL	-0.016752549	0.751079113	0.804179598
TUBA3D	-0.01673925	0.751270215	0.804340968
C17orf65	0.016731801	0.751377251	0.804412319
PCBP1	-0.016694994	0.751906263	0.804935399
ASB2	0.016688415	0.752000821	0.804993354
CCDC27	0.016678648	0.752141222	0.805100373
ZIM2	0.016669913	0.752266791	0.805174682
MPP2	-0.016668194	0.752291504	0.805174682
UNC80	-0.016612768	0.753088452	0.805984336
RBMY1F	-0.016609693	0.753132676	0.805988353
UBQLN3	0.016599166	0.753284072	0.806107055
PFN2	-0.016592101	0.753385683	0.806172472
TPO	-0.01655418	0.75393115	0.806712812
LPCAT3	-0.016544606	0.75406888	0.806816835
EPHX3	0.016528664	0.754298255	0.807018897
OR10H3	0.016521115	0.754406876	0.80709175
RNF216	-0.016500863	0.754698285	0.807360139
CHFR	0.016475783	0.755059231	0.807681402
GSTM1	-0.01647436	0.755079706	0.807681402
HLA-L	0.016459972	0.755286796	0.807859528
CXorf1	0.016444362	0.7555115	0.808056473
RHOV	0.016433098	0.755673656	0.808186502
B4GALNT2	-0.016417521	0.755897916	0.808382934
PRPS1L1	-0.016405596	0.756069606	0.808523128
FUT6	0.016402558	0.75611335	0.808526491
LRWD1	0.016397996	0.756179042	0.808553322
USP50	-0.016392467	0.756258649	0.808595029
LOC728819	-0.016368351	0.756605932	0.808922915
SPRR3	-0.016341025	0.756999514	0.809300264

ZNF286B	0.016323135	0.757257203	0.809532299
SNORA23	0.016265193	0.758092027	0.810381252
RTP4	-0.016256315	0.758219964	0.810474509
ZNF337	0.01621384	0.758832156	0.811085358
TMEM51	-0.016185038	0.759247353	0.811485592
AP2B1	-0.016173841	0.759408785	0.811614573
MSRA	-0.016169837	0.759466513	0.811616922
CHRNG	0.016168036	0.759492491	0.811616922
FSCN3	-0.016143723	0.759843073	0.811947996
OR2T4	0.016115623	0.760248308	0.812294047
STX19	0.016115611	0.760248493	0.812294047
NCRNA0008	-0.016105651	0.760392135	0.812360231
SLC6A7	0.016103282	0.760426305	0.812360231
OTOF	0.016102378	0.760439345	0.812360231
CNPY1	0.016100003	0.760473602	0.812360231
PRR23A	0.016087952	0.760647434	0.812477318
SYT14	-0.016081943	0.760734119	0.812477318
ASF1A	0.016081626	0.760738698	0.812477318
MDH1B	-0.016081092	0.7607464	0.812477318
CRIP2	-0.016075781	0.760823013	0.812515568
NSUN7	0.016067418	0.760943667	0.812600843
TTC23L	-0.016058503	0.761072276	0.812694604
MRGPRX2	-0.016053628	0.761142616	0.812726138
ARHGEF16	0.016018576	0.761648385	0.813182533
PAX9	0.016017522	0.761663594	0.813182533
SLC28A2	-0.016015517	0.761692542	0.813182533
HSF5	0.015985186	0.762130286	0.813606253
APOBEC3H	-0.015963885	0.762437742	0.813875527
MYBPC2	-0.015962049	0.762464258	0.813875527
ZCCHC13	-0.015899046	0.763373887	0.814778958
AP4S1	0.015897761	0.763392446	0.814778958
MCOLN3	-0.015882605	0.763611322	0.81496889
C1orf157	0.015874336	0.763730751	0.815052672
IL27RA	0.015862704	0.763898744	0.815188269
RAD18	0.015851766	0.764056737	0.815313182
DEPDC4	-0.015837106	0.764268501	0.815452463
GADL1	-0.015837061	0.764269155	0.815452463
LOC400940	0.01577968	0.765098195	0.81629329
DPY19L2P2	0.015774472	0.765173455	0.816329852
UGT2B4	0.015765753	0.765299463	0.816418523
TUBA8	0.015763047	0.765338559	0.816418523
SPINLW1	-0.015755834	0.7654428	0.816485986
MAGEB10	0.015733748	0.765762016	0.816782741
PBX4	0.015724219	0.765899753	0.816885903
SEMA3E	0.015714116	0.766045802	0.816997919
ATF7IP2	-0.015710492	0.766098193	0.817010042

ABI3	0.015689141	0.766406858	0.817295452
SCD	0.015664601	0.766761675	0.817630047
C14orf34	0.015661654	0.766804287	0.817631706
AKAP4	0.015649333	0.766982448	0.817735897
NLRP8	-0.015649218	0.766984124	0.817735897
C9orf96	0.015631727	0.767237063	0.81794925
PDCD7	-0.015628993	0.767276613	0.81794925
MAGEA5	0.015624823	0.767336922	0.81794925
HORMAD1	-0.01562402	0.767348523	0.81794925
WISP3	0.015583377	0.767936422	0.818532104
ANXA6	0.015574921	0.768058749	0.818618677
SMARCD3	-0.01556131	0.768255669	0.81878474
ATP6V0E2	0.015556557	0.768324428	0.818814202
FKSG83	-0.015544959	0.768492248	0.818911924
FCHSD1	0.015544536	0.768498366	0.818911924
FLJ40292	-0.01554161	0.768540705	0.818913223
C16orf58	0.015536342	0.768616933	0.81895063
COX15	-0.015532603	0.768671029	0.818964452
GRAMD1A	0.015529099	0.768721734	0.818974661
PSKH2	-0.015516438	0.76890496	0.819103915
FAM166A	0.015513719	0.76894431	0.819103915
TEKT3	0.015512189	0.768966447	0.819103915
MPO	-0.015496901	0.769187698	0.819266258
OR12D3	-0.015495079	0.769214072	0.819266258
FEZF1	-0.015493131	0.769242267	0.819266258
MAP2	-0.015487907	0.769317879	0.819302972
ISL1	0.015480893	0.769419405	0.819367278
FGFBP1	-0.015461961	0.769693451	0.819615286
ZNF454	-0.01544539	0.769933345	0.819826903
DENND2D	0.015424493	0.770235893	0.820064907
SHOX	0.015424263	0.770239221	0.820064907
CT47A1	-0.015396214	0.770645374	0.820412579
KC6	0.015396022	0.77064816	0.820412579
LOC100133	0.015381666	0.770856052	0.82059003
ST7	-0.015378781	0.770897833	0.820590643
GTPBP8	-0.015328011	0.771633228	0.821329542
ZNF35	-0.015310612	0.771885291	0.821535207
ASPDH	-0.015308979	0.771908952	0.821535207
RGPD8	0.01529844	0.772061646	0.821653806
YEATS4	0.0152908	0.772172349	0.821727708
SIGLEC12	0.015279527	0.772335689	0.821857614
C5orf56	-0.01526568	0.772536356	0.822027224
LOC645431	0.015251949	0.772735339	0.822195025
NBR2	0.015246197	0.77281871	0.822239802
PLEKHG4B	-0.015236809	0.772954769	0.822340628
RGS2	0.015226767	0.773100329	0.822451551

ZNF490	0.015219355	0.773207755	0.822521898
CPA1	0.015187368	0.773671466	0.822928845
C3orf20	0.015187265	0.773672948	0.822928845
SNORA3	-0.015156936	0.774112699	0.823352617
TBXAS1	-0.015149411	0.774221814	0.823424696
STAP1	0.015118729	0.774666766	0.823853927
SLC15A2	-0.015094712	0.775015103	0.824180369
VWDE	-0.015084482	0.775163492	0.824294156
TMEM64	-0.015078609	0.775248688	0.824340735
GBGT1	-0.015073256	0.775326337	0.824379284
OAS1	-0.015060682	0.775508755	0.824509625
XBP1	-0.015059099	0.775531725	0.824509625
DCLK3	-0.015047848	0.77569495	0.824639135
FAM10A4	0.01504021	0.775805778	0.824671328
ANXA2P1	0.015040053	0.775808051	0.824671328
MTBP	0.015028451	0.775976397	0.824806253
TCTA	0.015016505	0.77614974	0.824946474
OR1J2	0.01500421	0.776328167	0.825092082
SDHB	0.014973987	0.776766791	0.825514203
OR13G1	0.014969673	0.776829415	0.825536702
LOC340357	0.014931915	0.777377524	0.826075097
NTNG1	0.014899806	0.777843702	0.826526375
SERPINB3	-0.014896669	0.777889261	0.826530685
ALG9	0.014887354	0.778024521	0.826618418
C16orf57	-0.014885266	0.778054846	0.826618418
LDOC1	-0.014848664	0.778586404	0.827139028
ATXN3L	0.014829914	0.778858748	0.82738422
MC3R	-0.014777402	0.779621622	0.828150448
SPRR4	-0.014765301	0.779797445	0.828293036
TMEM65	-0.01474099	0.780150722	0.828624087
ACSL5	-0.014727855	0.780341613	0.828782637
CHL1	0.014720798	0.780444168	0.82882739
OR8S1	-0.014719228	0.780466987	0.82882739
KRT40	0.014711587	0.780578056	0.828858637
GPR109B	-0.014711477	0.78057965	0.828858637
PRF1	-0.014708566	0.780621958	0.828859368
CELA1	0.01470164	0.780722633	0.828922069
LOC100131	-0.014693724	0.780837694	0.829000036
ZNF503	0.014687492	0.780928282	0.829052015
OR4D10	-0.0146817	0.781012486	0.82909721
IRGM	-0.014678144	0.781064172	0.829107883
PLAC2	-0.014673605	0.78113016	0.829133735
SLC16A5	-0.014661656	0.781303869	0.829273919
TSPAN31	-0.014633805	0.781708819	0.829659515
SNW1	-0.014619803	0.781912425	0.829831385
MED4	0.014609014	0.782069315	0.829953661

OR2W5	0.014601579	0.782177435	0.830024172
ZNF202	-0.014587316	0.782384874	0.830200062
AKR1E2	0.014570087	0.782635459	0.830421715
TMEM143	0.01453884	0.783090007	0.830859749
PYGB	-0.014534046	0.78315974	0.830889469
CHRNA9	0.014526264	0.783272968	0.83096533
GPR22	0.014507906	0.783540057	0.831204402
TMBIM4	-0.014487687	0.783834264	0.831472215
TP53TG5	-0.014459826	0.784239722	0.831858006
RBP3	-0.014422762	0.784779188	0.832385893
PLEK2	0.014406144	0.785021096	0.832598132
HBG2	-0.014387222	0.785296578	0.832845956
FGF20	-0.014375855	0.785462077	0.832972077
ESYT3	0.014373308	0.785499152	0.832972077
LOC285548	0.014351677	0.785814126	0.833261717
LOC285375	-0.014347024	0.785881878	0.833289192
YWHAH	0.014323586	0.786223217	0.833606738
SERPINB12	-0.014318611	0.786295665	0.83363917
UPK2	0.014305304	0.786489488	0.833800274
DLX3	0.014289316	0.786722358	0.834002756
TMEM140	-0.014260876	0.78713666	0.83439754
PCDHA9	-0.014228519	0.787608102	0.834852851
GRIN3A	-0.014224096	0.787672542	0.834876719
C9orf78	0.014216395	0.787784758	0.834951221
ZDHHC8P1	0.014188626	0.788189432	0.835335668
NNMT	0.014168932	0.788476468	0.835595406
ALCAM	0.014151407	0.788731915	0.83582164
PPP1R2P9	-0.01414248	0.788862044	0.835915058
SH3GL3	-0.014135146	0.788968953	0.83593273
ZFR2	0.01413511	0.788969484	0.83593273
DCUN1D2	-0.014130872	0.789031271	0.83593273
SERPINA11	-0.014129819	0.789046621	0.83593273
SLC38A3	0.014119178	0.78920175	0.836052602
LOC284276	0.014110344	0.789330541	0.83614456
TATDN3	-0.014101851	0.789454367	0.83623125
SLC6A2	0.014031329	0.790482782	0.837276067
SAA1	-0.014001953	0.790911281	0.837685378
DNAI2	-0.013996076	0.790997016	0.83773163
CS	0.013992667	0.791046744	0.837739745
NT5E	0.013983816	0.791175866	0.837831936
MAST2	-0.013950009	0.791669125	0.838309705
ZMAT4	0.013931975	0.791932268	0.838543764
CRLF1	0.013929057	0.791974856	0.838544275
RNASE2	0.013914509	0.79218716	0.838724471
C15orf56	0.013900175	0.792396355	0.838901356
EGLN3	0.013891174	0.792527724	0.838995834

HARBI1	0.013884555	0.792624347	0.839053519
CCDC149	0.013870372	0.792831367	0.839226766
PIN1L	0.013867569	0.792872287	0.839226766
LOC150776	0.013855291	0.793051518	0.839371864
CD86	-0.013842544	0.793237607	0.839524204
SOX3	0.01383268	0.793381607	0.839631986
PPA2	0.013752085	0.794558527	0.840832832
CD40	-0.013686074	0.795522819	0.841772159
DPP3	0.013685535	0.795530696	0.841772159
COQ10A	0.013665987	0.795816313	0.842029639
FOXRED2	-0.013656483	0.795955193	0.842131843
CCL1	0.013645203	0.796120023	0.842237223
PTPDC1	-0.013643878	0.796139378	0.842237223
FAM154A	-0.013630824	0.796330153	0.842394295
AGTR1	-0.013624145	0.796427764	0.842452803
PYDC2	0.013620175	0.796485788	0.842469432
SFTPB	-0.013609658	0.796639502	0.842587269
GKN1	-0.013598815	0.796797982	0.842710134
ABCB9	0.013557348	0.797404148	0.843306442
GJD2	0.0135455	0.797577363	0.843444838
OR2T5	0.013529959	0.797804594	0.843640339
OXT	0.01352569	0.797867013	0.843661546
C1orf186	0.013520207	0.797947192	0.843675752
WDR16	-0.013518977	0.797965176	0.843675752
TSGA14	0.013508867	0.798113008	0.843787257
OPTC	-0.013483645	0.798481853	0.844128004
WFDC2	0.013478414	0.798558352	0.844128004
OR5B12	0.013478133	0.79856247	0.844128004
THEG	0.013468411	0.798704659	0.844233495
OSGIN2	-0.013461285	0.798808874	0.844256203
CRP	0.013459382	0.79883672	0.844256203
CCDC63	-0.013458247	0.798853321	0.844256203
SYCE1	0.013454268	0.798911514	0.8442729
MT2A	0.013439943	0.799121049	0.844449521
SCARNA22	0.013417432	0.799450363	0.844752691
OR51E2	0.013399762	0.799708874	0.844981018
FAM20A	-0.013393674	0.799797949	0.845021766
HLA-J	0.013391326	0.799832301	0.845021766
HTR1E	-0.01334717	0.800478425	0.845637475
ARHGAP15	0.013342348	0.800548997	0.845637475
IQCB1	-0.013341786	0.800557211	0.845637475
YPEL4	-0.013337305	0.800622796	0.845637475
TBC1D25	0.013336991	0.800627396	0.845637475
ABRA	0.01332023	0.800872693	0.845851701
TSPAN7	0.013308844	0.80103935	0.845982853
VASH2	0.013304369	0.801104854	0.846007168

LOC221442	-0.013300842	0.801156484	0.846016829
LRTM2	0.013294955	0.801242644	0.846062951
OR5AU1	-0.013276282	0.801515997	0.846306722
HLA-E	-0.013264739	0.801684986	0.846437042
C12orf61	0.013261421	0.801733554	0.846437042
PGS1	0.013259142	0.801766928	0.846437042
HTN3	-0.013251067	0.80188515	0.846516977
CYP11B1	0.013237593	0.802082427	0.846680353
DMRT3	-0.013223108	0.802294525	0.846859355
MMP10	0.013204943	0.80256052	0.847083537
FBXO47	0.013202795	0.802591979	0.847083537
CXorf50B	-0.013146043	0.803423195	0.847909831
FAM22F	-0.01314215	0.803480214	0.847909831
AKR1B1	0.01313778	0.803544232	0.847909831
CTNND2	0.013137715	0.803545179	0.847909831
A2LD1	0.013124041	0.803745498	0.848076273
ANKS1B	-0.013120963	0.803790588	0.848078917
SCGB3A2	-0.013115644	0.80386852	0.84811621
ZNF354A	0.013085191	0.804314686	0.848541981
FAM75A5	-0.013075465	0.804457213	0.848585706
SPOCK2	-0.01307539	0.804458309	0.848585706
KIF14	0.013073639	0.804483963	0.848585706
CYLC1	-0.013065509	0.804603092	0.848666414
RIC8A	-0.013062393	0.80464875	0.848669625
IFNE	-0.013056104	0.804740923	0.848721892
BLID	0.01304478	0.804906871	0.848851956
CELA2A	-0.013033743	0.805068627	0.848977586
HEBP1	-0.013014744	0.80534708	0.849226258
SLC25A27	0.012987938	0.805740015	0.849595616
HIST1H4L	0.012958362	0.806173605	0.850007801
ANKRD53	-0.012919421	0.806744568	0.850564776
LCE2C	0.012910698	0.806872477	0.850654599
SLC46A1	-0.012886838	0.807222397	0.850978457
HIGD1C	-0.012858416	0.807639261	0.85133375
LDLRAD1	0.01285803	0.807644918	0.85133375
MOV10L1	0.012847463	0.807799913	0.851452062
LEAP2	0.012824846	0.808131693	0.851756691
CASC1	0.012819829	0.808205297	0.851781559
LCE3A	0.012817396	0.808240993	0.851781559
SPDYE7P	0.012814491	0.8082836	0.851781559
OR1J1	0.01278699	0.808687092	0.852161672
OR10V1	-0.012763269	0.809035159	0.852483343
C6orf195	-0.012754206	0.809168158	0.852578375
PLA2G6	-0.012728045	0.809552088	0.852937775
CRIPAK	-0.012717263	0.809710329	0.853059367
POU6F2	-0.012710737	0.809806115	0.853115149

CRHR1	-0.012704324	0.809900244	0.853169181
HS3ST5	0.012690861	0.810097861	0.853332217
STRAP	0.012675651	0.810321136	0.853522263
CADM2	0.012662558	0.810513349	0.853679572
GGT7	-0.012653735	0.810642884	0.853733901
PTGES	-0.012653205	0.810650669	0.853733901
POLM	-0.012643871	0.81078771	0.853833073
C4orf45	0.012640002	0.810844516	0.853847744
GLB1L	0.012609895	0.811286594	0.854268097
ERC2	-0.012590594	0.811570026	0.854521364
C2orf56	0.012582916	0.811682786	0.854594907
PLD3	0.012563442	0.811968801	0.85485085
ARRB1	-0.012552944	0.812122989	0.854967983
C9orf84	-0.012543887	0.812256025	0.855031328
SCARNA9L	-0.012543002	0.812269028	0.855031328
TP53AIP1	0.012537045	0.812356528	0.855078237
ZNF20	-0.012509757	0.812757385	0.855454961
GPR110	-0.012461899	0.813460543	0.85614981
TRAF7	-0.012448596	0.813656037	0.856310309
HIST1H2BE	-0.01243923	0.813793661	0.85640989
MAG	-0.012428775	0.813947314	0.856526328
TRIM26	0.012423297	0.81402782	0.856565784
HOXA4	-0.012412915	0.814180411	0.856681085
SP140L	0.012401799	0.814343791	0.856807723
CLLU1OS	-0.012391612	0.814493517	0.856873884
CPPED1	-0.01238892	0.814533084	0.856873884
OR7D2	0.012385866	0.814577976	0.856873884
RUFY4	-0.012385812	0.814578778	0.856873884
HLX	-0.012369831	0.814813686	0.857075717
CNIH3	-0.012364399	0.814893544	0.857114446
SERPINA7	-0.012351039	0.815089937	0.857241535
CYP2J2	0.012350172	0.815102689	0.857241535
MLF1	-0.012347396	0.815143507	0.857241535
EREG	-0.012319321	0.815556272	0.857630328
CXCL16	-0.012313593	0.815640498	0.857673611
SORT1	-0.01228756	0.816023299	0.858030835
RBPMS	0.012279951	0.816135202	0.858103192
PKLR	0.012273277	0.816233342	0.858117744
IRS4	-0.01227315	0.816235221	0.858117744
THY1	-0.012269939	0.816282438	0.858122084
HLA-DPB1	0.012266319	0.816335683	0.85813276
E2F3	-0.012260843	0.81641622	0.858172122
NUDT13	-0.01224819	0.816602313	0.858295185
OPRM1	0.012247022	0.816619491	0.858295185
SYT12	-0.012236747	0.816770621	0.858408724
TPPP3	0.012226771	0.816917353	0.85851763

GFI1	0.012221369	0.816996816	0.858555833
GNAT3	-0.012180824	0.817593273	0.859137296
TEX14	-0.012152161	0.818015001	0.8595351
HIST2H3C	0.012147592	0.818082236	0.859560395
POMC	0.012131324	0.818321609	0.859766544
LOC134466	0.012128042	0.818369916	0.859771939
IGLON5	0.012123082	0.818442902	0.85980326
GRM2	-0.012110468	0.818628538	0.859952913
N4BP3	0.012094768	0.818859597	0.860150265
EPRS	0.012089949	0.818930527	0.860179402
ACOT11	-0.012079984	0.819077186	0.860288073
NEDD9	0.0120642	0.819309518	0.860486713
ABCC5	-0.01205841	0.819394755	0.860530852
MRRF	-0.012043033	0.819621107	0.860723178
ALPPL2	0.012036506	0.819717192	0.860778691
ANKRD22	0.011993746	0.820346752	0.861394367
44080	-0.011984021	0.820489943	0.8614993
COL24A1	0.01195938	0.82085279	0.861834843
UBASH3A	-0.011952487	0.820954305	0.861895988
DHCR24	-0.011930855	0.821272893	0.862185011
C20orf123	-0.011907598	0.821615439	0.862499155
ZNF773	0.011901895	0.821699442	0.862541871
COPS8	0.01189237	0.821839749	0.862643683
KRT73	0.011844616	0.822543272	0.863336633
ZNF629	-0.011824518	0.822839394	0.863601925
C1orf158	0.01181688	0.822951944	0.863674534
AHSP	0.011806864	0.82309954	0.863783914
SPANXN2	0.011797497	0.823237565	0.86386011
RHOH	-0.011796049	0.823258902	0.86386011
GPR172B	-0.011792745	0.8233076	0.863865693
SNORA53	-0.011787913	0.823378801	0.863894886
OR10P1	0.011771424	0.82362181	0.864104328
SLC10A4	0.011760107	0.823788608	0.864233795
PLA2G4C	-0.011752041	0.823907499	0.864312993
CCL28	0.011743788	0.824029145	0.864395071
ZDHHC3	0.011740601	0.824076118	0.864398815
HTRA4	-0.011728017	0.82426161	0.864547846
LOC644669	0.011700967	0.824660375	0.864920548
SLC4A5	0.011689279	0.824832682	0.865055709
RARS2	0.011664201	0.825202429	0.865397913
KRT39	0.011659241	0.825275568	0.865429042
TEX101	-0.011646577	0.825462301	0.865579282
RRBP1	0.011630152	0.82570451	0.865780579
FAM19A3	-0.011627663	0.825741216	0.865780579
KRTAP3-3	-0.011580535	0.826436281	0.866433029
GDF11	0.011579571	0.826450505	0.866433029

C1orf87	0.011569049	0.826605704	0.866550118
HPX	-0.011563859	0.826682265	0.866555896
ASL	0.011562776	0.826698241	0.866555896
LGALS12	-0.011552232	0.826853779	0.866673316
PIGY	0.011533303	0.827133016	0.866887453
PRINS	-0.011531186	0.827164251	0.866887453
BMP7	0.011528871	0.827198395	0.866887453
ADAM30	0.011525483	0.827248387	0.866887453
CASKIN1	0.01152363	0.827275724	0.866887453
INTS3	0.011520003	0.827329233	0.86689791
CTTN	0.011512863	0.827434574	0.866931538
ARX	-0.011508849	0.8274938	0.866931538
TBX3	-0.011503415	0.82757398	0.866931538
SNORA14A	-0.01150317	0.827577591	0.866931538
FXC1	0.011503075	0.827578985	0.866931538
TTC39C	-0.011484106	0.827858883	0.867179132
FGFBP2	0.011460933	0.828200838	0.867491699
EFHC2	-0.011439997	0.828509793	0.867769671
OR6B2	0.011434949	0.828584302	0.867795124
BAIAP2	0.011432446	0.828621245	0.867795124
PET112L	-0.011418257	0.828830661	0.867968797
RTP2	0.011408352	0.828976864	0.868076257
RRM1	-0.011397725	0.829133726	0.868194866
CLIP4	-0.01139202	0.829217936	0.868237392
DGAT2	0.011381968	0.829366307	0.86834709
TUBB4Q	0.011367102	0.829585765	0.868531202
LCK	0.01134481	0.82991488	0.86879124
NCRNA0016	-0.011344369	0.829921394	0.86879124
ZNF740	-0.011332431	0.830097644	0.86893007
ACTL6A	-0.011326401	0.830186681	0.868977596
COPZ2	-0.011288939	0.830739849	0.86951091
UTY	0.011263042	0.831122302	0.869865495
BCR	-0.011258382	0.831191132	0.869891817
ABHD1	-0.011254957	0.831241714	0.86989904
TTTY15	-0.011237447	0.831500337	0.870123966
CPXM1	-0.011228308	0.831635322	0.870219495
PPP1R3F	0.011212577	0.831867699	0.87041692
COTL1	0.011193115	0.832155198	0.870671996
NCRNA0017	-0.011142586	0.832901753	0.871377631
USP26	0.011141545	0.832917127	0.871377631
NUCKS1	0.011126522	0.833139116	0.871564085
TMEM213	0.011123133	0.833189197	0.871570693
DUSP26	-0.011092957	0.83363514	0.871991376
C9orf3	-0.011085394	0.83374692	0.871997561
OR2G6	0.011084506	0.833760039	0.871997561
C10orf32	0.011081563	0.833803544	0.871997561

ZNF488	0.011080706	0.833816197	0.871997561
ATP4A	0.01105219	0.834237679	0.872392531
KRTAP10-1	0.010993308	0.835108143	0.873256953
SMTN	0.010972049	0.835422464	0.873539765
HHATL	0.010926489	0.836096176	0.874198317
OR52N5	0.0109083	0.836365184	0.874433675
CROCC	0.010899126	0.836500863	0.874507471
FAM117A	0.010894694	0.836566416	0.874507471
AGR3	-0.01089462	0.836567503	0.874507471
PMPCB	0.010878018	0.836813069	0.874684783
OR56A4	0.010877214	0.836824965	0.874684783
CLCA3P	0.010863904	0.837021834	0.874844643
ATP6V1E2	-0.010860323	0.837074811	0.874854099
TNFAIP8L3	-0.010843881	0.837318042	0.875062385
FBXO17	-0.010834381	0.837458573	0.875163325
PSKH1	0.010815243	0.837741717	0.87541328
TOLLIP	0.010807339	0.837858651	0.875489534
SHB	0.010777905	0.838294169	0.875863609
APOB48R	-0.0107772	0.838304607	0.875863609
VWA3A	0.01077231	0.83837696	0.875893252
CYP3A5	-0.010758743	0.83857773	0.876057048
LOC644145	-0.010751115	0.838690607	0.876129009
LOC91948	-0.010702004	0.839417455	0.876838066
RBM28	-0.010699304	0.839457422	0.876838066
ACHE	0.010688132	0.839622789	0.8769648
HTA	0.01067352	0.839839093	0.877144721
CHORDC1	-0.010668377	0.839915218	0.877146478
SPRR2C	-0.010665941	0.839951283	0.877146478
GPR160	-0.01066448	0.839972909	0.877146478
MMP26	0.010649352	0.840196862	0.877324362
SNX24	-0.010647022	0.840231361	0.877324362
HOXD3	0.010625252	0.840553668	0.877614884
REXO2	0.010613141	0.840732992	0.877756096
TMPRSS12	0.01056997	0.841372249	0.878377456
OR1F2P	0.010545083	0.841740824	0.878716177
ARHGAP9	-0.01053763	0.841851206	0.878756723
KRTAP17-1	-0.010536012	0.841875168	0.878756723
C17orf44	-0.010533522	0.841912039	0.878756723
UBE2K	-0.010527498	0.84200126	0.878803789
PIGM	0.010522374	0.842077152	0.87883694
CACNA1S	0.010506785	0.842308063	0.879008695
WASF1	0.010505304	0.842329999	0.879008695
BEX4	-0.0104908	0.842544849	0.879186831
C1orf190	-0.010485947	0.842616743	0.879212535
CRB2	-0.010483176	0.842657778	0.879212535
GJA3	-0.010475831	0.842766589	0.879279998

C9orf130	-0.010468915	0.842869048	0.879335442
PCTP	0.010462705	0.84296105	0.879335442
C20orf202	0.01046179	0.842974598	0.879335442
SEMA7A	-0.010460322	0.842996347	0.879335442
SPINK7	-0.010447541	0.843185714	0.879485523
PCP4L1	-0.010444649	0.84322855	0.879485523
GPIHBP1	-0.010390588	0.844029591	0.880230953
TCEA2	-0.01039045	0.844031648	0.880230953
OR13C3	0.010339932	0.84478033	0.880965611
FAM75A2	-0.010325292	0.844997324	0.881127225
C16orf73	-0.010323506	0.845023795	0.881127225
IGF2AS	0.010314175	0.845162115	0.881225315
TOMM70A	0.010304854	0.84530028	0.881323233
HLA-DMB	-0.010286535	0.845571839	0.881560212
CFHR2	0.01027977	0.845672124	0.881601401
C7orf72	-0.010276557	0.845719758	0.881601401
HAPLN2	-0.010274912	0.845744151	0.881601401
ARG1	-0.010226554	0.846461131	0.882302597
METRN	0.010156107	0.847505844	0.883345312
FAM111B	0.010142906	0.847701629	0.883503137
MTMR2	0.010105575	0.848255376	0.883975305
ZG16B	0.010101203	0.848320233	0.883975305
FH	0.010101033	0.848322757	0.883975305
CD1B	0.010100396	0.848332214	0.883975305
LEUTX	0.010088885	0.848502969	0.884018841
PSME3	-0.010088665	0.848506229	0.884018841
HADHA	0.010088602	0.848507163	0.884018841
OVCH2	0.010077256	0.848675493	0.884147962
C2orf50	0.010059696	0.848936026	0.884373122
TPSB2	0.010049634	0.849085315	0.884464739
CD36	0.010047781	0.849112797	0.884464739
GPR26	0.010044544	0.849160829	0.884468509
SLMO1	0.010033512	0.84932453	0.884592753
CXCL5	-0.010023531	0.849472627	0.88470073
TFDP3	-0.009966851	0.850313765	0.885487088
PRKCDBP	-0.00996666	0.850316599	0.885487088
CXorf30	-0.009963476	0.850363855	0.885489996
TIMP1	0.009942763	0.850671296	0.885763822
SPATA12	0.009926526	0.850912297	0.885968442
TEPP	-0.009921625	0.85098505	0.885997871
MYPN	-0.009894766	0.851383768	0.886323587
CD200R1L	0.009891635	0.851430248	0.886323587
SLC13A5	-0.009891557	0.851431411	0.886323587
TGM7	-0.009877438	0.851641026	0.886495454
DUPD1	-0.009864662	0.8518307	0.886646547
POTEB	0.0098478	0.852081054	0.886815923

PPP1R2	-0.009847704	0.852082485	0.886815923
C8orf47	-0.009832493	0.852308339	0.887004629
MTHFR	-0.009823404	0.85244331	0.887098736
SLC9A2	-0.009816287	0.852548992	0.887162356
MT1A	0.009805567	0.852708184	0.887266885
SSTR4	0.009801121	0.85277421	0.887266885
NEIL1	-0.009800351	0.852785637	0.887266885
C21orf125	0.009797522	0.852827653	0.887266885
UCP3	0.009772951	0.853192553	0.887600151
MYH7	-0.009768441	0.85325954	0.887623471
LOC283663	-0.009759663	0.85338992	0.887712732
LOC284798	-0.009746929	0.853579055	0.887863099
C6orf58	0.009732635	0.853791373	0.88802728
NR2C1	0.009730298	0.853826079	0.88802728
PIP5KL1	0.009710499	0.854120194	0.88826154
TEX13A	-0.00970913	0.854140521	0.88826154
DDX20	0.009704696	0.854206392	0.888276449
GPR6	0.00970216	0.854244065	0.888276449
HIST1H3H	0.009698921	0.854292186	0.888280107
CHIA	0.009630326	0.855311325	0.889293361
PLA2G10	0.009617164	0.855506909	0.889450279
ENTHD1	-0.009594092	0.855849767	0.889760289
LOC283914	-0.009586939	0.855956076	0.889824359
PRR23B	0.009577342	0.856098695	0.889926168
DIO1	-0.009569802	0.856210766	0.889996211
KLHDC7B	0.009554042	0.856445003	0.890174516
ASB17	-0.009552245	0.8564717	0.890174516
TMEM156	-0.00953644	0.856706622	0.890372215
KCNG3	-0.009526995	0.856847019	0.890461174
OR5AP2	-0.009524665	0.856881644	0.890461174
NFS1	0.009510041	0.857099033	0.890595303
HRK	0.009509966	0.857100155	0.890595303
MAP4K1	-0.009500894	0.857235015	0.890688962
VENTX	-0.00949358	0.857343732	0.890755448
ETNK2	0.009465948	0.857754543	0.891135778
SLAMF1	-0.009461681	0.857817981	0.891155195
RPS6KA4	0.009451632	0.857967385	0.891263913
CCDC96	0.009447332	0.858031314	0.891283832
ZNF385A	0.00943571	0.858204121	0.89141684
STK39	-0.009432304	0.858254764	0.891422949
RSPO2	0.009424541	0.858370194	0.891496345
SLC38A10	-0.009399023	0.858749652	0.891843937
C3orf72	0.009391271	0.858864944	0.891865021
ZBP1	0.009389073	0.858897626	0.891865021
UPF3B	-0.009387485	0.858921238	0.891865021
TCF7L1	-0.009385613	0.858949088	0.891865021

POLN	-0.009372429	0.859145152	0.892001453
C15orf41	0.009370754	0.859170066	0.892001453
AGBL4	-0.00934837	0.859502982	0.892300573
IGSF22	-0.009338898	0.859643859	0.892400305
KRT19	0.009335225	0.85969849	0.892410499
NCRNA0016	-0.009331594	0.859752502	0.89242005
LSM14B	-0.009326927	0.859821915	0.892445585
HNF1B	-0.009318144	0.859952558	0.892534667
ZFP57	-0.009305178	0.860145432	0.892688325
LOC643837	0.009286811	0.860418664	0.892840231
DTX3	-0.009286403	0.860424726	0.892840231
C3orf24	-0.009284784	0.860448817	0.892840231
CXorf41	0.009283284	0.86047113	0.892840231
TM2D3	0.00924984	0.86096869	0.893309965
OLFM4	-0.009211919	0.861532931	0.893848831
XAGE2	0.009203784	0.861653976	0.893927846
C1QC	-0.009199888	0.861711945	0.893941417
FAM83A	-0.009183684	0.861953085	0.894082141
HLA-DPB2	-0.009183012	0.861963083	0.894082141
BTNL3	-0.009181722	0.86198228	0.894082141
ZNF578	-0.009172366	0.86212151	0.894179984
DGCR10	-0.009127652	0.862787	0.894803549
CORIN	-0.009125933	0.862812581	0.894803549
GRIN3B	0.009119775	0.862904246	0.894852013
BCL11A	0.009114114	0.862988511	0.894892799
DEPDC7	-0.009096807	0.863246129	0.895113332
TFAMP1	-0.009083152	0.863449391	0.895277483
CXorf57	0.009069545	0.863651955	0.895440894
CACNA1F	-0.009061793	0.863767367	0.895513932
OPN4	0.009058345	0.863818694	0.895520525
DSPP	0.009052676	0.863903091	0.8955614
PAQR8	-0.009023038	0.864344357	0.895972198
TGIF2LX	-0.009011099	0.864522122	0.896109825
FLYWCH1	-0.008968185	0.865161156	0.896725535
RSPH1	0.008962712	0.865242665	0.896758804
PRAMEF22	0.008959982	0.865283313	0.896758804
ALDH3A2	0.008949526	0.865439036	0.896873518
HMGCR	0.008942837	0.865538643	0.89693007
ZNF30	-0.008938798	0.86559881	0.896945746
MYBL1	-0.008910711	0.86601713	0.897312177
FAM18B2	0.008907451	0.866065684	0.897312177
PEX10	-0.008905979	0.866087606	0.897312177
GPSM2	-0.008898943	0.866192417	0.897374082
MYH8	-0.00887602	0.866533863	0.897681122
SEC24C	0.008872244	0.866590112	0.897692696
VAV1	-0.008866928	0.866669313	0.897724519

OR4F29	0.00886413	0.866710988	0.897724519
RASAL1	0.008849605	0.866927364	0.897901937
ZHX2	-0.008838433	0.8670938	0.898004056
BPNT1	0.008836934	0.867116143	0.898004056
CD3E	0.008831078	0.867203377	0.898047697
C13orf1	0.008820171	0.867365875	0.89816927
TRIM25	-0.008793412	0.86776457	0.898535404
ATP5S	0.008782051	0.867933862	0.898663974
NANP	-0.008771852	0.868085833	0.898774596
FBXL8	0.008763439	0.868211208	0.898857673
SLC1A2	-0.008756448	0.868315373	0.898918784
TCHHL1	0.008748744	0.868430182	0.898990908
CYTH1	-0.008707478	0.869045175	0.899580782
OR10A5	-0.008674965	0.869529785	0.900031375
ASPH	0.008672209	0.86957086	0.900031375
SNAI3	-0.008661	0.869737946	0.90015753
TAS2R8	-0.00864908	0.869915626	0.900294637
KIAA0100	-0.00863044	0.870193498	0.900520603
RNF2	0.008628367	0.870224404	0.900520603
VPS37C	-0.008600023	0.870646967	0.900911064
ID1	0.008579287	0.870956137	0.901184156
AGA	0.00855087	0.871379848	0.901575731
STAU2	0.00854671	0.871441877	0.901593069
KIF6	-0.008540045	0.871541265	0.901607565
FAM164A	-0.008539699	0.871546434	0.901607565
CAPNS2	0.008520644	0.87183058	0.901854665
SOBP	-0.008498328	0.872163389	0.902152074
STAMBPL1	0.008480823	0.872424454	0.902375247
C6orf146	-0.008476482	0.872489198	0.902395346
OR13H1	0.008447102	0.872927411	0.90275761
SCARNA4	-0.008445711	0.872948169	0.90275761
SNORA77	-0.008443882	0.872975449	0.90275761
RCN2	0.008432665	0.873142768	0.902790356
EIF3J	0.008430656	0.87317273	0.902790356
LBP	0.008430154	0.873180211	0.902790356
C22orf33	-0.008429603	0.873188443	0.902790356
NEURL4	-0.008423012	0.873286748	0.902845122
RAB9BP1	-0.008402206	0.873597123	0.903119119
KIF4B	0.008396348	0.873684527	0.903162594
C6orf142	0.008392906	0.873735865	0.903168782
HOXB9	0.008384244	0.873865094	0.903255481
RGN	0.008367036	0.874121821	0.903473949
ALG11	0.008317824	0.874856096	0.904185955
KLB	0.008303103	0.875075775	0.904366065
CARD17	-0.008274202	0.875507069	0.904764843
TRIM50	-0.008268476	0.87559252	0.904771779

PIM3	-0.008267663	0.875604645	0.904771779
SMARCA1	-0.008236952	0.876063008	0.905198444
SNORA68	-0.008233438	0.87611545	0.905205665
MAP2K3	-0.008227948	0.876197395	0.905243366
FBXO16	0.008208537	0.876487124	0.90547958
ASPHD2	-0.008205164	0.876537469	0.90547958
KRTAP13-2	0.008203492	0.876562431	0.90547958
ZNF598	0.008172496	0.877025129	0.905910552
CCNYL1	-0.008156462	0.877264494	0.906110803
AMDHD1	-0.008106094	0.878016478	0.906840481
TNNT3	0.008089806	0.878259685	0.907044631
TMPRSS3	-0.00807554	0.878472702	0.907217582
XK	0.008061885	0.878676609	0.907381108
FIBCD1	0.008052422	0.878817924	0.907479984
KLHL38	0.008038616	0.8790241	0.907645821
NCRNA0011	0.00803467	0.87908302	0.907647438
C16orf63	-0.008032407	0.879116818	0.907647438
SPHKAP	0.008012802	0.879409623	0.907902677
RNF40	0.007938232	0.880523443	0.909005462
BCORL2	0.007910175	0.880942589	0.909391025
C19orf29	-0.007898521	0.881116692	0.909523605
VTI1A	-0.007885682	0.881308501	0.909674448
ADAM8	0.007862622	0.881653052	0.909982925
HOXD4	0.007856047	0.881751284	0.910037148
LGALS3BP	0.007850021	0.881841328	0.910082917
MDFI	0.007845607	0.881907285	0.910103823
CYorf15A	0.00783021	0.882137343	0.910294066
NCAN	-0.007758655	0.883206687	0.91135032
SBF1P1	-0.007676258	0.884438308	0.912573903
CD209	-0.00764009	0.884979015	0.913084503
FLJ42289	0.007629223	0.8851415	0.913204837
OR51G2	0.007596326	0.885633357	0.913664956
C20orf166	0.007590388	0.885722147	0.913709223
ROBO1	-0.007574272	0.885963129	0.913910479
PRKY	0.007567664	0.886061939	0.913965066
PRSS1	-0.007557091	0.886220043	0.914080804
SNORD10	0.007527496	0.886662636	0.914458138
TBPL1	0.007525222	0.886696645	0.914458138
KIF20B	0.007523417	0.886723631	0.914458138
OR11H12	-0.007505105	0.886997502	0.914693209
SSTR5	0.007464347	0.887607111	0.915274459
EIF2S1	-0.007458416	0.887695835	0.915318554
C1orf65	0.007455239	0.887743349	0.915320155
C3orf18	-0.007438517	0.887993484	0.91553066
FBXO15	0.007431908	0.888092358	0.91555634
ELF5	-0.007430706	0.888110338	0.91555634

ONECUT2	-0.007412446	0.888383486	0.915790523
C1GALT1	-0.007397745	0.888603413	0.915969822
C14orf128	0.00736789	0.889050083	0.916382817
CACNG5	-0.007357656	0.8892032	0.916471914
ARHGAP19	-0.007355961	0.889228561	0.916471914
FAM53B	0.007342567	0.88942896	0.916631015
DOK3	0.007334713	0.889546475	0.916680716
CRYGN	-0.007333191	0.889569245	0.916680716
OR10A4	0.007308894	0.889932822	0.917007924
PLAUR	-0.007302956	0.890021674	0.91705203
CLEC4E	-0.007272151	0.890482664	0.917479552
CCDC109A	0.007263586	0.890610838	0.91756414
ACOXL	-0.007256155	0.890722047	0.917566015
KRTAP4-2	-0.007255496	0.89073191	0.917566015
SNRNP48	-0.007254229	0.89075088	0.917566015
CASP5	0.007226041	0.891172746	0.917941727
KDM5D	0.007223699	0.891207798	0.917941727
CDK6	-0.007206352	0.891467446	0.918161677
MST1P2	0.007199176	0.891574861	0.918224821
LOXL4	-0.007188093	0.89174075	0.918348177
TTC23	0.007168823	0.892029196	0.918597726
TCEAL1	-0.007156141	0.892219043	0.918745721
C17orf74	-0.007138823	0.892478299	0.918965168
ACACA	-0.007111134	0.892892826	0.919344464
OR4Q3	0.007096457	0.893112568	0.919458445
ACRC	0.007095413	0.893128195	0.919458445
NECAB2	-0.007094489	0.893142035	0.919458445
DMBX1	-0.007071935	0.893479723	0.919758539
KCNRG	-0.007048954	0.89382383	0.920051264
ST7OT2	0.007046143	0.893865914	0.920051264
TPD52L1	0.007041016	0.893942686	0.920051264
C9orf21	0.007040603	0.893948881	0.920051264
DPEP2	0.006992568	0.8946682	0.920744003
ARRDC5	0.006971187	0.894988403	0.921025943
UBE2L6	0.00695547	0.895223808	0.921220593
FAM117B	-0.006949768	0.895309209	0.92126087
SLC2A5	0.006946566	0.895357169	0.921262621
GPR137	0.006942218	0.89542228	0.921282017
DUSP5P	0.006934522	0.895537562	0.921353028
GOLGA8G	0.006929124	0.895618407	0.921388603
PACS1	-0.006920011	0.895754906	0.921448012
KRTAP10-10	0.006919091	0.895768692	0.921448012
RDH14	0.006903152	0.896007431	0.921645989
INSL5	-0.006891861	0.896176568	0.921772356
FAM181B	-0.006876416	0.896407944	0.921962723
PTP4A2	0.006865632	0.89656949	0.922081254

CSN3	0.006860145	0.896651681	0.922118163
HSF2	-0.006845027	0.896878177	0.922303464
THNSL2	0.006840967	0.896938996	0.922318382
ASXL1	0.006824572	0.89718463	0.922523332
DMRT1	-0.006816753	0.897301768	0.922596143
SLC22A4	-0.006813629	0.897348579	0.922596642
ZNF880	-0.006804589	0.897484015	0.922624992
ZFAND3	-0.006802927	0.897508916	0.922624992
DEFB125	-0.006802512	0.897515138	0.922624992
KLRG2	0.006785807	0.897765432	0.922834654
THSD1P1	0.006774265	0.897938381	0.922964792
TRIB1	-0.006770672	0.897992216	0.92297249
FAHD2A	0.006759064	0.898166151	0.923103623
PRH1	0.006738763	0.898470358	0.923368623
TMCO5A	-0.00673471	0.898531097	0.923383394
PATE3	0.006724379	0.898685918	0.923494843
CARS	0.006703053	0.899005504	0.923736349
SLC25A21	-0.006702506	0.899013704	0.923736349
DISC2	0.006696402	0.899105181	0.92378268
SCARNA6	0.006674727	0.899430031	0.924068771
CSMD3	0.006654979	0.899726009	0.924325173
CEL	0.006635852	0.900012699	0.924572006
FLJ42875	-0.006626951	0.900146111	0.924661361
TCP11	-0.006618511	0.900272625	0.924743621
ZNF610	0.006590981	0.900685297	0.925119794
CCNO	0.006581249	0.900831186	0.925221922
CHPT1	-0.006568177	0.901027152	0.92537547
RGS7	-0.006561546	0.90112656	0.925424634
PROP1	0.006558785	0.90116796	0.925424634
CRYL1	-0.006545045	0.901373944	0.925588434
SPRYD3	0.006520805	0.901737368	0.92591388
AADACL2	-0.006445368	0.902868491	0.927027531
SMOC1	-0.00643458	0.903030266	0.927145835
VPS24	-0.006429242	0.903110309	0.927156907
TAS1R1	-0.006427652	0.903134162	0.927156907
C20orf114	-0.006407179	0.903441182	0.927424286
C2orf72	0.00640162	0.903524551	0.927451065
LOC494141	0.006399229	0.90356041	0.927451065
DUSP14	-0.006378512	0.903871118	0.927722173
CCDC60	-0.006363328	0.904098853	0.927908094
GHSR	-0.006322883	0.904705493	0.92848286
BRD7	0.006301152	0.905031451	0.928723193
RPSAP52	0.006301053	0.905032941	0.928723193
LMO1	-0.006297063	0.905092796	0.928736759
CABLES2	0.006283351	0.905298488	0.928899964
ACBD7	0.0062782	0.905375762	0.928931391

HSPB3	-0.006261416	0.905627542	0.929141853
PTENP1	-0.006244636	0.905879277	0.929352246
MPHOSPH1	-0.006238083	0.905977593	0.929369702
C3orf27	-0.006237281	0.905989626	0.929369702
LILRA5	0.006233285	0.906049571	0.929383323
MSH6	0.006221691	0.906223517	0.929513872
IL9	0.006193923	0.906640141	0.929814206
FES	-0.006192942	0.906654857	0.929814206
LRPPRC	-0.00619284	0.906656393	0.929814206
UBL4B	0.006186785	0.906747249	0.929859498
ANG	0.006179707	0.906853443	0.929920514
HOXC12	-0.006170255	0.906995272	0.930018065
AKAP8	0.006149056	0.907313359	0.930296326
ASB18	0.00614176	0.907422843	0.930360684
AXIN1	-0.006094728	0.908128643	0.931036392
CLRN1	-0.00608795	0.908230366	0.931092748
IRX1	0.006048252	0.908826159	0.93165558
PENK	-0.006035514	0.909017338	0.931803597
C20orf26	0.006014287	0.909335958	0.932082228
LOC144486	-0.005979065	0.909864653	0.932576148
CLEC2A	-0.005966968	0.910046249	0.932699647
RELL2	0.005963258	0.910101933	0.932699647
DBNDD1	0.005960021	0.910150536	0.932699647
CT47A7	0.005958559	0.91017248	0.932699647
ST7OT4	-0.005949297	0.910311515	0.932794125
HBZ	-0.00593595	0.910511889	0.932951444
LILRB4	-0.005924826	0.910678886	0.933036856
SDHC	0.005924156	0.910688949	0.933036856
C17orf107	-0.005919789	0.910754506	0.933056021
HIGD2B	-0.005898168	0.911079115	0.933340564
SLC6A3	0.00588748	0.911239583	0.933456936
KRTAP5-2	0.005882005	0.911321779	0.933489436
WDR45L	-0.005879123	0.911365057	0.933489436
CHKB-CPT1	0.005863288	0.911602815	0.933640968
FLJ46111	-0.005863025	0.91160676	0.933640968
CD163L1	-0.005830827	0.912090219	0.934088076
ASCL3	0.005764434	0.913087238	0.935045139
NOLC1	-0.005762343	0.913118647	0.935045139
NUDT6	-0.005755684	0.913218639	0.93509945
ARHGAP8	0.005750887	0.913290687	0.935125143
LMO3	-0.005738892	0.913470831	0.935261509
C6orf124	-0.00573361	0.913550163	0.935294648
NOBOX	0.005712843	0.913862064	0.935565877
NDP	0.005688773	0.914223599	0.935864694
GLUL	0.005687153	0.914247935	0.935864694
APC2	-0.005667825	0.914538239	0.936113746

GRK4	-0.005655567	0.914722374	0.936254103
FAM57A	0.005648766	0.914824526	0.936310537
PLEKHG4	-0.005628981	0.915121737	0.936495458
UIMC1	-0.005628627	0.915127058	0.936495458
ANKRD23	0.00562536	0.915176131	0.936495458
OPALIN	-0.005622926	0.915212701	0.936495458
HEPACAM	0.005621087	0.915240326	0.936495458
PDZD9	-0.005599535	0.91556409	0.936778609
POU5F2	-0.005593663	0.915652306	0.936820738
YIPF4	0.005585039	0.915781867	0.936905162
SNTG1	0.005570107	0.916006192	0.937086522
JMJD7-PLA	-0.005563472	0.916105876	0.93714036
USH1C	-0.00552954	0.916615694	0.937613722
BAIAP3	0.005526297	0.916664421	0.937615406
KDELR3	-0.005522239	0.916725398	0.937629618
DEFB103B	-0.005510412	0.916903092	0.937763202
BPI	0.005454325	0.917745884	0.938543042
LIMS3	-0.005453396	0.91775984	0.938543042
CETN4P	-0.005428939	0.918127368	0.938870682
OFD1	-0.00541988	0.918263511	0.938961687
PMS2	0.005378626	0.918883515	0.939513616
CNTN5	0.005377687	0.918897626	0.939513616
CGB5	-0.005369192	0.9190253	0.939595916
TMEM88	0.005339962	0.919464631	0.939996823
ARG2	-0.005323042	0.919718945	0.940208552
OR13J1	0.005318763	0.919783272	0.940226048
COL28A1	-0.005266152	0.920574106	0.940901254
IFNA4	-0.005266075	0.920575271	0.940901254
CDH24	-0.005265392	0.920585535	0.940901254
ZNF683	0.005242524	0.920929304	0.941201285
C20orf117	0.005239577	0.92097361	0.941201285
NCRNA0020	0.005200024	0.921568243	0.941760651
RELA	0.005174034	0.921959008	0.942111635
POLR3D	-0.005156447	0.922223432	0.942333486
PAPOLB	0.005151733	0.922294305	0.942357553
C16orf82	-0.005139374	0.922480135	0.942499071
C2orf65	0.005110447	0.922915104	0.942895106
SNORA29	0.005078234	0.923399502	0.943341596
MRGPRX4	0.00507269	0.923482875	0.943378377
LIPJ	0.005049243	0.923835482	0.943690174
TCL1A	-0.005042854	0.92393156	0.943739909
FAM101A	0.005036929	0.924020669	0.943740769
COPG2	-0.005036496	0.924027178	0.943740769
KCTD8	0.005023451	0.924223363	0.943892732
BTG3	0.005010343	0.924420495	0.944045647
LOC100190	-0.005001658	0.924551128	0.944130639

MAST3	0.004988468	0.924749509	0.944284801
CCDC142	0.004962503	0.92514003	0.944635136
LOC284440	-0.004958489	0.92520041	0.944648355
FAM35B	-0.004954088	0.925266604	0.944667509
PHYHIP	-0.00494349	0.925426015	0.944781826
OR4M1	-0.004935225	0.925550324	0.944860298
SLC48A1	0.004904409	0.926013873	0.945285062
IRF1	-0.004878419	0.926404832	0.945635685
POU3F3	0.004875044	0.926455605	0.945639043
STAG3	-0.0048629	0.926638281	0.945777028
NCF1	0.004854231	0.926768702	0.945861667
MMP13	0.004836289	0.927038616	0.946046838
C16orf89	-0.004835855	0.927045144	0.946046838
ASB4	0.004787118	0.927778399	0.946732978
SPTLC3	-0.004784846	0.92781258	0.946732978
ZNF451	-0.004776782	0.927933903	0.946808263
CLDN5	0.004735888	0.928549211	0.947387547
GML	-0.004724354	0.928722769	0.947516084
OR6B3	0.004717934	0.928819376	0.947566102
GDPD5	-0.004692351	0.929204326	0.947910263
HOTAIR	-0.004678581	0.929411547	0.948073091
LOC100192	-0.004666358	0.929595488	0.948212156
C3orf55	0.004656733	0.929740328	0.948307573
ZNF350	0.004653813	0.929784267	0.948307573
TRYX3	0.00464286	0.929949097	0.948386664
FLJ43859	-0.004642332	0.929957056	0.948386664
ST14	-0.004630627	0.930133195	0.948475022
ELMO3	-0.004630245	0.93013895	0.948475022
LECT2	0.004624674	0.93022279	0.948511948
LOC96610	0.004609535	0.930450639	0.948695703
LOC100216	0.004566095	0.931104422	0.949313703
RASAL3	0.004552604	0.93130749	0.949472134
PRMT6	-0.004543418	0.931445751	0.94956448
DCDC2	-0.004524898	0.931724505	0.949800037
KRTAP5-4	0.004516567	0.931849904	0.949879247
SHC3	-0.00450176	0.932072792	0.950057818
EGF	0.004496145	0.932157321	0.950092707
NCRNA0017	-0.004493147	0.932202435	0.950092707
C19orf54	-0.004485402	0.932319028	0.950162911
PCIF1	0.004459466	0.932709445	0.950512158
PNP	-0.004455914	0.932762922	0.950518016
HOXD12	0.00444613	0.932910207	0.950525035
ZNRF3	-0.004443075	0.932956195	0.950525035
TNFSF4	-0.004442387	0.932966552	0.950525035
KCNA10	0.004440871	0.932989374	0.950525035
TNFRSF10C	-0.004439604	0.933008455	0.950525035

PAPL	-0.004426881	0.933199985	0.950645574
ALPK2	-0.004425403	0.933222243	0.950645574
HNRNPH3	0.004405563	0.93352092	0.950901188
PNO1	-0.004374173	0.933993515	0.951305867
PTGER1	0.00437283	0.934013739	0.951305867
AVPR1B	0.004367453	0.934094687	0.951339658
SLC35E4	0.004362016	0.93417655	0.95137438
WIPI1	-0.004339665	0.934513064	0.951501505
KRT85	0.004339032	0.9345226	0.951501505
C14orf176	0.004335894	0.934569851	0.951501505
GP2	0.004335621	0.934573957	0.951501505
C6orf103	0.004333483	0.93460615	0.951501505
ZNRF4	-0.004332313	0.934623763	0.951501505
OSCP1	0.004331512	0.934635824	0.951501505
SERPINC1	-0.00430702	0.935004603	0.951828281
ZNF229	-0.004302387	0.935074358	0.951850636
UNC93A	0.004286945	0.935306876	0.952038662
OSGEPL1	0.004283079	0.935365095	0.952049262
LOC284100	-0.004273195	0.935513929	0.952131413
MAP2K6	-0.004270192	0.935559152	0.952131413
UNC5D	-0.004268194	0.935589235	0.952131413
FAM197Y2	0.004250517	0.935855426	0.952353643
PRG2	0.004243331	0.935963639	0.952415097
HAS2AS	-0.004224812	0.936242516	0.9526502
SH3RF2	-0.004205689	0.936530489	0.952894533
OR2J3	-0.004193333	0.936716576	0.952985652
LRRC50	0.004191974	0.936737034	0.952985652
TRAF5	0.00419021	0.936763601	0.952985652
ZNF204P	-0.004167207	0.937110031	0.953289383
FAM9A	-0.004162457	0.937181563	0.953313455
TCEAL5	-0.004136225	0.937576649	0.95366663
ARL17A	0.004131456	0.937648479	0.953690984
C4orf39	-0.004116283	0.937877	0.953836323
ARHGDIB	-0.004115608	0.937887165	0.953836323
CNKSR1	0.00408817	0.938300437	0.954207895
PRG1	-0.004083021	0.938377996	0.954238041
RFC3	0.004075703	0.938488221	0.9543014
MYL2	0.004055858	0.938787132	0.954556608
LRRC14B	0.004023623	0.939272701	0.955001574
WDR87	0.004009062	0.939492043	0.955175824
PCDHB1	-0.004004212	0.939565113	0.955201349
IL26	0.003996572	0.939680198	0.955226496
HCCS	0.003996202	0.939685779	0.955226496
KRT14	0.003981666	0.939904742	0.955378829
GRASP	-0.003979885	0.939931579	0.955378829
SLC20A1	0.003950065	0.940380807	0.955786658

OR5B21	-0.003927694	0.940717839	0.956065202
MICB	0.00392224	0.940800003	0.956065202
C1orf68	0.003921621	0.940809333	0.956065202
HSD17B7P2	0.003919128	0.940846891	0.956065202
C20orf144	-0.003909759	0.940988043	0.956159849
GORASP2	0.003899466	0.941143126	0.95626864
CPNE6	-0.003862789	0.941695727	0.956742982
BSX	-0.003862104	0.941706047	0.956742982
SLURP1	-0.003849875	0.941890299	0.956801615
WAS	-0.003849116	0.941901733	0.956801615
KCNH8	0.003848708	0.941907891	0.956801615
CCDC110	0.003837115	0.942082565	0.956930239
SP8	-0.003833392	0.942138664	0.95693329
CHRNA5	-0.003830538	0.94218167	0.95693329
CORO6	0.003800694	0.942631355	0.957341192
FOXD3	0.003735465	0.943614294	0.9582906
MAT1A	0.003730626	0.943687214	0.958315785
TPSAB1	-0.003720916	0.943833534	0.958415502
C20orf173	0.003701541	0.94412552	0.958663118
SLC4A1AP	-0.003649073	0.944916258	0.959417114
TNFRSF21	-0.003634148	0.945141208	0.95955126
GALNT13	-0.003633913	0.945144741	0.95955126
FAM131A	0.003616447	0.945407985	0.959769589
HAVCR1	0.003600536	0.945647797	0.959964109
LOC442308	-0.003592045	0.945775782	0.960045094
TTC9B	0.003587013	0.945851622	0.960069264
ULK4	0.003584068	0.945896009	0.960069264
LOC643763	-0.003568838	0.94612558	0.960253334
GPR151	0.003563285	0.946209279	0.960253591
HEATR2	0.003562423	0.946222268	0.960253591
CYMP	-0.003539357	0.946569943	0.960557473
TFPI	-0.003534119	0.946648897	0.960588647
HRASLS	-0.003529201	0.94672304	0.960594675
TYMP	0.003527325	0.946751308	0.960594675
LOC100192	0.003501826	0.94713568	0.960935711
HEPACAM2	-0.003484181	0.947401672	0.961156612
ZNF252	-0.003474718	0.947544325	0.961252367
SLC6A13	-0.003466585	0.94766693	0.961327777
SNORA28	0.003458472	0.947789227	0.961402865
MAP7D2	-0.003446778	0.94796551	0.961532706
EDDM3B	0.003425457	0.948286943	0.961809752
SRPK3	0.00339404	0.948760585	0.962241141
CIB4	-0.003368456	0.949146301	0.962583316
STX3	-0.00335294	0.949380229	0.962742416
KATNB1	-0.003351637	0.949399864	0.962742416
RNF112	0.003343431	0.949523586	0.96281885

HTR3C	-0.003336356	0.949630264	0.962877996
SYCP2L	-0.003299509	0.950185825	0.963392257
PARN	-0.003286182	0.950386778	0.963546947
SPATA8	0.003279157	0.950492701	0.963605281
PRSS42	-0.003252731	0.950891161	0.963960167
44078	0.003209508	0.951542919	0.964571782
CCDC81	-0.003156633	0.952340269	0.965330914
TNFRSF8	-0.003153065	0.952394089	0.965336333
APOL5	-0.00312323	0.952844013	0.965743219
RBMXL2	0.003118496	0.952915402	0.965766424
GBX1	0.003106433	0.95309733	0.965879812
SLC30A2	0.0031024	0.953158148	0.965879812
CRISP3	0.00310143	0.953172781	0.965879812
CD48	0.003090014	0.95334495	0.966005122
FRG2C	-0.003080749	0.953484679	0.966097551
C1orf14	-0.003076836	0.953543705	0.966108206
CDH12	0.003055695	0.953862539	0.966382076
LOC730101	-0.00301479	0.954479496	0.966957939
MYH7B	0.003006786	0.954600221	0.967031049
GPC2	0.002991049	0.954837573	0.967222292
PTTG1IP	-0.002986127	0.954911809	0.967248293
SNORA44	0.002945902	0.955518556	0.967786646
SNORA81	0.002944448	0.955540489	0.967786646
GREB1	0.00287556	0.956579614	0.96878982
CNBD1	0.002868688	0.956683276	0.968845535
LGALS3	0.002856173	0.956872066	0.96898745
RHOBTB1	-0.002842368	0.95708033	0.969149071
C8orf12	0.002838536	0.957138133	0.969158325
DLX5	-0.002829609	0.957272801	0.969245403
NLRP13	0.002808465	0.957591777	0.969495603
ICK	0.002806775	0.957617274	0.969495603
CD79A	-0.00279252	0.957832318	0.96966402
DERA	0.002778386	0.958045554	0.969830591
C3orf21	-0.002759112	0.958336334	0.970075637
PCNXL3	-0.002740804	0.958612538	0.970305906
KLRC1	-0.00272897	0.958791086	0.970437309
RAB40C	0.002718547	0.958948336	0.970547142
ZKSCAN2	0.002709752	0.959081025	0.970632107
CABP4	-0.002699156	0.9592409	0.97067004
ESCO2	0.002694392	0.959312769	0.97067004
PDHA1	0.002694058	0.959317811	0.97067004
LRP11	0.002691553	0.959355609	0.97067004
ZC4H2	0.002691116	0.95936221	0.97067004
FUT1	-0.002676001	0.959590253	0.970851447
ODF4	-0.002668457	0.959704077	0.970917282
ADAM3A	0.002660323	0.959826811	0.970992123

UGT8	0.002652855	0.95993948	0.971056776
NELF	0.002645671	0.960047883	0.971117106
RAB3D	-0.002619922	0.960436401	0.971460761
ASS1	-0.002614221	0.960522421	0.971498426
QPCT	0.002579502	0.961046299	0.97192955
LOC151534	0.002573905	0.96113075	0.97192955
RTP1	-0.00257307	0.961143339	0.97192955
CHAC1	0.002573034	0.96114389	0.97192955
TSIX	0.002552503	0.961453696	0.972188207
TMEM59	-0.002549612	0.961497309	0.972188207
LAIR1	0.002542505	0.961604555	0.972247282
POLA1	-0.002527646	0.961828787	0.972424626
SEPT7L	-0.002521878	0.961915822	0.972463251
TDRD3	-0.002511755	0.962068575	0.972527
C2orf70	-0.002511227	0.962076547	0.972527
KRTAP1-3	0.002500253	0.962242153	0.972644676
LHFPL2	0.00249704	0.962290638	0.972644676
LYSMD2	-0.002485533	0.962464282	0.972766059
LYNX1	-0.002482608	0.96250842	0.972766059
ZNF879	-0.002461483	0.962827209	0.972987414
HGD	-0.002456914	0.962896158	0.972987414
MAEL	0.002456202	0.96290691	0.972987414
TGIF1	0.002455144	0.962922869	0.972987414
C7orf25	0.002427732	0.963336547	0.973356029
TYW3	-0.002419998	0.963453264	0.973424572
MYOD1	0.002413523	0.963550986	0.973436552
CD8A	-0.002412735	0.96356288	0.973436552
FAM3D	-0.002405497	0.963672116	0.973497524
IPW	0.002380132	0.964054921	0.973834834
SYCP3	0.002341374	0.964639853	0.974376276
ATP6V1B1	0.00232735	0.964851517	0.974540648
POMT2	0.002291043	0.965399491	0.975044673
DSP	0.002279413	0.965575018	0.97514113
NCF2	0.002278227	0.965592924	0.97514113
QSOX1	0.002263203	0.965819685	0.975320675
TRPM1	0.002243877	0.966111383	0.975565774
GUCA1A	-0.002227489	0.966358736	0.975747941
OR13A1	-0.002225433	0.966389776	0.975747941
TPSD1	0.002213526	0.966569499	0.975879927
C6orf10	-0.002200767	0.966762083	0.976024885
RGS13	-0.002195667	0.966839072	0.976046681
EPHB4	-0.002189958	0.966925245	0.976046681
FAM69C	-0.002188023	0.966954453	0.976046681
GSDMA	0.002186349	0.966979714	0.976046681
TRIML2	0.002170505	0.967218868	0.976238597
IMMP2L	0.002149829	0.967530971	0.976503335

C10orf25	0.002146632	0.967579227	0.976503335
CAP2	-0.002137668	0.96771454	0.976590405
UBE2D1	-0.002130535	0.967822206	0.976649569
ERN2	0.002118215	0.968008181	0.976783028
KIR2DS4	0.002115275	0.968052555	0.976783028
FFAR1	-0.002110993	0.968117203	0.976798769
LOC284233	-0.00210553	0.968199667	0.976832483
ATP6V0A4	0.002099631	0.968288704	0.976872824
AGPAT6	-0.002086089	0.968493133	0.977000562
TP73	-0.00208281	0.968542622	0.977000562
FRAS1	-0.00207626	0.968641502	0.977000562
TTTY6	0.002075237	0.968656955	0.977000562
PDZK1IP1	-0.002074994	0.968660612	0.977000562
C21orf90	0.002069076	0.968749946	0.977041182
PHF11	0.002052613	0.968998474	0.977206651
GUSBP3	-0.002051707	0.969012149	0.977206651
PLTP	-0.002045713	0.969102643	0.977248424
CEBPE	-0.002017851	0.969523243	0.977623058
C17orf100	-0.002013333	0.969591447	0.977642331
EID3	0.002002814	0.969750246	0.977752944
TRAF3IP3	0.001981012	0.970079387	0.977998477
SPATS1	-0.001980177	0.970091987	0.977998477
CYBASC3	-0.00195705	0.970441129	0.97830094
SCNN1D	0.001952153	0.970515066	0.978325953
CD1A	-0.001944548	0.970629878	0.978392166
GTF2H1	0.001940962	0.970684009	0.978397208
ROPN1L	0.001934319	0.970784307	0.978448781
OR10H2	0.001907461	0.97118979	0.978807929
GMPR2	-0.001879799	0.971607411	0.979179272
PCDH15	-0.001875523	0.971671976	0.979194788
C14orf53	-0.001859796	0.971909409	0.9793845
GCDH	-0.001855872	0.971968661	0.979394651
TMEM139	-0.001826908	0.972405959	0.979758345
LOC90110	0.001825449	0.972427992	0.979758345
ASPG	0.001811135	0.972644112	0.979849808
GLT25D2	-0.00180844	0.972684801	0.979849808
HSPH1	0.001806488	0.972714274	0.979849808
EPCAM	-0.001806401	0.972715576	0.979849808
LOC151009	-0.001780898	0.973100647	0.980173827
MT1M	0.001777675	0.973149302	0.980173827
CD247	-0.001775318	0.97318489	0.980173827
IL17D	-0.001769833	0.973267702	0.980207661
PAX7	-0.001755066	0.973490674	0.980382643
CEACAM22	-0.001747323	0.973607588	0.980450805
CELF6	-0.001742143	0.973685803	0.98047999
SAMD11	-0.001737985	0.973748572	0.980493619

MDH1	0.001713906	0.974112145	0.98081012
C2orf51	-0.001696037	0.974381966	0.981032197
ADCK4	0.001632639	0.975339253	0.981946374
FAM180B	0.001620574	0.975521441	0.982080149
NEFH	-0.001605999	0.975741532	0.982252066
KIAA0125	-0.001586673	0.976033348	0.982482794
CDC42EP5	0.001584286	0.976069399	0.982482794
PPAP2A	0.001580618	0.976124786	0.982488887
TULP2	-0.001543312	0.976688134	0.983006226
LCE1D	-0.001520929	0.977026135	0.983296719
C8orf41	0.00151194	0.977161882	0.983340209
FAM63A	0.001511528	0.977168101	0.983340209
SUMF1	-0.001507486	0.977229138	0.983351942
LRRC10	-0.001499746	0.977346018	0.983419864
PTCHD3	-0.001495964	0.977403136	0.983427649
ZNF207	0.001467863	0.977827502	0.983804926
MOCS2	0.0014603	0.977941707	0.983852503
PYDC1	-0.001458189	0.977973595	0.983852503
NTNG2	-0.001423565	0.978496469	0.984328796
THUMPD3	-0.00140001	0.978852193	0.984636904
FDPSL2A	-0.001395567	0.978919296	0.984654668
RGS17	-0.001390513	0.978995618	0.984681703
RHAG	0.001366532	0.979357777	0.984958937
ARPM1	0.001365712	0.979370167	0.984958937
CALCB	0.001361186	0.979438523	0.984970972
MPP7	-0.001353469	0.979555067	0.984970972
MMP27	-0.001351701	0.979581759	0.984970972
AREG	-0.001351512	0.97958461	0.984970972
ANKRD34B	-0.001348545	0.979629427	0.984970972
FGF10	-0.0013405	0.979750922	0.985043398
OR8B8	0.001299353	0.980372345	0.985615259
LRTM1	-0.001296284	0.980418694	0.985615259
GLT1D1	-0.001276527	0.980717084	0.985857451
VASN	-0.001273777	0.980758615	0.985857451
PLCD1	-0.001267355	0.9808556	0.985905177
KRT75	0.001259445	0.980975076	0.98593164
C15orf38	-0.001259056	0.980980941	0.98593164
CTNNA2	-0.001228451	0.981443179	0.986346432
SETD4	0.001221333	0.981550686	0.986398814
EDDM3A	0.001218441	0.981594361	0.986398814
HNRNPCL1	-0.001192077	0.981992536	0.986749148
MTHFS	0.001171046	0.982310187	0.987018536
C11orf70	-0.001166579	0.982377649	0.98703652
TDH	-0.001152433	0.982591308	0.987201387
PRDM15	-0.00113895	0.982794947	0.987356171
FAM167B	0.001134569	0.982861118	0.987372839

UGT1A7	-0.001126676	0.98298033	0.987442788
FAM35A	-0.00111461	0.983162577	0.987576048
TAAR9	0.001104856	0.9833099	0.987674215
CD300A	0.001087556	0.983571195	0.987867233
TMEM212	0.001085565	0.983601274	0.987867233
GLRA3	0.001068884	0.983853225	0.988070448
C1orf116	-0.001056342	0.984042657	0.988210858
OR2AK2	0.001052115	0.984106507	0.988215511
C17orf66	0.001049465	0.984146535	0.988215511
TTC16	-0.001041794	0.984262397	0.988282022
ODF1	-0.001036618	0.984340571	0.988310687
CELA2B	-0.001024686	0.984520801	0.988374639
BATF2	0.00102263	0.984551847	0.988374639
INTS7	-0.001022544	0.984553155	0.988374639
C22orf24	-0.000970341	0.985341658	0.989105319
PNPO	-0.00096778	0.985380343	0.989105319
CCL3L3	-0.000948275	0.985674963	0.989351186
FAM9C	0.000926735	0.986000315	0.989627874
ATP6V0D2	0.000886899	0.986602032	0.990181902
PHF15	0.000872513	0.986819335	0.990350083
AEN	0.000866496	0.986910227	0.990391391
LOC339788	0.000852659	0.987119239	0.99050915
POTEA	-0.000852142	0.987127045	0.99050915
C1orf146	0.000841025	0.987294976	0.990607656
PCNAP1	0.000839057	0.987324698	0.990607656
ANO2	-0.000818949	0.987628447	0.990862494
LOC441455	0.000806368	0.987818491	0.991003236
C3orf30	0.000790623	0.988056322	0.991191903
LOC391322	0.000784224	0.988152976	0.991238932
ZNF645	-0.000753168	0.988622109	0.99165958
TMEM170B	0.000742273	0.988786677	0.991734129
ADORA2B	0.000739343	0.988830939	0.991734129
C22orf34	0.000735433	0.988890003	0.991734129
OSBP2	-0.000735061	0.988895623	0.991734129
ZMYND15	0.000711033	0.989258587	0.992048178
LELP1	0.00068775	0.989610288	0.992350901
PDZK1P1	0.00068271	0.989686424	0.992377278
LCT	-0.000662553	0.989990917	0.992632621
NLRP4	0.000651655	0.99015555	0.992747711
MUCL1	-0.00063972	0.990335841	0.992878487
BCAT1	-0.000627126	0.990526089	0.993017484
TSPAN15	0.000620548	0.990625454	0.993017484
DNER	-0.000619336	0.990643766	0.993017484
C11orf92	0.000616654	0.990684271	0.993017484
VWC2L	-0.000614038	0.990723796	0.993017484
ZNF552	0.000587386	0.991126399	0.993371024

STAMBP	0.000583108	0.991191037	0.993385813
SI	0.00056897	0.991404605	0.993514105
ZNF213	-0.000568029	0.99141882	0.993514105
SPINT1	0.000562368	0.991504344	0.993549814
GSTZ1	0.000550898	0.991677602	0.993602532
SLC6A16	-0.000549351	0.991700975	0.993602532
ZNF613	0.000547975	0.991721762	0.993602532
IDO1	-0.000545674	0.991756522	0.993602532
PF4V1	0.000524256	0.992080068	0.993876681
IAPP	-0.000497982	0.992476977	0.994224296
OR5M10	-0.000466463	0.992953128	0.994640994
C13orf29	-0.000463835	0.992992832	0.994640994
FAM75A6	0.000441985	0.993322905	0.994921574
C6orf164	-0.000420282	0.993650766	0.995190873
HELT	-0.000417572	0.993691715	0.995190873
RFPL1S	0.000413275	0.993756625	0.995205832
C20orf103	0.000403219	0.993908532	0.99530791
CRYZL1	-0.000371282	0.994391011	0.995740998
SELPLG	-0.000351412	0.994691183	0.995991499
SLC18A3	0.000338418	0.994887483	0.99613797
C14orf169	0.000315026	0.995240865	0.996441698
TXNDC3	0.000305686	0.99538195	0.996532854
C7orf16	-0.000293888	0.995560182	0.996661189
SCYL1	0.000277221	0.995811977	0.99686315
KRT4	0.000269866	0.995923088	0.996924268
CNTNAP5	0.000244818	0.996301496	0.997248435
VHLL	0.0002418	0.996347081	0.997248435
LOC202781	0.000228715	0.996544766	0.997389776
PGRMC2	-0.000223165	0.996628599	0.997389776
C1orf100	0.000221095	0.996659874	0.997389776
LOC253724	0.000219192	0.996688623	0.997389776
WASF2	-0.000210888	0.99681407	0.99746519
FLJ39739	-0.000178588	0.997302031	0.997903329
GABRA4	-0.000175254	0.997352396	0.997903586
CCNJL	0.000161626	0.99755829	0.998059449
ZNF277	0.000151432	0.997712283	0.998163374
ZNF671	0.000143841	0.997826967	0.998227963
TSG1	0.000132475	0.997998674	0.998349588
SALL2	-0.00011856	0.998208882	0.998485307
OR1F1	-0.000116857	0.99823462	0.998485307
OCA2	0.000102011	0.998458894	0.998659478
TBX1	8.71279E-05	0.99868374	0.998834204
TMEM182	-6.29074E-05	0.999049644	0.999149985
KIAA1486	-2.37036E-05	0.999641904	0.999664576
SGCZ	2.22029E-05	0.999664576	0.999664576
