# Supplementary material for: SNRPC promotes hepatocellular carcinoma cell motility by inducing epithelial‐mesenchymal transition
Source: FEBS Open Bio. 2021 May 12;11(6):1757–70. doi: 10.1002/2211-5463.13175 (PMC8167856; doi:10.1002/2211-5463.13175)
Supplement: Supplementary file 3 — Table S3. KEGG pathway of SNRPC coexpressed genes. [file FEB4-11-1757-s005.pdf]

Supplementary Table 3. Kyoto Encyclopedia of Genes and Genomes (KEGG) pathway of SNRPC co-expressed genes.

geneSet	description	link	ES	NES	p Value	FDR	size	leading Edge Num	leading EdgeId	userId
hsa03010	Ribosome	http://www.kegg.jp/kegg-bin/show_pathway?hsa03010+64928+6204+55168+6169+6155+51069+6227+6222+6181+6147+6152+6165+6157+65003+6167+9349+6208+6143+6161+6176+6168+6156+6201+9045+6202+6224+6210+6207+7311+6217+6159+3921+6150+51264+6229+6205+6228+6233+25873+6193+6132+6141+6170+11224+54460+4736+6160+6203+6218+6158+51021+2197+6128+6175+6125+6235+6135+6187+65005+6223+6188+6209+63875+51373+6206+51081+6142+29093+6171+6182+6124+6138+10573+79590+55173+6130+6137+64960+9553+6146+23521+64969+6230+6164+28998+6194+6136+6139+51121+55052+6183+6189+64963+64979+124995+6191+6154+64965+6134+219927+6166+200916+6129+6144+51187+6231+63931	0.91	2.85	0	0	131	107	64928;6204;55168;6169;6155;51069;6227;6222;6181;6147;6152;6165;6157;65003;6167;9349;6208;6143;6161;6176;6168;6156;6201;9045;6202;6224;6210;6207;7311;6217;6159;3921;6150;51264;6229;6205;6228;6233;25873;6193;6132;6141;6170;11224;54460;4736;6160;6203;6218;6158;51021;2197;6128;6175;6125;6235;6135;6187;65005;6223;6188;6209;63875;51373;6206;51081;6142;29093;6171;6182;6124;6138;10573;79590;55173;6130;6137;64960;9553;6146;23521;64969;6230;6164;28998;6194;6136;6139;51121;55052;6183;6189;64963;64979;124995;6191;6154;64965;6134;219927;6166;200916;6129;6144;51187;6231;63931	FAU;MRPL10;MRPL11;MRPL12;MRPL13;MRPL14;MRPL17;MRPL2;MRPL20;MRPL21;MRPL22;MRPL23;MRPL24;MRPL27;MRPL28;MRPL33;MRPL36;MRPL9;MRPS10;MRPS11;MRPS12;MRPS14;MRPS15;MRPS16;MRPS17;MRPS18A;MRPS21;MRPS5;MRPS7;MRPS9;RPL10;RPL10A;RPL11;RPL12;RPL13;RPL13A;RPL14;RPL15;RPL17;RPL18;RPL18A;RPL19;RPL21;RPL22;RPL22L1;RPL23;RPL23A;RPL24;RPL26;RPL26L1;RPL27;RPL27A;RPL28;RPL29;RPL30;RPL31;RPL32;RPL34;RPL35;RPL35A;RPL36;RPL36AL;RPL37;RPL37A;RPL38;RPL39;RPL4;RPL41;RPL5;RPL6;RPL7;RPL7A;RPL8;RPLP0;RPLP1;RPLP2;RPS10;RPS11;RPS12;RPS13;RPS14;RPS15;RPS15A;RPS16;RPS17;RPS18;RPS19;RPS2;RPS20;RPS21;RPS23;RPS24;RPS25;RPS26;RPS27A;RPS29;RPS3;RPS3A;RPS4X;RPS5;RPS6;RPS7;RPS8;RPS9;RPSA;RSL24D1;UBA52
hsa03040	Spliceosome	http://www.kegg.jp/kegg-bin/show_pathway?hsa03040+6631+57819+6633+6628+6635+27258+10907+24148+10084+151903+6636+25804+6632+6626+10465+8896+6634+51645+6637+26121+51690+6629+6627+22	0.78	2.42	0	0	115	54	6631;57819;6633;6628;6635;27258;10907;24148;10084;151903;6636;25804;6632;6626;10465;8896;6634;51645;6637;26121;51690;6629;6627;22827;3178;9092;6100;83443;23658;27339;411	BUD31;CCDC12;CRNKL1;CTNNBL1;CWC15;DHX16;EFTUD2;EIF4A3;HNRNPA1;HNRNPA3;HNRNPC;ISY1;LSM2;LSM3;LSM4;LSM5;LSM7;MAGOH;MAGOHB;PHF5A;PPIE;PPIH;PPIL1;PQBP1;PRPF19;PRPF31;PRPF6;PUF60;RBM22;RBM8A;RP9;SA

		827+3178+9092+6100+83443+23658+27339+4116+51503+55696+7307+10450+56949+55110+9343+8175+153527+25949+56259+10262+199746+57461+9775+220988+3183+10946+8449+51340+9939+84844+6625							6;51503;55696;7307;10450;56949;55110;9343;8175;153527;25949;56259;10262;199746;57461;9775;220988;3183;10946;8449;51340;9939;84844;6625	RT1;SF3A2;SF3A3;SF3B4;SF3B5;SNRNP70;SNRPA;SNRPA1;SNRPB;SNRPB2;SNRPC;SNRPD1;SNRPD2;SNRPD3;SNRPE;SNRPF;SNRPG;SYF2;TXNL4A;U2AF1;U2AF1L4;XAB2;ZMAT2
hsa00190	Oxidative phosphorylation	http://www.kegg.jp/kegg-bin/show_pathway?hsa00190+54539+4726+4715+4695+1340+7381+126328+1345+1350+4713+4710+4725+10063+29796+51079+55967+4696+1337+4701+4728+7388+4722+4724+1329+4716+4709+1537+4718+4708+4707+1351+4702+9296+8992+440567+9377+533+4714+4694+27089+4706+10975+4697+4700+1347+1327+4712+4711+527+7384+529+4731+4717+84701+1349+4704+537+374291+4729+9167+56901	0.79	2.42	0	0	104	61	54539;4726;4715;4695;1340;7381;126328;1345;1350;4713;4710;4725;10063;29796;51079;55967;4696;1337;4701;4728;7388;4722;4724;1329;4716;4709;1537;4718;4708;4707;1351;4702;9296;8992;440567;9377;533;4714;4694;27089;4706;10975;4697;4700;1347;1327;4712;4711;527;7384;529;4731;4717;84701;1349;4704;537;374291;4729;9167;56901	ATP6AP1;ATP6V0B;ATP6V0C;ATP6V0E1;ATP6V1E1;ATP6V1F;COX17;COX4I1;COX4I2;COX5A;COX5B;COX6A1;COX6B1;COX6C;COX7A2;COX7A2L;COX7B;COX7C;COX8A;CYC1;NDUFA1;NDUFA11;NDUFA12;NDUFA13;NDUFA2;NDUFA3;NDUFA4;NDUFA4L2;NDUFA6;NDUFA7;NDUFA8;NDUFA9;NDUFAB1;NDUFB1;NDUFB10;NDUFB11;NDUFB2;NDUFB3;NDUFB4;NDUFB5;NDUFB6;NDUFB7;NDUFB8;NDUFB9;NDUFC1;NDUFC2;NDUFS3;NDUFS4;NDUFS5;NDUFS6;NDUFS7;NDUFS8;NDUFV2;NDUFV3;UQCR10;UQCR11;UQCRB;UQCRC1;UQCRH;UQCRHL;UQCRQ
hsa05012	Parkinson disease	http://www.kegg.jp/kegg-bin/show_pathway?hsa05012+54539+4726+4715+4695+1340+7381+126328+1345+1350+4713+4710+4725+29796+51079+55967+4696+1337+4701+27429+4728+7388+4722+4724+1329+4716+4709+1537+4718+4708+4707+7417+1351+4702+440567+9377+4714+4694+27089+4706+10975+4697+4700+1347+118424+1327+4712+7332+11315+4711	0.74	2.31	0	0	115	61	54539;4726;4715;4695;1340;7381;126328;1345;1350;4713;4710;4725;29796;51079;55967;4696;1337;4701;27429;4728;7388;4722;4724;1329;4716;4709;1537;4718;4708;4707;7417;1351;4702;440567;9377;4714;4694;27089;4706;10975;4697;4700;1347;118424;1327;4712;7332;11315;4711;7384;4731;4717;84701;1349;4704;293;73	COX4I1;COX4I2;COX5A;COX5B;COX6A1;COX6B1;COX6C;COX7A2;COX7A2L;COX7B;COX7C;COX8A;CYC1;HTRA2;NDUFA1;NDUFA11;NDUFA12;NDUFA13;NDUFA2;NDUFA3;NDUFA4;NDUFA4L2;NDUFA6;NDUFA7;NDUFA8;NDUFA9;NDUFAB1;NDUFB1;NDUFB10;NDUFB11;NDUFB2;NDUFB3;NDUFB4;NDUFB5;NDUFB6;NDUFB7;NDUFB8;NDUFB9;NDUFC1;NDUFC2;NDUFS3;NDUFS4;NDUFS5;NDUFS6;NDUFS7;NDUFS8;

		+7384+4731+4717+84701+1349+4704+293+7314+374291+4729+9167+56901							14;374291;4729;9167;56901	NDUFV2;NDUFV3;PARK7;SLC25A6;UBB;UBE2J2;UBE2L3;UQCR10;UQCR11;UQCRB;UQCRC1;UQCRH;UQCRHL;UQCRQ;VDAC2
hsa03050	Proteasome	http://www.kegg.jp/kegg-bin/show_pathway?hsa03050+5691+5719+5710+5692+5705+5685+5700+5687+5695+5702+5688+5694+11047+5689+5686+5704+5693+5714+51371+5721+5690+5720+5682+9491+5684+5683+5709+5699+5698+9861+5701+5713	0.84	2.3	0	0	44	32	5691;5719;5710;5692;5705;5685;5700;5687;5695;5702;5688;5694;11047;5689;5686;5704;5693;5714;51371;5721;5690;5720;5682;9491;5684;5683;5709;5699;5698;9861;5701;5713	ADRM1;POMP;PSMA1;PSMA2;PSMA3;PSMA4;PSMA5;PSMA6;PSMA7;PSMB1;PSMB10;PSMB2;PSMB3;PSMB4;PSMB5;PSMB6;PSMB7;PSMB9;PSMC1;PSMC2;PSMC3;PSMC4;PSMC5;PSMD13;PSMD3;PSMD4;PSMD6;PSMD7;PSMD8;PSME1;PSME2;PSMF1
hsa03030	DNA replication	http://www.kegg.jp/kegg-bin/show_pathway?hsa03030+6119+6742+10535+84153+5984+4176+56655+5982+5424+79621+4172+4174+2237+5558+23649+5111+57804+5425+6118+5557+3978+5427+4171+246243+4175	0.81	2.12	0	0	36	25	6119;6742;10535;84153;5984;4176;56655;5982;5424;79621;4172;4174;2237;5558;23649;5111;57804;5425;6118;5557;3978;5427;4171;246243;4175	FEN1;LIG1;MCM2;MCM3;MCM5;MCM6;MCM7;PCNA;POLA2;POLD1;POLD2;POLD4;POLE2;POLE4;PRIM1;PRIM2;RFC2;RFC4;RNASEH1;RNASEH2A;RNASEH2B;RNASEH2C;RPA2;RPA3;SSBP1
hsa05016	Huntington disease	http://www.kegg.jp/kegg-bin/show_pathway?hsa05016+5437+54539+1211+4726+5438+4715+5436+1175+4695+1340+5439+7381+126328+1345+1350+4713+5441+5435+4710+4725+29796+51079+55967+4696+1337+5440+4701+4728+7388+4722+4724+1329+4716+4709+1537+4718+4708+4707+7417+1351+4702+440567+9377+581+4714+4694+27089+4706+10975+4697+4700+1347+1327+5433+4712+1173+1212+4711+2876+7384+4731+10540+4	0.62	2.03	0	###	174	78	5437;54539;1211;4726;5438;4715;5436;1175;4695;1340;5439;7381;126328;1345;1350;4713;5441;5435;4710;4725;29796;51079;55967;4696;1337;5440;4701;4728;7388;4722;4724;1329;4716;4709;1537;4718;4708;4707;7417;1351;4702;440567;9377;581;4714;4694;27089;4706;10975;4697;4700;1347;1327;5433;4712;1173;1212;4711;2876;7384;4731;10540;4717;84701;148327;1349;4704;5434;293;37	AP2M1;AP2S1;BAX;CLTA;CLTB;COX4I1;COX4I2;COX5A;COX5B;COX6A1;COX6B1;COX6C;COX7A2;COX7A2L;COX7B;COX7C;COX8A;CREB3;CREB3L4;CYC1;DCTN2;GPX1;HDAC1;NDUFA1;NDUFA10;NDUFA11;NDUFA12;NDUFA13;NDUFA2;NDUFA3;NDUFA4;NDUFA4L2;NDUFA6;NDUFA7;NDUFA8;NDUFA9;NDUFAB1;NDUFB1;NDUFB10;NDUFB11;NDUFB2;NDUFB3;NDUFB4;NDUFB5;NDUFB6;NDUFB7;NDUFB8;NDUFB9;NDUFC1;NDUFC2;NDUFS3;NDUFS4;NDUFS5;NDUFS6;NDUFS7;NDUFS8;NDUFV2;NDU

		717+84701+148327+1349+4704+5434+293+374291+4729+3065+9167+56901+5468+6647+10488+4705							4291;4729;3065;9167;56901;5468;6647;10488;4705	FV3;POLR2D;POLR2E;POLR2F;POLR2G;POLR2H;POLR2I;POLR2J;POLR2K;POLR2L;PPARG;SLC25A6;SOD1;UQCR10;UQCR11;UQCRB;UQCRC1;UQCRH;UQCRHL;UQCRQ;VDAC2
hsa00240	Pyrimidine metabolism	http://www.kegg.jp/kegg-bin/show_pathway?hsa00240+30834+5437+9533+1841+4830+30833+5438+5436+4831+10201+5439+5441+5435+4832+5440+10623+7083+79077+54963+7371+318+51082+56655+5424+5433+654364+955+51728+5558+23649+7372+57804+6241+5425+5434+171568+4833+5557+5427+10621	0.66	1.97	0	###	96	40	30834;5437;9533;1841;4830;30833;5438;5436;4831;10201;5439;5441;5435;4832;5440;10623;7083;79077;54963;7371;318;51082;56655;5424;5433;654364;955;51728;5558;23649;7372;57804;6241;5425;5434;171568;4833;5557;5427;10621	DCTPP1;DTYMK;ENTPD6;NME1;NME1-NME2;NME2;NME3;NME4;NME6;NT5C;NUDT2;POLA2;POLD1;POLD2;POLD4;POLE2;POLE4;POLR1C;POLR1D;POLR2D;POLR2E;POLR2F;POLR2G;POLR2H;POLR2I;POLR2J;POLR2K;POLR2L;POLR3C;POLR3F;POLR3H;POLR3K;PRIM1;PRIM2;RRM2;TK1;UCK2;UCKL1;UMPS;ZNRD1
hsa03008	Ribosome biogenesis in eukaryotes	http://www.kegg.jp/kegg-bin/show_pathway?hsa03008+1460+3692+2091+55651+10436+51367+55505+10528+10556+10248+29107+10775+10799+54433+6949+10557+27341+55272+92856+51096+55813+51602+5901+10607	0.67	1.97	0	###	70	24	1460;3692;2091;55651;10436;51367;55505;10528;10556;10248;29107;10775;10799;54433;6949;10557;27341;55272;92856;51096;55813;51602;5901;10607	CSNK2B;EIF6;EMG1;FBL;GAR1;IMP3;IMP4;NHP2;NOP10;NOP56;NOP58;NXT1;POP4;POP5;POP7;RAN;RPP30;RPP38;RPP40;RRP7A;TBL3;TCOF1;UTP18;UTP6
hsa03013	RNA transport	http://www.kegg.jp/kegg-bin/show_pathway?hsa03013+79897+79833+79760+8662+51367+8665+8890+10556+10248+8666+29107+10775+8894+80145+9470+8893+79228+4116+57510+6396+8664+10799+8667+6613+10209+8086+10557+79023+8563+8668+7329+55110+8637+10460+10073+5901+1978+9775+8891+1973+7341+728689+3646+1967+79902+9669+8480+10921	0.6	1.96	0	###	158	49	79897;79833;79760;8662;51367;8665;8890;10556;10248;8666;29107;10775;8894;80145;9470;8893;79228;4116;57510;6396;8664;10799;8667;6613;10209;8086;10557;79023;8563;8668;7329;55110;8637;10460;10073;5901;1978;9775;8891;1973;7341;728689;3646;1967;79902;9669;8480;10921;9939	AAAS;EIF1;EIF2B1;EIF2B3;EIF2B4;EIF2B5;EIF2S2;EIF3B;EIF3CL;EIF3D;EIF3E;EIF3F;EIF3G;EIF3H;EIF3I;EIF4A1;EIF4A3;EIF4E2;EIF4EBP1;EIF4EBP3;EIF5B;GEMIN6;GEMIN7;MAGOH;MAGOHB;NUP37;NUP85;NXT1;POP4;POP5;POP7;RAE1;RAN;RBM8A;RNPS1;RPP21;RPP30;RPP38;RPP40;SEC13;SNUPN;SUMO1;SUMO2;TACC3;THOC5;THOC6;THOC7;UBE2I;XPO5

		+9939								
hsa04510	Focal adhesion	http://www.kegg.jp/kegg-bin/show_pathway?hsa04510+3082+1291+399694+3480+5602+3912+3918+1278+22801+3479+1292+2534+208+5156+7057+1499+56034+858+3685+7424+80310+7148+5155+2335+55742+3371+5500+7058+1284+2932+1398+131873+2316+5159+1399+207+329+8515+3911+3908+2002+1285+1293+596+3676+8503+6654+1286+2321+1282+2324+5594+8516+2889+3688+3680+7094+7414+331+4659+5604+10000+3672+3791+5170+1956+3690+5599+4638+9475+5290+5295+394+4660+6093+1793+6655	-0.6	-1.9	0	0	194	77	3082;1291;399694;3480;5602;3912;3918;1278;22801;3479;1292;2534;208;5156;7057;1499;56034;858;3685;7424;80310;7148;5155;2335;55742;3371;5500;7058;1284;2932;1398;131873;2316;5159;1399;207;329;8515;3911;3908;2002;1285;1293;596;3676;8503;6654;1286;2321;1282;2324;5594;8516;2889;3688;3680;7094;7414;331;4659;5604;10000;3672;3791;5170;1956;3690;5599;4638;9475;5290;5295;394;4660;6093;1793;6655	AKT1;AKT2;AKT3;ARHGAP5;BCL2;BIRC2;CAV2;COL1A2;COL4A1;COL4A2;COL4A3;COL4A4;COL6A1;COL6A2;COL6A3;COL6A6;CRK;CRKL;CTNNB1;DOCK1;EGFR;ELK1;FLNA;FLT1;FLT4;FN1;FYN;GSK3B;HGF;IGF1;IGF1R;ITGA1;ITGA10;ITGA11;ITGA4;ITGA8;ITGA9;ITGAV;ITGB1;ITGB3;KDR;LAMA2;LAMA5;LAMB1;LAMC2;MAP2K1;MAPK1;MAPK10;MAPK8;MYLK;PARVA;PDGFB;PDGFC;PDGFD;PDGFRA;PDGFRB;PDPK1;PIK3CA;PIK3R1;PIK3R3;PPP1CB;PPP1R12A;PPP1R12B;RAPGEF1;ROCK1;ROCK2;SHC4;SOS1;SOS2;THBS1;THBS2;TLN1;TNC;TNXB;VCL;VEGFC;XIAP
hsa03020	RNA polymerase	http://www.kegg.jp/kegg-bin/show_pathway?hsa03020+30834+5437+9533+5438+5436+5439+5441+5435+5440+10623+51082+5433+51728+5434+171568	0.74	1.85	0	0	31	15	30834;5437;9533;5438;5436;5439;5441;5435;5440;10623;51082;5433;51728;5434;171568	POLR1C;POLR1D;POLR2D;POLR2E;POLR2F;POLR2G;POLR2H;POLR2I;POLR2J;POLR2K;POLR2L;POLR3C;POLR3H;POLR3K;ZNRD1
hsa05010	Alzheimer disease	http://www.kegg.jp/kegg-bin/show_pathway?hsa05010+54539+4726+4715+4695+1340+55851+7381+126328+1345+1350+4713+4710+4725+29796+51079+55967+4696+1337+4701+4728+7388+4722+4724+1329+4716+4709+1537+4718+4708+4707+1351+4702+440567+2597+9377+4714+4694+27089+4706+10975+4697+4700+1347+1327+4712+	0.56	1.81	0	0	152	53	54539;4726;4715;4695;1340;55851;7381;126328;1345;1350;4713;4710;4725;29796;51079;55967;4696;1337;4701;4728;7388;4722;4724;1329;4716;4709;1537;4718;4708;4707;1351;4702;440567;2597;9377;4714;4694;27089;4706;10975;4697;4700;1347;1327;4712;4711;1020;637;7384;4731;4717;572;84701	BAD;BID;CDK5;COX4I1;COX4I2;COX5A;COX5B;COX6A1;COX6B1;COX6C;COX7A2;COX7C;COX8A;CYC1;GAPDH;NDUFA1;NDUFA11;NDUFA12;NDUFA13;NDUFA2;NDUFA3;NDUFA4;NDUFA6;NDUFA7;NDUFA8;NDUFAB1;NDUFB1;NDUFB10;NDUFB11;NDUFB2;NDUFB3;NDUFB4;NDUFB5;NDUFB6;NDUFB7;NDUFB8;NDUFB9;NDUFC1;NDUFC2;NDUFS3;NDUFS4;NDUFS5;NDUFS6;NDUFS8;

		4711+1020+637+7384+4731+4717+572+84701								NDUFV3;PSENEN;UQCR10;UQCR11;UQCRB;UQCRC1;UQCRH;UQCRHL;UQCRQ
hsa05322	Systemic lupus erythematosus	http://www.kegg.jp/kegg-bin/show_pathway?hsa05322+6628+6632+6634+3014+3020+8338+3015+55766+474382+8336+6741+8363+8352+85235+8364+8354+717+8351+8335+8366+3017+3021+8358+85236+8341+9555+8331+8337+3012+55506+8367+8370+8332+8339+8334+712+3108+8365+317772+8360+8345	0.59	1.82	0	0	121	41	6628;6632;6634;3014;3020;8338;3015;55766;474382;8336;6741;8363;8352;85235;8364;8354;717;8351;8335;8366;3017;3021;8358;85236;8341;9555;8331;8337;3012;55506;8367;8370;8332;8339;8334;712;3108;8365;317772;8360;8345	C1QA;C2;H2AFB1;H2AFJ;H2AFX;H2AFY;H2AFY2;H2AFZ;H3F3A;H3F3B;HIST1H2AB;HIST1H2AC;HIST1H2AE;HIST1H2AH;HIST1H2AJ;HIST1H2AL;HIST1H2AM;HIST1H2BD;HIST1H2BG;HIST1H2BH;HIST1H2BK;HIST1H2BN;HIST1H3B;HIST1H3C;HIST1H3D;HIST1H3I;HIST1H4B;HIST1H4C;HIST1H4D;HIST1H4E;HIST1H4H;HIST1H4J;HIST2H2AA3;HIST2H2AB;HIST2H2AC;HIST2H4A;HLA-DMA;SNRPB;SNRPD1;SNRPD3;SSB
hsa03430	Mismatch repair	http://www.kegg.jp/kegg-bin/show_pathway?hsa03430+6119+6742+5984+5982+5424+9156+5111+57804+5425+6118+3978	0.76	1.82	0	0	23	11	6119;6742;5984;5982;5424;9156;5111;57804;5425;6118;3978	EXO1;LIG1;PCNA;POLD1;POLD2;POLD4;RFC2;RFC4;RPA2;RPA3;SSBP1
hsa04714	Thermogenesis	http://www.kegg.jp/kegg-bin/show_pathway?hsa04714+91942+54539+4726+4715+4695+1340+3265+7381+126328+1345+1350+4713+6194+4710+4725+10063+29796+51079+55967+4696+1337+4701+4728+7388+4722+4724+1329+4716+64223+51548+84335+4709+1537+4718+25915+4708+4707+1351+4702+440567+9377+4714+4694+27089+4706+10975+4697+4700+1347+51241+1327+23028+4712+90639+4711+7384+29078+4731+4717+84701+148327+6199+1349+4704	0.55	1.8	0	0	189	73	91942;54539;4726;4715;4695;1340;3265;7381;126328;1345;1350;4713;6194;4710;4725;10063;29796;51079;55967;4696;1337;4701;4728;7388;4722;4724;1329;4716;64223;51548;84335;4709;1537;4718;25915;4708;4707;1351;4702;440567;9377;4714;4694;27089;4706;10975;4697;4700;1347;51241;1327;23028;4712;90639;4711;7384;29078;4731;4717;84701;148327;6199;1349;4704;374291;4729;9167;6605;56901;5468;6598;10	AKT1S1;COX16;COX17;COX19;COX4I1;COX4I2;COX5A;COX5B;COX6A1;COX6B1;COX6C;COX7A2;COX7A2L;COX7B;COX7C;COX8A;CREB3;CREB3L4;CYC1;HRAS;KDM1A;MLST8;NDUFA1;NDUFA10;NDUFA11;NDUFA12;NDUFA13;NDUFA2;NDUFA3;NDUFA4;NDUFA4L2;NDUFA6;NDUFA7;NDUFA8;NDUFA9;NDUFAB1;NDUFAF2;NDUFAF3;NDUFAF4;NDUFB1;NDUFB10;NDUFB11;NDUFB2;NDUFB3;NDUFB4;NDUFB5;NDUFB6;NDUFB7;NDUFB8;NDUFB9;NDUFC1;NDUFC2;NDUFS3;NDUFS4;NDUFS5;NDUFS6;NDUFS7;NDUFS8;NDUFV2;NDUFV

		+374291+4729+9167+6605+56901+5468+6598+10488+4705							488;4705	3;PPARG;RPS6;RPS6KB2;SIRT6;SMARCB1;SMARCE1;UQCR10;UQCR11;UQCRB;UQCRC1;UQCRH;UQCRHL;UQCRQ
hsa04512	ECM-receptor interaction	http://www.kegg.jp/kegg-bin/show_pathway?hsa04512+3675+3673+3696+1277+10319+1291+3912+3918+1278+22801+2814+1292+2811+7057+3685+7148+2335+3371+51206+7058+1284+1605+131873+8515+3911+3908+1285+1293+3676+1286+1282+8516+3688+3680+3672+3339+3690	-0.6	-1.8	0	0	80	37	3675;3673;3696;1277;10319;1291;3912;3918;1278;22801;2814;1292;2811;7057;3685;7148;2335;3371;51206;7058;1284;1605;131873;8515;3911;3908;1285;1293;3676;1286;1282;8516;3688;3680;3672;3339;3690	COL1A1;COL1A2;COL4A1;COL4A2;COL4A3;COL4A4;COL6A1;COL6A2;COL6A3;COL6A6;DAG1;FN1;GP1BA;GP5;GP6;HSPG2;ITGA1;ITGA10;ITGA11;ITGA2;ITGA3;ITGA4;ITGA8;ITGA9;ITGAV;ITGB1;ITGB3;ITGB8;LAMA2;LAMA5;LAMB1;LAMC2;LAMC3;THBS1;THBS2;TNC;TNXB
hsa04630	JAK-STAT signaling pathway	http://www.kegg.jp/kegg-bin/show_pathway?hsa04630+3574+4170+3560+208+5156+3569+8554+7297+8835+5155+3597+3977+6777+6774+5159+207+9063+8027+2273+9180+1270+596+9655+2475+8503+6654+3570+3575+9306+10254+1387+3717+2690+10000+1956+122809+3716+5290+2033+5295+5781+6655+3572	-0.6	-1.9	0	0	151	43	3574;4170;3560;208;5156;3569;8554;7297;8835;5155;3597;3977;6777;6774;5159;207;9063;8027;2273;9180;1270;596;9655;2475;8503;6654;3570;3575;9306;10254;1387;3717;2690;10000;1956;122809;3716;5290;2033;5295;5781;6655;3572	AKT1;AKT2;AKT3;BCL2;CNTF;CREBBP;EGFR;EP300;FHL1;GHR;IL13RA1;IL2RB;IL6;IL6R;IL6ST;IL7;IL7R;JAK1;JAK2;LIFR;MCL1;MTOR;OSMR;PDGFB;PDGFRA;PDGFRB;PIAS1;PIAS2;PIK3CA;PIK3R1;PIK3R3;PTPN11;SOCS2;SOCS4;SOCS5;SOCS6;SOS1;SOS2;STAM;STAM2;STAT3;STAT5B;TYK2
hsa04930	Type II diabetes mellitus	http://www.kegg.jp/kegg-bin/show_pathway?hsa04930+3098+8660+2645+3643+3551+9021+776+5602+3667+8835+2475+8503+5594+775+122809+5581+5599+5290+5295	-0.7	-1.8	0	0	45	19	3098;8660;2645;3643;3551;9021;776;5602;3667;8835;2475;8503;5594;775;122809;5581;5599;5290;5295	CACNA1C;CACNA1D;GCK;HK1;IKBKB;INSR;IRS1;IRS2;MAPK1;MAPK10;MAPK8;MTOR;PIK3CA;PIK3R1;PIK3R3;PRKCE;SOCS2;SOCS3;SOCS4
hsa04015	Rap1 signaling pathway	http://www.kegg.jp/kegg-bin/show_pathway?hsa04015+3082+3480+5332+8817+5899+3479+999+1902+2260+208+5156+5587+7057+1499+56034+7074+284+	-0.6	-1.8	0	0	202	73	3082;3480;5332;8817;5899;3479;999;1902;2260;208;5156;5587;7057;1499;56034;7074;284;7424;1969;80310;5155;3815;23094;51378;196883	ADCY3;ADCY4;ADCY5;ADCY9;AKT1;AKT2;AKT3;ANGPT1;ANGPT4;ARAP3;CDH1;CRK;CRKL;CSF1;CTNNB1;CTNND1;DOCK4;EFNA2;EGFR;EPHA2;F2R;FARP2;FGF18;FGF7;FGFR1;

		7424+1969+80310+5155+3815+23094+51378+196883+83593+1398+1943+5900+260425+23683+25780+5159+1399+207+111+1435+2846+9863+2775+2252+109+3845+115+64411+8503+2321+2324+9855+5594+9732+2889+3688+2149+7094+11069+26037+2776+9771+5604+10000+5028+889+3791+1956+7010+3690+1500+5290+5295+9223+9693+51735						;83593;1398;1943;5900;260425;23683;25780;5159;1399;207;111;1435;2846;9863;2775;2252;109;3845;115;64411;8503;2321;2324;9855;5594;9732;2889;3688;2149;7094;11069;26037;2776;9771;5604;10000;5028;889;3791;1956;7010;3690;1500;5290;5295;9223;9693;51735	FLT1;FLT4;GNAO1;GNAQ;HGF;IGF1;IGF1R;ITGB1;ITGB3;KDR;KIT;KRAS;KRIT1;LPAR1;LPAR4;MAGI1;MAGI2;MAGI3;MAP2K1;MAPK1;P2RY1;PDGFB;PDGFC;PDGFD;PDGFRA;PDGFRB;PIK3CA;PIK3R1;PIK3R3;PLCB4;PRKD1;PRKD3;RALB;RALGDS;RAPGEF1;RAPGEF2;RAPGEF4;RAPGEF5;RAPGEF6;RASGRP3;RASSF5;SIPA1L1;SIPA1L3;TEK;THBS1;TIAM1;TLN1;VEGFC
hsa04611	Platelet activation	http://www.kegg.jp/kegg-bin/show_pathway?hsa04611+3937+3709+340156+3673+5567+23533+112+1277+5321+6786+8773+5332+1278+5566+2814+3710+2534+208+2811+5336+51206+10672+196883+5500+2977+1281+207+111+109+115+4846+8503+5294+5594+64805+3688+2149+7094+4659+2776+3708+10000+5028+3690+4638+9475+5290+5295+6093+5592+23365	-0.6	-1.8	0	0	118	51	3937;3709;340156;3673;5567;23533;112;1277;5321;6786;8773;5332;1278;5566;2814;3710;2534;208;2811;5336;51206;10672;196883;5500;2977;1281;207;111;109;115;4846;8503;5294;5594;64805;3688;2149;7094;4659;2776;3708;10000;5028;3690;4638;9475;5290;5295;6093;5592;23365	ADCY3;ADCY4;ADCY5;ADCY6;ADCY9;AKT1;AKT2;AKT3;ARHGEF12;COL1A1;COL1A2;COL3A1;F2R;FYN;GNA13;GNAQ;GP1BA;GP5;GP6;GUCY1A2;ITGA2;ITGB1;ITGB3;ITPR1;ITPR2;ITPR3;LCP2;MAPK1;MYLK;MYLK4;NOS3;P2RY1;P2RY12;PIK3CA;PIK3CG;PIK3R1;PIK3R3;PIK3R5;PLA2G4A;PLCB4;PLCG2;PPP1CB;PPP1R12A;PRKACA;PRKACB;PRKG1;ROCK1;ROCK2;SNAP23;STIM1;TLN1
hsa04932	Non-alcoholic fatty liver disease (NAFLD)	http://www.kegg.jp/kegg-bin/show_pathway?hsa04932+54539+4726+4715+4695+1340+7381+126328+1345+1350+4713+4710+4725+29796+51079+55967+4696+1337+4701+4728+7388+4722+4724+1329+4716+4709+1537+4718+4708+4707+1351+4702+440567+9377+581+4714+5879+4694+27089+4706+10975+4697	0.55	1.76	0	0	143	60	54539;4726;4715;4695;1340;7381;126328;1345;1350;4713;4710;4725;29796;51079;55967;4696;1337;4701;4728;7388;4722;4724;1329;4716;4709;1537;4718;4708;4707;1351;4702;440567;9377;581;4714;5879;4694;27089;4706;10975;4697;4700;1347;7186;1327;4712;468;4711;1649;637;	ATF4;BAX;BID;CEBPA;COX4I1;COX4I2;COX5A;COX5B;COX6A1;COX6B1;COX6C;COX7A2;COX7B;COX7C;COX8A;CYC1;DDIT3;NDUFA1;NDUFA11;NDUFA12;NDUFA13;NDUFA2;NDUFA3;NDUFA4;NDUFA6;NDUFA7;NDUFA8;NDUFA9;NDUFAB1;NDUFB1;NDUFB10;NDUFB11;NDUFB2;NDUFB3;NDUFB4;NDUFB5;NDUFB6;NDUFB7;NDUFB8;NDUFB9;NDUFC1;N

		+4700+1347+7186+1327+4712+468+4711+1649+637+7384+4731+4717+84701+1349+10062+4704+1050+374291+4729							7384;4731;4717;84701;1349;10062;4704;1050;374291;4729	DUFC2;NDUFS3;NDUFS4;NDUFS5;NDUFS6;NDUFS7;NDUFS8;NDUFV2;NDUFV3;NR1H3;RAC1;TRAF2;UQCR10;UQCR11;UQCRB;UQCRC1;UQCRH;UQCRHL;UQCRQ
hsa04360	Axon guidance	http://www.kegg.jp/kegg-bin/show_pathway?hsa04360+816+5530+1969+6387+1949+5336+817+10512+57689+10509+64101+57522+23654+2932+1943+56920+6586+7223+8829+9037+5534+80031+3845+1808+8503+2042+59277+4917+91584+5727+10154+25+151449+5594+3688+1948+2043+4690+8633+5921+4773+9353+9475+5290+5295+5781+6093+54434+659+23365	-0.6	-1.8	0	0	173	50	816;5530;1969;6387;1949;5336;817;10512;57689;10509;64101;57522;23654;2932;1943;56920;6586;7223;8829;9037;5534;80031;3845;1808;8503;2042;59277;4917;91584;5727;10154;25;151449;5594;3688;1948;2043;4690;8633;5921;4773;9353;9475;5290;5295;5781;6093;54434;659;23365	ABL1;ARHGEF12;BMPR2;CAMK2B;CAMK2D;CXCL12;DPYSL2;EFNA2;EFNB2;EFNB3;EPHA2;EPHA3;EPHA4;GDF7;GSK3B;ITGB1;KRAS;LRRC4;LRRC4C;MAPK1;NCK1;NFATC2;NRP1;NTN3;NTN4;PIK3CA;PIK3R1;PIK3R3;PLCG2;PLXNA4;PLXNB2;PLXNC1;PPP3CA;PPP3R1;PTCH1;PTPN11;RASA1;ROCK1;ROCK2;SEMA3C;SEMA3G;SEMA4B;SEMA5A;SEMA6D;SLIT2;SLIT3;SRGAP1;SSH1;TRPC4;UNC5C
hsa04668	TNF signaling pathway	http://www.kegg.jp/kegg-bin/show_pathway?hsa04668+6401+7185+10059+3725+841+3551+9021+4792+840+153090+5602+4323+1385+208+3569+5743+192111+7424+6416+182+7128+2353+6885+207+329+1435+257397+64764+9252+8837+83737+355+8503+4217+8809+5594+23118+5604+10000+4790+1386+5599+5290+5295+9530	-0.6	-1.8	0	0	110	45	6401;7185;10059;3725;841;3551;9021;4792;840;153090;5602;4323;1385;208;3569;5743;192111;7424;6416;182;7128;2353;6885;207;329;1435;257397;64764;9252;8837;83737;355;8503;4217;8809;5594;23118;5604;10000;4790;1386;5599;5290;5295;9530	AKT1;AKT2;AKT3;ATF2;BAG4;BIRC2;CASP7;CASP8;CFLAR;CREB1;CREB3L2;CSF1;DAB2IP;DNM1L;FAS;FOS;IKBKB;IL18R1;IL6;ITCH;JAG1;JUN;MAP2K1;MAP2K4;MAP3K5;MAP3K7;MAPK1;MAPK10;MAPK8;MMP14;NFKB1;NFKBIA;PGAM5;PIK3CA;PIK3R1;PIK3R3;PTGS2;RPS6KA5;SELE;SOCS3;TAB2;TAB3;TNFAIP3;TRAF1;VEGFC
hsa00562	Inositol phosphate metabolism	http://www.kegg.jp/kegg-bin/show_pathway?hsa00562+5287+5336+3707+5297+79837+8897+4329+55300+8776+8394+5294+3631+64768+8871+22908+4534+253430+9107+5290+20057	-0.6	-1.8	0	0	74	22	5287;5336;3707;5297;79837;8897;4329;55300;8776;8394;5294;3631;64768;8871;22908;4534;253430;9107;5290;200576;8867;5286	ALDH6A1;INPP4A;IPMK;IPPK;ITPKB;MTM1;MTMR1;MTMR3;MTMR6;PI4K2B;PI4KA;PIK3C2A;PIK3C2B;PIK3CA;PIK3CG;PIKFYVE;PIP4K2C;PIP5K1A;PLCG2;SACM1L;SYNJ1;SYNJ2

		6+8867+5286								
hsa04392	Hippo signaling pathway	http://www.kegg.jp/kegg-bin/show_pathway?hsa04392+166824+9770+25937+10413+8642+122786+8994+60485+9113+26524+7003+79633	-0.8	-1.7	0	0	25	12	166824;9770;25937;10413;8642;122786;8994;60485;9113;26524;7003;79633	DCHS1;FAT4;FRMD6;LATS1;LATS2;LIMD1;RASSF2;RASSF6;SAV1;TEAD1;WWTR1;YAP1
hsa04070	Phosphatidylinositol signaling system	http://www.kegg.jp/kegg-bin/show_pathway?hsa04070+5287+5336+3707+5297+79837+9677+8897+55300+8776+8503+8394+3631+64768+8871+23262+22908+3708+4534+253430+9107+5290+5295+200576+8867+5286	-0.6	-1.7	0	0	97	25	5287;5336;3707;5297;79837;9677;8897;55300;8776;8503;8394;3631;64768;8871;23262;22908;3708;4534;253430;9107;5290;5295;200576;8867;5286	INPP4A;IPMK;IPPK;ITPKB;ITPR1;MTM1;MTMR1;MTMR3;MTMR6;PI4K2B;PI4KA;PIK3C2A;PIK3C2B;PIK3CA;PIK3R1;PIK3R3;PIKFYVE;PIP4K2C;PIP5K1A;PLCG2;PPIP5K1;PPIP5K2;SACM1L;SYNJ1;SYNJ2
hsa04550	Signaling pathways regulating pluripotency of stem cells	http://www.kegg.jp/kegg-bin/show_pathway?hsa04550+657+3480+7482+3479+7472+8325+2260+208+1499+7483+90+8321+3977+91+2932+6774+7484+207+4090+8322+648+463+3845+3626+8503+7473+3175+5594+3624+4093+3717+6498+5604+10000+4086+324+3716+56916+55183+5290+5295+659+3572+5978	-0.6	-1.7	0	0	136	44	657;3480;7482;3479;7472;8325;2260;208;1499;7483;90;8321;3977;91;2932;6774;7484;207;4090;8322;648;463;3845;3626;8503;7473;3175;5594;3624;4093;3717;6498;5604;10000;4086;324;3716;56916;55183;5290;5295;659;3572;5978	ACVR1;ACVR1B;AKT1;AKT2;AKT3;APC;BMI1;BMPR1A;BMPR2;CTNNB1;FGFR1;FZD1;FZD4;FZD8;GSK3B;IGF1;IGF1R;IL6ST;INHBA;INHBC;JAK1;JAK2;KRAS;LIFR;MAP2K1;MAPK1;ONECUT1;PIK3CA;PIK3R1;PIK3R3;REST;RIF1;SKIL;SMAD1;SMAD5;SMAD9;SMARCAD1;STAT3;WNT2;WNT2B;WNT3;WNT9A;WNT9B;ZFHX3
hsa04022	cGMP-PKG signaling pathway	http://www.kegg.jp/kegg-bin/show_pathway?hsa04022+340156+7225+2768+3643+23533+5140+112+3778+776+8654+5332+3710+1385+208+3667+5530+5350+1909+4208+10672+196883+5500+2977+10335+624+147+493+207+111+5534+64764+109+115+4846+6546+5294+1910+5594+775+5138+148+488+4659+27	-0.5	-1.7	0	0	160	58	340156;7225;2768;3643;23533;5140;112;3778;776;8654;5332;3710;1385;208;3667;5530;5350;1909;4208;10672;196883;5500;2977;10335;624;147;493;207;111;5534;64764;109;115;4846;6546;5294;1910;5594;775;5138;148;488;4659;2776;3708;5604;10000;4773;1386;5139;5581;2969;46	ADCY3;ADCY4;ADCY5;ADCY6;ADCY9;ADRA1A;ADRA1B;AKT1;AKT2;AKT3;ATF2;ATP2A2;ATP2B4;BDKRB2;CACNA1C;CACNA1D;CREB1;CREB3L2;EDNRA;EDNRB;GNA11;GNA12;GNA13;GNAQ;GTF2I;GUCY1A2;INSR;IRS1;ITPR1;ITPR3;KCNMA1;MAP2K1;MAPK1;MEF2A;MEF2C;MRVI1;MYLK;MYLK4;NFATC2;NOS3;PDE2A;PDE3A;PDE3B;PDE5A;PIK3CG;PIK3R5

		76+3708+5604+10000+4773+1386+5139+5581+2969+4638+9475+6093+5592+2767+4205							38;9475;6093;5592;2767;4205	;PLCB4;PLN;PPP1CB;PPP1R12A;PPP3CA;PPP3R1;PRKCE;PRKG1;ROCK1;ROCK2;SLC8A1;TRPC6
hsa05205	Proteoglycans in cancer	http://www.kegg.jp/kegg-bin/show_pathway?hsa05205+3082+3480+4313+7482+5566+3479+3710+4478+7472+8325+1655+2260+208+4193+7057+1499+7483+816+858+4060+7074+3685+5336+817+2335+288+5500+7078+8321+1634+6774+7484+2316+207+8322+8826+2549+2002+3845+10818+355+2475+8503+7473+5727+6654+2099+5594+7099+3688+5962+3091+867+4659+3708+5604+10000+3791+5170+1956+3339+3690+9475+5290+5295+5781+4660+6093+6655+23365	-0.5	-1.7	0	0	196	70	3082;3480;4313;7482;5566;3479;3710;4478;7472;8325;1655;2260;208;4193;7057;1499;7483;816;858;4060;7074;3685;5336;817;2335;288;5500;7078;8321;1634;6774;7484;2316;207;8322;8826;2549;2002;3845;10818;355;2475;8503;7473;5727;6654;2099;5594;7099;3688;5962;3091;867;4659;3708;5604;10000;3791;5170;1956;3339;3690;9475;5290;5295;5781;4660;6093;6655;23365	AKT1;AKT2;AKT3;ANK3;ARHGEF12;CAMK2B;CAMK2D;CAV2;CBL;CTNNB1;DCN;DDX5;EGFR;ELK1;ESR1;FAS;FGFR1;FLNA;FN1;FRS2;FZD1;FZD4;FZD8;GAB1;HGF;HIF1A;HSPG2;IGF1;IGF1R;IQGAP1;ITGAV;ITGB1;ITGB3;ITPR1;ITPR3;KDR;KRAS;LUM;MAP2K1;MAPK1;MDM2;MMP2;MSN;MTOR;PDPK1;PIK3CA;PIK3R1;PIK3R3;PLCG2;PPP1CB;PPP1R12A;PPP1R12B;PRKACA;PTCH1;PTPN11;RDX;ROCK1;ROCK2;SOS1;SOS2;STAT3;THBS1;TIAM1;TIMP3;TLR4;WNT2;WNT2B;WNT3;WNT9A;WNT9B
hsa04270	Vascular smooth muscle contraction	http://www.kegg.jp/kegg-bin/show_pathway?hsa04270+1909+4629+9826+10672+196883+5500+2977+10335+147+111+109+115+81579+5583+5594+552+775+148+10203+4659+2776+3708+5604+5581+4638+9475+800+4660+6093+5592+2767+23365	-0.6	-1.7	0	0	118	32	1909;4629;9826;10672;196883;5500;2977;10335;147;111;109;115;81579;5583;5594;552;775;148;10203;4659;2776;3708;5604;5581;4638;9475;800;4660;6093;5592;2767;23365	ADCY3;ADCY4;ADCY5;ADCY9;ADRA1A;ADRA1B;ARHGEF11;ARHGEF12;AVPR1A;CACNA1C;CALCRL;CALD1;EDNRA;GNA11;GNA13;GNAQ;GUCY1A2;ITPR1;MAP2K1;MAPK1;MRVI1;MYH11;MYLK;PLA2G12A;PPP1CB;PPP1R12A;PPP1R12B;PRKCE;PRKCH;PRKG1;ROCK1;ROCK2
hsa04923	Regulation of lipolysis in adipocytes	http://www.kegg.jp/kegg-bin/show_pathway?hsa04923+4881+5291+107+113+8660+5567+3643+5140+112+3991+5566+208+5743+5733+3667+51099+11343+4886+196883+207+111+109+115+8503+10000+5290	-0.6	-1.7	0	0	54	28	4881;5291;107;113;8660;5567;3643;5140;112;3991;5566;208;5743;5733;3667;51099;11343;4886;196883;207;111;109;115;8503;10000;5290;5295;5592	ABHD5;ADCY1;ADCY3;ADCY4;ADCY5;ADCY6;ADCY7;ADCY9;AKT1;AKT2;AKT3;INSR;IRS1;IRS2;LIPE;MGLL;NPR1;NPY1R;PDE3B;PIK3CA;PIK3CB;PIK3R1;PIK3R3;PRKACA;PRKACB;PRKG1;PTGER3;PTGS2

		+5295+5592								
hsa04380	Osteoclast differentiation	http://www.kegg.jp/kegg-bin/show_pathway?hsa04380+9846+3725+2354+3551+9021+4792+140885+5602+2534+1385+208+814+9103+5530+7297+5336+2274+4286+2353+6885+7046+2355+207+1435+5534+11024+8503+5594+23118+7048+3554+7189+5604+10000+4773+1540+4790+3716+3690+5599+5290+10326+5295	-0.6	-1.7	0	0	126	43	9846;3725;2354;3551;9021;4792;140885;5602;2534;1385;208;814;9103;5530;7297;5336;2274;4286;2353;6885;7046;2355;207;1435;5534;11024;8503;5594;23118;7048;3554;7189;5604;10000;4773;1540;4790;3716;3690;5599;5290;10326;5295	AKT1;AKT2;AKT3;CAMK4;CREB1;CSF1;CYLD;FCGR2C;FHL2;FOS;FOSB;FOSL2;FYN;GAB2;IKBKB;IL1R1;ITGB3;JAK1;JUN;LILRA1;MAP2K1;MAP3K7;MAPK1;MAPK10;MAPK8;MITF;NFATC2;NFKB1;NFKBIA;PIK3CA;PIK3R1;PIK3R3;PLCG2;PPP3CA;PPP3R1;SIRPA;SIRPB1;SOCS3;TAB2;TGFBR1;TGFBR2;TRAF6;TYK2
hsa04020	Calcium signaling pathway	http://www.kegg.jp/kegg-bin/show_pathway?hsa04020+776+3356+6786+5332+4842+5566+6869+3710+56413+4843+5156+5024+814+5733+816+5530+5350+5336+817+1812+1909+3707+1129+3363+196883+5136+5137+5724+624+5737+57620+3274+147+493+5159+5255+5260+5534+109+115+4846+6546+2185+9630+1910+10800+552+775+2149+148+5027+488+57105+2776+3708+1956+4638+2767	-0.5	-1.7	0	0	181	58	776;3356;6786;5332;4842;5566;6869;3710;56413;4843;5156;5024;814;5733;816;5530;5350;5336;817;1812;1909;3707;1129;3363;196883;5136;5137;5724;624;5737;57620;3274;147;493;5159;5255;5260;5534;109;115;4846;6546;2185;9630;1910;10800;552;775;2149;148;5027;488;57105;2776;3708;1956;4638;2767	ADCY3;ADCY4;ADCY9;ADRA1A;ADRA1B;ATP2A2;ATP2B4;AVPR1A;BDKRB2;CACNA1C;CACNA1D;CAMK2B;CAMK2D;CAMK4;CHRM2;CYSLTR1;CYSLTR2;DRD1;EDNRA;EDNRB;EGFR;F2R;GNA11;GNA14;GNAQ;HRH2;HTR2A;HTR7;ITPKB;ITPR1;ITPR3;LTB4R2;MYLK;NOS1;NOS2;NOS3;P2RX3;P2RX7;PDE1A;PDE1C;PDGFRA;PDGFRB;PHKA1;PHKG1;PLCB4;PLCG2;PLN;PPP3CA;PPP3R1;PRKACA;PTAFR;PTGER3;PTGFR;PTK2B;SLC8A1;STIM1;STIM2;TACR1
hsa04024	cAMP signaling pathway	http://www.kegg.jp/kegg-bin/show_pathway?hsa04024+7410+5732+5337+51196+2492+51+117+5567+5140+3725+112+6548+4792+776+3991+2550+64399+5602+5566+1385+208+814+5733+816+627+7074+5142+4886+5350+817+1812+3351+1909+5465+1129+196883+5500+2353+493+2	-0.5	-1.7	0	0	194	67	7410;5732;5337;51196;2492;51;117;5567;5140;3725;112;6548;4792;776;3991;2550;64399;5602;5566;1385;208;814;5733;816;627;7074;5142;4886;5350;817;1812;3351;1909;5465;1129;196883;5500;2353;493;207;111;64764;109;2737;115;64411;8503;5727;5594;	ACOX1;ADCY3;ADCY4;ADCY5;ADCY6;ADCY9;ADCYAP1R1;AKT1;AKT2;AKT3;ARAP3;ATP2A2;ATP2B4;BDNF;CACNA1C;CACNA1D;CAMK2B;CAMK2D;CAMK4;CHRM2;CREB1;CREB3L2;CREBBP;DRD1;EDNRA;EP300;F2R;FOS;FSHR;GABBR1;GLI3;HHIP;HTR1B;JUN;LIPE;MAP2K1;MAPK1;MAPK10;MAPK8;NFKB1;NFKBIA;NPY1R;P

		07+111+64764+109+2737+115+64411+8503+5727+5594+775+2149+1387+11069+488+4659+5604+10000+56670+4790+5139+10846+5599+9475+5290+2033+5295+6093							775;2149;1387;11069;488;4659;5604;10000;56670;4790;5139;10846;5599;9475;5290;2033;5295;6093	DE10A;PDE3A;PDE3B;PDE4B;PIK3CA;PIK3R1;PIK3R3;PLCE1;PLD1;PLN;PPARA;PPP1CB;PPP1R12A;PRKACA;PRKACB;PTCH1;PTGER2;PTGER3;RAPGEF4;ROCK1;ROCK2;SLC9A1;SUCNR1;TIAM1;VAV2
hsa05142	Chagas disease (American trypanosomiasis)	http://www.kegg.jp/kegg-bin/show_pathway?hsa05142+7097+5519+1147+148022+3725+841+3551+4792+7043+5332+5602+4843+208+3569+51135+6416+5520+2353+624+5516+7046+1636+207+2775+8837+4615+355+10333+8503+9630+5594+7099+7048+7189+2776+10000+4790+5599+5290+5295+2767	-0.6	-1.7	0	0	101	41	7097;5519;1147;148022;3725;841;3551;4792;7043;5332;5602;4843;208;3569;51135;6416;5520;2353;624;5516;7046;1636;207;2775;8837;4615;355;10333;8503;9630;5594;7099;7048;7189;2776;10000;4790;5599;5290;5295;2767	ACE;AKT1;AKT2;AKT3;BDKRB2;CASP8;CFLAR;CHUK;FAS;FOS;GNA11;GNA14;GNAO1;GNAQ;IKBKB;IL6;IRAK4;JUN;MAP2K4;MAPK1;MAPK10;MAPK8;MYD88;NFKB1;NFKBIA;NOS2;PIK3CA;PIK3R1;PIK3R3;PLCB4;PPP2CB;PPP2R1B;PPP2R2A;TGFB3;TGFBR1;TGFBR2;TICAM1;TLR2;TLR4;TLR6;TRAF6
hsa04014	Ras signaling pathway	http://www.kegg.jp/kegg-bin/show_pathway?hsa04014+5337+1147+51196+7039+5567+3643+9846+4804+3551+2246+5321+3082+5869+399694+3480+5602+4303+8817+5899+5566+3479+2260+208+5156+56034+627+7074+4908+8831+284+4915+7424+1969+80310+5336+5155+3815+3363+51378+382+59345+8036+6789+83593+1943+5900+25780+5159+207+1435+55970+2549+2002+2114+2252+5868+10928+3845+81579+5924+9462+8503+23179+6654+2321+2324+25+5594+29110+4763+9771+5604+10000+5921+3791+1956+4790+5922+7010+5599+5290+5295+5781+2113+	-0.5	-1.7	0	0	225	86	5337;1147;51196;7039;5567;3643;9846;4804;3551;2246;5321;3082;5869;399694;3480;5602;4303;8817;5899;5566;3479;2260;208;5156;56034;627;7074;4908;8831;284;4915;7424;1969;80310;5336;5155;3815;3363;51378;382;59345;8036;6789;83593;1943;5900;25780;5159;207;1435;55970;2549;2002;2114;2252;5868;10928;3845;81579;5924;9462;8503;23179;6654;2321;2324;25;5594;29110;4763;9771;5604;10000;5921;3791;1956;4790;5922;7010;5599;5290;5295;5781;2113;5966;6655	ABL1;AKT1;AKT2;AKT3;ANGPT1;ANGPT4;ARF6;BDNF;CHUK;CSF1;EFNA2;EGFR;ELK1;EPHA2;ETS1;ETS2;FGF1;FGF18;FGF7;FGFR1;FLT1;FLT4;FOXO4;GAB1;GAB2;GNB4;GNG12;HGF;HTR7;IGF1;IGF1R;IKBKB;INSR;KDR;KIT;KRAS;MAP2K1;MAPK1;MAPK10;MAPK8;NF1;NFKB1;NGFR;NTF3;NTRK2;PDGFB;PDGFC;PDGFD;PDGFRA;PDGFRB;PIK3CA;PIK3R1;PIK3R3;PLA2G12A;PLA2G4A;PLCE1;PLCG2;PLD1;PRKACA;PRKACB;PTPN11;RAB5A;RAB5B;RALB;RALBP1;RALGDS;RAPGEF5;RASA1;RASA2;RASAL2;RASGRF2;RASGRP3;RASSF5;REL;RGL1;SHC4;SHOC2;SOS1;SOS2;STK4;SYNGAP1;TBK1;TEK;TGFA;TIAM1;VEGFC

		5966+6655								
hsa04919	Thyroid hormone signaling pathway	http://www.kegg.jp/kegg-bin/show_pathway?hsa04919+5332+10231+5566+1734+208+9882+4193+1499+9969+25942+3685+5350+5336+6567+7067+2932+5469+207+9968+1827+3845+4854+4851+2475+8503+2308+2099+5594+4855+4853+9611+1387+3091+488+8850+8648+5604+10000+5170+9282+3690+23389+5290+10499+2033+5295	-0.6	-1.7	0	0	116	46	5332;10231;5566;1734;208;9882;4193;1499;9969;25942;3685;5350;5336;6567;7067;2932;5469;207;9968;1827;3845;4854;4851;2475;8503;2308;2099;5594;4855;4853;9611;1387;3091;488;8850;8648;5604;10000;5170;9282;3690;23389;5290;10499;2033;5295	AKT1;AKT2;AKT3;ATP2A2;CREBBP;CTNNB1;DIO2;EP300;ESR1;FOXO1;GSK3B;HIF1A;ITGAV;ITGB3;KAT2B;KRAS;MAP2K1;MAPK1;MDM2;MED1;MED12;MED13;MED13L;MED14;MTOR;NCOA1;NCOA2;NCOR1;NOTCH1;NOTCH2;NOTCH3;NOTCH4;PDPK1;PIK3CA;PIK3R1;PIK3R3;PLCB4;PLCG2;PLN;PRKACA;RCAN1;RCAN2;SIN3A;SLC16A2;TBC1D4;THRA
hsa05414	Dilated cardiomyopathy (DCM)	http://www.kegg.jp/kegg-bin/show_pathway?hsa05414+70+3675+3673+5567+112+3696+776+7043+22801+5566+3479+3685+5350+7273+196883+1605+6444+783+111+8515+3908+109+115+6443+3676+6546+1756+8516+3688+775+3680+781+488+3672+3690	-0.6	-1.7	0	0	89	35	70;3675;3673;5567;112;3696;776;7043;22801;5566;3479;3685;5350;7273;196883;1605;6444;783;111;8515;3908;109;115;6443;3676;6546;1756;8516;3688;775;3680;781;488;3672;3690	ACTC1;ADCY3;ADCY4;ADCY5;ADCY6;ADCY9;ATP2A2;CACNA1C;CACNA1D;CACNA2D1;CACNB2;DAG1;DMD;IGF1;ITGA1;ITGA10;ITGA11;ITGA2;ITGA3;ITGA4;ITGA8;ITGA9;ITGAV;ITGB1;ITGB3;ITGB8;LAMA2;PLN;PRKACA;PRKACB;SGCB;SGCD;SLC8A1;TGFB3;TTN
hsa04072	Phospholipase D signaling pathway	http://www.kegg.jp/kegg-bin/show_pathway?hsa04072+8526+56894+5335+10411+107+113+5894+5337+9162+2768+2205+3643+23533+9846+2918+112+3579+5321+399694+5332+5899+2534+1902+208+5156+56034+80310+1785+5336+5155+3815+26052+10672+382+196883+5737+5900+5159+207+111+2846+2549+109+3845+115+2475+8503+8394+6654+2185+5294+5594+552+9265+2149+11069+5604+10000+1956+5290+5295+5781+6655	-0.5	-1.7	0	0	140	63	8526;56894;5335;10411;107;113;5894;5337;9162;2768;2205;3643;23533;9846;2918;112;3579;5321;399694;5332;5899;2534;1902;208;5156;56034;80310;1785;5336;5155;3815;26052;10672;382;196883;5737;5900;5159;207;111;2846;2549;109;3845;115;2475;8503;8394;6654;2185;5294;5594;552;9265;2149;11069;5604;10000;1956;5290;5295;5781;6655	ADCY1;ADCY3;ADCY4;ADCY5;ADCY6;ADCY7;ADCY9;AGPAT3;AKT1;AKT2;AKT3;ARF6;AVPR1A;CXCR2;CYTH3;DGKE;DGKI;DNM2;DNM3;EGFR;F2R;FCER1A;FYN;GAB1;GAB2;GNA12;GNA13;GRM8;INSR;KIT;KRAS;LPAR1;LPAR4;MAP2K1;MAPK1;MTOR;PDGFB;PDGFC;PDGFD;PDGFRA;PDGFRB;PIK3CA;PIK3CG;PIK3R1;PIK3R3;PIK3R5;PIP5K1A;PLA2G4A;PLCB4;PLCG1;PLCG2;PLD1;PTGFR;PTK2B;PTPN11;RAF1;RALB;RALGDS;RAPGEF3;RAPGEF4;SHC4;SOS1;SOS2

hsa00640	Propanoate metabolism	http://www.kegg.jp/kegg-bin/show_pathway?hsa00640+23417+5095+38+1962+8801+1738+32+18+92483+4329+594+8803+34+1629	-0.7	-1.7	0	0	31	14	23417;5095;38;1962;8801;1738;32;18;92483;4329;594;8803;34;1629	ABAT;ACACB;ACADM;ACAT1;ALDH6A1;BCKDHB;DBT;DLD;EHHADH;LDHAL6B;MLYCD;PCCA;SUCLA2;SUCLG2
hsa00970	Aminoacyl-tRNA biosynthesis	http://www.kegg.jp/kegg-bin/show_pathway?hsa00970+5859+7407+10056+10667+5917+3735+2193+118672+57505+54938+3035+6301+2617+57176+79587+8565+4141+23438+25973	0.63	1.68	0	0	43	19	5859;7407;10056;10667;5917;3735;2193;118672;57505;54938;3035;6301;2617;57176;79587;8565;4141;23438;25973	AARS2;CARS2;FARS2;FARSA;FARSB;GARS;HARS;HARS2;KARS;MARS;PARS2;PSTK;QARS;RARS;SARS;SARS2;VARS;VARS2;YARS
hsa03420	Nucleotide excision repair	http://www.kegg.jp/kegg-bin/show_pathway?hsa03420+2968+9978+6119+5886+5984+2067+56655+5982+5424+404672+2068+1022+7507+5111+57804+5425+6118+1069+3978+5427+4331	0.62	1.67	0	0	45	21	2968;9978;6119;5886;5984;2067;56655;5982;5424;404672;2068;1022;7507;5111;57804;5425;6118;1069;3978;5427;4331	CDK7;CETN2;ERCC1;ERCC2;GTF2H4;GTF2H5;LIG1;MNAT1;PCNA;POLD1;POLD2;POLD4;POLE2;POLE4;RAD23A;RBX1;RFC2;RFC4;RPA2;RPA3;XPA
hsa04122	Sulfur relay system	http://www.kegg.jp/kegg-bin/show_pathway?hsa04122+81605+90353+4357+348180	0.89	1.66	0	0	8	4	81605;90353;4357;348180	CTU1;CTU2;MPST;URM1
hsa03410	Base excision repair	http://www.kegg.jp/kegg-bin/show_pathway?hsa03410+23583+27343+4913+56655+5424+4595+4350+2237+4968+5111+57804+328+5425+55247+3978+5427	0.67	1.67	0	0	33	16	23583;27343;4913;56655;5424;4595;4350;2237;4968;5111;57804;328;5425;55247;3978;5427	APEX1;FEN1;LIG1;MPG;MUTYH;NEIL3;NTHL1;OGG1;PCNA;POLD1;POLD2;POLD4;POLE2;POLE4;POLL;SMUG1
hsa00983	Drug metabolism	http://www.kegg.jp/kegg-bin/show_pathway?hsa00983+4830+3704+3615+4831+10201+4832+7083+54963+7371+9446+654364+7372+6241+4833+4258+54578+83549+7378+119391+2940+1854+4259	0.56	1.67	0	0	76	22	4830;3704;3615;4831;10201;4832;7083;54963;7371;9446;654364;7372;6241;4833;4258;54578;83549;7378;119391;2940;1854;4259	DUT;GSTA3;GSTO1;GSTO2;IMPDH2;ITPA;MGST2;MGST3;NME1;NME1-NME2;NME2;NME3;NME4;NME6;RRM2;TK1;UCK1;UCK2;UCKL1;UGT1A6;UMPS;UPP1
hsa03060	Protein export	http://www.kegg.jp/kegg-bin/show_pathway?hsa030	0.71	1.65	0.01	0	22	12	23480;196294;58477;10952;6727;6728;28972;9	IMMP1L;OXA1L;SEC11A;SEC11C;SEC61B;SEC61G;SPCS1;SP

		60+23480+196294+58477+10952+6727+6728+28972+90701+9789+23478+5018+6730							0701;9789;23478;5018;6730	CS2;SRP14;SRP19;SRP68;SRPRB
hsa00230	Purine metabolism	http://www.kegg.jp/kegg-bin/show_pathway?hsa00230+30834+5437+9533+4830+30833+3704+5438+3615+5436+4831+10201+5439+471+158+5441+5435+374659+4832+5440+10623+353+1716+318+51082+56655+11164+5424+5433+2987+654364+955+51728+5558+23649+57804+6241+5425+5434+171568+4833+5557+5427+10621	0.51	1.65	0	0	158	43	30834;5437;9533;4830;30833;3704;5438;3615;5436;4831;10201;5439;471;158;5441;5435;374659;4832;5440;10623;353;1716;318;51082;56655;11164;5424;5433;2987;654364;955;51728;5558;23649;57804;6241;5425;5434;171568;4833;5557;5427;10621	ADSL;APRT;ATIC;DGUOK;ENTPD6;GUK1;HDDC3;IMPDH2;ITPA;NME1;NME1-NME2;NME2;NME3;NME4;NME6;NT5C;NUDT2;NUDT5;POLA2;POLD1;POLD2;POLD4;POLE2;POLE4;POLR1C;POLR1D;POLR2D;POLR2E;POLR2F;POLR2G;POLR2H;POLR2I;POLR2J;POLR2K;POLR2L;POLR3C;POLR3F;POLR3H;POLR3K;PRIM1;PRIM2;RRM2;ZNRD1
hsa04260	Cardiac muscle contraction	http://www.kegg.jp/kegg-bin/show_pathway?hsa04260+1340+7381+1345+1350+29796+1337+7388+1329+1537+1351+440567+9377+27089+10975+1347+1327+7384+84701+1349+7134	0.56	1.63	0	0	74	20	1340;7381;1345;1350;29796;1337;7388;1329;1537;1351;440567;9377;27089;10975;1347;1327;7384;84701;1349;7134	COX4I1;COX4I2;COX5A;COX5B;COX6A1;COX6B1;COX6C;COX7A2;COX7B;COX7C;COX8A;CYC1;TNNC1;UQCR10;UQCR11;UQCRB;UQCRC1;UQCRH;UQCRHL;UQCRQ
hsa00480	Glutathione metabolism	http://www.kegg.jp/kegg-bin/show_pathway?hsa00480+6723+2879+79017+9446+2876+2539+51060+6241+2937+2877+6611+4258+5226+119391+2940+2878+4259+2941	0.57	1.6	0.01	0	55	18	6723;2879;79017;9446;2876;2539;51060;6241;2937;2877;6611;4258;5226;119391;2940;2878;4259;2941	G6PD;GGCT;GPX1;GPX2;GPX3;GPX4;GSS;GSTA3;GSTA4;GSTO1;GSTO2;MGST2;MGST3;PGD;RRM2;SMS;SRM;TXNDC12
