# Supplementary material for: SNRPC promotes hepatocellular carcinoma cell motility by inducing epithelial‐mesenchymal transition
Source: FEBS Open Bio. 2021 May 12;11(6):1757–70. doi: 10.1002/2211-5463.13175 (PMC8167856; doi:10.1002/2211-5463.13175)
Supplement: Supplementary file 4 — Table S4. Kinase enrichment of SPRNC coexpressed genes. [file FEB4-11-1757-s002.pdf]

Supplementary Table 4. Kinase enrichment of SPRNC co-expressed genes.

geneSet	description	link	ES	NES	P Value	FDR	size	Leading Edge Num	leadingEdgeId	userId
Kinase_CDK5	cyclin dependent kinase 5	https://www.ncbi.nlm.nih.gov/gene/?term=CDK5	-0.58361	-1.66127	0	0.059058	66	29	10059;5663;54820;1809;5289;5899;23621;1499;273;11076;7498;6774;1742;5925;4846;1808;2308;120892;4780;10725;367;3091;7094;2908;5604;472;22853;9575;4205	AMPH;AR;ATM;BACE1;CLOCK;CTNNB1;DLG4;DNM1L;DPYSL2;DPYSL3;FOXO1;HIF1A;LMTK2;LRRK2;MAP2K1;MEF2A;NDE1;NFAT5;NFE2L2;NOS3;NR3C1;PIK3C3;PSEN1;RALB;RB1;STAT3;TLN1;TPPP;XDH
Kinase_MAPK1	mitogen-activated protein kinase 1	https://www.ncbi.nlm.nih.gov/gene/?term=MAPK1	-0.50338	-1.64079	0	0.064933	197	77	9846;3725;841;7068;6548;4089;5321;6786;23152;10018;4170;3560;1848;5610;2260;2697;3667;6615;1740;2309;1981;5142;11076;10140;2627;5782;4286;3977;9600;2932;2353;10006;5469;6774;6667;1843;4090;55294;23054;5241;2549;2002;80824;3845;9252;596;1612;4155;6654;2099;5594;81565;4040;4093;1387;	ABI1;ADAM17;AR;ATF2;BCL2;BCL2L11;CALD1;CASP8;CIC;CREBBP;DAPK1;DLG2;DUSP1;DUSP16;DUSP6;EGFR;EIF2AK2;EIF4G1;ELK1;EP300;ERG;ESR1;ETS1;FBXW7;FGFR1;FOS;FOXO3;GAB1;GAB2;GATA6;GJA1;GSK3B;GTF2I;HIF1A;IL2RB;IRS1;JUN;KLF8;KRAS;LIFR;LRP6;MAP2K1;MAPK1;MBP;MCL1;MED1;MITF;MYLK;NCOA1

									367;6196;3091;6670;11279;2078;6095;8648;5604;1956;6868;4086;4790;1386;2969;4638;9475;800;10499;2033;2113;6093	;NCOA2;NCOA6;NDEL1;NFKB1;PDE4B;PGR;PITPNM1;PLA2G4A;PTPN12;ROCK1;ROCK2;RORA;RPS6KA2;RPS6KA5;SLC9A1;SMAD1;SMAD4;SMAD5;SMAD9;SNAI1;SOS1;SP1;SP3;STAT3;STIM1;THRB;TOB1;TPPP
Kinase_MAPK7	mitogen-activated protein kinase 7	https://www.ncbi.nlm.nih.gov/gene/?term=MAPK7	-0.67973	-1.67295	0.0053	0.065269	30	13	2005;4208;2353;6774;2002;6446;1612;1739;6196;3091;4790;2113;4205	DAPK1;DLG1;ELK1;ELK4;ETS1;FOS;HIF1A;MEF2A;MEF2C;NFKB1;RPS6KA2;SGK1;STAT3
Kinase_MYLK	myosin light chain kinase	https://www.ncbi.nlm.nih.gov/gene/?term=MYLK	0.846405	1.685046	0.002247	0.068161	10	4	4637;140465;103910;4636	MYL12B;MYL5;MYL6;MYL6B
Kinase_MYLK3	myosin light chain kinase 3	https://www.ncbi.nlm.nih.gov/gene/?term=M	0.846405	1.685046	0.002247	0.068161	10	4	4637;140465;103910;4636	MYL12B;MYL5;MYL6;MYL6B

		YLK3								
Kinase_MYLK4	myosin light chain kinase family member 4	https://www.ncbi.nlm.nih.gov/gene/?term=MYLK4	0.846405	1.685046	0.002247	0.068161	10	4	4637;140465;103910;4636	MYL12B;MYL5;MYL6;MYL6B
Kinase_HCK	HCK proto-oncogene, Src family tyrosine kinase	https://www.ncbi.nlm.nih.gov/gene/?term=HCK	-0.72766	-1.6758	0	0.092851	23	8	6777;8503;2185;5294;25;2889;5290;5295	ABL1;PIK3CA;PIK3CG;PIK3R1;PIK3R3;PTK2B;RAPGEF1;STAT5B
Kinase_SYK	spleen associated tyrosine kinase	https://www.ncbi.nlm.nih.gov/gene/?term=SYK	-0.62151	-1.57225	0.014787	0.097146	35	17	1845;5291;118788;10320;5335;1080;3937;7410;23533;6622;9103;5336;8503;5294;867;5290;5295	CBL;CFTR;DUSP3;FCGR2C;IKZF1;LCP2;PIK3AP1;PIK3CA;PIK3CB;PIK3CG;PIK3R1;PIK3R3;PIK3R5;PLCG1;PLCG2;SNCA;VAV2
Kinase_PDPK1	3-phosphoinositide dependent protein kinase 1	https://www.ncbi.nlm.nih.gov/gene/?term=PDPK1	-0.69088	-1.58253	0.015474	0.106273	21	10	207;6446;4217;5586;5604;10000;5170;5581;3690;2034	AKT1;AKT3;EPAS1;ITGB3;MAP2K1;MAP3K5;PDPK1;PKN2;PRKCE;SGK1
Kinase_FGR	FGR proto-	https://w	-0.78127	-1.57273	0.0055	0.106	12	5	8503;2185;5294;5290;	PIK3CA;PIK3CG;PIK3R1

	oncogene, Src family tyrosine kinase	ww.ncbi.nlm.nih.gov/gene/?term=FGR			87	958			5295	;PIK3R3;PTK2B
Kinase_MAPK3	mitogen-activated protein kinase 3	https://www.ncbi.nlm.nih.gov/gene/?term=MAPK3	-0.49467	-1.58325	0.00173	0.120012	171	66	9846;3725;841;7068;4089;860;23152;10018;4170;3560;1848;4843;2260;2697;3667;2309;1981;10140;2627;5782;4286;3977;2932;2353;10006;5469;83861;6774;6667;4090;55294;5241;2002;5868;3845;9252;9031;596;1612;4155;2099;6886;4040;4093;1387;367;6196;3091;6670;2908;8648;5604;54700;1956;6868;9282;4086;4790;1386;2969;4638;9475;800;10499;2113;6093	ABI1;ADAM17;AR;ATF2;BAZ1B;BCL2;BCL2L11;CALD1;CASP8;CIC;CREBBP;DAPK1;DUSP6;EGFR;EIF4G1;ELK1;ESR1;ETS1;FBXW7;FGFR1;FOS;FOXO3;GAB2;GATA6;GJA1;GSK3B;GTF2I;HIF1A;IL2RB;IRS1;JUN;KRAS;LIFR;LRP6;MAP2K1;MBP;MCL1;MED1;MED14;MITF;MYLK;NCOA1;NCOA2;NFKB1;NOS2;NR3C1;PGR;PTPN12;RAB5A;ROCK1;ROCK2;RPS6KA2;RPS6KA5;RRN3;RSPH3;RUNX2;SMAD1;SMAD4;SMAD5;SMAD9;SP1;SP3;STAT3;TAL1;THRB;TOB1
Kinase_EGFR	epidermal	https://w	-0.64492	-1.69938	0.0018	0.123	46	26	10013;7410;6004;9052	ABCA1;ANXA1;ATM;CB

	growth factor receptor	ww.ncbi.nlm.nih.gov/gene/?term=EGFR			02	801			;152137;301;64855;399694;1499;2035;5336;1398;6777;79705;1399;868;5770;2549;3752;19;867;5604;1956;472;1500;7098	L;CBLB;CCDC50;CRK;CRKL;CTNNB1;CTNND1;EGFR;EPB41;FAM129B;GAB1;GPRC5A;HDAC6;KCND3;LRRK1;MAP2K1;PLCG2;PTPN1;RGS16;SHC4;STAT5B;TLR3;VAV2
Kinase_CSNK1D	casein kinase 1 delta	https://www.ncbi.nlm.nih.gov/gene/?term=CSNK1D	-0.64609	-1.5271	0.014414	0.125233	25	13	999;23621;2697;4193;1499;8864;2736;1633;2737;10413;4040;3091;324	APC;BACE1;CDH1;CTNNB1;DCK;GJA1;GLI2;GLI3;HIF1A;LRP6;MDM2;PER2;YAP1
Kinase_DYRK1A	dual specificity tyrosine phosphorylation regulated kinase 1A	https://www.ncbi.nlm.nih.gov/gene/?term=DYRK1A	-0.74777	-1.52998	0.008993	0.130118	14	7	1827;23451;91750;2308;10253;5430;1859	DYRK1A;FOXO1;LIN52;POLR2A;RCAN1;SF3B1;SPRY2
Kinase_PAK3	p21 (RAC1) activated kinase 3	https://www.ncbi.nlm.nih.gov/gene/?term=PAK3	-0.81339	-1.53452	0.01341	0.131284	9	2	5604;4638	MAP2K1;MYLK

Kinase_PAK5	p21 (RAC1) activated kinase 5	https://www.ncbi.nlm.nih.gov/gene/?term=PAK5	-0.81339	-1.53452	0.01341	0.131284	9	2	5604;4638	MAP2K1;MYLK
Kinase_PAK2	p21 (RAC1) activated kinase 2	https://www.ncbi.nlm.nih.gov/gene/?term=PAK2	-0.68475	-1.58431	0.012987	0.137066	21	10	340156;3725;840;1981;5597;25;80243;5604;4638;800	ABL1;CALD1;CASP7;EIF4G1;JUN;MAP2K1;MAPK6;MYLK;MYLK4;PREX2
Kinase_PIK3CA	phosphatidylinositol-4,5-bisphosphate 3-kinase catalytic subunit alpha	https://www.ncbi.nlm.nih.gov/gene/?term=PIK3CA	-0.79607	-1.53819	0.01518	0.147919	10	6	208;2932;207;10000;5170;5295	AKT1;AKT2;AKT3;GSK3B;PDPK1;PIK3R1
Kinase_PIK3CG	phosphatidylinositol-4,5-bisphosphate 3-kinase catalytic subunit gamma	https://www.ncbi.nlm.nih.gov/gene/?term=PIK3CG	-0.76899	-1.47402	0.03125	0.164571	10	6	208;2932;207;5294;10000;5170	AKT1;AKT2;AKT3;GSK3B;PDPK1;PIK3CG
Kinase_SGK1	serum/gluco	https://w	-0.65166	-1.47731	0.0369	0.165	21	12	23385;3551;4792;5744	CREB1;FBXW7;FOXO3;I

	corticoid regulated kinase 1	ww.ncbi.nlm.nih.gov/gene/?term=SGK1				971			7;1385;2309;6416;55294;253260;65125;5594;200576	KBKB;MAP2K4;MAPK1;NCSTN;NDRG2;NFKBIA;PIKFYVE;RICTOR;WNK1
Kinase_MAPK9	mitogen-activated protein kinase 9	https://www.ncbi.nlm.nih.gov/gene/?term=MAPK9	-0.57259	-1.47763	0.021164	0.173638	41	23	3726;8660;5663;3725;2354;64689;4303;3667;2309;10140;9927;2353;6774;334;2355;122953;2002;9031;351;2308;54700;4773;1386	APLP2;APP;ATF2;BAZ1B;ELK1;FOS;FOSB;FOSL2;FOXO1;FOXO3;FOXO4;GORASP1;IRS1;IRS2;JDP2;JUN;JUNB;MFN2;NFATC2;PSEN1;RRN3;STAT3;TOB1
Kinase_RPS6KA4	ribosomal protein S6 kinase A4	https://www.ncbi.nlm.nih.gov/gene/?term=RPS6KA4	0.714299	1.597018	0.015419	0.175633	19	7	3020;468;3150;8352;8354;3021;8358	ATF4;H3F3A;H3F3B;HIST1H3B;HIST1H3C;HIST1H3I;HMGN1
Kinase_PIK3CD	phosphatidylinositol-4,5-bisphosphate 3-kinase catalytic subunit delta	https://www.ncbi.nlm.nih.gov/gene/?term=PIK3CD	-0.79478	-1.46451	0.029963	0.177903	8	5	208;2932;207;10000;5170	AKT1;AKT2;AKT3;GSK3B;PDPK1
Kinase_PAK6	p21 (RAC1) activated	https://www.ncbi.	-0.73452	-1.49377	0.036538	0.178201	12	3	367;5604;4638	AR;MAP2K1;MYLK

	kinase 6	nlm.nih.gov/gene/?term=PAK6								
Kinase_NUAK1	NUAK family kinase 1	https://www.ncbi.nlm.nih.gov/gene/?term=NUAK1	-0.9609	-1.47803	0.001832	0.182245	4	2	4659;9113	LATS1;PPP1R12A
Kinase_MAPK10	mitogen-activated protein kinase 10	https://www.ncbi.nlm.nih.gov/gene/?term=MAPK10	-0.62816	-1.45825	0.034904	0.185439	25	16	3726;8660;3725;2354;4303;3667;2309;1822;2353;334;2355;2002;351;2308;4773;1386	APLP2;APP;ATF2;ATN1;ELK1;FOS;FOSB;FOSL2;FOXO1;FOXO3;FOXO4;IRS1;IRS2;JUN;JUNB;NFATC2
Kinase_JAK2	Janus kinase 2	https://www.ncbi.nlm.nih.gov/gene/?term=JAK2	-0.62803	-1.47952	0.034672	0.18886	23	7	6777;6774;4217;5594;3717;2969;5781	GTF2I;JAK2;MAP3K5;MAPK1;PTPN11;STAT3;STAT5B
Kinase_TAF1	TATA-box binding protein associated	https://www.ncbi.nlm.nih.gov/gene/	-0.97032	-1.43545	0.005254	0.196019	3	1	2957	GTF2A1

	factor 1	?term=TAF1							
Kinase_RAF1	Raf-1 proto-oncogene, serine/threonine kinase	https://www.ncbi.nlm.nih.gov/gene/?term=RAF1	-0.67006	-1.48087	0.025362	0.19722	19	6	5594;4659;5604;7071;4790;5599	KLF10;MAP2K1;MAPK1;MAPK8;NFKB1;PPP1R12A
Kinase_AURKB	aurora kinase B	https://www.ncbi.nlm.nih.gov/gene/?term=AURKB	0.508658	1.522171	0.006424	0.284905	87	33	6204;6132;332;10726;9212;3326;3151;3020;113130;11004;29101;6144;1058;11186;79080;10403;103910;3856;8352;5347;79980;24137;10014;8354;55577;5935;79016;157313;27316;55143;29127;3021;8358	AURKB;BIRC5;CCDC86;CDCA2;CDCA5;CDCA8;CENPA;DDA1;DSN1;H3F3A;H3F3B;HDAC5;HIST1H3B;HIST1H3C;HIST1H3I;HMGN2;HSP90AB1;KIF2C;KIF4A;KRT8;MYL12B;NAGK;NDC80;NUDC;PLK1;RACGAP1;RASSF1;RBM3;RBMX;RPL21;RPL8;RPS10;SSU72
Kinase_MYLK2	myosin light chain kinase 2	https://www.ncbi.nlm.nih.gov/gene/?term=MYLK2	0.751744	1.536764	0.042129	0.290192	11	4	4637;140465;103910;4636	MYL12B;MYL5;MYL6;MYL6B
Kinase_BUB1	BUB1	https://w	0.846287	1.450261	0.0593	0.512	5	2	51529;991	ANAPC11;CDC20

	mitotic checkpoint serine/threonine kinase	ww.ncbi.nlm.nih.gov/gene/?term=BUB1			41	318				
Kinase_PKMYT1	protein kinase, membrane associated tyrosine/threonine 1	https://www.ncbi.nlm.nih.gov/gene/?term=PKMYT1	0.901513	1.403437	0.067538	0.681191	3	3	891;9133;983	CCNB1;CCNB2;CDK1
Kinase_RPS6KA6	ribosomal protein S6 kinase A6	https://www.ncbi.nlm.nih.gov/gene/?term=RPS6KA6	0.683195	1.235382	0.258278	0.781459	7	4	3265;9088;468;699	ATF4;BUB1;HRAS;PKMYT1
Kinase_CSNK2A1	casein kinase 2 alpha 1	https://www.ncbi.nlm.nih.gov/gene/?term=CSNK2A1	0.386992	1.31272	0.012346	0.804231	255	78	1460;25855;90480;23;1936;51154;9141;332;3178;8894;1616;3326;5303;4150;8125;3020;10541;995;4605;9616;51053;10728;9049;55502;55611;5888;8841;1978;23028;3183;64151;11124;997;10921;1649	ABCF1;AIP;ANP32A;ANP32B;ARRB2;BCAM;BIRC5;BRMS1;CALM3;CDC25B;CDC25C;CDC34;CDC37;CDK1;CEBPD;CSNK2B;DAXX;DDIT3;EEF1D;EIF2S2;EIF4EBP1;EXOSC9;FAF1;GADD45GIP1;GGA1;GMNN;H3F3A;

									;6722;3150;103910;23435;5883;11140;6741;8363;983;8352;8364;5905;10062;808;10381;8354;9894;994;6804;51447;4793;3065;3978;5393;8366;4171;6520;6732;3021;8358;7298;7515;9129;4059;9656;409;7518;6749;26088;8367;23397;54926;1052	H3F3B;HDAC1;HDAC3;HES6;HIST1H3B;HIST1H3C;HIST1H3I;HIST1H4B;HIST1H4C;HIST1H4E;HIST1H4J;HMGN1;HNRNPA1;HNRNPC;HSP90AB1;IP6K2;KDM1A;LIG1;MAZ;MCM2;MDC1;MRTO4;MYBL2;MYL12B;NCAPG;NCAPH;NFKBIB;NR1H3;OTUB1;PDCD5;PIN4;PRPF3;PTGES3;RAD51;RAD9A;RANGAP1;RNF7;RNPS1;SLC3A2;SRF;SRPK1;SSB;SSRP1;STX1A;TARDBP;TELO2;TUBB3;TYMS;UBE2R2;XRCC1;XRCC4
Kinase_ATR	ATR serine/threonine kinase	https://www.ncbi.nlm.nih.gov/gene/?term=ATR	0.404061	1.164754	0.176471	0.813056	66	27	2873;84126;1616;3014;4869;51659;2193;11186;7507;55215;1111;4172;1869;5883;11200;6118;2130;50485;64421;63922;65108;4171;2177;9656;7517;1029;64852	ATRIP;CDKN2A;CHEK1;CHEK2;CHTF18;DAXX;DCLRE1C;E2F1;EWSR1;FANCD2;FANCI;FARSA;GINS2;GPS1;H2AFX;MARCKSL1;MCM2;MCM3;MDC1;NPM1;RAD9A;RASSF1;RPA2;SMARCAL

										1;TUT1;XPA;XRCC3
Kinase_CHEK1	checkpoint kinase 1	https://www.ncbi.nlm.nih.gov/gene/?term=CHEK1	0.371179	1.174065	0.099307	0.813778	130	30	1460;6143;79171;22913;2547;10775;6137;6742;9212;10155;4000;3020;3833;995;5528;5605;51530;10969;1819;23223;5888;11186;993;4904;3838;2178;1111;4172;4174;2237	AURKB;CDC25A;CDC25C;CHEK1;CSNK2B;DRG2;EBNA1BP2;FANCE;FEN1;H3F3A;KIFC1;KPNA2;LMNA;MAP2K2;MCM3;MCM5;POP4;PPP2R5D;RAD51;RALY;RASSF1;RBM42;RPL13;RPL19;RRP12;SSBP1;TRIM28;XRCC6;YBX1;ZC3HC1
Kinase_LMTK2	lemur tyrosine kinase 2	https://www.ncbi.nlm.nih.gov/gene/?term=LMTK2	0.786932	1.183819	0.307203	0.814477	3	1	5499	PPP1CA
Kinase_PBK	PDZ binding kinase	https://www.ncbi.nlm.nih.gov/gene/?term=PBK	0.772451	1.2371	0.270742	0.814703	4	1	5052	PRDX1
Kinase_DAPK1	death associated protein	https://www.ncbi.nlm.nih.g	0.585281	1.349982	0.101322	0.822795	21	10	23521;6194;3297;5300;4172;103910;4636;6804;1613;5595	DAPK3;HSF1;MAPK3;MCM3;MYL12B;MYL5;PIN1;RPL13A;RPS6;STX1

	kinase 1	ov/gene/?term=DAPK1								A
Kinase_MAPKAPK2	mitogen-activated protein kinase-activated protein kinase 2	https://www.ncbi.nlm.nih.gov/gene/?term=MAPKAPK2	0.498159	1.295728	0.109409	0.823335	36	10	26574;3315;10155;3297;995;9532;3875;6722;5347;994	AATF;BAG2;CDC25B;CDC25C;HSF1;HSPB1;KRT18;PLK1;SRF;TRIM28
Kinase_MAP2K3	mitogen-activated protein kinase kinase 3	https://www.ncbi.nlm.nih.gov/gene/?term=MAP2K3	0.734092	1.212498	0.285383	0.823592	5	3	1432;5603;6300	MAPK12;MAPK13;MAPK14
Kinase_CSNK2A2	casein kinase 2 alpha 2	https://www.ncbi.nlm.nih.gov/gene/?term=CSNK2A2	0.570022	1.200309	0.240618	0.828457	14	6	1460;3297;11140;6732;7298;7515	CDC37;CSNK2B;HSF1;SRPK1;TYMS;XRCC1
Kinase_UHMK1	U2AF homology motif kinase 1	https://www.ncbi.nlm.nih.gov/gene/	0.787279	1.187197	0.318681	0.836979	3	1	3925	STMN1

		?term=UHMK1								
Kinase_WEE1	WEE1 G2 checkpoint kinase	https://www.ncbi.nlm.nih.gov/gene/?term=WEE1	0.796933	1.318664	0.142169	0.838856	5	3	891;9133;983	CCNB1;CCNB2;CDK1
Kinase_SRPK1	SRSF protein kinase 1	https://www.ncbi.nlm.nih.gov/gene/?term=SRPK1	0.863468	1.362269	0.111814	0.840206	3	3	5936;9939;6732	RBM4;RBM8A;SRPK1
Kinase_MAP2K6	mitogen-activated protein kinase kinase 6	https://www.ncbi.nlm.nih.gov/gene/?term=MAP2K6	0.697075	1.252585	0.246411	0.847074	7	4	1432;5603;3299;6300	HSF4;MAPK12;MAPK13;MAPK14
Kinase_CDK2	cyclin dependent kinase 2	https://www.ncbi.nlm.nih.gov/gene/?term=CDK2	0.362908	1.240075	0.024814	0.849204	278	65	1460;51529;51070;51035;3615;26121;6135;22913;26284;6188;28973;2547;3159;84126;10726;55929;124944;283899;6136;3835;81620;	AARS2;ANAPC11;ANAPC5;ANAPC7;ATRIP;C17orf49;C6orf106;CDC20;CDC25C;CDC6;CDK7;CDT1;CHEK1;CRTC2;CSNK2B;DBNL;DLGAP5;DMAP1

									6949;991;27343;5499;7083;3925;995;4605;27043;51434;4869;4176;51530;112398;5451;9775;23421;1022;81890;57505;84289;1111;28988;29920;4172;6625;1869;3024;23557;5883;6829;7283;6241;200186;51433;6118;22974;2146;64771;3978;1870;990;28987;9787	;E2F1;E2F2;EGLN2;EIF4A3;ERAL1;EZH2;HIST1H1A;HMGA1;IMPDH2;ING5;INO80E;ITGB3BP;KIF22;LIG1;MCM3;MCM7;MRPS18B;MYBL2;NOB1;NOSIP;NPM1;NUDC;PELP1;POLL;POU2F1;PPP1CA;PRPF31;PYCR2;QTRT1;RAD9A;RALY;RPA2;RPL11;RPL12;RPS3;RRM2;SNAPIN;SNRNP70;STMN1;SUPT5H;TCOF1;TK1;TPX2;TUBG1;UBXN1;XRCC6;ZC3HC1
Kinase_MARK3	microtubule affinity regulating kinase 3	https://www.ncbi.nlm.nih.gov/gene/?term=MARK3	0.876361	1.329568	0.121413	0.855667	3	1	995	CDC25C
Kinase_NEK2	NIMA related kinase 2	https://www.ncbi.nlm.nih.gov/gene/?term=N	0.67955	1.270492	0.216092	0.877189	8	7	4869;10403;5501;4751;2175;11190;116840	CEP250;CNTROB;FANCA;NDC80;NEK2;NPM1;PPP1CC

		EK2								
