# Supplementary material for: SNRPC promotes hepatocellular carcinoma cell motility by inducing epithelial‐mesenchymal transition
Source: FEBS Open Bio. 2021 May 12;11(6):1757–70. doi: 10.1002/2211-5463.13175 (PMC8167856; doi:10.1002/2211-5463.13175)
Supplement: Supplementary file 5 — Table S5. miRNA enrichment of SPRNC coexpressed genes. [file FEB4-11-1757-s004.pdf]

**Supplementary Table 5. miRNA enrichment of SPRNC co-expressed genes.**

| geneSet | link | ES | NES | P Value | FDR | size | Leading Edge Num | leadingEdgeId | userId |
|---|---|---|---|---|---|---|---|---|---|
| AAGCCAT,MIR-135A,MIR-135B | http://www.broadinstitute.org/gsea/msigdb/cards/AAGCCAT,MIR-135A,MIR-135B | -0.55263 | -1.87627 | 0 | 0 | 310 | 129 | 776;3750;284252;51230;23452;9874;9671;55008;657;23152;9334;2869;259230;51444;1452;9792;26960;6477;7532;23621;84280;1740;2824;8831;84189;6059;220441;7095;9686;121536;4208;571;288;285382;11178;55167;9228;57162;5915;5229;91;8539;1605;158471;23005;283149;1112;168667;23683;55691;162466;8622;9317;493;6667;64750;7046;57161;3839;122953;115207;57509;2002;57089;2444;80031;901;54602;9849;84132;1285;8874;10928;395;7813;84220;373;80216;9462;8997;2042;29068;22982;2308;23179;8243;91694;9958;27115;4306;166968;54014;54545;11234;23174;90102;3624;23411;22856;29110;3091;6670;9690;3717;4121;9648;9444;10210;9459;6497;23035;8648;10 | ABCE1;ACVR1B;AEBP2;AKT3;ALPK1;ANGPTL2;ANK3;API5;ARHGAP6;ARHGEF6;ARHGEF7;ATP2B4;B4GALT5;BACE1;BACH1;BCL9L;BMPER;BMPR1A;BMPR2;BRWD1;BTBD10;C3orf70;CACNA1D;CCNG2;CHSY1;CIC;COL4A3;CSNK1A1;DAG1;DDX3X;DIP2C;DLG2;DLGAP2;ELK1;ELK3;ENTPD4;ENTPD7;EPHA3;EVI5;FCHO2;FOXN3;FOXO1;FRK;FRMD4A;GCC2;GPM6B;GRK5;HERC6;HIF1A;HPS5;IL6ST;INHBA;JAK2;JDP2;KALRN;KCND1;KCTD1;KCTD12;KPNA3;LMBRD2;LONRF1;LZTS1;MAN1A1;MAPKBP1;MEF2C;MIER3;MSL2;MTMR12;MTUS1;NBEA;NCOA1;NDFIP2;NR3C2;PATL1;PDE7B;PDE8B;PELI1;PELI2;PGGT1B;PHF20;PHLDB2;PHLPP2;PHOSPHO1;PRKD3;PRUNE2;PTER;QKI;RALBP1;RAPGEF6;RARB;RASAL2;RBAK;RGL1;RGPD5;RNF138;RNF152;ROCK2;SEC62;SEMA6D;SERTAD2;SGMS1;SIA |

| | | | | | | | | | |
|---|---|---|---|---|---|---|---|---|---|
| | | | | | | | | 000;1654;122809;92255;115548;7776;9583;219988;6651;54822;9475;57786;659;51735;54861;3572;2004 | H1;SIRT1;SKI;SLITRK6;SMC1A;SMURF2;SNRK;SOCS4;SON;SP1;SP3;SYNGAP1;TBK1;TGFBR1;TLK1;TOPORS;TRIM23;TRPM7;UBE3C;USP15;USP42;VGLL4;WSCD2;YWHAG;ZBTB44;ZCCHC14;ZNF236;ZNF518A |
| CACTTTG,MIR-520G,MIR-520H | http://www.broadinstitute.org/gsea/msigdb/cards/CACTTTG,MIR-520G,MIR-520H | -0.57613 | -1.88109 | 0 | 0 | 219 | 97 | 813;6515;894;58476;389524;26039;1147;4293;56137;29843;1026;64067;6310;253738;841;25909;9842;55284;7224;11278;6299;25822;860;50717;23152;166336;26960;54910;56135;8729;5530;51727;7273;1785;1952;8013;25852;161742;4091;7067;85456;160335;81848;4929;388403;80000;23768;51592;10773;3839;323;9988;5783;51199;9863;51341;3836;56134;23637;1982;116984;23314;6653;7126;23516;2308;1756;7091;51742;2185;122830;51306;19;4092;23774;9306;3091;27125;11057;64895;10618;8239;6497;57515;1859;27327;5576;22890;80829;22862;2113;2034;5049;54726;23499;80205;219333 | ABCA1;ABHD2;AFF4;AHCTF1;APBB2;ARAP2;ARID4B;ARMC8;ATXN1;BRD1;CALU;CASP8;CCND2;CDKN1A;CELSR2;CHD9;CHUK;CIC;CMPK1;DCAF8;DMD;DMTF1;DNAJB5;DNM2;DYRK1A;EBF3;EIF4G2;EPAS1;ETS1;FAM13B;FLRT2;FNDC3A;FOXO1;GBF1;GREB1L;GTF2IRD2B;HIF1A;KLF12;KPNA1;KPNA3;MACF1;MAGI2;MAP3K9;NAA30;NBEA;NIN;NPAS3;NR4A2;NR4A3;OTUD4;PAFAH1B2;PAPOLG;PCDHA12;PCDHAC1;PCDHAC2;PLEKHM1;PPP3CA;PRICKLE2;PRKAR2A;PTK2B;PTPN13;RABGAP1;RUNX2;SALL1;SATB2;SEMA4C;SENP1;SERINC1;SKI;SLC2A3;SLC39A14;SMAD6;SMAD7;SOCS6;SORL1;SPRED1;SPRY4;SS18L1;TGOLN2;THRA;TLE4;TMTC2;TNFAIP1;TNKS1BP1;TNRC6A;TP53INP2;TRIM33;TRPC5;TTN;UBE2W;US |

| | | | | | | | | | |
|---|---|---|---|---|---|---|---|---|---|
| | | | | | | | | | P12;USP9X;YPEL2;ZBTB1;ZBTB6;ZBTB7A;ZFP91 |
| GTGCCTT,MIR-506 | http://www.broadinstitute.org/gsea/msigdb/cards/GTGCCTT,MIR-506 | -0.52703 | -1.88718 | 0 | 0 | 674 | 229 | 8728;259230;83742;6845;9354;9949;9792;143279;5989;196;56256;23299;56135;257415;5587;9414;5066;51201;1462;50862;64328;1289;84061;5142;51727;51099;54834;55512;1969;2332;10040;51339;84189;57403;1949;5933;1785;9683;2627;5782;84188;80055;8013;6416;182;2683;5048;155435;345557;4026;8879;11178;4286;161742;4628;23189;22998;6789;83593;4548;255252;5900;6856;55119;79837;26130;203228;266812;60468;8763;10730;56650;11127;6774;10773;10788;493;1959;6667;5306;7046;22941;65117;4090;845;22850;8322;6330;3839;81537;8829;538;4643;148867;8828;1827;84295;8826;223;57610;51631;55623;114134;8031;4784;56134;22904;57658;23235;255967;80031;8555;83607;9472;494470;10928;115;7337;55568;9794;781 | ABCA1;ABHD5;ADAM19;ADCY9;ADNP2;AFF4;AHR;AKAP6;ALDH9A1;AMMECR1;AMMECR1L;ANTXR2;AP1G1;AR;ARFIP1;ARHGEF6;ARL5B;ATF7IP;ATP2B4;ATP7A;B4GALT1;BACH2;BICD2;BRWD1;BTBD7;C2orf69;C9orf72;CALCOCO1;CASQ2;CBL;CD164;CDC14B;CHSY1;CLDND1;CLOCK;CMPK1;COL4A1;COL5A1;CTNND1;DACT1;DAPK1;DCAF5;DDX3X;DDX6;DNAJB14;DNM2;DPY19L3;EAF1;EDEM1;EDNRB;EFNB3;EGR2;ELK3;EPHA2;EPS8;ERN1;ESYT2;EVI5;FAM122A;FAM133B;FAM76B;FAR1;FBXO38;FCHO2;FCHSD2;FMR1;FZD4;G3BP2;GALNT10;GAPVD1;GATA6;GDAP2;GPATCH8;HECTD2;HIP1;HIPK1;HIVEP1;HIVEP2;HIVEP3;IQGAP1;IQGAP2;ITGB1;ITSN2;JAG1;KANK1;KIAA1958;KIF16B;KIF3A;KLF13;KLHL24;KPNA3;LEMD3;LIMCH1;LPP;LRIG1;LRRC57;LUC7L2;LZTS1;MAGT1;MAML1;MAP2K4;MARVELD1;MIB1;MITF;MTR;MYH10;MYH9;MYLK;MYO10;M |

| | | | | | | | | | |
|---|---|---|---|---|---|---|---|---|---|
| | | | | | | | | 3;2054;55300;205327;6840;26018;6446;9655;84669;339745;1612;4651;85403;123606;7091;147179;2059;4306;55727;1910;57045;1282;57630;4926;54014;81545;9873;23592;6886;9695;158405;19;10725;55614;89796;3688;5780;143684;27107;118429;1656;22856;57534;65059;51621;677;84068;9908;59269;367;27236;27125;7094;2908;9895;9525;23131;55031;8816;151011;54521;9444;55729;221079;51449;867;164;4627;7109;9459;4863;6497;57477;9517;1654;3096;57713;57488;219749;54800;114882;3097;116224;114885;50618;7799;23060;115548;56916;54464;5007;84640;4638;1500;147991;7003;204851;5781;6239;79982;23112;23001;10019;10194;7182;3092;2081;9575;2004 | YO1E;N4BP1;NAP1L5;NAV1;NCOA4;NFAT5;NFIX;NIPA1;NPAT;NR2C2;NR3C1;NR3C2;NR4A3;NRP1;NRP2;NUMA1;OSBP;OSBPL11;OSBPL8;PAFAH1B1;PAM;PAN3;PCDHAC1;PCDHAC2;PCYOX1;PDE4B;PGAP1;PHF6;PI4K2B;PIP4K2C;PITPNA;PLCXD3;PRDM2;PRKD1;PRPF38B;PTPN11;PTPN12;PTPN9;QKI;RAB22A;RALBP1;RALGDS;RANBP10;RAPH1;RASSF5;RBL1;RBM33;RCAN1;RFX1;RNF141;RNF165;RREB1;RSRC2;SBNO2;SCN4B;SEMA6D;SEPT10;SERTAD2;SERTAD4;SFMBT2;SGK1;SGMS1;SGPL1;SGPP1;SH2B3;SH3RF1;SHANK2;SHROOM4;SIK2;SKI;SLC10A7;SLC2A13;SLC30A7;SLITRK6;SMAD5;SMARCAD1;SMPD3;SOCS5;SP1;SPOPL;SPRED1;SPTLC2;STAT3;STK4;STX2;SVIL;SYPL1;TAL1;TEAD1;TECPR2;TGFBR1;THUMPD1;TJP2;TLE4;TLN1;TNRC6B;TOM1L1;TRAPPC10;TSHZ1;TWSG1;UBE3A;UBE4A;USP32;USP38;USP47;VAMP7;VCAN;VPS4B;WDFY3;WDR44;WIPF2;XPO4;XRN1;YME1L1;ZBTB11;ZBTB6;ZDHHC2;ZFP36L1;ZNF25;ZNF609 |

| | | | | | | | | | |
|---|---|---|---|---|---|---|---|---|---|
| TGGTGCT,MIR-29A,MIR-29B,MIR-29C | http://www.broadinstitute.org/gsea/msigdb/cards/TGGTGCT,MIR-29A,MIR-29B,MIR-29C | -0.54488 | -1.90394 | 0 | 0 | 479 | 201 | 23142;3340;8050;6310;605;149420;5979;1277;154796;4774;28951;3778;3174;92999;80232;83648;11278;94015;153090;89795;166336;83690;4313;5549;84541;8728;51444;140685;54413;196;9924;3479;1734;56135;3038;1831;9715;405;5187;8455;57561;858;8493;57118;4135;10776;3556;51727;56261;9612;55512;6059;81566;8242;5155;55288;10891;26052;80055;23503;25852;3363;7528;26959;288;23429;23368;2861;5915;10979;170692;1284;55334;1605;2353;23005;9935;283149;5813;84206;90634;63916;81573;388403;60468;80000;23473;1281;160418;493;5159;6667;5306;55294;25921;55074;57590;9754;25897;55970;55654;23476;8863;22992;57609;23114;2549;7092;8031;4784;56134;64795;83872;83607;84132;1285;494470;89797;357;8723;84220;59338;91947;1317;1293;6446;51496;7126;390;11160;144 | ABCE1;ADAM19;ADAMTS18;AFF4;AHR;AKAP13;AKT3;AMMECR1L;AMOT;ANK3;ANKRD13C;AP1G1;AP4E1;ARMC8;ARNT;ARPP19;ARRDC3;ARRDC4;ATP2B4;ATRN;ATXN1;BACH2;BCL7A;BCL9L;BIRC6;BRD4;BRWD1;BRWD3;C5orf24;CAMK1D;CAPN7;CAV2;CCNT2;CHSY1;CMPK1;COL15A1;COL1A1;COL3A1;COL4A1;COL4A2;COL4A3;COL4A4;COL6A3;CPEB3;CPNE8;CRISPLD1;CSRNP2;CTDSPL2;CTNND1;DAB2IP;DAG1;DBT;DCUN1D4;DDX3X;DENND1B;DICER1;DIO2;DIP2B;DNM3;ELF2;ELMO2;EPHA3;EPS15;ERLIN2;FAM126B;FAM131B;FAM13B;FAM167A;FBN1;FBXW7;FEM1B;FERMT2;FOS;FOXJ2;GAB1;GCC2;GNG12;GOPC;GPCPD1;GPR37;GREB1L;HAS3;HBP1;HMCN1;HNF4G;HS3ST3B1;HTR7;IGF1;IL1RAP;INO80D;IREB2;ITGB1;KBTBD8;KCNMA1;KDM2A;KDM5C;KIAA0355;KLF12;LUZP1;MAFB;MAP6;MAPKBP1;MBLAC2;MEX3B;MFAP3;MMP2;N4BP2L1;NAV1;NAV2;NAV3;NCOA4;NCOR2;NDST1;NFASC;NFAT5;NFIA;NFIX;NKTR;NL |

| | | | | | | | | | |
|---|---|---|---|---|---|---|---|---|---|
| | | | | | | | | 108;8503;2042;55810;84919;163486;27314;2060;1286;1306;51028;1282;54891;54014;10735;23431;153364;51306;1629;10725;89796;3688;144402;7798;4820;10116;7538;22856;10252;677;57120;1998;55014;2200;6018;57605;254065;7543;27125;285172;134553;3658;9648;4238;23405;9444;9953;164;22849;23013;6497;10000;9517;1654;9710;114885;23060;81846;29966;80829;5007;1500;11214;5295;23001;905;23345;257218;57448;54726;5980 | GN3;OSBP;OSBPL11;OTUD4;OXR1;PAN2;PCDHAC1;PCDHAC2;PDGFB;PDGFRB;PDHX;PDIK1L;PER1;PER3;PGAP1;PIK3R1;PIK3R3;PITPNA;PITPNM2;PLEKHA1;PPARGC1A;PPM1D;PPP1R13B;PPP1R15B;PRELP;PRICKLE2;PURA;QKI;RAB30;RARB;RET;REV3L;RGPD5;RHOT1;RLF;RMND5A;RND3;RNF138;RNF165;RNF19A;RYBP;SBF2;SGK1;SHPRH;SHROOM2;SKI;SLC31A1;SLC39A9;SMPD3;SNX4;SP1;SPEN;SPRY1;SPTLC2;SPTY2D1;STAG2;STARD8;STRN3;STX17;SYNE1;TLL1;TMEM127;TMTC3;TNFAIP1;TRIB2;TSC22D3;TTYH2;USP42;VPS36;WDFY1;WDFY3;WDR26;YPEL2;YY1;ZBTB46;ZBTB47;ZDHHC5;ZFP36;ZFP36L1;ZFP91;ZFX;ZFYVE26;ZNF609 |
| ACACTGG,MIR-199A,MIR-199B | http://www.broadinstitute.org/gsea/msigdb/cards/ACACT | -0.62799 | -1.95843 | 0 | 0 | 146 | 72 | 10890;4779;857;26278;3551;55327;23213;5887;11278;25822;23193;196441;51271;55082;23293;64844;57018;6096;94134;57419;26249;56963;7565;10659;10138;10891;10513;6789;4287;2932;6444;9935;113251;79577;23095;8322;64398;257397;5420; | ADAMTSL1;ADAMTSL3;ALS2;AP1G1;APPBP2;ARGLU1;ARHGAP12;ARHGAP21;ARID1A;ATXN3;ATXN7;CAV1;CBL;CCNL1;CDC73;CELF2;CLIP1;DDX3X;DNAJB5;ETS1;FZD4;GANAB;GSK3B;HIF1A;HIPK2;HMCN1;IKBKB;KIAA0355;KIF1B;KL;KLF12;KLHL3;LARP4;LIN7C;MAFB;MARCH7;MPP5 |

| | | | | | | | | | |
|---|---|---|---|---|---|---|---|---|---|
| | GG,MIR-199A, MIR-199B | | | | | | | 57610;23353;57679;253959;8289;255967;83872;92949;26994;9365;57188;65267;152006;54877;23411;6249;5780;5903;3091;28996;5495;54521;23038;867;164;4627;1654;9710;6314;80829;57584;10499;2113 | ;MYH9;NCOA2;NFE2L1;PAN3;PODXL;PPARGC1A;PPM1B;PTPN9;RAB10;RAD23B;RALGAPA1;RANBP10;RANBP2;RGMA;RNF11;RNF38;RORB;SACS;SGCD;SIRT1;SLC24A3;SMG6;STK4;SULF1;SUN1;TAB3;UBAP1;WDR44;WDTC1;WNK3;YAF2;ZCCHC2;ZFC3H1;ZFP91;ZNF17 |
| TGCTGCT,MIR-15A,MIR-16,MIR-15B,MIR-195,MIR-424,MIR-497 | http://www.broadinstitute.org/gsea/msigdb/cards/TGCTGCT,MIR-15A,MIR-16,MIR-15B,MIR-195,MIR- | -0.55627 | -1.96493 | 0 | 0 | 548 | 198 | 6845;51444;196441;140685;863;9949;8473;10479;51271;6477;56135;11244;257415;23621;1831;3037;54897;79869;11276;54815;94134;131566;55205;8554;84910;8493;627;23168;22824;9823;9053;3759;63982;8434;5774;9683;11176;7290;1812;256356;1602;389941;8013;2683;5048;9201;372;57154;23429;161742;219527;55605;9529;55664;55161;5915;9497;10979;170692;81848;56977;7049;83593;6444;6542;23051;6856;6812;283149;5813;22844;63916;255488;60468;80000;8763;65986;5306;23095;50488;1399;4090;2747;55294;27244;51232;6330;660;154214;8 | ABHD13;ACSL1;ACTR2;ADAMTS18;ADAMTSL3;AFF4;AHCYL2;AKAP11;AKT3;AMMECR1;ANO3;APP;ARCN1;ARHGAP12;ARHGAP20;ARHGAP5;ARHGEF9;ARID1A;ARMCX2;B4GALT1;BACE1;BACH2;BAG5;BAZ2A;BCL2;BCL2L2;BCL9L;BDNF;BMX;C1QL3;CAB39;CAMSAP1;CASZ1;CBFA2T3;CCNT2;CD164;CDC37L1;CDK17;CLOCK;COBLL1;COL12A1;CPEB2;CPEB3;CPSF7;CRIM1;CRKL;DACH1;DCAF5;DCBLD2;DCLK1;DDX3X;DLL1;DMTF1;DRD1;DYNC1LI2;EIF3A;ELMO2;FAM133B;FBXW7;FERMT2;FRMPD1;GATAD2A;GHR;GK5;GLUD2;GOLGA1;GPATCH8;GREB1L;HAS2;HELZ;HIRA;HSPA4L;IGF2R;ITPR1;KALRN;KBTBD4;KCNJ2;KIF1B;KIF21A;LATS2; |

| | | | | | | | | | |
|---|---|---|---|---|---|---|---|---|---|
| | 424,MIR-497 | | | | | | | 4945;22837;23229;5069;9988;8828;257397;5166;5090;9204;1783;7092;8289;84870;56134;80031;84132;2180;23321;157922;22841;8661;4215;253260;59338;23314;28514;91746;26018;9860;6446;596;57188;2800;351;8671;8997;9931;5930;29068;57826;5727;7091;5494;9958;7750;9880;51719;6814;54877;1303;4040;4092;89796;3482;7798;57534;10097;9306;599;6645;54941;8578;27125;57569;54665;27253;5194;23131;8816;23038;9444;2690;22849;22911;23013;23035;3708;29761;5604;10000;1654;1130;132864;5128;55066;153020;130507;55709;23362;54778;23382;23060;6651;4638;26524;5295;10611;11215;23112;394;7586;905;23345;862;9575;8867;54726;54861 | LRIG1;LRIG2;LRP6;LRRC55;LUZP1;LYST;MAP2K1;MAP3K3;MAP7;MIB1;MINK1;MYLK;N4BP1;NAV1;NR4A3;NRP2;OGT;OTUD4;PAFAH1B1;PAPPA;PBX3;PCDH17;PCDHAC1;PCDHAC2;PDK4;PDLIM5;PDPR;PEX13;PHLPP2;PIAS1;PIK3R1;PITPNA;PLEKHA1;PPM1A;PPM1D;PSD3;PTCH1;PTPN3;PURA;QKI;RAB11FIP2;RAP2C;RARB;RASGEF1B;RASSF5;RBBP6;RECK;RICTOR;RNF111;RNF125;RNF138;RNF144B;RNF217;RSBN1;RSPO3;RTF1;RUNX1T1;RYBP;SATB2;SCARF1;SCN4B;SEMA6D;SESN1;SGCD;SGK1;SIAH1;SLC4A4;SLC4A7;SLC7A2;SLC9A6;SMAD5;SMAD7;SMURF1;SNRK;SNTB2;SOCS6;SON;SPEN;SPRED1;SPRY4;STOX2;STXBP1;STXBP3;SYNE1;SYNJ1;SYNRG;SYPL1;TAB3;TGFBR3;TLE4;TLL1;TMEM33;TMEM87B;TNRC6B;TRIM2;TSC22D3;UBAP1;UBR3;USP15;USP25;USP42;VAMP7;WDR47;WDTC1;YTHDC1;ZBTB10;ZBTB39;ZBTB44;ZBTB46;ZCCHC2;ZFC3H1;ZHX1;ZHX3;ZKSCAN1;ZMYM2;ZMYM6;ZNF532;ZNF609 |

| | | | | | | | | | |
|---|---|---|---|---|---|---|---|---|---|
| ACATTCC,MIR-1,MIR-206 | http://www.broadinstitute.org/gsea/msigdb/cards/ACATTCC,MIR-1,MIR-206 | -0.58819 | -1.97476 | 0 | 0 | 274 | 129 | 6760;254170;169611;605;2354;149420;4804;55327;22797;29855;23213;55108;11231;8674;10231;51444;9354;8473;3479;3214;1655;2697;64786;10090;79869;7705;84255;57561;627;3895;392255;6668;11076;11342;10370;2335;7095;1665;9686;5048;372;29979;7078;161742;5218;5915;7049;55193;4929;23005;9590;6542;5469;114826;388403;266812;60468;10773;55294;9554;636;8829;538;4643;84295;54816;57580;1742;1389;285636;84870;196528;23032;80031;5756;83607;1285;494470;23321;64393;6422;205327;253260;523;23317;57659;85403;55917;23369;147179;51320;152006;56262;145567;8301;51621;677;29767;23270;152485;8871;8491;54665;5793;1948;9444;9953;867;164;23002;222194;23035;5921;23527;80351;3097;23362;26040;1182;23041;7776;4638;22862;7328;10611;2113;11215;168850;953 | ACAP2;AKAP11;AKAP12;AMMECR1L;AP1G1;ARCN1;ARID2;ARRDC3;ATP6V1A;ATP7A;BACH2;BAG4;BCL7A;BDNF;BICD1;BSDC1;C2orf69;C5orf51;CBL;CDK14;CITED2;CLCN3;COL4A3;CPSF7;CREBL2;CTTNBP2NL;DAAM1;DDX5;DHX15;DLG4;DNAJC13;EAF1;EFNB2;ETS1;FBXO33;FBXW7;FN1;FNDC3A;FOSB;GDF6;GJA1;HIVEP2;HOXB4;HS3ST3B1;IGF1;KLF13;KTN1;LIN7C;LRRC8A;MAP4K3;MAPKBP1;MED1;MEX3C;MON2;MYLK;MYO1E;NAP1L5;NGFR;NR4A2;NRP1;OGT;OLFML2A;PAFAH1B1;PBRM1;PDIK1L;PDLIM5;PHF6;PHLPP2;PICALM;PREX1;PSD3;PTPRG;PUM2;QKI;RARB;RASA1;RCAN2;RICTOR;RNF13;RNF138;RNF165;RNF38;RSBN1;RSBN1L;RSPO3;SEC22B;SEC62;SEC63;SEMA6D;SETBP1;SFRP1;SLC37A3;SLC7A2;SMYD4;SP2;SPRED1;SS18;SULF1;SYNJ2;TBC1D15;TFEC;TGFBR3;TIMP3;TMOD2;TNKS2;TPPP;TRIM2;TSPYL4;TTC7B;TWF1;UBE2H;UBE4A;UBN1;UBQLN1;USP33;UST;VAMP4;VGLL4;WIPF2;YPEL2;ZBTB4;ZBTB6;ZFP36L |

| | | | | | | | | | |
|---|---|---|---|---|---|---|---|---|---|
| | | | | | | | | 0 | 1;ZMAT3;ZNF146;ZNF236;ZNF280D;ZNF800;ZNF827 |
| AATGTGA,MIR-23A,MIR-23B | http://www.broadinstitute.org/gsea/msigdb/cards/AATGTGA,MIR-23A,MIR-23B | -0.5701 | -1.98052 | 0 | 0 | 389 | 194 | 81609;9865;26118;7039;57526;201176;79571;9069;7155;526;3340;6310;253738;7980;2354;9883;5887;80155;3174;840;10586;860;54874;9874;7323;140885;5332;55749;85464;4303;665;2869;8728;863;9949;142685;639;3214;4360;7532;2697;3037;64786;154807;1740;4094;23168;55626;5142;51704;9891;57532;2332;10659;6387;10891;1602;26052;1665;64101;4208;345557;4026;382;5325;957;5500;23429;23368;55167;9228;6789;23673;7049;1398;9590;51362;2744;29969;760;90411;266812;6777;56980;337876;1960;91833;5306;22941;138151;9749;64398;1456;257397;23300;23054;96459;51199;55366;10664;3836;57509;4883;54556;6304;80031;54602;4041;64854;9794;22841;8661;4215;205327;23314;26018;355;6446;11160;8671;339745;6397;2906 | ADAM19;AFF1;AKAP12;AMBRA1;AMMECR1;ANKRD50;ARF6;ARFIP1;ARHGAP20;ARHGAP27;ASB15;ATMIN;ATP11B;ATP6V1B2;ATXN1;AUTS2;BNIP3L;BRWD1;BTAF1;BTBD7;C2orf69;CA2;CAB39;CASP7;CBFA2T3;CCAR1;CCNT2;CDC40;CELF2;CHSY3;CLCN3;CLDN12;CPEB2;CPEB4;CREBZF;CRK;CSNK1G3;CTCF;CXCL12;DACH1;DENND1B;DHX15;DIP2C;DLG2;DLGAP2;DMXL1;DNM3;EBF3;EGR3;EIF3A;ELF2;ENTPD5;EPS15;ERLIN2;FAM13B;FAS;FBN1;FBXO11;FMR1;FNBP1L;FNIP1;FOSB;FOXO4;G3BP2;GCC1;GJA1;GLS;GLYR1;GPRC5B;GRK5;HAS2;HMGXB4;HNF4G;HOXB4;IL6R;ING3;IRF2;KDM4A;KDM6A;KLF3;KPNA1;LGR4;LPP;LRIG1;LRP5;LRRC4;MAB21L2;MAF;MAGI1;MAML1;MAP3K3;MAP3K5;MCFD2;MDFIC;MEF2C;MEX3C;MPP5;MRC1;MSL2;MTUS1;NAA15;NACC2;NAP1L5;NCOA1;NCOA6;NDFIP2;NDST1;NIN;NPR3;NUAK1;NUFIP2;OSBPL8;PAPOLG;PCDH19; |

| | | | | | | | | | |
|---|---|---|---|---|---|---|---|---|---|
| | | | | | | | | 8;4217;22982;163486;23369;65267;2060;51320;2185;55727;7750;51014;51719;54014;6505;9682;7403;58517;54877;10042;51306;145567;51274;8301;10253;3570;29072;389136;84656;9306;1998;55236;55014;2200;26053;57616;58487;9908;10484;27236;7871;57569;9044;54665;7048;3660;6935;23200;80204;9444;64895;331;4659;8648;7150;57182;132864;80351;114882;27327;23090;5529;1182;4299;7003;1657;23112;80315;905;862;9223;8867 | PDE4B;PHACTR2;PICALM;PITPNA;PLAGL1;PLCB4;PLCXD3;POM121;PPARGC1A;PPP1CB;PPP1R12A;PPP1R13B;PPP2R5E;PRDM1;PRDM10;PTK2B;PUM2;QKI;RAB11FIP2;RAD23B;RBM25;RSBN1;RTF1;RUNX1T1;RUNX2;RYBP;SATB1;SATB2;SEC14L1;SEC23A;SEMA6D;SETD2;SGK1;SHANK2;SIRPA;SLC1A1;SLC4A4;SLMAP;SNX27;SOCS6;SPOPL;SPRY2;SSH2;STAT5B;STK4;STX12;STX17;SYNJ1;TAB3;TBC1D15;TEAD1;TFPI2;TGFA;TGFBR2;TGFBR3;TLK1;TMED7;TNKS2;TNRC6A;TNRC6B;TOP1;TOP2B;TRIL;TSHZ3;TTC7B;UBA6;UBE2D3;USP46;VGLL3;VKORC1L1;WDR20;WNK3;WSB1;XIAP;YWHAG;ZBTB44;ZCCHC2;ZEB1;ZMYM2;ZNF423 |
| TTGGAGA,MIR-515-5P,MIR-519E | http://www.broadinstitute.org/gsea/msigdb/cards/TTGGAG | -0.64548 | -1.99043 | 0 | 0 | 130 | 53 | 5156;9706;8455;1740;57561;3842;4781;10138;256356;91801;8879;9698;4287;80000;8826;11167;4983;3836;8289;10299;6334;55125;6671;8671;83604;29068;57826;54918;26091;51719;123879;6867;4853;22856;677;147339;26053;9908;27125;54665;70 | AFF4;AHCYL2;ALKBH8;ARID1A;ARRDC3;ATRN;ATXN3;AUTS2;C18orf25;CAB39;CEP192;CHSY1;CMTM6;CPEB2;DCUN1D3;DDX3X;DLG2;EPC2;ETS1;FSTL1;G3BP2;GK5;GREB1L;HERC4;IQGAP1;ITSN1;KPNA1;MARCH6;MOSPD2;NCOA1;NFIB;NOTCH2;OPHN1;PDGFRA;PUM1;RAP2C;RBL2;RSB |

| | | | | | | | | | |
|---|---|---|---|---|---|---|---|---|---|
| | A,MIR-515-5P,MIR-519E | | | | | | | 48;6453;26122;158747;8648;1654;132864;23382;22890;80829;7328;2113;5934 | N1;SCN8A;SGPL1;SLC4A4;SP4;TACC1;TGFBR2;TMEM47;TNPO1;UBE2H;ULK2;YAF2;ZBTB1;ZBTB44;ZFP36L1;ZFP91 |
| AGCACTT,MIR-93,MIR-302A,MIR-302B,MIR-302C,MIR-302D,MIR-372,MIR-373,MIR-520E,MIR-520A,MIR-526B,MIR-520B,MIR-520C,MIR-520D | http://www.broadinstitute.org/gsea/msigdb/cards/AGCACTT,MIR-93,MIR-302A, MIR-302B, MIR-302C, MIR-302D, MIR-372,MIR- | -0.59258 | -1.99334 | 0 | 0 | 321 | 120 | 4978;22893;26249;56963;57459;1969;51334;57532;10659;57403;5933;4781;84084;79665;1812;8013;7095;23503;23094;121536;4208;9848;55188;9770;9135;26468;4929;1398;5900;5813;81573;388403;55691;10773;4756;65117;50488;9828;152273;51232;55074;9988;5090;84295;222236;51631;57509;5534;56134;255967;5756;23637;114879;84132;8897;64854;9252;9794;8690;7126;57659;351;25976;55810;59277;23179;7091;51742;2321;1910;166968;22884;23390;10628;2099;84312;10150;57169;6886;19;27107;10766;51621;57616;5870;56929;6049;55279;54665;80818;9639;91754;3660;54617;80204;23414;8850;23035;5926;80351;114882;5638;51277;130507;196527;9321;80829;5488 | ABCA1;AEBP2;ANKRD13C;ANO6;APP;ARHGEF10;ARHGEF17;ARID4A;ARID4B;BAHD1;BRMS1L;CELF2;CRIM1;CRK;DCUN1D1;DHX40;DMTF1;DNAJC27;DRD1;EDNRB;EPAS1;EPHA2;ESR1;FAM13C;FBXO11;FEM1C;FGD5;FLT1;FNDC3A;FOXJ2;FRMD4A;GATAD2B;INO80;IRF2;JRKL;KAT2B;KLF13;KLHL3;LATS2;LHX6;LUC7L2;MAML1;MARCH8;MBNL2;MEF2C;MFAP3L;MIER3;MINK1;MTMR3;MTUS1;NAPEPLD;NEK9;NEO1;NFIB;NR2C2;NR4A2;NR4A3;NTN4;NUFIP2;OPCML;OSBPL5;OSBPL8;OTUD4;OXR1;PAN3;PBX3;PCDHAC2;PHF2;PHF6;PHLPP2;PPP3R1;PRR16;PRRG1;PURA;RAB22A;RAB6A;RAB6C;RABEP1;RABGAP1;RALGDS;RASSF2;RBL1;RGL1;RGMA;RIC8B;RNF6;RPS6KA5;RSBN1;RSRC2;SEC62;SIPA1L3;TAL1;TBC1D8B;TIPARP;TLE4;TNFAIP1;TNKS2;TOB2;TRIP11;TSHZ3;TWF1;TXNIP;UBR |

| | | | | | | | | | |
|---|---|---|---|---|---|---|---|---|---|
| | 373,MIR-520E, MIR-520A, MIR-526B, MIR-520B, MIR-520C, MIR-520D | | | | | | | 5;26524;220965;22862;5253;168850;10771;220972;54165;2034;7586;7182;54726 | 3;USP42;USP46;WDR37;YPEL2;ZBTB11;ZBTB4;ZBTB6;ZDHHC17;ZFP91;ZFPM2;ZFYVE26;ZKSCAN1;ZMYND11;ZNF436;ZNF654;ZNF800;ZNFX1 |
| GCACCTT,MIR-18A,MIR-18B | http://www.broadinstitute.org/gsea/msigdb/cards/GCACCTT,MIR-18A,MIR-18B | -0.65684 | -2.00484 | 0 | 0 | 112 | 45 | 55288;3815;27319;121536;6789;55691;51232;55852;22992;2549;619279;1389;23637;23321;23677;22841;9655;57659;29068;22982;23122;8470;2099;9873;10725;79813;89796;677;57616;3091;254065;79618;2908;3660;23037;8648;57182;23362;6651;5253;10905;23112;27252;55252;54622 | AEBP2;ANKRD50;ARL15;ASXL2;BHLHE22;BRWD3;CLASP2;CREBL2;CRIM1;DIP2C;EHMT1;ESR1;FCHSD2;FRMD4A;GAB1;HIF1A;HMBOX1;IRF2;KDM2A;KIT;KLHL20;MAN1A2;NAV1;NCOA1;NFAT5;NR3C1;PDZD2;PHF2;PSD3;RAB11FIP2;RABGAP1;RHOT1;SH3BP4;SOCS5;SON;SORBS2;STK4;TEX2;TNRC6B;TRIM2;TSHZ3;ZBTB4;ZBTB44;ZFP36L1;ZNF704 |
| TACTTGA,MIR- | http://w | -0.60047 | -2.02506 | 0 | 0 | 285 | 145 | 6815;8715;4325;23136;123041; | ABHD2;ACADSB;ACBD5;ACVR1C;A |

| | | | | | | | | | |
|---|---|---|---|---|---|---|---|---|---|
| 26A,MIR-26B | ww.broadinstitute.org/gsea/msigdb/cards/TACTTGA,MIR-26A,MIR-26B | | | | | | | 4139;9132;55114;894;2245;36;130399;9644;219654;5999;253943;7326;8050;26278;9883;154796;28951;80155;57507;4089;3356;6299;1300;54829;3082;114799;85464;57552;84541;8728;4170;9949;3479;53981;3038;9857;5156;9882;9706;5743;4345;273;5074;23168;10776;1969;3759;10659;6103;10140;54407;182;1983;57522;4286;4810;55161;79776;9497;2932;57634;5813;84206;266812;56650;113251;9317;5209;57161;23196;5783;257397;84295;96459;57610;57679;55366;5534;5597;196528;255967;10135;5756;54602;9472;54510;157922;1982;8661;4664;8671;8997;1612;59277;23122;57826;55917;65267;9958;55727;26091;54014;6505;152503;90102;4052;7764;8204;286410;57534;775;6018;58487;9908;6196;7543;6049;26122;91452;80204;11057;8239;8553;29761;27327;130507;4086;324;114885;7071;23362 | DAM19;AKAP6;ALS2;AMMECR1;AMOT;AMPH;APC;ARHGAP17;ARID2;ARPP19;ASPN;ATF2;ATM;ATP11C;BAZ2B;BHLHE40;BRWD1;BTBD7;CACNA1C;CAMSAP1;CCND2;CD200;CELF2;CEP350;CLASP2;CLDND1;COL10A1;CPSF2;CREBZF;CTTNBP2NL;DAPK1;DMXL1;EIF3A;EIF4G2;EIF5;EP400;EPB41L3;EPC2;EPHA2;ESCO1;FAM120A;FBXO11;FGD1;FNIP1;G3BP2;GSK3B;HAS3;HERC4;HGF;HTR2A;IGF1;JAG1;KALRN;KBTBD8;KCNJ2;KCNQ4;KLF10;LARP4;LGR4;LTBP1;MAN2A1;MAPK6;MARK1;MCL1;MED13L;MEX3B;MIB1;MITF;MMP16;NAA15;NAB1;NAMPT;NAP1L5;NCEH1;NDFIP2;NHS;NOL4;NRIP1;NTN4;OSBPL11;OTUD4;PAN3;PAWR;PCDH18;PDGFRA;PDHX;PELI2;PFKFB3;PHF6;PHLDB2;POM121;PPP3R1;PSD3;PTER;PTGS2;PTPN13;PURA;RANBP10;RAP2C;RGS4;RLF;RNF6;RPGR;RPS6KA2;RTF1;SACS;SALL1;SH3D19;SH3PXD2A;SLC1A1;SLC24A4;SLC38A2;SLC4A4;SLC4A7;SMAD1;SMAD4;SRGAP1;SSH2;STYX;TAB3;TBC1D4;TMEM33;T |

| | | | | | | | | | |
|---|---|---|---|---|---|---|---|---|---|
| | | | | | | | | ;29994;1386;472;4124;23389;58499;1657;23112;54726 | NRC6A;TNRC6B;TOB1;TRIB2;TWF1;UBE2G1;UBR3;ULK2;USP15;USP25;USP9X;WNK3;YTHDF3;ZCCHC24;ZFHX4;ZFX;ZNF217;ZNF462;ZNF608 |
| CAGTATT,MIR-200B,MIR-200C,MIR-429 | http://www.broadinstitute.org/gsea/msigdb/cards/CAGTATT,MIR-200B,MIR-200C,MIR-429 | -0.58048 | -2.0377 | 0 | 0 | 442 | 221 | 9972;5577;813;9132;3398;50807;2245;6305;9782;3751;23015;8076;64778;9644;55089;219654;9465;1266;10914;10238;9865;253943;5567;4035;254170;3340;10777;6310;149420;54206;29883;23213;4774;55284;121441;9522;11278;22846;84064;25822;9508;79365;135112;89795;9962;85464;9957;1039;143279;639;2624;6533;8473;6563;4478;6477;84251;7532;9857;115294;9706;5066;55205;26249;9969;23168;55626;4908;57532;2332;3759;51339;8434;10370;10140;55145;6567;55288;2335;1602;54407;267;5310;26060;6529;121536;9202;5099;288;29979;55167;23189;10979;1605;10769;23164;9815;84206;3188;388403;266812;51592;1960;64750;1843;1399;9828;55294;27244;6710;6439 | ADAMTS3;ADIPOR2;AEBP2;AKAP2;AKAP7;AMBRA1;AMFR;AMMECR1L;AMOTL2;ANK3;ANKRD28;APPL1;ARHGAP20;ARHGEF17;ARID2;ARID4B;ARPP21;ASAP1;ATP2A2;ATRX;ATXN1;BAZ2B;BHLHE41;BMI1;BPTF;BRMS1L;BRWD1;CAB39;CACNA1C;CALU;CDH11;CDK17;CDR2;CEP350;CHD9;CITED2;CLASP1;CLASP2;CLIC4;CLIP1;CNN3;CNOT7;CNTN4;CRKL;CSNK1G3;CTDSPL2;CYP1B1;DACH1;DACT1;DAG1;DCAF7;DCUN1D1;DDX3X;DENND5A;DLC1;DNAJB5;DUSP1;EFNB2;EGR3;ELF2;EPS8;ERRFI1;ETS1;EVI5;FAM76B;FBXO33;FBXW11;FBXW2;FBXW7;FERMT2;FGD1;FHL1;FLI1;FMR1;FN1;FNDC3B;GABPA;GATA2;GEM;GIT2;GLI3;GOLGA8A;GPM6A;HDHD2;HECTD2;HIPK1;HMBOX1;HNRNPH2;HS3ST1;ID2;ITPR1;KANK1;KAT2B;KCND2;KCNJ2;KCNQ4;KIAA0355;KLF10;KLF12;KLF9;KLHL3;K |

| | | | | | | | | | |
|---|---|---|---|---|---|---|---|---|---|
| | | | | | | | | 8;648;55074;51421;1456;8828;2273;114299;2823;5783;84295;57610;51199;23047;2313;57509;687;23011;196528;2737;26190;80031;79602;83607;1545;23321;3845;1009;7813;23258;4664;2669;5334;51496;5358;2186;390;4008;123606;23122;57826;23179;23369;51742;51320;2059;51455;10395;51719;54014;84312;153364;23291;25932;7764;6249;152330;143684;8204;775;4247;1998;55236;29110;6018;6196;10484;9839;57569;79618;8491;2908;376940;1948;5495;2551;23243;6935;488;9444;23414;331;8850;22864;57515;3708;29761;1654;5170;9710;5128;151987;114885;7071;29994;445815;546;9819;56916;7003;10499;204851;23332;2113;23001;54165;200576;8867;54726;80205;11217 | RAS;LMO7;LRP1;MAP4K3;MATR3;MBLAC2;MED13;MEX3B;MEX3C;MFAP5;MGAT2;MPP5;MPRIP;MSL2;MSN;MTUS1;NAB1;NAP1L5;NAV3;NCOA2;NCOA7;NDST1;NEDD1;NFIA;NIN;NIPA1;NR3C1;NRIP1;NRP2;NTF3;NUFIP2;NUP153;OGT;OSBPL11;OTUD4;OXR1;PALM2;PALM2-AKAP2;PAM;PAPOLA;PCDH7;PCMTD1;PDIK1L;PDPK1;PDS5B;PHF6;PIKFYVE;PKD1;PLCL1;PLK2;PLS3;PPM1B;PPP4R2;PRDM1;PRKACB;PRKAR2B;PTPN13;PUM2;QKI;R3HDM2;RAB21;RANBP10;RAP2C;RECK;REV1;RGL1;RHOT1;RLF;RND3;RPS6KA2;RTF1;SBF1;SCAMP1;SEC23A;SEMA6D;SERINC1;SESN1;SGIP1;SH3PXD2A;SIAH1;SLC14A1;SLC16A2;SLC23A2;SLC38A2;SLC38A4;SLC6A1;SLC6A6;SMARCAD1;SMURF2;SPTB;SSH2;SULF1;SYNJ1;TBK1;TEAD1;THAP1;TOB1;TRIL;TRIM2;TRIM33;TSC22D2;UBA6;UBE2W;UBQLN1;ULK2;USP25;VASH1;WDFY3;XIAP;YPEL2;YTHDF3;YWHAG;ZC3H6;ZCCHC24;ZEB1;ZEB2;ZFPM2;ZMYM4;ZNF217;ZNF532 |

| | | | | | | | | | |
|---|---|---|---|---|---|---|---|---|---|
| ACTGAAA,MIR-30A-3P,MIR-30E-3P | http://www.broadinstitute.org/gsea/msigdb/cards/ACTGAAA,MIR-30A-3P,MIR-30E-3P | -0.63308 | -2.04209 | 0 | 0 | 185 | 78 | 1740;7763;11080;51809;50862;10776;392255;57532;10659;10140;7290;10512;51100;9778;4208;382;26045;1958;55664;4810;5915;3738;55193;51362;5813;3188;55294;1743;1456;25777;22887;2252;5597;5756;3845;6422;253260;83604;8028;5562;26091;23431;51306;1303;2140;389136;118429;9372;1387;9839;7543;6670;2908;134553;9444;64895;867;164;5926;10142;1654;23534;7150;51277;324;114885;5529;23382;115548;9819;2033;79982;23112;2034;7182;862;57448;2004 | AHCYL2;AKAP9;ANTXR2;AP1G1;AP4E1;APC;ARF6;ARID4A;ARPP19;BIRC6;C5orf24;CBL;CDC37L1;CDC40;CELF2;COL12A1;CREBBP;CSNK1G3;DDX3X;DLG2;DLST;DNAJB14;DNAJB4;DNAJC27;EGR1;ELK3;EP300;EPAS1;EYA3;FAM13B;FBXW7;FCHO2;FGF7;FOXJ3;GALNT7;GDF6;HERC4;HIRA;HNRNPH2;KCNA3;KIAA0232;KRAS;LRRTM2;MAPK6;MEF2C;MLLT10;NHS;NR2C2;NR3C1;NUFIP2;OSBPL11;PAPOLG;PBRM1;PPP2R5E;PRKAA1;PURA;QKI;RARB;RICTOR;RNF141;RUNX1T1;SEMA3C;SFRP1;SH3GLB1;SP3;SUN2;TMEM47;TNPO3;TNRC6B;TOB1;TOP1;TSC22D2;TWF1;VGLL3;ZEB2;ZFAND5;ZFX;ZFYVE9 |
| ATGCTGC,MIR-103,MIR-107 | http://www.broadinstitute.org/gsea/msigdb/cards/ATGCTGC,MIR- | -0.62699 | -2.04358 | 0 | 0 | 200 | 81 | 627;5142;56261;63982;9683;11176;1952;64225;389941;25852;1983;57522;23429;219527;55605;5218;223082;7049;83593;23051;55691;60468;80000;65986;5306;2747;55294;8828;25777;3836;1742;5411;1783;84870;4154;23235;5756;84132;22841;23314;28514;9467;57188;55810;57 | ADAMTSL3;AFF4;AKAP2;ANO3;ARMC8;ATL2;BACH2;BAZ2A;BDNF;C1QL3;CAB39;CACNA2D1;CDK14;CELSR2;CPEB3;DICER1;DLG4;DLL1;DYNC1LI2;EIF5;FBXW7;FOXJ2;FRMD4A;GLUD2;GPATCH8;GPCPD1;GREB1L;HERC2;KIF21A;KPNA1;LATS2;LCOR;LRRC55;MBNL1;MIB1;N4BP1;NDEL1;NF1;NFAT5;NKTR;NR2C2;NRP2;O |

| | | | | | | | | | |
|---|---|---|---|---|---|---|---|---|---|
| | 103,MIR-107 | | | | | | | 826;7091;7750;9880;51719;152006;54877;81565;10725;4820;23264;57534;6645;54941;27125;54665;23131;781;23405;23262;4763;23414;22849;8924;84458;130507;23362;445815;26524;5295;23112;7182;862;8867;54726;54861;11217 | TUD4;PALM2-AKAP2;PDE4B;PIK3R1;PITPNA;PNN;PPIP5K2;PSD3;RAB11FIP2;RAP2C;RASSF5;RNF125;RNF38;RSBN1;RSPO3;RUNX1T1;RYBP;SATB2;SH3BP5;SIK2;SNRK;SNTB2;SRGAP1;SUN2;SYNJ1;TGFBR3;TLE4;TNRC6B;TWF1;UBR3;USP42;ZBTB10;ZBTB39;ZC3H7B;ZCCHC2;ZFPM2;ZHX3;ZMYM2;ZNRF2 |
| ATGTAGC,MIR-221,MIR-222 | http://www.broadinstitute.org/gsea/msigdb/cards/ATGTAGC,MIR-221,MIR-222 | -0.66026 | -2.056 | 0 | 0 | 133 | 64 | 56137;4294;6310;29116;3344;22846;23452;5602;51312;9949;143279;8473;5861;56135;7532;54511;54897;405;140460;4908;2332;10891;1602;9686;85406;10144;285382;7078;55167;10979;3977;2353;25853;5813;266812;317649;22941;25777;10664;253959;5534;8289;56134;2114;80031;284273;4651;166968;2099;55614;144402;58487;9839;3660;9444;23414;222194;1859;22862;6938;204851;5253;2113;54165 | AMMECR1;ANGPTL2;ARID1A;ARNT;ASB7;ATXN1;C3orf70;CASZ1;CPNE8;CREBZF;CTCF;DACH1;DCAF12;DCUN1D1;DNAJC14;DYRK1A;EIF4E3;ESR1;ETS1;ETS2;FAM13A;FERMT2;FMR1;FNDC3A;FOS;FOXN2;HECTD2;HIPK1;HMGCLL1;IRF2;KIF16B;LIFR;MAP3K10;MAPK10;MIER3;MSL2;MYLIP;MYO10;NAP1L5;NTF3;OGT;PCDHA12;PCDHAC1;PCDHAC2;PHF2;PPARGC1A;PPP3R1;PURA;QKI;RAB1A;RALGAPA1;RSBN1L;SEMA6D;SHANK2;SLC25A37;SUN2;TCF12;TIMP3;VASH1;VGLL4;YWHAG;ZADH2;ZEB2;ZFPM2 |
| TGTTTAC,MIR- | http://w | -0.58469 | -2.0723 | 0 | 0 | 545 | 260 | 7514;51141;9213;4684;8453;25 | ABL1;ACAP2;ACTC1;ACVR1;ADAM |

| | | | | | | | | | |
|---|---|---|---|---|---|---|---|---|---|
| 30A-5P,MIR-30C,MIR-30D,MIR-30B,MIR-30E-5P | ww.broadinstitute.org/gsea/msigdb/cards/TGTTTAC,MIR-30A-5P,MIR-30C,MIR-30D,MIR-30B,MIR-30E-5P | | | | | | | 778;813;81553;84002;6305;9782;54541;56999;57718;9331;70;9644;5916;219654;9729;3768;10914;84196;4335;4974;253943;57526;2590;7072;84333;10890;6310;2589;253738;8754;9734;133522;55818;9021;55327;29855;8577;5887;57507;79739;1305;64412;9522;860;60481;9508;23351;51130;10018;114799;388;23315;57147;85464;22847;665;2591;8728;196441;1452;639;1734;2697;646;3760;84280;56995;64786;154807;9715;3667;6615;7763;2035;22893;51809;5074;4135;8864;286133;9612;8731;57532;10659;7148;4781;79665;7786;817;54407;1909;23503;10144;345557;4026;90;7078;4628;55664;4810;5915;80267;3977;56977;64839;1605;158471;4515;4929;57620;6856;84206;22844;388403;57763;6787;56650;113251;783;1399;85440;868;84632;51133;3839;9749;8829;144165;122953;51421;8828;114299;578 | 19;ADAM9;ADAMTS3;ADAMTS9;AFAP1L2;AFF4;AHNAK;AMOTL2;ANKRA2;ANKRD17;ARHGEF6;ARID4A;ARID4B;ASB3;ASCC3;ATP2A2;ATXN1;B3GNT5;B4GALT6;BAHD1;BAZ2B;BCL2L11;BCOR;BNC1;BNC2;BNIP3L;BRD1;BRWD1;BTBD10;BTBD7;CACNB2;CALU;CAMK2D;CBLB;CCNT2;CCPG1;CDC37L1;CELF2;CLDND1;COL13A1;CPEB2;CPEB4;CPNE8;CRKL;CSNK1A1;CUL2;DAG1;DCUN1D3;DDIT4;DHX40;DIO2;DIP2B;DMD;DNAJC13;DOCK7;DPYSL2;DSTYK;EAF1;EBF3;EDEM3;EDNRA;EDNRB;ELOVL5;EPB41;EPC2;ERG;ESCO1;FAM131B;FAM13A;FAM160B1;FAM49A;FBXL17;FBXO34;FCHO2;FGD6;FNDC3A;FRK;FRMD6;FRMPD1;FYCO1;GALNT1;GALNT2;GALNT3;GALNT7;GIGYF2;GJA1;GLCCI1;GNAO1;GOLGA4;GZF1;HDAC9;HECW2;HERC2;IKZF2;INSIG2;IRS1;ITGB3;ITSN1;JDP2;KCNJ12;KCNJ3;KCTD3;KDM3A;KHNYN;KIAA0355;KIAA0408;KIAA2026;KLF10;KLF9;KLHL20;KLHL24;KPNA3;KRAS;LARP4;LIFR;LIN7C;LPP;LYST;MAGI2;MA |

| | | | | | | | | | |
|---|---|---|---|---|---|---|---|---|---|
| | | | | | | | | 3;257397;84295;57610;54796;9863;51341;4734;57609;5411;22807;5534;2775;2444;254827;687;5756;10299;6334;494470;3845;9794;2054;10973;23314;91746;23317;6443;9649;55785;113263;6671;54880;1808;26058;83604;85403;4217;57826;23179;1756;79026;51742;57700;55727;51455;1910;166968;23390;57630;9880;54014;84146;10735;25977;54877;10150;25;81565;122786;989;23174;123879;8301;10725;23411;23774;144402;8204;65059;29767;10484;7543;27125;9690;6453;27253;340481;55031;11069;26122;54521;57520;488;9444;4659;8633;23013;2078;9459;5926;5921;23527;79443;8924;1130;54800;132864;9236;27327;9710;7071;29994;115548;55238;55030;84102;3690;6651;54822;23335;57551;2803;26057;22862;10905;23112;158358;27252;80315;905;54726;160760 | ML1;MAN1A2;MAP3K12;MAP3K5;MAP6;MARCH6;MATR3;MBNL2;MEX3B;MIER3;MNT;MTCP1;MYH10;NAALADL2;NCAM1;NCOR2;NDEL1;NECAP1;NEDD4;NEK4;NFAT5;NFIB;NHS;NR4A2;NRIP1;NRP1;NRP2;NUFIP2;OMG;OTUD4;PALM2;PAPOLA;PAWR;PCDH17;PCDH19;PCGF5;PER2;PHACTR2;PHF6;PICALM;PLCXD3;PNN;PPARGC1B;PPP1R12A;PPP3R1;PPP4R4;PPTC7;PRDM1;PRICKLE1;PRUNE2;PTPN13;QKI;RAB10;RAD23B;RALGPS1;RANBP10;RAP2C;RAPGEF4;RAPH1;RARB;RARG;RASA1;REV1;RGL1;RHOB;RNF165;RNMT;RUNX2;SATB2;SBF1;SCAMP1;SCARA5;SCN8A;SCYL3;SEC23A;SEPT7;SGCB;SH3PXD2A;SH3RF1;SIRT1;SLC38A2;SLC38A7;SLC41A2;SLC9A8;SNAI1;SOCS3;SON;SP4;SPEN;SSH2;STAG2;STIM2;STOX2;STX2;SYPL1;TAB3;TAOK1;TBC1D15;TIA1;TIMP3;TMEFF1;TMEM47;TMOD2;TNRC6A;TNRC6B;TNXB;TRPM7;TTLL7;TULP4;TWF1;UBE3C;UBN1;UNC5C;USP47;USP48;VKORC1L1;WDR44;WDR7;XPO1;XPR1;YPEL2;YTHDC1;YT |

| | | | | | | | | | |
|---|---|---|---|---|---|---|---|---|---|
| | | | | | | | | | HDF3;ZBTB39;ZBTB7A;ZCCHC14;ZCCHC2;ZCCHC24;ZDHHC17;ZDHHC21;ZFAND5;ZFC3H1;ZFX;ZFYVE26;ZNF507;ZNF608;ZNF644 |
| TGCACTG,MIR-148A,MIR-152,MIR-148B | http://www.broadinstitute.org/gsea/msigdb/cards/TGCACTG,MIR-148A,MIR-152,MIR-148B | -0.61643 | -2.0755 | 0 | 0 | 279 | 141 | 9465;81609;4293;7039;84196;4335;9098;254170;22903;3340;7862;6310;605;2354;54206;23213;80155;55284;860;166336;9334;259230;22801;55082;3038;84280;6096;57419;4094;66008;56963;57561;64328;10776;51704;56261;23204;392255;2332;11176;10891;134957;23503;51167;9778;90;56243;5500;4286;219527;4810;9373;170692;9935;29969;60468;51132;23469;57572;91833;9828;1647;154214;9749;1435;8829;538;2823;5166;64795;27020;23032;494470;10928;9252;253260;55832;90627;84669;4520;8503;8028;2060;5562;3949;51014;10628;2099;152006;23431;54877;51306;54545;19;4052;8301;7764;152330;1656;9265;10251;147339;2200;27236;51422;8065;9044;140735;1948;231 | ABCA1;ACVR1;ADAMTS18;AKAP7;AP4E1;ARFIP1;ARGLU1;ARHGAP21;ARHGEF12;ARHGEF17;ARL6IP1;ARPP19;ARRDC3;ATP2A2;ATP7A;ATXN1;B4GALT5;BACH2;BAZ2A;BCL7A;BRPF1;BTAF1;BTBD10;BTBD3;C18orf25;CAND1;CHD9;CLOCK;CNTN4;CPEB4;CSF1;CUL5;CYB5R4;CYTH3;DDX6;DICER1;DMXL1;DOCK6;DYNLL2;EFNB2;EP300;EPS15;ERRFI1;ESR1;FAM13B;FBN1;FBXO11;FBXO33;FMR1;FOSB;GADD45A;GDF6;GPATCH8;GPCPD1;GPM6A;GPRC5B;HAS3;HECW2;INO80;ITGA11;ITSN2;KIAA0232;KIAA1217;LDLR;LMTK2;LRRC55;LTBP1;MAF;MAFB;MAP3K9;MDFIC;MIER1;MITF;MLLT10;MNT;MTF1;MTMR12;NAA15;NCOA1;NDST1;NHS;NPTN;NRP1;OSBPL11;OTUD4;PATL1;PDK4;PHACTR2;PHF3;PICALM;PIK3R3;PLAA;PPARGC1A;PPP1CB;PPP1R12A;PRICKLE2;PRKAA1;PRKAG2;PTPR |

| | | | | | | | | | |
|---|---|---|---|---|---|---|---|---|---|
| | | | | | | | | 31;54617;57520;80204;488;23405;9444;23414;5797;4659;1901;222194;8648;23534;27327;114885;50618;219988;57584;7003;22853;2033;1657;23112;197131;80315;57708;9575;8867;54726;80205;23365 | M;QKI;RALBP1;RGMA;RICTOR;RLIM;RMND5A;RNF165;RNF217;RNF38;RORB;RPS6KA5;RSBN1L;RUNX2;S1PR1;SGMS1;SLC24A3;SNX27;SPRY3;STARD13;STXBP5;SULF1;SYNJ1;TEAD1;TGFA;TMED7;TNPO3;TNRC6A;TNRC6B;TRAK2;TXNIP;UBE2W;UBR1;USP32;USP33;USP48;USP6;WDR20;XPO4;ZCCHC2;ZFPM2;ZFYVE26;ZNF217 |
| CAGTGTT,MIR-141,MIR-200A | http://www.broadinstitute.org/gsea/msigdb/cards/CAGTGTT,MIR-141,MIR-200A | -0.62226 | -2.09967 | 0 | 0 | 292 | 141 | 115704;219654;9729;81609;26118;65084;9194;253943;460;5567;526;5663;51062;6310;3725;7068;54206;8577;5887;64376;80232;146760;94015;9962;29953;1452;8473;3214;6477;23299;1655;7532;23621;10439;64844;23057;56995;2824;22893;23168;6660;114823;1969;3759;6387;2627;10138;134957;6416;153241;85458;5520;155435;4026;26468;4628;5915;51136;55334;10363;406;6812;1112;55691;6777;11127;3839;323;8829;9043;5069;1456;8828;56890;57609;23047;23114;4154;64795;10135;23637 | ANKFY1;APBB2;ARNTL;ASTN1;ATL1;ATP2A2;ATP6V1A;ATP6V1B2;ATXN1;ATXN7;BACE1;BAHD1;BHLHE40;BICD2;BRD3;CBL;CDC42BPB;CDK13;CDK17;CEP120;CHD9;CLASP2;CSNK1A1;CSNK1G3;CXCL12;DCUN1D3;DDX5;DIP2B;DIXDC1;DLC1;DMXL1;DNAJC13;ELF2;EPHA2;ERRFI1;EVI5L;EXOC5;FOXN3;FRMD4A;FRMD6;GATA6;GPM6B;HIPK1;HMG20A;HOXB4;IKZF5;INO80D;ITSN1;JUN;KALRN;KCNJ2;KIAA0408;KIF3A;KPNA3;LENG8;LHX6;LPP;LRRC8A;MAP2K4;MARCH7;MBD5;MBNL1;MDM1;MED13L;MIB1;MYH10;NAMPT;NFASC;NMNAT2;NR2C2;NRP1;NRP2;OGT;OLFM |

| | | | | | | | | | |
|---|---|---|---|---|---|---|---|---|---|
| | | | | | | | | ;7337;2054;10179;523;91746;23317;6671;8997;55777;84919;23122;57826;10395;8019;54891;84146;6505;56262;8621;122786;51663;989;123879;23411;57534;1998;57616;9839;5793;6453;54521;488;9444;867;64759;23037;8239;8553;23035;51479;9578;7150;80351;10640;5128;114885;23060;6314;6651;23389;6938;204851;1657;23112;6801;23001;5049;7182;146057;80205 | 1;OSBPL11;PAFAH1B2;PAPPA;PDS5B;PDZD2;PHLPP2;PPP1R15B;PPP2R2A;PRKACB;PSEN1;PTPRG;QKI;RABGAP1;RAD23B;RAP2C;RARB;RBM33;RBM7;RMND5A;RNFT1;RTF1;RTN4RL1;SEPT7;SIAH1;SIRT1;SLC16A7;SLC1A1;SLC23A2;SLC39A9;SNX27;SON;SOX5;SP4;SPAG9;STAT5B;STRN;STX2;STXBP1;STXBP5;TCF12;THRB;TMEFF1;TMEM135;TNKS2;TNRC6B;TNS3;TOP1;TRHDE;TSHZ3;TTBK2;TTYH2;TULP4;UBE3A;USP9X;WDFY3;WDR26;WDR44;WSB1;YAF2;YTHDC1;YTHDF3;YWHAG;ZCCHC24;ZEB2;ZFR;ZNF609;ZNF644 |
| GTGCAAT,MIR-25,MIR-32,MIR-92,MIR-363,MIR-367 | http://www.broadinstitute.org/gsea/msigdb/cards/GTGCAAT,MIR-25,MIR | -0.66239 | -2.20026 | 0 | 0 | 294 | 141 | 64112;29116;4774;6548;55108;3344;55284;153090;64399;60313;3799;23152;10018;6047;8728;196441;1278;4170;863;9792;5989;2624;8473;3038;1385;56995;23360;6990;57419;8320;66008;57561;57459;50862;8864;3685;9464;2332;4781;10140;2627;11176;7360;54407;8013;6416;26060;4189;2274;5048;441531;9228;4091;4287;6943;51552;107 | ADAM10;ADAM19;ADAMTSL3;ADCY3;APPL1;ARHGEF17;ARID1B;ARRDC3;ARRDC4;ATP2A2;ATRX;ATXN3;BAZ2A;BAZ2B;BCL2L11;BMPR2;BRMS1L;BSDC1;CBFA2T3;CIC;CNNM4;CNTN4;COL12A1;COL1A2;CPEB2;CPEB3;CPEB4;CREB1;CREB3L2;CXXC5;DAB2IP;DDX3X;DLGAP2;DMXL1;DNAJB9;DOCK9;DYNLT3;EDEM1;EGR2;EOMES;EXOC5;FAM126B;FAM135A;FAM13B;FAM160B1;FBN1;FBXW7; |

| | | | | | | | | | |
|---|---|---|---|---|---|---|---|---|---|
| | -32,MIR-92,MIR-363,MIR-367 | | | | | | | 88;1959;5306;25998;9828;55294;25921;10129;81537;26504;96459;57579;51523;23047;4784;64764;109;27020;132660;59338;57646;91947;143686;9655;57188;8997;8503;57700;152006;4430;84312;25977;51306;9695;1303;4092;10725;152330;10766;7798;84656;2200;9908;9839;54665;9895;285172;488;22849;23037;4659;1901;4627;8850;23035;3708;1654;3096;8924;1130;132864;10640;399687;29994;5581;7204;115548;546;57492;4124;23515;51426;26524;7003;2803;1657;102;80315;23077;200576;659;8867;54726;5980;23348 | FCHO2;FHL2;FMR1;FNBP4;FNIP1;FOXN2;FRY;G3BP2;GATA2;GATA6;GATAD2B;GLYR1;GOLGA4;GPBP1L1;HAND2;HAS3;HERC2;HHIP;HIVEP1;IBTK;IQGAP2;ITGAV;ITPR1;KALRN;KAT2B;KIF5B;LATS2;LIN54;LUZP1;LYST;MAN2A1;MAP2K4;MCL1;MOAP1;MORC3;MYCBP2;MYH9;MYLIP;MYO18A;MYO1B;NECAP1;NFAT5;NFIA;NFIB;NFIX;NPTN;NR4A3;OGT;OTUD4;PAFAH1B1;PDS5B;PDZD2;PER2;PGAM4;PHLPP2;PIK3R3;PIKFYVE;PITPNA;PLEKHA1;POLK;PPP1R12A;PRKCE;RAB14;REV3L;RFX1;RNF141;RNF38;RNF4;RSBN1;S1PR1;SERTAD2;SESN3;SGPP1;SLC24A3;SLC38A2;SLC9A1;SMAD6;SMAD7;SOCS5;SYNJ1;TCF21;TEAD1;TECPR2;TOB1;TOB2;TRAK2;TRIO;TULP4;UBE2W;UGP2;USP28;ZDHHC5;ZEB2;ZFC3H1 |
| TGAATGT,MIR-181A,MIR-181B,MIR-181C,MIR-181D | http://www.broadinstitute.org/gsea/msigdb/c | -0.63597 | -2.22117 | 0 | 0 | 449 | 192 | 4323;51312;863;221687;8473;10479;26225;54910;1012;56135;7532;1385;5156;9882;56995;59343;5066;66008;56963;23228;57561;5074;23168;8864;51696;6660;8731;10040;8434;5357;640 | ABI3BP;ABTB2;ACAP2;ACSL1;ADAMTS1;ADAMTS18;ADARB1;ADCY9;AFF1;AFF4;AKAP6;AKT3;ANKRD13C;ANKRD50;ANO1;AP1G1;ARFGEF2;ARHGEF7;ARL5A;ARRDC3;ARSJ;ATP11C;ATP2A2;B4GALT1;BACH2;BAZ |

| | | | | | | | | | |
|---|---|---|---|---|---|---|---|---|---|
| | ards/TGAATGT,MIR-181A, MIR-181B, MIR-181C, MIR-181D | | | | | | | 62;2627;11176;84067;54407;9877;8013;2683;4026;55553;8545;5500;7078;4628;9698;23189;1316;170692;81848;144348;2353;2744;26130;84206;79642;81573;60468;113251;23469;79577;1960;91833;51088;783;50488;85440;51232;868;25841;64398;23125;1456;114299;257397;5166;5090;54816;8476;3836;57609;1783;5534;56134;25890;27020;23032;3617;2615;84132;9472;4131;2180;23321;157922;8874;115;22841;4215;55300;205327;104;23317;2800;57659;2186;9774;55619;1808;8503;55107;55917;2060;51320;91694;22884;2099;54014;256691;10150;23592;5594;51306;2140;54545;11275;4092;8301;10725;23411;6249;23774;6733;5780;152330;25836;286410;677;9510;6018;58487;9908;8065;27125;6049;54665;2908;9690;376940;6400;285172;2043;26122;54617;80204;488;23262;9444;64895;164;8850;8553; | 2A;BAZ2B;BCLAF1;BHLHE40;BIRC6;BMPR2;BPTF;BRD1;BRWD1;C2orf69;CACNB2;CAMSAP1;CAMTA2;CBFA2T3;CBLB;CDC42BPA;CDC73;CDH13;CGGBP1;CHD9;CLASP1;CLIP1;CNTN4;CPEB4;CREB1;CREBZF;CRIM1;CSNK1G3;CTTNBP2NL;CUL5;DCUN1D1;DDX3X;DIP2B;DNAJC13;DOCK10;DOCK7;DPYSL2;DYNC1LI2;EGR3;EPC2;EPHA4;EPS15;ESR1;ETS1;EYA3;FAM126B;FAM13B;FAM160A2;FBXO11;FNDC3A;FOS;G3BP2;GAPVD1;GATA6;GLS;GOLGA1;HECA;IMPG1;INO80;KANK1;KAT2B;KLF6;KLHL2;KLHL5;KPNA1;LARP4;LEMD3;LMBRD2;LONRF1;LPP;LRBA;LRRC32;MAMDC2;MAP1B;MAP2K1;MAP3K3;MAPK1;MBNL2;MEX3B;MEX3C;MINK1;MMP14;MPP5;MTMR12;MTMR9;MYH10;NCOA2;NFAT5;NIPBL;NPTN;NR2C2;NR3C1;NR4A3;OGT;OSBPL8;OTUD4;PALM2;PAM;PAPOLG;PAWR;PBX3;PCDHAC1;PCDHAC2;PDGFRA;PDK4;PDLIM5;PER2;PHF3;PI4K2B;PICALM;PIK3R3;PLCL2;PLS1;PPIP5K2;PPP1CB;PPP1R12B;PPP3R1;PRKCE;PTPN9 |

| | | | | | | | | | |
|---|---|---|---|---|---|---|---|---|---|
| | | | | | | | | 5604;10000;23527;1654;66036;57182;10274;114882;987;92255;29994;5581;4299;9819;10499;10564;22862;23332;10611;2113;168850;23112;4660;80315;54165;7182;23345;659;57448;54726;80205 | ;PUM1;QKI;RAB11FIP2;RBM26;RECK;RGMA;RLF;RNF182;RNF6;RNMT;RSBN1;RTF1;SEL1L;SEMA4C;SENP2;SIRT1;SLC25A37;SLC38A2;SLC9A6;SMAD7;SOX5;SOX6;SPRY4;SRPK2;STAG1;SYNE1;TAB3;TBC1D4;TIMP3;TNRC6B;TOM1L1;TRAK2;TRIM2;TSC22D2;TULP4;UBE3C;USP33;USP42;WDR20;WDR37;YWHAG;ZBTB4;ZC3H11A;ZC3H6;ZFP36L1;ZNF280D;ZNF664;ZNF800 |
| GCACTTT,MIR-17-5P,MIR-20A,MIR-106A,MIR-106B,MIR-20B,MIR-519D | http://www.broadinstitute.org/gsea/msigdb/cards/GCACTTT,MIR-17-5P,MIR-20A,MIR-106A, | -0.63479 | -2.24282 | 0 | 0 | 556 | 275 | 64778;26039;51322;9371;9644;23130;10914;22924;4293;27430;4952;9098;253943;56137;29843;1026;79589;9344;5596;64067;10890;2152;6310;8754;26278;25909;3696;29883;9842;3778;92999;65977;55284;11278;50717;54874;79365;5869;23152;10018;80821;85464;399665;84541;259230;4170;9792;26960;23299;56135;1655;8729;5156;3037;84280;94134;131566;55205;22893;56963;81839;57459;5530;51727;51696;9053;51334;57403;5357;5933;1785;5774;7786;140 | ABCA1;ABHD2;ADAM9;AFF4;AHCTF1;AKAP13;AKT3;ANKFY1;ANKRD13C;ANKRD28;ANKRD50;ANO6;APBB2;APP;ARHGAP12;ARHGEF10;ARHGEF11;ARID4A;ARID4B;ATG16L1;ATG2A;ATXN1;BAHD1;BCL2L11;BCL2L2;BHLHE41;BICD2;BMPR2;BNC2;BNIP2;BRMS1L;BTBD10;BTBD7;CALD1;CAMTA2;CCNG2;CCNT2;CDC37L1;CDKN1A;CELSR2;CEP120;CEP97;CHD9;CIC;CLOCK;CMPK1;CNOT7;COL4A3;CRIM1;CRK;CRY2;CTDSPL2;DCAF8;DCBLD2;DCUN1D3;DDHD1;DDX5;DIP2A;DLGAP2;DMTF1;DNAJB9;DNAJC16;DNAJC27;DNM2;DPYSL2; |

| | | | | | | | | | |
|---|---|---|---|---|---|---|---|---|---|
| | MIR-106B, MIR-20B,MIR-519D | | | | | | | 8;1952;8013;153241;4642;5310;4189;23503;23094;10509;9826;5465;5520;5048;9848;80143;9770;26959;9135;9527;30061;26468;55664;9228;4091;9497;7067;85456;81848;4929;1398;5813;81573;388403;55691;23341;6774;10773;1959;55054;65117;50488;4090;256435;152273;51232;3839;323;55074;23125;55654;9988;8828;5090;84295;8462;54796;222236;51341;22887;57580;22992;26234;1783;2549;5534;619279;51474;56134;5775;687;5925;901;5756;23637;114879;1285;8897;64854;1982;9252;7707;4215;83737;6653;26018;9467;8690;51496;7525;84669;7126;7458;57659;351;144108;339745;1808;55810;59277;123606;4217;57826;23179;7091;27314;65267;30845;51742;55727;122830;2321;3949;22884;10628;64375;663;84312;23014;57169;81565;6886;23291;122786;123879;19;4092;10725;7764;7798;7959 | DYNC1LI2;DYRK1A;EGR2;EHD3;EIF4G2;EIF4H;ELK3;EPAS1;EPHA4;F3;FAM102A;FAM126B;FAM13C;FBXL5;FBXO21;FBXW11;FCHO2;FEM1C;FGD5;FLT1;FNBP1L;FNDC3A;FNDC3B;FOXJ2;FOXJ3;FRMD4A;FRMD6;GAB1;GAN;GATAD2B;GBF1;GOSR1;HAS2;HBP1;HECA;HIF1A;IKZF4;INO80;ITCH;ITGB8;JRKL;KAT2B;KBTBD8;KCNMA1;KDM2A;KIF3B;KLF11;KLF12;KLF9;KLHL20;KPNA3;LDLR;LHX6;LIMA1;LRIG1;LUZP1;MAP3K12;MAP3K2;MAP3K3;MAP3K5;MAP3K9;MAP7;MAPK4;MAPRE3;MARCH8;MAT2B;MCL1;MFAP3L;MINK1;MTMR3;MYO1D;NAA30;NAPEPLD;NBEA;NDEL1;NEK9;NFAT5;NIPA1;NPAS3;NPAT;NR2C2;NR4A2;NR4A3;NRP2;NTN4;OCRL;OSBPL5;OTUD4;OXR1;PAFAH1B1;PAFAH1B2;PAPOLA;PBX3;PCDHA12;PCDHAC1;PCDHAC2;PDGFRA;PHF6;PHLPP2;PIK3R1;PKD1;PKD2;PLEKHA3;PLEKHM1;PLS1;PPARA;PPP2R2A;PPP3CA;PPP3R1;PREX1;PRR16;PRRG1;PTPN3;PTPN4;PURA;QKI;RAB10;RAB22A;RAB30;RAB5B;RABEP1;RAB |

| | | | | | | | | | |
|---|---|---|---|---|---|---|---|---|---|
| | | | | | | | | 8;85461;9306;23181;599;9372;57616;3091;27125;56929;6049;54665;7048;9690;9639;91754;8139;285172;2043;23243;11069;54617;11057;9444;10618;23414;1901;8850;10210;4863;6497;5311;23035;10000;5926;51479;219749;1859;57182;80351;5638;27327;51277;196527;9321;115548;7776;54464;80829;54885;800;220965;22862;11214;5295;168850;23112;23001;27252;220972;5934;2034;5049;905;7182;23328;9575;659;54726;80205;10746;2004 | GAP1;RAP2C;RAPGEF4;RASSF2;RB1;RBL1;RBL2;RGL1;RGMA;RNF128;RNF6;RPS6KA5;RSBN1;RSRC2;S1PR1;SACS;SASH1;SEMA4B;SENP1;SERTAD2;SGMS1;SH3BP5;SH3PXD2A;SIKE1;SIPA1L3;SKI;SLC40A1;SLC4A7;SMAD5;SMAD6;SMAD7;SOCS6;SORL1;SPOPL;SPRY4;SPTY2D1;SS18L1;SSH2;ST6GALNAC3;STAT3;TAL1;TANC1;TAOK2;TBC1D8B;TGFBR2;TGOLN2;THRA;TLE4;TMEM127;TNFAIP1;TNKS1BP1;TNKS2;TNRC6A;TNRC6B;TOPORS;TRIP11;TSHZ3;TWF1;TXNIP;UBE2W;UBE3C;USP32;USP46;USP6;VANGL1;WAC;WDFY3;WDR37;WNK3;XRN1;YES1;YPEL2;YTHDF3;ZBTB4;ZBTB47;ZBTB6;ZBTB7A;ZFP91;ZFPM2;ZFYVE26;ZFYVE9;ZNF148;ZNF217;ZNF236;ZNF25;ZNF532;ZNF704;ZNF800;ZNFX1 |
| TGCACTT,MIR-519C,MIR-519B,MIR-519A | http://www.broadinstitute.org/gsea/msigdb/c | -0.65576 | -2.26628 | 0 | 0 | 413 | 199 | 9262;26278;25909;29883;4774;3781;65977;10420;5991;5893;11278;54874;79365;8674;5869;23152;85464;11078;4323;259230;196441;140685;9792;8473;3479;26960;23299;56135;1655;872 | ABHD2;ACVR1;ADIPOR2;AFF4;AHCTF1;AKAP13;AKT3;ANKFY1;ANKRD12;ANKRD50;ARHGAP12;ARHGAP24;ARHGAP29;ARHGEF11;ARID4A;ARID4B;ASAP2;ASXL2;ATG16L1;ATP2C1;ATRN;BAHD1;BCL2L2;BHLHE41;B |

| | | | | | | | | | |
|---|---|---|---|---|---|---|---|---|---|
| | ards/TGCACTT,MIR-519C, MIR-519B, MIR-519A | | | | | | | 9;5156;3037;84280;8455;6096;94134;131566;22893;23493;81839;57459;5530;51727;51696;6660;9053;10659;1785;9683;7786;1952;153241;4189;10509;9826;5048;10144;155435;90;26959;3920;9527;30061;55664;79776;9497;7067;85456;81848;8853;3213;23341;113251;6774;91833;22941;55054;4090;3839;51755;55654;8828;8462;27097;54796;83478;22887;57580;22992;79753;22807;26234;4784;619279;56134;5775;5597;4154;687;5925;79602;27032;1285;4131;1982;7337;9794;4215;83737;253260;6653;26018;9467;7525;2800;84669;57659;144108;339745;1808;123606;5930;4217;57826;65267;51742;51347;9958;55727;122830;5562;166968;54014;64375;84312;57169;81565;23291;122786;11234;4092;10725;7764;389136;57534;79598;57120;9306;23181;599;147339;9372;3091;27125;6670;7048;285172;2043 | ICD2;BIRC6;BMPR2;BNC2;BRMS1L;BRWD1;BTBD10;BTBD7;C18orf25;CALD1;CDC37L1;CDK12;CELF2;CELSR2;CEP120;CEP97;CHD9;CIC;CMPK1;CNOT7;COL4A3;CPEB2;DCBLD2;DDX3X;DDX5;DIP2A;DNAJB9;DNAJC16;DNM2;DPYSL2;DYRK1A;EIF4G2;ELK3;EPC2;EPHA4;FAM126B;FAM13A;FBXL5;FBXW11;FCHO2;FNBP1L;FOXJ3;FRMD6;GATAD2B;GBF1;GOLGA1;GOPC;GOSR1;HAS2;HBP1;HECA;HEY2;HIF1A;HIVEP2;HOXB3;HPS5;IGF1;IKZF2;IKZF4;ITCH;ITPR1;KCNN2;KDM2A;KLF11;KLF12;KLF9;KLHL20;KPNA3;LAMP2;LARP4;LRIG1;MAML1;MAP1B;MAP3K12;MAP3K2;MAP3K3;MAP3K5;MAP7;MAPK6;MBNL1;MED14;MIB1;MIER3;MMP14;N4BP1;NAA30;NBEA;NDEL1;NF1;NFAT5;NFIA;NFIX;NIPA1;NPAT;NRP2;OGT;PAFAH1B1;PCDHAC1;PCDHAC2;PDGFRA;PIK3R1;PKD2;PLEKHA3;PPP3CA;PREX1;PRKAA1;PTPN4;QKI;RAB5B;RAD52;RAP2C;RAPGEF4;RB1;RBBP6;RBM33;RFX3;RICTOR;ROCK2;RORB;SACS;SASH1;SEMA4B;SERTAD2;SG |

| | | | | | | | | | |
|---|---|---|---|---|---|---|---|---|---|
| | | | | | | | | ;11069;26122;11057;9444;4763;23414;10210;4863;6497;5311;3708;10000;23253;5926;51479;1654;1859;57182;132864;27327;9282;3097;115548;7776;80829;9411;9475;800;7003;11214;5295;23001;27252;23328;55252;659;57448;80205;10746;54861;2004 | MS1;SH3BP5;SHANK2;SKI;SLC40A1;SLC4A7;SMAD5;SMAD7;SNIP1;SNRK;SOCS6;SORL1;SOX5;SP3;SPOPL;SPRY4;SPTY2D1;SSH2;STAT3;STK17B;TAF5L;TAOK3;TEAD1;TESK2;TGFBR2;THRA;TMEM127;TNKS1BP1;TNRC6A;TOPORS;TRIOBP;UBE3A;USP15;USP32;VAMP4;VANGL1;VGLL3;WDFY3;WDR20;WNK3;YES1;ZBTB4;ZBTB46;ZFC3H1;ZFHX4;ZFP91;ZFPM2;ZFYVE9;ZNF217;ZNF236;ZNF704;ZNFX1 |
| TTGCACT,MIR-130A,MIR-301,MIR-130B | http://www.broadinstitute.org/gsea/msigdb/cards/TTGCACT,MIR-130A,MIR-301,MIR-130B | -0.66481 | -2.27774 | 0 | 0 | 365 | 175 | 7482;9334;84541;196441;8473;55082;26960;2697;5156;9706;8455;94134;131566;57419;4094;22893;57561;51727;51696;56261;6660;2332;1785;9683;7786;11176;55288;3815;153241;26060;9686;23503;5048;155435;90;26959;4810;57162;9373;170692;57620;9935;3213;29969;23683;60468;23341;57649;23469;91833;5209;22941;55054;4090;1647;9749;1435;8829;23219;8828;10742;22992;79753;137492;90693;4154;255967;5868;27020;2 | ABCA1;ACBD5;ACSL1;ACVR1;ADAMTS18;ANKRD12;ANKRD28;AP1G1;APPL1;ARFIP1;ARGLU1;ARHGAP12;ARHGAP21;ARHGEF12;ARID4B;ARRDC3;ASXL2;ATG16L1;ATP11A;ATRN;ATRX;B4GALT5;BACH2;BAHD1;BAZ2A;BIRC6;BMPR2;BPTF;BRWD1;BTAF1;BTBD7;CCDC126;CEP120;CEP170;CHD9;CLIP1;CLOCK;CMPK1;CPEB4;CSF1;CYLD;DCBLD2;DDX6;DICER1;DIP2A;DLL1;DNAJC16;DNM2;DPYSL2;DYNLL2;EFNB2;ELK3;EPC2;EPS15;ESR1;EXOC5;FBXO28;FMR1;G3BP2;GADD45A;GJA1;GPATCH8;GPCPD1; |

| | | | | | | | | | |
|---|---|---|---|---|---|---|---|---|---|
| | | | | | | | | 3032;2180;494470;23321;10928;9794;28514;26018;9655;90627;84669;57659;2186;144108;339745;4520;1808;11138;123606;8028;57826;27314;65267;2060;51742;91694;4306;55727;122830;5562;3949;166968;2099;54014;152006;64375;81565;54545;23174;152503;10198;19;7764;6249;389136;1656;57534;51621;9306;23181;9859;23250;9372;9908;27236;57605;7871;9839;9044;140735;5793;7048;1948;23243;23131;11069;26122;91452;4012;54617;57520;23405;9444;23414;5797;164;1901;22911;23013;4863;3708;8648;23253;10640;27327;3097;1540;23060;546;80829;57584;168850;23112;23001;27252;80315;57708;23328;55252;9575;659;57448;54726;80205;23365;2004 | HBP1;HECA;HECW2;HIVEP2;HOXB3;IKZF4;INO80;ITPR1;KBTBD8;KDM2A;KIT;KLF13;KLHL20;LDLR;LNPEP;LONRF1;LRIG1;MAF;MAFB;MAML1;MAP3K12;MBNL1;MDFIC;MIB1;MIER1;MIER3;MLLT10;MPHOSPH9;MTF1;MTMR12;N4BP1;NAA30;NBEA;NCOA1;NDEL1;NHS;NIPA1;NPAT;NPTN;NR3C2;NRP1;NRP2;OGT;OTUD4;PAFAH1B1;PAN3;PDGFRA;PELI1;PFKFB3;PHACTR2;PHF12;PHF3;PITPNM2;PLAA;PRKAA1;PRKD3;PTPRG;PTPRM;QKI;RAB30;RAB5A;RAI2;RALBP1;RAP2C;RAPGEF4;RBM33;RHOT1;RNF165;RNF38;S1PR1;SASH1;SH3D19;SHANK2;SLC24A3;SLMAP;SMAD5;SNIP1;SOCS5;SOCS6;SOX5;SPEN;SPOPL;SPTY2D1;STARD13;STIM2;TBC1D8;TGFBR2;TNRC6A;TNRC6B;TRIM2;ULK2;USP32;USP33;VGLL3;VGLL4;VPS37A;WDFY3;WDR20;WDR47;WNK3;WNT2B;ZBTB4;ZCCHC14;ZEB2;ZFC3H1;ZFP91;ZFPM2;ZFYVE26;ZFYVE9;ZNF217;ZNF609;ZNF800 |
| TTTGCAC,MIR-19A,MIR-19B | http://www.bro | -0.66427 | -2.3365 | 0 | 0 | 479 | 232 | 29883;23213;29116;6548;80232;10420;56907;159;860;60481;5 | ABCA1;ABR;ACBD5;ADAMTS18;ADCY9;ADIPOR2;ADNP;ADSS;AFF1;AN |

| | | | | | | | | | |
|---|---|---|---|---|---|---|---|---|---|
| | adinstitute.org/gsea/msigdb/cards/TTTGCAC,MIR-19A,MIR-19B | | | | | | | 1230;8654;89795;5869;166336;80124;388;23492;9334;84541;5989;8473;3479;10479;55082;26960;54910;8325;29;56135;9857;23621;10439;2697;27348;57018;94134;131566;57419;66008;57561;10776;56261;6660;2332;3759;63982;23208;79665;7786;3815;64225;26060;9686;23503;5465;25852;4026;55553;26959;285382;56243;4810;9373;170692;1605;158471;4929;8853;29969;831;6777;79705;23341;11127;57649;51592;65986;91833;5209;22941;55054;50488;4090;3839;51762;23219;8828;10742;1318;54796;9863;22992;79753;137492;90693;1389;56134;5597;4154;23011;27020;23032;79602;54602;9849;27032;494470;157922;115;26018;91947;26994;6446;9649;9655;84669;57659;2186;55125;8503;55107;11138;123606;57826;23179;7473;54453;2060;51742;91694;27115;2185;55727;166968;10395;2099;51719; | KRD12;ANO1;ANO3;AP1G1;APPL1;ARFIP1;ARGLU1;ARHGAP12;ARHGAP21;ARHGAP5;ARHGEF12;ARID4B;ARMC8;ARPP19;ARRDC3;ARRDC4;ASAP2;ASXL2;ATG16L1;ATL2;ATP11A;ATP2C1;ATRX;B4GALT5;BACE1;BMPR2;BNC2;BPTF;BRWD1;BTAF1;BTBD7;C3orf70;CAB39;CACNA1C;CAMSAP1;CAST;CBX7;CCDC126;CCNL1;CCNT2;CDK13;CEP170;CEP192;CEP350;CLIP1;CLOCK;CNOT7;CPEB4;CREBL2;CYLD;DAG1;DCBLD2;DDX3X;DDX6;DHX40;DICER1;DLC1;DNAJC16;EFNB2;ELK3;ELOVL5;EPC2;EPS15;ESR1;EXOC5;FAM114A1;FBXO28;FBXO8;FEM1C;FMR1;FNDC3A;FZD8;G3BP2;GJA1;GPCPD1;HBP1;HECW2;HIPK1;IGF1;IGF2R;INO80;ITPR1;ITSN1;KBTBD8;KCNJ2;KDM2A;KIAA1217;KIF3A;KIT;KLF10;KLF13;KLHL20;KPNA3;LNPEP;LONRF1;LPP;LRIG1;LRRK1;MACF1;MAGI2;MAP3K12;MAPK6;MBNL1;MBNL2;MDFIC;MED13L;MIB1;MIER1;MIER3;MINK1;MON2;MPHOSPH9;MYLIP;NAV3;NBEA;NDFIP2;NHS;NIPA1;NPTN;NR4A2;NRP2; |

| | | | | | | | | | |
|---|---|---|---|---|---|---|---|---|---|
| | | | | | | | | 54014;152006;23394;10154;26269;10150;8621;989;152503;10198;19;7764;6249;3482;1656;27303;57534;775;22931;51621;5412;29110;9859;23250;9372;57616;83452;8578;9908;27236;57605;7871;9839;56929;9044;5793;7048;6453;1948;9525;11069;26122;91452;4012;54521;54617;57520;23405;9444;10618;23414;164;1901;22911;23013;3708;23253;1654;6595;10640;27327;84932;1540;7071;5529;54778;23060;23041;4299;546;219988;81846;80829;57584;23389;22862;204851;168850;10771;23112;394;23001;27252;80315;57708;905;92689;55252;9575;659;23499;160760;23365;2004 | OGT;OLFM1;PATL1;PCDHAC1;PCDHAC2;PDE5A;PDE7B;PFKFB3;PHF12;PHF20;PIK3R3;PITPNM2;PLAA;PLXNC1;PPARA;PPP2R5E;PPTC7;PRICKLE2;PRUNE2;PTK2B;PTPRG;QKI;RAB18;RAB21;RAB2B;RAB33B;RAB5B;RAB8B;RAI2;RALGPS1;RAP2C;RAPGEF4;RBMS3;RFX1;RGL1;RHOB;RIN2;RNF11;RNF111;RNF165;RNF38;RUNX2;S1PR1;SBF2;SCARF1;SEMA4C;SEPT7;SGK1;SH3D19;SHANK2;SLC24A3;SLC31A2;SLC9A1;SLC9A6;SLMAP;SMAD5;SMARCA2;SNIP1;SOCS5;SOX5;SOX6;SPEN;SPIRE1;STAT5B;SULF1;SYT11;TBC1D8;TBK1;TESK2;TGFBR2;TGOLN2;TNRC6A;TNRC6B;TOR1B;TRAK2;TRIM33;TSHZ3;UBL3;USP32;USP33;VCPIP1;VGLL4;VPS37A;VPS4B;WDFY3;WDR20;WDR26;WDR44;WDR47;WNT3;ZBTB10;ZBTB4;ZEB2;ZFP91;ZFPM2;ZFYVE26;ZFYVE9;ZMYND11;ZNF217;ZNF518A;ZNF609;ZNF800 |
| CGGTGTG,MIR-220 | http://www.broadinstit | 0.464281 | 0.783337 | 0.748344 | 0.928274 | 5 | 1 | 2876 | GPX1 |

| | | | | | | | | | |
|---|---|---|---|---|---|---|---|---|---|
| | ute.org/gsea/msigdb/cards/CGGTGTG,MIR-220 | | | | | | | | |
| CCTGAGT,MIR-510 | http://www.broadinstitute.org/gsea/msigdb/cards/CCTGAGT,MIR-510 | 0.31378 | 0.832976 | 0.74833 | 1 | 39 | 8 | 10169;126382;55215;51447;5935;51147;5564;55624 | FANCI;ING4;IP6K2;NR2C2AP;POMGNT1;PRKAB1;RBM3;SERF2 |
