# Supplementary material for: SNRPC promotes hepatocellular carcinoma cell motility by inducing epithelial‐mesenchymal transition
Source: FEBS Open Bio. 2021 May 12;11(6):1757–70. doi: 10.1002/2211-5463.13175 (PMC8167856; doi:10.1002/2211-5463.13175)
Supplement: Supplementary file 6 — Table S6. Transcription factor enrichment of SPRNC coexpressed genes. [file FEB4-11-1757-s001.pdf]

Supplementary Table 6. Transcription factor enrichment of SPRNC co-expressed genes.

geneSet	link	ES	NES	P Value	FDR	size	Leading Edge Num	Leading Edge Id	User ID
GGAANCGGAANY_UNKNOWN	http://www.broadinstitute.org/gsea/msigdb/cards/GGAANCGGAANY_UNKNOWN	0.702829	2.151884	0	0	97	38	1460;55168;6169;6635;23480;7311;10856;5692;51035;51649;8815;54460;6881;6158;59286;27335;1340;84545;64320;6388;23583;27243;170622;8667;11333;112950;51371;219927;64601;5917;10969;1678;1347;84285;2193;529;5190;56658	ATP6V1E1;BANF1;CHMP2A;COMMD6;COX6B1;COX7A2;CSNK2B;EBNA1BP2;EIF1AD;EIF3H;EIF3K;FARSA;MED8;MRPL21;MRPL43;MRPS18A;MRPS21;MRPS23;PDAP1;PEX6;POMP;PSMB4;RARS;RNF25;RPL28;RPL38;RUVBL2;SDF2;SEC61G;SMUG1;SNRPE;TAF10;TIMM8A;TRIM39;UBA52;UBL5;UBXN1;VPS16
YNGTTNNNATT_UNKNOWN	http://www.broadinstitute.org/gsea/msigdb/cards/YNGTTNNNATT_UNKNOWN	-0.56794	-1.92991	0	0	342	109	9957;639;9924;3214;5087;23704;6096;114883;8320;57132;93986;57118;627;23509;6660;23204;2332;3759;4781;10370;10140;28981;267;55297;9686;443;64101;90;4286;26468;5136;5137;27332;64135;160335;29951;1112;4958;64750;23196;868;9197;79789;84295;4734;22807;2775;4784;6304;687;4919;63971;83468;7707;596;90627;6671;55534;8470;1756;84614;10395;93649;10735;7403;25977;54843;9732;6886;84253;25925;6526;4853;25836;65059;79875;57616;367;	ACVR1;AMFR;AR;ARL6IP1;ASPA;ATP2A2;BCL2;BDNF;CALD1;CAMK1D;CBLB;CCDC91;CHD2;CHD6;CHMP1B;CITED2;CLMN;CPEB4;CTNND1;DAAM1;DCUN1D1;DIS3L;DLC1;DMD;DOCK4;EMP1;EOMES;FAM120A;FMR1;FOXN3;FOXP2;GARNL3;GLT8D2;GNAO1;HIPK1;HOXB4;HS3ST1;IFIH1;IFT81;IKZF2;ITGA1;ITPR1;JMJD1C;KCNE4;KCNJ2;KDM6A;KIF13A;KLF9;LHX6;LRRC4;MAGI1;MAML3;MIA2;MITF;MYOCD;NECAP1;NEDD4;NFIB;NFIX;NIPBL;NOTCH2;NUMB;OMD;OSBPL9;PAN2;PBX1;PDE1A;PDE1C;PDZRN4;PHF6;PIK3R1;POFUT1;PPP2R5E;PRDM1;R3HDM2;RAPH1;ROR1;RORB;RSF1;R

								23118;115752;488;23038;23414;1901;23002;22864;9871;3708;3672;8650;1106;6595;84181;2012;5529;9819;51773;1500;800;4253;6938;5295;204851;221037;80315;54165;121274;862;9223	UNX1T1;S1PR1;SATB1;SEC24D;SLC33A1;SLC5A3;SMARCA2;SMURF2;SORBS2;SOX5;SP4;STAG2;STARD13;SYTL2;TAB2;TAL1;TCF12;THSD4;TMTC2;TOB1;TSC22D2;TSHZ3;VGLL4;WDTC1;ZBTB37;ZFPM2;ZNF148;ZNF521;ZNF638;ZNF641
V$HFH3_01	http://www.broadinstitute.org/gsea/msigdb/cards/V$HFH3_01	-0.56584	-1.79514	0	4.35E-04	173	66	4774;54806;653;1305;84928;9522;11278;860;89795;26166;2674;64116;639;9966;23057;1740;11276;4094;56963;93986;84189;222255;10140;3351;8013;4208;83990;5500;6586;3213;4958;10788;1959;1843;51232;51199;22887;4734;2313;2775;6446;51496;2800;9185;1756;84614;4306;4163;23291;389136;25836;84068;7082;10602;27253;8609;54521;80204;488;6595;84181;7328;197131;80315;5286;5978	AHI1;ATP2A2;ATXN7L1;BMP5;BRIP1;CDC42EP3;CHD6;COL13A1;CPEB4;CRIM1;CTDSPL2;DLG2;DMD;DUSP1;EGR2;FBXO11;FBXW11;FLI1;FOXJ3;FOXP2;GFRA1;GNAO1;GOLGA1;HOXB3;HTR1B;IQGAP2;KLF12;KLF7;MAF;MCC;MEF2C;NAV3;NEDD4;NFIA;NIN;NIPBL;NMNAT2;NR3C2;NR4A3;OMD;PCDH17;PIK3C2A;PPP1CB;PRDM1;REPS2;REST;RGMA;RGS22;RUNX2;SCAMP1;SGK1;SLC10A7;SLC39A8;SLIT3;SLITRK6;SMARCA2;SYNRG;TJP1;TMEM209;TNFSF15;TOB1;UBE2H;UBR1;VGLL3;WDR44;ZBTB37
TTAYRTAA_V$E4BP4_01	http://www.broadinstitute.org/gsea/msigdb/cards/TTAYRTAA_V$E4BP4_0	-0.54286	-1.79582	0	4.54E-04	237	74	143279;55784;23221;1655;7532;1831;405;2035;57561;8864;55118;10140;8814;7402;57222;161742;26468;54414;27332;10218;81848;79772;5813;114905;57161;2570;55294;25921;51232;	ADNP;AHNAK;AK3;ANGPTL7;ANO1;ARID1B;ARNT;ARRDC3;C1QTNF7;CDK8;CDKL1;CNTF;COL15A1;CRIM1;CRTAC1;CYLD;DDX5;DENND4A;DMD;DPYSL2;ENPEP;EP300;EPB41;ERGIC1;FBXO3;FBXW7;FRY;GABRR2;GPM6A;HECTD2;HI

	1							10129;64398;8741;144165;2823;222236;51341;80333;22807;253827;687;1270;90627;1808;8503;55107;23179;1756;79026;1306;50808;3631;2028;23394;10150;25836;9372;59269;80243;10260;22911;8633;8500;26273;1024;1540;57492;4638;2033;7328;221037;10019;5592;3572;9728	VEP3;IKZF2;IL6ST;INPP4A;JMJD1C;KCNIP4;KLF9;LHX6;MBNL2;MCTP1;MCTP2;MPP5;MSRB3;MYLK;NAPEPLD;NIPBL;PELI2;PER2;PIK3R3;PPFIA1;PREX2;PRICKLE1;PRKG1;PURA;RGL1;RHOBTB2;SECISBP2L;SH2B3;SIAE;SPRED1;SPRY4;STARD13;TNFSF13;TOB1;TSC22D3;UBE2H;UNC5C;UTRN;WDR47;YWHAG;ZBTB7A;ZDHHC5;ZFYVE9;ZNF638
SMTTTTGT_UNKNOWN	http://www.broadinstitute.org/gsea/msigdb/cards/SMTTTTGT_UNKNOWN	-0.52394	-1.80645	0	4.71E-04	369	129	2844;1195;9874;51380;55749;1879;4323;1452;79646;9949;1848;54413;2628;2624;3479;4478;54910;1600;1655;7532;23057;26298;57396;1740;7483;55809;8554;9969;93986;1123;25942;7074;4007;6660;4915;55512;3782;10370;5155;54407;8013;64101;57522;56062;23429;26468;80267;27332;91607;2932;9205;4929;3213;5813;1112;60468;57649;23095;57161;1399;9828;660;2823;51199;57580;22992;10664;22807;51631;57509;2775;4784;79895;2114;5597;4154;4919;4131;1982;10818;23314;8447;351;4651;7174;285513;1756;55917;	AMMECR1;APP;ARHGEF17;ARID4A;ATP8B4;ATRX;BACH2;BMPR2;BMX;CALD1;CCAR1;CHD2;CHN1;CITED2;CLASP1;CLK1;CLK4;CPEB4;CRKL;CSAD;CSNK1A1;CTCF;CTTNBP2NL;DAAM1;DAB1;DDX3X;DDX5;DDX6;DLG2;DMD;DOC2B;DPF3;DUSP6;EBF1;EDEM3;EHF;EIF4G2;EPC1;ERG;ETS2;FOXN3;FOXP2;FRS2;GATA2;GATM;GLYR1;GNAO1;GNAQ;GPM6A;GPR173;GPR21;GPRIN3;GSK3B;HOXB3;IGF1;IKZF2;ITPR1;KCNN3;KDM2A;KIF1B;KLF7;KLHL4;LHX6;LRRC4;LTBP1;LUC7L2;MAP1B;MAPK6;MBNL1;MED13;MMP14;MSN;MTUS1;MYO10;NF1;NFIX;NIN;NIPBL;NLGN3;NMNAT2;NR4A2;NR4A3;NTRK2;ONECUT1;PANK3;PCF11;PDGFB;PELI2;PHF12;PIAS1;PREX1;PRI

								8110;25862;3175;7750;54328;23174;4052;6733;27107;1656;25836;23186;84656;677;6925;23118;6670;80818;8609;9444;4763;10927;51585;2776;8633;2078;23002;3708;5926;1654;1106;4086;546;800;5629;23332;80314;80315;862;659;167465	CKLE3;PROX1;PURA;QKI;RCOR1;ROR1;RUNX1T1;RYBP;SATB2;SEMA4C;SIN3A;SLC38A2;SLFN11;SMAD1;SMPD3;SOX5;SP3;SPIN1;SRGAP1;SRPK2;TAB2;TCF4;TIAM1;TLK1;TPP2;TRERF1;UNC5C;USP49;WNT9A;YWHAG;ZBTB11;ZCCHC14;ZFP36L1;ZMYM2;ZMYM5;ZNF366;ZNF436;ZNF638
YTATTTTNR_V$MEF2_02	http://www.broadinstitute.org/gsea/msigdb/cards/YTATTTTNR_V$MEF2_02	-0.50399	-1.79594	0	4.75E-04	637	254	57631;90952;1513;6547;222663;7077;54875;64388;11221;151126;6781;8848;595;1160;81629;23433;8715;4684;3164;5577;4139;4015;2857;3726;3398;84002;4168;57158;79628;51778;2000;3636;70;51322;146862;9162;284723;64770;51266;4974;10943;9099;26191;2257;1026;28513;9069;201232;6310;9734;53354;3725;2918;2354;51105;8987;8509;122011;4774;3290;3174;22848;146760;5991;11278;10586;7043;8573;9874;10018;140885;267004;1879;4323;9957;1278;9949;142685;159091;639;6563;26960;388591;7532;1385;5156;115294;3037;2822;389677;1740	AAK1;ACTC1;ADAMTS12;AFF4;AGTPBP1;AKAP10;AMMECR1;ANGPTL7;ANKRD28;ANKRD44;ANO1;ARAP2;ARHGEF15;ARNTL;ARRDC3;ASB15;ASB7;ATP2C1;ATP6V1A;ATXN1;B3GNT5;BCL2L11;BDNF;BHLHE40;BMPR2;C18orf25;CASK;CASQ2;CAST;CAT;CCDC14;CCND1;CDC42EP3;CDH19;CDKL5;CDKN1A;CHD2;CHN1;CKMT2;CLASP1;CLDN12;CLEC1B;CLIC5;CNST;CNTF;CNTLN;COG6;COL1A2;COL8A1;CREB1;CSNK1A1L;CTNND1;CTR9;CTSK;DACT1;DBH;DENND2C;DGKI;DLG2;DMD;DNAJC3;DOCK8;DUSP10;EBF1;ELF4;EPHA3;ERG;ESAM;ETS1;FAM107B;FAM122C;FBXO11;FGF12;FOS;FOSB;FOXJ3;FOXP2;FRMD4A;GABRR2;GNAO1;GNB4;GPLD1;GPR34;GREM2;GRM8;GTF3C3;HAS2;HBP1;HDAC9;HIPK

								;140460;7763;66008;4978;9969;93986;1123;57561;81839;627;9330;23509;22899;3895;3782;11343;56114;51339;7273;4781;81704;10891;23287;80055;5611;64101;5465;5520;26959;59345;5325;9358;23387;4286;55167;4628;163882;3977;10218;3738;8082;29951;6444;9646;2353;84230;406;847;53405;81847;831;55691;79041;23469;51592;4958;2570;845;25921;6710;9830;8741;8515;55654;8828;5090;5770;11043;22887;4734;3908;7092;2775;4784;51562;23011;23235;55591;26051;27032;284273;63971;4041;1270;9794;116984;523;57646;5965;6443;3752;51086;9462;6671;55534;55107;7174;2042;57826;23179;1756;7091;5494;84614;4306;93649;54891;10735;10150;57169;81565;54545;64805;51274;10725;3570;91526;8204;147339;11099;83641;1621;163259;1295;7871;10602;27125;79618;6670;11183;6453;54	1;HIPK3;HMBOX1;HNF4G;HS3ST1;HSD11B1;ID2;IL6R;INO80D;INPPL1;ITGA10;ITGBL1;ITSN1;JMJD1C;JPH2;JUN;JUNB;KCNA3;KCND3;KCNN3;KIAA0355;KIF13A;KLF12;KLF3;KTN1;LAMA2;LIFR;LOX;LRCH2;LRP5;LRRC4;LRRC8C;MAB21L2;MAML1;MAML3;MAP4K5;MARK1;MBIP;MBNL2;MCF2;MED13;MGLL;MIA2;MID2;MITF;MMP14;MSL2;MSL3;MTMR12;MYH10;MYO9A;MYOCD;MYOZ2;NBEA;NCAM1;NDEL1;NDST2;NEDD4;NFAT5;NFIA;NFIB;NFIX;NOL4;NR3C2;NR4A1;NRIP1;NRP2;OMD;OMG;OPCML;P2RY12;PANK1;PBX3;PCDHGA1;PCMTD1;PDGFRA;PDLIM5;PDZRN4;PGAP1;PGBD3;PHF20L1;PHF3;PLAGL1;POFUT1;PPARA;PPARGC1A;PPM1A;PPM1B;PPM1L;PPP1R16B;PPP2R2A;PPTC7;PRDM1;PRKAR2B;PTPN1;PTPN21;PTPN22;RAB21;RAP2C;RASAL2;RBM12B;RECQL;RFX3;RGL1;RHOQ;RNF146;RNF207;RTN4RL1;SBF2;SCUBE3;SGCB;SGCD;SH3TC2;SIK2;SIK3;SIPA1L1;SIRPA;SLC14A1;SLC16A13;SLC25A34;SLC30A4;SLC8A3;SLMAP;SMARCA2;SP3;SP4;SPTB;SSPN;STAG2;STBD1;STC1;SYNE1;TGFB3;TIMP2;TLE4;TL

								95;23243;80204;26037;57511;23414;81792;2078;8553;151742;29761;1106;6595;7150;9710;6792;81846;1500;7782;4253;204851;23332;10611;2113;221037;11216;4649;23345;23528;659;160760;10114	K1;TLL1;TMEM127;TMEM38A;TNFSF13;TNNI3K;TOP1;TPP2;TRAK2;TRIM14;TRIM33;TSC22D1;TSSK3;TTN;UNC45B;USP2;USP25;USP28;VANGL1;VEZT;WAC;YWHAG;ZADH2;ZBTB37;ZDHHC5;ZFAND5;ZFPM2;ZNF281;ZNF385B;ZNFX1
V$IPF1_Q4	http://www.broadinstitute.org/gsea/msigdb/cards/V$IPF1_Q4	-0.54915	-1.81091	0	4.97E-04	230	88	123041;4015;652276;5362;79819;3636;2530;56937;4952;25913;10777;64067;94121;57685;8573;267004;9719;342096;639;2202;84251;10439;4969;2668;9969;2309;627;10776;284;27319;153241;4629;4208;5520;29126;1806;957;4929;5900;23051;1634;5813;23086;1112;60468;1959;4756;64750;57161;51088;64398;648;8829;2846;2313;58533;4784;285636;4154;80031;28514;90627;55534;4520;23516;163486;1756;7091;8110;25977;151449;51274;152485;10602;64641;79811;7026;26122;55729;1121;1859;6595;6938;10611;80314;57708;1793;56099	ADAMTSL2;ANGPT1;ARPP19;ARPP21;ATF7IP;BACH2;BDNF;BHLHE22;BMI1;C5orf51;CACHD1;CASK;CD274;CDC42EP3;CEP120;CHM;DCN;DENND1B;DLL1;DMD;DOCK1;DPF3;DPYD;DYRK1A;EBF2;EFEMP1;EGR2;ENTPD5;EPC1;EPC2;EXPH5;FLI1;FOXN3;FOXO3;FUT8;GDF7;GDNF;GOLGA6A;INPPL1;KLF3;KLHL5;LOC652276;LOX;LPAR4;MAML3;MBNL1;MED13;MEF2C;MIER1;MPP5;MTF1;MYH11;NECAP1;NEO1;NFIX;NPAS3;NR2F2;NR4A2;NRP1;OCRL;OGN;OLFM1;PCDHGB7;PDLIM5;PELI2;PGBD3;PLXNA2;PMEPA1;POT1;PPP2R2A;PRDM1;PURA;RALGDS;SEMA6D;SGIP1;SLC24A4;SLC39A14;SLTM;SMARCA2;SMURF2;SNX6;STARD13;SYTL4;TCF12;TLE4;WDR78;ZHX3;ZNF827
V$SRY_	http://www.b	-0.54467	-1.79884	0	4.97E	228	98	255082;6310;9726;4779;122011	ABCA6;ANGPT1;APBA1;ATXN1;BNC2;C

02	roadinstitute.org/gsea/msigdb/cards/V$SRY_02				-04			;4774;3781;3290;51705;7043;23460;1879;4303;9957;1848;2624;84251;7532;1831;10439;1499;1740;8554;4978;93986;1123;8861;8493;7074;4908;284;6660;4781;81566;79665;2335;8013;55553;4286;9698;64135;144348;29951;8976;5813;260425;57161;5928;8829;54796;11043;22807;2775;4784;6304;284273;4131;4041;1982;9794;320;28514;84669;53349;1105;55534;8642;55917;84614;4306;5294;7750;10735;25977;10150;51274;3624;1656;25836;54149;9908;6925;80243;10602;9839;64641;376940;80818;8609;9444;51585;2776;9771;27327;399687;7007;4638;5295	21orf91;CASC2;CDC42EP3;CHD1;CHN1;CSNK1A1L;CSRNP2;CTNNB1;CTTNBP2NL;DCHS1;DDX6;DHX40;DLG2;DLL1;DUSP6;EBF1;EBF2;EIF4G2;EMCN;FN1;FOXO4;FOXP2;G3BP2;GATA2;GNAO1;GNAQ;HS3ST1;HSD11B1;IFIH1;IKZF2;INHBA;KCNN2;KLF3;KLF7;LDB1;LRP5;MAGI3;MAML1;MAML3;MAP1B;MBNL2;MID2;MITF;MYLK;MYO18A;NECAP1;NFE2L1;NFIA;NFIB;NFIX;NIPBL;NR3C2;NR4A3;NRP1;NTF3;OLFM1;OPCML;PCF11;PDZRN4;PELI2;PIAS1;PIK3CG;PIK3R1;PPM1D;PREX2;PUM1;PURA;QKI;RAPGEF5;RBBP4;SATB1;SGIP1;SOX5;SOX6;STAG2;TCF4;TECTA;TGFB3;TIAM1;TNRC6A;TSC22D3;USP32;WASL;YWHAG;ZADH2;ZBTB37;ZC3H6;ZEB2;ZFYVE1;ZMYM2;ZNF436;ZNF646;ZNF664
V$GATA1_05	http://www.broadinstitute.org/gsea/msigdb/cards/V$GATA1_05	-0.54053	-1.82477	0	4.97E-04	265	93	27334;51043;6310;5140;3090;3725;154796;23213;54893;11278;151887;89866;8573;9874;3574;6563;55363;56256;1831;3569;54897;7024;6096;4094;93986;627;25942;80168;6660;161882;3790;2627;5782;25852;56062;91	AMOT;ARHGEF10L;ARMC8;ATXN1;BDNF;BMP10;BRWD3;CASK;CAST;CASZ1;CCDC80;COL4A3;COL4A4;CPEB4;CTCF;CYTH3;DIS3L;EGR2;ERG;FERMT2;FILIP1;FLI1;FOXP2;GATA6;GSK3B;HEMGN;HIC1;IKZF2;IL6;IL7;JUN;KCNS3;KLF12;KLHL4;LHX6;LMTK2;MAF;MAP4K5;MEC

								35;5325;23429;26468;5915;55160;10979;27302;2932;29951;831;65986;1959;26137;4756;5069;2829;51341;10664;4734;22807;2313;2122;80177;1285;7337;10743;6422;59338;8503;83604;23369;1286;27145;29072;6733;9265;5412;84068;23118;254065;115752;5793;11183;2908;7026;55031;23414;23037;2078;222194;27327;22853;6239;80315;23224;862;8867	OM;MOGAT2;MTMR10;MYCT1;NEDD4;NEO1;NR2F2;NR3C1;P2RY10;PAPPA;PDE3B;PDZD2;PDZRN4;PIK3R3;PLAGL1;PLEKHA1;PTPN12;PTPRG;PUM2;RABEP1;RAI1;RARB;RORB;RREB1;RSBN1L;RUNX1T1;RYBP;SEC16B;SERTAD4;SETD2;SFRP1;SIN3A;SLC10A7;SLC14A1;SOX5;SRPK2;SULF1;SYNE2;SYNJ1;TAB2;TFCP2;TLK1;TMEM47;TNRC6A;TSC22D3;UBE3A;UBL3;USP47;XCR1;ZBTB10;ZBTB20;ZBTB7A;ZBTB7B;ZFPM1;ZFPM2
V$CDC5_01	http://www.broadinstitute.org/gsea/msigdb/cards/V$CDC5_01	-0.56188	-1.85576	0	4.97E-04	230	80	2257;5567;23678;22903;25913;3983;54206;51179;11231;11278;6299;7043;5098;2674;159091;2534;5087;7532;11117;6096;1740;93986;57561;81839;284;11343;10370;64062;10891;3707;56062;4330;29951;6943;406;5813;266812;55691;60468;6774;1959;26137;50488;6332;8829;1456;54556;4154;2737;9794;170384;143686;51086;6578;9462;54880;8503;55917;10735;23186;775;152485;59269;10602;11279;23414;331;6095;1106;6595;11622	ABLIM1;ANGPT1;ARNTL;ARRDC3;ATRX;BACH2;BCOR;BMPR2;BTBD3;CACNA1C;CALD1;CDC42EP3;CHD2;CITED2;CLASP1;CPEB4;CSNK1G3;CTNND1;CTTNBP2NL;DLG2;EGR2;EMILIN1;ERRFI1;FAM122A;FAM122C;FGF12;FOXP2;FRMD4A;FUT11;FYN;GFRA1;GLI3;HAO2;HIPK1;HIVEP3;ING3;ITPKB;KLF12;KLF8;KLHL4;MAML1;MBNL1;MGLL;MINK1;MN1;NAP1L5;NRP1;PBX1;PCDHGC3;PDZRN4;PIK3R1;PIK3R3;POT1;PPARGC1A;PRKACB;PURA;RASAL2;RBM26;RCOR1;RORA;RORB;RREB1;SALL1;SCN7A;SEC63;SESN3;SGK3;SLCO2A1;SMARCA2;ST

								4;546;1500;800;5295;204851;23332;6239;80315;659	AG2;STAT3;TCF21;TGFB3;TNNI3K;VANGL1;XIAP;YWHAG;ZBTB20;ZFPM2;ZNF827
V$S8_01	http://www.broadinstitute.org/gsea/msigdb/cards/V$S8_01	-0.55071	-1.80036	0	5.22E-04	230	83	1826;7479;36;3636;1266;55103;64084;22903;11036;64067;5140;2354;653;64376;10420;5991;9508;85464;348654;3912;9039;22801;5566;8473;3710;1734;1655;26298;3037;6096;11276;140460;8320;23493;6660;10659;222255;1949;153241;55959;64101;4208;85458;4286;143425;79776;55160;160335;29951;406;55119;5813;6774;26137;4756;50488;8829;2199;57509;4154;7707;26994;9655;8503;23516;8028;1756;7091;54843;84068;80243;9839;64641;9079;23414;7405;23090;55787;5599;1500;800;6239;862	ACADSB;ADAMTS3;ARHGEF10L;ARNTL;ASB7;ATXN7L1;BMP5;BTBD3;CALD1;CELF2;CEP120;CLSTN2;CNN3;CTNND1;DDX5;DIO2;DIXDC1;DMD;DSCAM;EBF2;EFNB3;EHF;EOMES;FBLN2;FOSB;GEN1;GTF2A1L;HAS2;HEY2;IKZF5;INPPL1;ITGA11;ITPR3;LAMB1;LDB2;LRRC4;MAPK8;MBNL1;MEF2C;MINK1;MITF;MLLT10;MTUS1;NEO1;NPAS3;NRP1;OGT;PDE3B;PDZRN4;PIK3R3;PREX2;PRKACA;PRPF38B;PURA;RALGPS2;RFX3;RNF11;RORB;RREB1;RUNX1T1;SLC10A7;SLC39A14;SOCS5;SOX5;SSH2;STAT3;SULF2;SYNRG;SYT9;SYTL2;TESK2;TLE4;TMTC2;TXLNG;UBA3;UVRAG;WNT8B;ZBTB20;ZEB2;ZFHX4;ZFPM2;ZNF148;ZNF423
V$NFAT_Q6	http://www.broadinstitute.org/gsea/msigdb/cards/V$NFAT_Q6	-0.54617	-1.81096	0	5.26E-04	227	70	221687;728642;3214;26960;22893;26249;93986;8774;8864;6660;1969;7148;5155;8013;90;29126;382;4286;2932;81575;3213;79041;1281;8832;122953;257397;51068;84295;54796;22807;7	ACVR1;ANKRD17;ANKRD28;APOLD1;ARF6;ARHGAP5;ARHGAP6;ARL5B;ATG2B;BAHD1;BNC2;CD274;CD84;CDK11A;COL25A1;COL3A1;COL8A1;CPEB4;CRY1;CTNND1;DDR2;ELK3;EMP1;EPHA2;ERG;FLT4;FOXP2;GHR;GNAO1;GSK3B;HOX

								092;2775;55016;1407;395;7126;4306;2324;57630;84146;84570;29964;27303;51621;1295;5870;80243;79056;55279;6670;7026;23243;221079;2690;22911;2078;5028;54800;3339;2012;3987;7010;1500;7003;4921;26057;55102;394;80315;2004	B3;HOXB4;HSPG2;IKZF2;JDP2;KLF13;KLHL24;KLHL3;LIMS1;MARCH1;MITF;NAPG;NBEA;NMD3;NR2F2;NR3C2;NR4A3;P2RY1;PDGFB;PER2;PHF6;PREX2;PRICKLE4;PRRG4;RAB6A;RBMS3;RNF182;SH3RF1;SOX5;SP3;TAB3;TEAD1;TEK;TLL1;TMEM38A;TNFAIP1;TNXB;WDR47;ZNF644;ZNF654
V$SOX9_B1	http://www.broadinstitute.org/gsea/msigdb/cards/V$SOX9_B1	-0.55351	-1.82559	0	5.42E-04	223	89	79668;7072;255082;9734;9726;4804;51294;56882;8577;28951;3781;8573;10018;1879;2869;1848;5087;2260;1831;10439;10100;1499;5187;6660;3842;4781;9683;79665;5782;2335;26468;9698;55334;144348;51132;113251;4756;57161;10516;5928;124637;23047;2775;4784;80177;5868;26051;7337;9794;55632;320;4215;2186;53349;1808;4155;23516;8470;1756;5583;10395;25977;54843;9732;6886;51274;8301;3624;89796;1656;163259;9908;23301;2078;23002;23012;29761;6711;7150;84181;1024;116224;3339;399687;9353;7010;7003;6239;23328	APBA1;BCL2L11;BPTF;CASC2;CASK;CDC42SE1;CDK8;CHD6;CTNNB1;CYB5D1;DAAM1;DDX6;DENND2C;DHX40;DLC1;DMD;DOCK4;DPYSL2;DUSP6;EBF1;EHBP1;ERG;FAM122A;FBLN5;FGFR1;FN1;G2E3;G3BP2;GNAO1;GRK5;HDAC9;HSPG2;INHBA;KCNN2;KLF3;LARP4;LHX6;MAML1;MAP3K3;MBP;MYCT1;MYO18A;N4BP1;NAV1;NECAP1;NEO1;NFIB;NFIX;NGFR;OLFM1;PARP8;PBX1;PCDH12;PDS5B;PELI2;PER1;PICALM;PPP1R16B;PRKCH;PTPN12;PUM1;RAB5A;RBBP4;RLIM;RREB1;SASH1;SLC39A14;SLC39A9;SLIT2;SORBS2;SOX5;SPTBN1;STK38L;SYTL2;TAL1;TEAD1;TEK;TIA1;TMEFF1;TNPO1;TOP1;TRIB2;TSC22D3;TSPAN2;UBE3A;USP25;ZFYVE1;ZNF646;ZNF664

V$AR_Q2	http://www.broadinstitute.org/gsea/msigdb/cards/V$AR_Q2	-0.57197	-1.75935	0	5.59E-04	114	37	80124;2660;9949;54910;1831;7483;93986;627;10891;1602;57222;10513;5915;2932;81575;113251;22807;2595;4131;143686;7473;1756;23394;3624;57616;10602;11183;6498;7189;22911;50640;3096;1540;23090;9321;6239;80315	ADNP;AMMECR1;APOLD1;APPBP2;BDNF;CDC42EP3;CPEB4;CYLD;DACH1;DMD;ERGIC1;FOXP2;GANC;GSK3B;HIVEP1;IKZF2;INHBA;LARP4;MAP1B;MAP4K5;MSTN;PNPLA8;PPARGC1A;RARB;RREB1;SEMA4C;SESN3;SKIL;TRAF6;TRIP11;TSC22D3;TSHZ3;VCPIP1;WDR47;WNT3;WNT9A;ZNF423
V$NKX22_01	http://www.broadinstitute.org/gsea/msigdb/cards/V$NKX22_01	-0.56501	-1.81191	0	5.59E-04	172	62	1740;2035;93986;7074;9823;80705;9891;10418;2627;121536;7067;2932;29951;1398;6586;29969;79837;84897;3188;6777;56650;1959;26137;4756;55294;667;55628;54764;9754;8828;4734;64324;90693;4784;5597;63971;4851;2800;26058;285513;1756;10395;2099;10735;54843;56262;6733;25836;775;9265;9372;57605;55279;6400;27253;22864;3708;130507;23090;5295;221037;80315	AEBP2;ARMCX2;CACNA1C;CCDC126;CLDND1;CPEB4;CRK;CYTH3;DLC1;DLG2;DMD;DST;EGR2;EPB41;ESR1;FBXW7;FOXP2;GATA6;GIGYF2;GOLGA1;GPRIN3;GSK3B;HNRNPH2;ITPR1;JMJD1C;KIF13A;LRRC8A;MAPK6;MDFIC;NEDD4;NEO1;NFIX;NIPBL;NOTCH1;NRP2;NSD1;NUAK1;PCDH17;PDZRN4;PIK3R1;PIP4K2C;PITPNM2;R3HDM2;SEL1L;SLIT3;SPON1;SRPK2;STAG2;STARD8;STAT5B;SYTL2;TBRG1;THRA;TIAM1;TSGA10;UBR3;ZBTB20;ZFYVE9;ZNF407;ZNF423;ZNF654;ZRANB1
V$HLF_01	http://www.broadinstitute.org/gsea/msigdb/cards/V$HLF_01	-0.55733	-1.84546	0	5.59E-04	237	74	23221;56256;23039;1655;9966;7532;1831;2035;51201;2309;1123;57561;22824;57532;10659;23208;10140;55975;161742;81848;6586;5493;113251;283537;3	ADNP;ANGPTL1;ANO1;ARID1B;ARRDC3;BHLHE40;CALCRL;CDK8;CDKL5;CELF2;CHD2;CHN1;CLASP1;CLIC4;CNTF;COL15A1;CRIM1;DDX5;DENND4A;DMD;ECM2;EGFR;EPB41;FBXO3;FBXW7;FCH

								44148;57161;55294;25921;51232;1842;8741;144165;201799;222236;9068;687;80031;1270;55107;8394;1756;1306;3631;23394;9873;158219;10150;25932;9372;59269;80243;55279;10260;7026;10203;8239;80012;8553;6095;26273;1956;1106;7150;1024;118987;6792;57492;55787;56916;5295;23332;10019;5592;9728	SD2;FOXO3;HIVEP3;HSPA4L;INPP4A;KLF9;KLHL7;LARP4;MBNL2;NAPEPLD;NCKAP5;NR2F2;NUFIP2;PDZD8;PELI2;PHC3;PIK3R1;PIP5K1A;PPL;PREX2;PRICKLE1;PRKG1;RHOBTB2;RORA;SECISBP2L;SEMA6D;SERTAD4;SH2B3;SLC46A3;SLIT3;SMARCAD1;SPRED1;SPRY4;SYT11;TMEM154;TNFSF13;TNFSF15;TOB1;TOP1;TSC22D3;TTC39B;TXLNG;USP9X;XPO7;YWHAG;ZDHHC2;ZDHHC5;ZFYVE9;ZNF654
V$PITX2_Q2	http://www.broadinstitute.org/gsea/msigdb/cards/V$PITX2_Q2	-0.54773	-1.81951	0	5.74E-04	234	77	131578;57447;8573;166336;10497;4303;639;3214;5087;27197;6096;2668;22893;9969;93986;5142;4915;6101;675;443;90;8545;4286;23429;85456;2932;6943;5813;56980;65986;2355;667;8322;648;55273;114299;64784;5939;54796;51341;9507;80333;10664;58533;4784;25890;1285;1009;92949;143686;596;351;9462;8470;7091;54453;1286;10395;23394;4040;4052;58487;7094;151011;6095;151742;3097;55728;5581;84102;4638;10499;23332;221037;80315;659;11217	ABI3BP;ACVR1;ADAMTS4;ADAMTSL1;ADNP;AKAP2;APP;ASPA;BAHD1;BCL2;BMI1;BMPR2;BNC2;BRCA2;CASK;CDH11;CGGBP1;CLASP1;COL4A3;COL4A4;CPEB4;CREBZF;CRTC3;CTCF;DLC1;DST;FOSL2;FOXO4;FOXP2;FZD4;GDNF;GPR82;GSK3B;HIVEP2;HOXB4;JMJD1C;KCNIP4;LRP6;LRRC15;LTBP1;MED13;MITF;MYLK;N4BP2;NCOA2;NDRG2;NFIX;NTRK2;PALM2;PBX1;PDE4B;PPM1L;PRDM1;PRDM10;PRICKLE2;PRKCE;PURA;RASAL2;RBMS2;RIN2;RORA;RORB;RP1;RYBP;SEPT10;SESN3;SLC41A2;SNX6;SORBS2;TCF21;TLE4;TLN1;TMEM100;TNKS1BP1;UNC13B;ZBTB10;ZBTB7A

TTGTTT_V$FOXO4_01	http://www.broadinstitute.org/gsea/msigdb/cards/TTGTTT_V$FOXO4_01	-0.46813	-1.76071	0	5.77E-04	1879	649	51266;10521;133584;4974;253943;10943;1592;9099;3673;152137;26191;2257;199870;64084;201798;146691;28513;22903;5663;23142;50484;29982;3643;7862;255082;953;11036;64067;84333;23180;10890;6310;5140;9846;9734;3983;4916;605;2354;55818;9455;8444;9120;22797;3696;301;1277;154796;122011;23466;4774;8577;139818;54739;3781;653;51705;3174;55284;1836;716;22848;7224;159;84928;10972;10076;11278;10586;151887;23336;158866;257068;10857;860;2844;7043;23460;64399;89795;9874;9457;5648;10018;140885;80124;3480;388;56000;2674;85464;10205;5602;1879;4303;2869;3918;170685;51444;9957;1278;143279;221687;1848;639;2624;53371;8473;3479;3710;55784;7472;5087;54910;2798;2202;84251;79872;23039;1600;9857;1831;10439;5156;220388;80231;9706;23057;26298;5587;	AAK1;ABCA6;ABLIM1;ACVR1;ACVR1B;ADSS;AGL;AKT3;AMFR;AMOT;ANGPT1;ANK3;ANKRD28;ANO3;ANXA1;APBA1;APC;APLP2;APOLD1;APP;APPBP2;ARFIP1;ARHGAP20;ARHGAP6;ARHGEF6;ARID1B;ARID4A;ARID4B;ARRDC3;ASB7;ASPA;ATF6;ATF7;ATF7IP;ATP10D;ATP2A2;ATP6V1A;ATXN1;BCL2;BCL2L11;BCL2L2;BCL7A;BCOR;BDNF;BICD1;BIRC6;BMI1;BMP5;BMPR2;BMX;BNC2;BPTF;BRD3;BRIP1;BRPF1;BTBD3;C1QTNF7;C1S;C21orf91;C2orf69;C3orf36;CACNA2D1;CASC2;CASQ2;CAV2;CBLB;CBLL1;CBX6;CCDC50;CCDC80;CCDC89;CCNG2;CCPG1;CDC14B;CDH11;CDH19;CDH5;CDK17;CDKL5;CEP120;CEP350;CEP97;CGGBP1;CHD1;CHD2;CHD6;CHMP1B;CHN1;CITED2;CLCC1;CLEC1B;CLINT1;CLK4;CLPX;CLSTN2;CMTM6;CNTF;COL1A1;COL1A2;COL8A1;CPEB3;CPEB4;CPOX;CREBL2;CRIM1;CRY2;CSNK1A1L;CSNK1G3;CTNNB1;CTNND1;CTR9;CTTNBP2NL;CXCL12;CXXC5;CXorf21;CYB5D1;CYP26A1;DAAM2;DAB1;DACT1;DCN;DCUN1D3;DCUN1D4;DDX17;DDX6;DENND1B;DENND2C;DENND4A;DICER1;DIX

								27197;3037;9414;10100;57396;1499;2822;3667;7175;5187;6096;1740;10913;5066;55809;140460;7763;55205;23710;8554;4094;4978;11080;26249;57132;93986;1123;8861;57561;858;8493;93034;627;5074;8864;7074;1981;4908;6451;284;7957;80705;6660;4915;54708;55512;7096;9464;11343;1969;9053;57532;80310;51339;3842;63982;84189;6387;5350;11342;353345;10418;7273;1801;22926;7148;4781;10370;80111;3790;84084;10140;6567;2627;79884;5155;23155;1408;5782;10891;2335;9674;54538;6101;7572;51100;3351;54407;84188;267;55609;8013;153241;9686;221935;182;443;6529;23503;55959;10509;3597;79807;4208;85458;342372;117289;4026;90;3753;9770;5099;7528;83990;55553;26959;8545;59345;288;23387;1958;5500;4286;30061;29979;5136;55167;25891;8036;10513;4091;9698;5915;2977;231	DC1;DLC1;DLG2;DLL1;DMD;DMXL1;DNAJB14;DNAJB4;DNAJC22;DOCK11;DOCK4;DOCK9;DPH1;DST;DUSP1;DUSP6;DYRK1A;DYRK3;EBF1;EBF2;ECM2;EDAR;EFEMP1;EGFLAM;EGR1;EGR2;EHBP1;EHF;EIF2AK3;EIF4G1;EIF4H;ELF1;EMCN;EML1;EMP1;ENTPD1;EP300;EPHA2;EPM2A;ERG;ETS1;ETS2;EXOC5;FAM107B;FAM124B;FAM76A;FAR1;FAT4;FBXO11;FCHSD2;FGD4;FGF12;FGF7;FHL5;FILIP1;FLI1;FLT1;FMNL3;FN1;FOSB;FOSL2;FOXN3;FOXO1;FOXO4;FOXP2;FRMD4A;FRMPD1;FRS2;FRY;FSTL1;G2E3;G3BP2;GAB2;GABARAPL1;GARNL3;GATA2;GATA6;GCC2;GFRA1;GHR;GLUD2;GNAO1;GNAQ;GNB4;GNE;GNRHR;GOLGB1;GPLD1;GPM6A;GPR141;GPR21;GPR82;GPRIN3;GRAMD1C;GRK5;GSTCD;GTF2A1;GTF2A1L;GUCY1A2;HAND2;HAS2;HBP1;HDAC9;HECTD2;HHIP;HIF1A;HIVEP1;HMCN1;HNF4G;HOMER2;HOXB3;HS3ST1;HSPG2;HTR1B;IFIH1;IGF1;IGF1R;IKZF2;IKZF3;IKZF4;IL13RA1;IL18R1;IL6ST;IL7R;INHBA;INHBC;INO80D;INPP4A;INSR;IREB2;IRS1;ITGA1;ITGA10;ITGA2;ITGB8;ITPR1;ITPR3;JAG1;JMJD1C;KANK1;

								89;79815;9497;132671;223082;91;64135;7067;144348;29951;9646;84898;4929;6943;57620;6586;91010;81575;3213;7223;1634;55119;5813;59350;1112;260425;22844;266812;1371;286016;55691;6777;25780;23469;6774;4958;334;1959;5209;5159;26137;6667;4756;114905;2355;57161;1843;5928;4090;2747;79843;202018;845;339122;667;51232;868;660;10129;1842;9830;124637;648;636;8829;9043;4643;8515;5069;1456;8828;54762;2823;5166;5090;5939;84295;201799;23054;57610;25777;11167;54796;11043;80333;22992;4734;54989;22807;51523;10463;253827;64324;2313;2009;5534;2122;2775;4784;1389;2114;2252;6304;254827;64795;83872;80031;8555;901;23637;114879;284273;4131;54510;55016;4041;1270;64393;1009;395;7337;9794;357;55632;10818;320;4215;205327;132660;23314;55276;523;2851	KCNE1;KCNIP4;KCNN2;KCNS3;KDM2A;KDM3A;KDM5A;KDM6A;KIAA0040;KIAA0586;KLF12;KLF3;KLF7;KLF8;KLHL20;KLHL24;KLHL3;LAMC2;LDB1;LDB2;LIN54;LPP;LRP5;LRP6;MAB21L2;MAF;MAGI1;MAGI3;MAML1;MAML2;MAML3;MAN1A1;MANEA;MAP1B;MAP3K13;MAP3K3;MAP4K5;MAP7;MAP9;MAPK10;MAPK8;MARCH1;MARCH5;MASP1;MAST4;MBD5;MBNL2;MCTP2;MECOM;MEF2C;MGLL;MID2;MITF;MOSPD2;MPZL2;MSL2;MSL3;MSRB3;MTF1;MYLK;MYO18A;MYO1E;MYOCD;NAALADL2;NAP1L5;NAV3;NCOA6;NEDD4;NEK1;NEO1;NF1;NFE2L2;NFIA;NFIB;NFIX;NIPAL2;NIPBL;NMNAT2;NNT;NOS1AP;NOS3;NPAS3;NR2C2;NR2F2;NR3C1;NR3C2;NR4A2;NR4A3;NRBF2;NRP1;NRP2;NSD1;NT5C1B;NTF3;NTN4;NTRK2;NTRK3;NUDT10;NUFIP2;NUP54;NXF3;OGT;OLFM1;OMD;OMG;OPCML;OSBPL5;PAM;PAMR1;PAPPA;PAWR;PBX1;PBX3;PCDH17;PCDH18;PCDH7;PCF11;PCGF5;PDE1A;PDE3B;PDGFB;PDGFD;PDGFRA;PDGFRB;PDK4;PDPR;PDZRN4;PELI2;PER1;PER2;PFKFB3;PGM2;PGRMC1;PHF3;PHF6;PIAS1;PIK

								4;4846;10580;91746;9467;143686;6446;3626;596;90627;7458;26009;157680;2186;351;9185;53349;6671;1105;55534;8671;54880;4520;55777;59277;178;285513;2308;8470;163486;1756;55917;5727;9722;6654;27314;65267;2804;51742;25862;84614;100505741;4306;54918;5294;10845;2321;23530;8809;5927;55761;7750;10395;8019;93649;79652;10628;3631;54891;84146;27145;6505;152006;10735;7403;64375;9873;6814;29066;10150;54843;57169;9732;6886;79962;26054;23174;123879;4040;84253;4780;9786;51274;3624;7764;25925;6733;389136;1656;29766;25836;23186;79634;54149;79598;3575;5412;55014;79694;112574;84068;599;6645;10020;9372;11099;9175;83641;22872;163259;1295;9908;10955;7082;5870;6925;3091;27236;80243;51422;7871;9839;22806;55279;57569;64641;10260;11183;2908;7981	3C2A;PIK3CG;PIK3R1;PKD1L3;PKN2;PLCXD2;PLN;PLXDC2;PPARGC1A;PPM1B;PPM1D;PPM1L;PPP1CB;PPP3R1;PRDM1;PREPL;PREX2;PRKAG2;PRKD1;PROX1;PRPF38B;PSEN1;PTCH1;PTPN12;PTPN21;PTPN22;PTPRM;PTPRU;PUM1;PURA;PXK;QKI;RAB10;RAB30;RAB43;RAB6A;RAB6C;RABGAP1;RANBP10;RARB;RASGEF1B;RASGRP3;RASSF2;RBBP4;RBL2;RBM43;RBMS2;RCOR1;REPS2;REST;RFTN1;RHOB;RMND5A;RNF111;RNF13;RNF138;RNF182;RNF38;ROBO4;ROCK1;RORA;RORB;RP1;RREB1;RRM2B;RRN3;RSF1;RUNX1T1;RUNX2;RXFP1;S1PR1;SASH1;SATB1;SATB2;SCRN3;SDK1;SEC24B;SEC31A;SEMA4B;SEMA4C;SEMA6D;SENP6;SERINC3;SESN3;SGIP1;SGK1;SH3BGRL;SH3BP5;SH3GLB1;SHOC2;SHROOM2;SIK3;SIPA1L1;SIRPA;SLC10A7;SLC12A6;SLC16A2;SLC16A6;SLC1A1;SLC26A2;SLC30A9;SLC38A2;SLC40A1;SLC4A4;SLC4A7;SLC6A1;SLIT3;SLITRK6;SLMAP;SLTM;SMAD1;SMAD5;SMAD6;SMARCA2;SMG1;SMPD3;SNTB2;SNX18;SORBS1;SORBS2;SOS1;SOX5;SOX6;SP1;SP4;SPAG9;SPATA1;SPATA18;SPON1;SRPK2;SSH2;

								1;80818;11279;27253;57205;7026;11016;5495;4121;255394;3658;9648;23243;781;9685;23500;8609;9079;54521;54899;7414;80204;488;26037;23405;9581;9444;55729;23301;4763;23414;158747;5797;2690;22849;25851;51585;2776;1901;2078;9459;23012;5586;3708;6095;151742;29761;10000;3672;5926;23534;54700;3096;4750;1106;1859;6595;7150;54800;84181;80351;10640;10274;9236;27327;5128;55066;153020;9990;4086;3339;324;163590;399687;2012;54778;23049;1003;7010;8615;6792;57492;55787;375449;9451;51773;5599;4638;1500;7003;5629;2033;7328;5295;6239;2113;1657;10427;23190;221037;79982;121512;27252;5934;80315;6093;84441;1997;7182;23328;79633;862;2957;163049;9223;659;57448;5286;167465;146057;375287;23348;3572;5978	STAG1;STAG2;STARD13;STAT3;STAT5B;STIM2;STK38L;STX17;STXBP3;SULF2;SUN2;SYNM;SYTL2;TAGAP;TAL1;TAPT1;TCF21;TCF4;TCP11L2;TEAD1;TECPR1;TEK;TFEC;TGFB3;THRA;TIAM1;TIGD4;TJP1;TJP2;TLK1;TLR1;TMED10;TMEFF1;TMEM154;TMEM204;TMEM209;TMOD3;TNKS2;TNPO1;TNPO3;TNRC6A;TNXB;TOB1;TOM1L2;TOP1;TOR1AIP2;TPI1P2;TPR;TRERF1;TRIM14;TRPC4;TRPC5;TSC22D3;TSGA10;TSPAN2;TTBK2;TTC17;TTN;TXLNG;TXNIP;UBE2H;UBE2W;UBE3A;UBL3;UBQLN1;UBXN4;ULK2;USO1;USP2;USP25;USP49;VCL;VCPIP1;VGLL3;VGLL4;VPS13B;WDR44;WNK3;WNT2;XAF1;XPO7;YTHDC1;YTHDF3;YY1;ZADH2;ZBTB20;ZBTB37;ZC3H7A;ZCCHC14;ZDHHC15;ZEB2;ZFAND5;ZFPM2;ZFYVE1;ZFYVE26;ZFYVE9;ZMAT3;ZMYM2;ZNF217;ZNF24;ZNF280C;ZNF366;ZNF436;ZNF521;ZNF532;ZNF644;ZNF654;ZNF664;ZNF770;ZNF791;ZNFX1;ZNRF2;ZZZ3
V$DBP_	http://www.b	-0.53341	-1.76134	0	5.97E	227	73	4162;5991;3483;153090;79365;	ARID4A;ATP2C1;AUTS2;BHLHE41;CAL

Q6	roadinstitute.org/gsea/msigdb/cards/V$DBP_Q6				-04			2674;23193;143279;1848;3710;5861;2822;1740;93986;10140;1408;10891;8013;6098;5520;3707;57154;4286;4330;6943;5813;266812;54505;55294;8515;2823;257397;4784;4154;27032;132660;6840;285513;8642;8470;1756;5727;7091;23369;84614;9955;10150;51274;10725;6526;6733;57120;55014;22918;51429;26053;57616;9839;64641;5793;11183;27253;8609;23037;2078;5926;6595;9710;50618;4638;800;220965;7328	D1;CD93;CRY2;DAB2IP;DCHS1;DHX29;DLG2;DMD;DUSP6;EBF2;ERG;FAM13C;FBXW7;FOXP2;GANAB;GFRA1;GOPC;GPLD1;GPM6A;GPRIN3;HECTD2;HS3ST3A1;IGFALS;ITGA10;ITPKB;ITPR3;ITSN2;KIAA0355;KLF3;KLF7;LIN54;MAP4K5;MBNL1;MBNL2;MCAM;MITF;MN1;MYLK;NAP1L5;NFAT5;NFIX;NR4A3;PCDH17;PDZD2;PPARGC1A;PPP2R2A;PTCH1;PTPRG;PUM2;PURA;RAB1A;RFX3;ROS1;SLC5A3;SMARCA2;SMURF1;SNX9;SORBS2;SRPK2;STX17;SVIL;TAB3;TCF21;TLE4;TOB1;TSHZ3;UBE2H;ZBTB37;ZEB2
V$GATA6_01	http://www.broadinstitute.org/gsea/msigdb/cards/V$GATA6_01	-0.54028	-1.81249	0	5.97E-04	247	89	4641;7227;57631;222663;54875;157769;64388;9066;6781;8848;84674;8645;3953;23136;7025;5362;7832;51043;23180;5140;55818;112;54206;25909;151556;11278;860;5648;358;3574;2628;84251;5156;54897;6096;23509;80168;6660;81704;2627;10891;4208;57522;26468;6642;55160;81848;3213;344148;2355;783;4090;257397;8826;51341;10664;80177;1285;10743;116984;64	ADCY6;AHCTF1;AQP1;ARAP2;ARHGEF10L;BRWD3;BTG2;CACNB2;CARD6;CASZ1;CDC42EP3;CMTM6;CNTLN;COL4A3;COL4A4;CTCF;DDR2;DENND1B;DOCK8;EPB41L3;ERG;ERRFI1;FAM107B;FAM91A1;FOSL2;GATA6;GATM;GPR155;GREM2;HOXB3;IGFBP5;IL7;IQGAP1;ITPR1;KCNK5;KDM3A;KDM6A;KLF12;LEPR;LEPROT;LHX6;LIMS1;LRCH2;MASP1;MEF2C;MOGAT2;MYCT1;MYO1C;NCKAP5;NR2F1;NR2F2;PDE3B;PDGFRA;PDZD2;PLXNA2;POFUT1;PPARGC1A;PTPRG;P

								22;10580;23516;163486;7091;23369;1286;54918;7403;6867;29072;83641;10602;254065;5793;3488;6498;7026;255394;23414;23037;2078;222194;3708;3987;4921;7328;862;54741	UM2;RAI1;RFTN1;RORB;RSBN1L;RUNX1T1;RUNX2;SCUBE3;SETD2;SFRP1;SGIP1;SKIL;SLC39A14;SMAD5;SNX1;SORBS1;SOX5;SPRY4;SRGAP1;STC1;SYT7;TAB3;TACC1;TCP11L2;TLE4;TRPS1;TSC22D1;UBE2H;ZBTB7A;ZBTB7B;ZFPM2
CATTGTYY_V$SOX9_B1	http://www.broadinstitute.org/gsea/msigdb/cards/CATTGTYY_V$SOX9_B1	-0.5334	-1.82891	0	5.97E-04	337	133	1026;10584;51062;7072;7862;9734;53354;149420;4804;8509;51294;9348;30850;28951;3781;3290;653;22846;153090;57447;166614;10018;2674;64116;2869;639;3479;5861;5087;2260;10439;5024;57533;1740;7763;93986;1123;8864;7074;6660;57532;3842;5782;29123;57522;9770;288;4286;26468;79815;55596;1316;4330;55334;144348;84898;6774;2355;23095;3839;124637;9043;23219;84295;25777;54796;23047;2313;2775;8289;80177;5775;26051;4131;54510;23321;1270;4854;7337;9794;8661;6422;83737;59338;2186;4155;139324;8470;1756;374655;5583;10395;26091;23394;54843;9732;25932;8301;3624;25925;89796;348	ADNP;ANK3;ANKRD11;ARID1A;ATL1;BCL2L11;BMP5;BNC2;BPTF;BRPF1;CALD1;CDK8;CDKN1A;CDR2L;CHN1;CLIC4;CLOCK;CNTF;COLEC10;CYB5D1;DAB2IP;DCLK2;DENND2C;DLC1;DLG2;DMD;DOCK4;DYRK1A;EHBP1;EIF3A;ERG;ETS1;EXOC5;FAM13C;FAT4;FBXO11;FBXO28;FGD4;FGFR1;FLI1;FOSL2;FOXP2;G3BP2;GFRA1;GNAO1;GRK5;HDAC9;HDX;HERC4;HSD11B1;HSPG2;IGF1;IGF2R;INHBA;ITCH;KCNN2;KIF1B;KLF6;KPNA3;LHX6;MAML1;MAP1B;MBP;MITF;MN1;MOSPD2;MYCT1;MYLK;MYO18A;NAV1;NDRG2;NDST2;NDST3;NGFR;NIPAL2;NOTCH3;NUFIP2;OLFM1;P2RX3;PAFAH1B2;PANK1;PATL1;PBX1;PCDH12;PCDH18;PDIK1L;PDS5B;PER2;PHF6;PICALM;PLEKHA1;PLXDC2;PPP1R16B;PRDM1;PRKCH;PTPN12;PTPN4;RAB1A;RAPH1;RASSF2;RREB1;SERINC3;SFRP1;SLC25A40

								2;8467;65059;163259;9908;10955;6925;80818;6619;7414;80204;23301;158747;2078;1859;7150;10640;55972;1024;3339;399687;219988;4638;800;7003;220965;6239;2113;121512;5049;79633;9575	;SLC39A8;SLC39A9;SMARCA5;SNAPC3;SORBS2;SOX5;SPAG9;SRGAP1;STAT3;SUN2;SYTL2;TBC1D14;TCF4;TEAD1;TIA1;TIAM1;TNPO1;TOP1;TRIB2;TRIM2;UBE3A;VASH1;VCL;ZCCHC8;ZFAND5;ZNF436;ZNF521;ZNF664;ZNF710
V$AP3_Q6	http://www.broadinstitute.org/gsea/msigdb/cards/V$AP3_Q6	-0.56474	-1.85704	0	5.97E-04	220	79	5140;4916;84239;23213;653;10420;8573;9628;5648;114799;348654;6869;196;55142;6678;94134;93986;1981;2936;4915;2335;1909;55297;26468;5218;27302;81848;5737;1398;255252;166785;79677;23469;344148;4756;8829;5069;11167;64324;2313;58533;4154;1285;10928;84669;8028;7473;1756;1286;93649;2028;6886;8301;6526;1656;29110;599;64641;8139;11069;23414;8633;6095;151742;3096;60485;1106;84181;4086;166929;7799;219988;57584;4638;1500;800;56103;221037;2081	AHR;ARHGAP12;ARHGAP21;ATP13A4;BCL2L2;BMP10;BMP5;CALD1;CASK;CCDC91;CDK14;CHD2;CHD6;COL4A3;COL4A4;CRK;CTNND1;DDX6;DMD;EBF2;EDNRA;EIF4G1;ENPEP;ERN1;ESCO1;FLI1;FN1;FOXP2;FSTL1;GAN;GEN1;GSR;HAUS2;HIVEP1;JMJD1C;LHX6;LRRC57;MASP1;MBNL1;MLLT10;MMAA;MYLK;MYOCD;NCKAP5;NEO1;NRP1;NSD1;NTRK2;NTRK3;PAPPA;PATL1;PCDHGB2;PDE3B;PHF3;PICALM;PPM1L;PRDM2;PTGFR;RALBP1;RAPGEF4;RGS6;RORA;SAV1;SGMS2;SLC5A3;SMAD1;SMC6;SNX6;SPARC;SPRY4;SULF1;TACR1;TAL1;TBK1;TESK2;UNC5C;USP32;WNT3;ZFPM2
V$FOXO4_02	http://www.broadinstitute.org/gsea/msi	-0.53411	-1.76208	0	6.17E-04	237	97	2257;201798;3643;255082;11036;64067;6310;4916;605;55818;3696;5991;11278;7043;2674;56	ACVR1B;ARFIP1;ARID1B;ARID4A;ATXN1;BCL7A;BDNF;CACNA2D1;CASC2;CCDC89;CHD2;CITED2;CLPX;COL8A1;C

	gdb/cards/V $FOXO4_02							02;9957;639;3214;23039;220388;3037;55809;93986;8861;627;7074;80310;10370;182;85458;26959;5915;91;7067;29951;84898;4929;7223;5813;266812;4090;10129;5069;5166;55852;4734;22807;51523;2009;2775;4784;1389;83872;284273;54510;4041;1982;23314;28514;2308;5727;10845;10628;10735;7403;64375;9732;25925;79634;4826;84068;9372;1295;152485;5870;27236;7871;2908;781;2078;5586;5926;1106;54800;27327;153020;4086;2012;7010;57492;51773;7328;7357;23328;862;3572	REBL2;CXXC5;DIXDC1;DLL1;DOCK4;EIF4G2;EML1;EMP1;ERG;FGF12;FOXO1;FOXP2;FRY;GFRA1;GNAO1;GTF2A1L;HAS2;HBP1;HMCN1;HOXB4;HS3ST1;IKZF2;IKZF4;IL6ST;INSR;ITGB8;JAG1;KDM3A;KDM6A;KLF12;KLHL24;LDB1;LRP5;MAPK10;NAP1L5;NEDD4;NFIX;NNAT;NPAS3;NR3C1;NR4A2;NTRK3;PAPPA;PCDH18;PDGFD;PDK4;PDZRN4;PKN2;PLXDC2;PRDM1;PTCH1;PURA;RAB6A;RARB;RASGEF1B;RFX3;RSF1;RUNX1T1;SASH1;SATB2;SCRN3;SLC10A7;SLMAP;SMAD1;SMAD5;STAG2;TEK;TEX2;TGFB3;THRA;TIAM1;TIGD4;TNRC6A;TRERF1;TRPC4;TXNIP;UBE2H;UGCG;XPO7;ZADH2;ZFYVE9;ZNF521;ZNF827
V$HNF3ALPHA_Q6	http://www.broadinstitute.org/gsea/msigdb/cards/V$HNF3ALPHA_Q6	-0.54301	-1.76301	0	6.39E-04	194	73	54206;8509;55286;4774;115426;1305;84928;11278;7043;89795;2660;64116;1848;639;23057;23710;4094;56963;93986;64328;4007;11343;10140;2627;8013;4208;3707;5500;10006;3213;5813;1959;4756;2823;223;27097;9068;22887;2775;83872;284273;4041;6446;2800;9185;7174;285513;1756;23369;84614;4163;74	ABI1;AKT3;ALDH9A1;ANGPTL1;C4orf19;CALD1;CDC42EP3;CHD2;CHD6;COL13A1;DMD;DUSP6;EBF2;EGR2;ERRFI1;FOXJ3;FOXP2;GABARAPL1;GATA6;GNAO1;GOLGA1;GPM6A;GPRIN3;GTF2A1;HMCN1;HOXB3;ITPKB;KDM6A;KLF12;KLF3;LRP5;MAF;MAP3K13;MCC;MEF2C;MGLL;MSTN;NAV3;NDST2;NEO1;NFIA;NMNAT2;NR4A3;PATL1;PCDH17;PIK3C2A;PPP1CB;PRDM1;PRICKLE3;PTPRG;PU

								03;51274;389136;83891;9175;57616;7082;10602;64641;5793;80818;27253;10000;1106;6595;84181;54790;219988;800;6239;2957;5286	M2;PURA;REPS2;RGMA;RREB1;SGK1;SLC39A8;SMARCA2;SNX25;TAF5L;TET2;TGFB3;TJP1;TMEM209;TOB1;TPP2;TSHZ3;UHRF2;VGLL3;XPO4;ZADH2;ZBTB37;ZNF436
KMCATNNWGGA_UNKNOWN	http://www.broadinstitute.org/gsea/msigdb/cards/KMCATNNWGGA_UNKNOWN	-0.62615	-1.81295	0	6.39E-04	77	37	1452;1848;23786;8729;57533;1740;1969;57532;8434;121536;8545;8976;406;81575;83861;54556;92949;9655;1756;5727;25;10042;27107;200734;8491;6670;7026;164;10274;151987;130507;23161;9411;10194;7592;862;23528	ABL1;ADAMTSL1;AEBP2;AP1G1;APOLD1;ARHGAP29;ARNTL;BCL2L13;CGGBP1;CSNK1A1;DLG2;DMD;DUSP6;EPHA2;GBF1;HMGXB4;ING3;MAP4K3;NR2F2;NUFIP2;PPP4R2;PTCH1;RECK;RSPH3;RUNX1T1;SNX13;SOCS5;SP3;SPRED2;STAG1;TBC1D14;TSHZ1;UBR3;WASL;ZBTB11;ZNF281;ZNF41
V$LHX3_01	http://www.broadinstitute.org/gsea/msigdb/cards/V$LHX3_01	-0.56978	-1.85313	0	6.39E-04	209	66	23213;3778;146760;131578;79365;64399;219699;22836;639;3710;56256;115294;3037;54897;6096;7483;284;3782;10659;57403;10140;10512;1602;55959;85458;94122;5915;8082;29951;406;26137;4756;51088;8829;122953;2199;2775;4883;4154;1285;8503;1756;7091;1286;6873;8516;6733;1656;4853;57616;11177;9839;64641;8609;9079;57669;6095;27327;1540;7007;6792;5576;5290;6239;5592;167465	ANGPT1;ARNTL;BAZ1A;BHLHE41;CASZ1;CDKL5;CELF2;COL4A3;COL4A4;CYLD;DACH1;DDX6;DIXDC1;DMD;EBF2;EPB41L5;FBLN2;GNAO1;HAS2;HHIP;ITGA8;ITPR3;JDP2;KCNMA1;KCNN3;KLF7;KLHL5;LDB2;LRRC15;MBNL1;NEO1;NOTCH2;NPR3;NRP1;PCMTD1;PDZRN4;PIK3CA;PIK3R3;PRDM1;PRKAR2A;PRKG1;RAB22A;RARB;RHOBTB3;RORA;RORB;RREB1;RTN4RL1;SEMA3C;SERTAD4;SRPK2;SSPN;SULF1;SULF2;SYTL5;TAF2;TECTA;TLE4;TNRC6A;TOB1;TSHZ3;UNC5B;WNT9A;ZBTB20;ZEB2;ZNF366

SCGGAAGY_V$ELK1_02	http://www.broadinstitute.org/gsea/msigdb/cards/SCGGAAGY_V$ELK1_02	0.49021	1.830753	0	0.001004	1076	306	1460;4201;51406;10471;9277;116138;55168;63943;6155;5437;9533;6222;6181;140823;11267;6628;6635;51504;84336;8625;6167;6208;6882;6143;6161;10436;51255;6168;5719;122704;9587;56915;51070;339487;51264;25804;27166;7266;200185;4738;6193;51035;3704;8815;54663;51651;51645;54460;1163;6160;26574;6881;5685;59286;6128;8409;1175;116092;6135;1340;91582;84522;6188;29081;84545;5700;22827;10574;6015;26519;10452;25824;10204;9092;10641;5687;10093;55173;5695;6388;81605;10726;23583;23658;54555;54949;9553;147007;1933;55850;6742;10445;27339;55486;5435;140465;27243;6230;84727;6194;10695;283899;56342;5531;5689;56616;53917;57510;28991;79159;10063;56993;6396;5686;84975;146956;11313;1337;6189;51506;5704;83461;5440;3326;6903;170622;30968;51503;516	AATF;ACYP1;AIP;AKT1S1;ALDOA;AMZ2;ANKS3;AP2S1;AP4M1;APTX;ARFGAP1;ARFIP2;ARMC7;ARPC4;ASB6;AURKA;B3GALT6;B3GAT3;BAD;BANF1;BZW2;C12orf57;C14orf119;C19orf47;C1orf122;CBX8;CCDC71;CCDC85B;CCT7;CD2BP2;CDC123;CDC37;CDC45;CDCA3;CHMP2A;CKS1B;CLN3;CNOT10;CNPY3;COMMD5;COMMD6;COPE;COPS3;COX17;COX5B;COX6A1;COX6B1;COX8A;CPSF3;CSNK2B;CTNNBL1;CUTC;CWC15;CXXC1;CYB561D2;DDIT3;DDOST;DDX49;DGUOK;DIABLO;DNAJC7;DNTTIP1;DPCD;EBNA1BP2;EEF1B2;EEFSEC;EFTUD2;EIF1AD;EIF3H;EIF4A1;EIF5A;EME1;EMG1;ERCC1;ERH;EXOSC3;EXOSC5;FBXL6;FBXW9;FIBP;FKBPL;FOXH1;GAPDH;GAR1;GPN2;GRWD1;GTF2A2;HARS;HSP90AB1;HSPA4;IFI30;INO80B;INO80E;ITGB1BP1;ITPA;JAGN1;KIF4A;KIF9;KLHDC3;KRTCAP2;KTI12;LAS1L;LLPH;LMAN2;LSM4;LSM5;LYPLA2;MAD2L1BP;MCM7;MCRS1;MCTS1;MEA1;MED30;MED8;METTL5;METTL6;MFSD5;MORN2;MOSPD3;MPDU1;MRPL27;MRPL33;MRPL40;MRPL43;MRPL52;MRPS10;MRPS18A;MRPS

								92;51157;7388;55135;8667;84292;54433;79797;1201;6949;25911;64976;80324;1329;11333;7391;55738;51548;84335;27343;112950;113246;7922;84298;10948;55696;10421;64419;51371;55017;25915;55621;11316;11068;6154;84759;60678;140739;1716;729967;51006;1351;64601;5886;6166;221504;51076;10960;81559;79023;83743;5984;6727;64147;2597;6810;10616;56949;29101;4913;81887;2067;9049;9409;4176;58485;26229;23647;124641;89891;9343;55611;84261;6992;83707;10969;10474;6728;56259;9158;25904;1984;126792;5982;10262;6293;10016;11164;2079;1973;79637;7341;84285;124401;29100;10946;2958;90390;9526;84065;28985;57215;26155;54840;8872;54707;83590;51399;8318;26073;26233;25880;10921;1649;83444;10910;11007;6257;140686;30827;51248;9179;9270;7284;84844;7336;5190;	21;MTMR14;MYL6B;NAGK;NASP;NDUFAF3;NEDD8;NKIRAS2;NOC2L;NOL12;NOL7;NOSIP;NPRL2;NTHL1;NUDC;NUDT5;NUP37;NUTF2;OGG1;OTUB1;OVCA2;PABPC1;PARL;PCGF1;PDAP1;PDCD6;PDLIM1;PDZD11;PEX16;PEX6;PFDN6;PHF5A;PIGC;PIGT;POLDIP2;POLL;POLR1C;POLR2F;POLR2H;POLR2K;POLR3H;POMP;PPAN;PPIL1;PPP1R11;PPP4C;PRDX5;PRELID1;PRPF19;PSMA4;PSMA5;PSMA6;PSMB1;PSMB7;PSMC1;PSMC4;PSMD13;PTRH2;PUF60;PUS1;RAB24;RABGEF1;RAD23A;RBCK1;RBM22;RFC2;RFC4;RFXANK;RING1;RNF181;RNPS1;ROMO1;RPL11;RPL19;RPL26;RPL27;RPL31;RPL32;RPL36AL;RPL37;RPL37A;RPL6;RPLP2;RPS14;RPS18;RPS19BP1;RPS25;RPS3;RPS3A;RPS5;RPS6;RRS1;RXRB;SART1;SCAMP2;SDF2;SDHAF2;SEC11A;SEC13;SF3A3;SF3B4;SH3GLB2;SIRT3;SIRT6;SLC25A1;SLC35C2;SLC39A7;SMUG1;SNF8;SNRPB;SNRPE;SPSB2;SRP14;SRP19;SSBP1;SSU72;STARD3;STOML2;STX10;STX4;SUGT1;SUMO1;SUPT5H;TADA3;TAF10;TAF11;TBC1D13;TBCC;TCOF1;THAP11;TIMM10;TMEM101;TMEM186;TMEM199;T

								79759;54662;57332;112970;4968;572;23410;56658;11140;3035;51060;27342;22888;8533;10437;1650;23212;3308;127687;6829;6576;84124;226;26986;23478;51010;4678;8928;131965;28511;24137;51321;6790;5279;28969;171568;140459;22974;9804;9124;51604;64598;126526;56904;8677;97;64925;10066;85437;55577;8078	MEM208;TMEM222;TMUB1;TOMM20;TOMM22;TOMM40;TPX2;TRAPPC1;TRAPPC4;TRIM11;TRIM39;TRMT1;TRMT112;TRPT1;TUFM;TXNDC12;UBE2F;UBE2V2;UBL5;UBOX5;UBXN1;UFC1;UQCRH;URM1;USE1;USF1;USP5;UXT;VPS16;VPS52;WDR34;WDR46;WDR74;WDR83;WFDC3;WRAP53;XAB2;XPO5;ZBTB8OS;ZBTB9;ZCRB1;ZNF394;ZNF408;ZNF580;ZNF668
V$ELK1_02	http://www.broadinstitute.org/gsea/msigdb/cards/V$ELK1_02	0.473799	1.586339	0	0.023254	226	57	4201;116138;6222;11267;6628;6167;6143;7266;200185;54663;51651;1163;26521;1175;5700;10452;3159;23583;23658;1933;140465;28998;56616;6396;11313;6903;30968;51692;7388;79797;25911;51548;84335;27343;112950;84298;55696;51371;55621;55272;1351;10960;79078;5984;10450;84261;6728;1984;6293;29100;23421;2958;7332;8318;83444;9270;7284	AKT1S1;AP2S1;C1orf50;CDC45;CKS1B;COX8A;CPSF3;DIABLO;DNAJC7;DPCD;EEF1B2;EIF5A;FBXW9;GTF2A2;HMGA1;IMP3;INO80B;ITGB1BP1;ITGB3BP;KLHDC3;KRTCAP2;LLPH;LMAN2;LSM5;LYPLA2;MEA1;MED8;MRPL13;MYL6B;POLL;POMP;PPIE;PSMC1;PTRH2;RBM22;RFC4;RPL19;RPL37;RPS18;SEC13;SIRT6;SMUG1;SNF8;SNRPB;SRP19;STOML2;TBCC;TIMM8B;TMEM208;TOMM40;TRMT1;TUFM;UBE2L3;UQCRH;VPS52;WDR74;ZNF408
V$NRF2_01	http://www.broadinstitute.org/gsea/msi	0.470791	1.601709	0	0.025429	248	63	1460;6204;6155;6222;51504;6167;10436;5719;6159;7266;6193;51035;54663;51651;1163;2652	AAAS;AKT1S1;B3GAT3;C14orf119;CBX8;CCDC85B;CD2BP2;CDC123;CKS1B;CSNK2B;CUTC;DAXX;DDIT3;DIABLO;DNAJ

	gdb/cards/V$NRF2_01							1;6128;55851;10452;25824;54949;27339;6164;1616;56616;56993;8664;51506;6903;7388;79797;7391;84335;8086;112950;10421;51371;55017;140739;51076;2067;26229;10467;84261;9158;1984;10262;199746;6293;11164;29100;2958;26155;8872;80153;10921;1649;11007;7284;113878;57332;112970;23410	C7;DTX2;EDC3;EIF3D;EIF5A;EMG1;ERCC1;FBXW9;FIBP;GTF2A2;KTI12;MED8;NOC2L;NUDT5;POMP;PRDX5;PRPF19;PSENEN;PSMD13;PTRH2;RNPS1;RPL27;RPL29;RPL34;RPL37;RPL6;RPS10;RPS18;RPS5;SDHAF2;SF3B4;SIRT3;TBCC;TIMM8B;TMEM208;TOMM22;TOMM40;TRMT112;TUFM;U2AF1L4;UBE2F;UBXN1;UFC1;UQCRH;USF1;VPS52;WDR74;ZNF408;ZNHIT1
V$MYCMAX_01	http://www.broadinstitute.org/gsea/msigdb/cards/V$MYCMAX_01	0.460296	1.543483	0	0.031184	235	54	5036;200185;10528;9274;6223;26519;26520;26528;3178;3159;2873;10726;1933;5902;56616;80142;1201;6949;64976;80324;3020;79077;3925;51602;4869;5216;7873;55646;1678;3276;403;5757;25879;10669;7866;51303;27037;83858;81890;55210;80758;51333;2719;26986;56888;7023;92579;10642;1050;84315;2130;8501;121268;2118	ARL3;ATAD3A;ATAD3B;BCL7C;CEBPA;CGREF1;CLN3;DAZAP1;DCAF13;DCTPP1;DIABLO;EEF1B2;ETV4;EWSR1;FKBP11;G6PC3;GPC3;GPS1;H3F3A;HMGA1;HNRNPA1;IFRD2;IGF2BP1;KCMF1;KRTCAP2;LYAR;MANF;MON1A;MRPL40;NOP56;NOP58;NPM1;NUDC;PA2G4;PABPC1;PFN1;PRMT1;PRR7;PTGES2;PTMA;PUS1;QTRT1;RANBP1;RHEBL1;RPS19;SLC43A1;STMN1;TCOF1;TFAP4;TIMM10;TIMM8A;TIMM9;TRMT2A;ZNF771
V$PPARG_01	http://www.broadinstitute.org/gsea/msigdb/cards/V$PPARG_01	0.561424	1.471509	0.026786	0.062068	40	12	6155;10436;79005;6636;10005;1933;10322;55937;2063;28957;140831;64925	ACOT8;APOM;CCDC71;EEF1B2;EMG1;MRPS28;NR2F6;RPL27;SCNM1;SMYD5;SNRPF;ZSWIM3

V$USF_C	http://www.broadinstitute.org/gsea/msigdb/cards/V$USF_C	0.424339	1.444026	0	0.071843	263	51	10471;9277;5437;5691;6143;2091;56915;5036;51035;6223;26519;26520;26528;10726;6146;23521;5441;3336;56616;5202;9521;3326;3151;7922;3020;3925;11337;51096;4869;5216;9343;55646;1984;2870;3276;5757;8704;51303;23404;2876;4904;10058;6257;51333;51060;3308;26986;7023;124044;92579;10642	ABCB6;B4GALT2;DAZAP1;DIABLO;EEF1E1;EFTUD2;EIF5A;EXOSC2;EXOSC5;FBL;FKBP11;G6PC3;GABARAP;GPX1;GRK6;H3F3A;HMGN2;HSP90AB1;HSPA4;HSPE1;IGF2BP1;LYAR;NPM1;NUDC;PA2G4;PABPC1;PFDN2;PFDN6;PFN1;POLR2H;POLR2L;PRMT1;PSMB3;PTMA;RPL13A;RPL19;RPL22;RPS19;RXRB;SLC39A7;SPATA2L;STMN1;TFAP4;TIMM10;TIMM9;TXNDC12;UBXN1;UTP18;WDR46;YBX1;ZNF771
RCGCANGCGY_V$NRF1_Q6	http://www.broadinstitute.org/gsea/msigdb/cards/RCGCANGCGY_V$NRF1_Q6	0.380937	1.415178	0	0.087204	812	198	1460;57819;64928;6633;5437;8625;56915;83463;7311;151903;6217;123207;4733;54543;51567;4726;6228;9136;1163;26574;5705;5685;7203;55957;6125;51614;27335;6235;84522;55851;28973;10248;63875;51192;84661;9040;26528;10898;3178;51293;1603;29093;8894;10641;10067;1345;125476;10445;84306;126382;3336;5435;27243;11070;3094;5252;57510;29796;51079;192286;25796;51491;10056;51692;10155;64979;283989;54814;10209;11340;56647;55657;64223	AATF;ABCB6;ABCF3;ABHD14A;ACOT13;ACTR1A;AKR7A2;AKT1S1;ANAPC16;ANP32A;AP1S1;ATAD3A;AURKA;B9D1;B9D2;BAD;BCCIP;C12orf65;C14orf119;C15orf40;CAPNS1;CBY1;CCDC107;CCDC12;CCDC127;CCDC97;CCNF;CCS;CCT3;CCT5;CD320;CDK10;CFL1;CHCHD4;CHMP2A;CKLF;CKS1B;CKS2;COX6C;COX7A2L;CPNE1;CPSF3;CPSF4;CRIPT;CSNK2B;CYB561D2;DAD1;DAZAP1;DDIT3;DPY30;DRG1;DYNLT1;EBP;EGLN2;EIF1;EIF2B3;EIF2S2;EIF3K;EIF5A;EIF5B;ERGIC3;EWSR1;EXOSC2;EXOSC5;EXOSC8;FARSB;FKBP2;FTSJ3;GMNN;GSS;HIGD2A;HINT1;HIST1H1C;HNRNPA1;HNRNPL;HS

								;51548;84335;4150;7922;55017;8125;11068;2286;84759;826;64965;10376;22948;4702;203260;9025;1164;51053;51096;25864;5605;1072;4869;79031;9409;9482;27095;58485;117246;10467;54896;6129;112398;126299;84311;5877;5451;79177;10682;1984;79022;131474;5757;84885;199746;8189;6748;23028;8891;337867;51368;2648;56257;9669;9419;9526;27077;23404;57215;5250;51389;11186;1649;5833;55210;7384;10058;6257;3006;9270;79759;6625;5201;10055;55856;572;3191;53615;56658;9973;23649;133957;5876;9524;5111;5261;11020;1174;8904;26986;7283;25776;400569;899;3329;7390;10190;6790;7447;26100;3421;2937;94086;10121;9352;2130;10066;23546;7709;80776;5281;119504;28987;219790;9167;10284;7347;91574;8574;6993;55324;8558;6598;90324;51247	PB9;HSPD1;HSPE1;IDH3G;IFT27;INO80C;ITGB1BP1;JAGN1;KAT2A;KDM1A;LIN37;LSM2;MAP2K2;MAZ;MBD3;MCRS1;MED11;MEPCE;MLST8;MPDU1;MRPL14;MRPL17;MRPL22;MRPL36;MRPL45;MRPS18B;MRPS9;MXD3;NDUFA13;NDUFA8;NDUFS6;NOB1;NOP16;NPM1;NPRL2;NR2C2AP;PABPC1;PAIP2;PCGF1;PCNA;PCYT2;PDCD2L;PDCL3;PEX16;PFDN1;PGLS;PHF1;PHKG2;PIGF;POLA2;POLR2F;POLR2H;POP7;POU2F1;PQLC2;PSENEN;PSMA4;PSMC5;PTMA;QPCTL;RABGGTB;RABIF;RASSF1;RFXANK;RNF8;RPL5;RPL7;RPS16;RPS23;RPS29;RRP9;RTKN2;RWDD1;RXRB;SAE1;SAP18;SCAMP2;SCAMP3;SIRT6;SLC25A3;SLC39A7;SMARCB1;SNRNP70;SNRPD2;SSR4;STX8;SYMPK;SYNGR4;TDP2;TECR;TEX264;THAP11;TMEM106C;TMEM115;TOMM7;TRAPPC1;TRAPPC3;TRIM28;TRIM39;TSEN54;TUBA1B;TUBG1;TXNDC9;TXNL1;U2AF1L4;UBA52;UBAC2;UBE2M;UCHL3;UQCR10;UQCRC1;UROS;UTP18;VSNL1;WIPI2;XPO5;ZBTB17;ZDHHC12;ZNF428;ZNF576;ZNF668;ZNF692;ZNHIT1
ACTAY	http://www.b	0.38536	1.368724	0	0.106	408	105	79897;63943;10919;5036;6205;	AAMP;ACTR1A;ALKBH4;AP2M1;AP2S1

RNNNCCCR_UNKNOWN	roadinstitute.org/gsea/msigdb/cards/ACTAYRNNNCCCR_UNKNOWN				402			6233;10330;6128;1175;8890;55851;81858;6138;10641;10067;23583;54958;79228;81490;29099;1350;27339;5510;140465;84232;51451;283899;29796;10535;10799;5704;83461;55135;119710;54344;54985;84153;11068;3925;54784;9296;55192;83743;91272;5546;57405;4913;1072;6129;51024;150274;116541;2288;51058;124790;199746;29100;1173;2648;57215;8318;993;5833;2189;10540;84936;55663;10567;113878;14;4351;2237;56926;6199;9789;9156;1349;2224;56931;26986;400569;81688;7023;353355;200186;5905;26145;94086;84315;10121;9784;50485;8078;4793;5557;3978;24140;23558;990;22934;165545;246184;124817;27352;84955	;ATP6V1F;BOD1;C11orf74;C6orf62;CDC25A;CDC26;CDC45;CDC6;CDCA3;CFL1;CNPY2;CNTD1;COMMD9;COX7B;COX7C;CRTC2;CYB561D2;DCTN2;DNAJC17;DPM3;DQX1;DTX2;DUS3L;EHMT2;EIF2B4;EXO1;FANCG;FDPS;FEN1;FIS1;FKBP4;FKBPL;FTSJ1;GRWD1;HCFC1R1;HEXIM2;HSCB;HSPB9;INO80E;IRF2BP1;KAT2A;LCMT1;LIG1;MAF1;MED11;MON1A;MPI;MRPL54;MYL6B;NCLN;NFKBIB;NPRL2;NTHL1;NUDCD1;PA2G4;PABPC1;PCYT2;PPP1R7;PRCC;PRIM1;PRPF19;PSENEN;PSMC4;PTDSS2;RABAC1;RANGAP1;RNASEH2A;RNASEH2C;RPIA;RPL15;RPL6;RPL7;RPP21;RPP40;RPS11;RPS27A;RPS6KB2;SCAMP3;SGSM3;SHARPIN;SMARCAL1;SMUG1;SNX17;SPC25;SPCS2;STMN1;TFAP4;THAP11;THOC6;TMEM160;TMEM208;U2AF1L4;UQCR10;USP5;WBP2;WRAP53;ZFYVE19;ZNF233;ZNF446;ZNF691
V$CETS1P54_01	http://www.broadinstitute.org/gsea/msigdb/cards/V$CETS1P54	0.402959	1.349853	0.014742	0.111137	234	44	1460;6222;6181;6628;5719;122704;51070;200185;4738;54663;51651;51645;1163;6128;81502;27339;5531;10063;56993;11313;51506;6903;30968;7388;79797	AKT1S1;B3GAT3;CKS1B;COX17;CSNK2B;DPCD;EBNA1BP2;EIF5A;FGFR4;GPN2;HM13;INO80B;ITGB3BP;KRTCAP2;LYPLA2;MCTS1;MED8;MRPL52;NEDD8;NOSIP;POLL;PPIL1;PPP4C;PRPF19;PSMD13

	_01							;25911;84335;27343;112950;6154;140739;5721;10376;56949;58485;26229;10969;1984;6293;2264;23421;28985;54707;83444	;PSME2;PTRH2;RPL26;RPL6;RPLP2;RPS18;SNRPB;STOML2;TBCC;TOMM22;TRAPPC1;TUBA1B;UBE2F;UFC1;UQCRH;VPS52;WDR74;XAB2;ZNF408
GKCGCNNNNNNNTGAYG_UNKNOWN	http://www.broadinstitute.org/gsea/msigdb/cards/GKCGCNNNNNNNTGAYG_UNKNOWN	0.495203	1.372634	0.052392	0.111521	52	12	11267;25855;2197;8409;51451;9616;11137;51726;5690;79080;10921;84844	BRMS1;CCDC86;DNAJB11;FAU;LCMT1;PHF5A;PSMB2;PWP1;RNF7;RNPS1;SNF8;UXT
AACYNNNNTTCCS_UNKNOWN	http://www.broadinstitute.org/gsea/msigdb/cards/AACYNNNNTTCCS_UNKNOWN	0.447746	1.356065	0.036036	0.112285	86	21	6147;6233;55851;25886;23564;27339;5704;119710;199746;7866;14;4351;4968;1349;200186;50485;5557;2931;25876;8819;4141	AAMP;C11orf74;COX7B;CRTC2;DDAH2;GSK3A;IFRD2;MARS;MPI;OGG1;POC1A;PRIM1;PRPF19;PSENEN;PSMC4;RPL23A;RPS27A;SAP30;SMARCAL1;SPEF1;U2AF1L4
V$E2F_Q6_01	http://www.broadinstitute.org/gsea/msigdb/cards/V$E2F_Q6_01	0.405225	1.339557	0.01139	0.115579	219	53	4201;116138;83463;339487;10330;22913;6223;22827;126328;3178;10204;54949;1616;5902;57510;3151;6839;4150;55017;79077;3925;6811;51053;3015;2597;29101;4176;9088;5888;1984;5757;5424;27037;84320;8318;99	ACBD6;AP4M1;C14orf119;CDC25A;CDC45;CDC6;CNPY2;DAXX;DCTPP1;E2F1;EIF5A;ETV4;EZH2;FANCG;GAPDH;GMNN;H2AFZ;HMGN2;HNRNPA1;KLHDC3;LIG1;MAZ;MCM3;MCM7;MEA1;MXD3;NASP;NDUFA11;NUTF2;PCNA;PCYT2;PKMYT1;POLD1;POLE2;PRIM1;PRKCSH;PT

								3;5833;3516;4172;2189;9179;1869;5111;4678;6241;54962;2146;5589;5557;3978;990;2118;5427	MA;PUF60;RAD51;RALY;RANBP1;RBPJ;RPS19;RRM2;SDHAF2;SSU72;STMN1;STX5;SUV39H1;TIPIN;TRMT2A;XPO5;ZBTB8OS
V$GABP_B	http://www.broadinstitute.org/gsea/msigdb/cards/V$GABP_B	0.39413	1.333411	0.004728	0.116328	242	68	1460;6161;10436;9587;7266;51035;8815;54663;54460;6881;116092;10452;10067;23658;54949;55850;6164;5902;5689;56993;1337;6839;7388;8667;55696;10421;64419;51371;55017;84759;140739;64601;26229;6992;10969;9158;1984;10262;6748;84285;29100;2958;27037;9419;26155;83590;57060;10921;1649;11007;140686;51248;113878;56658;1650;6829;84124;23478;51010;28511;24137;6790;3421;64925;10066;2783;5281;25839	AURKA;B3GAT3;BANF1;C14orf119;CCDC71;CCDC85B;CD2BP2;COG4;COX6A1;CRIPT;CSNK2B;DDIT3;DDOST;DNAJC7;DNTTIP1;DTX2;EBNA1BP2;EIF1AD;EIF3H;EIF5A;EMG1;EXOSC3;FIBP;GNB2;GTF2A2;IDH3G;KIF4A;LSM5;MAD2L1BP;MRPS21;MTMR14;NKIRAS2;NOC2L;PCBP4;PCGF1;PDZD11;PIGF;POMP;PPP1R11;PSMB1;RANBP1;RBM22;RNPS1;RPL32;RPL34;SCAMP2;SCAMP3;SDHAF2;SEC11A;SF3B4;SSR4;SUPT5H;SUV39H1;TAF10;TMEM208;TMUB1;TOMM22;TOMM40;TRIM39;TRMT2A;UBE2F;UBXN1;UQCRH;USE1;VPS16;WDR74;WFDC3;ZNF394
GGAMTNNNNNTCCY_UNKNOWN	http://www.broadinstitute.org/gsea/msigdb/cards/GGAMTNNNNNTCCY_UNKNOWN	0.4501	1.373	0.014493	0.123392	104	20	1460;4201;116138;5036;26528;10204;79590;11070;283899;119710;84335;3925;1072;8189;55182;2237;56926;51205;26145;84315	ACP6;AKT1S1;C11orf74;CFL1;CSNK2B;DAZAP1;FEN1;INO80E;IRF2BP1;KLHDC3;MEA1;MON1A;MRPL24;NCLN;NUTF2;PA2G4;RNF220;STMN1;SYMPK;TMEM115

KRCTCNNNNMANAGC_UNKNOWN	http://www.broadinstitute.org/gsea/msigdb/cards/KRCTCNNNNMANAGC_UNKNOWN	0.465225	1.320625	0.065678	0.125285	63	20	84759;221504;8338;11065;3006;3024;8336;8352;85235;8354;3009;8351;3008;8335;3017;8358;85236;8341;8331;3012	HIST1H1A;HIST1H1B;HIST1H1C;HIST1H1E;HIST1H2AB;HIST1H2AE;HIST1H2AH;HIST1H2AJ;HIST1H2AM;HIST1H2BD;HIST1H2BK;HIST1H2BN;HIST1H3B;HIST1H3C;HIST1H3D;HIST1H3I;HIST2H2AC;PCGF1;UBE2C;ZBTB9
TGCGCANK_UNKNOWN	http://www.broadinstitute.org/gsea/msigdb/cards/TGCGCANK_UNKNOWN	0.367633	1.314386	0.002404	0.126399	485	114	4201;116138;6633;8625;83658;6143;9045;83463;7311;6217;54543;51567;200185;5685;7203;51329;6125;27335;28973;10248;51192;84661;9040;3178;1603;29093;8894;10641;50626;8893;11066;10445;3094;5252;54814;10209;7391;10557;11068;2286;84759;9296;1164;2879;64147;25864;4714;9049;203054;10467;9088;84311;5877;5451;51174;124790;4700;1984;403;5757;10987;11335;3646;2648;83858;56257;27077;348793;54901;5833;3516;55210;10817;113878;79759;6625;5201;10055;55856;3191;133957;51060;5876;9524;11020;1174;5683;10263;26986;7283;4	ABHD14A;ACOT13;ACTR1A;ADCK5;AIP;AP1S1;ARL3;ARL6IP4;ATAD3A;ATAD3B;ATP6V1F;AURKA;B9D1;C12orf65;CALM3;CBX3;CCDC127;CCDC97;CCT3;CDK2AP2;CDKAL1;CKLF;CKS2;COPS5;COX7A2L;CYB561D2;CYHR1;DAD1;DLEU1;DPY30;DTX2;DYNLRB1;DYNLT1;E2F6;EIF1;EIF2B5;EIF2S2;EIF3E;EIF3K;EIF5A;EWSR1;FKBP2;FLYWCH2;FRS3;GPX4;HEXIM2;HINT1;HNRNPA1;HNRNPL;HSPB9;IFT27;KAT2A;KIF9;KIFC2;KLHDC3;KRTCAP2;LSM12;MCRS1;MEA1;MED11;MEPCE;MRPL22;MRPL45;MRPS18B;MXD3;NDRG1;NDUFA6;NDUFB8;NPRL2;OTUD5;PABPC1;PCGF1;PCYT2;PFDN1;PHF1;PKMYT1;POP7;POU2F1;PSMA2;PSMA4;PTMA;QPCTL;RABGGTB;RABIF;RBPJ;RFXANK;RPL14;RPL19;RPL5;RPL7L1

								00569;7023;6790;808;90990;7447;285855;124801;94086;55593;10121;2130;23546;92335;25809;9167;91574;114984;6993;6598;90324;10301;10397;1876	;RPP38;RPS16;SAE1;SMARCB1;SNRNP35;SNRNP70;SNRPD2;STRADA;SYNGR4;TDP2;TECR;TFAP4;TOMM7;TTLL1;TUBD1;TUBG1;TXNDC12;UBA52;UBE2M;USF1;VSNL1;WDR53;ZNF668;ZNHIT1
MGGAAGTG_V$GABP_B	http://www.broadinstitute.org/gsea/msigdb/cards/MGGAAGTG_V$GABP_B	0.353407	1.30111	0	0.129753	699	133	6155;5437;6222;51504;6167;6882;10436;4830;5719;9587;3921;55011;6205;7266;4738;51035;54663;51651;1163;8409;4831;55851;28973;10574;6015;10452;25824;6171;10093;5695;3159;23658;81502;55850;6194;5902;283899;56342;5689;56616;57510;28991;79159;10063;6396;51506;3326;6903;7388;84292;54433;79797;6949;4722;25911;7391;8086;27343;55696;10421;64419;51371;55017;84759;140739;1716;10519;64601;51076;10960;83743;8992;5984;581;2067;23647;89891;51024;84261;6992;10474;9158;25904;1984;54758;10262;199746;6293;2079;1973;5585;7341;29100;2958;27037;9526;7332;57215;26155;8480;25828;83590;8318;80153;10921;164	AAAS;ARFIP2;ARPC4;ATP6V0E1;AURKA;BAX;C14orf119;C1orf122;CBX8;CCDC85B;CCT7;CD2BP2;CDC45;CDK5;CIB1;CKS1B;CNOT10;COMMD5;COPS3;COX17;CUTC;DDIT3;DDOST;DGUOK;DIABLO;DNAJC7;DPCD;EDC3;EIF4A1;EIF5A;EMG1;ERCC1;ERH;FBXW9;FIBP;FIS1;GAR1;GLA;GRWD1;GTF2A2;HM13;HMGA1;HSP90AB1;INO80E;KLHDC4;LMAN2;LSM5;MAD2L1BP;MPDU1;MRPS18B;MTMR14;NASP;NDUFS3;NEDD8;NKIRAS2;NME1;NME2;NOC2L;NOL12;PCGF1;PIGC;PIH1D1;PKN1;POLL;POLR2H;POMP;PPAN;PPP1R11;PRDX5;PRIM2;PSENEN;PSMB1;PSMB7;PSMD13;PTRH2;RAE1;RANBP1;RBM22;RFC4;RGL2;RGS14;RING1;RNPS1;RPA2;RPL27;RPL37;RPL41;RPS11;RPS18;RPS6;RPSA;SEC11A;SEC13;SF3B4;SIRT3;SLC9A3R1;SUMO1;TACO1;TADA3;TAF11;TBCC;TCOF1;THAP11;TMEM208;TMUB1;TOMM40;TRIM39;TRIP13;TR

								9;1020;11007;7284;7336;79759;57332;9319;23410;5558;56658;8673;10636;51060;51204;8533;1650;127687;9368;23478;4678;28511;5863;6790;5279;6118;26100;2717	MT112;TRMT2A;TUFM;TXN2;TXNDC12;U2AF1L4;UBE2F;UBE2L3;UBE2V2;UBXN1;UFC1;UQCRH;USE1;USF1;UXT;VAMP8;VPS16;VPS52;WDR34;WDR74;WDR83;WIPI2;XPO5;ZNF408;ZNF668
V$MYC_Q2	http://www.broadinstitute.org/gsea/msigdb/cards/V$MYC_Q2	0.40015	1.304991	0.022989	0.131571	173	30	5036;200185;51035;51154;26519;3178;10726;6146;5441;23378;5202;51042;23412;3151;3020;3925;533;1072;10969;126792;403;79084;8704;51303;83858;4904;3611;55210;30836;51060	ARL3;ATAD3A;ATAD3B;ATP6V0B;B3GALT6;B4GALT2;CFL1;COMMD3;DNTTIP2;EBNA1BP2;FKBP11;H3F3A;HMGN2;HNRNPA1;ILK;KRTCAP2;MRTO4;NUDC;PA2G4;PFDN2;POLR2L;RPL22;RRP8;STMN1;TIMM10;TXNDC12;UBXN1;WDR77;YBX1;ZNF593
V$USF2_Q6	http://www.broadinstitute.org/gsea/msigdb/cards/V$USF2_Q6	0.381363	1.282454	0.03271	0.149029	235	41	5036;339487;200185;51035;22818;51154;26519;3178;2647;10067;10726;6146;5441;23378;5202;51042;84975;23412;3151;10623;3020;3925;2597;533;1072;10969;126792;402;403;79084;8704;51303;83858;11124;4904;3611;55210;2237;30836;5883;51060	ARL2;ARL3;ATAD3A;ATAD3B;ATP6V0B;B3GALT6;B4GALT2;BLOC1S1;CFL1;COMMD3;COPZ1;DNTTIP2;EBNA1BP2;FAF1;FEN1;FKBP11;GAPDH;H3F3A;HMGN2;HNRNPA1;ILK;KRTCAP2;MFSD5;MRTO4;NUDC;PA2G4;PFDN2;POLR2L;POLR3C;RAD9A;RPL22;RRP8;SCAMP3;STMN1;TIMM10;TXNDC12;UBXN1;WDR77;YBX1;ZBTB8OS;ZNF593
CACGTG_V$MYC_Q2	http://www.broadinstitute.org/gsea/msigdb/cards/C	0.329642	1.234904	0	0.23366	951	168	4201;10471;9277;116138;5437;5691;2794;6143;2091;6156;56915;83463;5036;8662;339487;51264;6205;27166;6626;200185;9	AATF;ABCB6;AIFM3;ALDOA;APEX1;ARL2;ARL3;ASNA1;ASPSCR1;ATAD3A;ATAD3B;ATF4;ATIC;ATP6V0B;B3GALT6;B4GALT2;BAX;BCL7C;BLOC1S1;CEBPA

	ACGTG_V$MYC_Q2							2609;51035;26574;10330;22818;80742;10528;6187;9274;51154;6223;26519;26520;3178;2647;10067;3159;2873;10726;471;6146;23481;23521;1933;5441;3336;23378;10695;5902;56616;5202;53917;57510;51042;84975;146956;23412;9521;4701;3326;3151;80142;54433;1201;6949;64976;80324;10623;7922;3020;79077;3925;64601;83985;11337;3015;2597;533;51096;4913;581;51602;1072;4869;11065;5216;112398;9343;10969;7873;55646;1678;2870;126792;3276;402;403;5757;79058;25879;79084;10669;1973;5585;220988;3646;83475;7866;8704;51303;27037;468;83858;56257;11124;2876;83590;81890;162979;29886;4904;3611;55210;10058;6257;29078;90701;140825;80758;10055;2237;30836;5883;51333;23593;51060;23212;3308;226;2719;26986;56888;7023;328;3329;55644;10043;124044;6666;92579;10642;	;CFL1;CGREF1;CLN3;CNPY2;CNPY3;COMMD3;COPZ1;DCAF13;DCTPP1;DIABLO;DLX2;DNTTIP2;DOHH;EBNA1BP2;EEF1B2;EEF1E1;EFTUD2;EGLN2;EIF3B;EIF3E;EIF4A1;EME1;EWSR1;EXOSC5;FAF1;FBL;FEN1;FKBP11;G6PC3;GABARAP;GAPDH;GAR1;GLA;GNB2;GNL1;GPC3;GPS1;GPX1;GRK6;H2AFZ;H3F3A;HEBP2;HMGA1;HMGN2;HNRNPA1;HNRNPA3;HNRNPM;HSP90AB1;HSPA4;HSPD1;HSPE1;IFRD2;IGF2BP1;ILK;KCMF1;KLHDC3;KRTCAP2;LYAR;MANF;MEA1;MEPCE;MFSD5;MON1A;MRPL27;MRPL40;MRTO4;MXD3;NDUFA7;NDUFAF4;NEURL2;NOP56;NOP58;NPM1;NTHL1;NUDC;OSGEP;PA2G4;PABPC1;PES1;PFDN2;PFDN6;PFN1;PKN1;POLR2H;POLR2L;POLR3C;PRELID1;PRMT1;PRR3;PRR7;PSMB3;PTGES2;PTMA;PUS1;QTRT1;RAB24;RAD9A;RANBP1;RBM3;RHEBL1;RPIA;RPL13A;RPL19;RPL22;RPL30;RPS11;RPS19;RPS2;RRP8;RRS1;RXRB;SAE1;SCAMP3;SEC11C;SLC39A7;SLC43A1;SNRPA;SNX8;SOX12;SPATA2L;SPNS1;STMN1;TCOF1;TFAP4;TIMM10;TIMM50;TIMM8A;TIMM9;TMUB1;TOM1;TRMT2A;TSR2;TXNDC12;UBE2

								90121;1050;2717;84315;10813;57176;2130;2783;5935;8501;121268;1746;23558;22934;150209;4670;439	C;UBXN1;UTP14A;UTP18;VARS2;VPS16;WBP2;WDR46;WDR77;XPO5;YBX1;ZBTB8OS;ZNF296;ZNF593;ZNF771
SGCGSSAAA_V$E2F1DP2_01	http://www.broadinstitute.org/gsea/msigdb/cards/SGCGSSAAA_V$E2F1DP2_01	0.378093	1.219984	0.072072	0.247717	155	39	6224;83463;6632;3159;5902;6839;64976;4150;79077;199745;51053;3015;4176;56655;9088;5757;5424;27037;56257;84320;993;4172;2189;9179;84844;284403;1869;56658;5111;1174;85235;4678;4439;7023;6241;23234;23546;990;5427	ACBD6;AP1S1;AP4M1;CDC25A;CDC6;DCTPP1;DNAJC9;E2F1;FANCG;GMNN;H2AFZ;HIST1H2AH;HMGA1;MAZ;MCM3;MCM7;MEPCE;MRPL40;MSH5;MXD3;NASP;PCNA;PHF5A;PKMYT1;POLD1;POLE2;POLE4;PTMA;RANBP1;RPS20;RRM2;SNRPD1;SUV39H1;SYNGR4;TFAP4;THAP8;TRIM39;TRMT2A;WDR62
GCGNNANTTCC_UNKNOWN	http://www.broadinstitute.org/gsea/msigdb/cards/GCGNNANTTCC_UNKNOWN	0.399585	1.220208	0.092715	0.258399	112	22	6631;4795;6633;6882;22827;158;3336;56616;283989;54814;147807;84335;94107;11337;6727;4176;122509;1973;11335;7341;10540;9179	ADSL;AKT1S1;AP4M1;CBX3;DCTN2;DIABLO;EIF4A1;GABARAP;HSPE1;IFI27L1;MCM7;NFKBIL1;PUF60;QPCTL;SNRPC;SNRPD2;SRP14;SUMO1;TAF11;TMEM203;TSEN54;ZNF524
V$COUP_DR1_Q6	http://www.broadinstitute.org/gsea/msigdb/cards/V$COUP_DR1_Q6	0.356138	1.190198	0.066826	0.295127	221	59	5437;65003;10436;79005;1163;6048;10005;1933;3336;65010;29796;23412;30968;55135;10554;64223;112950;10322;9618;9409;5216;199746;8189;83719;79885;57060;55937;2063;10540;80758;28957;115811;9491;23381	ACOT8;ACTA1;AGPAT1;APOM;BCAM;CCDC71;CKS1B;COMMD3;DCTN1;DCTN2;EEF1B2;EMG1;FABP1;FOXA3;HDAC11;HSD17B8;HSPD1;HSPE1;IQCD;LMAN2L;MED8;MLST8;MRPL11;MRPS28;NR2F6;NSUN5P2;NUDCD1;PCBP4;PEX16;PFN1;PHB2;POLR2H;POU5F1;PPP1R14D;PRD

								;3329;140831;64925;51246;5589;10284;283375;84955;84283;3171;7066;260294;81562;1639;4059;7923;5460;59336;54866;11331;7169;54926;2168;58510;58	M13;PRKCSH;PRODH2;PRR7;PSMF1;RNF5;SAP18;SCNM1;SHISA5;SLC26A6;SLC39A5;SMG5;SMYD5;STOML2;SYMPK;THPO;TMEM79;TPM2;TRAF4;U2AF1L4;UBE2R2;UQCR10;WRAP53;YPEL3;ZSWIM3
TMTCGCGANR_UNKNOWN	http://www.broadinstitute.org/gsea/msigdb/cards/TMTCGCGANR_UNKNOWN	0.378158	1.194002	0.09434	0.295783	149	40	9533;6201;6210;4736;6223;63906;22827;126328;92703;84316;6194;6136;6139;5052;6839;80764;6154;1716;318;10024;5690;221150;79084;6748;51657;3516;10540;25926;3308;1349;6829;23567;23381;3421;8562;10284;11011;4191;8402;84283	COX7B;DCTN2;DENR;DGUOK;GPATCH3;HSPA4;IDH3G;MDH2;NAA38;NDUFA11;NOL11;NUDT2;POLR1C;PRDX1;PSMB2;PUF60;RBPJ;RPL10A;RPL12;RPL17;RPL26;RPS15A;RPS19;RPS6;RPS7;SAP18;SKA3;SLC25A11;SMG5;SSR4;STYXL1;SUPT5H;SUV39H1;THAP7;TLK2;TMEM183A;TMEM79;TROAP;WDR77;ZNF346
